# Supplementary material for: Copper(I)-catalysed site-selective C(sp3)–H bond chlorination of ketones, (E)-enones and alkylbenzenes by dichloramine-T
Source: Nat Commun. 2021 Jul 1;12:4065. doi: 10.1038/s41467-021-23988-y (PMC8249392; doi:10.1038/s41467-021-23988-y)
Supplement: Supplementary file 1 — Supplementary Information [file 41467_2021_23988_MOESM1_ESM.pdf]

Supplementary Information

for

Copper(I)-Catalysed Site-Selective C(sp^3)–H Bond Chlorination of Ketones, (*E*)-Enones and Alkylbenzenes by Dichloramine-T

Jianwen Jin,[1,5] Yichao Zhao,[1,5] Sara Helen Kyne,[1] Kaveh Farshadfar,[2] Alireza Ariafard,[2,3]* and Philip Wai Hong Chan[1,4]*

[1]School of Chemistry, Monash University, Clayton, Victoria 3800, Australia

[2]Department of Chemistry, Islamic Azad University, Poonak, Tehran, Iran

[3]School of Natural Sciences–Chemistry, University of Tasmania, Hobart, Tasmania 7001, Australia

[4]Department of Chemistry, University of Warwick, Coventry CV4 7AL, United Kingdom

[5]These authors contributed equally to this work

Email: phil.chan@monash.edu (P. W H. C.); alireza.ariafard@utas.edu.au (A. A.)

Table of Contents

1. Supplementary Methods. **3**

2. Experimental Procedures. **4**

2.1 General Procedure A for the Preparation of Substrates (1b–1d, 1f– 1g).

2.2 General Procedure B for the Preparation of Substrates (1e, 1k–1x).

2.3 General Procedure C for the Preparation of Substrates (1y–1α).

2.4 General Procedure D for the Preparation of Substrates (3a–3l).

2.5 General Procedure E for the Preparation of Substrates (5i–5k, 9s–13s).

2.6 General Procedure F for the Optimisation of the Reaction Conditions for the Chlorination of Substrate 1a.

2.7 General Procedure G for the Copper(I)-Catalysed Site-Selective Chlorination of the Tertiary C(sp^3)–H Bond of Ketones 1.

2.8 General Procedure H for the Copper(I)-Catalysed Site-Selective Chlorination of the Secondary C(sp^3)–H Bond of Ketones 1.

2.9 General Procedure I for Copper(I)-Catalysed Site-Selective Chlorination of the Allylic C(sp^3)–H Bond of (*E*)-Enones 3.

2.10 General Procedure J for the Copper(I)-Catalysed Site-Selective Chlorination of the Benzylic C(sp^3)–H Bond of Alkylbenzenes 5.

2.11 General Procedures K for the Synthesis of Deuterated Substrates.

2.12 General Procedures for the Control Experiments of Fig. 3.

2.13 Gram-Scale Synthesis of Nonpeptide δ-Opioid Agonist 7.

3. Compound Characterisation Data. **27**

4. ORTEP Drawings. **71**

5. Computational Details. **72**

6. ^{1}H and ^{13}C NMR Spectra. **81**

7. References. **190**

1. Supplementary Methods.

General laboratory procedures. All reactions were performed in oven-dried glassware under a nitrogen atmosphere fitted with rubber septa. Flash column chromatography was performed using silica gel and a gradient solvent system (*n*hexane:EtOAc as eluent).

Materials and instrumentation. Unless specified, all non-commercially available compounds were prepared and characterised as described in the Experimental Procedures section. All other substrates and reagents were purchased from commercial sources. Anhydrous acetonitrile was freshly distilled from calcium hydride. Analytical thin layer chromatography (TLC) was performed using pre-coated silica gel plate with visualisation was achieved by UV light (254 nm). ^{1}H and ^{13}C NMR spectra were recorded on 300 and 400 MHz spectrometers. Chemical shifts (ppm) were recorded with tetramethylsilane (TMS) as the internal reference standard. Multiplicities are given as: s (singlet), br s (broad singlet), d (doublet), t (triplet), dd (doublet of doublets) or m (multiplet). The number of protons (*n*) for a given resonance is indicated by *n*H and coupling constants are reported as a *J* value in Hz. Infrared spectra were taken on a IR spectrometer. High-resolution mass spectra (HRMS) were obtained on a LC/HRMS TOF spectrometer using simultaneous electrospray (ESI).

Abbreviations. byp = 2,2′-bypyiridine, 1,10-phen = 1,10-phenanthroline, pybox = 2,6-bis((3a*R*,8a*S*)-3a,8a-dihydro-8*H*-indeno[1,2-*d*]oxazol-2-yl)pyridine, Xantphos = 4,5-bis(diphenylphosphino)-9,9-dimethylxanthene, NCS = *N*-chlorosuccinimide, DCDMH = 1,3-dichloro-5,5-dimethylhydantoin, TCCA = trichloroisocyanuric acid, TEMPO = (2,2,6,6-tetramethylpiperidin-1-yl)oxyl, BHT = dibutylhydroxytoluene, TOF = turnover frequency.

2. Experimental Procedures.

2.1 General Procedure A for the Preparation of Substrates (1b–1d, 1f–1j).

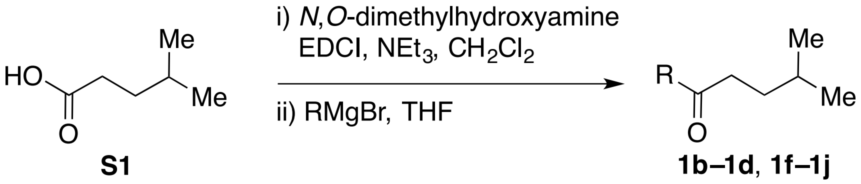

Substrates (**1b–1d, 1f–1j**) were prepared following literature procedures.[1] To a solution of 4-methylpentanoic acid **S1** (1.16 g, 10.0 mmol), and *N*,*O*-dimethylhydroxyamine hydrochloride (1.03 g, 10.5 mmol) in CH_2Cl_2 (20 mL) was sequentially added Et_3N (1.21 g, 1.6 mL, 12.0 mmol) and 1-ethyl-3-(3-dimethyllaminopropyl)carbodiimide hydrochloride (1.63 g, 10.5 mmol) at room temperature. The resultant mixture was stirred for 12 h at room temperature before being quenched with water (30 mL). The phases were separated and the aqueous phase extracted with CH_2Cl_2 (2 × 20 mL). The combined organic phases were sequentially washed with HCl (1 M, 2 × 20 mL), saturated solution of $NaHCO_3$ (2 × 20 mL) and brine (20 mL). The resulting organic phase was dried (Na_2SO_4) and concentrated *in vacuo* to give the crude Weinreb amide as a colourless oil (1.59 g, >99% yield). The crude Weinreb amide was directly used for the next step without any further purification.

To a solution of crude Weinreb amide (0.32 g, 2.0 mmol) in THF (5 mL) was slowly added the Grignard reagent (RMgBr, 3 equiv.) at 0 °C. The resulting mixture was stirred for 12 h at room temperature and quenched with NH_4Cl solution (5 mL). The phases were separated and the aqueous phase extracted with EtOAc (2 × 5 mL). The combined organic phases were washed with brine (5 mL), dried over Na_2SO_4 and concentrated *in vacuo*. Purification by flash column chromatography on silica gel (eluent: petroleum ether/EtOAc) provided the title compound (**1b–1d, 1f–1j**).

2.2 General Procedure B for the Preparation of Substrates (1e, 1k–1x).

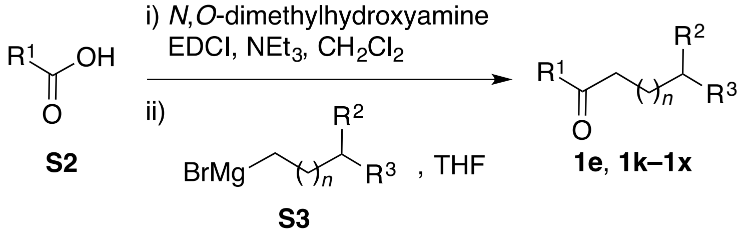

To a solution of carboxyl acid **S2** (2.0 mmol), and *N*,*O*-dimethylhydroxyamine hydrochloride (0.2 g, 2.05 mmol) in CH_2Cl_2 (5 mL) was sequentially added Et_3N (0.21 g, 0.28 mL, 2.1 mmol) and 1-ethyl-3-(3-dimethyllaminopropyl) carbodiimide hydrochloride (0.32 g, 2.05 mmol) at room temperature. The resultant mixture was stirred for 12 h at room temperature and quenched with water (5 mL). The phases were separated and the aqueous phase extracted with CH_2Cl_2 (2 × 5 mL). The combined organic phases were sequentially washed with HCl (1 M, 2 × 5 mL), saturated solution of $NaHCO_3$ (2 × 5 mL) and brine (5 mL). The resulting organic phase was dried over Na_2SO_4 and concentrated *in vacuo* to give the crude Weinreb amide. The crude Weinreb amide was directly used for the next step without any further purification.

To a solution of the crude Weinreb amide (2.0 mmol) in THF (5 mL) was slowly added the Grignard reagent **S3** (3 equiv.) at 0 °C. The resultant mixture was stirred for 12 h at room temperature and quenched with NH_4Cl solution (5 mL). The phases were separated and the aqueous phase extracted with EtOAc (2 × 5 mL). The combined organic phases were washed with brine (5 mL), dried over Na_2SO_4 and concentrated *in vacuo*. Purification by flash column chromatography on silica gel (eluent: petroleum ether/EtOAc) provided the title compound (**1e**, **1k–1x**).

2.3 General Procedure C for the Preparation of Substrates (1y–1α).

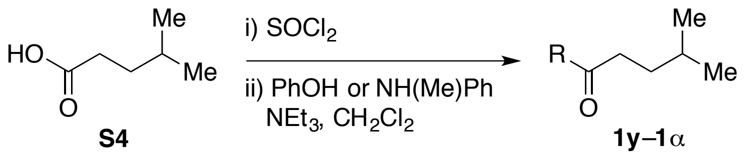

4-Methylpentanoic acid (0.46 g, 4.0 mmol) was added with thionyl chloride (0.98 g, 0.58 mL, 8 mmol) at room temperature and the resultant mixture was heated to 50°C for 1 h. On completion, excess thionyl chloride was removed under reduced pressure to give the crude reaction mixture. Without further purification, to a solution of the crude reaction mixture and phenol (0.53 mL, 6 mmol) or 2-(2-hydroxyethyl)isoindoline-1,3-dione (1.15 g, 6 mmol) or *N*-methylaniline (0.65 mL, 6 mmol) in CH_2Cl_2 (5 mL) was added Et_3N (1.21 g, 1.6 mL, 12.0 mmol) at room temperature. The resultant mixture was stirred for 12 h at room temperature and quenched with water (5 mL). The phases were separated and the aqueous phase extracted with CH_2Cl_2 (2 × 10 mL). The combined organic phases were sequentially washed with HCl (1 M, 2 × 10 mL), and brine (10 mL). The resulting organic phase was dried over Na_2SO_4 and concentrated *in vacuo* to give the crude product. Purification by flash column chromatography on silica gel (eluent: petroleum ether/EtOAc) provided the title compound.

2.4 General Procedure D for the Preparation of Substrates (3a–3l).

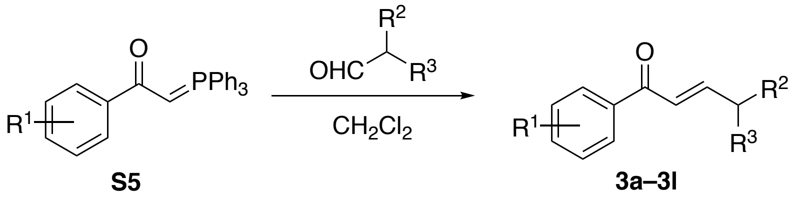

2-(Triphenylphosphoranylidene)acetophenone (**S5**) was prepared following literature procedures.[2] To a solution of compound **S5** (5.0 mmol) in CH_2Cl_2 (10 mL) was added the aldehyde (5 mmol). The resulting reaction mixture was stirred at room

temperature for 12 h. Upon completion, the solvent was removed under reduced pressure. The crude mixture was purified by flash column chromatography on silica gel (eluent: *n*hexane/Et_2O = 20/1) to furnish the title compound (**3a–3l**).

2.5 General Procedure E for the Preparation of Substrates (5i–5j, 9s–13s).

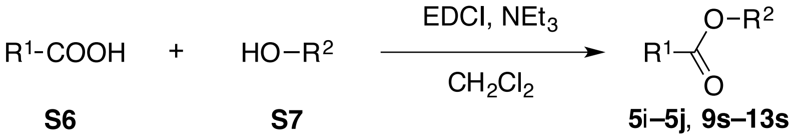

To a solution of carboxyl acid **S6** (2.0 mmol), and alcohol **S7** (2.1 mmol) in CH_2Cl_2 (5 mL) was sequentially added Et_3N (0.21 g, 0.28 mL, 2.1 mmol) and 1-ethyl-3-(3-dimethyllaminopropyl)carbodiimide hydrochloride (0.32 g, 2.05 mmol) at room temperature. The resultant mixture was stirred for 12 h at room temperature and quenched with water (5 mL). The phases were separated and the aqueous phase extracted with CH_2Cl_2 (2 × 5 mL). The combined organic phases were sequentially washed with HCl (1 M, 2 × 5 mL), saturated solution of $NaHCO_3$ (2 × 5 mL) and brine (5 mL). The organic phase was dried over Na_2SO_4 and concentrated *in vacuo* to give the crude reaction mixture. The crude reaction mixture was purified by flash column chromatography on silica gel (eluent: petroleum ether/EtOAc) to furnish the title compound (**5i–5j** and **9s–13s**).

2.6 General Procedure F for the Optimisation of the Reaction Conditions for the Chlorination of Substrate 1a.

To an oven-dried 10 mL round-bottom flask was added the substrate **1a** (35.2 mg, 0.2 mmol), CuOTf·0.5PhMe (5.2 mg, 0.02 mmol), ligand (0.02 mmol), $NaHCO_3$ (25.2 mg, 0.3 mmol) and 4 Å MS (50 mg). The reaction vessel was capped and charged with a nitrogen atmosphere through three cycles of the vacuum-nitrogen-backfill method over 10 min. A solution of acetonitrile (1 mL) containing dichloramine-T (72 mg, 0.3 mmol) was added and the resulting reaction mixture was stirred at room

temperature for 24 h. Upon completion, the reaction mixture was filtered through a Celite pad and washed with CH_2Cl_2 (3 × 1 mL). On removing the organic solvent *in vacuo*, the residue was analysed by ^{1}H NMR measurements to reveal the results described in Supplementary Table S1.

Supplementary Table 1. Solvent, ligand and chlorinating agent source on reactivity[a]

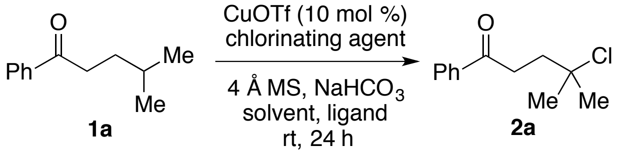

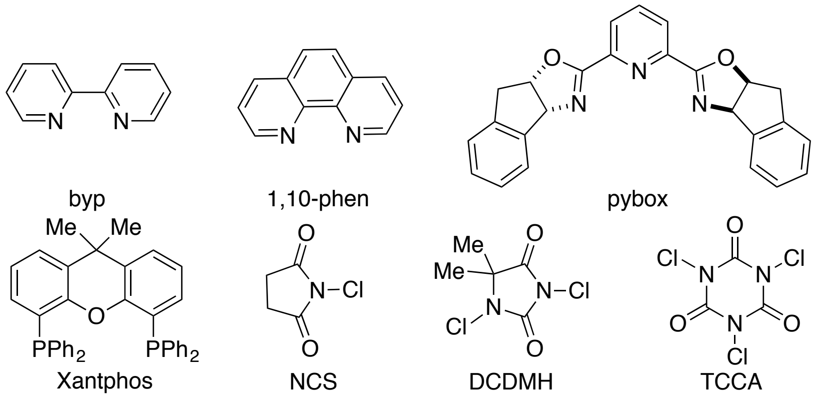

Entry	Solvent	Chlorinating Agent	Ligand	Yield (%)[b]
1	MeCN	$TsNCl_2$	byp	-[c]
2	MeCN	$TsNCl_2$	1,10-phen	-[c]
3	MeCN	$TsNCl_2$	pybox	-[c]
4	MeCN	$TsNCl_2$	Xantphos	-[c]
5	MeCN	TsNClNa	-[d]	-[c]
6	MeCN	NaOCl	-[d]	-[c]
7	MeCN	NCS	-[d]	-[c]
8	MeCN	DCDMH	-[d]	-[c]
9	MeCN	TCCA	-[d]	-[c]
10	MeOH	$TsNCl_2$	-[d]	-[c]
11	THF	$TsNCl_2$	-[d]	-[c]
12	1,3-Dioxane	$TsNCl_2$	-[d]	-[c]
13	$MeNO_2$	$TsNCl_2$	-[d]	-[c]
14	CH_2Cl_2	$TsNCl_2$	-[d]	-[c]
15	Toluene	$TsNCl_2$	-[d]	-[c]
16	PhCN	$TsNCl_2$	-[d]	41 (0.17)[f]
17[e]	MeCN	$TsNCl_2$	-[d]	48 (0.20)[f]

[a] All reactions were performed with **1a** (35.2 mg, 0.2 mmol), $TsNCl_2$ (72 mg, 0.3 mmol), CuOTf·0.5PhMe (5.2 mg, 0.02 mmol), ligand (0.2 mmol), 4 Å MS (50 mg), and $NaHCO_3$ (25.2 mg, 0.3 mmol) in solvent (1 mL, 0.2 M) at room temperature for 24 h; [b] Crude product yield based on ^{1}H NMR analysis with CH_2Br_2 as the internal standard; [c] No reaction based on TLC analysis and ^{1}H NMR measurements of the crude reaction mixture; [d] Reaction performed in the absence of a ligand; [e] Reaction performed in the absence of 4 Å MS; [f] Value in parentheses denote TOF (h^{-1}).

2.7 General Procedure G for the Copper(I)-Catalysed Site-Selective Chlorination of the Tertiary C(sp^3)–H Bond of Ketones 1.

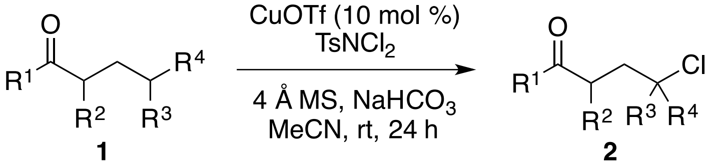

To an oven-dried 10 mL round-bottom flask was added the substrate **1** (0.2 mmol), CuOTf·0.5PhMe (5.2 mg, 0.02 mmol), $NaHCO_3$ (25.2 mg, 0.3 mmol) and 4 Å MS (50 mg). The reaction vessel was capped and charged with a nitrogen atmosphere through three cycles of the vacuum-nitrogen-backfill method over 10 min. An anhydrous solution of acetonitrile (1 mL) containing dichloramine-T (72 mg, 0.3 mmol) was then added and the resulting reaction mixture was stirred at room temperature for 24 h. Upon completion, the reaction mixture was filtered through a Celite pad and washed with CH_2Cl_2 (3 × 1 mL). After evaporation of the organic solvent on a rotary evaporator at 30 °C, the residue obtained was purified by flash column chromatography on silica gel (petroleum ether/Et_2O as eluent) to give the desired chlorination product **2**.

2.8 General Procedure H for the Copper(I)-Catalysed Site-Selective Chlorination of the Secondary C(sp^3)–H Bond of Ketones 1.

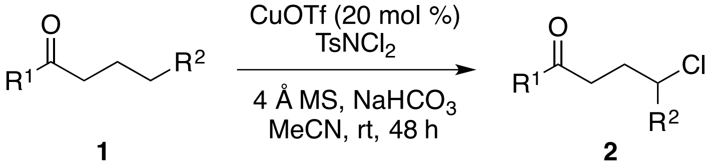

To an oven-dried 10 mL round-bottom flask was added the substrate **1** (0.2 mmol), CuOTf·0.5PhMe (5.2 mg, 0.02 mmol), $NaHCO_3$ (25.2 mg, 0.3 mmol) and 4 Å MS (50 mg). The reaction vessel was capped and charged with a nitrogen atmosphere through three cycles of the vacuum-nitrogen-backfill method over 10 min. An

anhydrous solution of acetonitrile (1 mL) containing dichloramine-T (72 mg, 0.3 mmol) was then added and the resulting reaction mixture was stirred at room temperature for 24 h. A second batch of CuOTf·0.5PhMe (5.2 mg, 0.02 mmol) and $TsNCl_2$ (72 mg, 0.3 mmol) was added at this point and the reaction was stirred for an additional 24 h. Upon completion, the reaction mixture was filtered through a Celite pad and washed with CH_2Cl_2 (3 × 1 mL). After evaporation of the organic solvent on a rotary evaporator at 30 °C, the residue obtained was purified by flash column chromatography on silica gel (petroleum ether/Et_2O ether as eluent) to give the desired chlorination product **2**.

2.9 General Procedure I for Copper(I)-Catalysed Site-Selective Chlorination of the Allylic C(sp^3)–H Bond of (*E*)-Enones 3.

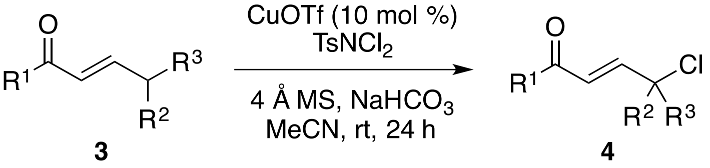

To an oven-dried 10 mL round-bottom flask was added the substrate **3** (0.2 mmol), CuOTf·0.5PhMe (5.2 mg, 0.02 mmol), $NaHCO_3$ (25.2 mg, 0.3 mmol) and 4 Å MS (50 mg). The reaction vessel was capped and charged with a nitrogen atmosphere through three cycles of the vacuum-nitrogen-backfill method over 10 min. An anhydrous solution of acetonitrile (1 mL) containing dichloramine-T (57.6 mg, 0.24 mmol) was added and the resulting reaction mixture was stirred at room temperature for 24 h. Upon completion, the reaction mixture was filtered through a Celite pad and washed with CH_2Cl_2 (3 × 1 mL). After evaporation of the organic solvent on a rotary evaporator at 30 °C, the residue obtained was purified by flash column chromatography on silica gel (petroleum ether/Et_2O as eluent) to give the desired chlorination product **4**.

2.10 General Procedure J for the Copper(I)-Catalysed Site-Selective Chlorination of the Benzylic C(sp^3)–H Bond of Alkylbenzenes 5.

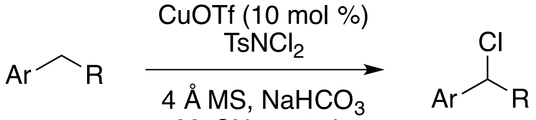

To an oven-dried 10 mL round-bottom flask was added the substrate **5** (0.2 mmol), CuOTf·0.5PhMe (5.2 mg, 0.02 mmol), $NaHCO_3$ (25.2 mg, 0.3 mmol) and 4 Å MS (50 mg). The reaction vessel was capped and charged with a nitrogen atmosphere through three cycles of the vacuum-nitrogen-backfill method over 10 min. An anhydrous solution of acetonitrile (1 mL) containing dichloramine-T (57.6 mg, 0.24 mmol) was then added and the resulting reaction mixture was stirred at room temperature for 24 h. Upon completion, the reaction mixture was filtered through a Celite pad and washed with CH_2Cl_2 (3 × 1 mL). After evaporation of the organic solvent on a rotary evaporator at 30 °C, the residue obtained was purified by flash column chromatography on silica gel (petroleum ether/Et_2O ether as eluent) to give the desired chlorination product **6**.

2.11 General Procedures K for the Synthesis of Deuterated Substrates.

2.11.1 Procedure for the Synthesis of Deuterated Substrate *d*$_2$-1s.[3]

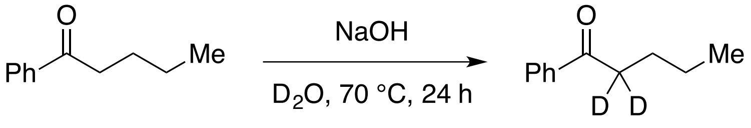

A mixture of 1-phenylpentan-1-one (0.81 g, 5 mmol), NaOH (0.04 g, 1 mmol) and D_2O (8 mL) was stirred at 70 ºC for 24 h under a nitrogen atmosphere. The reaction mixture was extracted with Et_2O (3 × 10 mL). The combined organic phases were dried was dried over Na_2SO_4 and concentrated *in vacuo* to give the crude reaction

mixture. The crude reaction mixture was purified by flash column chromatography on silica gel (*n*hexane/EtOAc as eluent) to affording d_2-**1s** (0.78 g, 95% yield).

2.11.2 Procedure for the Synthesis of Deuterated Substrate d_3-5c.

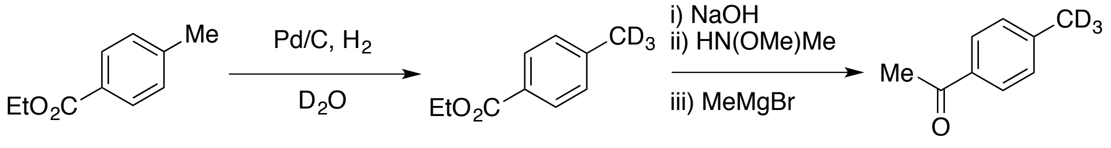

S10 d_3-**5c**

A suspension of the ethyl 4-ethylbenzoate (0.8 g, 5.0 mmol) and Pd/C (80 mg, 10%) in D_2O (5 mL) was stirred at 50 °C in a sealed reaction tube filled with hydrogen gas. After 3 days, the reaction mixture was diluted with Et_2O (10 mL), and filtered through a pad of Celite pad. The filtrate was partitioned between ethereal and aqueous layers. The aqueous layer was extracted with Et_2O (2 × 20 mL). The combined ethereal layers were washed with brine (30 mL), dried over Na_2SO_4, filtered, and concentrated *in vacuo* to give the deuterated compound **S10** (0.8 g, 99% yield).

To a solution of **S10** (0.8g, 5 mmol) in THF (5 ml) was added 10% NaOH solution (1 mL) and the resulting reaction mixture was stirred for 1 h at room temperature. Upon completion, the organic solvent was removed on a rotary evaporator, the remaining aqueous solution was acidified with 1 M of HCl to pH = 6 and extracted with EtOAc (2 × 20 mL). The combined organic layers were washed with brine (10 mL), dried over Na_2SO_4, filtered, and concentrated *in vacuo* to give the crude hydrolysis product 4-(methyl-d_3)benzoic acid.

Without any further purification, to a solution of crude product 4-(methyl-d_3)benzoic acid (0.69g, 5.0 mmol) and *N*,*O*-dimethylhydroxyamine hydrochloride (0.52 g, 5.1 mmol) in CH_2Cl_2 (10 mL) was sequentially added Et_3N (0.6 g, 0.8 mL, 6.0 mmol) and 1-ethyl-3-(3-dimethyllaminopropyl)carbodiimide hydrochloride (0.8 g, 5.1 mmol) at room temperature. The resultant reaction mixture was stirred for 12 h at room

temperature and quenched with water (10 mL). The phases were separated and the aqueous phase extracted with CH_2Cl_2 (2 × 20 mL). The combined organic phases were sequentially washed with HCl (1 M, 2 × 20 mL), saturated solution of $NaHCO_3$ (2 × 20 mL) and brine (20 mL). The organic phase was dried over Na_2SO_4 and concentrated *in vacuo* to give the crude Weinreb amide as a colourless oil (0.9g, >99% yield). The crude Weinreb amide was directly used for the next step without any further purification.

To a solution of the Weinreb amide (0.9g, 5.0 mmol) in THF (10 mL) was slowly added methylmagnesium bromide solution (5 mL, 3 M, 3 equiv.) at 0 °C. The resultant reaction mixture was stirred at room temperature for 2 h and quenched with NH_4Cl solution (5 mL). The phases were separated and the aqueous phase extracted with EtOAc (2 × 5 mL). The combined organic layers were washed with brine (30 mL), dried over Na_2SO_4, filtered, and concentrated *in vacuo* to give the deuterated substrate *d*$_3$-**5c**.

2.12 General Procedures for the Control Experiments of Fig. 3.

2.12.1 Procedure for the Control Experiment of Fig. 3a.

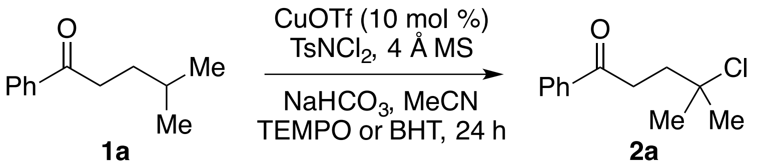

1a **2a**

To an oven-dried 10 mL round-bottom flask was added the substrate **1a** (35.2 mg, 0.2 mmol), CuOTf · 0.5PhMe (5.2 mg, 0.02 mmol), $NaHCO_3$ (25.2mg, 0.3 mmol), TEMPO (234.4 mg, 1.5 mmol) or BHT (330.5 mg, 1.5 mmol) and 4 Å MS (50 mg). The reaction vessel was capped and charged with a nitrogen atmosphere through three cycles of the vacuum-nitrogen-backfill method over 10 min. An anhydrous solution of acetonitrile (1 mL) containing dichloramine-T (72 mg, 0.3 mmol) was added and the

resulting reaction mixture was stirred at room temperature for 24 h. Upon completion, the reaction mixture was filtered through a Celite pad and washed with CH_2Cl_2 (3 × 1 mL). On removing the organic solvent *in vacuo*, the residue was analysed by ^1H NMR measurements. In both control experiments and with dibromomethane (34.8 mg, 0.2 mmol) as the internal standard, this revealed only the ketone was observed in near quantitative yield.

2.12.2 Procedure for the Control Experiment of Fig. 3b.

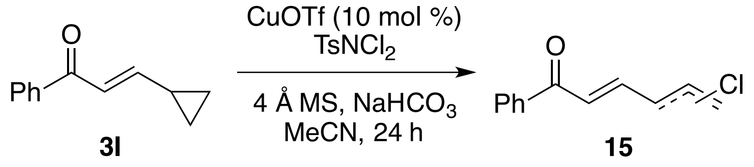

To an oven-dried 10 mL round-bottom flask was added the substrate **3l** (34.4 mg, 0.2 mmol), CuOTf·0.5PhMe (5.2 mg, 0.02 mmol), $NaHCO_3$ (25.2 mg, 0.3 mmol) and 4 Å MS (50 mg). The reaction vessel was capped and charged with a nitrogen atmosphere through three cycles of the vacuum-nitrogen-backfill method over 10 min. An anhydrous solution of acetonitrile (1 mL) containing dichloramine-T (72 mg, 0.3 mmol) was then added and the resulting reaction mixture was stirred at room temperature for 24 h. Upon completion, the reaction mixture was filtered through a Celite pad and washed with CH_2Cl_2 (3 × 1 mL). On removing the organic solvent *in vacuo*, the residue was analysed by ^1H NMR measurements and dibromomethane (34.8 mg, 0.2 mmol) as the internal standard, which revealed the complete consumption of the starting material. However, due to significant overlap of signals, neither identification of the desired product **15** by ^1H NMR analysis nor its isolation by flash column chromatography on silica gel was successful. HRMS analysis of crude reaction mixture indicated the anticipated product could be detected at *m/z* 207.05725.

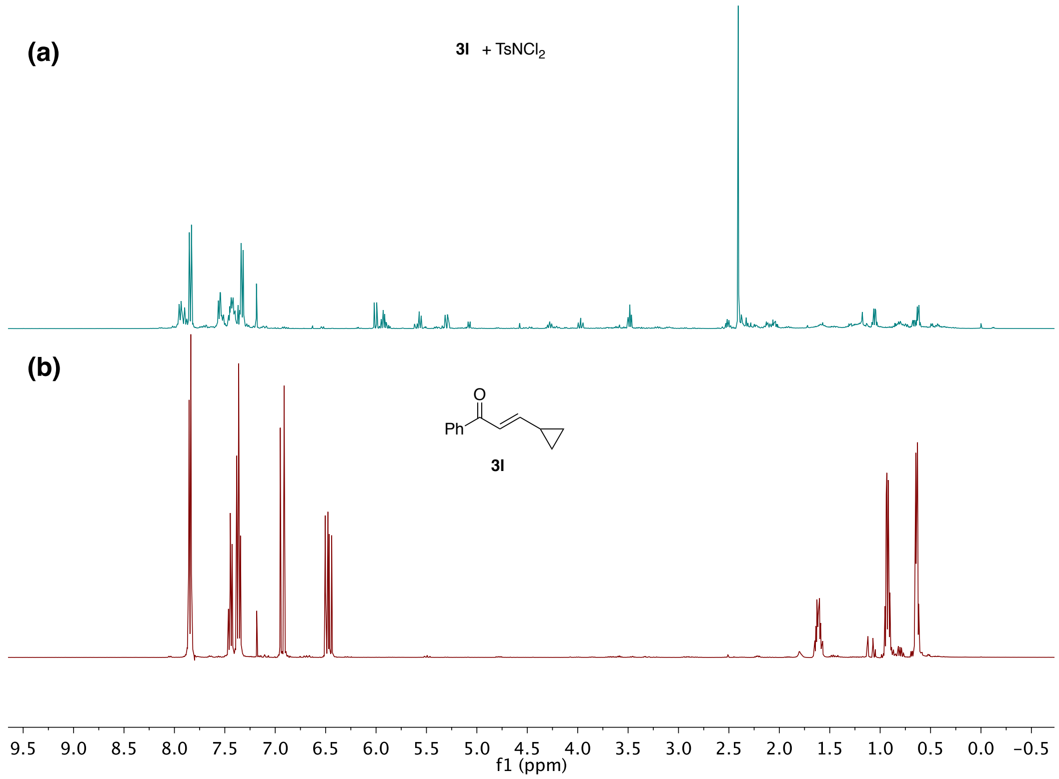

Supplementary Fig. 1 ^{1}H NMR spectra of (a) the crude mixture obtained from the reaction of (**3l** + $TsNCl_2$) and (b) **3l**.

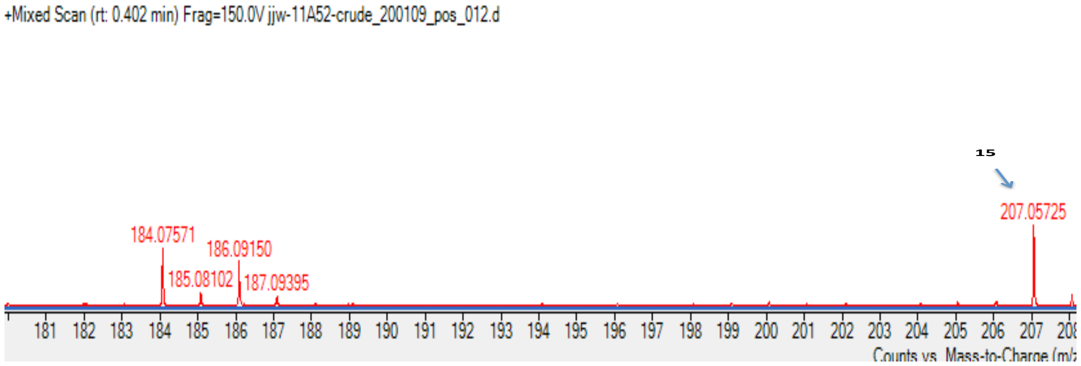

Supplementary Fig. 2 HRMS (ESI) spectrum of the crude mixture obtained from the reaction of (**3l** + $TsNCl_2$).

2.12.3 Procedure for the Control Experiments of Fig. 3c–3e.

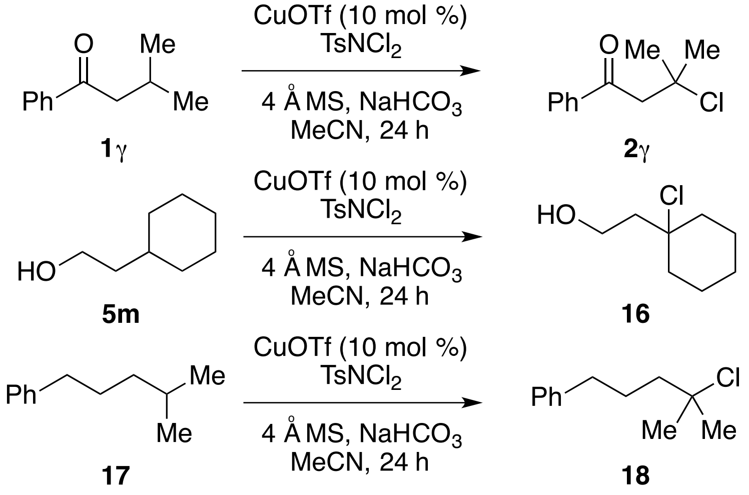

To an oven-dried 10 mL round-bottom flask was added the substrate **1*γ*** (32.4 mg, 0.2 mmol) or **5m** (25.6 mg, 0.2 mmol) or **17** (32.4 mg, 0.2 mmol), CuOTf·0.5PhMe (5.2 mg, 0.02 mmol), $NaHCO_3$ (25.2mg, 0.3 mmol) and 4 Å MS (50 mg). The reaction vessel was capped and charged with a nitrogen atmosphere through three cycles of the vacuum-nitrogen-backfill method over 10 min. An anhydrous solution of acetonitrile (1 mL) containing dichloramine-T (72 mg, 0.3 mmol) was added and the resulting reaction mixture was stirred at room temperature for 24 h. The reaction mixture was then filtered through a Celite pad and washed with CH_2Cl_2 (3 × 1 mL). On removing the organic solvent *in vacuo*, the residue was analysed by ^{1}H NMR measurements. In control experiments with **1*γ*** or **17** as the substrate and with dibromomethane (34.8 mg, 0.2 mmol) as the internal standard, this revealed only the starting material was observed in near quantitative yield. In the control reaction with **5m** as the substrate, purification by flash column chromatography on silica gel (petroleum ether/Et_2O as eluent) furnished the *α*-chlorinated adduct **6m** as the only product in 70% yield.

2.12.4 Procedure for the Control Experiment of Fig. 3f.

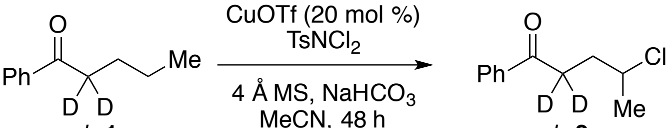

*d*₂-**1s**
97% D-content

*d*₂-**2s**

To an oven-dried 10 mL round-bottom flask was added the substrate d_2-**1s** (32.8 mg, 0.2 mmol), CuOTf·0.5PhMe (10.4 mg, 0.04 mmol), $NaHCO_3$ (25.2 mg, 0.3 mmol) and 4 Å MS (50 mg). The reaction vessel was capped and charged with a nitrogen atmosphere through three cycles of the vacuum-nitrogen-backfill method over 10 min. A solution of acetonitrile (1 mL) containing dichloramine-T (144 mg, 0.6 mmol) was added and the resulting reaction mixture was stirred at room temperature for 48 h. Upon completion, the reaction mixture was filtered through a Celite pad and washed with CH_2Cl_2 (3 × 1 mL). On removing the organic solvent *in vacuo*, the residue was purified by flash column chromatography on silica gel (petroleum ether/Et_2O = 99/1) to afford the product d_2-**2s** in 45% yield. ^{1}H NMR measurements of d_2-**2s** indicated there was no deuterium atom incorporation at γ-position. The deuterium atom content at the α-position of d_2-**2s** was found to remain at 96%.

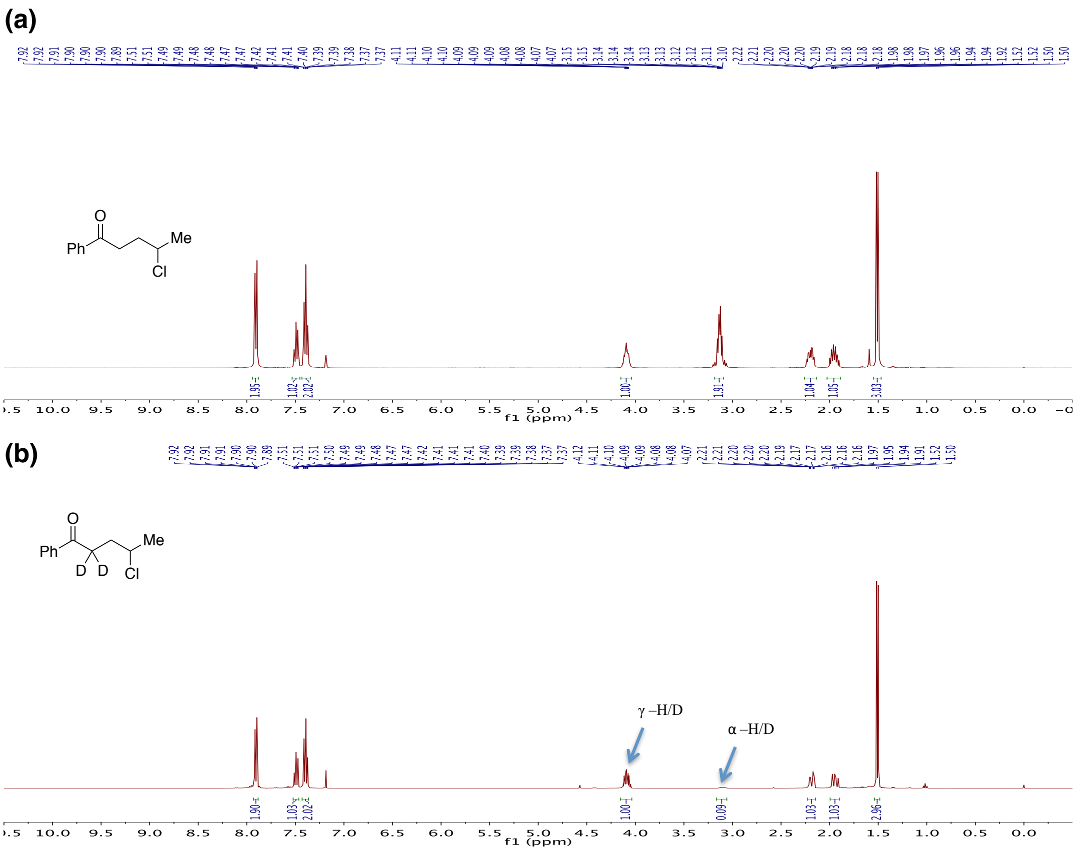

Supplementary Fig. 3 ^{1}H NMR spectrum of (a) **2s** and (b) ***d*$_2$-2s**.

2.12.5 Procedure for the Control Experiments of Fig. 3g.

a) Procedure for the Parallel KIE Control Experiments of Fig. 3g.

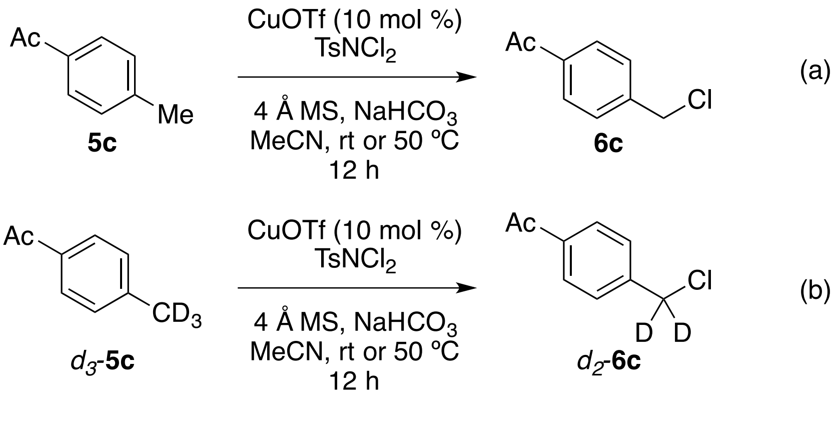

In two separate oven-dried 10 mL round-bottom flasks, one was charged with (a) **5c** (26.8 mg, 0.2 mmol), and the other one was charged with (b) *d*$_3$-**5c** (27.4 mg, 0.2 mmol). To both reaction vessels was added CuOTf·0.5PhMe (5.2 mg, 0.02 mmol), $NaHCO_3$ (25.2 mg, 0.3 mmol) and 4 Å MS (50 mg). The reaction vessels were capped

and charged with a nitrogen atmosphere through three cycles of the vacuum-nitrogen-backfill method over 10 min. A solution of acetonitrile (1 mL) containing dichloramine-T (48 mg, 0.2 mmol) was added to each reaction vessel and the resulting reaction mixtures were stirred at room temperature or 50 °C for 12 h. Upon completion, the two reaction mixtures were separately filtered through a Celite pad and washed with CH_2Cl_2 (3 × 1 mL). On removing the organic solvent *in vacuo*, the two corresponding residues were analysed by ^{1}H NMR measurements. In control experiment (a) at room temperature and with dibromomethane (34.8 mg, 0.2 mmol) as the internal standard, this revealed the conversion of **5c** to **6c** in 35% yield and an isolated yield of 32%. The analogous control reaction at 50 °C gave **6c** with a ^{1}H NMR yield of 37% yield and an isolated yield of 36%. In control experiment (b) at room temperature and with dibromomethane (34.8 mg, 0.2 mmol) as the internal standard, however, no reaction was detected with *d*$_3$-**5c** found to remain in 98% yield. On the other hand, the repeated control reaction at 50 °C afforded *d*$_2$-**6c** with a ^{1}H NMR yield of 6% and an isolated yield of 6%.

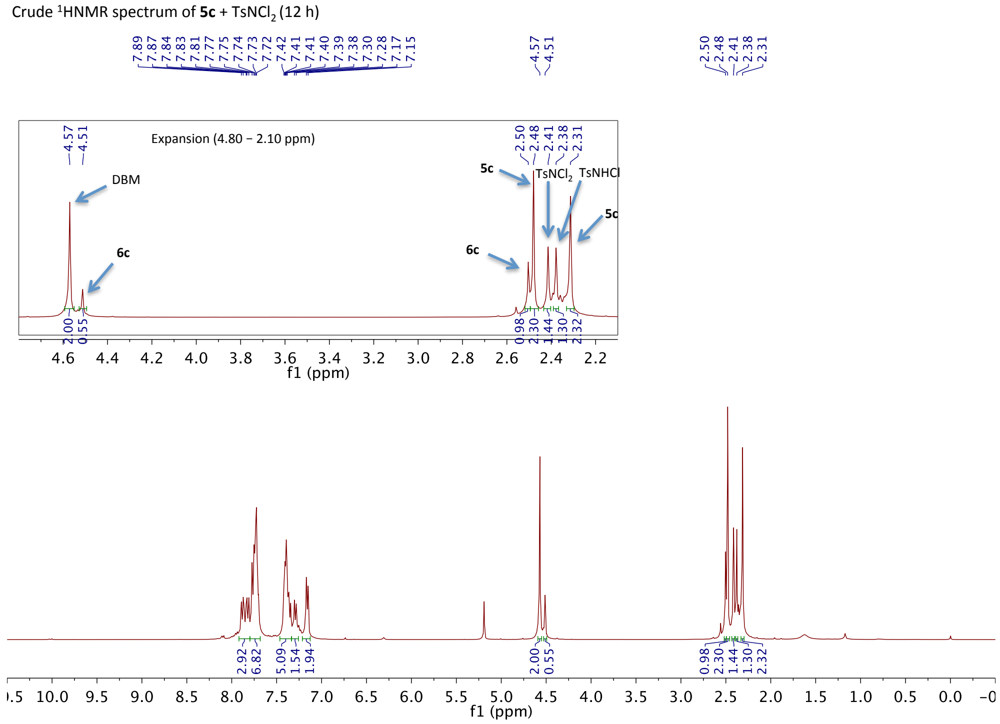

Supplementary Fig. 4 ^{1}H NMR spectrum of the crude mixture obtained from the reaction of (**5c** + $TsNCl_2$) at room temperature after 12 h.

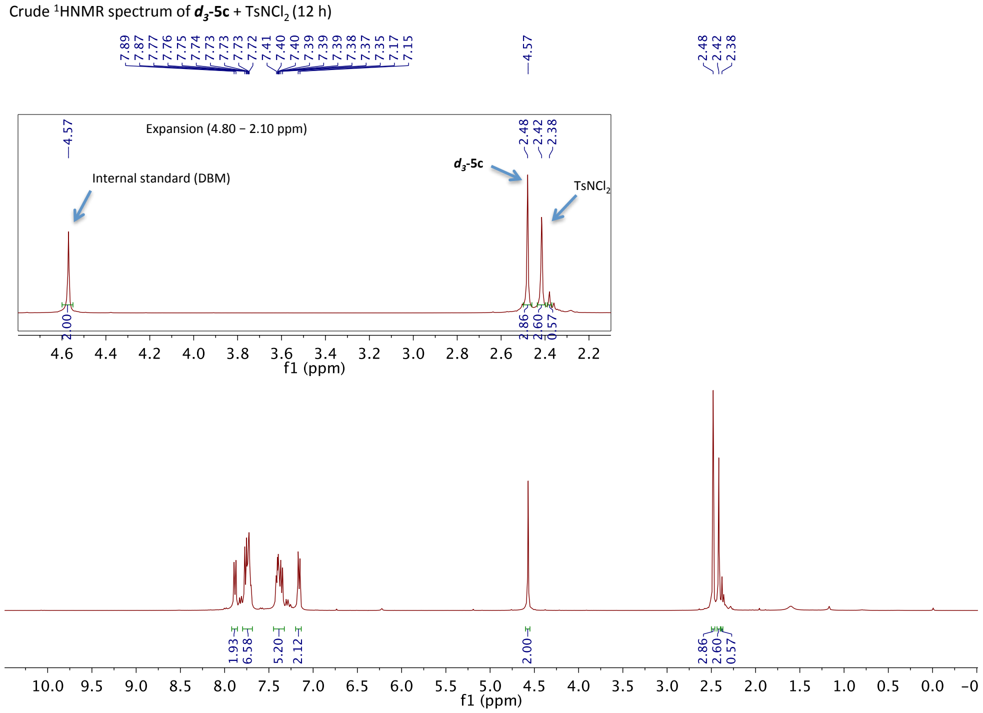

Supplementary Fig. 5 ^{1}H NMR spectrum of the crude mixture obtained from the reaction of (d_3-**5c** + $TsNCl_2$) at room temperature after 12 h.

b) Procedure for the Competitive KIE Control Experiment of Fig. 3g.

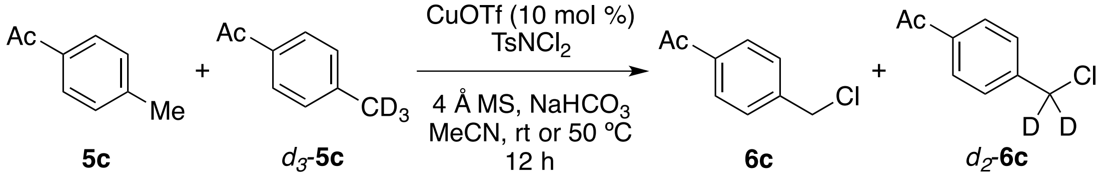

To an oven-dried 10 mL round-bottom flask was added **5c** (26.8 mg, 0.2 mmol), d_3-**5c** (27.4 mg, 0.2 mmol), CuOTf·0.5PhMe (5.2 mg, 0.02 mmol), $NaHCO_3$ (25.2 mg, 0.3 mmol) and 4 Å MS (50 mg). The reaction vessel was capped and charged with a nitrogen atmosphere through three cycles of the vacuum-nitrogen-backfill method over 10 min. A solution of acetonitrile (1 mL) containing dichloramine-T (48 mg, 0.2 mmol) was added and the resulting reaction mixture was stirred at room temperature or 50 °C for 12 h. Upon completion, the reaction mixture was filtered through a Celite pad and washed with CH_2Cl_2 (3 × 1 mL). For the control reaction at room temperature, on removing the organic solvent *in vacuo*, the residue was analysed by ^{1}H NMR measurements. These measurements indicated that **6c** was afforded in 32% yield while d_2-**6c** was not observed. Repeating the competitive KIE control reaction at room temperature once more gave the same result. On the other hand, for the control reaction at 50 °C, after purification by flash column chromatography on silica gel (petroleum ether/Et_2O as eluent), ^{1}H NMR measurements showed that **6c** and d_2-**6c** was obtained in a ratio = 5.8:1.

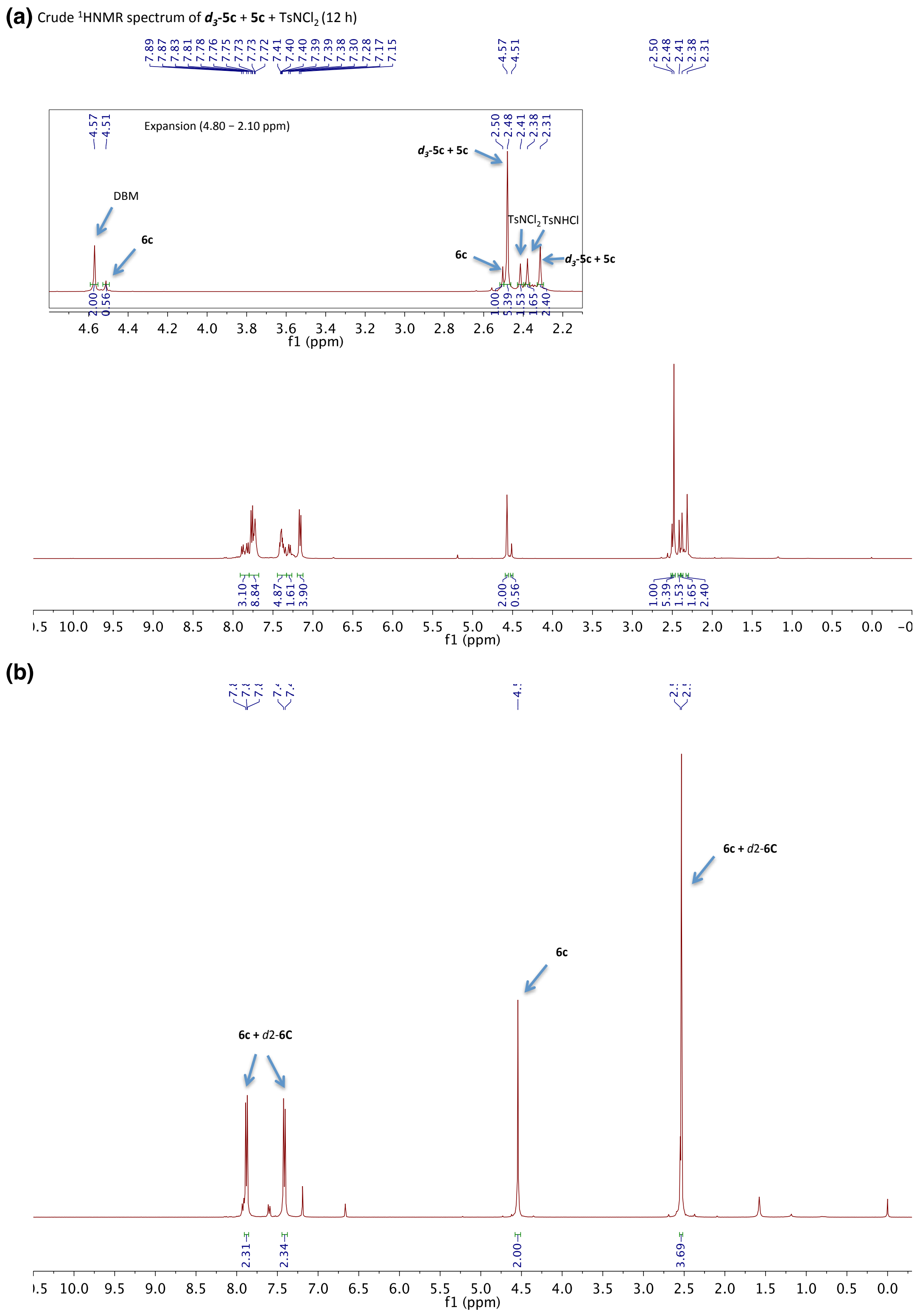

Supplementary Fig. 6 ^{1}H NMR spectrum of the crude mixture obtained from the reaction of (**5c** + d_3-**5c** + $TsNCl_2$) at (a) at room temperature and (b) 50 °C after 12 h.

2.12.6 Procedure for the Control Experiments of Figs. 3h and 3i.

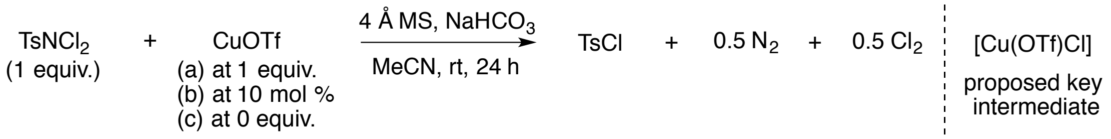

In three separate oven-dried 10 mL round-bottom flasks, one was charged with (a) CuOTf·0.5PhMe (52 mg, 0.2 mmol), the second with (b) CuOTf·0.5PhMe (5.2 mg, 0.02 mmol), and the third with (c) no copper(I) salt. To each of the three reaction vessels was added $NaHCO_3$ (25.2 mg, 0.3 mmol) and 4 Å MS (50 mg). The reaction vessels were capped and charged with a nitrogen atmosphere through three cycles of the vacuum-nitrogen-backfill method over 10 min. A solution of acetonitrile (1 mL) containing dichloramine-T (48 mg, 0.2 mmol) was added to each reaction vessel and the resulting reaction mixtures were stirred at room temperature for 24 h. Upon completion, the three reaction mixtures were separately filtered through a Celite pad and washed with CH_2Cl_2 (3 × 1 mL). On removing the organic solvent of control experiment (a) *in vacuo*, the residue was analysed by HRMS measurements. The residues of control experiments (b) and (c), obtained after the removing the organic solvent *in vacuo*, were analysed by ^1H NMR measurements. In control experiment (a), HRMS analysis detected a molecular ion at *m/z* 247.9136 ($[M + H]^+$) that could be assigned to [Cu(OTf)Cl], which suggested this Cu(II) species could be formed *in situ* and be involved in the reaction pathway. In control experiment (b) and with dibromomethane (34.8 mg, 0.2 mmol) as the internal standard, ^1H NMR analysis revealed the formation of TsCl, which could be isolated in 80% yield. In control experiment (c) and with dibromomethane (34.8 mg, 0.2 mmol) as the internal standard, ^1H NMR analysis detected no reaction with dichloramine-T subsequently isolated in 95% yield. Taken together, control experiments (b) and (c) provided further support for the possible involvement of [Cu(OTf)Cl] in the reaction pathway.

The generation of TsCl could be attributed to the following proposed tentative mechanism shown below.[4]

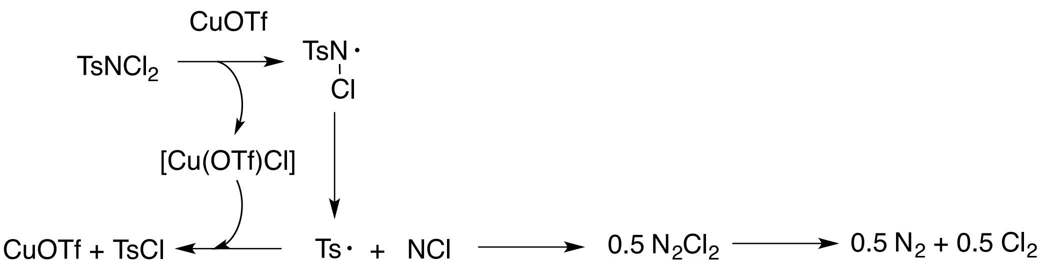

Supplementary Fig. 7 Proposed mechanism for the CuOTf-catalysed decomposition of dichloramine-T.

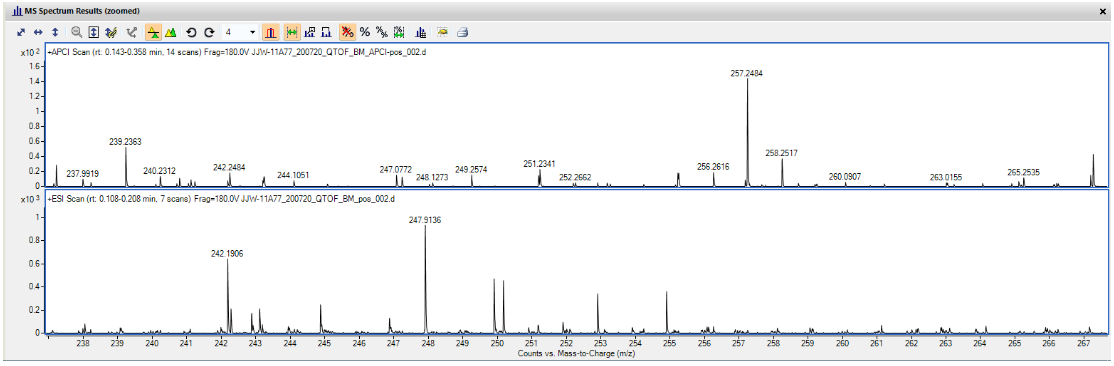

Supplementary Fig. 8 HRMS (ESI) spectrum of the crude mixture obtained from the reaction of a stoichiometric amount of CuOTf and $TsNCl_2$.

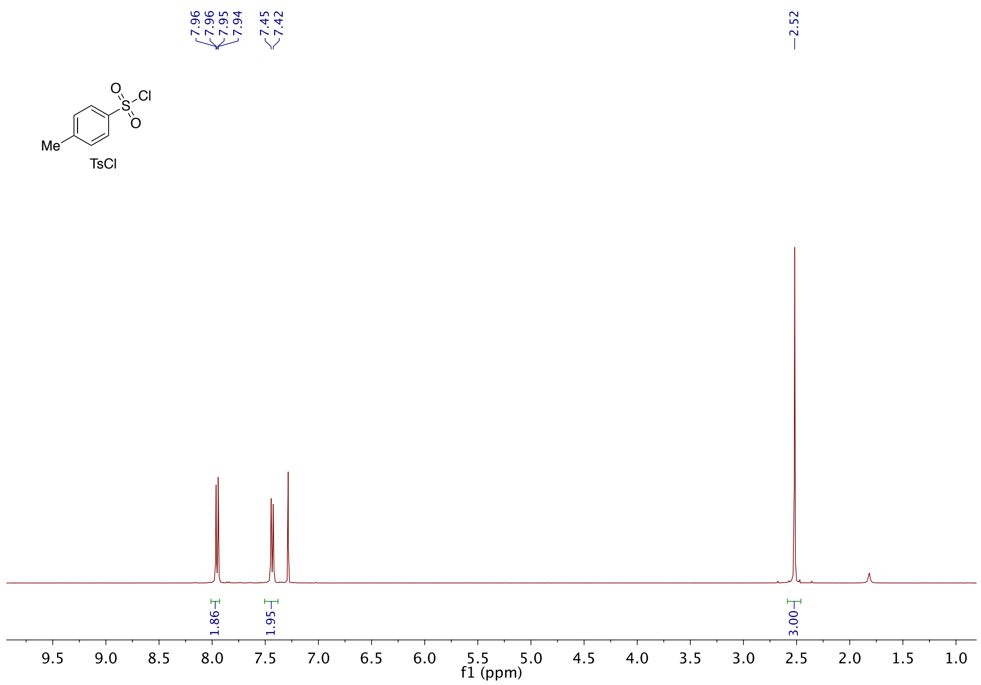

Supplementary Fig. 9 ^{1}H NMR spectrum of *p*-tosyl chloride.

2.13 Gram-Scale Synthesis of Nonpeptide *δ*-Opioid Agonist 7.

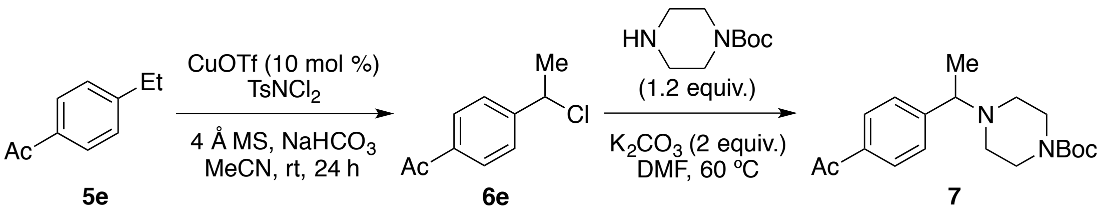

To an oven-dried 100 mL round-bottom flask was added substrate **5e** (1.0 g, 6.8 mmol), CuOTf·0.5PhMe (174.2 mg, 0.68 mmol), $NaHCO_3$ (857 mg, 10.2 mmol) and 4 Å MS (1.5 g). The reaction vessel was capped and charged with a nitrogen atmosphere through three cycles of the vacuum-nitrogen-backfill method over 10 min. A solution of acetonitrile (30 mL) containing dichloramine-T (1.96 g, 8.2 mmol) was added and the resulting reaction mixture was stirred at room temperature for 24 h. Upon completion, the reaction mixture was filtered through a Celite pad and washed with CH_2Cl_2 (3 × 10 mL). On removing the organic solvent under reduced pressure, the residue obtained was purified by flash column chromatography on silica gel

(eluent: petroleum ether/Et_2O = 20/1) to give the desired chlorination product **6e** as a pale-yellow oil (1.1 g, 90% yield).

To an oven-dried 100 mL round-bottom flask was added compound **6e** (1.1 g, 6.0 mmol) and K_2CO_3 (1.6 g, 1.2 mmol). The reaction vessel was capped and charged with a nitrogen atmosphere through three cycles of the vacuum-nitrogen-backfill method over 10 min. A solution of DMF (10 mL) containing *tert*-butyl piperazine-1-carboxylate (1.4 g, 7.2 mmol) was added at room temperature and the resulting reaction mixture was then stirred at 60 °C for 12 h. Upon completion, the reaction was cooled to room temperature and H_2O (50 mL) and EtOAc (20 mL) was added. The two phases were separated and the aqueous phase extracted with EtOAc (2 × 20 mL). The combined organic layers were washed with brine (30 mL), dried over Na_2SO_4, and concentrated *in vacuo*. The residue obtained was purified by flash column chromatography on silica gel (eluent: petroleum ether/EtOAc = 8/1) to give the nonpeptide δ-opioid agonist **7**as a pale-yellow oil (1.9 g, 96% yield).

3. Compound Characterisation Data.

Starting materials **1a** (CAS: 2050-07-9), **1s** (CAS: 1009-14-9), **1u** (CAS: 25017-08-7), **1*β*** (CAS: 106–70–7), **1*γ*** (CAS: 582–62–7), **5a** (CAS: 108-88-3)**, 5b** (CAS: 585-74-0)**, 5c** (CAS: 122-00-9), **5d** (CAS: 134-84-9), **5e** (CAS: 937-30-4), **5g** (CAS: 122-72-5), **5k** (CAS: 83-33-0), **5l** (CAS: 529-34-0), **5m** (CAS: 4442-79-9), **17** (CAS: 4215-86-5) and celestolide (**S14**, CAS: 13171-00-1) were purchased from commercial sources and directly used as received.

1-(4-(*tert*-Butyl)phenyl)-4-methylpentan-1-one (1b)[5]

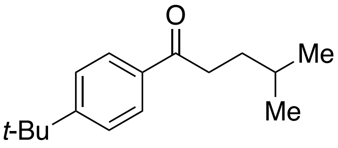

Yield: 78%; colourless liquid; ^{1}H NMR (400 MHz, $CDCl_3$) δ 7.85–7.81 (m, 2H), 7.39 (d, *J* = 8.7 Hz, 2H), 2.90–2.83 (m, 2H), 1.61–1.52 (m, 3H), 1.26 (s, 9H), 0.87 (d, *J* = 6.3 Hz, 6H); ^{13}C NMR (100 MHz, $CDCl_3$) δ 200.4, 156.5, 134.5, 128.0, 125.5, 36.6, 35.1, 33.4, 31.1, 27.9, 22.5.

1-(4-Methoxyphenyl)-4-methylpentan-1-one (1c)[5]

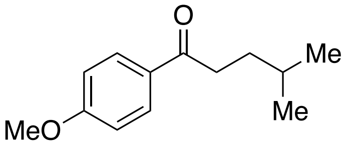

Yield: 90%; colourless liquid; ^{1}H NMR (400 MHz, $CDCl_3$) δ 7.86 (d, *J* = 8.9 Hz, 2H), 6.84 (d, *J* = 8.9 Hz, 2H), 3.77 (s, 3H), 2.87–2.79 (m, 2H), 1.60–1.49 (m, 3H), 0.92–0.83 (m, 6H); ^{13}C NMR (100 MHz, $CDCl_3$) δ 199.4, 163.3, 130.3, 130.2, 113.7, 55.4, 36.3, 33.5, 27.9, 22.5.

4-Methyl-1-(4-(trifluoromethyl)phenyl)pentan-1-one (1d)[6]

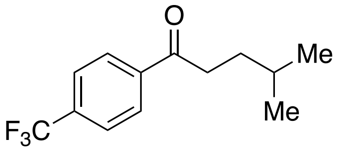

Yield: 68%; colourless liquid; ^{1}H NMR (400 MHz, $CDCl_3$) δ 7.98 (dt, J = 7.8, 1.0 Hz, 2H), 7.65 (d, J = 8.2 Hz, 2H), 2.96–2.87 (m, 2H), 1.57 (ddd, J = 8.9, 5.3, 2.2 Hz, 3H), 0.88 (d, J = 6.3 Hz, 6H); ^{13}C NMR (100 MHz, $CDCl_3$) δ 199.6, 139.7, 134.1 (q, $J_{C\text{-}F}$ = 32.6 Hz), 128.3, 125.6 (q, $J_{C\text{-}F}$ = 3.8 Hz), 36.9, 32.9, 27.7, 22.3.

1-(4-Bromophenyl)-4-methylpentan-1-one (1e)[6]

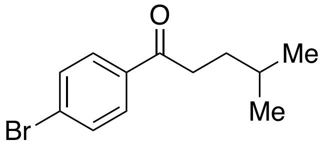

Yield: 92%; white solid; ^{1}H NMR (400 MHz, $CDCl_3$) δ 7.73 (d, J = 8.5 Hz, 2H), 7.55–7.46 (m, 2H), 2.84 (t, J = 7.3 Hz, 2H), 1.60–1.47 (m, 3H), 0.86 (d, J = 5.9 Hz, 6H); ^{13}C NMR (100 MHz, $CDCl_3$) δ 198.3, 139.4, 134.6, 134.2, 128.4, 125.7, 125.7, 125.6, 125.6, 70.1, 39.3, 35.0, 32.6.

1-(3-Methoxyphenyl)-4-methylpentan-1-one (1f)

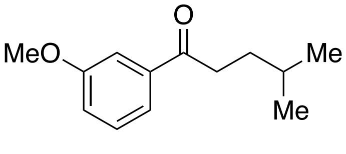

Yield: 95%; colourless oil; ^{1}H NMR (400 MHz, $CDCl_3$) δ 7.46 (dt, J = 7.7, 1.3 Hz, 1H), 7.41 (dd, J = 2.7, 1.6 Hz, 1H), 7.28 (t, J = 7.9 Hz, 1H), 7.01 (ddd, J = 8.2, 2.7, 1.0 Hz, 1H), 3.77 (s, 3H), 2.93–2.80 (m, 2H), 1.56 (ddd, J = 7.6, 4.5, 2.1 Hz, 3H), 0.92–0.82 (m, 6H); ^{13}C NMR (100 MHz, $CDCl_3$) δ 200.5, 159.8, 138.5, 129.5, 120.6, 119.2, 112.4, 55.4, 36.7, 33.3, 27.8, 22.4; HRMS (ESI) calcd. for $C_{13}H_{18}O_2Na$ (M^+ + Na): 229.1199, found: 229.1182; IR (neat, cm^{-1}) 3288, 1594, 1477, 1332, 1157, 1130, 1034, 766, 715.

4-Methyl-1-(*o*-tolyl)pentan-1-one (1g)[5]

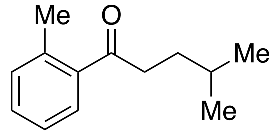

Yield: 92%; colourless liquid; ^{1}H NMR (400 MHz, $CDCl_3$) δ 7.53 (dd, J = 8.1, 1.3 Hz, 1H), 7.27 (td, J = 7.6, 1.4 Hz, 1H), 7.20–7.13 (m, 2H), 2.84–2.77 (m, 2H), 2.40 (s, 3H), 1.59–1.47 (m, 3H), 0.88–0.82 (m, 6H); ^{13}C NMR (100 MHz, $CDCl_3$) δ 205.1, 138.4, 137.7, 131.8, 130.9, 128.1, 125.6, 39.7, 33.2, 27.8, 22.4, 21.1.

1-(3-Fluorophenyl)-4-methylpentan-1-one (1h)[7]

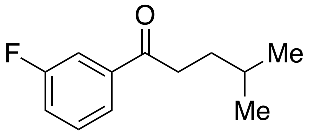

Yield: 92%; colourless liquid; ^{1}H NMR (400 MHz, $CDCl_3$) δ 7.70–7.63 (m, 1H), 7.56 (ddd, J = 9.6, 2.7, 1.6 Hz, 1H), 7.36 (td, J = 8.0, 5.5 Hz, 1H), 7.17 (tdd, J = 8.3, 2.7, 1.0 Hz, 1H), 2.92–2.81 (m, 2H), 1.61–1.49 (m, 3H), 0.92–0.82 (m, 6H); ^{13}C NMR (100 MHz, $CDCl_3$) δ 199.3 (d, $J_{C\text{-}F}$ = 2.0 Hz), 162.8 (d, $J_{C\text{-}F}$ = 247.8 Hz), 139.2 (d, $J_{C\text{-}F}$ = 5.9 Hz), 130.1 (d, $J_{C\text{-}F}$ = 7.7 Hz), 123.7 (d, $J_{C\text{-}F}$ = 2.8 Hz), 119.8 (d, $J_{C\text{-}F}$ = 21.5 Hz), 114.7 (d, $J_{C\text{-}F}$ = 22.1 Hz), 36.76, 33.08, 27.80, 22.39.

1-(3-Methoxy-5-(trifluoromethyl)phenyl)-4-methylpentan-1-one (1i)[8]

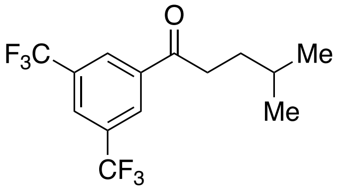

Yield: 65%; colourless liquid; ^{1}H NMR (400 MHz, $CDCl_3$) δ 8.34–8.24 (m, 2H), 8.01–7.94 (m, 1H), 2.99–2.91 (m, 2H), 1.65–1.55 (m, 3H), 0.94–0.86 (m, 6H); ^{13}C NMR (100 MHz, $CDCl_3$) δ 197.5, 138.5, 132.9, 132.3 (q, $J_{C\text{-}F}$ = 33.9 Hz), 128.0 (d, $J_{C\text{-}F}$ = 3.9 Hz), 126.3 – 125.7 (m), 124.3, 121.6, 36.8, 32.7, 27.7, 22.3.

4-Methyl-1-(phenanthren-9-yl)pentan-1-one (1j)

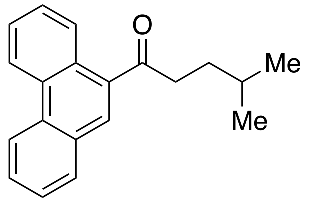

Yield: 88%; white solid; m.p. 90–92 °C; ^{1}H NMR (400 MHz, $CDCl_3$) δ 8.60–8.47 (m, 2H), 8.42–8.34 (m, 1H), 7.93 (d, *J* = 1.7 Hz, 1H), 7.79 (d, *J* = 7.9 Hz, 1H), 7.62–7.44 (m, 4H), 3.03–2.94 (m, 2H), 1.59 (dp, *J* = 13.2, 6.8 Hz, 3H), 0.85 (d, *J* = 6.0 Hz, 6H); ^{13}C NMR (100 MHz, $CDCl_3$) δ 205.3, 135.8, 131.6, 130.8, 130.1, 129.6, 128.6, 128.5, 128.4, 127.4, 127.1, 127.0, 126.5, 122.8, 122.7, 40.5, 33.6, 27.9, 22.5; HRMS (ESI) calcd. for $C_{20}H_{21}O$ (M^+ + H): 277.1592, found: 277.2546; IR (neat, cm^{-1}) 2927, 1288, 1443, 1371, 1273, 1173, 1148, 882, 794, 746, 680.

4-Methyl-1-phenyloctan-1-one (1k)[9]

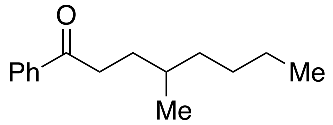

Yield: 95%; colourless oil; ^{1}H NMR (400 MHz, $CDCl_3$) δ 7.93–7.84 (m, 2H), 7.52–7.44 (m, 1H), 7.38 (dd, *J* = 8.3, 6.7 Hz, 2H), 2.88 (ddd, *J* = 9.1, 8.0, 6.0 Hz, 2H), 1.77–1.63 (m, 1H), 1.54–1.36 (m, 2H), 1.34–1.03 (m, 7H), 0.90–0.78 (m, 6H); ^{13}C NMR (100 MHz, $CDCl_3$) δ 200.8, 137.1, 132.8, 128.5, 128.0, 36.5, 36.3, 32.6, 31.4, 29.2, 22.9, 19.5, 14.1.

4-Ethyl-1-phenyloctan-1-one (1l)[10]

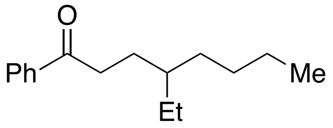

Yield: 90%; colourless oil; ^{1}H NMR (400 MHz, $CDCl_3$) δ 7.91–7.82 (m, 2H), 7.45 (t, *J* = 7.3 Hz, 1H), 7.36 (t, *J* = 7.5 Hz, 2H), 2.91–2.79 (m, 2H), 1.61 (td, *J* = 8.1, 4.5 Hz,

2H), 1.32–1.11 (m, 9H), 0.80 (q, J = 7.2, 6.7 Hz, 6H); ^{13}C NMR (100 MHz, $CDCl_3$) δ 200.8, 137.1, 132.7, 128.5, 128.0, 38.6, 36.0, 32.7, 28.8, 27.7, 25.7, 23.1, 14.1, 10.8.

2,4-Dimethyl-1-phenylpentan-1-one (1m)[6]

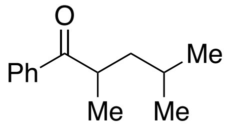

Yield: 76%; colourless liquid; ^{1}H NMR (400 MHz, $CDCl_3$) δ 7.91–7.86 (m, 2H), 7.50–7.44 (m, 1H), 7.42–7.36 (m, 2H), 3.48 (q, J = 6.9 Hz, 1H), 1.70–1.50 (m, 2H), 1.22 (ddd, J = 13.1, 7.2, 6.0 Hz, 1H), 1.10 (d, J = 6.8 Hz, 3H), 0.83 (dd, J = 13.6, 6.5 Hz, 6H); ^{13}C NMR (100 MHz, $CDCl_3$) δ 204.6, 136.8, 132.8, 128.6, 128.2, 42.8, 38.5, 25.9, 23.1, 22.4, 17.5.

3-Cyclopentyl-1-phenylpropan-1-one (1n)[6]

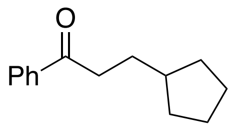

Yield: 70%; colourless liquid; ^{1}H NMR (400 MHz, $CDCl_3$) δ 7.91–7.85 (m, 2H), 7.50–7.44 (m, 1H), 7.41–7.34 (m, 2H), 2.94–2.86 (m, 3H), 1.80–1.62 (m, 6H), 1.62–1.39 (m, 5H), 1.07 (ddt, J = 10.0, 4.9, 1.7 Hz, 2H); ^{13}C NMR (100 MHz, $CDCl_3$) δ 199.6, 136.0, 131.8, 127.5, 127.0, 38.8, 36.9, 31.5, 29.6, 24.1.

3-Cyclohexyl-1-phenylpropan-1-one (1o)[6]

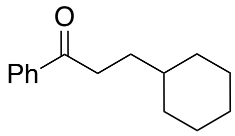

Yield: 72%; colourless oil; ^{1}H NMR (400 MHz, $CDCl_3$) δ 7.92–7.83 (m, 2H), 7.51–7.43 (m, 1H), 7.42–7.32 (m, 2H), 2.94–2.85 (m, 2H), 1.73–1.50 (m, 7H), 1.29–1.02 (m, 4H), 0.97–0.77 (m, 2H); ^{13}C NMR (100 MHz, $CDCl_3$) δ 200.8, 137.1, 132.8, 128.5, 128.1, 37.5, 36.2, 33.2, 31.8, 26.6, 26.3.

6-Methyl-2-phenylheptan-3-one (1p)[11]

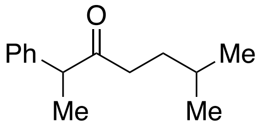

Yield: 88%; colourless liquid; ^{1}H NMR (400 MHz, $CDCl_3$) δ 7.27–7.22 (m, 2H), 7.20–7.16 (m, 1H), 7.16–7.11 (m, 2H), 3.69 (q, J = 7.0 Hz, 1H), 2.27 (ddd, J = 8.0, 6.2, 2.0 Hz, 2H), 1.31 (d, J = 7.0 Hz, 6H), 0.70 (dd, J = 12.6, 6.2 Hz, 6H); ^{13}C NMR (100 MHz, $CDCl_3$) δ 211.1, 140.7, 128.8, 127.8, 127.0, 52.9, 39.0, 32.7, 27.5, 22.3, 22.1, 17.4.

7-Methyl-3-phenyloctan-4-one (1q)

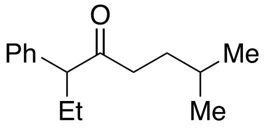

Yield: 68%; colourless oil; ^{1}H NMR (400 MHz, $CDCl_3$) δ 7.23 (dd, J = 8.0, 6.5 Hz, 2H), 7.20–7.15 (m, 1H), 7.15–7.09 (m, 2H), 3.46 (t, J = 7.4 Hz, 1H), 2.32–2.23 (m, 2H), 2.05–1.91 (m, 1H), 1.63 (dt, J = 13.8, 7.5 Hz, 1H), 1.41–1.23 (m, 3H), 0.80–0.65 (m, 10H); ^{13}C NMR (100 MHz, $CDCl_3$) δ 210.9, 139.1, 128.8, 128.3, 127.1, 60.8, 40.0, 32.6, 27.5, 25.3, 22.4, 22.1, 12.1; HRMS (ESI) calcd. for $C_{15}H_{21}O$ (M^+ – H): 217.1592, found: 217.1595; IR (neat, cm^{-1}) 2958, 2923, 2872, 1709, 1687, 1454, 1412, 1366, 1265, 1248, 1170, 1137, 754, 701.

6-Methyl-2-phenoxyheptan-3-one (1r)

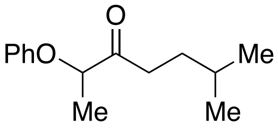

Yield 96%; colourless oil; ^{1}H NMR (400 MHz, $CDCl_3$) δ 7.23–7.15 (m, 2H), 6.88 (t, J = 7.4 Hz, 1H), 6.76 (d, J = 8.1 Hz, 2H), 4.56 (q, J = 6.9 Hz, 1H), 2.57 (ddd, J = 17.9, 9.1, 6.0 Hz, 1H), 2.35 (ddd, J = 17.8, 8.6, 6.0 Hz, 1H), 1.47–1.28 (m, 6H), 0.75

(dd, J = 6.4, 2.6 Hz, 6H); ^{13}C NMR (100 MHz, $CDCl_3$) δ 211.5, 156.5, 128.6, 120.4, 113.9, 78.0, 33.6, 30.7, 26.5, 21.2, 21.2, 16.8; HRMS (ESI) calcd. for $C_{14}H_{19}O_2$ (M^+ – H): 219.1385, found: 219.1332; IR (neat, cm^{-1}) 2957, 2934, 2871, 1719, 1599, 1589, 1229, 1174, 1102, 752, 692.

1-(4-(Trifluoromethyl)phenyl)pentan-1-one (1t)[12]

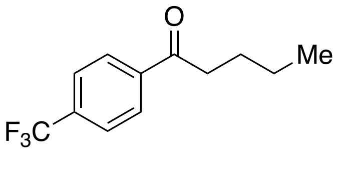

Yield: 80%; colourless oil; ^{1}H NMR (400 MHz, $CDCl_3$) δ 8.03–7.93 (m, 2H), 7.72–7.59 (m, 2H), 2.99–2.86 (m, 2H), 1.73–1.59 (m, 2H), 1.44–1.25 (m, 2H), 0.88 (t, J = 7.3 Hz, 3H); ^{13}C NMR (100 MHz, $CDCl_3$) δ 199.4, 139.8, 134.2 (q, $J_{C\text{-}F}$ = 32.6 Hz), 128.34, 125.62 (d, $J_{C\text{-}F}$ = 3.8 Hz), 38.6, 26.2, 22.4, 13.8.

1-(3-Fluorophenyl)pentan-1-one (1v)[12]

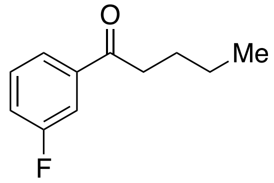

Yield: 98%; colourless liquid; ^{1}H NMR (400 MHz, $CDCl_3$) δ 7.79–7.71 (m, 1H), 7.64 (ddt, J = 9.5, 3.0, 1.5 Hz, 1H), 7.44 (tdd, J = 7.4, 5.5, 1.4 Hz, 1H), 7.25 (tdt, J = 8.3, 2.4, 1.2 Hz, 1H), 2.95 (td, J = 7.4, 1.5 Hz, 2H), 1.80–1.66 (m, 2H), 1.42 (qd, J = 7.4, 1.5 Hz, 2H), 0.96 (td, J = 7.4, 1.5 Hz, 3H); ^{13}C NMR (101 MHz, $CDCl_3$) δ 199.14 (d, $J_{C\text{-}F}$ = 2.0 Hz), 162.86 (d, $J_{C\text{-}F}$ = 247.7 Hz), 139.1 (d, $J_{C\text{-}F}$ = 6.1 Hz), 130.1 (d, $J_{C\text{-}F}$ = 7.6 Hz), 123.7 (d, $J_{C\text{-}F}$ = 3.1 Hz), 119.8 (d, $J_{C\text{-}F}$ = 21.5 Hz), 114.7 (d, $J_{C\text{-}F}$ = 22.2 Hz), 38.4, 26.3, 22.4, 13.9.

1-(3-Chloro-4-methoxyphenyl)pentan-1-one (1w)

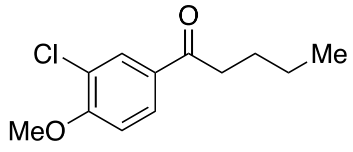

Yield: 65%; colourless oil; ^{1}H NMR (400 MHz, $CDCl_3$) δ 7.91 (d, *J* = 2.2 Hz, 1H), 7.79 (dd, *J* = 8.6, 2.2 Hz, 1H), 6.89 (d, *J* = 8.6 Hz, 1H), 3.89 (s, 3H), 2.86–2.78 (m, 2H), 1.69–1.56 (m, 2H), 1.40–1.28 (m, 2H), 0.88 (t, *J* = 7.3 Hz, 3H); ^{13}C NMR (100 MHz, $CDCl_3$) δ 198.1, 158.6, 130.7, 130.4, 128.4, 122.8, 111.3, 56.3, 38.0, 26.6, 22.5, 13.9; HRMS (ESI) calcd. for $C_{12}H_{16}ClO_2$ (M^+ + H): 227.0838, found: 227.0847; IR (neat, cm^{-1}) 2963, 2872, 1672, 1593, 1571, 1498, 1407, 1312, 1266, 1191, 1057, 822, 696.

2-Phenoxyheptan-3-one (1x)[11]

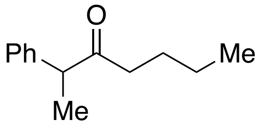

Yield: 96%; colourless oil; ^{1}H NMR (400 MHz, $CDCl_3$) δ 7.28–7.22 (m, 2H), 7.20–7.16 (m, 1H), 7.16–7.10 (m, 2H), 3.67 (q, *J* = 6.9 Hz, 1H), 2.27 (td, *J* = 7.5, 1.3 Hz, 2H), 1.47–1.34 (m, 2H), 1.31 (d, *J* = 7.0 Hz, 3H), 1.18–1.05 (m, 2H), 0.73 (t, *J* = 7.3 Hz, 3H); ^{13}C NMR (100 MHz, $CDCl_3$) δ 210.9, 140.8, 128.8, 127.9, 127.0, 53.0, 40.8, 26.0, 22.2, 17.5, 13.7.

Phenyl 4-Methylpentanoate (1y)[13]

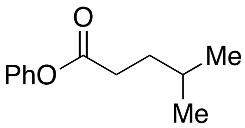

Yield: 68%; colourless oil; ^{1}H NMR (400 MHz, $CDCl_3$) δ 7.32–7.26 (m, 2H), 7.18–7.10 (m, 1H), 7.03–6.97 (m, 2H), 2.52–2.45 (m, 2H), 1.59 (dt, *J* = 7.2, 3.4 Hz, 3H),

0.89 (d, *J* = 6.2 Hz, 6H); ^{13}C NMR (100 MHz, $CDCl_3$) δ 172.4, 150.8, 129.4, 125.7, 121.6, 33.7, 32.5, 27.7, 22.2.

2-(1,3-Dioxoisoindolin-2-yl)ethyl 4-methylpentanoate (1z)

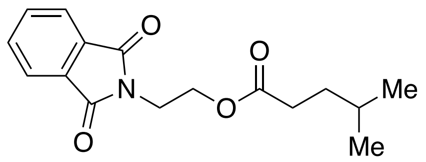

Yield: 67%; colourless liquid; ^{1}H NMR (400 MHz, $CDCl_3$) δ 7.85 (dt, *J* = 7.4, 3.7 Hz, 2H), 7.72 (dq, *J* = 7.2, 4.2 Hz, 2H), 4.32 (t, *J* = 5.3 Hz, 2H), 3.95 (t, *J* = 5.3 Hz, 2H), 2.26 (t, *J* = 7.7 Hz, 2H), 1.57–1.38 (m, 3H), 0.84 (d, *J* = 6.2 Hz, 6H); ^{13}C NMR (100 MHz, $CDCl_3$) δ 173.8, 168.1, 134.0, 132.0, 123.4, 61.3, 37.1, 33.5, 32.1, 27.6, 22.2. HRMS (ESI) calcd. for $C_{16}H_{20}NO_4$ (M^+ + H): 290.1392, found: 290.1394; IR (neat, cm^{-1}) 2956, 2929, 2870, 1776, 1737, 1711, 1390, 1322, 1159, 1106, 998, 719.

***N*,4-Dimethyl-*N*-phenylpentanamide (1*α*)[14]**

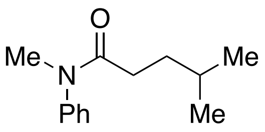

Yield: 60%; yellow oil; ^{1}H NMR (400 MHz, $CDCl_3$) δ 7.34 (dd, *J* = 8.4, 6.8 Hz, 2H), 7.26 (t, *J* = 7.3 Hz, 1H), 7.11 (dd, *J* = 7.4, 1.8 Hz, 2H), 3.18 (s, 3H), 1.99 (t, *J* = 7.7 Hz, 2H), 1.37 (dq, *J* = 17.6, 6.7 Hz, 3H), 0.67 (d, *J* = 6.3 Hz, 6H); ^{13}C NMR (100 MHz, $CDCl_3$) δ 173.5, 144.3, 129.7, 127.7, 127.3, 37.3, 34.5, 32.1, 27.6, 22.2.

(*E*)-4-Methyl-1-phenylpent-2-en-1-one (3a)[15]

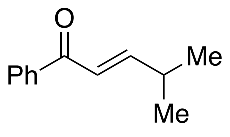

Yield 62%; colourless liquid; ^{1}H NMR (400 MHz, $CDCl_3$) δ 7.88–7.83 (m, 2H), 7.51–7.45 (m, 1H), 7.43–7.37 (m, 2H), 6.96 (dd, *J* = 15.5, 6.7 Hz, 1H), 6.75 (dd, *J* =

15.5, 1.4 Hz, 1H), 2.57–2.47 (m, 1H), 1.07 (d, *J* = 6.8 Hz, 6H); ^{13}C NMR (100 MHz, $CDCl_3$) δ 191.3, 156.1, 138.1, 132.6, 128.5, 128.5, 123.1, 31.5, 21.4.

(*E*)-1-(4-Methoxyphenyl)-4-methylpent-2-en-1-one (3b)

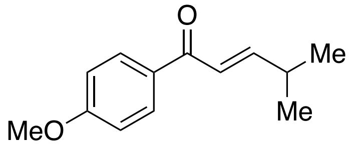

Yield 46%; colourless oil; ^{1}H NMR (400 MHz, $CDCl_3$) δ 7.90–7.83 (m, 2H), 6.93 (ddd, *J* = 15.3, 6.7, 0.9 Hz, 1H), 6.89–6.83 (m, 2H), 6.75 (dt, *J* = 15.4, 1.1 Hz, 1H), 3.83–3.73 (m, 3H), 2.48 (ddt, *J* = 13.5, 6.8, 1.1 Hz, 1H), 1.05 (dt, *J* = 6.9, 0.9 Hz, 6H); ^{13}C NMR (100 MHz, $CDCl_3$) δ 189.5, 163.2, 155.0, 130.9, 130.8, 122.7, 113.7, 55.4, 31.5, 21.5; HRMS (ESI) calcd. for $C_{13}H_{17}O_2$ (M^+ + H): 205.1228, found: 205.1230; IR (neat, cm^{-1}) 2956, 2870, 1670, 1350, 1332, 1034, 1220, 1014, 982, 826, 694.

(*E*)-4-Methyl-1-(*p*-tolyl)pent-2-en-1-one (3c)[15]

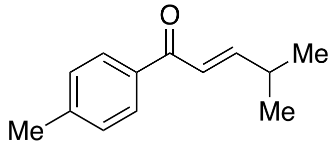

66% yield; colourless liquid; ^{1}H NMR (400 MHz, $CDCl_3$) δ 7.79–7.72 (m, 2H), 7.21–7.12 (m, 2H), 6.93 (ddd, *J* = 15.5, 6.7, 0.9 Hz, 1H), 6.73 (dt, *J* = 15.4, 1.0 Hz, 1H), 2.47 (dddd, *J* = 13.6, 6.9, 2.5, 1.3 Hz, 1H), 2.31 (d, *J* = 2.5 Hz, 3H), 1.08–1.00 (m, 6H); ^{13}C NMR (100 MHz, $CDCl_3$) δ 190.7, 155.4, 143.3, 135.5, 129.2, 128.6, 123.0, 31.5, 21.6, 21.4.

(*E*)-1-([1,1'-biphenyl]-4-yl)-4-methylpent-2-en-1-one (3d)[15]

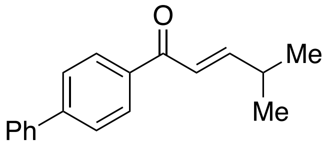

Yield: 66%; colourless oil; ^{1}H NMR (400 MHz, CDCl$_3$) δ 8.08–8.02 (m, 2H), 7.75–7.69 (m, 2H), 7.69–7.64 (m, 2H), 7.50 (tt, *J* = 6.7, 0.9 Hz, 2H), 7.46–7.39 (m, 1H), 7.10 (dd, *J* = 15.5, 6.7 Hz, 1H), 6.90 (dd, *J* = 15.5, 1.3 Hz, 1H), 2.62 (qd, *J* = 6.7, 1.4 Hz, 1H), 1.18 (d, *J* = 6.8 Hz, 6H); ^{13}C NMR (100 MHz, CDCl$_3$) δ 190.7, 155.9, 145.3, 140.0, 136.8, 129.1, 128.9, 128.1, 127.3, 127.2, 123.0, 31.6, 21.4.

(*E*)-4-Methyl-1-(4-(trifluoromethyl)phenyl)pent-2-en-1-one (3e)

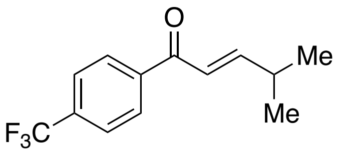

Yield: 75%; colourless oil; ^{1}H NMR (400 MHz, CDCl$_3$) δ 7.96–7.90 (m, 2H), 7.69–7.63 (m, 2H), 6.99 (dd, *J* = 15.5, 6.7 Hz, 1H), 6.71 (dd, *J* = 15.5, 1.4 Hz, 1H), 2.52 (qd, *J* = 6.8, 1.4 Hz, 1H), 1.07 (d, *J* = 6.8 Hz, 6H); ^{13}C NMR (100 MHz, CDCl$_3$) δ 190.4, 157.5, 140.9, 133.8 (d, $J_{C\text{-}F}$ = 32.6 Hz), 128.7, 125.5 (d, $J_{C\text{-}F}$ = 3.7 Hz), 122.9, 31.6, 21.3; HRMS (ESI) calcd. for $C_{13}H_{14}F_3O_3$ (M^+ + H): 243.0996, found: 243.0505; IR (neat, cm^{-1}) 2959, 2871, 1670, 1620, 1322, 1167, 1127, 1066, 827, 757, 686.

(*E*)-1-(2,4-Dimethylphenyl)-4-methylpent-2-en-1-one (3f)

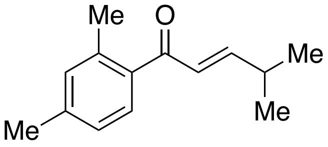

Yield: 80%; colourless oil; ^{1}H NMR (400 MHz, CDCl$_3$) δ 7.25 (d, *J* = 7.7 Hz, 1H), 6.99–6.92 (m, 2H), 6.63 (dd, *J* = 15.8, 6.6 Hz, 1H), 6.37 (dd, *J* = 15.8, 1.4 Hz, 1H), 2.43 (ddd, *J* = 13.5, 6.7, 1.4 Hz, 1H), 2.30 (s, 3H), 2.26 (s, 3H), 1.00 (d, *J* = 6.8 Hz, 7H); ^{13}C NMR (100 MHz, CDCl$_3$) δ 196.7, 156.6, 140.6, 137.2, 136.2, 132.1, 128.6,

127.7, 125.9, 31.3, 21.3, 20.3; HRMS (ESI) calcd. for $C_{14}H_{19}O$ (M^+ + H): 203.1436, found: 203.1435; IR (neat, cm^{-1}) 2964, 2933, 2871, 1670, 1620, 1459, 1304, 1273, 1221, 1181, 1015, 982, 827, 686.

(*E*)-4-Methyl-1-(naphthalen-2-yl)pent-2-en-1-one (3g)

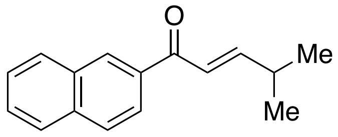

Yield: 48%; white solid; m.p. 106–107 °C; ^{1}H NMR (400 MHz, $CDCl_3$) δ 8.38–8.33 (m, 1H), 7.94 (dd, *J* = 8.6, 1.8 Hz, 1H), 7.89 (ddt, *J* = 7.8, 1.4, 0.7 Hz, 1H), 7.85–7.76 (m, 2H), 7.55–7.43 (m, 2H), 7.03 (dd, *J* = 15.5, 6.6 Hz, 1H), 6.91 (dd, *J* = 15.5, 1.2 Hz, 1H), 2.54 (ddd, *J* = 13.5, 6.8, 1.2 Hz, 1H), 1.09 (d, *J* = 6.7 Hz, 6H); ^{13}C NMR (100 MHz, $CDCl_3$) δ 191.1, 156.0, 135.5, 135.4, 132.5, 129.9, 129.5, 128.4, 128.2, 127.8, 126.7, 124.6, 123.1, 31.6, 21.5. HRMS (ESI) calcd. for $C_{16}H_{17}O$ (M^+ + H): 225.1280, found: 225.1257; IR (neat, cm^{-1}) 2980, 2929, 1668, 1618, 1459, 1352, 1157, 1130, 1034, 808, 766, 715.

(*E*)-4-Methyl-1-(thiophen-2-yl)pent-2-en-1-one (3h)[15]

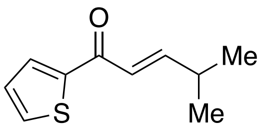

Yield: 42%; colourless liquid; ^{1}H NMR (400 MHz, $CDCl_3$) δ 7.77 (dd, *J* = 3.8, 1.2 Hz, 1H), 7.65 (dd, *J* = 4.9, 1.1 Hz, 1H), 7.15 (dd, *J* = 4.9, 3.8 Hz, 1H), 7.10 (dd, *J* = 15.4, 6.8 Hz, 1H), 6.76 (dd, *J* = 15.4, 1.4 Hz, 1H), 2.58 (qd, *J* = 6.8, 1.4 Hz, 1H), 1.14 (d, *J* = 6.8 Hz, 6H); ^{13}C NMR (100 MHz, $CDCl_3$) δ 182.7, 155.2, 145.3, 133.6, 131.8, 128.1, 122.5, 31.4, 21.4.

(*E*)-4-Methyl-1-phenylhex-2-en-1-one (3i)[17]

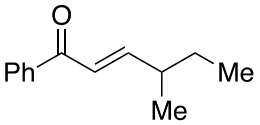

Yield: 80%; colourless liquid; ^{1}H NMR (400 MHz, $CDCl_3$) δ 7.88–7.82 (m, 2H), 7.50–7.43 (m, 1H), 7.42–7.34 (m, 2H), 6.88 (dd, *J* = 15.5, 7.7 Hz, 1H), 6.75 (dd, *J* = 15.4, 1.0 Hz, 1H), 2.25 (p, *J* = 7.0 Hz, 1H), 1.39 (td, *J* = 7.3, 1.9 Hz, 2H), 1.03 (d, *J* = 6.7 Hz, 3H), 0.84 (t, *J* = 7.5 Hz, 3H); ^{13}C NMR (100 MHz, $CDCl_3$) δ 191.1, 155.1, 138.1, 132.5, 128.5, 128.5, 124.3, 38.7, 28.9, 19.1, 11.7.

(*E*)-4-Ethyl-1-phenylhex-2-en-1-one (3j)[15]

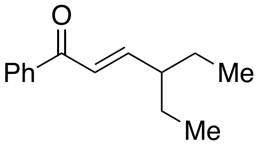

Yield: 82%; colourless liquid; ^{1}H NMR (400 MHz, $CDCl_3$) δ 7.90–7.83 (m, 2H), 7.53–7.45 (m, 1H), 7.45–7.37 (m, 2H), 6.76 (dd, *J* = 4.2, 0.8 Hz, 2H), 2.08–1.98 (m, 1H), 1.55–1.43 (m, 3H), 1.41–1.30 (m, 2H), 0.83 (t, *J* = 7.4 Hz, 6H); ^{13}C NMR (100 MHz, $CDCl_3$) δ 190.9, 154.0, 138.1, 132.5, 128.5, 128.5, 126.0, 46.7, 26.9, 11.8.

(*E*)-3-Cyclohexyl-1-phenylprop-2-en-1-one (3k)[15]

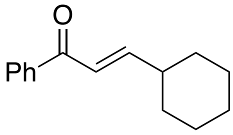

Yield: 70%; colourless oil; ^{1}H NMR (400 MHz, $CDCl_3$) δ 7.87–7.82 (m, 2H), 7.50–7.44 (m, 1H), 7.42–7.35 (m, 2H), 6.93 (dd, *J* = 15.5, 6.8 Hz, 1H), 6.75 (dd, *J* = 15.5, 1.3 Hz, 1H), 2.23–2.11 (m, 1H), 1.83–1.58 (m, 6H), 1.32–1.08 (m, 6H); ^{13}C NMR (100 MHz, $CDCl_3$) δ 191.1, 154.2, 138.1, 132.5, 128.5, 128.5, 124.0, 43.5, 32.6, 25.4.

4-Ethyl-*N*-methoxy-*N*-methylbenzamide (5f)[18]

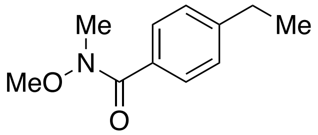

Yield: 95%; ^{1}H NMR (400 MHz, $CDCl_3$) δ 7.66–7.58 (m, 2H), 7.26–7.20 (m, 2H), 3.58 (t, *J* = 1.0 Hz, 3H), 3.36 (t, *J* = 1.0 Hz, 3H), 2.75–2.64 (m, 2H), 1.26 (tt, *J* = 7.7, 0.9 Hz, 3H); ^{13}C NMR (100 MHz, $CDCl_3$) δ 170.0, 147.1, 131.4, 128.4, 127.5, 61.0, 33.9, 28.8, 15.3.

1-(4-Butylphenyl)pentan-1-one (5h)

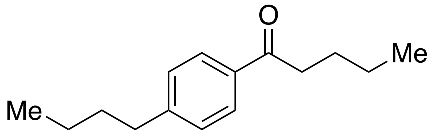

Yield 94%; colourless oil; ^{1}H NMR (400 MHz, $CDCl_3$) δ 7.84–7.77 (m, 2H), 7.21–7.15 (m, 2H), 2.90–2.82 (m, 2H), 2.61–2.54 (m, 2H), 1.70–1.60 (m, 2H), 1.60–1.51 (m, 2H), 1.39–1.29 (m, 2H), 1.25 (dtt, *J* = 9.9, 6.2, 3.4 Hz, 4H), 0.87 (t, *J* = 7.4 Hz, 3H), 0.81 (t, *J* = 6.8 Hz, 3H); ^{13}C NMR (100 MHz, $CDCl_3$) δ 200.2, 148.5, 134.9, 128.5, 128.2, 38.2, 35.9, 31.4, 30.8, 26.6, 22.5, 22.5, 13.9, 13.9; HRMS (ESI) calcd. for $C_{15}H_{23}O$ (M^+ + H): 219.1749, found: 219.1750; IR (neat, cm^{-1}) 2958, 2930, 2340, 1685, 1409, 1372, 1265, 1210, 1034, 976, 766, 715.

Methyl 4-butylbenzoate (5i)[19]

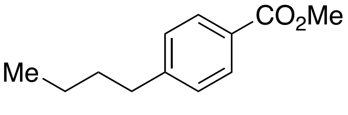

Yield: 90%; colourless oil; ^{1}H NMR (400 MHz, $CDCl_3$) δ 7.97 (dd, *J* = 8.1, 1.3 Hz, 2H), 7.25 (dd, *J* = 8.1, 1.4 Hz, 2H), 3.91 (d, *J* = 1.3 Hz, 3H), 2.67 (t, *J* = 8.0 Hz, 2H), 1.73–1.58 (m, 2H), 1.34 (pd, *J* = 6.7, 6.2, 3.2 Hz, 4H), 0.91 (td, *J* = 7.0, 1.1 Hz, 3H); ^{13}C NMR (100 MHz, $CDCl_3$) δ 167.1, 148.5, 129.6, 128.4, 127.6, 51.9, 36.0, 31.4, 30.8, 22.5, 14.0.

4-Butyl-*N*-methoxy-*N*-methylbenzamide (5j)[20]

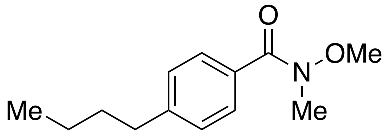

Yield 96%; pale yellow oil; ^{1}H NMR (400 MHz, $CDCl_3$) δ 7.52 (dd, *J* = 8.2, 1.4 Hz, 2H), 7.16–7.08 (m, 2H), 3.48 (d, *J* = 1.3 Hz, 3H), 3.26 (d, *J* = 1.3 Hz, 3H), 2.55 (t, *J* = 7.8 Hz, 2H), 1.60–1.49 (m, 2H), 1.25 (qd, *J* = 4.6, 2.3 Hz, 4H), 0.87–0.73 (m, 3H); ^{13}C NMR (100 MHz, $CDCl_3$) δ 170.0, 145.8, 131.3, 128.3, 128.0, 60.9, 35.8, 33.9, 31.4, 30.8, 22.5, 14.0.

1-(2-Chloropyridin-4-yl)-4-methylpentan-1-one (S8)[18]

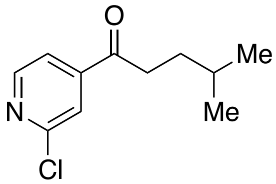

Yield 78%; yellow oil; ^{1}H NMR (400 MHz, $CDCl_3$) δ 8.48 (s, 1H), 7.68 (s, 1H), 7.58 (dd, *J* = 5.1, 1.4 Hz, 1H), 2.87 (dd, *J* = 8.0, 6.4 Hz, 2H), 1.56 (ddd, *J* = 9.4, 4.8, 2.4 Hz, 3H), 0.87 (d, *J* = 6.0 Hz, 6H); ^{13}C NMR (100 MHz, $CDCl_3$) δ 198.4, 152.8, 150.8, 145.8, 122.3, 119.9, 37.1, 32.5, 27.6, 22.3.

Methyl 2-(1,3-dioxoisoindolin-2-yl)-4-methylpentanoate (S9)[18]

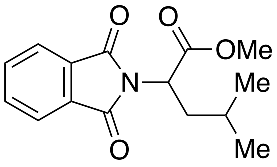

Yield 74%; white solid; ^{1}H NMR (400 MHz, $CDCl_3$) δ 7.88 (ddd, *J* = 5.4, 3.1, 0.9 Hz, 2H), 7.76 (ddd, *J* = 5.6, 3.1, 0.9 Hz, 2H), 4.97 (dd, *J* = 11.6, 4.3 Hz, 1H), 3.74 (d, *J* = 0.9 Hz, 3H), 2.35 (ddd, *J* = 15.0, 11.6, 4.0 Hz, 1H), 2.04–1.94 (m, 1H), 1.50 (ddt, *J* = 13.8, 6.8, 4.0 Hz, 1H), 1.03–0.91 (m, 6H); ^{13}C NMR (100 MHz, $CDCl_3$) δ 170.3, 167.7, 134.2, 131.8, 123.5, 52.7, 50.6, 37.3, 25.1, 23.2, 21.0.

Methyl 2-(4-Isobutylphenyl)propanoate (S10)[21]

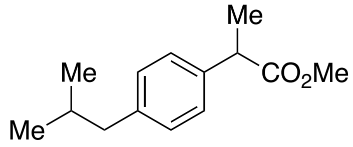

Yield: 96%; white solid; ^{1}H NMR (400 MHz, $CDCl_3$) δ 7.12 (d, J = 7.8 Hz, 2H), 7.02 (d, J = 7.9 Hz, 2H), 3.62 (d, J = 7.2 Hz, 1H), 3.58 (d, J = 1.1 Hz, 3H), 2.37 (d, J = 7.1 Hz, 2H), 1.77 (dt, J = 13.5, 6.7 Hz, 1H), 1.41 (dd, J = 7.2, 1.1 Hz, 3H), 0.82 (dd, J = 6.6, 1.0 Hz, 6H); ^{13}C NMR (100 MHz, $CDCl_3$) δ 175.2, 140.5, 137.8, 129.4, 127.2, 127.1, 52.0, 45.1, 45.0, 30.2, 22.4, 18.6.

Methyl 4'-hexyl-[1,1'-biphenyl]-4-carboxylate (S11)[22]

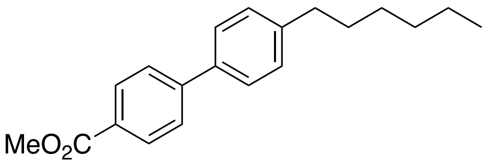

Yield 95%; white solid; ^{1}H NMR (400 MHz, $CDCl_3$) δ 8.04–7.99 (m, 2H), 7.61–7.54 (m, 2H), 7.49–7.43 (m, 2H), 7.23–7.16 (m, 2H), 3.86 (s, 3H), 2.58 (t, J = 7.8 Hz, 2H), 1.64–1.49 (m, 2H), 1.37–1.14 (m, 6H), 0.92–0.76 (m, 3H); ^{13}C NMR (100 MHz, $CDCl_3$) δ 130.1, 129.0, 127.1, 126.8, 52.1, 35.7, 31.7, 31.4, 29.0, 22.6, 14.1.

4-Ethylbenzyl 7,7-Dimethyl-3-oxo-2-oxabicyclo[2.2.1]heptane-6-carboxylate (S12)[21]

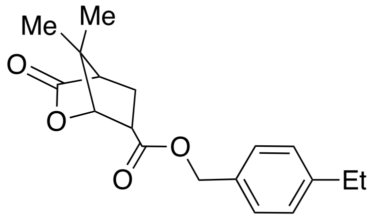

Yield: 60%; white solid; ^{1}H NMR (400 MHz, $CDCl_3$) δ 7.18–7.10 (m, 2H), 6.95 (dd, J = 8.4, 1.5 Hz, 2H), 2.58 (q, J = 7.6 Hz, 2H), 2.49 (dddd, J = 14.9, 10.7, 4.3, 1.4 Hz, 1H), 2.11 (dddd, J = 13.6, 9.4, 4.6, 1.3 Hz, 1H), 1.92 (tdd, J = 12.2, 4.5, 1.4 Hz, 1H),

1.73–1.64 (m, 1H), 1.16 (td, J = 7.6, 1.4 Hz, 3H), 1.08 (dd, J = 7.1, 1.3 Hz, 6H), 1.02 (d, J = 1.3 Hz, 3H); ^{13}C NMR (100 MHz, $CDCl_3$) δ 177.9, 166.3, 147.9, 142.4, 128.9, 121.0, 90.9, 54.9, 54.7, 30.8, 29.0, 28.3, 16.9, 15.6, 9.8.

4-Ethylphenyl 2-(4-(4-Chlorobenzoyl)phenoxy)-2-methylpropanoate (S13)[21]

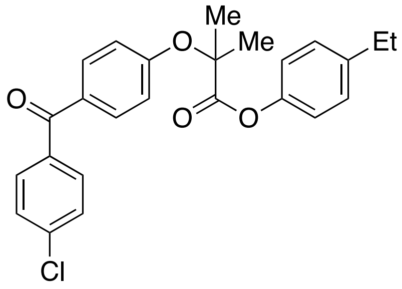

Yield: 70%; white solid; ^{1}H NMR (400 MHz, $CDCl_3$) δ 7.74–7.68 (m, 1H), 7.68–7.62 (m, 1H), 7.41–7.35 (m, 1H), 7.14–7.07 (m, 1H), 6.95–6.89 (m, 1H), 6.85–6.79 (m, 1H), 2.56 (q, J = 7.6 Hz, 1H), 1.75 (s, 3H), 1.14 (dd, J = 8.3, 6.9 Hz, 2H); ^{13}C NMR (100 MHz, $CDCl_3$) δ 194.2, 172.6, 159.6, 148.3, 142.3, 138.5, 136.3, 132.1, 131.2, 130.6, 128.9, 128.6, 120.8, 117.4, 79.5, 28.3, 25.5, 15.6.

(*E*)-3-Cyclopropyl-1-phenylprop-2-en-1-one (3l)[23]

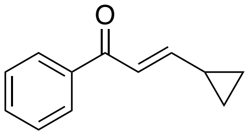

Yield: 78%; colourless oil; ^{1}H NMR (400 MHz, $CDCl_3$) δ 7.88–7.81 (m, 2H), 7.48–7.41 (m, 1H), 7.41–7.33 (m, 2H), 6.93 (d, J = 15.1 Hz, 1H), 6.47 (dd, J = 15.1, 10.2 Hz, 1H), 1.68–1.56 (m, 1H), 0.97–0.90 (m, 2H), 0.64 (dd, J = 4.5, 2.4 Hz, 2H); ^{13}C NMR (100 MHz, $CDCl_3$) δ 189.9, 155.2, 138.2, 132.4, 128.5, 128.4, 122.9, 15.4, 9.3.

1-Phenylpentan-1-one-2,2-d_2 (d_2-1s)[24]

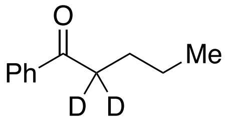

Yield: 98%; colourless liquid; ^{1}H NMR (600 MHz, $CDCl_3$) δ 7.98 (dt, J = 8.3, 1.2 Hz, 2H), 7.56 (dd, J = 7.2, 1.5 Hz, 1H), 7.48 (td, J = 7.8, 1.6 Hz, 2H), 1.76–1.71 (m, 2H), 1.43 (qd, J = 7.4, 1.1 Hz, 2H), 0.98 (td, J = 7.4, 0.9 Hz, 3H); ^{13}C NMR (150 MHz, $CDCl_3$) δ 200.7, 137.1, 132.9, 128.5, 128.1, 26.4, 22.5, 14.0.

1-(4-(Methyl-d3)phenyl)ethan-1-one (d_3-5c)[3]

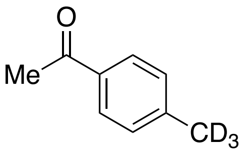

Yield: 78%; colourless liquid; ^{1}H NMR (400 MHz, $CDCl_3$) δ 7.85–7.73 (m, 2H), 7.18 (d, J = 8.0 Hz, 2H), 2.50 (s, 3H); ^{13}C NMR (100 MHz, $CDCl_3$) δ 197.9, 143.8, 134.7, 129.2, 128.4, 26.5.

4-Chloro-4-methyl-1-phenylpentan-1-one (2a)

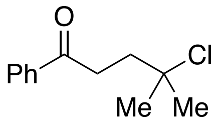

Colourless oil; TOF = 0.34 h^{-1};^{1}H NMR (400 MHz, $CDCl_3$) δ 7.94–7.89 (m, 2H), 7.51–7.45 (m, 1H), 7.42–7.35 (m, 2H), 3.18–3.12 (m, 2H), 2.15–2.08 (m, 2H), 1.55 (d, J = 0.7 Hz, 6H); ^{13}C NMR (100 MHz, $CDCl_3$) δ 199.4, 136.8, 133.1, 128.6, 128.1, 70.4, 39.6, 34.7, 32.6; HRMS (ESI) calcd. for $C_{12}H_{14}ClO$ (M^+ – H): 209.0733, found: 209.0747; IR (neat, cm^{-1}) 2970, 2920, 1684, 1580, 1394, 1370, 1310, 1210, 1108, 1066, 996, 830, 782, 734.

1-(4-(*tert*-Butyl)phenyl)-4-chloro-4-methylpentan-1-one (2b)

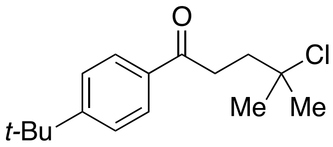

Colourless oil; TOF = 0.24 h^{-1}; ^{1}H NMR (400 MHz, $CDCl_3$) δ 7.92–7.82 (m, 2H), 7.46–7.33 (m, 2H), 3.21–3.06 (m, 2H), 2.18–2.05 (m, 2H), 1.55 (s, 6H), 1.27 (s, 9H); ^{13}C NMR (100 MHz, $CDCl_3$) δ 199.1, 156.8, 134.2, 128.1, 125.6, 70.5, 39.7, 35.1, 34.7, 32.6, 31.1; HRMS (ESI) calcd. for $C_{16}H_{24}ClO$ (M^+ + H): 267.1515, found: 267.1522; IR (neat, cm^{-1}) 2962, 2906, 2869, 1689, 1606, 1465, 1404, 1371, 1317, 1303, 1220, 1186, 1106, 1004, 981, 832, 786, 734, 703.

4-Chloro-1-(4-methoxyphenyl)-4-methylpentan-1-one (2c)

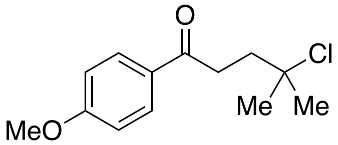

Colourless oil; TOF = 0.24 h h^{-1}; ^{1}H NMR (400 MHz, $CDCl_3$) δ 7.91 (d, J = 8.9 Hz, 2H), 6.87 (d, J = 8.9 Hz, 2H), 3.80 (s, 3H), 3.16–3.03 (m, 2H), 2.16–2.04 (m, 2H), 1.56 (s, 6H); ^{13}C NMR (100 MHz, $CDCl_3$) δ 198.0, 163.5, 130.4, 129.9, 113.8, 70.6, 55.5, 55.5, 39.8, 34.4, 32.6; HRMS (ESI) calcd. for $C_{13}H_{18}ClO_2$ (M^+ + H): 241.0995, found: 241.0870; IR (neat, cm^{-1}) 2970, 2931, 1667, 1600, 1575, 1508, 1457, 1420, 1369, 1296, 1255, 1216, 1171, 1108, 1028, 978, 836, 808, 781.

4-Chloro-4-methyl-1-(4-(trifluoromethyl)phenyl)pentan-1-one (2d)

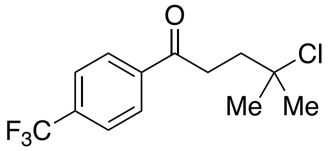

Colourless oil; TOF = 0.35 h^{-1}; ^{1}H NMR (400 MHz, $CDCl_3$) δ 8.03 (d, J = 8.1 Hz, 2H), 7.67 (d, J = 8.2 Hz, 2H), 3.24–3.14 (m, 2H), 2.18–2.06 (m, 2H), 1.57 (s, 6H); ^{13}C NMR (100 MHz, $CDCl_3$) δ 198.4, 139.4, 134.4 (d, $J_{C\text{-}F}$ = 32.6 Hz), 128.4 , 125.7

(q, $J_{C\text{-}F}$ = 3.7 Hz), 124.9, 122.2, 70.1, 39.4, 35.1, 32.6; HRMS (ESI) calcd. for $C_{13}H_{14}F_3O$ (M^+ – Cl): 243.0996, found: 243.0992; IR (neat, cm^{-1}) 2964, 1684, 1605, 1593, 1371, 1322, 1294, 1188, 1173, 1107, 1080, 1068, 843, 810, 700.

1-(4-Bromophenyl)-4-chloro-4-methylpentan-1-one (2e)

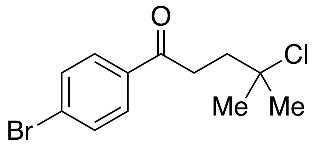

Colourless oil; TOF = 0.34 h^{-1}; ^1H NMR (400 MHz, $CDCl_3$) δ 7.83–7.75 (m, 2H), 7.59–7.47 (m, 2H), 3.19–3.05 (m, 2H), 2.18–2.05 (m, 2H), 1.56 (s, 6H); ^{13}C NMR (100 MHz, $CDCl_3$) δ 198.3, 135.5, 132.0, 129.6, 128.3, 70.3, 39.5, 34.7, 32.6; HRMS (ESI) calcd. for $C_{12}H_{14}BrO$ (M^+ – Cl): 253.0228, 255.0207, found: 253.0222, 255.0201; IR (neat, cm^{-1}) 2971, 2922, 1685, 1584, 1396, 1371, 1312, 1293, 1212, 1106, 1069, 998, 978, 830, 782, 769.

4-Chloro-1-(3-methoxyphenyl)-4-methylpentan-1-one (2f)

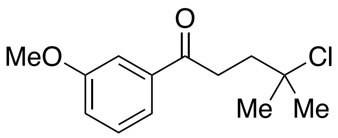

Colourless oil; TOF = 0.35 h^{-1}; ^1H NMR (400 MHz, $CDCl_3$) δ 7.60 (dd, J = 7.7, 1.8 Hz, 1H), 7.38 (ddd, J = 8.3, 7.3, 1.8 Hz, 1H), 6.96–6.87 (m, 2H), 3.85 (s, 2H), 3.18–3.12 (m, 2H), 2.12–2.06 (m, 2H), 1.54 (s, 6H); ^{13}C NMR (100 MHz, $CDCl_3$) δ 201.9, 158.5, 133.4, 130.3, 128.4, 120.7, 111.6, 70.5, 55.5, 39.9, 32.6; HRMS (ESI) calcd. for $C_{13}H_{18}ClO_2$ (M^+ + H): 241.0995, found: 241.0982; IR (neat, cm^{-1}) 2970, 2928, 1674, 1596, 1484, 1464, 1437, 1370, 1290, 1243, 1179, 1162, 1111, 1023, 974, 754, 708.

4-Chloro-4-methyl-1-(*o*-tolyl)pentan-1-one (2g)

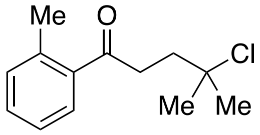

Colourless oil; TOF = 0.22 h^{-1}; ^{1}H NMR (400 MHz, $CDCl_3$) δ 7.62 (dd, *J* = 7.7, 1.4 Hz, 1H), 7.30 (td, *J* = 7.5, 1.4 Hz, 1H), 7.23–7.14 (m, 2H), 3.11–3.03 (m, 2H), 2.42 (s, 3H), 2.14–2.06 (m, 2H), 1.55 (s, 6H); ^{13}C NMR (100 MHz, CDCl3) δ 203.3, 138.0, 137.8, 131.9, 131.3, 128.4, 125.7, 70.2, 70.2, 39.6, 37.6, 32.6, 21.2; HRMS (ESI) calcd. for $C_{13}H_{18}ClO$ (M^+ + H): 225.1046, found: 225.0973; IR (neat, cm^{-1}) 2972, 2925, 1677, 1599, 1571, 1448, 1387, 1369, 1295, 1212, 1161, 1110, 973, 749, 725.

4-Chloro-1-(3-fluorophenyl)-4-methylpentan-1-one (2h)

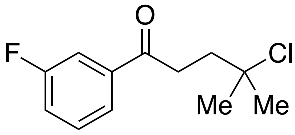

Colourless oil; TOF = 0.24 h^{-1}; ^{1}H NMR (400 MHz, $CDCl_3$) δ 7.71 (ddd, *J* = 7.8, 1.7, 0.9 Hz, 1H), 7.59 (ddd, *J* = 9.5, 2.7, 1.6 Hz, 1H), 7.38 (td, *J* = 8.0, 5.5 Hz, 1H), 7.23–7.16 (m, 1H), 3.17–3.11 (m, 2H), 2.15–2.08 (m, 2H), 1.56 (s, 6H); ^{13}C NMR (100 MHz, $CDCl_3$) δ 198.0 (d, $J_{C\text{-}F}$ = 2.1 Hz), 162.9 (d, $J_{C\text{-}F}$ = 248.0 Hz), 138.9 (d, $J_{C\text{-}F}$ = 6.0 Hz), 130.3 (d, $J_{C\text{-}F}$ = 8.2 Hz), 123.8 (d, $J_{C\text{-}F}$ = 2.8 Hz), 120.1 (d, $J_{C\text{-}F}$ = 23.9 Hz), 114.8 (d, $J_{C\text{-}F}$ = 22.3 Hz), 70.1, 39.4, 34.9, 32.6; HRMS (ESI) calcd. for $C_{12}H_{14}FO$ (M^+ – Cl): 193.1028, found: 193.1023; IR (neat, cm^{-1}) 2972, 2924, 1686, 1588, 1566, 1472, 1443, 1425, 1371, 1299, 1244, 1174, 1110, 727, 681.

1-(3,5-bis(Trifluoromethyl)phenyl)-4-chloro-4-methylpentan-1-one (2i)

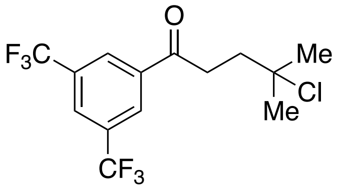

Colourless oil; TOF = 0.21 h^{-1}; ^{1}H NMR (400 MHz, $CDCl_3$) δ 8.35 (d, J = 1.7 Hz, 2H), 8.01 (s, 1H), 3.26–3.18 (m, 2H), 2.19–2.12 (m, 2H), 1.59 (s, 6H); ^{13}C NMR (100 MHz, $CDCl_3$) δ 196.4, 138.2, 132.4 (q, $J_{C\text{-}F}$ = 34.0 Hz), 130.2, 128.0 (d, $J_{C\text{-}F}$ = 3.9 Hz), 127.0, 126.4 – 126.2 (m), 69.8, 39.1, 35.0, 32.6; HRMS (ESI) calcd. for $C_{14}H_{13}F_6O$ (M^+ – Cl): 311.0870, found: 311.0843; IR (neat, cm^{-1}) 3305, 3109, 2208, 1594, 1477, 1383, 1324, 1221, 1159, 1108, 1020, 809, 782, 716.

4-Chloro-4-methyl-1-(phenanthren-9-yl)pentan-1-one (2j)

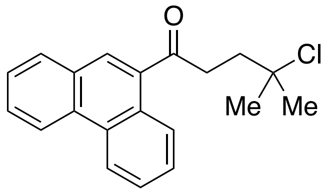

White solid; m.p. 120–122 °C; TOF = 0.23 h^{-1}; ^{1}H NMR (400 MHz, $CDCl_3$) δ 8.67–8.56 (m, 2H), 8.46 (dd, J = 8.0, 1.6 Hz, 1H), 8.09 (s, 1H), 7.89 (dd, J = 8.0, 1.4 Hz, 1H), 7.69–7.53 (m, 4H), 3.36–3.26 (m, 2H), 2.28–2.20 (m, 2H), 1.59 (s, 6H); ^{13}C NMR (100 MHz, $CDCl_3$) δ 203.6, 135.2, 131.8, 130.8, 130.0, 129.8, 129.3, 128.8, 128.4, 127.5, 127.2, 126.5, 122.9, 122.7, 70.4, 40.0, 38.3, 32.7; HRMS (ESI) calcd. for $C_{20}H_{20}ClO$ (M^+ + H): 311.1202, found: 311.1187; IR (neat, cm^{-1}) 2972, 2928, 1686, 1588, 1443, 1371, 1295, 1273, 1244, 1173, 1148, 1110, 882, 794, 772, 746, 681.

4-Chloro-4-methyl-1-phenyloctan-1-one (2k)

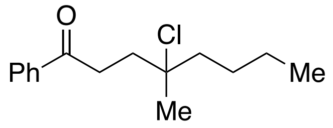

Colourless oil; TOF = 0.33 h^{-1}; ^{1}H NMR (400 MHz, $CDCl_3$) δ 7.96–7.90 (m, 2H), 7.53–7.46 (m, 1H), 7.40 (ddd, *J* = 8.2, 6.6, 1.3 Hz, 2H), 3.15 (ddd, *J* = 8.4, 6.5, 1.3 Hz, 2H), 2.12 (ddd, *J* = 15.5, 8.7, 6.4 Hz, 2H), 1.75 (ddd, *J* = 11.5, 10.3, 5.3 Hz, 2H), 1.50 (s, 4H), 1.46–1.33 (m, 2H), 1.33–1.22 (m, 2H), 0.86 (t, *J* = 7.2 Hz, 3H); ^{13}C NMR (100 MHz, $CDCl_3$) δ 199.5, 136.8, 133.1, 128.6, 128.1, 74.3, 44.4, 37.9, 34.4, 29.7, 26.9, 22.9, 14.0; HRMS (ESI) calcd. for $C_{15}H_{20}ClO$ (M^{+} – H): 251.1202, found: 251.1126; IR (neat, cm^{-1}) 2956, 2933, 2865, 1685, 1597, 1448, 1379, 1297, 1213, 1179, 1002, 742, 689, 657.

4-Chloro-4-ethyl-1-phenyloctan-1-one (2l)

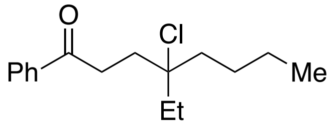

Colourless oil; TOF = 0.20 h^{-1}; ^{1}H NMR (400 MHz, $CDCl_3$) δ 7.96–7.91 (m, 2H), 7.53–7.47 (m, 1H), 7.41 (dd, *J* = 8.2, 6.8 Hz, 2H), 3.15–3.08 (m, 2H), 2.12 (dd, *J* = 9.0, 6.8 Hz, 2H), 1.84–1.70 (m, 4H), 1.36–1.22 (m, 5H), 0.92 (t, *J* = 7.4 Hz, 3H), 0.89–0.82 (m, 3H); ^{13}C NMR (100 MHz, $CDCl_3$) δ 199.6, 136.9, 133.1, 128.6, 128.1, 40.4, 35.1, 34.0, 33.9, 26.5, 22.9, 14.0, 8.9; HRMS (ESI) calcd. for $C_{16}H_{24}ClO$ (M^{+} + H): 267.1515, found: 267.1474; IR (neat, cm^{-1}) 2929, 1675, 1593, 1566, 1499, 1446, 1410, 1374, 1309, 1273, 1252, 1198, 1059, 1012, 823, 786, 698.

4-Chloro-2,4-dimethyl-1-phenylpentan-1-one (2m)

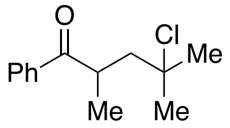

Colourless oil; TOF = 0.28 h^{-1}; ^{1}H NMR (400 MHz, $CDCl_3$) δ 7.99–7.93 (m, 2H), 7.53–7.47 (m, 1H), 7.45–7.38 (m, 2H), 3.84 (ddd, *J* = 8.5, 7.2, 2.3 Hz, 1H), 2.66 (dd, *J* = 14.7, 8.5 Hz, 1H), 1.74 (dd, *J* = 14.7, 2.4 Hz, 1H), 1.56 (s, 3H), 1.31 (s, 3H), 1.16 (d, *J* = 7.2 Hz, 3H); ^{13}C NMR (100 MHz, $CDCl_3$) δ 203.8, 136.2, 133.1, 128.7, 128.4, 71.2, 48.2, 37.7, 34.0, 32.3, 20.5; HRMS (ESI) calcd. for $C_{13}H_{16}ClO$ (M^+ – H): 223.0889, found: 223.0873; IR (neat, cm^{-1}) 2972, 2931, 1682, 1596, 1579, 1448, 1371, 1262, 1224, 1205, 1175, 1110, 973, 705, 687.

3-(1-Chlorocyclopentyl)-1-phenylpropan-1-one (2n)

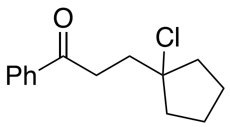

Colourless oil; TOF = 0.18 h^{-1}; ^{1}H NMR (400 MHz, $CDCl_3$) δ 7.94 (dd, *J* = 8.4, 1.4 Hz, 2H), 7.53–7.47 (m, 1H), 7.44–7.38 (m, 2H), 3.27–3.21 (m, 2H), 2.27–2.21 (m, 2H), 2.13–2.05 (m, 2H), 1.95–1.86 (m, 2H), 1.75–1.65 (m, 4H); ^{13}C NMR (100 MHz, $CDCl_3$) δ 199.6, 136.8, 133.1, 128.6, 128.1, 128.1, 82.6, 42.5, 37.4, 35.6, 23.1; HRMS (ESI) calcd. for $C_{14}H_{16}ClO$ (M^+ – H): 235.0889, found: 235.0832; IR (neat, cm^{-1}) 2970, 2925, 1720, 1665, 1619, 1597, 1489, 1447, 1291, 1235, 1110, 1090, 1006, 828, 767, 734, 697, 669.

3-(1-Chlorocyclohexyl)-1-phenylpropan-1-one (2o)

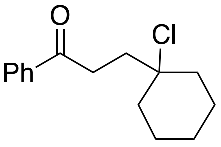

Colourless oil; TOF = 0.17 h^{-1}; ^1H NMR (400 MHz, $CDCl_3$) δ 7.95–7.90 (m, 2H), 7.52–7.46 (m, 1H), 7.40 (dd, *J* = 8.3, 6.9 Hz, 2H), 3.22–3.15 (m, 2H), 2.17–2.10 (m, 2H), 1.92 (ddt, *J* = 13.4, 4.1, 1.7 Hz, 2H), 1.72–1.57 (m, 3H), 1.57–1.44 (m, 4H), 1.24–1.10 (m, 1H); ^{13}C NMR (100 MHz, $CDCl_3$) δ 199.7, 136.9, 133.1, 128.6, 128.6, 128.1, 128.1, 75.5, 39.9, 39.1, 33.6, 25.4, 22.3; HRMS (ESI) calcd. for $C_{15}H_{18}ClO$ (M^+ – H): 249.1046, found: 249.1022; IR (neat, cm^{-1}) 3065, 2933, 2860, 1667, 1613, 1446, 1343, 1301, 1273, 1232, 1211, 1013, 989, 854, 763, 691.

6-Chloro-6-methyl-2-phenylheptan-3-one (2p)

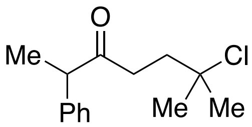

Colourless oil; TOF = 0.28 h^{-1}; ^1H NMR (400 MHz, $CDCl_3$) δ 7.28–7.22 (m, 2H), 7.22–7.17 (m, 1H), 7.17–7.13 (m, 2H), 3.73 (q, *J* = 7.0 Hz, 1H), 2.57–2.49 (m, 2H), 1.99–1.89 (m, 1H), 1.84–1.71 (m, 1H), 1.39 (d, *J* = 23.2 Hz, 6H), 1.33 (d, *J* = 7.0 Hz, 3H); ^{13}C NMR (100 MHz, $CDCl_3$) δ 210.0, 140.5, 128.9, 127.8, 127.2, 70.1, 53.1, 39.3, 37.1, 32.6, 32.2, 17.5; HRMS (ESI) calcd. for $C_{31}H_{31}NO_3S.Na$ (M^+ + Na): 520.1922, found: 520.1374; IR (neat, cm^{-1}) 3259, 2969, 2925, 1685, 1598, 1449, 1370, 1308, 1159, 1106, 1016, 974, 750, 697.

7-Chloro-7-methyl-3-phenyloctan-4-one (2q)

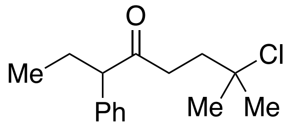

Colourless oil; TOF = 0.32 h^{-1}; ^{1}H NMR (400 MHz, $CDCl_3$) δ 7.28–7.22 (m, 2H), 7.21–7.17 (m, 1H), 7.15 (dd, *J* = 8.2, 1.5 Hz, 2H), 3.50 (t, *J* = 7.4 Hz, 1H), 2.57–2.50 (m, 2H), 2.07–1.90 (m, 2H), 1.83–1.61 (m, 2H), 1.40 (d, *J* = 25.6 Hz, 7H), 0.76 (t, *J* = 7.4 Hz, 3H); ^{13}C NMR (100 MHz, $CDCl_3$) δ 209.7, 138.8, 128.9, 128.3, 127.2, 70.1, 61.0, 39.1, 38.0, 32.6, 32.2, 25.3, 12.1; HRMS (ESI) calcd. for $C_{15}H_{21}O$ (M^+ – Cl): 217.1592, found: 217.1554; IR (neat, cm^{-1}) 2967, 2931, 2875, 1711, 1492, 1453, 1388, 1371, 1285, 1112, 1081, 1029, 909, 731, 700.

Phenyl 4-chloro-4-methylpentanoate (2r)

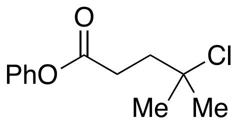

Colourless oil; TOF = 0.25 h^{-1}; ^{1}H NMR (400 MHz, $CDCl_3$) δ 7.33–7.26 (m, 2H), 7.18–7.11 (m, 1H), 7.03–6.98 (m, 2H), 2.78–2.68 (m, 2H), 2.17–2.07 (m, 2H), 1.55 (s, 6H); ^{13}C NMR (100 MHz, $CDCl_3$) δ 171.7, 150.7, 129.4, 125.8, 121.5, 69.5, 40.3, 32.4, 30.6; HRMS (ESI) calcd. for $C_{12}H_{15}O_2$ (M^+ – Cl): 191.1072, found: 191.1052; IR (neat, cm^{-1}) 2973, 2926, 1756, 1593, 1493, 1372, 1292, 1238, 1195, 1162, 1132, 1131, 1024, 931, 815, 752, 688.

4-Chloro-1-phenylpentan-1-one (2s)

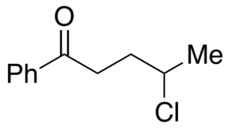

Colourless liquid; TOF = 0.05 h^{-1}; ^{1}H NMR (400 MHz, $CDCl_3$) δ 7.91 (dt, *J* = 8.5, 1.4 Hz, 2H), 7.49 (td, *J* = 7.3, 1.4 Hz, 1H), 7.39 (ddd, *J* = 8.7, 6.9, 1.5 Hz, 2H), 4.09 (dddd, *J* = 9.8, 6.7, 3.4, 1.3 Hz, 1H), 3.20–3.08 (m, 2H), 2.26–2.12 (m, 1H), 2.04–

1.88 (m, 1H), 1.51 (dd, J = 6.6, 1.4 Hz, 3H); ^{13}C NMR (100 MHz, $CDCl_3$) δ 199.2, 136.8, 133.2, 128.6, 128.0, 58.5, 35.6, 34.3, 25.7; HRMS (ESI) calcd. for $C_{11}H_{13}O$ (M^+ – Cl): 161.0966, found: 161.0960; IR (neat, cm^{-1}) 3305, 3109, 2208, 1594, 1477, 1383, 1324, 1221, 1159, 1108, 1020, 809, 782, 716.

4-Chloro-1-(4-(trifluoromethyl)phenyl)pentan-1-one (2t)

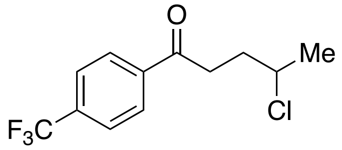

Colourless solid; m.p. 34-35 °C; TOF = 0.05 h^{-1}; ^{1}H NMR (400 MHz, $CDCl_3$) δ 8.08–7.98 (m, 2H), 7.67 (d, J = 8.2 Hz, 2H), 4.10 (ddd, J = 9.8, 6.5, 3.3 Hz, 1H), 3.21–3.12 (m, 2H), 2.22 (dddd, J = 14.8, 7.9, 6.9, 3.3 Hz, 1H), 1.97 (dddd, J = 14.6, 9.7, 7.6, 5.8 Hz, 1H), 1.53 (d, J = 6.6 Hz, 3H); ^{13}C NMR (100 MHz, $CDCl_3$) δ 198.2, 152.8, 139.4, 134.4 (q, $J_{C\text{-}F}$ = 32.7 Hz), 128.4, 125.7 (q, $J_{C\text{-}F}$ = 3.8 Hz), 58.2, 35.9, 34.1, 25.6; HRMS (ESI) calcd. for $C_{12}H_{13}ClF_3O$ (M^+ + H): 265.0607, found: 265.0662; IR (neat, cm^{-1}) 2976, 2929, 1689, 1410, 1323, 1267, 1168, 1129, 1066, 1016, 850.

4-Chloro-1-(4-chlorophenyl)pentan-1-one (2u)

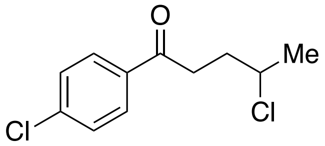

Colourless oil; TOF = 0.05 h^{-1}; ^{1}H NMR (400 MHz, $CDCl_3$) δ 7.90–7.81 (m, 2H), 7.43–7.32 (m, 2H), 4.08 (ddd, J = 9.8, 6.5, 3.3 Hz, 1H), 3.15–3.03 (m, 2H), 2.20 (dddd, J = 14.8, 8.0, 6.9, 3.4 Hz, 1H), 1.94 (dddd, J = 14.5, 9.6, 7.6, 5.8 Hz, 1H), 1.51 (d, J = 6.5 Hz, 3H); ^{13}C NMR (100 MHz, $CDCl_3$) δ 197.9, 129.4, 139.6, 135.1, 129.4, 128.9, 58.3, 35.5, 34.2, 25.6; HRMS (ESI) calcd. for $C_{11}H_{13}Cl_2O$ (M^+ + H): 231.0343, found: 231.02973; IR (neat, cm^{-1}) 2969, 2928, 1685, 1589, 1400, 1341, 1400, 1341, 1279, 1206, 1091, 1012, 981, 836, 813, 781.

4-Chloro-1-(3-fluorophenyl)pentan-1-one (2v)

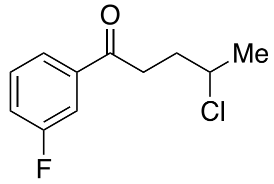

Colourless oil; TOF = 0.05 h^{-1}; ^{1}H NMR (400 MHz, $CDCl_3$) δ 7.70 (dt, J = 7.7, 1.3 Hz, 1H), 7.59 (ddd, J = 9.5, 2.6, 1.6 Hz, 1H), 7.38 (td, J = 8.0, 5.5 Hz, 1H), 7.23–7.17 (m, 1H), 4.09 (ddd, J = 9.8, 6.5, 3.4 Hz, 1H), 3.16–3.06 (m, 2H), 2.20 (dddd, J = 14.8, 8.0, 6.9, 3.4 Hz, 1H), 2.02–1.90 (m, 1H), 1.52 (d, J = 6.6 Hz, 3H); ^{13}C NMR (100 MHz, $CDCl_3$) δ 197.9 (d, $J_{C\text{-}F}$ = 2.2 Hz), 138.8 (d, $J_{C\text{-}F}$ = 6.1 Hz), 130.3 (d, $J_{C\text{-}F}$ = 7.5 Hz), 123.8 (d, $J_{C\text{-}F}$ = 3.1 Hz), 120.2 (d, $J_{C\text{-}F}$ = 21.5 Hz), 114.7 (d, $J_{C\text{-}F}$ = 22.3 Hz), 58.3, 35.7, 34.2, 25.6; HRMS (ESI) calcd. for $C_{11}H_{12}FO$ (M^+ – Cl): 179.0872, found: 179.0865; IR (neat, cm^{-1}) 2959, 2935, 1692, 1469, 1411, 1322, 1206, 1167, 1127, 1016, 846, 833, 777, 731.

4-Chloro-1-(3-chloro-4-methoxyphenyl)pentan-1-one (2w)

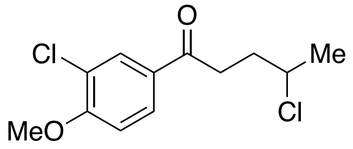

Colourless oil; TOF = 0.05 h^{-1}; ^{1}H NMR (400 MHz, $CDCl_3$) δ 7.95 (d, J = 2.1 Hz, 1H), 7.83 (dd, J = 8.7, 2.2 Hz, 1H), 6.90 (d, J = 8.6 Hz, 1H), 4.08 (ddd, J = 9.7, 6.5, 3.3 Hz, 1H), 3.90 (s, 3H), 3.10–3.03 (m, 2H), 2.24–2.14 (m, 1H), 2.00–1.89 (m, 1H), 1.51 (d, J = 6.5 Hz, 3H); ^{13}C NMR (100 MHz, $CDCl_3$) δ 196.8, 158.8, 130.4, 130.4, 128.5, 123.0, 111.3, 58.4, 56.4, 35.2, 34.3, 25.6; HRMS (ESI) calcd. for $C_{12}H_{16}Cl_2O_2$ (M^+ + H): 261.0449, found: 261.0436; IR (neat, cm^{-1}) 2971, 2928, 1674, 1593, 1565, 1498, 1410, 1374, 1309, 1272, 1250, 1198, 1059, 1011, 892, 823, 786, 697.

6-Chloro-2-phenylheptan-3-one (2x)

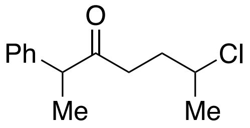

Colourless oil; TOF = 0.08 h^{-1}; ^{1}H NMR (400 MHz, $CDCl_3$) δ 7.26 (dd, *J* = 8.1, 6.6 Hz, 2H), 7.21–7.16 (m, 1H), 7.16–7.13 (m, 2H), 3.88–3.77 (m, 1H), 3.70 (qd, *J* = 7.0, 1.9 Hz, 1H), 2.50 (tt, *J* = 6.8, 0.8 Hz, 2H), 2.01–1.83 (m, 1H), 1.81–1.62 (m, 1H), 1.37 (dd, *J* = 9.8, 6.5 Hz, 3H), 1.33 (d, *J* = 7.0 Hz, 3H); ^{13}C NMR (100 MHz, $CDCl_3$) δ 210.0, 209.9, 140.5, 140.4, 129.0, 129.0, 128.8, 128.0, 127.8, 127.2, 58.1, 58.1, 53.2, 53.1, 38.1, 37.8, 34.0, 25.5, 25.3, 17.4, 17.4; HRMS (ESI) calcd. for $C_{13}H_{18}ClO$ (M^{+} + H): 225.1046, found: 225.1063; IR (neat, cm^{-1}) 2973, 2930, 1741, 1712, 1493, 1452, 1374, 1232, 1068, 1030, 759, 699.

Phenyl 4-Chloro-4-methylpentanoate (2y)

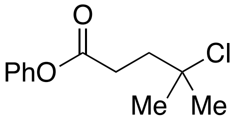

Colourless oil; TOF = 0.21 h^{-1}; ^{1}H NMR (400 MHz, $CDCl_3$) δ 7.33–7.26 (m, 2H), 7.18–7.11 (m, 1H), 7.04–6.98 (m, 2H), 2.77–2.70 (m, 2H), 2.17–2.08 (m, 2H), 1.55 (s, 6H); ^{13}C NMR (100 MHz, $CDCl_3$) δ 171.7, 150.7, 129.4, 125.8, 121.5, 69.5, 40.3, 32.4, 30.6; HRMS (ESI) calcd. for $C_{12}H_{15}O_2$ (M^{+} – Cl): 191.1072, found: 191.1052; IR (neat, cm^{-1}) 2973, 2926, 1756, 1593, 1493, 1372, 1292, 1238, 1195, 1162, 1132, 1131, 1024, 931, 815, 752, 688.

2-(1,3-Dioxoisoindolin-2-yl)ethyl 4-chloro-4-methylpentanoate (2z)

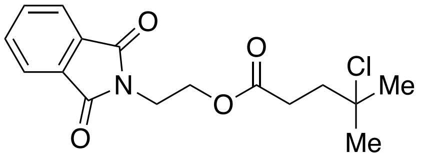

colourless liquid; TOF = 0.21 h^{-1}; ^{1}H NMR (400 MHz, $CDCl_3$) δ 7.78 (dt, *J* = 7.2, 3.6 Hz, 2H), 7.66 (dd, *J* = 5.6, 3.1 Hz, 2H), 4.27 (t, *J* = 5.3 Hz, 2H), 3.90 (t, *J* = 5.3 Hz, 2H), 2.44 (dd, *J* = 9.7, 6.2 Hz, 2H), 2.02–1.92 (m, 2H), 1.47 (s, 6H); ^{13}C NMR (100 MHz, $CDCl_3$) δ 173.0, 168.1, 134.1, 132.0, 123.4, 69.5, 69.5, 61.7, 40.2, 37.0, 32.3, 30.2; HRMS (ESI) calcd. for $C_{16}H_{19}ClNO_4$ (M^+ + H): 324.1003, found: 324.1012; IR (neat, cm^{-1}) 2927, 2256, 1775, 1711, 1428, 1391, 1158, 1113, 719.

4-Chloro-*N*,4-dimethyl-*N*-phenylpentanamide (2*α*)

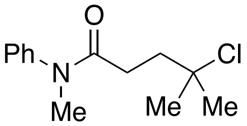

Yellow oil; TOF = 0.17 h^{-1}; ^{1}H NMR (400 MHz, $CDCl_3$) δ 7.36 (dd, *J* = 8.3, 6.8 Hz, 2H), 7.29 (d, *J* = 7.3 Hz, 1H), 7.16–7.11 (m, 2H), 3.20 (s, 3H), 2.22 (dd, *J* = 9.9, 6.0 Hz, 2H), 2.04–1.94 (m, 2H), 1.38 (s, 6H); ^{13}C NMR (100 MHz, $CDCl_3$) δ 172.4, 143.9, 129.8, 127.9, 127.2, 70.2, 41.1, 37.4, 32.3, 30.2; HRMS (ESI) calcd. for $C_{13}H_{19}ClNO$ (M^+ + H): 240.1155, found: 240.1109; IR (neat, cm^{-1}) 2970, 2927, 1654, 1595, 1540, 1495, 1451, 1420, 1384, 1348, 1289, 1165, 1109, 1088, 1073, 772, 730, 699.

(*E*)-4-Chloro-4-methyl-1-phenylpent-2-en-1-one (4a)

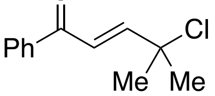

Colourless oil; TOF = 0.34 h^{-1}; ^{1}H NMR (400 MHz, $CDCl_3$) δ 7.87 (dt, *J* = 8.3, 1.2 Hz, 2H), 7.54–7.46 (m, 1H), 7.45–7.36 (m, 2H), 7.00 (dd, *J* = 3.3, 1.1 Hz, 2H), 1.71

(s, 6H); ^{13}C NMR (100 MHz, $CDCl_3$) δ 190.6, 151.4, 137.5, 133.1, 128.7, 128.6, 122.7, 66.6, 31.9; HRMS (ESI) calcd. for $C_{12}H_{14}ClO$ (M^+ + H): 209.0733, found: 209.0717; IR (neat, cm^{-1}) 2956, 2868, 1758, 1673, 1599, 1458, 1447, 1363, 1297, 1255, 1232, 1167, 892, 695.

4-Chloro-*N*,4-dimethyl-*N*-phenylpentanamide (4b)

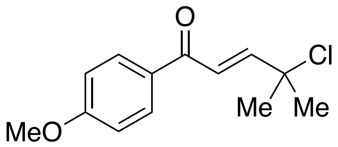

Colourless oil; TOF = 0.29 h^{-1}; ^{1}H NMR (400 MHz, $CDCl_3$) δ 7.88 (d, *J* = 8.9 Hz, 2H), 6.98 (s, 2H), 6.88 (d, *J* = 8.9 Hz, 2H), 3.80 (s, 3H), 1.71 (s, 6H); ^{13}C NMR (100 MHz, $CDCl_3$) δ 188.8, 163.7, 150.5, 131.0, 130.5, 122.5, 113.9, 66.8, 55.5, 32.0; HRMS (ESI) calcd. for $C_{13}H_{16}ClO_2$ (M^+ + H): 239.0838, found: 239.0833; IR (neat, cm^{-1}) 2956, 2871, 1670, 1655, 1606, 1351, 1304, 1221, 1015, 982, 827, 695.

(*E*)-4-Chloro-4-methyl-1-(*p*-tolyl)pent-2-en-1-one (4c)

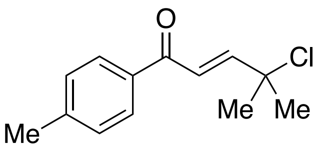

Colourless oil; TOF = 0.32 h^{-1}; ^{1}H NMR (400 MHz, $CDCl_3$) δ 7.89 (d, *J* = 8.3 Hz, 2H), 7.33–7.28 (m, 2H), 7.09 (d, *J* = 1.7 Hz, 2H), 2.45 (s, 3H), 1.81 (s, 6H); ^{13}C NMR (100 MHz, $CDCl_3$) δ 190.1, 151.0, 144.0, 135.0, 129.4, 128.8, 122.7, 66.7, 31.9, 21.7; HRMS (ESI) calcd. for $C_{13}H_{16}ClO$ (M^+ + H): 223.0889, found: 223.0875; IR (neat, cm^{-1}) 2975, 2926, 1671, 1622, 1603, 1371, 1294, 1259, 1173, 1109, 1032, 1015, 810, 698.

(*E*)-1-([1,1'-Biphenyl]-4-yl)-4-chloro-4-methylpent-2-en-1-one (4d)

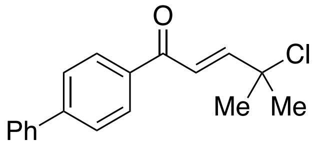

Colourless oil; TOF = 0.32 h^{-1}; ^{1}H NMR (400 MHz, $CDCl_3$) δ 7.99–7.93 (m, 2H), 7.66–7.60 (m, 2H), 7.58–7.52 (m, 2H), 7.43–7.36 (m, 2H), 7.36–7.28 (m, 1H), 7.03 (d, J = 0.7 Hz, 2H), 1.73 (s, 6H); ^{13}C NMR (100 MHz, $CDCl_3$) δ 190.0, 151.3, 145.9, 139.8, 136.3, 129.3, 129.0, 128.3, 127.3, 127.3, 122.7, 66.7, 32.0; HRMS (ESI) calcd. for $C_{18}H_{18}ClO$ (M^+ + H): 285.1046, found: 285.1040; IR (neat, cm^{-1}) 2971, 1664, 1618, 1698, 1328, 1290, 1254, 1219, 1109, 1208, 1005, 971, 830, 767, 733, 683.

(*E*)-4-Chloro-4-methyl-1-(4-(trifluoromethyl)phenyl)pent-2-en-1-one (4e)

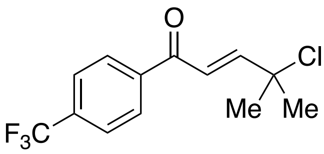

Colourless oil; TOF = 0.35 h^{-1}; ^{1}H NMR (400 MHz, $CDCl_3$) δ 7.99–7.94 (m, 2H), 7.68 (dt, J = 8.0, 0.7 Hz, 2H), 7.09–6.91 (m, 2H), 1.73 (s, 6H); ^{13}C NMR (100 MHz, $CDCl_3$) δ 189.7, 152.7, 140.3, 134.3 (d, $J_{C\text{-}F}$ = 32.8 Hz), 128.9, 125.7 (d, $J_{C\text{-}F}$ = 3.8 Hz), 122.4, 66.4, 31.8.; HRMS (ESI) calcd. for $C_{13}H_{11}ClF_3O$ (M^+ – H): 275.0450, found: 275.0433; IR (neat, cm^{-1}) 2977, 2927, 1664, 1618, 1318, 1292, 1218, 1166, 1128, 1109, 1066, 1014, 972, 830, 766, 733, 696.

(*E*)-4-Chloro-1-(2,4-dimethylphenyl)-4-methylpent-2-en-1-one (4f)

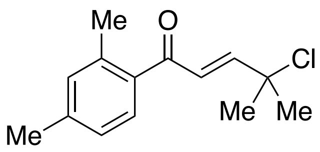

Colourless oil; TOF = 0.26 h^{-1}; ^{1}H NMR (400 MHz, $CDCl_3$) δ 7.37–7.32 (m, 1H), 7.02–6.96 (m, 2H), 6.79–6.57 (m, 2H), 2.35 (s, 3H), 2.29 (s, 3H), 1.69 (s, 6H); ^{13}C NMR (100 MHz, $CDCl_3$) δ 195.3, 151.5, 141.5, 137.9, 135.4, 132.4, 129.0, 126.7,

126.2, 66.4, 31.7, 21.4, 20.6; HRMS (ESI) calcd. for $C_{14}H_{18}ClO$ (M^+ + H): 237.1046, found: 237.1042; IR (neat, cm^{-1}) 2973, 2924, 1664, 1618, 1598, 1447, 1290, 1255, 1218, 1109, 971, 829, 776, 733, 697, 683.

(*E*)-4-Chloro-4-methyl-1-(naphthalen-2-yl)pent-2-en-1-one (4g)

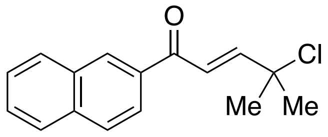

White solid; m.p. 110–112 °C; TOF = 0.16 h^{-1}; ^1H NMR (400 MHz, $CDCl_3$) δ 8.42–8.36 (m, 1H), 7.99–7.88 (m, 2H), 7.88–7.78 (m, 2H), 7.57–7.46 (m, 2H), 7.19–7.04 (m, 2H), 1.76 (s, 6H); ^{13}C NMR (100 MHz, $CDCl_3$) δ 190.3, 151.4, 135.6, 134.9, 132.5, 130.3, 129.6, 128.7, 128.6, 127.8, 126.9, 124.4, 122.7, 66.7, 32.0; HRMS (ESI) calcd. for $C_{16}H_{16}ClO$ (M^+ + H): 259.0889, found: 259.0878; IR (neat, cm^{-1}) 2979, 2928, 1667, 1618, 1458, 1352, 1295, 1188, 1124, 1109, 1014, 986, 823, 770, 752.

(*E*)-4-Chloro-4-methyl-1-(thiophen-2-yl)pent-2-en-1-one (4h)

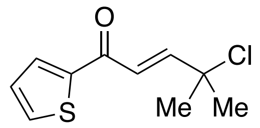

Colourless oil; TOF = 0.15 h^{-1}; ^1H NMR (400 MHz, $CDCl_3$) δ 7.74 (dd, *J* = 3.8, 1.1 Hz, 1H), 7.62 (dd, *J* = 4.9, 1.1 Hz, 1H), 7.10 (dd, *J* = 5.0, 3.8 Hz, 1H), 7.07 (d, *J* = 15.2 Hz, 1H), 6.90 (d, *J* = 15.2 Hz, 1H), 1.72 (s, 6H); ^{13}C NMR (100 MHz, $CDCl_3$) δ 182.1, 150.7, 144.9, 134.4, 132.4, 128.3, 122.5, 66.5, 32.0; HRMS (ESI) calcd. for $C_{10}H_{12}ClOS$ (M^+ + H): 215.0297, found: 215.0292; IR (neat, cm^{-1}) 2987, 2255, 1664, 1619, 1516, 1415, 1297, 1266, 1112, 971, 908, 696.

(*E*)-4-Chloro-4-methyl-1-phenylhex-2-en-1-one (4i)

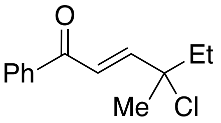

Colourless oil; TOF = 0.37 h^{-1}; ^{1}H NMR (400 MHz, $CDCl_3$) δ 7.89 (dd, *J* = 8.4, 1.4 Hz, 2H), 7.54–7.47 (m, 1H), 7.46–7.38 (m, 2H), 7.10–6.88 (m, 2H), 1.89 (q, *J* = 7.3 Hz, 2H), 1.67 (s, 3H), 0.96 (t, *J* = 7.3 Hz, 3H); ^{13}C NMR (100 MHz, $CDCl_3$) δ 190.4, 150.8, 137.6, 133.1, 128.7, 128.6, 128.5, 124.2, 71.6, 37.2, 29.5, 9.4; HRMS (ESI) calcd. for $C_{13}H_{16}ClO$ (M^+ + H): 223.0889, found: 223.0866; IR (neat, cm^{-1}) 2971, 2933, 1667, 1600, 1575, 1507, 1457, 1420, 1396, 1296, 1255, 1216, 1197, 1171, 1108, 1208, 978, 838, 808, 781.

(*E*)-4-Chloro-4-ethyl-1-phenylhex-2-en-1-one (4j)

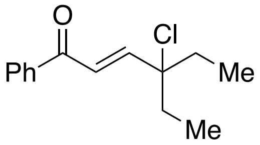

Colourless oil; TOF = 0.26 h^{-1}; ^{1}H NMR (400 MHz, $CDCl_3$) δ 7.93–7.88 (m, 2H), 7.49 (d, *J* = 7.5 Hz, 1H), 7.44–7.38 (m, 2H), 7.14 (d, *J* = 15.0 Hz, 1H), 6.82 (d, *J* = 15.1 Hz, 1H), 1.97–1.80 (m, 4H), 0.93 (t, *J* = 7.3 Hz, 6H); ^{13}C NMR (100 MHz, $CDCl_3$) δ 190.0, 149.8, 137.6, 133.1, 128.6, 128.6, 128.5, 128.5, 126.2, 35.3, 9.1; HRMS (ESI) calcd. for $C_{14}H_{18}ClO$ (M^+ + H): 237.1046, found: 237.1042; IR (neat, cm^{-1}) 2971, 2928, 1667, 1660, 1448, 1374, 1296, 1221, 1159, 1108, 1020, 809, 782.

(*E*)-3-(1-Chlorocyclohexyl)-1-phenylprop-2-en-1-one (4k)

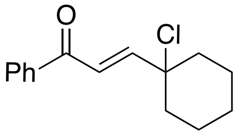

Colourless solid; m.p. 67–68 °C; TOF = 0.34 h^{-1}; ^{1}H NMR (400 MHz, $CDCl_3$) δ 7.91–7.85 (m, 2H), 7.53–7.47 (m, 1H), 7.41 (dd, *J* = 8.4, 6.9 Hz, 2H), 7.08–6.96 (m,

2H), 1.98 (dt, J = 6.6, 3.5 Hz, 2H), 1.82–1.69 (m, 4H), 1.67–1.52 (m, 3H), 1.28–1.16 (m, 1H); ^{13}C NMR (100 MHz, $CDCl_3$) δ 190.7, 151.5, 137.6, 133.1, 128.6, 123.2, 71.6, 39.0, 25.0, 22.0; HRMS (ESI) calcd. for $C_{15}H_{18}ClO$ (M^+ + H): 249.1046, found: 249.1022; IR (neat, cm^{-1}) 3258, 2929, 1593, 1448, 1374, 1296, 1172, 1159, 1096, 1080, 1016, 811, 698.

1-(3-(Chloromethyl)phenyl)ethan-1-one (6b)

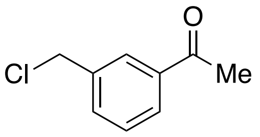

Colourless oil; TOF = 0.29 h^{-1}; ^{1}H NMR (400 MHz, $CDCl_3$) δ 7.98 (t, J = 1.9 Hz, 1H), 7.91 (dt, J = 7.8, 1.4 Hz, 1H), 7.61 (dd, J = 7.6, 1.7 Hz, 1H), 7.48 (t, J = 7.7 Hz, 1H), 4.64 (s, 2H), 2.62 (s, 3H); ^{13}C NMR (100 MHz, $CDCl_3$) δ 197.6, 138.1, 137.6, 133.1, 129.1, 128.3, 128.3, 45.6, 26.6; HRMS (ESI) calcd. for $C_9H_{10}ClO$ (M^+ + H): 169.0420, found: 169.0391; IR (neat, cm^{-1}) 1681, 1602, 1585, 1440, 1357, 1280, 1258, 1191, 1164, 798, 704, 688.

1-(4-(Chloromethyl)phenyl)ethan-1-one (6c)

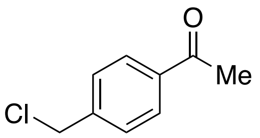

Colourless oil; TOF = 0.24 h^{-1}; ^{1}H NMR (400 MHz, $CDCl_3$) δ 7.87 (d, J = 8.1 Hz, 2H), 7.40 (d, J = 8.1 Hz, 2H), 4.53 (s, 2H), 2.52 (s, 3H); ^{13}C NMR (100 MHz, $CDCl_3$) δ 197.5, 142.4, 137.0, 128.8, 128.8, 128.7, 126.5, 45.3, 26.6; HRMS (ESI) calcd. for $C_9H_{10}ClO$ (M^+ + H): 169.0420, found: 169.0391; IR (neat, cm^{-1}) 1680, 1607, 1572, 1410, 1357, 1324, 1263, 1182, 1164, 1066, 1016, 958, 821, 743, 696, 670.

(4-(Chloromethyl)phenyl)(phenyl)methanone (6d)

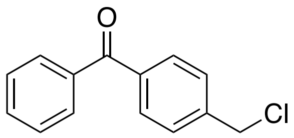

White solid; m.p. 58–60 °C; TOF = 0.23 h^{-1}; ^1H NMR (400 MHz, $CDCl_3$) δ 7.78–7.68 (m, 4H), 7.57–7.50 (m, 1H), 7.48–7.39 (m, 4H), 4.58 (s, 2H); ^{13}C NMR (100 MHz, $CDCl_3$) δ 196.1, 141.7, 137.5, 137.4, 132.6, 130.5, 130.0, 128.4, 128.4, 45.4; HRMS (ESI) calcd. for $C_{14}H_{12}ClO$ (M^+ + H): 231.0576, found: 231.0593; IR (neat, cm^{-1}) 3065, 2933, 2959, 1647, 1610, 1594, 1573, 1449, 1414, 1320, 1280, 1267, 1178, 1148, 944, 928, 853, 749, 701, 688.

1-(4-(1-Chloroethyl)phenyl)ethan-1-one (6e)

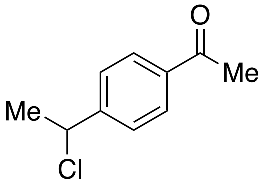

Colourless oil; TOF = 0.38 h^{-1}; ^1H NMR (400 MHz, $CDCl_3$) δ 7.85 (d, J = 8.4 Hz, 2H), 7.42 (d, J = 8.3 Hz, 2H), 5.02 (q, J = 6.8 Hz, 1H), 2.50 (d, J = 1.1 Hz, 3H), 1.76 (d, J = 6.8 Hz, 3H); ^{13}C NMR (100 MHz, $CDCl_3$) δ 197.4, 147.7, 136.9, 128.7, 128.4, 126.7, 125.8, 57.7, 26.6, 26.4; HRMS (ESI) calcd. for $C_{10}H_{12}ClO$ (M^+ + H): 183.0576, found: 183.0547; IR (neat, cm^{-1}) 2979, 1681, 1608, 1413, 1357, 1263, 1046, 1015, 957, 832, 739, 681.

4-(1-Chloroethyl)-*N*-methoxy-*N*-methylbenzamide (6f)

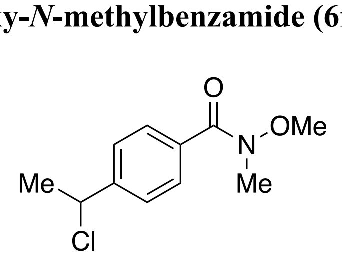

Pale yellow oil; TOF = 0.31 h^{-1}; ^1H NMR (400 MHz, $CDCl_3$) δ 7.71–7.65 (m, 2H), 7.48–7.42 (m, 2H), 5.10 (q, J = 6.8 Hz, 1H), 3.56 (s, 3H), 3.36 (s, 3H), 1.85 (d, J =

6.9 Hz, 3H); ^{13}C NMR (100 MHz, $CDCl_3$) δ 197.7, 154.6, 149.6, 136.1, 128.5, 127.7, 79.5, 64.4, 50.3, 28.4, 26.6, 19.4; HRMS (ESI) calcd. for $C_{11}H_{15}ClNO_2$ (M^+ + H): 228.0791, found: 228.0771; IR (neat, cm^{-1}) 2980, 2935, 1620, 1459, 1414, 1221, 1181, 1056, 982, 827, 686.

3-Chloro-3-phenylpropyl acetate (6g)

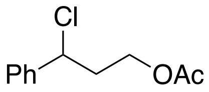

Yellow oil; TOF = 0.35 h^{-1}; ^1H NMR (400 MHz, $CDCl_3$) δ 7.32–7.22 (m, 5H), 4.92 (dd, *J* = 8.7, 5.9 Hz, 1H), 4.16 (ddd, *J* = 11.3, 7.7, 5.5 Hz, 1H), 4.06 (dt, *J* = 11.4, 5.8 Hz, 1H), 2.38–2.21 (m, 2H), 1.96 (s, 3H); ^{13}C NMR (100 MHz, $CDCl_3$) δ 170.8, 140.9, 128.8, 128.6, 126.9, 61.5, 59.9, 38.7, 20.9; HRMS (ESI) calcd. for $C_{11}H_{14}ClO_2$ (M^+ + H): 213.0682, found: 213.0689; IR (neat, cm^{-1}) 2963, 1737, 1454, 1365, 1229, 1036, 759, 697.

1-(4-(1-Chloropentyl)phenyl)pentan-1-one (6h)

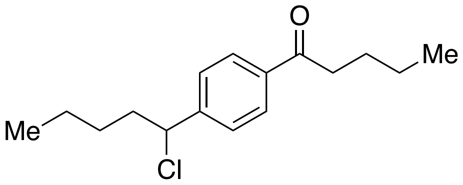

Colourless oil; TOF = 0.25 h^{-1}; ^1H NMR (400 MHz, $CDCl_3$) δ 7.89–7.85 (m, 2H), 7.39 (d, *J* = 8.3 Hz, 2H), 4.79 (dd, *J* = 8.0, 6.5 Hz, 1H), 2.92–2.83 (m, 2H), 2.10–1.89 (m, 2H), 1.70–1.59 (m, 2H), 1.44–1.17 (m, 6H), 0.88 (t, *J* = 7.3 Hz, 3H), 0.82 (t, *J* = 7.1 Hz, 3H); ^{13}C NMR (100 MHz, $CDCl_3$) δ 199.9, 146.7, 136.8, 130.2, 128.4, 127.2, 127.1, 62.8, 39.6, 38.4, 29.1, 26.5, 22.5, 22.1, 13.9, 13.9; HRMS (ESI) calcd. for $C_{16}H_{24}ClO$ (M^+ + H): 267.1515, found: 267.1485; IR (neat, cm^{-1}) 2958, 2931, 2871, 2340, 2103, 1685, 1608, 1466, 1409, 1379, 1265, 1210, 1174, 1012, 977, 733.

Methyl 4-(1-chloropentyl)benzoate (6i)

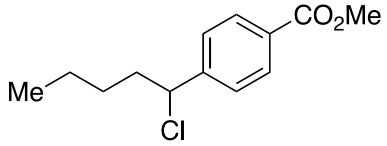

Pale yellow oil; TOF = 0.31 h^{-1}; ^{1}H NMR (400 MHz, $CDCl_3$) δ 8.08–8.01 (m, 2H), 7.50–7.44 (m, 2H), 4.88 (dd, *J* = 8.0, 6.5 Hz, 1H), 3.93 (d, *J* = 1.2 Hz, 3H), 2.20–1.98 (m, 2H), 1.55–1.23 (m, 4H), 0.96–0.86 (m, 3H); ^{13}C NMR (100 MHz, $CDCl_3$) δ 166.6, 146.8, 130.2, 129.9, 129.9, 127.0, 127.0, 62.8, 52.1, 39.6, 29.1, 22.1, 13.9; HRMS (ESI) calcd. for $C_{13}H_{18}ClO_2$ (M^+ + H): 241.0995, found: 241.0987; IR (neat, cm^{-1}) 2954, 2862, 1720, 1161, 1435, 1416, 1379, 1274, 1175, 1110, 1019, 809, 771, 724, 704.

4-(1-Chloropentyl)-*N*-methoxy-*N*-methylbenzamide (6j)

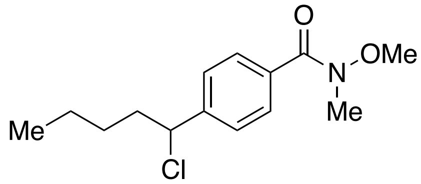

Yellow oil; TOF = 0.26 h^{-1}; ^{1}H NMR (400 MHz, $CDCl_3$) δ 7.56–7.51 (m, 2H), 7.27 (d, *J* = 8.2 Hz, 2H), 4.72 (dd, *J* = 8.0, 6.5 Hz, 1H), 3.42 (s, 3H), 3.22 (s, 3H), 2.04–1.83 (m, 2H), 1.40–1.09 (m, 5H), 0.75 (t, *J* = 7.1 Hz, 3H); ^{13}C NMR (150 MHz, $CDCl_3$) δ 169.3, 144.4, 133.8, 128.6, 126.7, 63.1, 61.1, 39.7, 29.1, 22.1, 13.9, 13.9; HRMS (ESI) calcd. for $C_{14}H_{21}ClNO_2$ (M^+ + H): 270.1260, found: 270.1220; IR (neat, cm^{-1}) 2958, 2933, 2865, 1637, 1458, 1419, 1376, 1214, 1166, 1110, 979, 849, 824, 732, 695.

3-Chloro-2,3-dihydro-1*H*-inden-1-one (6k)

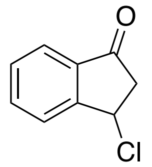

Pale yellow oil; TOF = 0.26 h^{-1}; ^{1}H NMR (400 MHz, $CDCl_3$) δ 7.70 (dd, *J* = 7.7, 1.1 Hz, 1H), 7.67–7.64 (m, 2H), 7.47–7.41 (m, 1H), 5.45 (dd, *J* = 7.2, 3.0 Hz, 1H), 3.26 (dd, *J* = 19.4, 7.2 Hz, 1H), 2.88 (dd, *J* = 19.4, 3.0 Hz, 1H); ^{13}C NMR (100 MHz, $CDCl_3$) δ 201.3, 153.5, 136.2, 135.6, 129.8, 126.9, 123.3, 52.4, 47.7; HRMS (ESI) calcd. for C_9H_8ClO (M^+ + H): 167.0263, found: 167.0252; IR (neat, cm^{-1}) 1718, 1603, 1465, 1277, 1251, 1229, 1174, 1041, 1014, 928, 850, 759.

4-Chloro-3,4-dihydronaphthalen-1(2*H*)-one (6l)

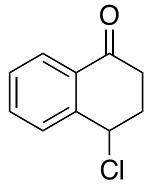

Pale yellow oil; TOF = 0.35 h^{-1}; ^{1}H NMR (400 MHz, $CDCl_3$) δ 7.97 (dd, *J* = 7.9, 1.3 Hz, 1H), 7.51 (td, *J* = 7.5, 1.4 Hz, 1H), 7.45–7.33 (m, 2H), 5.30 (t, *J* = 3.9 Hz, 1H), 3.08 (ddd, *J* = 17.0, 11.7, 5.0 Hz, 1H), 2.67–2.57 (m, 1H), 2.53 (dt, *J* = 11.9, 3.8 Hz, 1H), 2.49–2.41 (m, 1H); ^{13}C NMR (100 MHz, $CDCl_3$) δ 196.3, 142.2, 134.2, 131.1, 129.3, 129.0, 127.4, 56.6, 34.0, 31.8; HRMS (ESI) calcd. for $C_{10}H_{10}ClO$ (M^+ + H): 181.0420, found: 181.0390; IR (neat, cm^{-1}) 2959, 1687, 1598, 1455, 1411, 1323, 1285, 1234, 1205, 920, 881, 778, 720, 700.

(1-Chloro-4-methylpentyl)benzene (6m)[25]

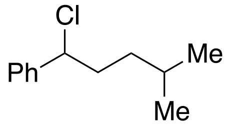

Colourless liquid; TOF = 0.29 h^{-1}; ^{1}H NMR (400 MHz, $CDCl_3$) δ 7.48–7.30 (m, 5H), 4.85 (t, *J* = 7.3 Hz, 1H), 2.23–2.01 (m, 2H), 1.60 (td, *J* = 13.3, 6.6 Hz, 1H), 1.49–1.36 (m, 1H), 1.26–1.14 (m, 1H), 0.92 (d, *J* = 6.6 Hz, 6H); ^{13}C NMR (100 MHz, $CDCl_3$) δ 142.0, 128.6, 128.2, 126.9, 64.2, 38.0, 36.2, 27.7, 22.5, 22.5.

***tert*-Butyl 4-(1-(4-Acetylphenyl)ethyl)piperazine-1-carboxylate (7)**

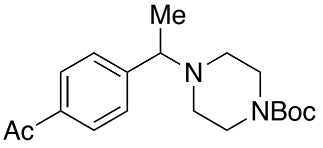

Pale yellow oil; ^{1}H NMR (400 MHz, $CDCl_3$) δ 7.83 (d, *J* = 8.3 Hz, 2H), 7.34 (d, *J* = 8.3 Hz, 2H), 3.33 (dt, *J* = 10.4, 6.0 Hz, 5H), 2.51 (s, 3H), 2.30 (dq, *J* = 45.3, 5.9 Hz, 4H), 1.36 (s, 9H), 1.28 (d, *J* = 6.7 Hz, 3H); ^{13}C NMR (100 MHz, $CDCl_3$) δ 197.7, 154.6, 149.6, 136.1, 128.5, 127.7, 79.5, 64.4, 50.3, 28.4, 26.6, 19.4; HRMS (ESI) calcd. for $C_{19}H_{29}N_2O_3$ (M^+ + H): 333.2178, found: 333.2196; IR (neat, cm^{-1}) 2975, 1682, 1607, 1413, 1364, 1263, 1246, 1166, 1123, 1097, 1002, 957, 835, 768.

4-Chloro-1-(2-chloropyridin-4-yl)-4-methylpentan-1-one (8)

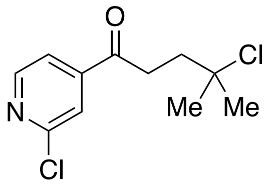

Yellow oil; TOF = 0.27 h^{-1}; ^{1}H NMR (400 MHz, $CDCl_3$) δ 8.56 (d, *J* = 5.2 Hz, 1H), 7.85–7.65 (m, 2H), 3.28–3.11 (m, 2H), 2.24–2.09 (m, 2H), 1.63 (s, 6H); ^{13}C NMR (100 MHz, $CDCl_3$) δ 197.2, 152.9, 150.9, 145.4, 122.3, 119.9, 69.8, 38.9, 35.3, 32.6; HRMS (ESI) calcd. for $C_{11}H_{14}Cl_2NO$ (M^+ + H): 246.0452, found: 246.0446; IR (neat,

cm^{-1}) 2974, 2928, 1699, 1587, 1543, 1463, 1371, 1295, 1213, 1195, 1127, 1109, 1084, 909, 847, 811, 774, 728.

Methyl 4-Chloro-2-(1,3-dioxoisoindolin-2-yl)-4-methylpentanoate (9)

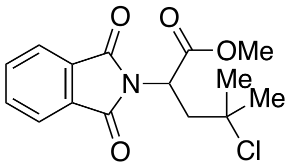

White solid; m.p. 80–82 °C; TOF = 0.21 h^{-1}; ^{1}H NMR (400 MHz, $CDCl_3$) δ 7.80 (dd, *J* = 5.4, 3.1 Hz, 2H), 7.67 (dd, *J* = 5.5, 3.0 Hz, 2H), 5.15 (dd, *J* = 9.5, 3.1 Hz, 1H), 3.66 (s, 3H), 2.80–2.60 (m, 2H), 1.54 (d, *J* = 35.7 Hz, 6H); ^{13}C NMR (100 MHz, $CDCl_3$) δ 169.7, 167.6, 134.2, 131.9, 131.5, 130.4, 123.6, 68.2, 53.1, 49.3, 42.9, 33.2, 32.0; HRMS (ESI) calcd. for $C_{15}H_{17}ClNO_4$ (M^+ + H): 310.0846, found: 310.0843; IR (neat, cm^{-1}) 2975, 1745, 1714, 1387, 1270, 1233, 1172, 1138, 1089, 1023, 910, 720.

Methyl 2-(4-(1-Chloro-2-methylpropyl)phenyl)propanoate (10)

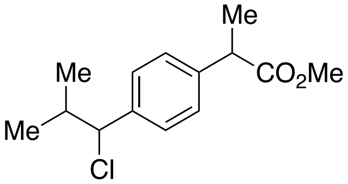

Pale yellow oil; TOF = 0.35 h^{-1}; ^{1}H NMR (400 MHz, $CDCl_3$) δ 7.37–7.21 (m, 4H), 4.65 (d, *J* = 7.6 Hz, 1H), 3.74 (t, *J* = 7.2 Hz, 1H), 3.69 (s, 3H), 2.30–2.20 (m, 1H), 1.52 (d, *J* = 7.2 Hz, 3H), 1.12 (d, *J* = 6.6 Hz, 3H), 0.89 (d, *J* = 6.6 Hz, 3H); ^{13}C NMR (100 MHz, $CDCl_3$) δ 174.8, 140.2, 140.2, 139.9, 127.7, 127.4, 70.5, 52.0, 45.1, 36.6, 20.2, 19.5, 18.6; HRMS (ESI) calcd. for $C_{14}H_{19}O_2$ (M^+ – Cl): 219.1385, found: 219.1390; IR (neat, cm^{-1}) 2965, 2874, 1735, 1151, 1458, 1434, 1332, 1249, 1207, 1162, 1064, 1021, 967, 861, 818, 794, 737.

Methyl 4'-(1-Chlorohexyl)-[1,1'-biphenyl]-4-carboxylate (11)

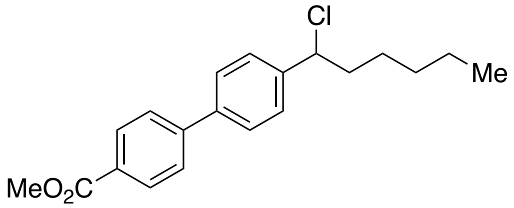

White solid; m.p. 65–66 °C; TOF = 0.32 h^{-1}; ^{1}H NMR (400 MHz, $CDCl_3$) δ 8.18–8.09 (m, 2H), 7.73–7.58 (m, 4H), 7.55–7.46 (m, 2H), 4.93 (dd, J = 8.2, 5.7 Hz, 1H), 4.02–3.91 (m, 3H), 2.27–2.03 (m, 2H), 1.62–1.46 (m, 1H), 1.45–1.25 (m, 5H), 0.92 (q, J = 3.2 Hz, 3H); ^{13}C NMR (100 MHz, $CDCl_3$) δ 166.9, 144.9, 142.0, 139.8, 130.1, 129.1, 127.6, 127.5, 127.0, 63.4, 52.2, 39.9, 31.2, 26.8, 22.5, 14.0; HRMS (ESI) calcd. $C_{20}H_{23}O_2$ (M^+ – Cl): 295.1698, found: 295.1687; IR (neat, cm^{-1}) 2955, 2923, 2857, 1719, 1607, 1436, 1277, 1184, 1111, 1004, 957, 826, 771, 728, 698.

4-(1-Chloroethyl)phenyl (4*R*)-4,7,7-Trimethyl-3-oxo-2-oxabicyclo[2.2.1]heptane-1-carboxylate (12)

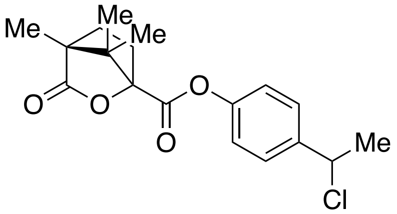

White solid; m.p. 110–111 °C; TOF = 0.40 h^{-1}; ^{1}H NMR (400 MHz, $CDCl_3$) δ 7.81 (d, J = 8.9 Hz, 2H), 7.74 (d, J = 8.5 Hz, 2H), 7.45 (dd, J = 14.5, 8.6 Hz, 4H), 7.05–6.97 (m, 4H), 5.09 (d, J = 6.8 Hz, 1H), 1.89–1.80 (m, 8H); ^{13}C NMR (100 MHz, $CDCl_3$) δ 196.4, 138.2, 132.9, 132.6, 132.3, 131.9, 128.1, 128.0, 126.4, 126.3, 126.3, 126.3, 124.2, 121.5, 69.8, 39.1, 35.0, 32.6; HRMS (ESI) calcd. for $C_{18}H_{21}O_4$ (M^+ – Cl): 301.1440, found: 301.1457; IR (neat, cm^{-1}) 2966, 2930, 2870, 1781, 1758, 1600, 1508, 1443, 1306, 1258, 1231, 1195, 1167, 1124, 1097, 1045, 1015, 990, 958, 935, 876, 810, 725, 654.

4-(1-Chloroethyl)phenyl 2-(4-(4-Chlorobenzoyl)phenoxy)-2-methylpropanoate (13)

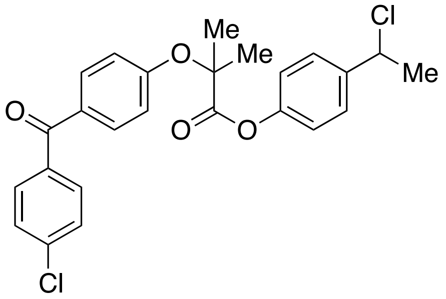

White solid; m.p. 129–130 °C; TOF = 0.40 h^{-1}; ^{1}H NMR (400 MHz, $CDCl_3$) δ 7.48 (dd, *J* = 8.8, 2.8 Hz, 2H), 7.24–7.06 (m, 2H), 5.11 (dq, *J* = 6.9, 3.9 Hz, 1H), 2.67–2.46 (m, 2H), 2.31–2.14 (m, 1H), 2.01 (dd, *J* = 8.9, 5.0 Hz, 1H), 1.86 (dd, *J* = 7.0, 2.7 Hz, 4H), 1.27–1.08 (m, 9H); ^{13}C NMR (100 MHz, $CDCl_3$) δ 194.1, 172.3, 159.5, 150.0, 140.9, 138.5, 136.3, 132.2, 131.2, 130.7, 128.6, 127.8, 121.3, 121.0, 117.3, 79.5, 57.8, 26.6, 25.5; HRMS (ESI) calcd. for $C_{25}H_{22}ClO_4$ (M^+ – Cl): 421.1206, found: 421.1238; IR (neat, cm^{-1}) 2992, 1748, 1650, 1589, 1566, 1504, 1420, 1276, 1251, 1207, 1162, 1119, 1086, 1012, 969, 927, 853, 817, 761, 729, 681.

1-(6-(*tert*-Butyl)-3-chloro-1,1-dimethyl-2,3-dihydro-1*H*-inden-4-yl)ethan-1-one (14)

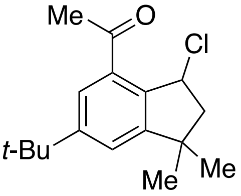

White solid; m.p. 82–83 °C; TOF = 0.17 h^{-1}; ^{1}H NMR (400 MHz, $CDCl_3$) δ 7.65 (t, *J* = 1.7 Hz, 1H), 7.30 (d, *J* = 1.7 Hz, 1H), 5.96 (dt, *J* = 6.6, 1.6 Hz, 1H), 2.57 (d, *J* = 1.6 Hz, 3H), 2.40–2.28 (m, 2H), 1.37 (d, *J* = 1.4 Hz, 3H), 1.28 (dd, *J* = 12.1, 1.5 Hz, 14H); ^{13}C NMR (100 MHz, $CDCl_3$) δ 199.7, 154.1, 153.4, 138.6, 133.6, 129.6, 127.5, 126.0, 123.5, 60.3, 51.0, 43.1, 35.0, 31.4, 31.3, 30.6, 29.3, 28.5; HRMS (ESI) calcd.

for $C_{17}H_{23}O$ ($M^+ - Cl$): 243.1749, found: 243.1762; IR (neat, cm^{-1}) 2959, 2867, 1684, 1600, 1464, 1356, 1298, 1257, 1234, 1162, 1097, 948, 904, 883, 813, 731, 682, 661.

4-Chloro-1-phenylpentan-1-one-2,2-*d*₂ (*d*₂-2s)

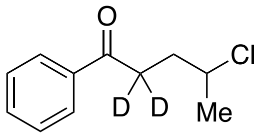

Colourless oil; ^{1}H NMR (400 MHz, $CDCl_3$) δ 7.93–7.88 (m, 2H), 7.53–7.46 (m, 1H), 7.43–7.36 (m, 2H), 4.15–4.04 (m, 1H), 2.24–2.13 (m, 1H), 1.94 (dd, J = 14.7, 9.4 Hz, 1H), 1.51 (d, J = 6.5 Hz, 3H); ^{13}C NMR (100 MHz, $CDCl_3$) δ 199.3, 136.8, 133.2, 128.6, 128.0, 58.4, 34.2, 25.6; HRMS (ESI) calcd. for $C_{11}H_{10}D_2ClO$ (M^+ – H): 197.0702, found: 197.0967; IR (neat, cm^{-1}) 2974, 2928, 1681, 1598, 1581, 1449, 1380, 1316, 1270, 1179, 1208, 885, 746, 728, 690.

1-(4-(Chloromethyl-*d*₂)phenyl)ethan-1-one (*d*₂-6c)

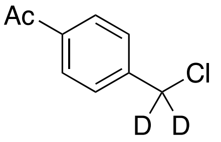

Colourless oil; ^{1}H NMR (400 MHz, $CDCl_3$) δ 7.94–7.82 (m, 2H), 7.46–7.36 (m, 2H), 2.53 (s, 3H); ^{13}C NMR (100 MHz, $CDCl_3$) δ 197.5, 142.3, 137.0, 128.8, 128.7, 26.7; HRMS (ESI) calcd. for $C_9H_8D_2ClO$ (M^+ + H): 171.0545, found: 171.0684; IR (neat, cm^{-1}) 2959, 2927, 2853, 2256, 1775, 1737, 1711, 1638, 1468, 1428, 1391, 1372, 1158, 1113, 1012, 910, 719.

4. ORTEP Drawings.

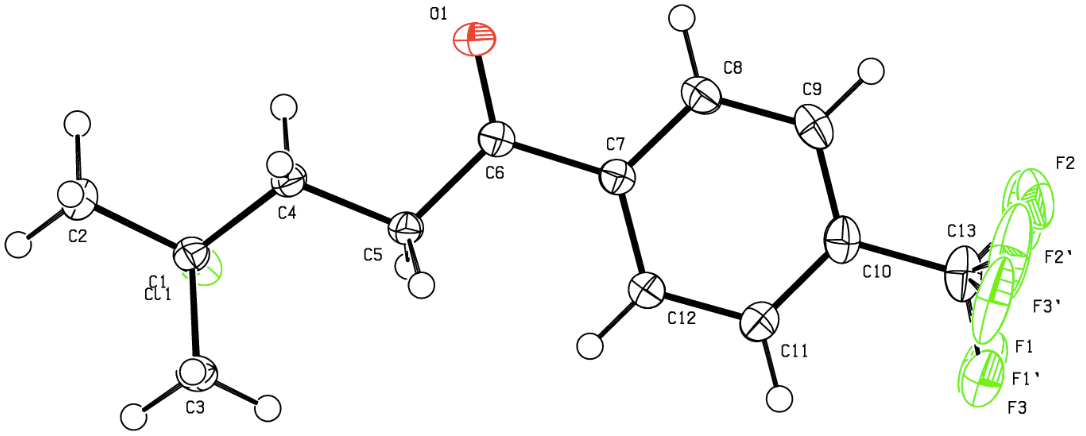

Supplementary Fig. 10 ORTEP drawing of **2d** with thermal ellipsoids at 50% probability levels.[26]

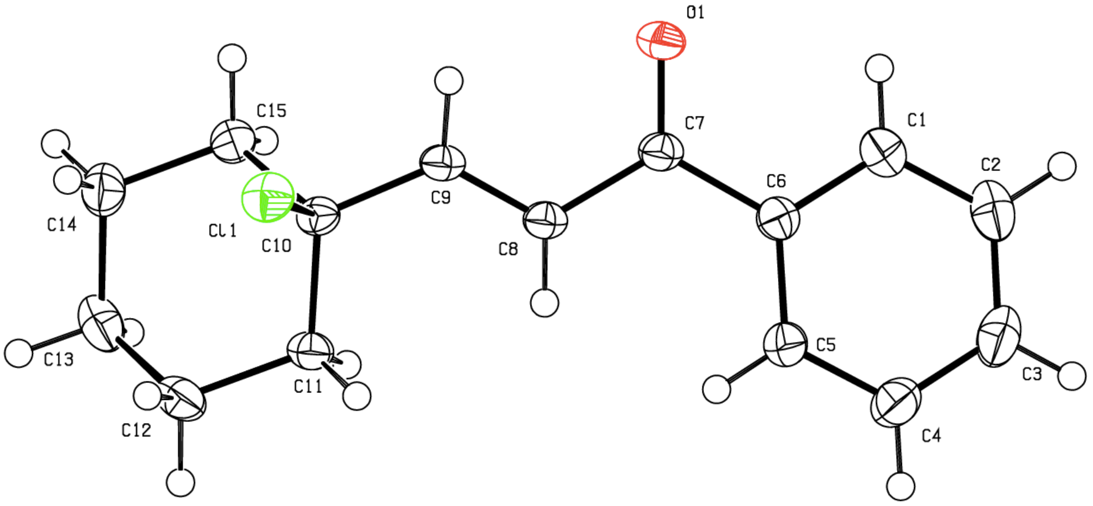

Supplementary Fig. 11 ORTEP drawing of **4k** with thermal ellipsoids at 50% probability levels[27]

5. Computational Details.

Gaussian 16 was used to fully optimise all the structures reported in this paper at the B3LYP level of theory.[28–40] For all the calculations, solvent effects were considered using the SMD solvation model with acetonitrile as the solvent.[41] For geometry optimisations, we used two different combinations of basis sets (designated BS1 and BS2 herein for convienience). For BS1, the SDD basis set with effective core potential (ECP) was chosen to describe copper and the 6-31G(d,p) basis set was employed for all other atoms.[42–44] For BS2, the effective core potential of Hay and Wadt with a double-ξ valence basis set (LANL2DZ) was chosen to describe copper, and a polarisation function was also added for this atom (ξ_f = 3.525).[45–47] The [6-31G(d)] basis set was used for all other atoms.[44] We also employed the D3 empirical dispersion correction for all calculations at the B3LYP level of theory.[48] Therefore, BS1 = SMD/B3LYP-D3/6-31G(d,p),SDD and BS2 = SMD/B3LYP-D3/6-31G(d),LANL2DZ(f). Frequency calculations were carried out at the same level of theory as those for the structural optimisation. Transition state structures were located using the Berny algorithm. Intrinsic reaction coordinate (IRC) calculations were used to confirm the connectivity between transition structures and minima.[49,50] To further refine the energies obtained from the BS1 and BS2 calculations, we carried out single-point energy calculations using the B3LYP-D3 functional method with SMD solvation model in acetonitrile along with a larger basis set on all atoms, namely the def2-TZVP basis set.[51] This basis set considers the f polarisation function on the copper atom (ξ_f = 2.233).[52] Therefore, BS3 = SMD/B3LYP-D3/def2-TZVP. Tight convergence criterion and ultrafine integral grid were exploited to increase the accuracy of the single point calculations. The results obtained at the BS3//B1 level of theory are reported in the main text, and shown in Figs. 4 and 5, and Supplementary

Figs. 12–15, and Supplementary Table 1 in Supplementary Data 1 and those obtained at the BS3//B2 are provided in Supplementary Figs. 16–23 and Supplementary Table 2 in Supplementary Data 1 for comparison.

Minimum energy crossing points (MECPs) between singlet and triplet intermediates were located using the code of Harvey *et al.*[53] In the main text for the structures optimised from the BS1 calculations, the free energy for each species in solution was calculated using the following formula:

$$G = E(\mathrm{BS3}) + G(\mathrm{BS1}) - E(\mathrm{BS1}) + \Delta G^{1\ \mathrm{atm}\rightarrow 1\ \mathrm{M}} \quad (1)$$

where $\Delta G^{1\mathrm{atm}\rightarrow 1\mathrm{M}} = 1.89$ kcal/mol is the free-energy change for compression of 1 mol of an ideal gas from 1 atm to the 1 M solution phase standard state.

An additional correction to Gibbs free energies was made to consider solvent (acetonitrile) concentration where an MeCN molecule is directly involved in transformations. In such a case, the free energy of MeCN is described as follows:

$$G(\mathrm{MeCN}) = E(\mathrm{BS3}) + G(\mathrm{BS1}) - E(\mathrm{BS1}) + \Delta G^{1\ \mathrm{atm}\rightarrow 1\ \mathrm{M}} + RT\ln(19.15) \quad (2)$$

where the last term corresponds to the free energy required to change the standard state of MeCN from 19.15 to 1 M.[54] These numerical correction values for an MeCN molecule is 1.75 kcal/mol.

For the structures optimised from the BS2 calculations, the free energy for each species in solution was calculated using the following formulae:

$$G = E(\mathrm{BS3}) + G(\mathrm{BS2}) - E(\mathrm{BS2}) + \Delta G^{1\ \mathrm{atm}\rightarrow 1\ \mathrm{M}} \quad (3)$$

$$G(\mathrm{MeCN}) = E(\mathrm{BS3}) + G(\mathrm{BS2}) - E(\mathrm{BS2}) + \Delta G^{1\ \mathrm{atm}\rightarrow 1\ \mathrm{M}} + RT\ln(19.15) \quad (4)$$

Spin density distributions were obtained by Mulliken population analysis using the BS3//BS1 level of theory in acetonitrile.

$Cu(MeCN)_2^+$	$CuOTf(MeCN)_2$	CuOTf(MeCN)
**II**	**III**	**IV**
0.0 (0.0)	4.6 (-4.6)	6.2 (4.1)
$Cu(MeCN)_4^+$	$CuOTf(MeCN)_3$	$Cu(NCl_2Ts)(MeCN)^+$
**V**	**VI**	**VII**
1.1 (-7.6)	4.5 (-7.9)	10.9 (10.1)
$Cu(OTf)_2^-$	$Cu(ClNClTs)(MeCN)^+$	$Cu(NCl_2Ts)(OTf)$
**VIII**	**IX**	**X**
14.5 (10.5)	19.5 (17.6)	21.1 (14.2)

Supplementary Fig. 12 Free energies (potential energies) of different copper(I) complexes relative to the most stable species $[Cu(MeCN)_2]^+$ **II**. The relative Gibbs and potential energies (in parentheses) obtained from BS3//BS1 calculations are given in kcal/mol (red).

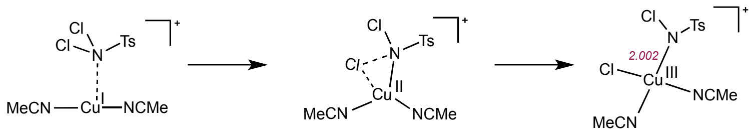

XI 1.5 (-7.3) | $\mathbf{TS^S_{XII\text{-}XIII}}$ 27.9 (18.7) | $\mathbf{XIII^S}$ 20.6 (9.7)

Supplementary Fig. 13 Calculated mechanism for the oxidative addition of dichloramine-T to $[Cu(MeCN)_2]^+$ **II** *via* a concerted mechanism. The relative Gibbs and potential energies (in parentheses) obtained from BS3//BS1 calculations are given in kcal/mol (red) and selected bond length in Å (purple).

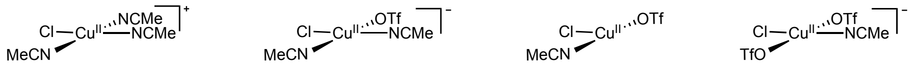

$\mathbf{I^i}$ 0.5 (-4.3) | $\mathbf{I^{ii}}$ 6.6 (-4.5) | $\mathbf{I^{iii}}$ 7.4 (9.4) | $\mathbf{I^{iv}}$ 10.6 (9.6)

Supplementary Fig. 14 Free energies (potential energies) of different copper(II) complexes relative to the copper(II) species **I**. The relative Gibbs and potential energies (in parentheses) obtained from BS3//BS1 calculations are given in kcal/mol (red).

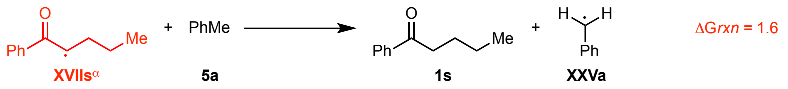

Supplementary Fig. 15 Isodesmic reaction of **XVIIs**$^{\alpha}$ and **5a** to **1s** and **XXVa**. The relative Gibbs free energy of the reaction obtained from BS3//BS1 calculations are given in kcal/mol (red).

$Cu(MeCN)_2^+$ **II** 0.0 (0.0)	$CuOTf(MeCN)_2$ **III** 4.2 (-4.5)	CuOTf(MeCN) **IV** 5.7 (3.9)
$Cu(MeCN)_4^+$ **V** 6.2 (-7.1)	$CuOTf(MeCN)_3$ **VI** 7.1 (-7.6)	$Cu(NCl_2Ts)(MeCN)^+$ **VII** 11.2 (10.9)
$Cu(OTf)_2^-$ **VIII** 14.2 (10.0)	$Cu(ClNClTs)(MeCN)^+$ **IX** 18.1 (17.7)	$Cu(NCl_2Ts)(OTf)$ **X** 18.6 (13.6)

Supplementary Fig. 16 Free energies (potential energies) of different copper(I) complexes relative to the most stable species $[Cu(MeCN)_2]^+$ **II**. The relative Gibbs and potential energies (in parentheses) obtained from BS3//BS2 calculations are given in kcal/mol (red).

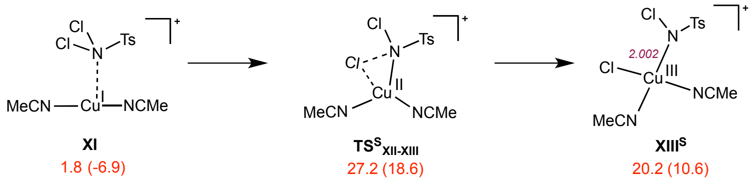

Supplementary Fig. 17 Calculated mechanism for the oxidative addition of dichloramine-T to $[Cu(MeCN)_2]^+$ **II** *via* a concerted mechanism. The relative Gibbs and potential energies (in parentheses) obtained from BS3//BS2 calculations are given in kcal/mol (red) and selected bond length in Å (purple).

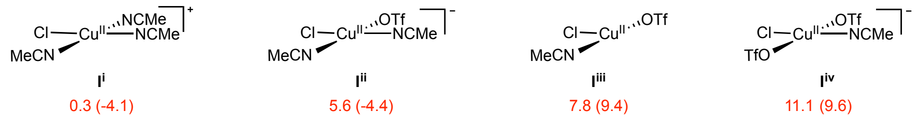

Supplementary Fig. 18 Free energies (potential energies) of different copper(II) complexes relative to the copper(II) species **I**. The relative Gibbs and potential energies (in parentheses) obtained from BS3//BS2 calculations are given in kcal/mol (red).

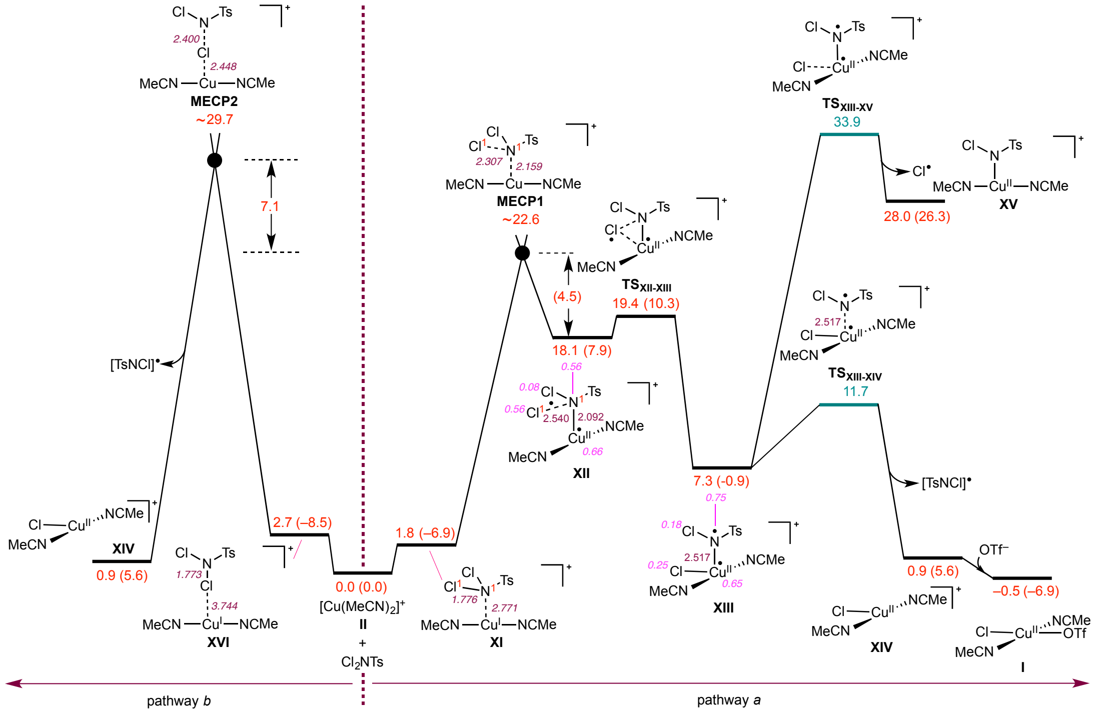

Supplementary Fig 19 Energy profiles for the oxidative addition of dichloramine-T to $[Cu(CH_3CN)_2]^+$ **II** *via* the SET mechanistic pathways *a* and *b*. The relative Gibbs free and potential energies (in parentheses) obtained from BS3//BS2 calculations are given in kcal/mol (red), bond lengths in Å (purple) and spin density distribution values in e/Å^3 (pink).

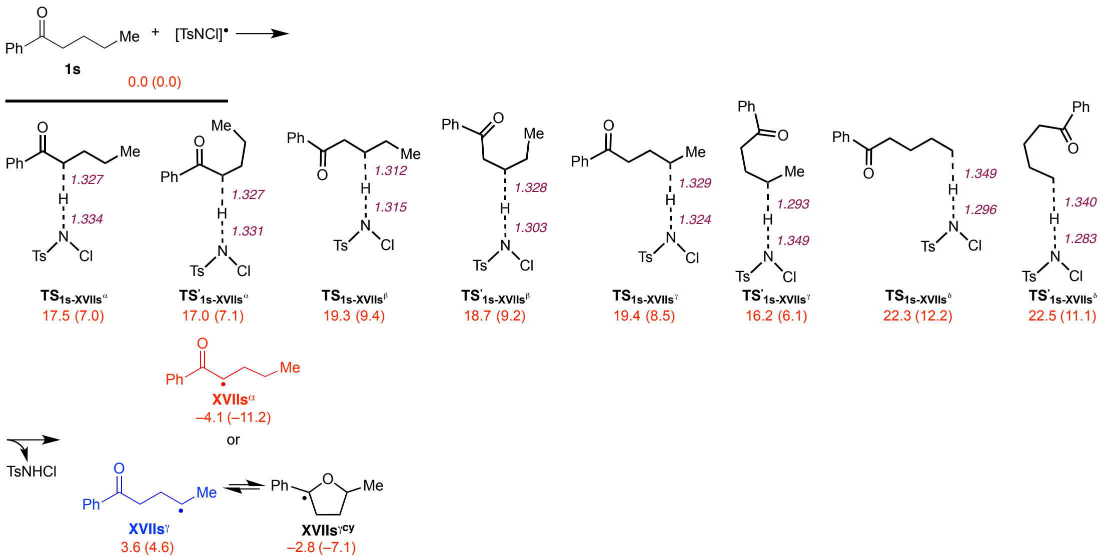

Supplementary Fig 20 Energy barriers for the HAT reaction of [TsNCl]$^{\bullet}$ at different positions of **1s** and relative energies of the ensuing radical species **XVIIs**$^{\alpha}$, and **XVIIs**$^{\gamma}$ and **XVIIs**$^{\gamma cy}$ produced from the two most favoured transition states, **TS′**$_{\mathbf{1s\text{-}XVIIs\alpha}}$ and **TS′**$_{\mathbf{1s\text{-}XVIIs\gamma}}$. The relative Gibbs free and potential energies (in parentheses) obtained from BS3//BS2 calculations are given in kcal/mol (red), bond lengths in Å (purple) and spin density distribution values in e/Å^3 (pink).

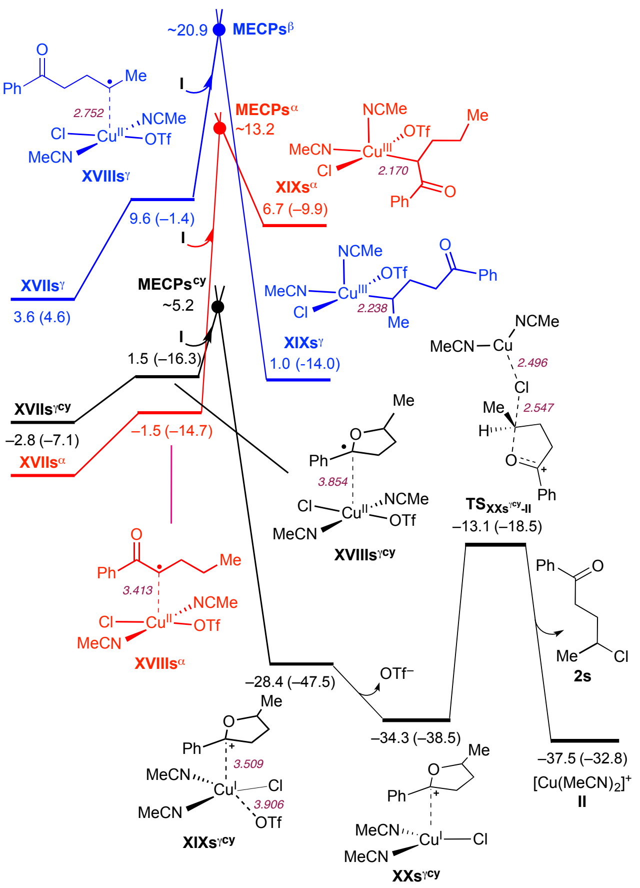

Supplementary Fig 21 Energy profile for the chlorination of **XVIIs**$^{\alpha}$, **XVIIs**$^{\gamma}$ and **XVIIs**$^{\gamma cy}$ to **2s**. The relative Gibbs free and potential energies (in parentheses) obtained from BS3//BS2 calculations are given in kcal/mol (red), bond lengths in Å (purple) and spin density distribution values in e/Å^3 (pink).

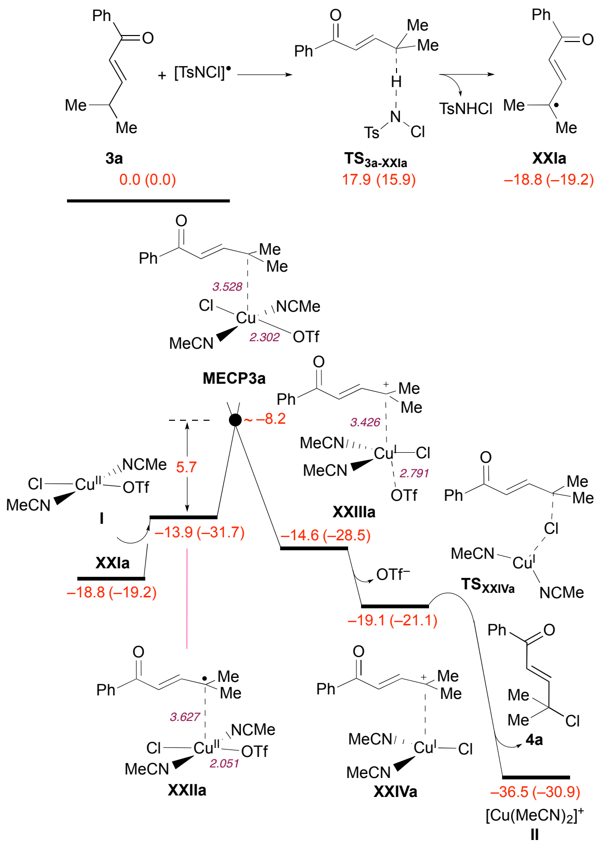

Supplementary Fig 22 Energy profile for the chlorination of **XXIa** to **4a** from **3a** and [TsNCl]• motif. The relative Gibbs free energies and potential energies (in parentheses) obtained from BS3//B2 calculations are given in kcal/mol (red) and bond lengths in Å (purple).

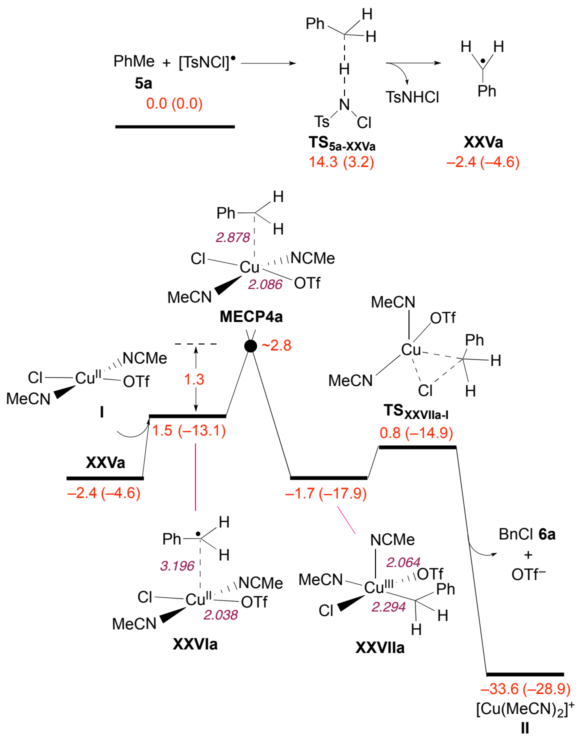

Supplementary Fig 23 Energy profile for the chlorination of **XXVa** to **6a** from **5a** and [TsNCl]• motif. The relative Gibbs free energies and potential energies (in parentheses) obtained from BS3//B2 calculations are given in kcal/mol (red) and bond lengths in Å (purple).

6. ^{1}H and ^{13}C NMR Spectra.

^{1}H and ^{13}C NMR spectra of 1-(4-(tert-butyl)phenyl)-4-methylpentan-1-one (**1b**)

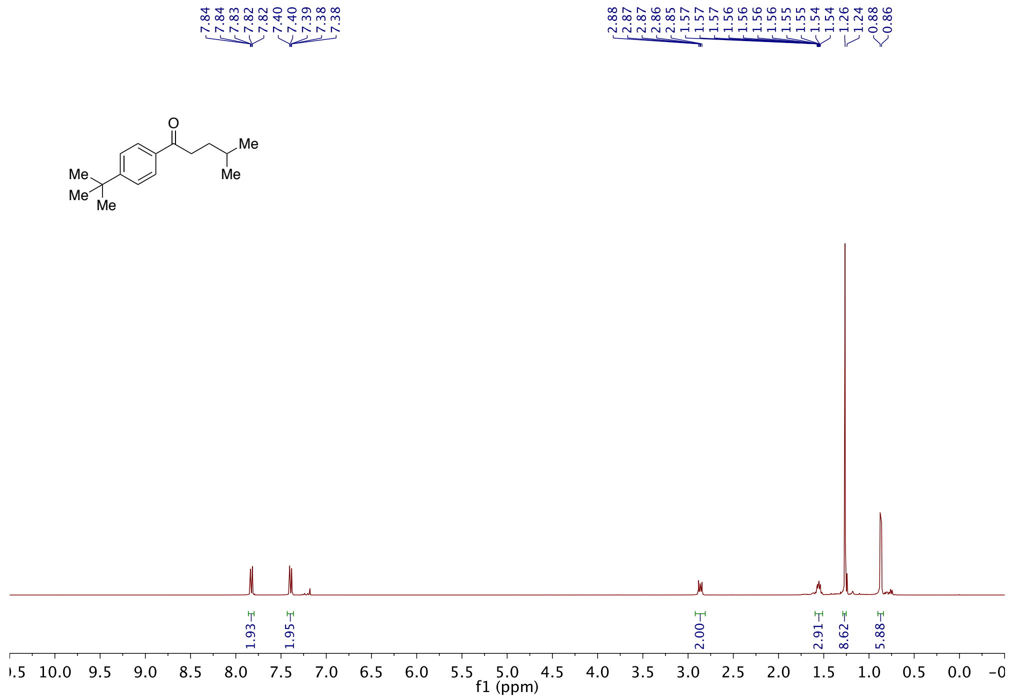

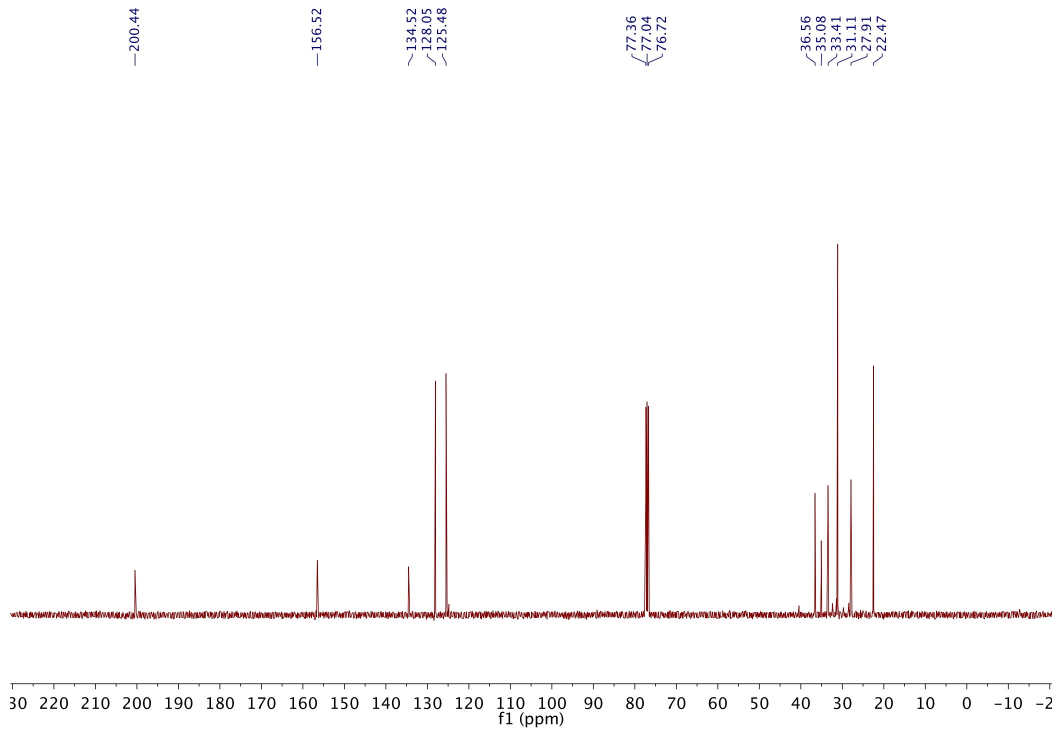

^{1}H and ^{13}C NMR spectra of 1-(4-methoxyphenyl)-4-methylpentan-1-one (**1c**)

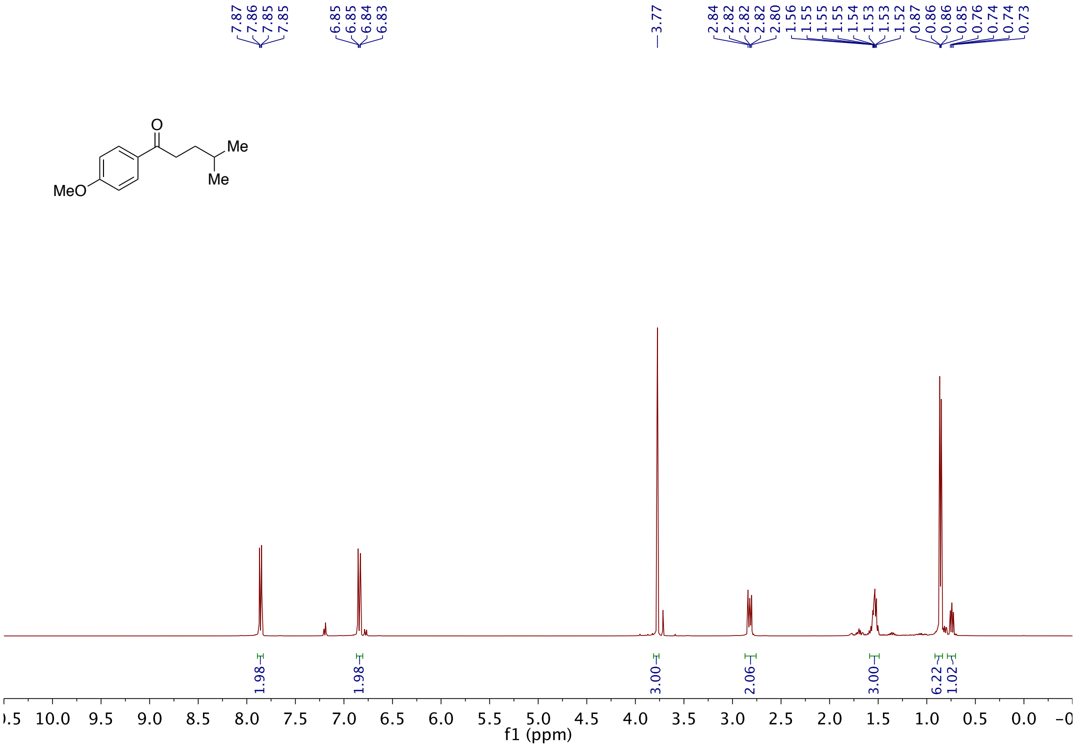

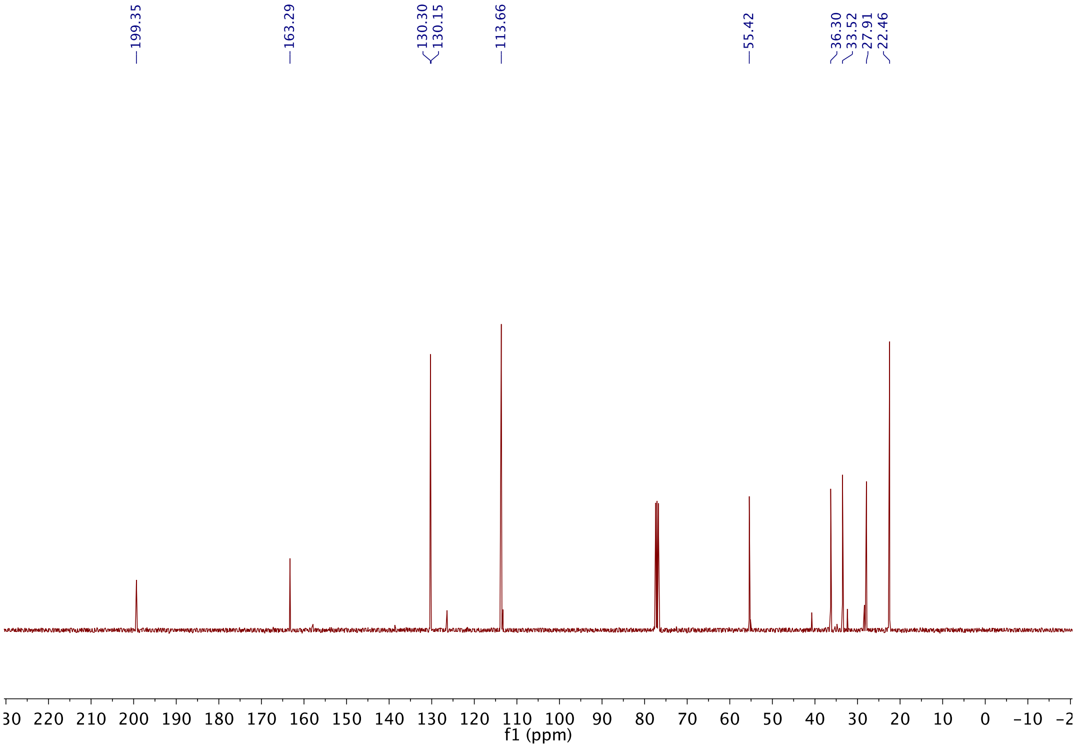

^{1}H and ^{13}C NMR spectra of 4-methyl-1-(4-(trifluoromethyl)phenyl)pentan-1-one (**1d**)

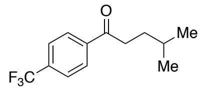

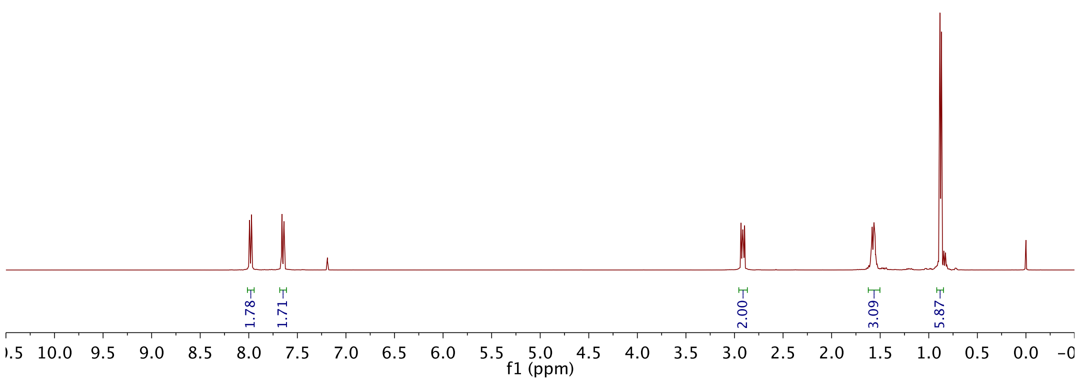

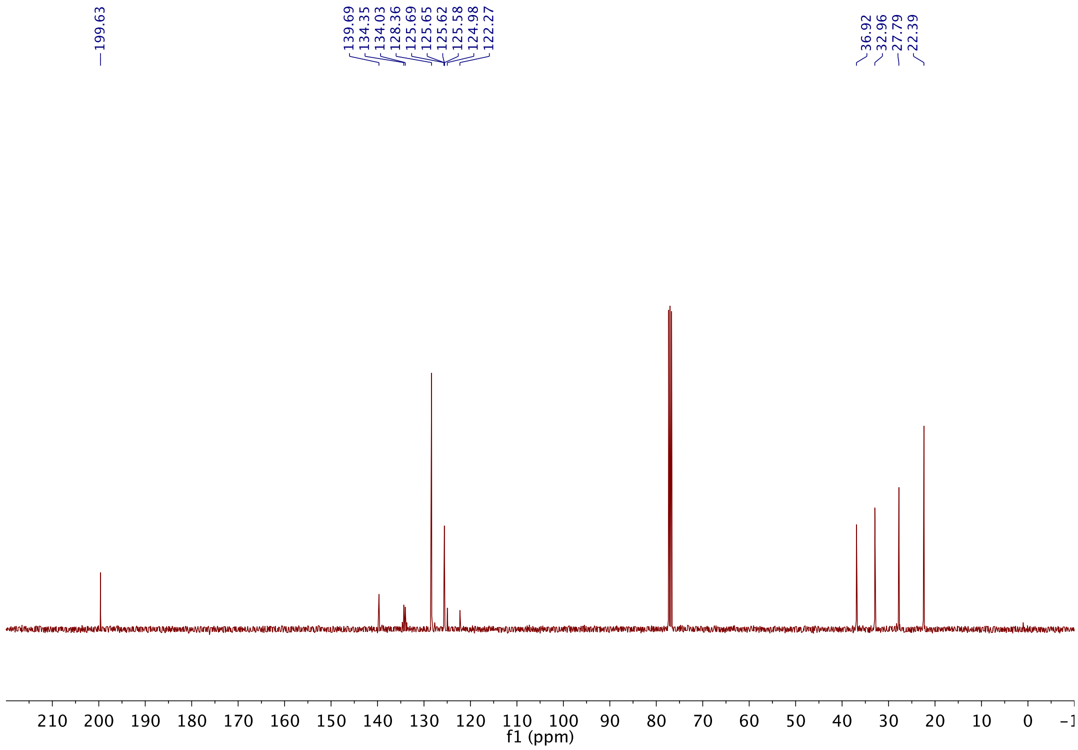

^{1}H and ^{13}C NMR spectra of 1-(4-bromophenyl)-4-methylpentan-1-one (**1e**)

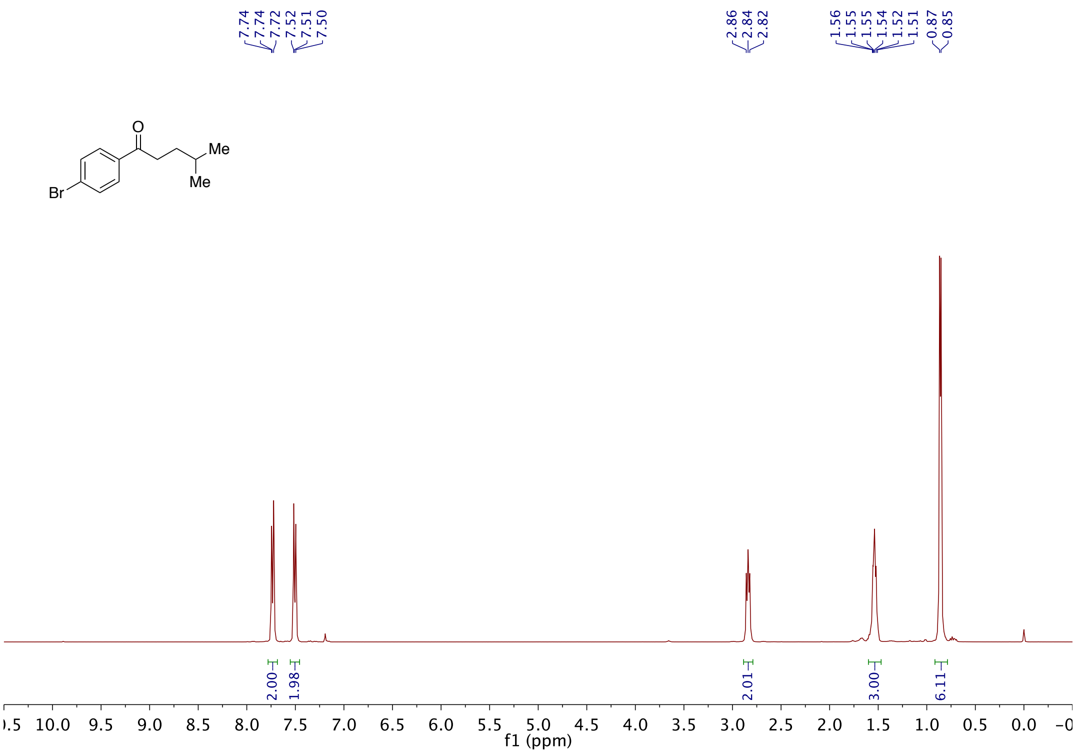

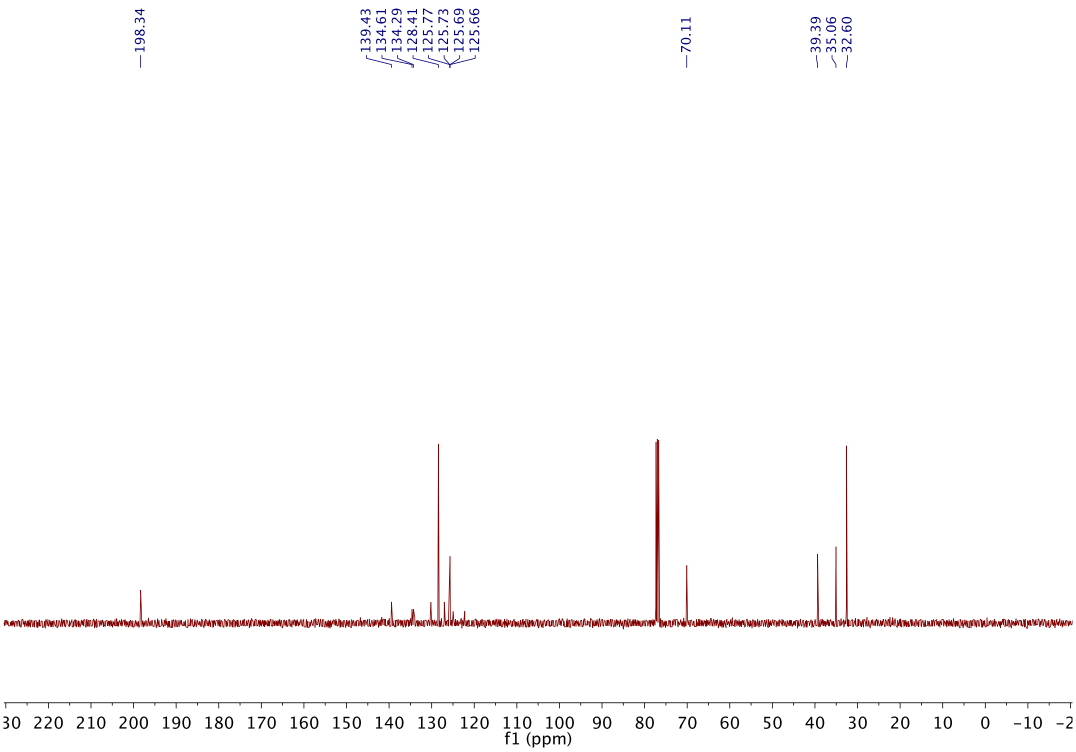

^{1}H and ^{13}C NMR spectra of 1-(3-methoxyphenyl)-4-methylpentan-1-one (**1f**)

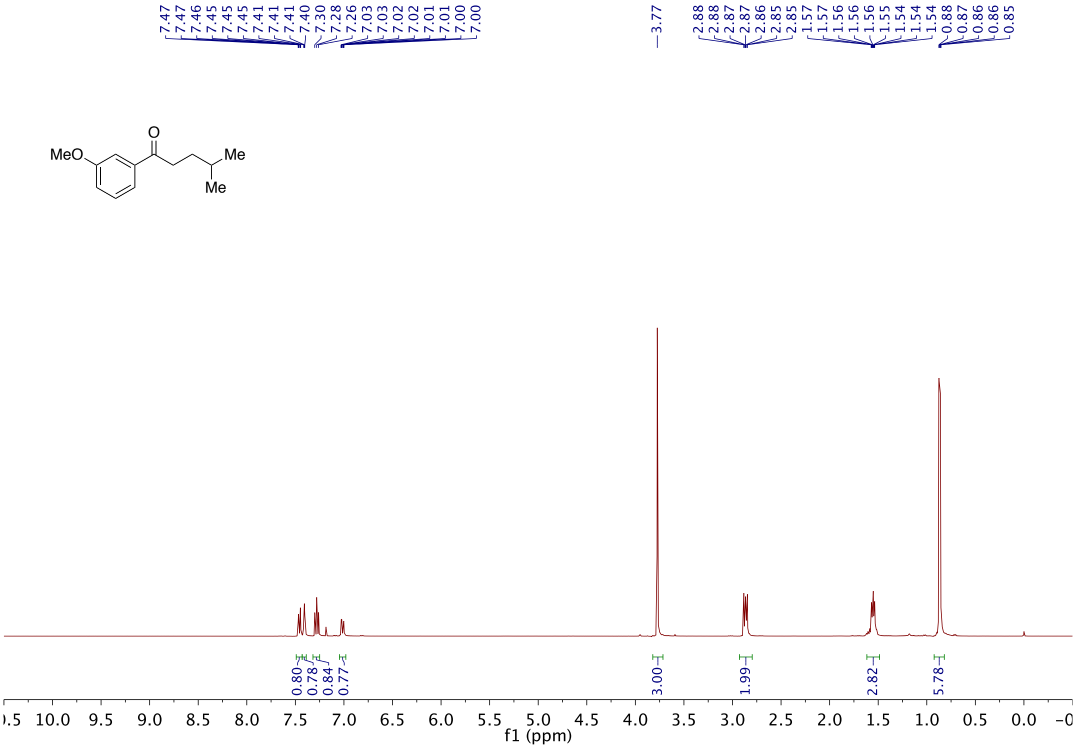

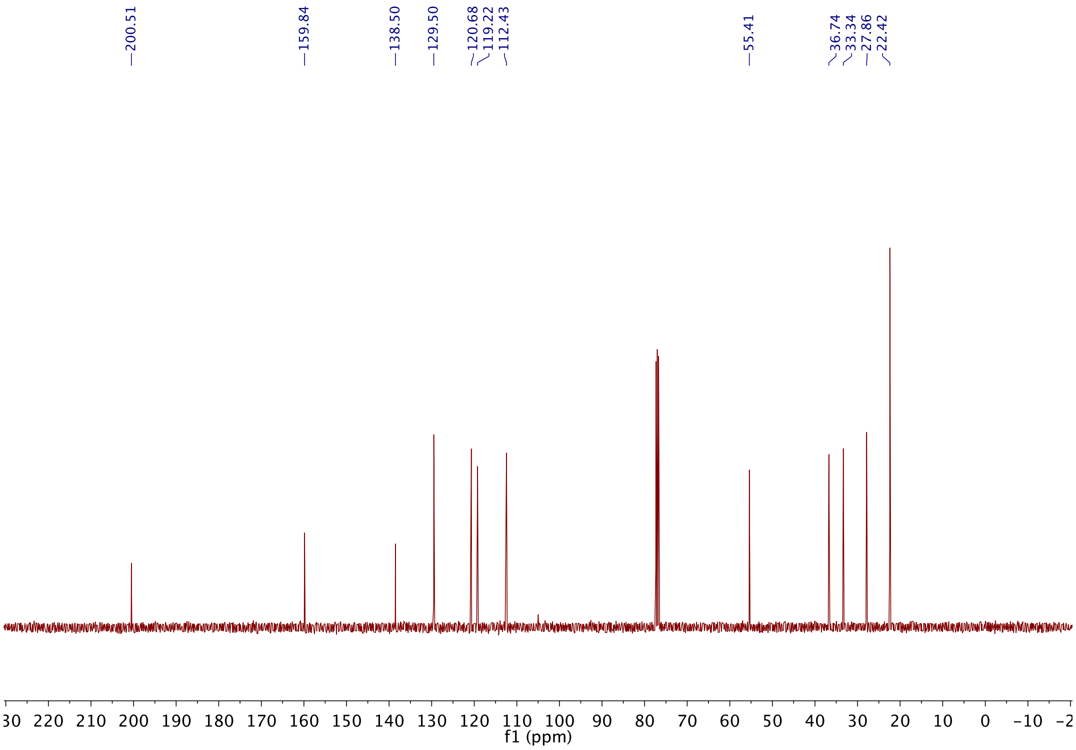

^{1}H and ^{13}C NMR spectra of 4-methyl-1-(*o*-tolyl)pentan-1-one (**1g**)

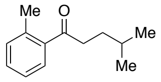

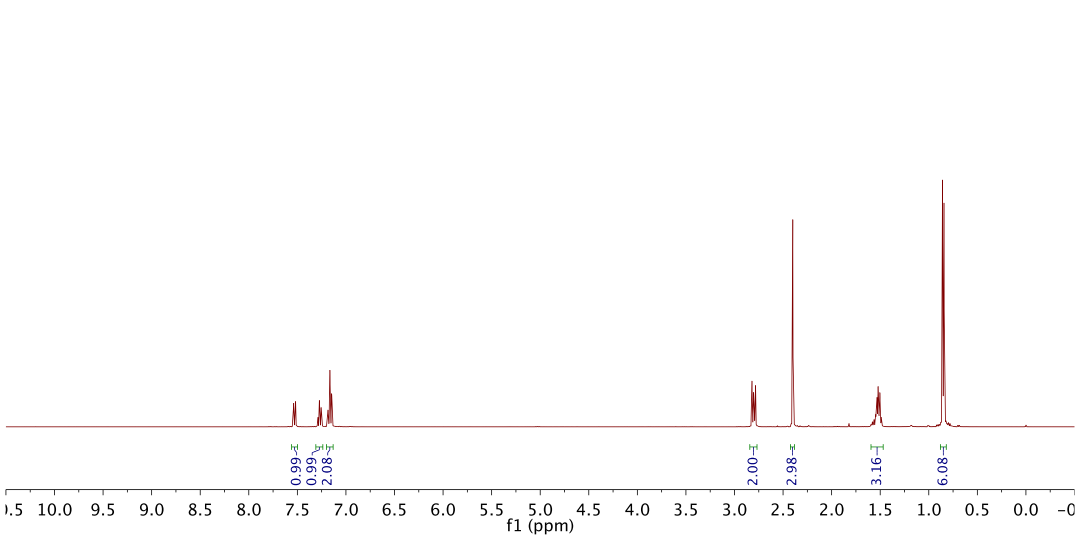

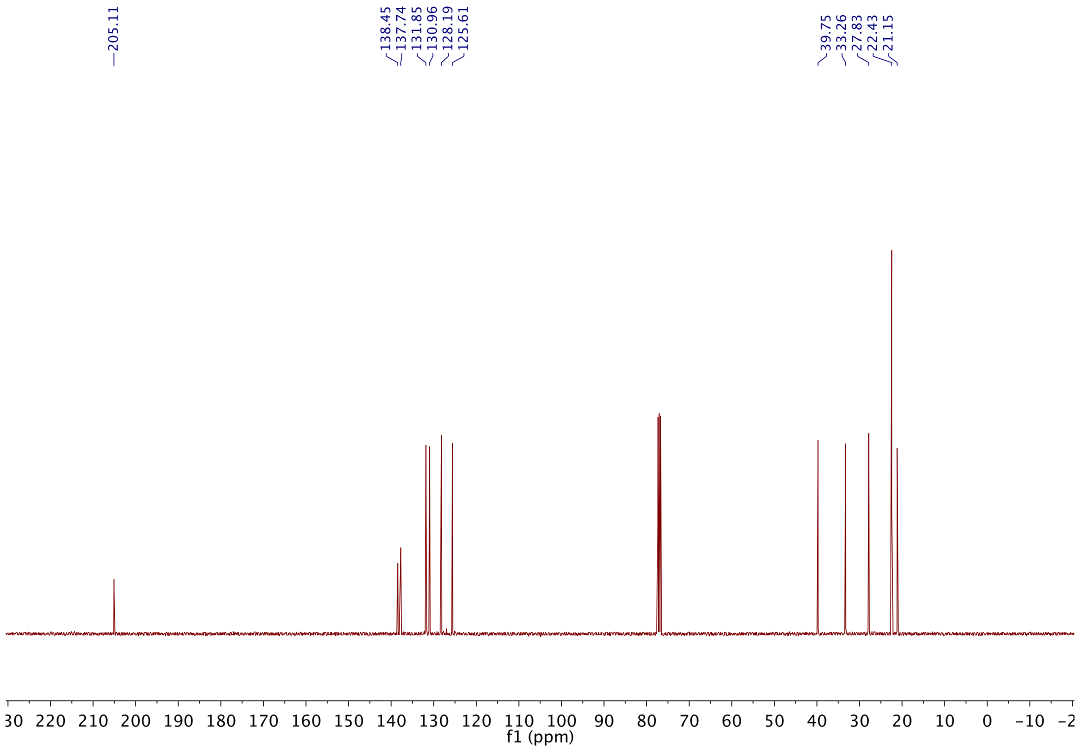

^{1}H and ^{13}C NMR spectra of 1-(3-fluorophenyl)-4-methylpentan-1-one (**1h**)

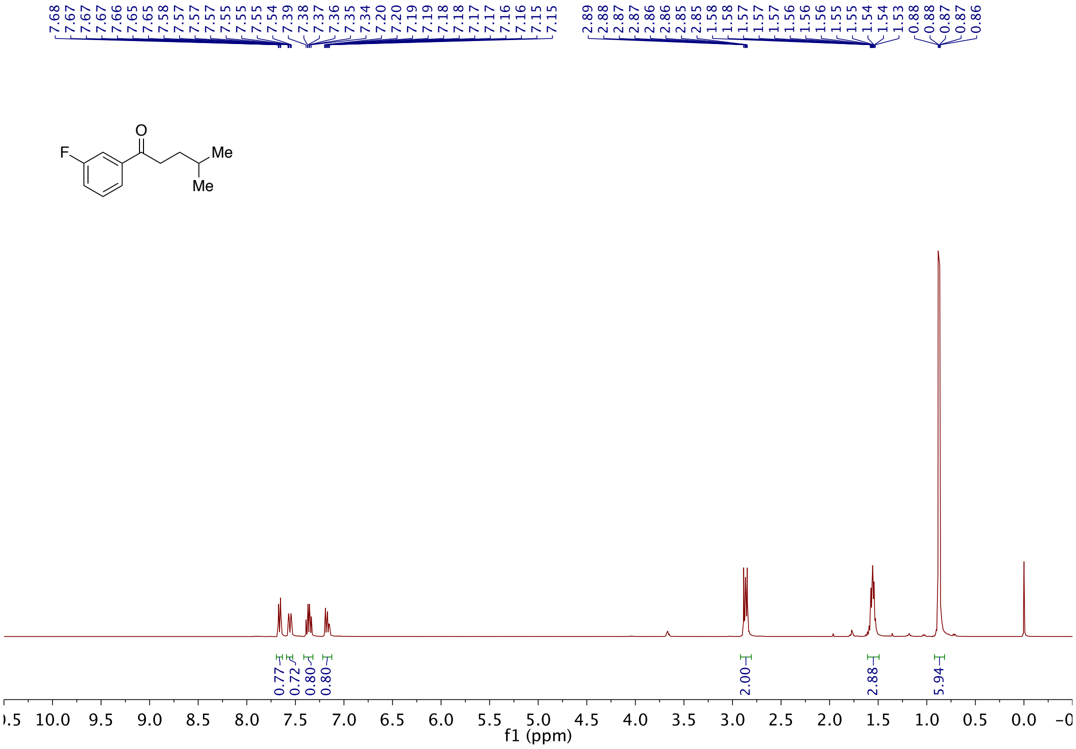

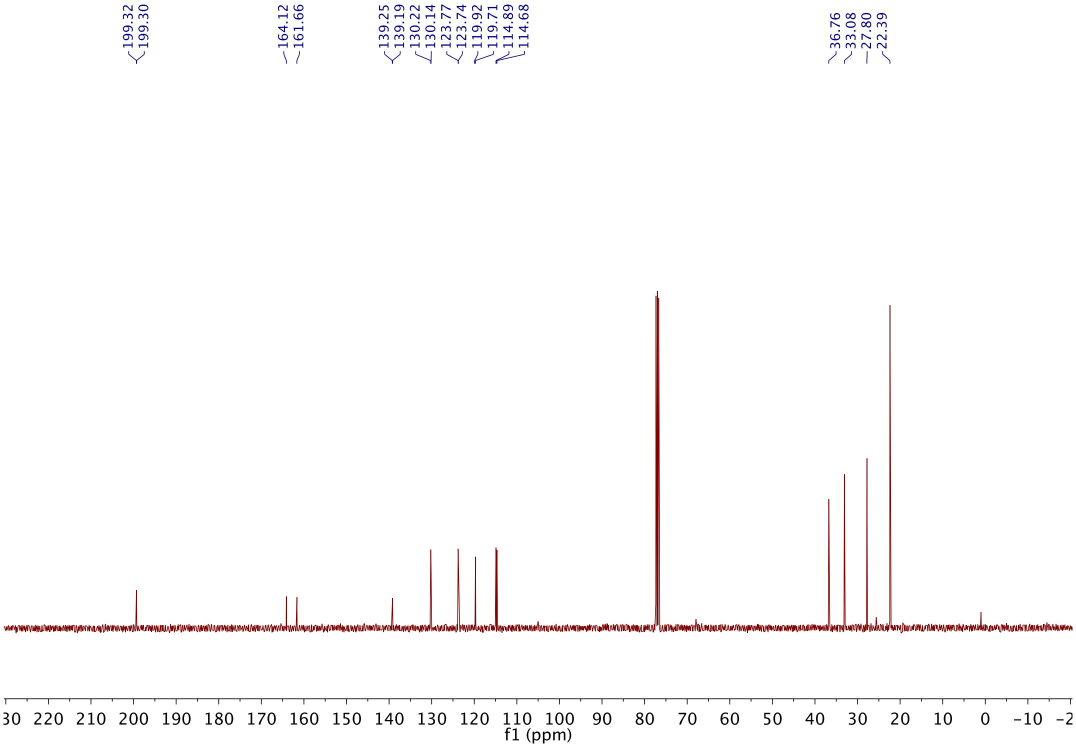

^{1}H and ^{13}C NMR spectra of 1-(3-methoxy-5-(trifluoromethyl)phenyl)-4-methylpentan-1-one (**1i**)

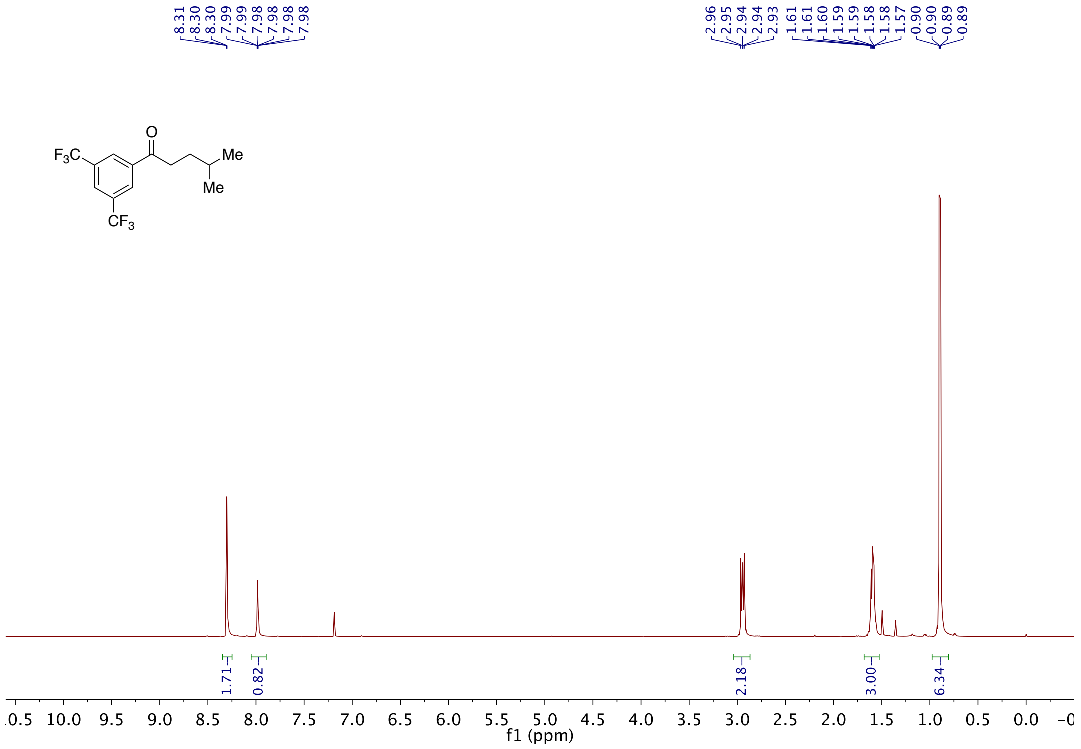

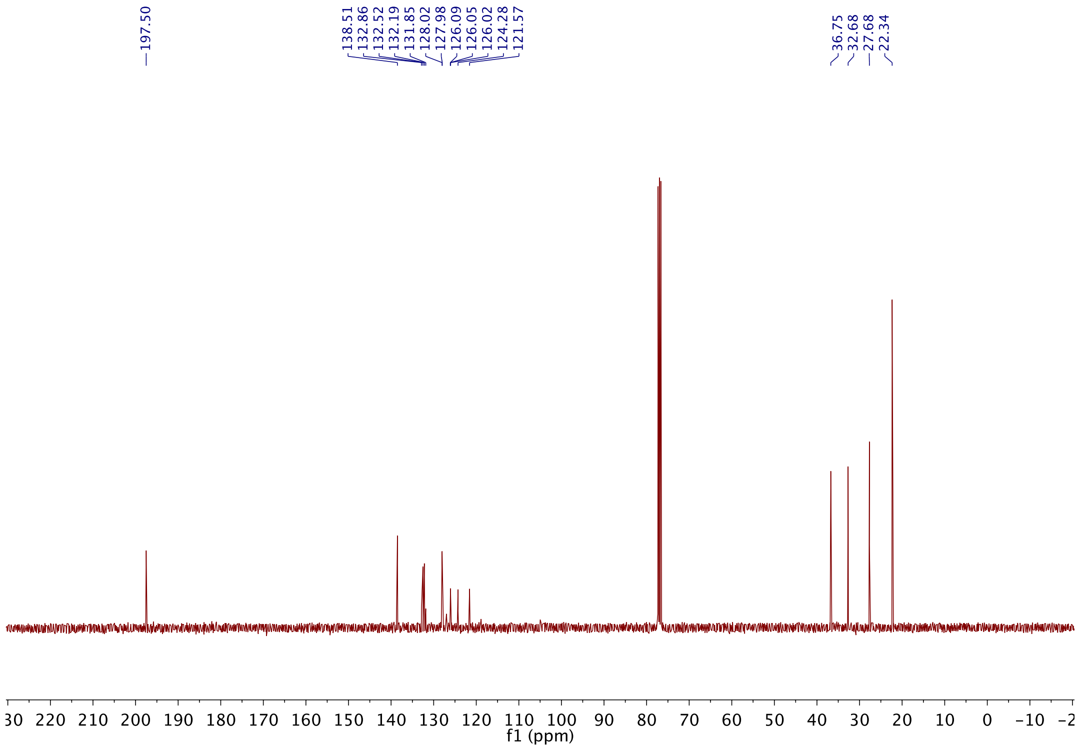

^{1}H and ^{13}C NMR spectra of 4-methyl-1-(phenanthren-9-yl)pentan-1-one (**1j**)

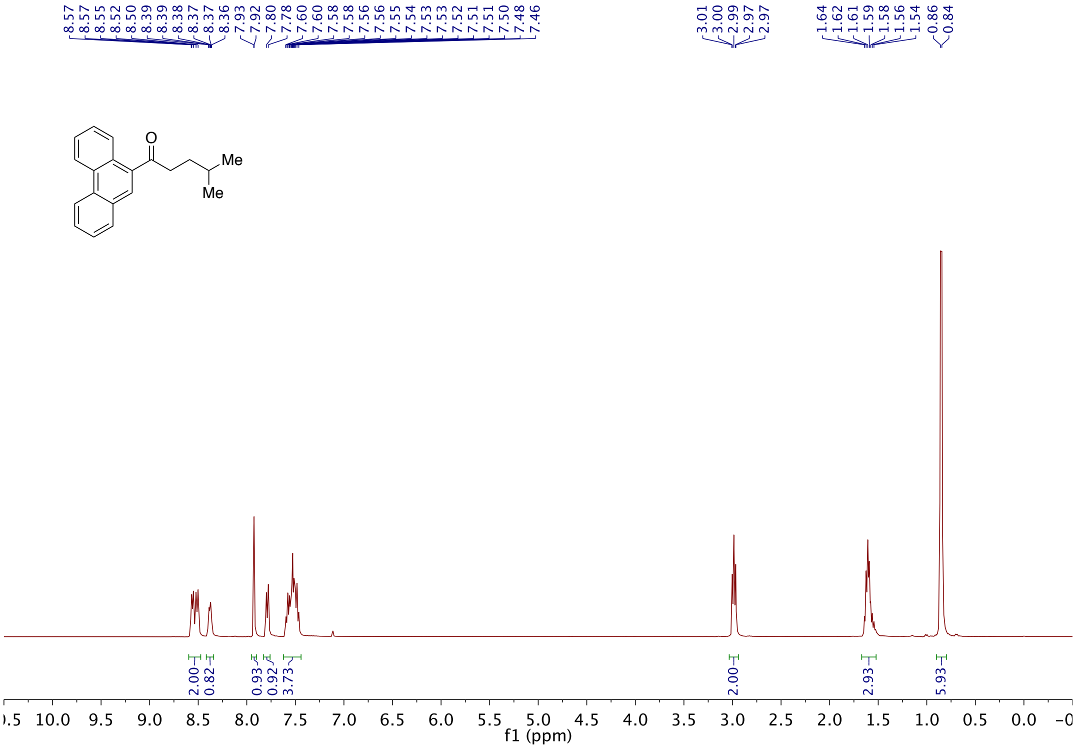

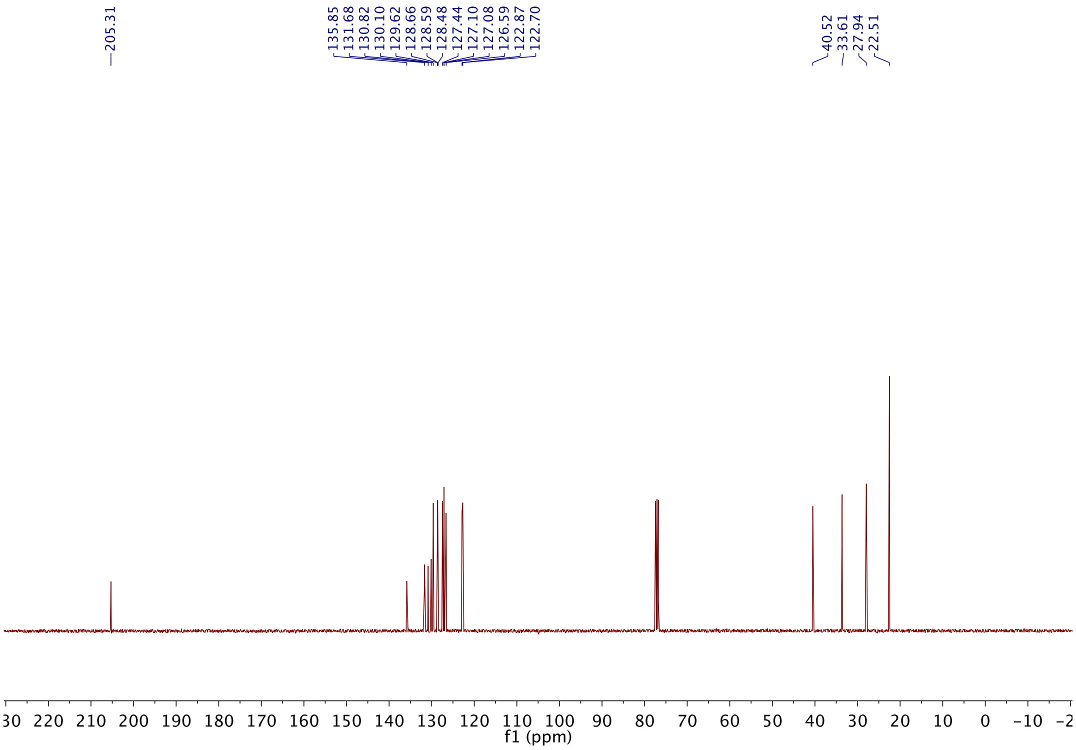

^{1}H and ^{13}C NMR spectra of 4-methyl-1-phenyloctan-1-one (**1k**)

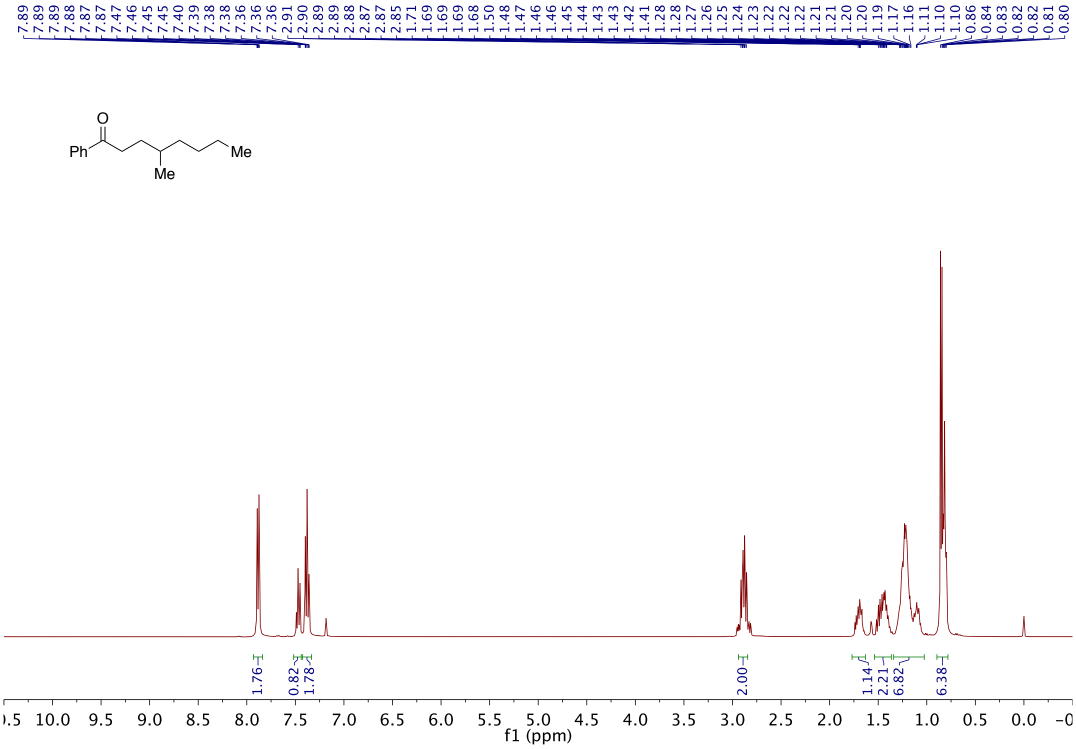

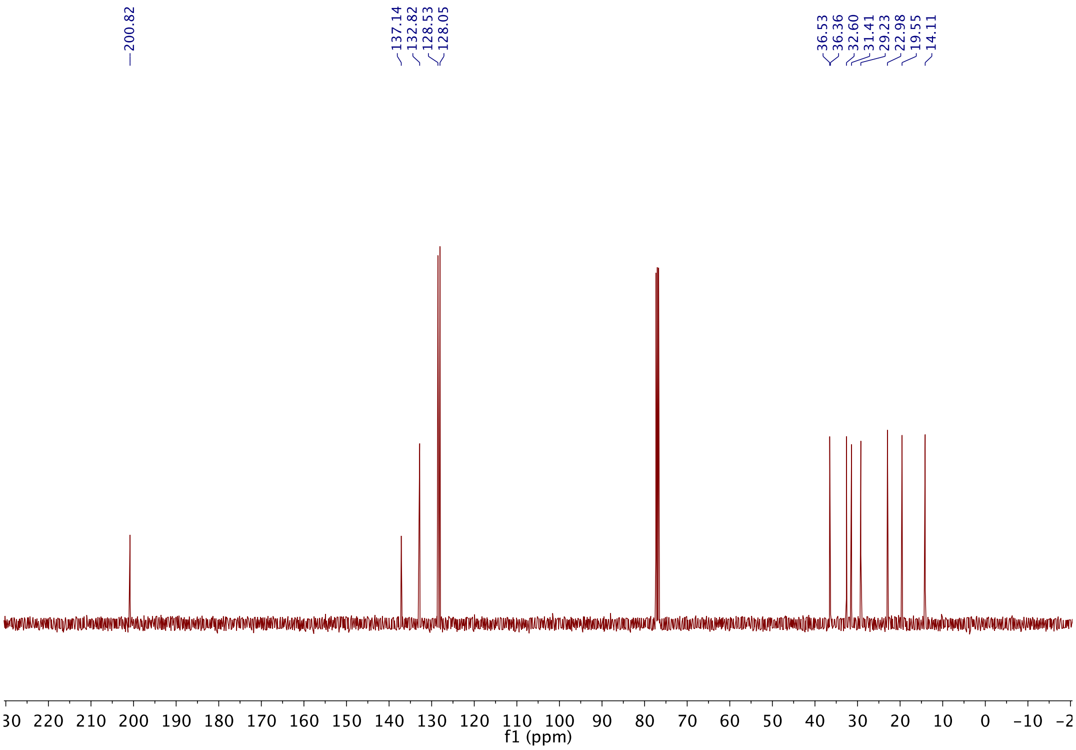

^{1}H and ^{13}C NMR spectra of 4-ethyl-1-phenyloctan-1-one (**1l**)

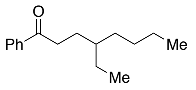

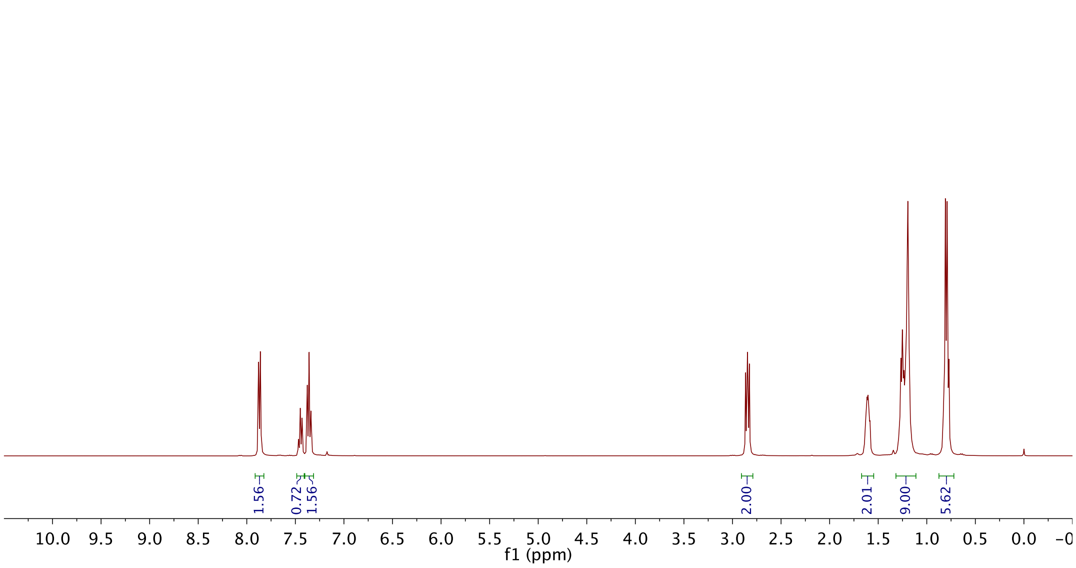

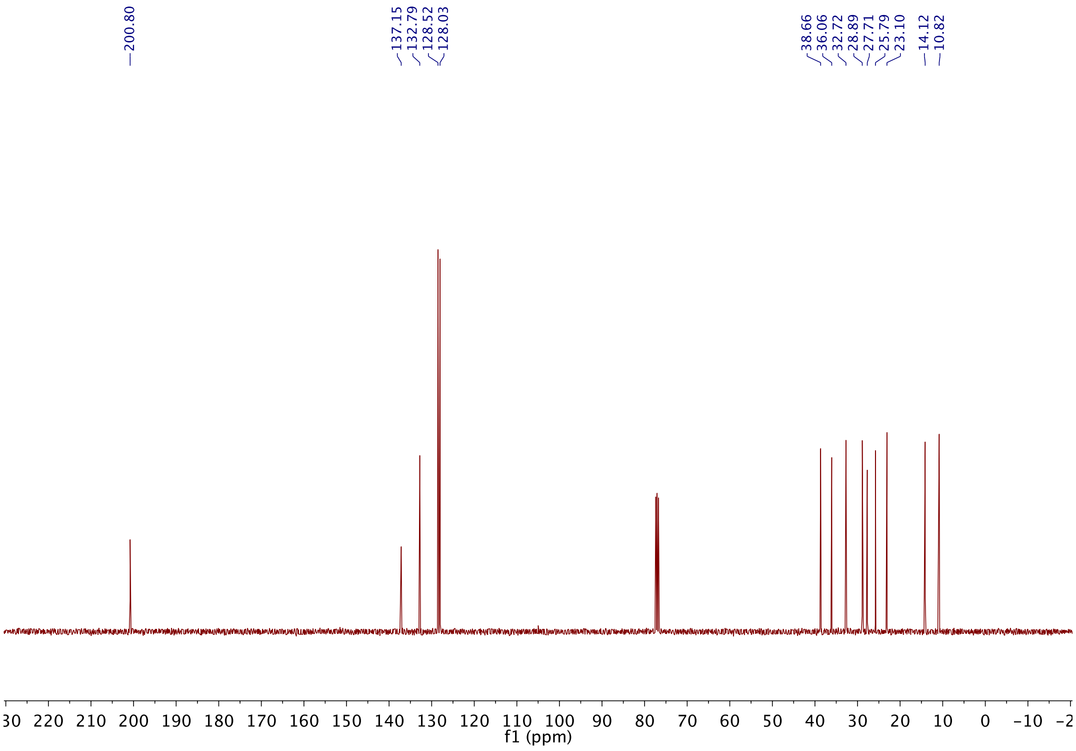

^{1}H and ^{13}C NMR spectra of 2,4-dimethyl-1-phenylpentan-1-one (**1m**)

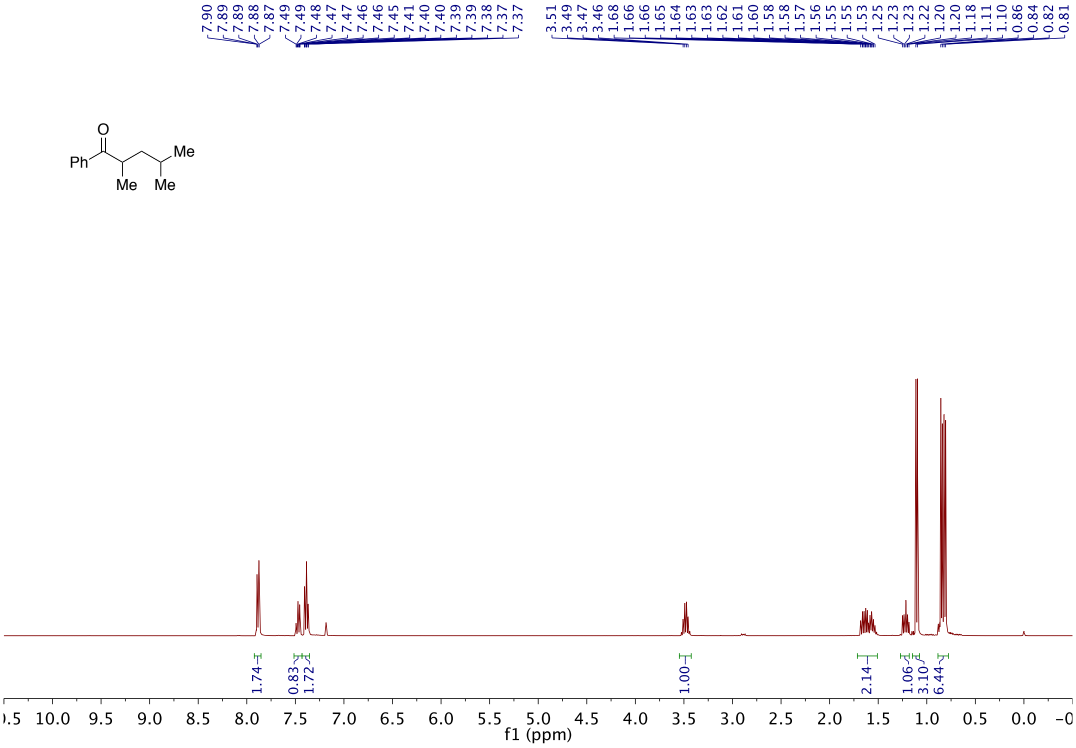

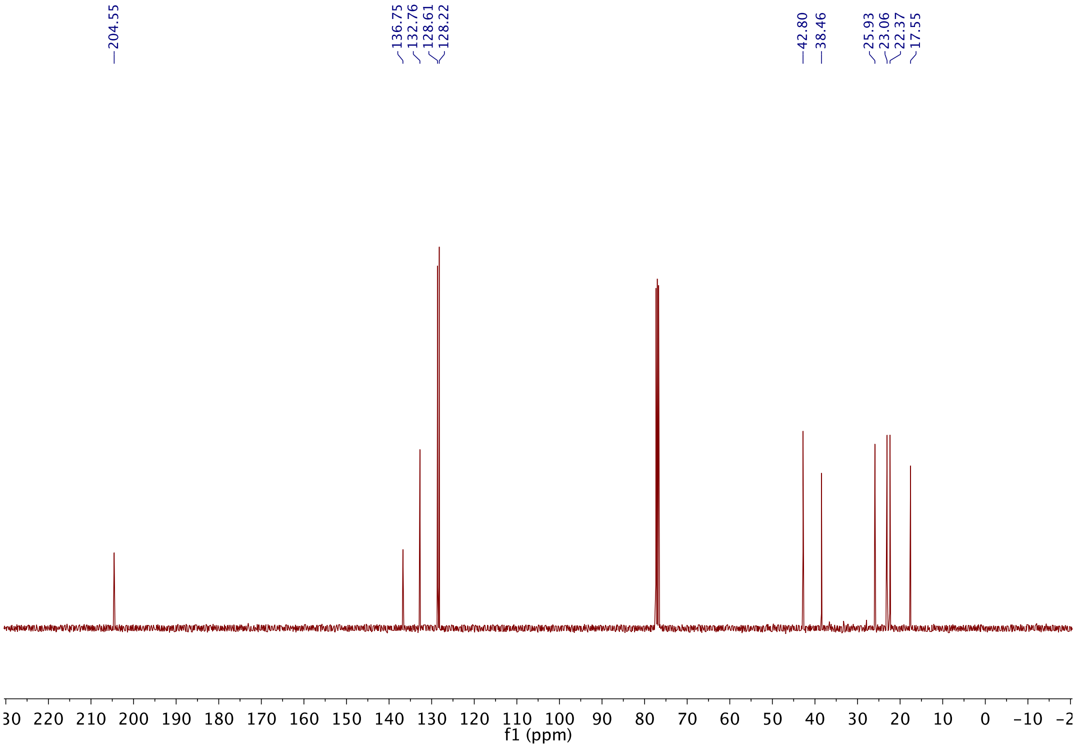

^{1}H and ^{13}C NMR spectra of 3-cyclopentyl-1-phenylpropan-1-one (**1n**)

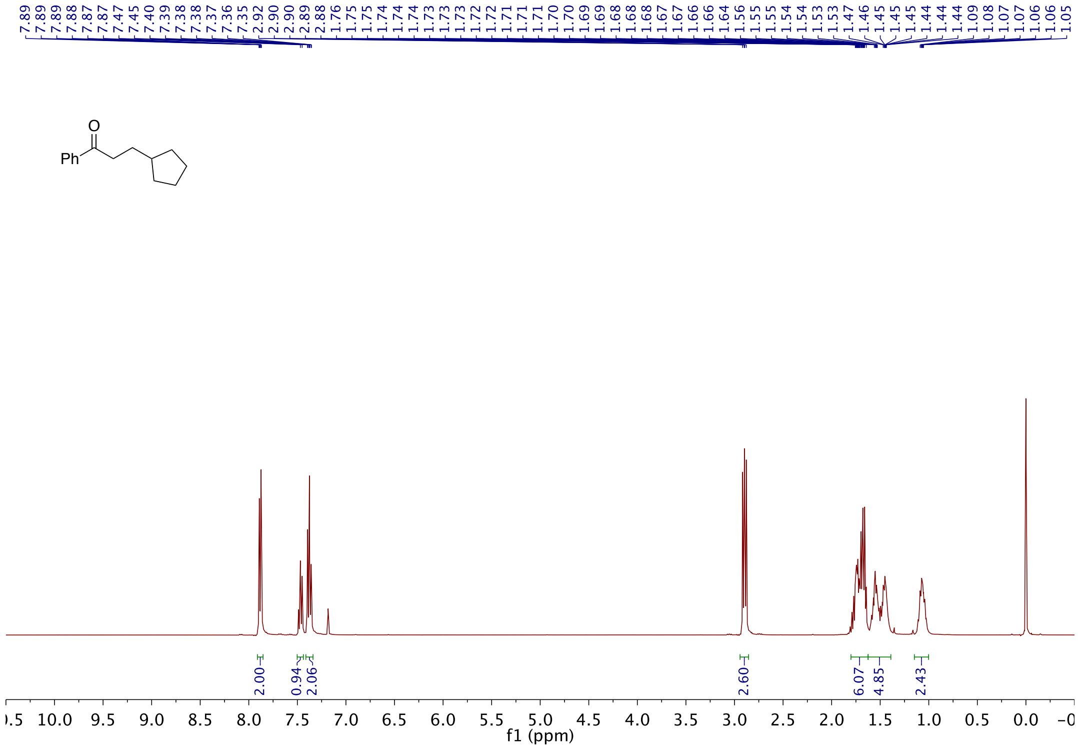

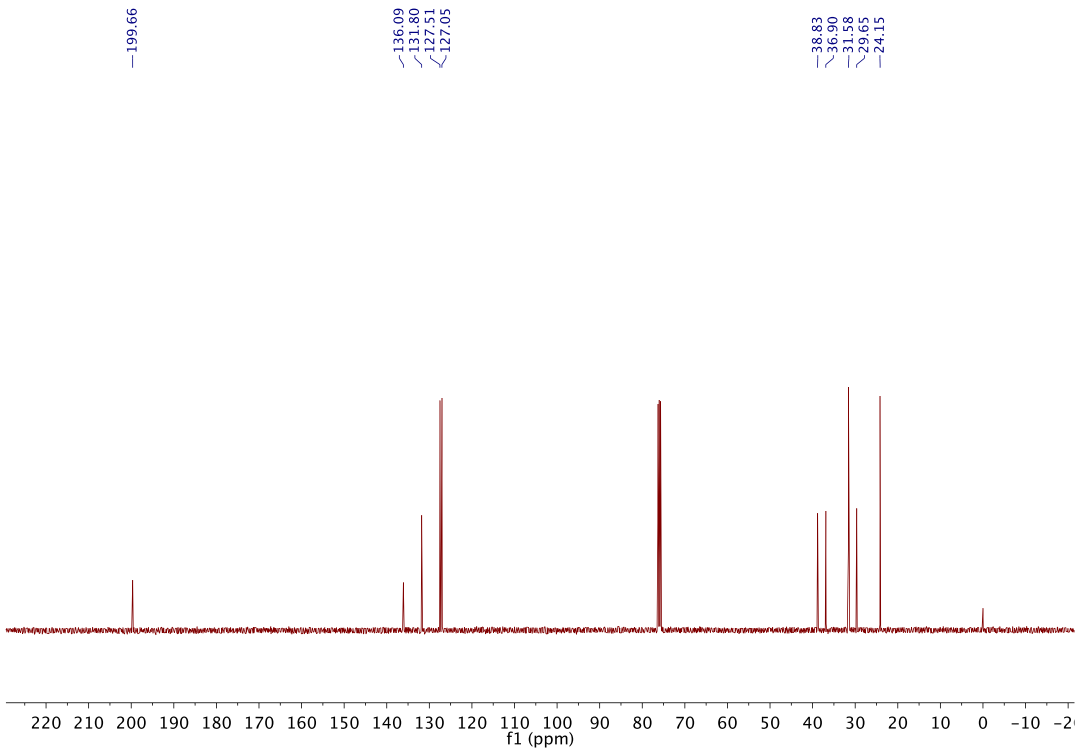

^{1}H and ^{13}C NMR spectra of 3-cyclohexyl-1-phenylpropan-1-one (**1o**)

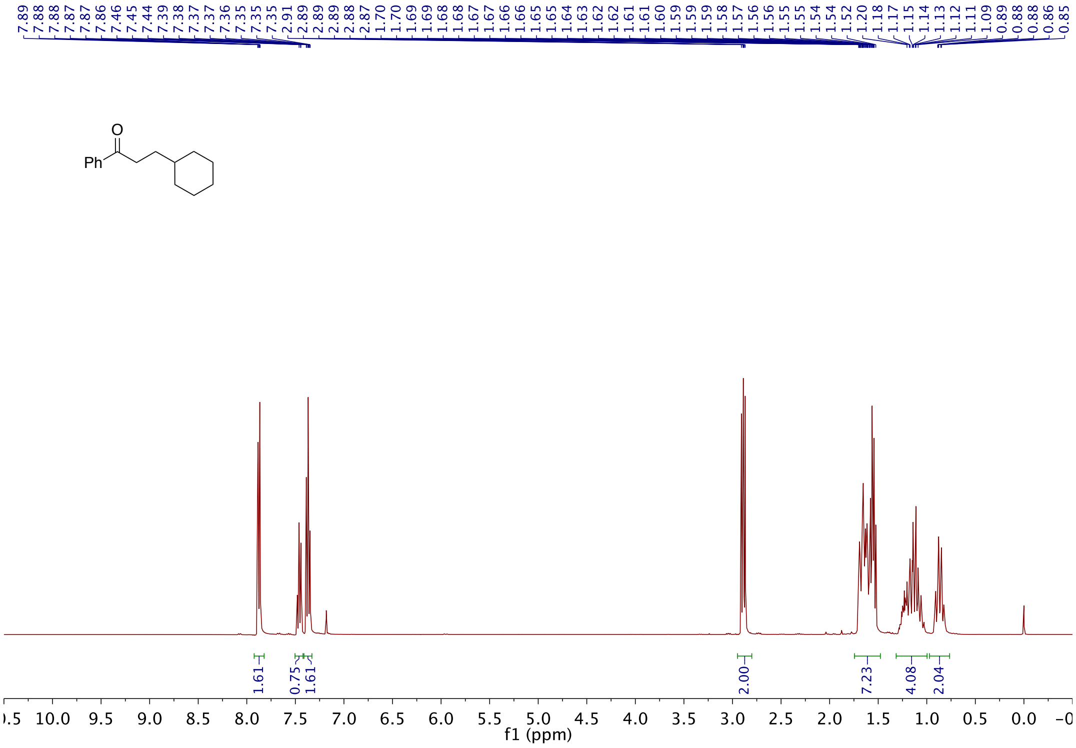

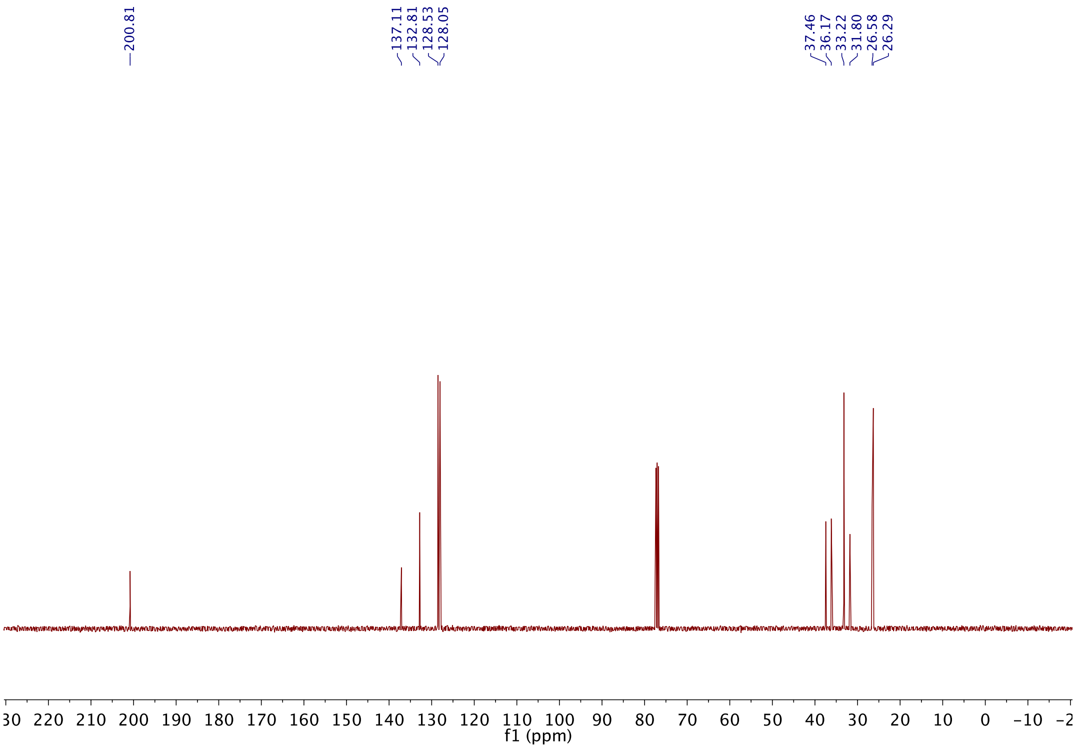

^{1}H and ^{13}C NMR spectra of 6-methyl-2-phenylheptan-3-one (**1p**)

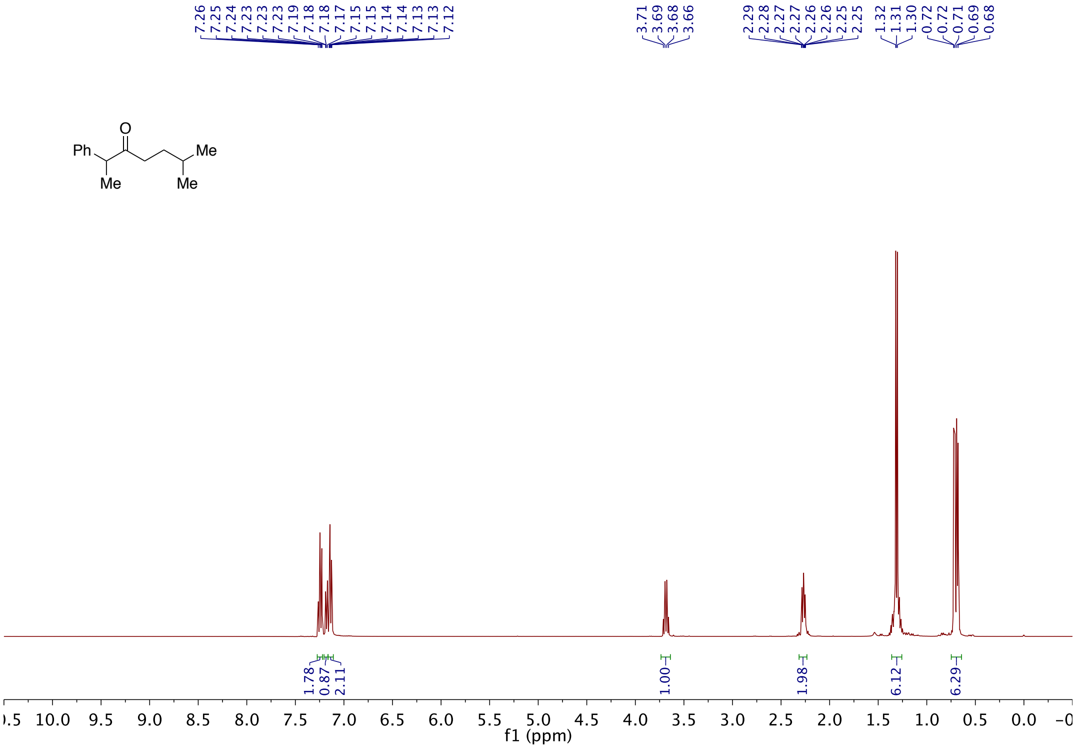

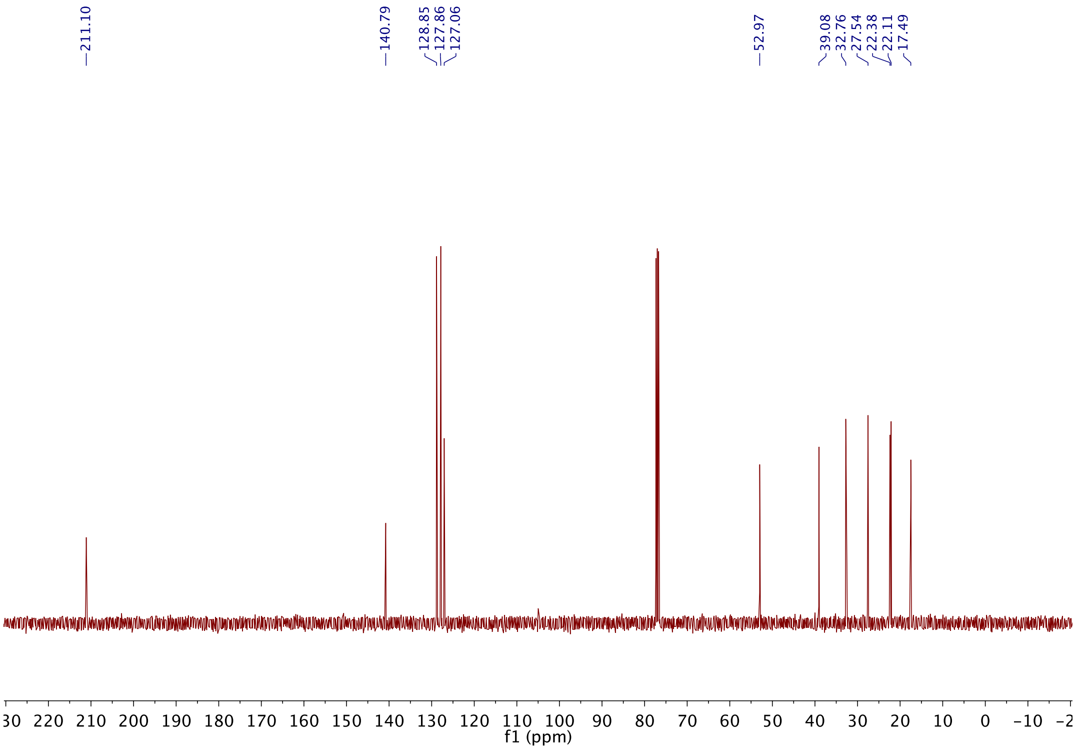

^{1}H and ^{13}C NMR spectra of 7-methyl-3-phenyloctan-4-one (**1q**)

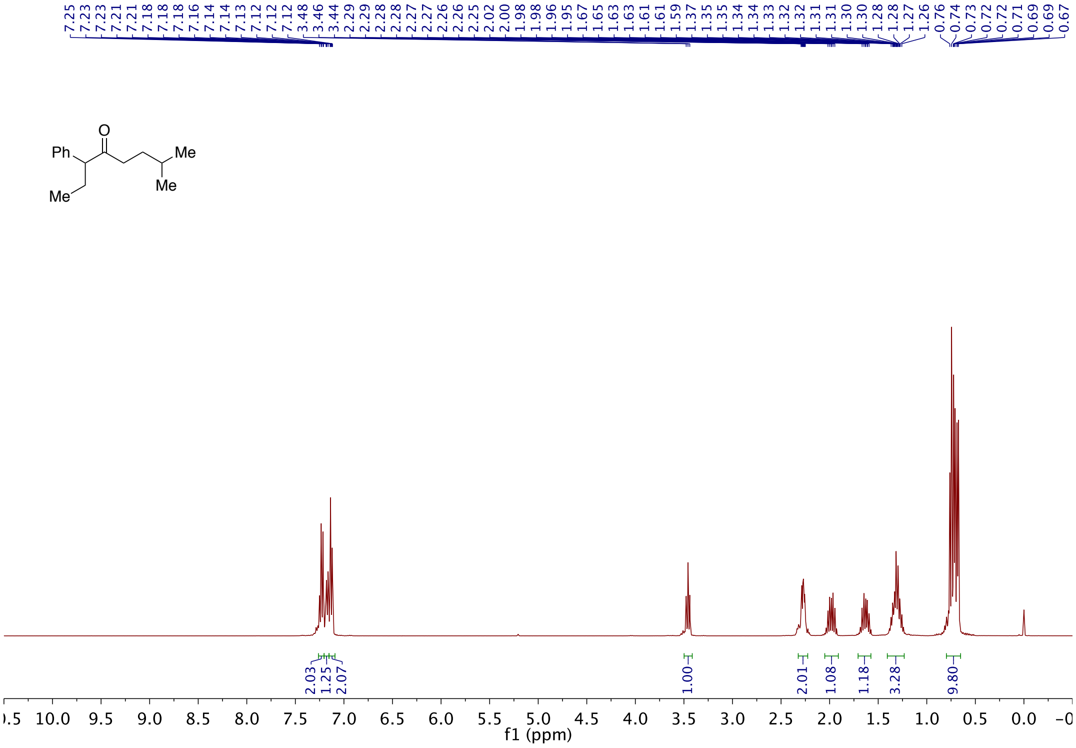

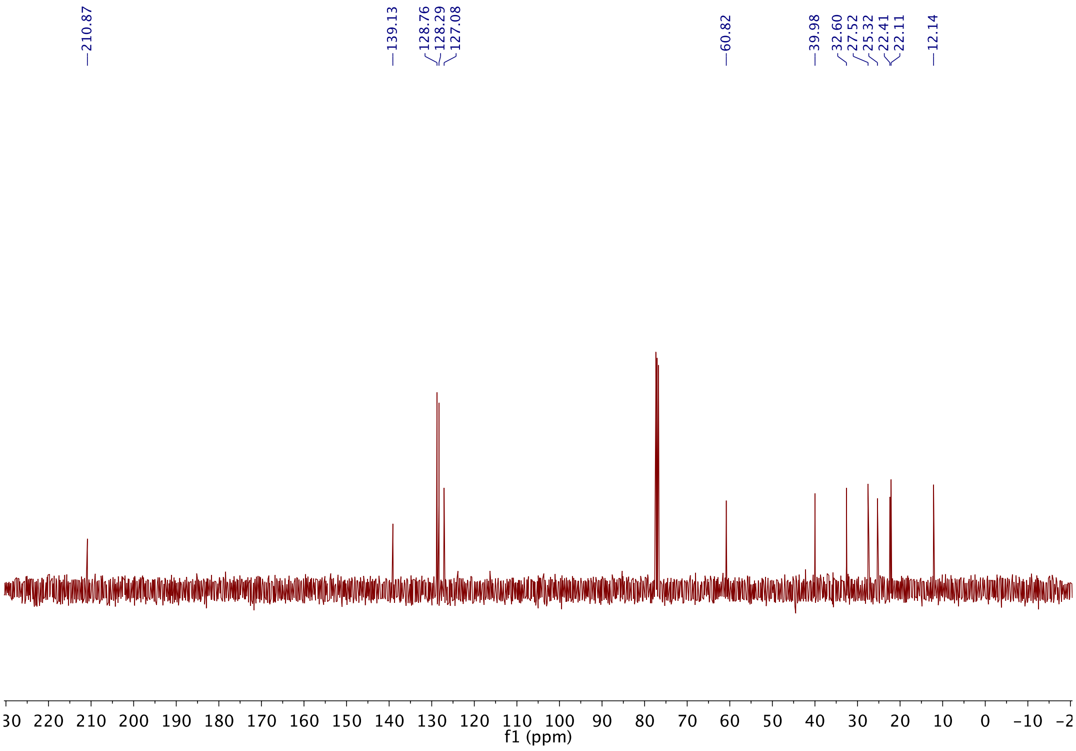

^{1}H and ^{13}C NMR spectra of 6-methyl-2-phenoxyheptan-3-one (**1r**)

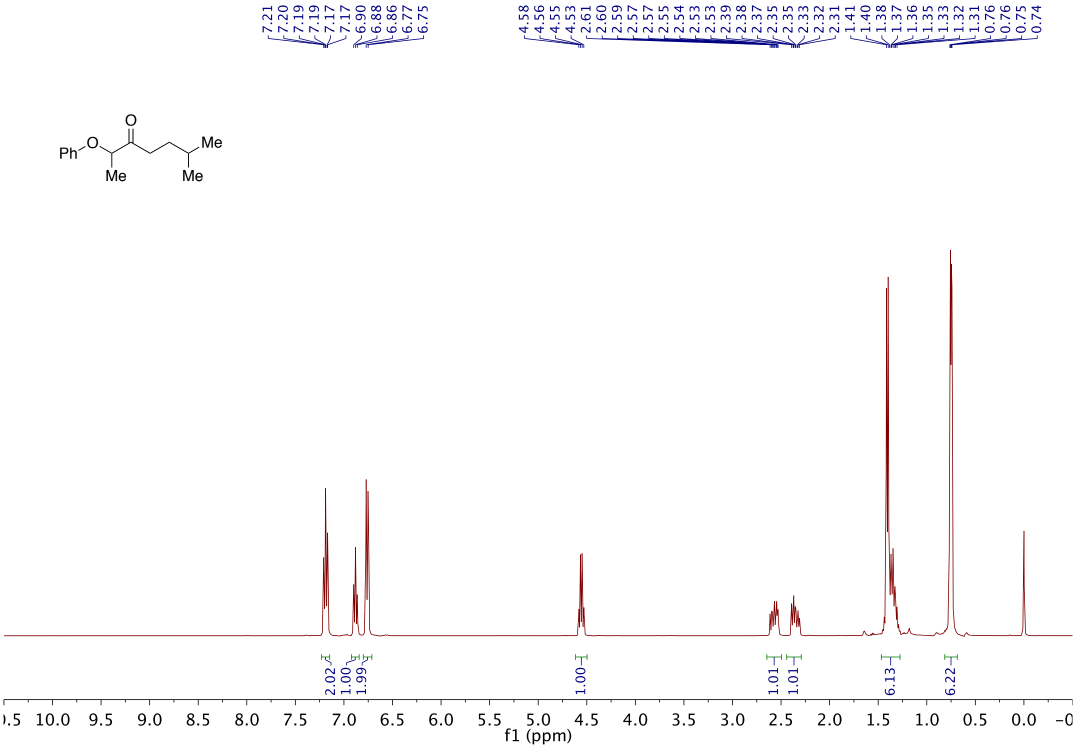

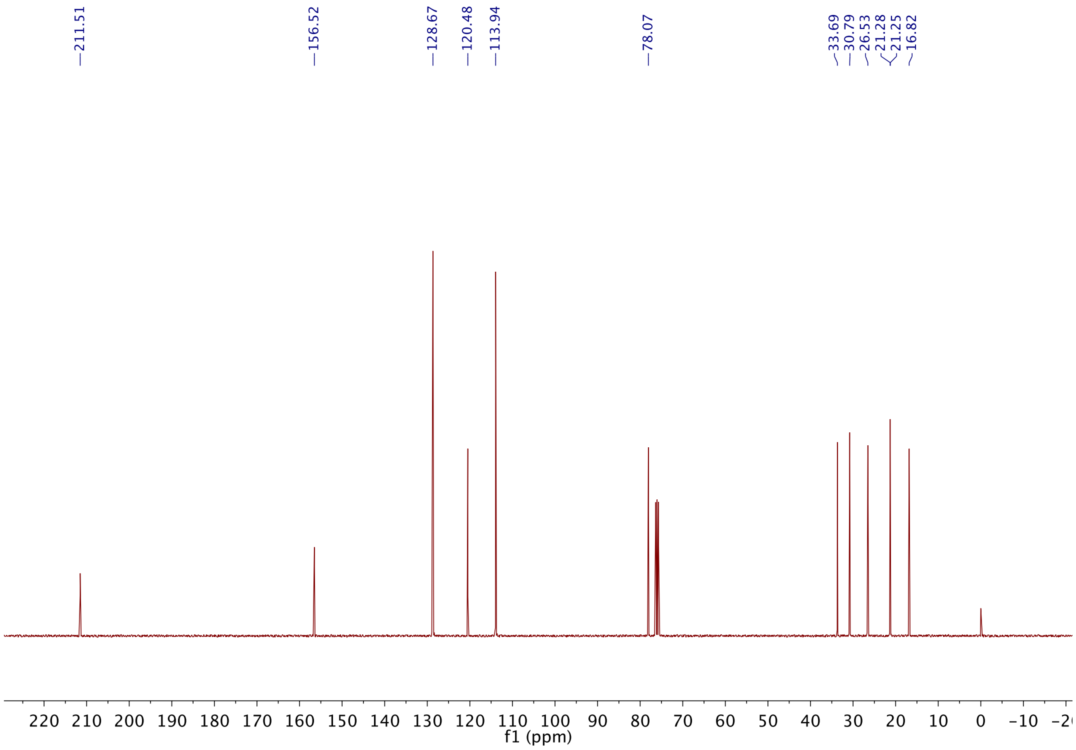

^{1}H and ^{13}C NMR spectra of 1-(4-(trifluoromethyl)phenyl)pentan-1-one (**1t**)

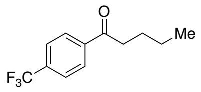

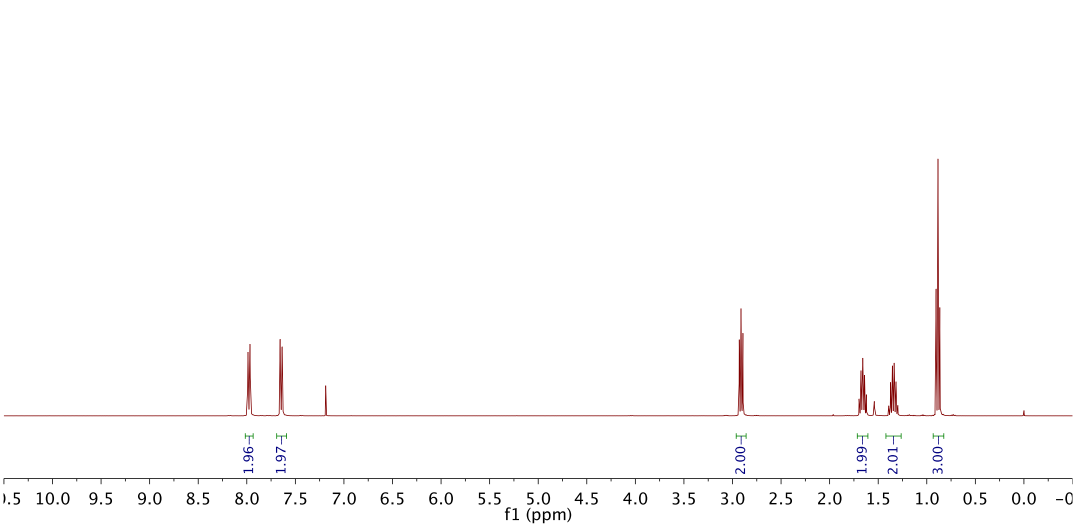

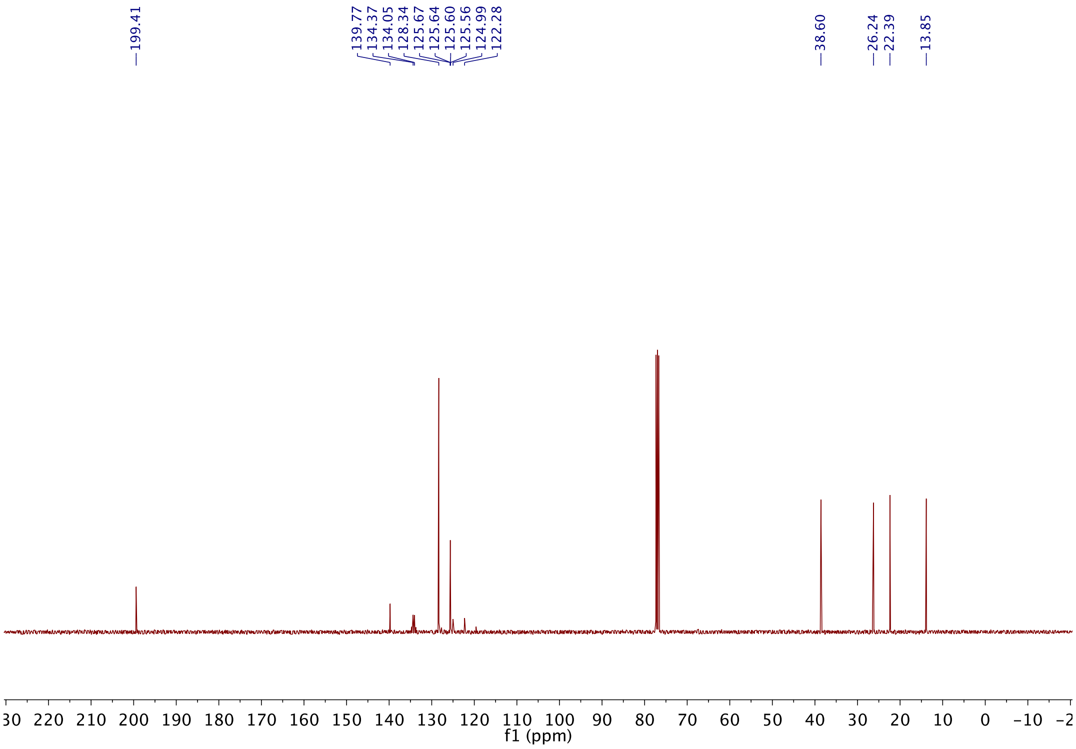

^{1}H and ^{13}C NMR spectra of 1-(3-fluorophenyl)pentan-1-one (**1v**)

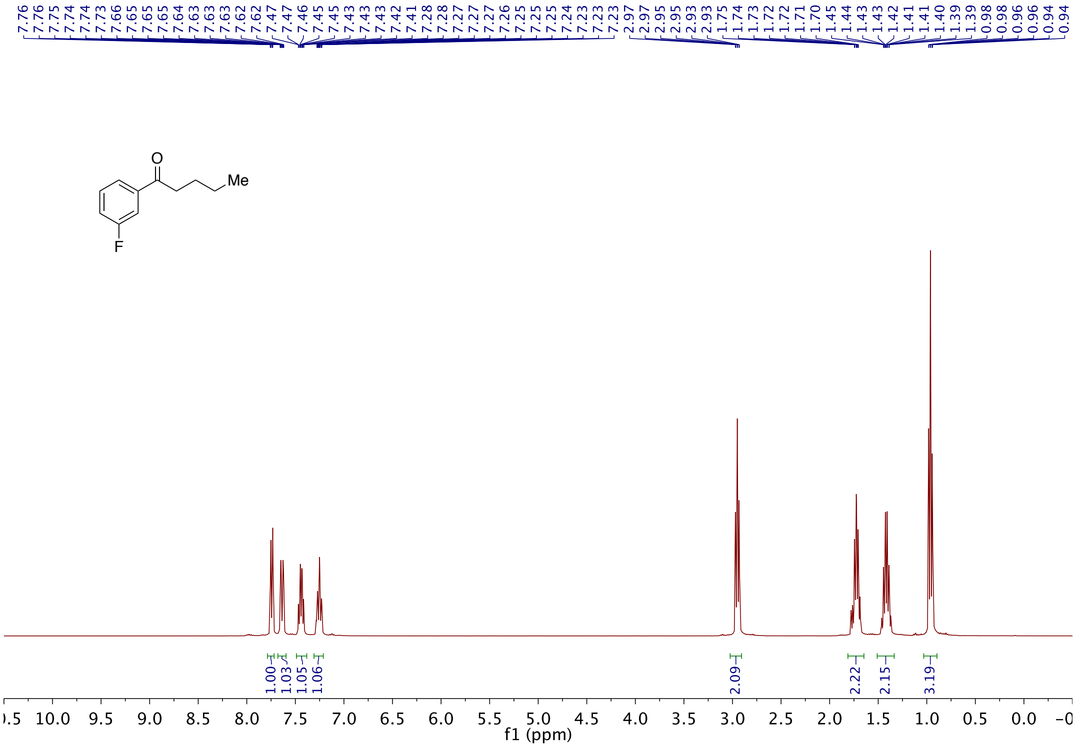

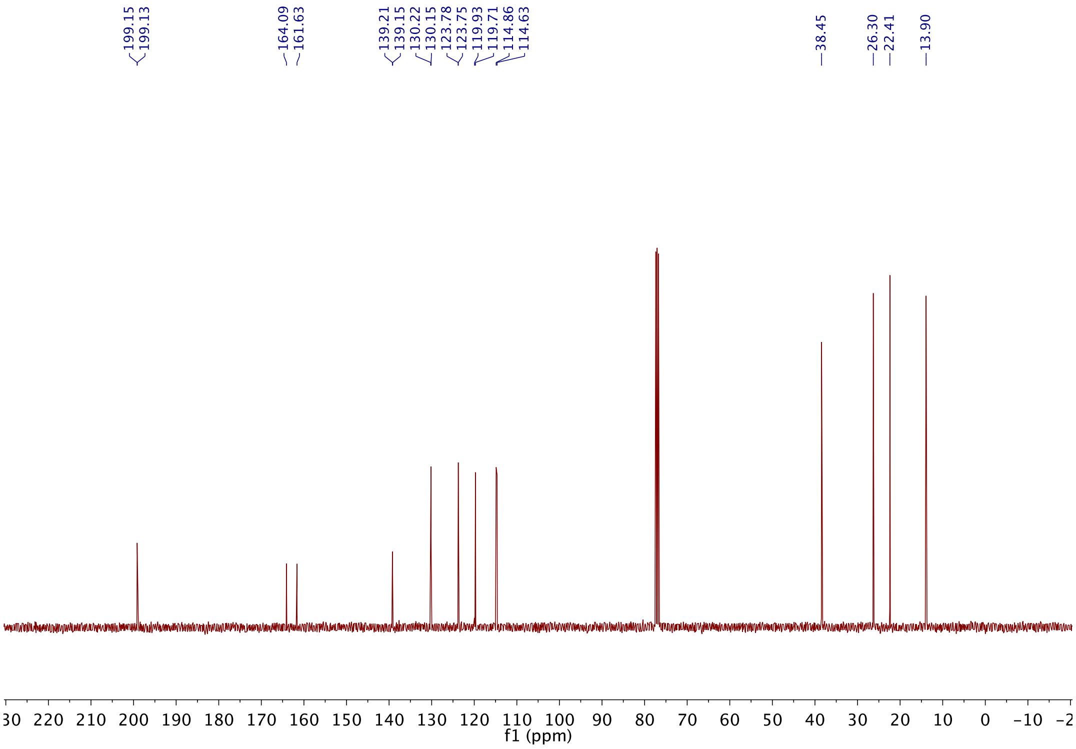

^{1}H and ^{13}C NMR spectra of 1-(3-chloro-4-methoxyphenyl)pentan-1-one (**1w**)

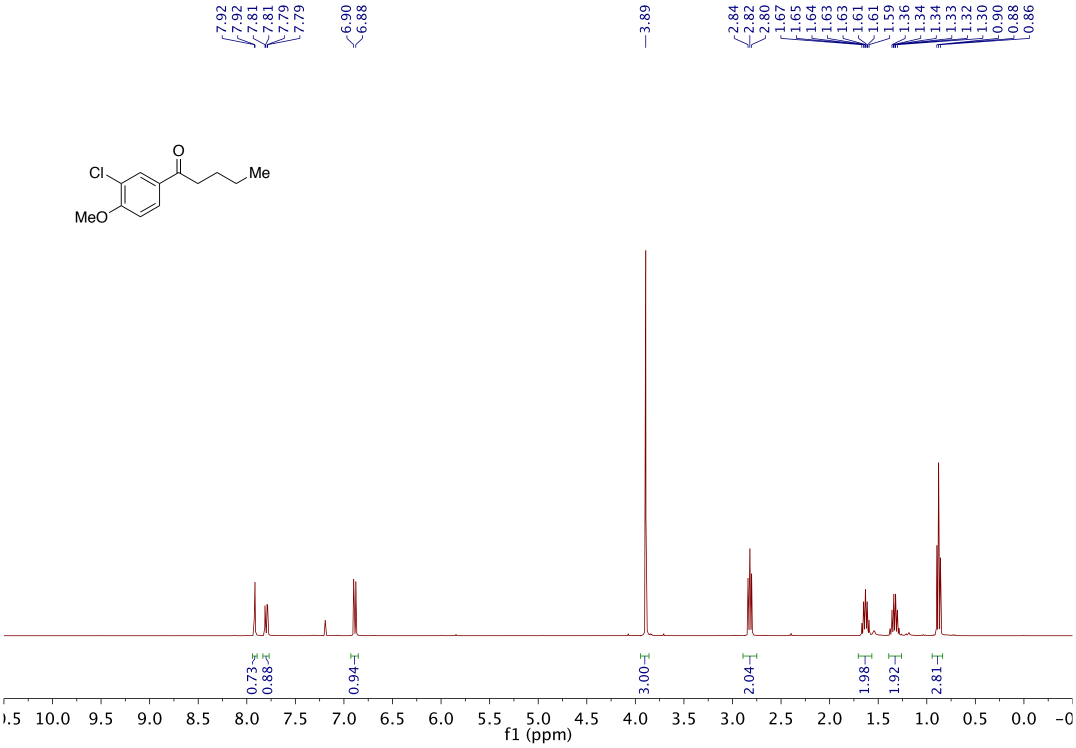

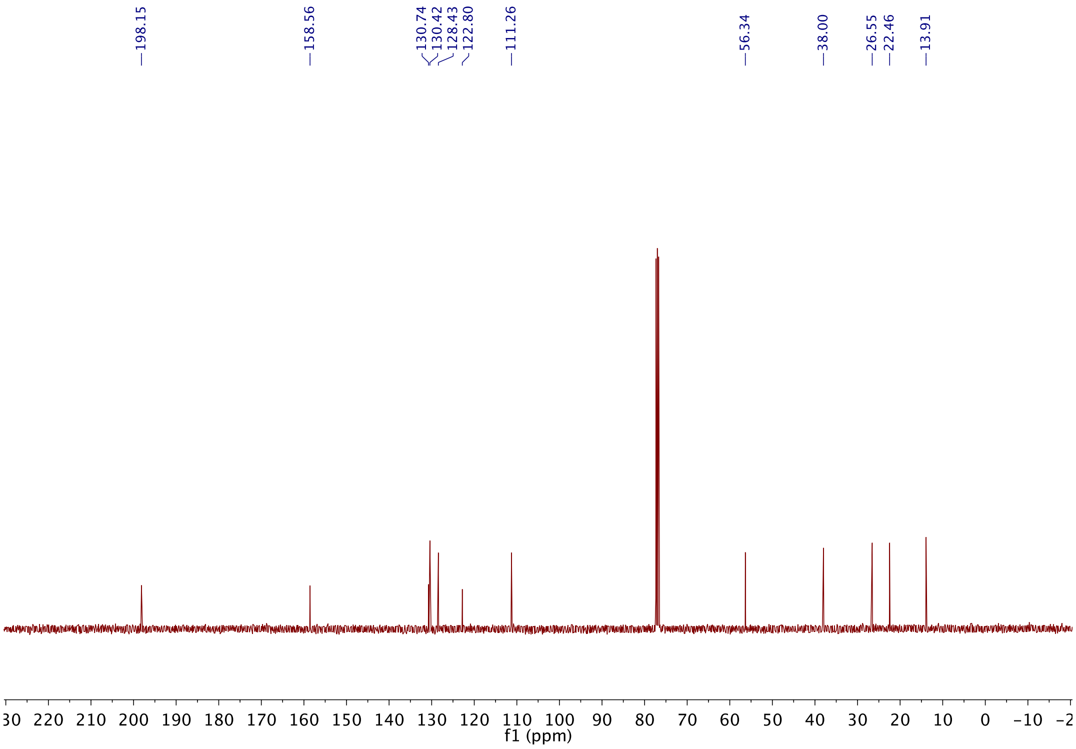

^{1}H and ^{13}C NMR spectra of 2-phenoxyheptan-3-one (**1x**)

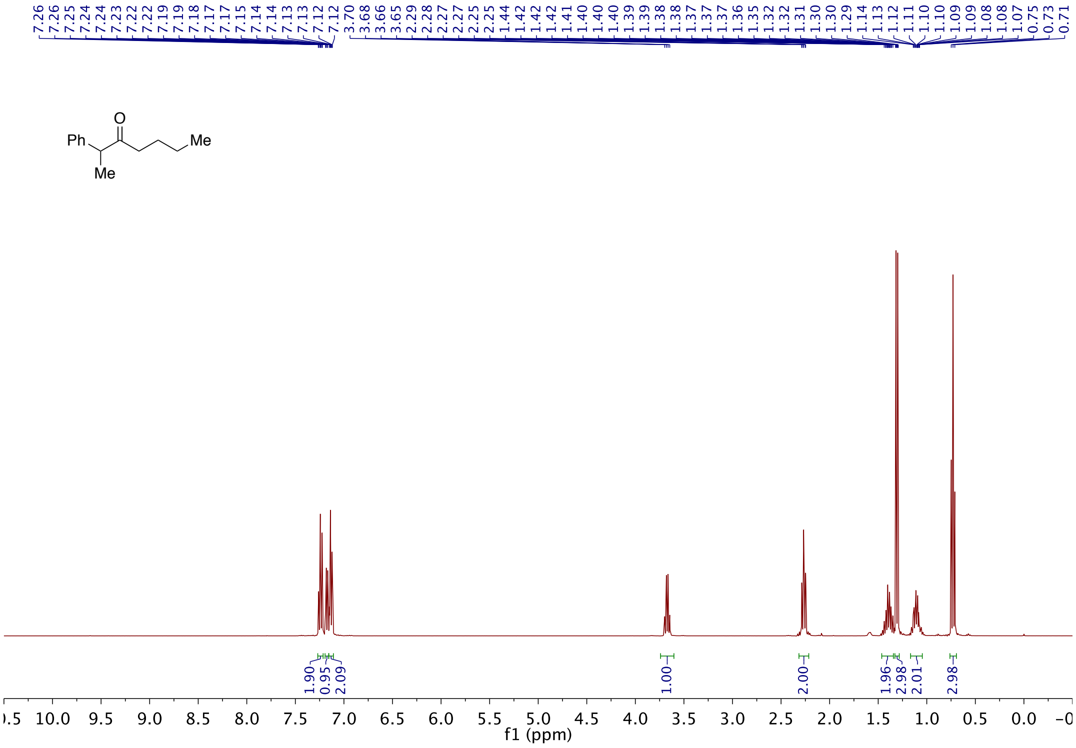

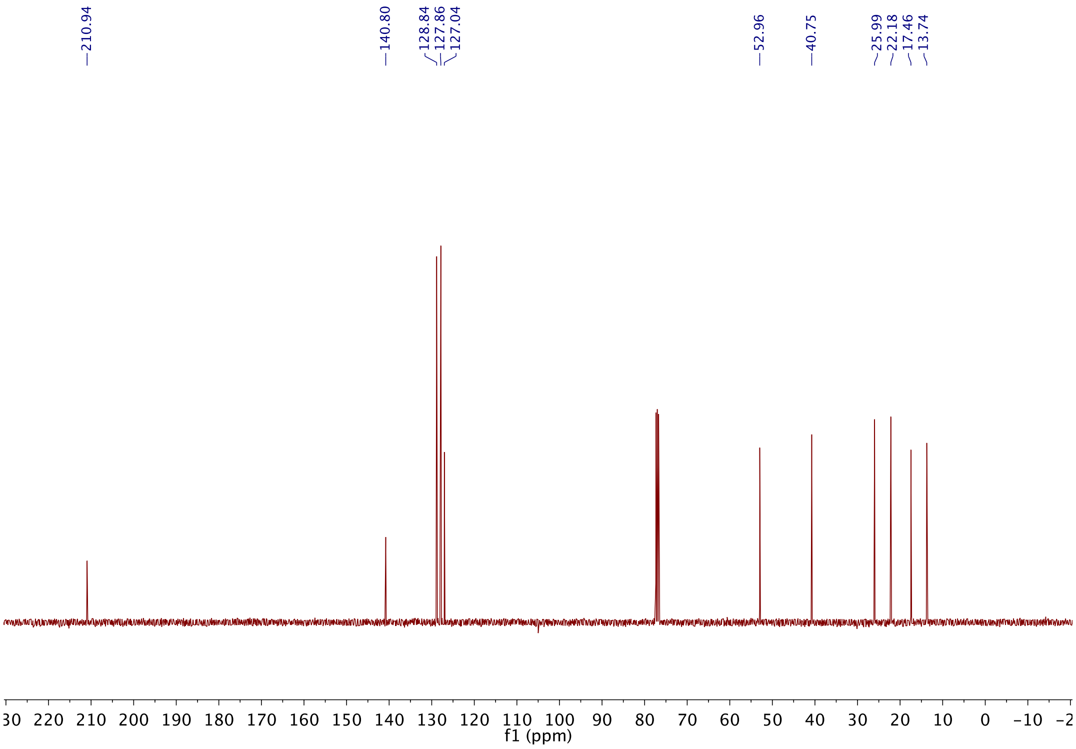

^{1}H and ^{13}C NMR spectra of phenyl 4-methylpentanoate (**1y**)

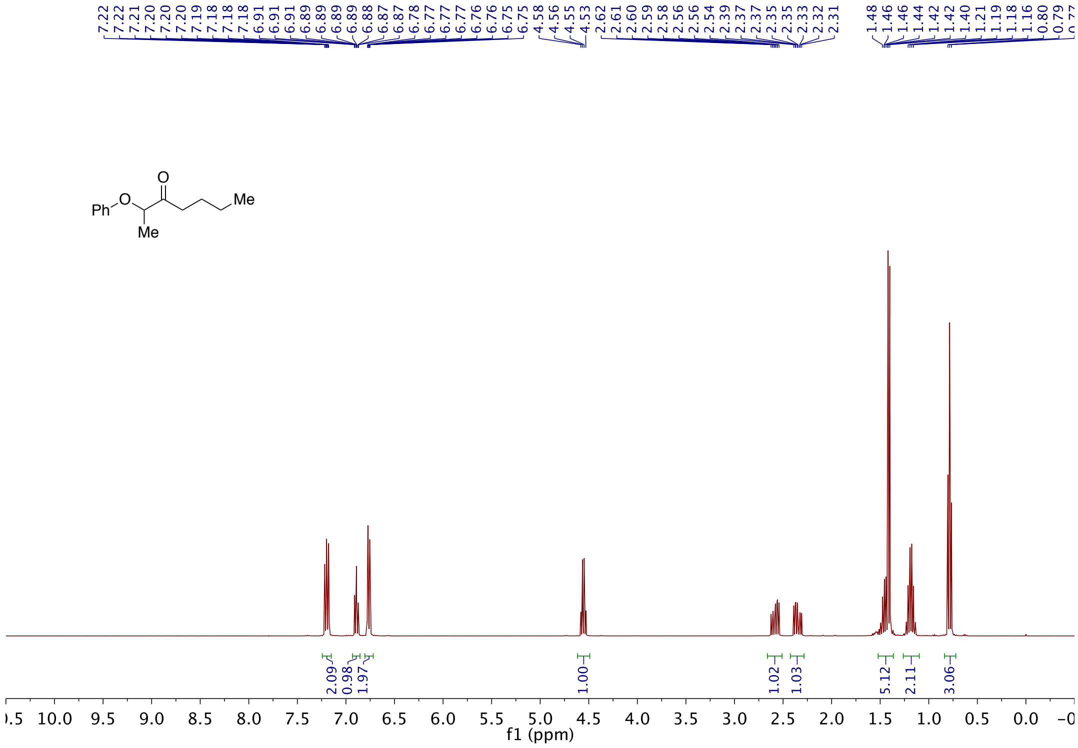

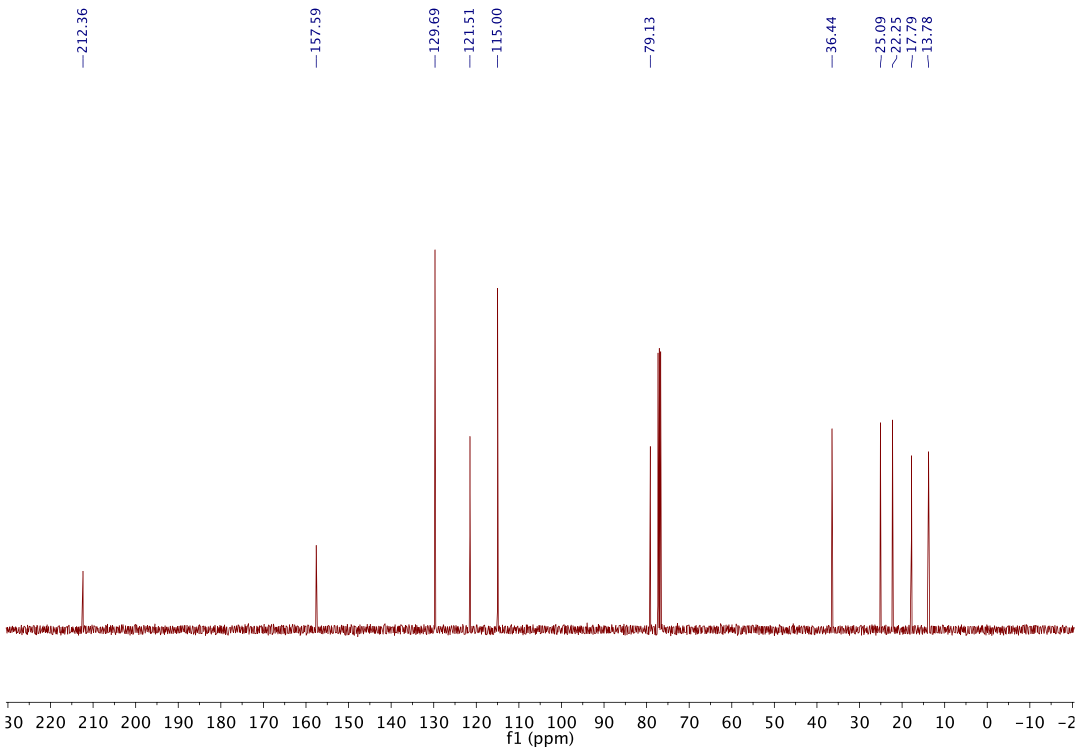

^{1}H and ^{13}C NMR spectra of of 2-(1,3-dioxoisoindolin-2-yl)ethyl 4-methylpentanoate (**1z**)

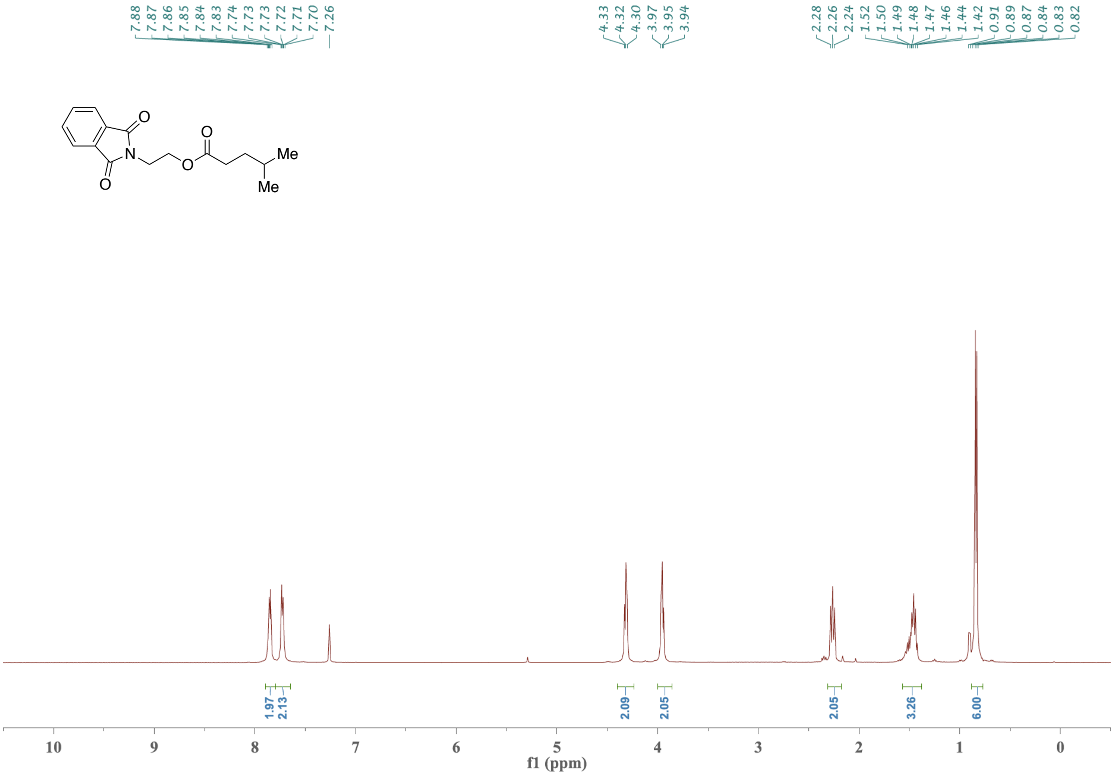

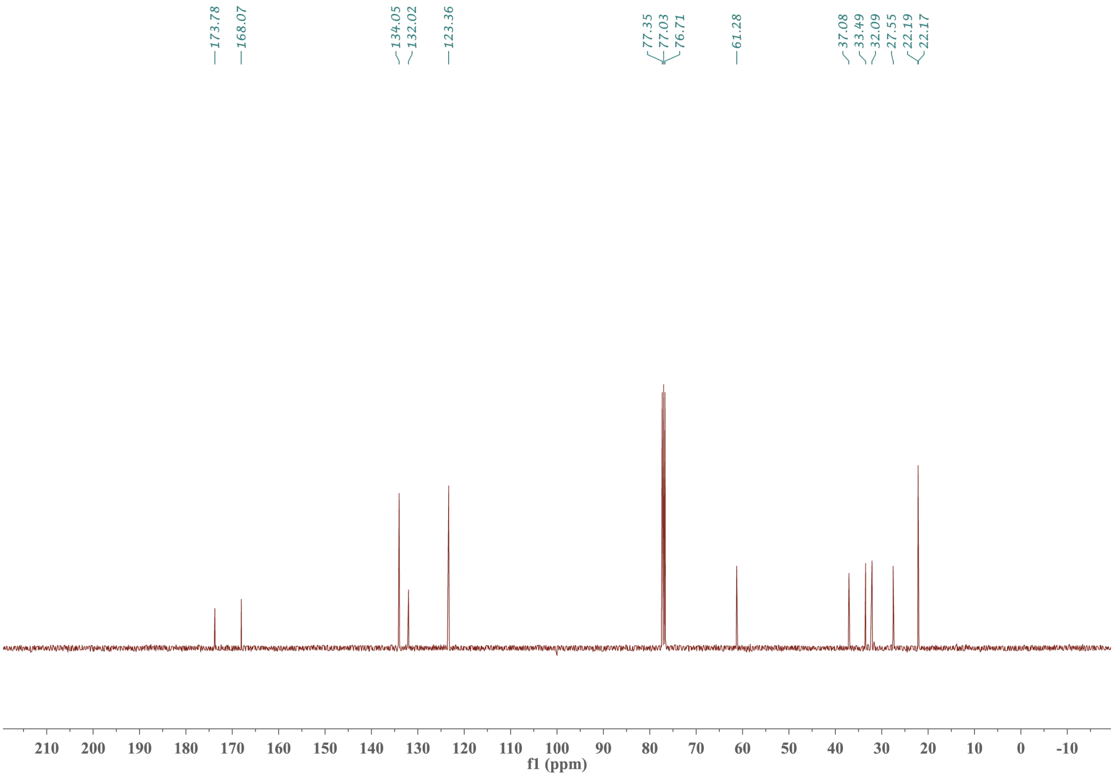

^{1}H and ^{13}C NMR spectra of *N*,4-dimethyl-*N*-phenylpentanamide (**1α**)

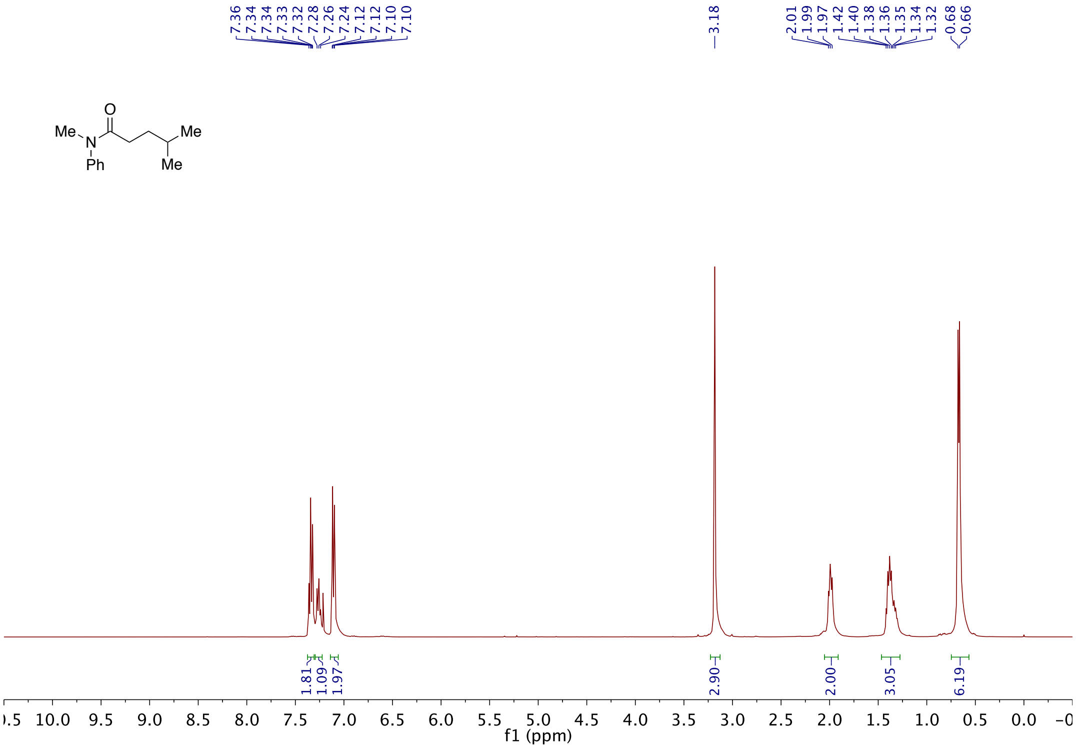

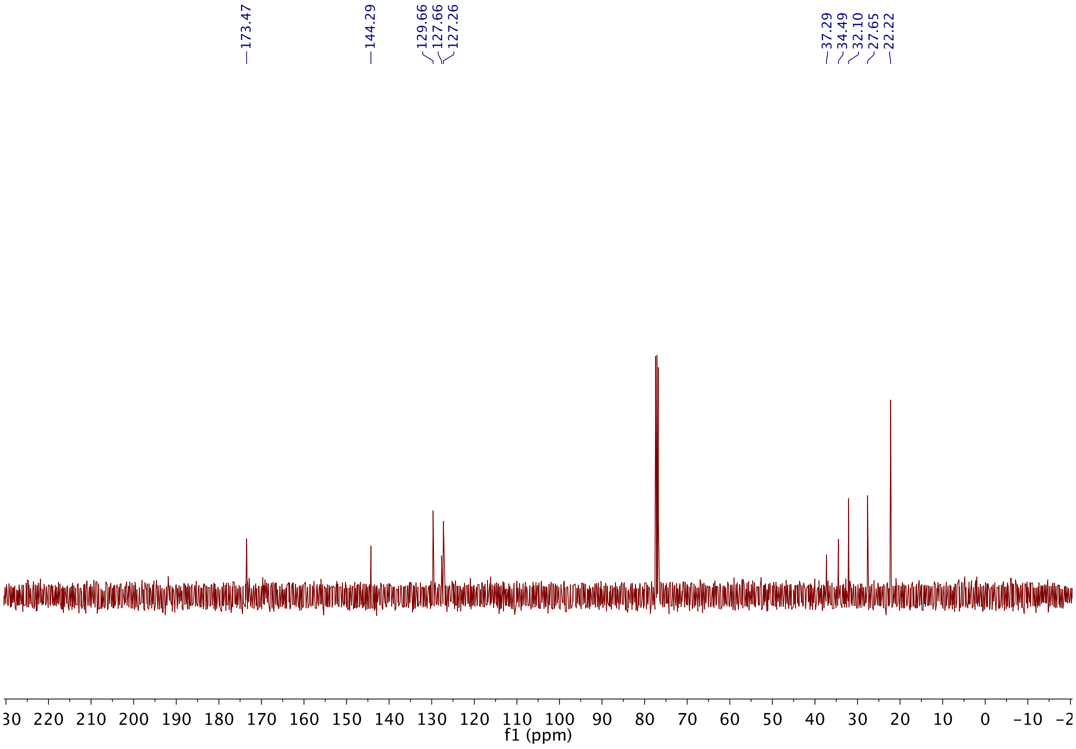

^{1}H and ^{13}C NMR spectra of (*E*)-4-methyl-1-phenylpent-2-en-1-one (**3a**)

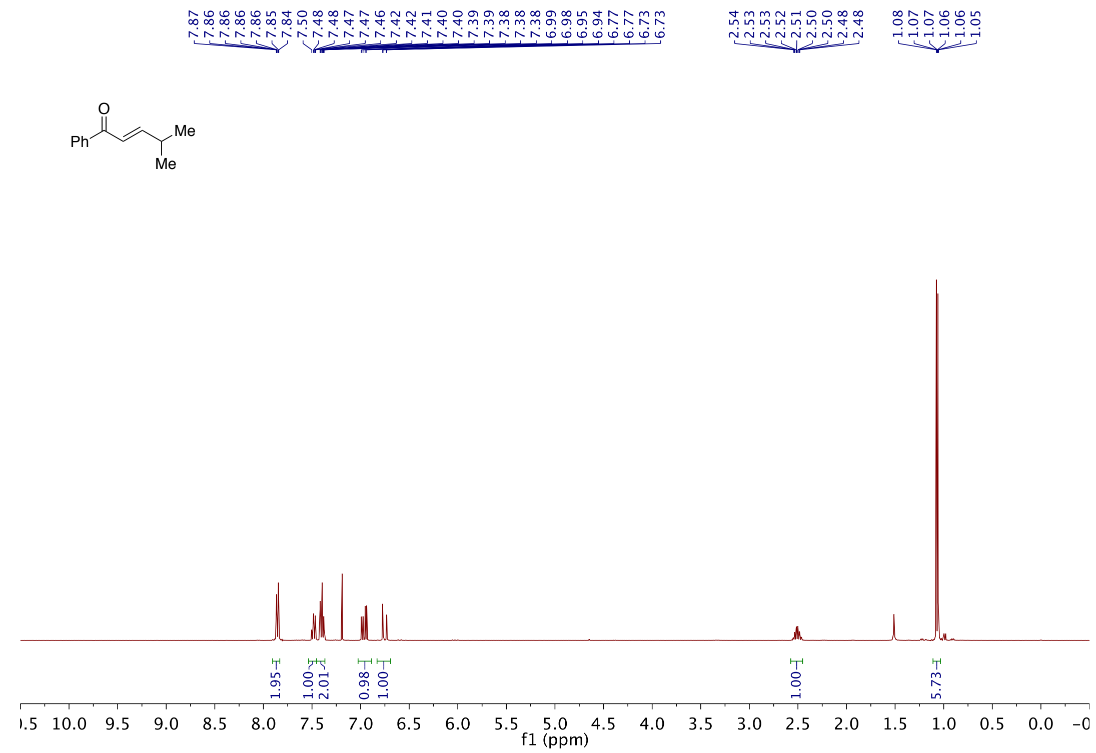

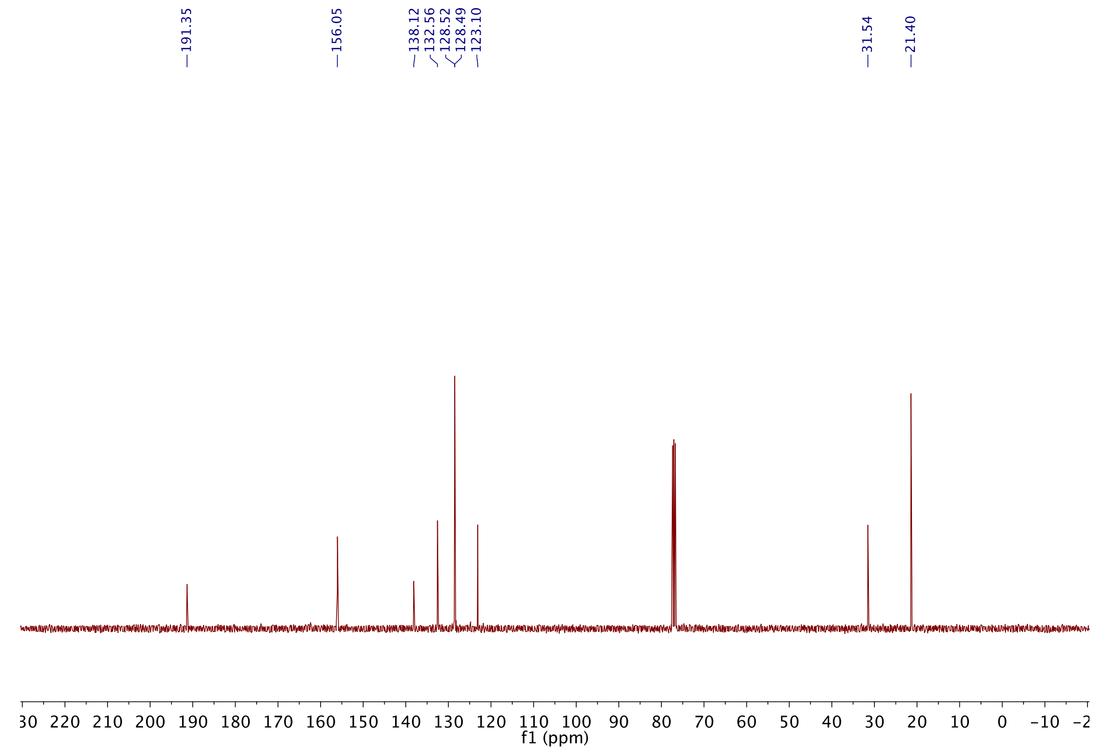

^{1}H and ^{13}C NMR spectra of (*E*)-1-(4-methoxyphenyl)-4-methylpent-2-en-1-one (**3b**)

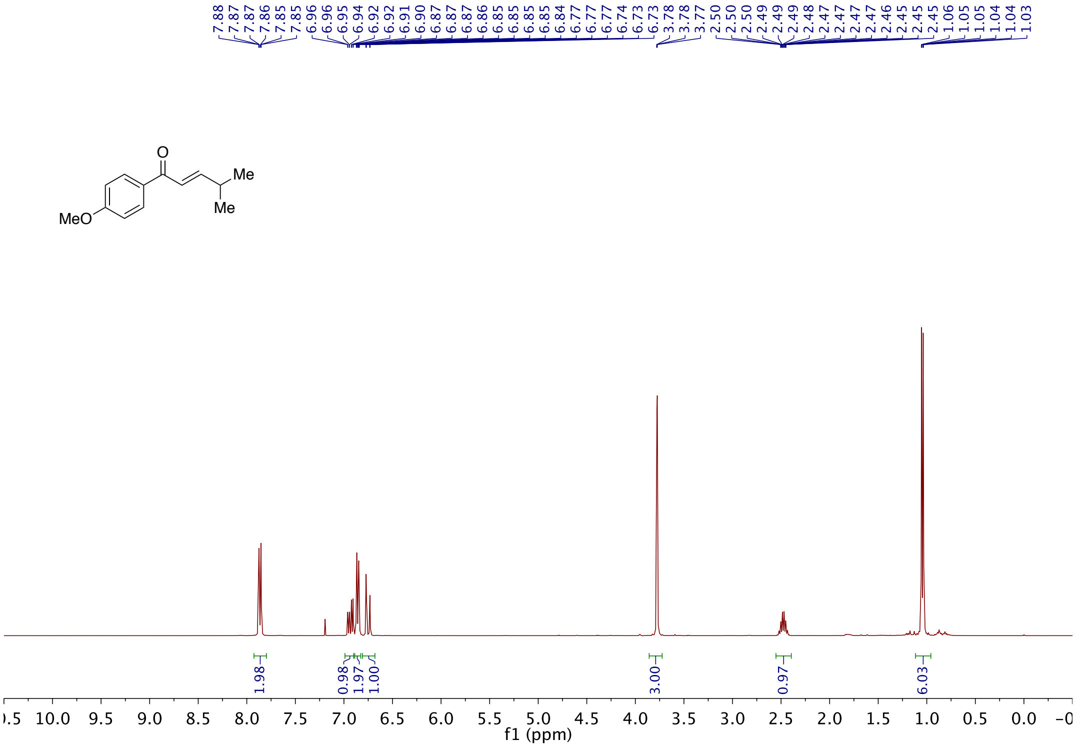
O
Me
Me
MeO
7.88 7.87 7.87 7.86 7.85 7.85 6.96 6.96 6.95 6.94 6.92 6.92 6.91 6.90 6.87 6.87 6.87 6.86 6.85 6.85 6.85 6.84 6.77 6.77 6.77 6.74 6.73 6.73 3.78 3.78 3.77 2.50 2.50 2.49 2.49 2.48 2.47 2.47 2.47 2.46 2.45 2.45 1.06 1.05 1.05 1.04 1.04 1.03
1.98
0.98
1.97
1.00
3.00
0.97
6.03
f1 (ppm)

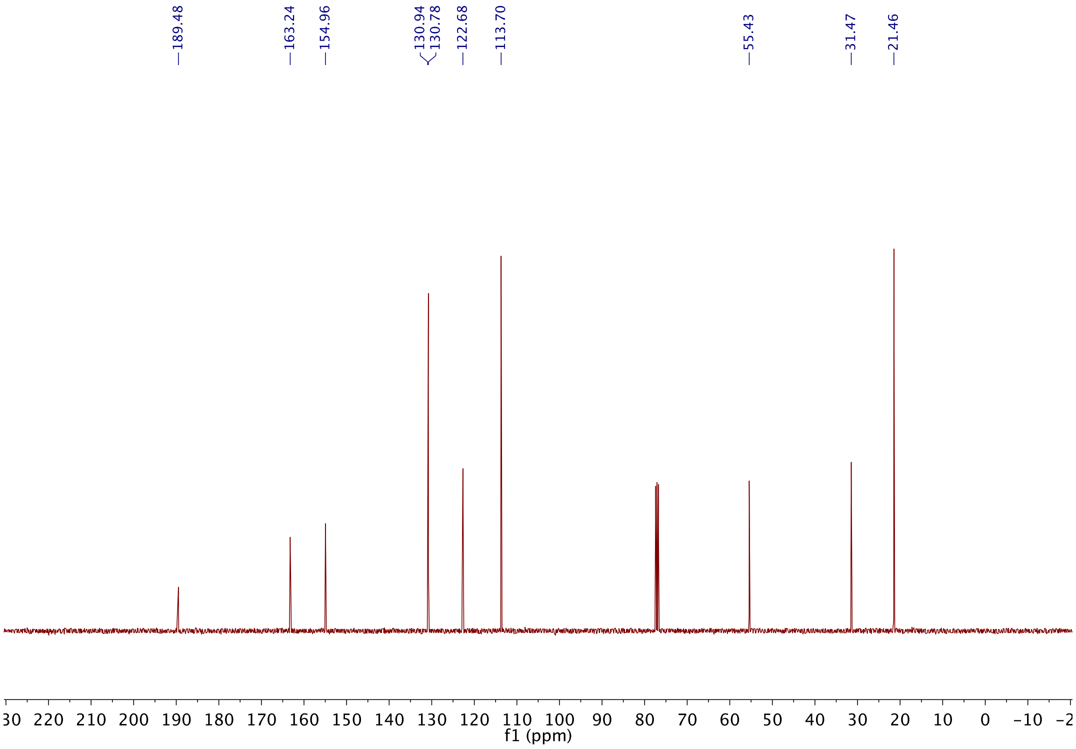
189.48
163.24
154.96
130.94
130.78
122.68
113.70
55.43
31.47
21.46
f1 (ppm)

^{1}H and ^{13}C NMR spectra of (*E*)-4-methyl-1-(*p*-tolyl)pent-2-en-1-one (**3c**)

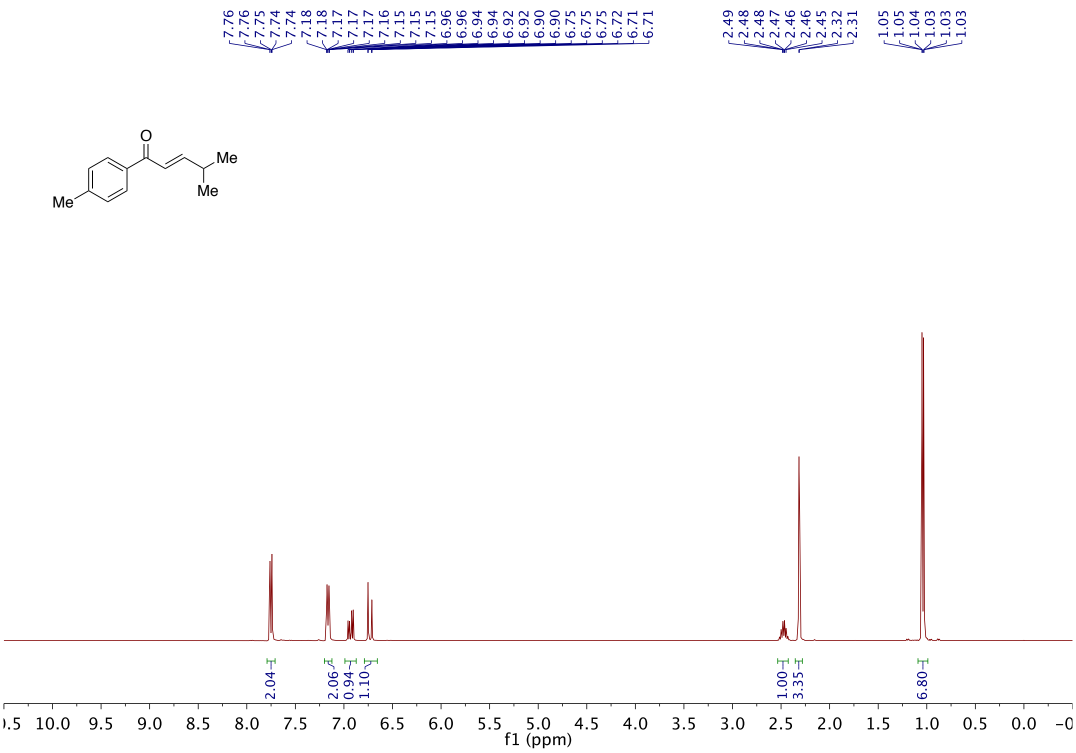

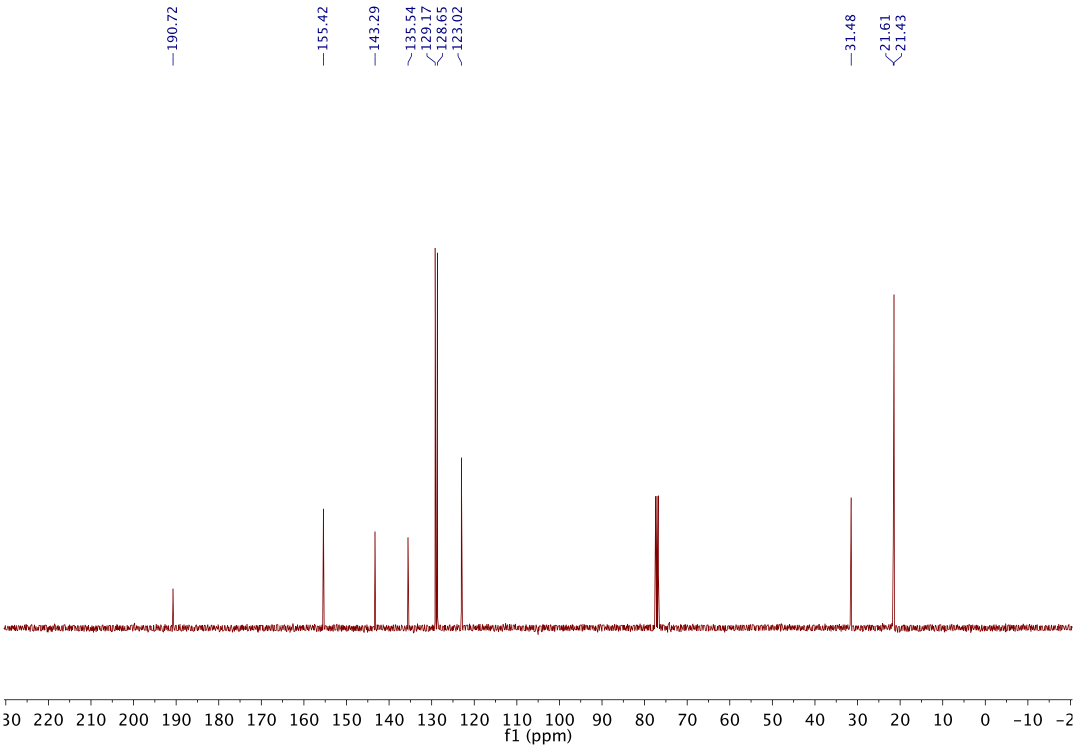

^{1}H and ^{13}C NMR spectra of (*E*)-1-([1,1'-biphenyl]-4-yl)-4-methylpent-2-en-1-one (**3d**)

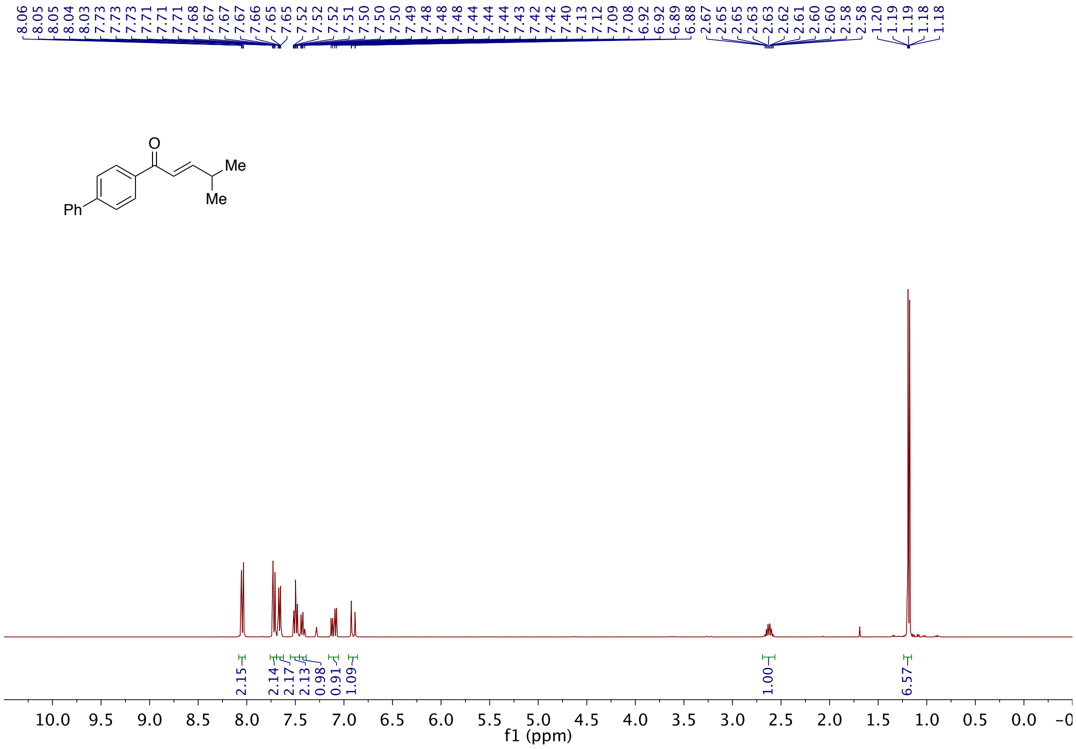

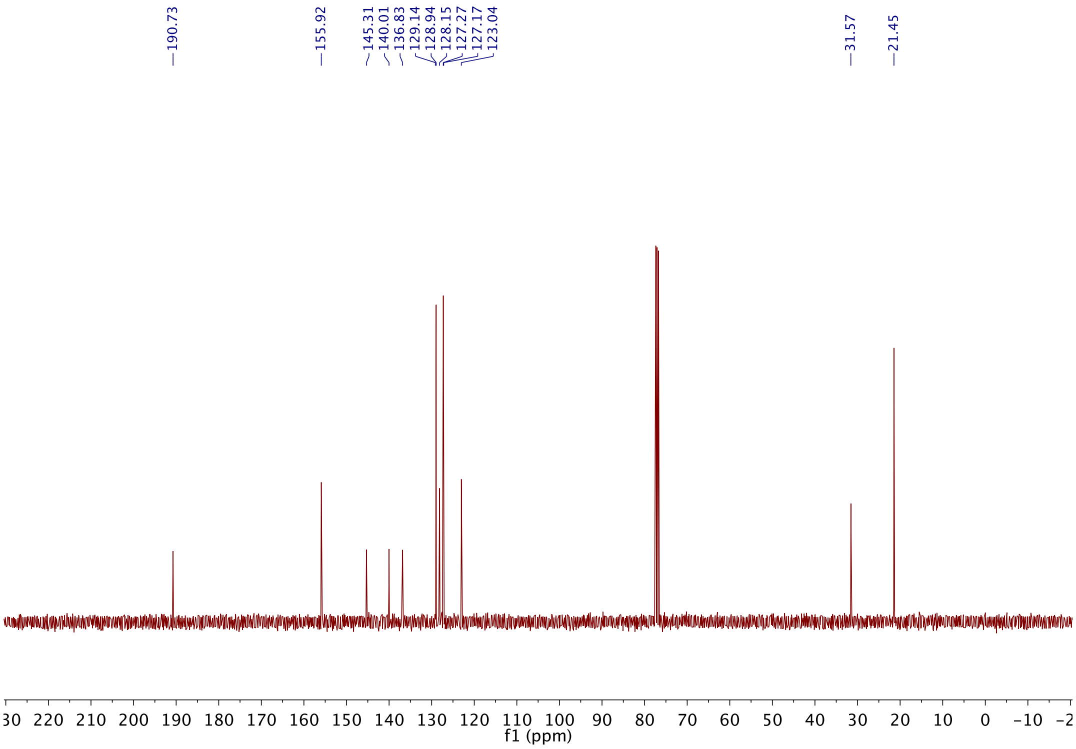

^{1}H and ^{13}C NMR spectra of (*E*)-4-methyl-1-(4-(trifluoromethyl)phenyl)pent-2-en-1-one (**3e**)

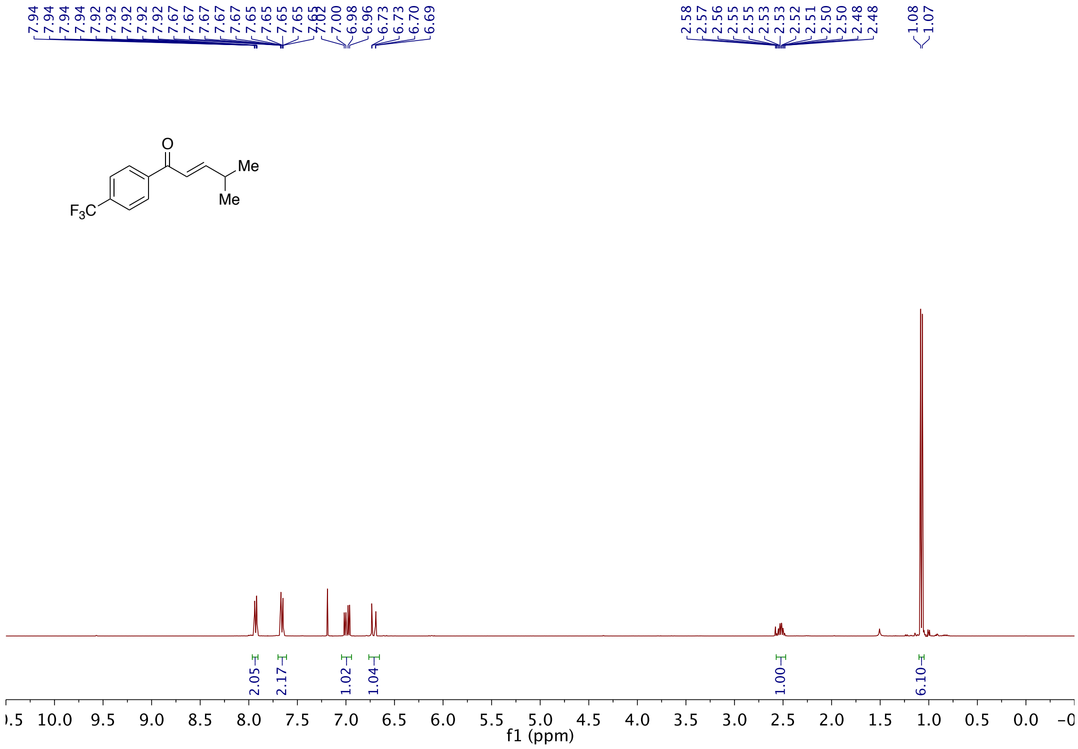

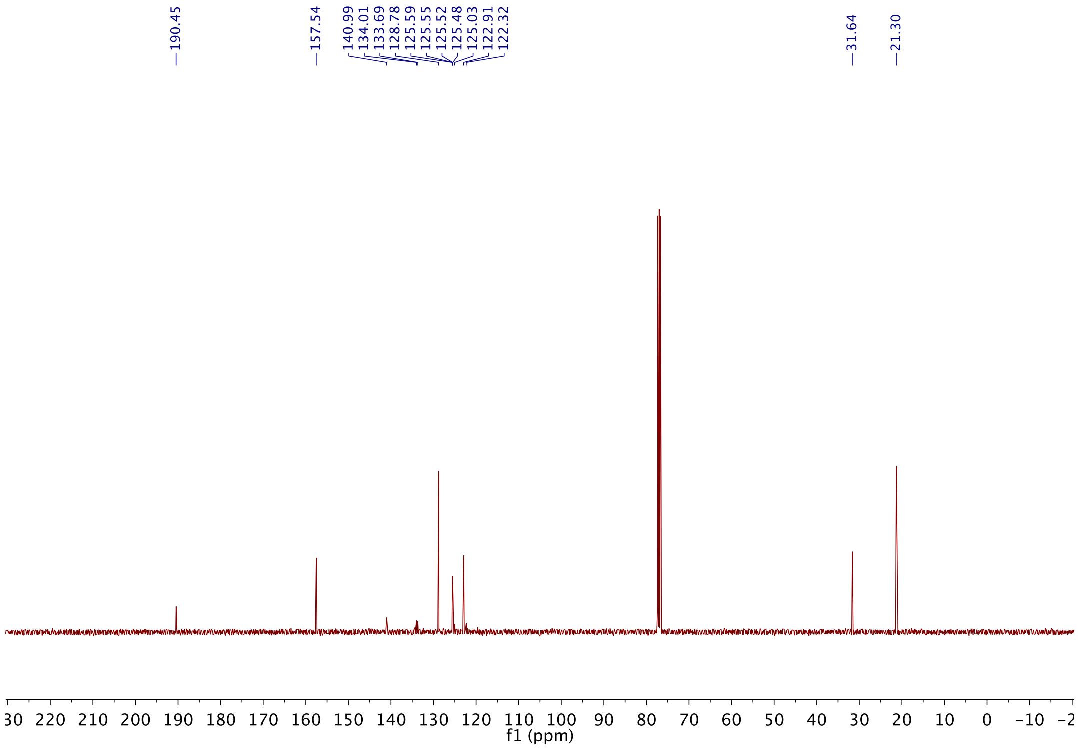

^{1}H and ^{13}C NMR spectra of (*E*)-1-(2,4-dimethylphenyl)-4-methylpent-2-en-1-one (**3f**)

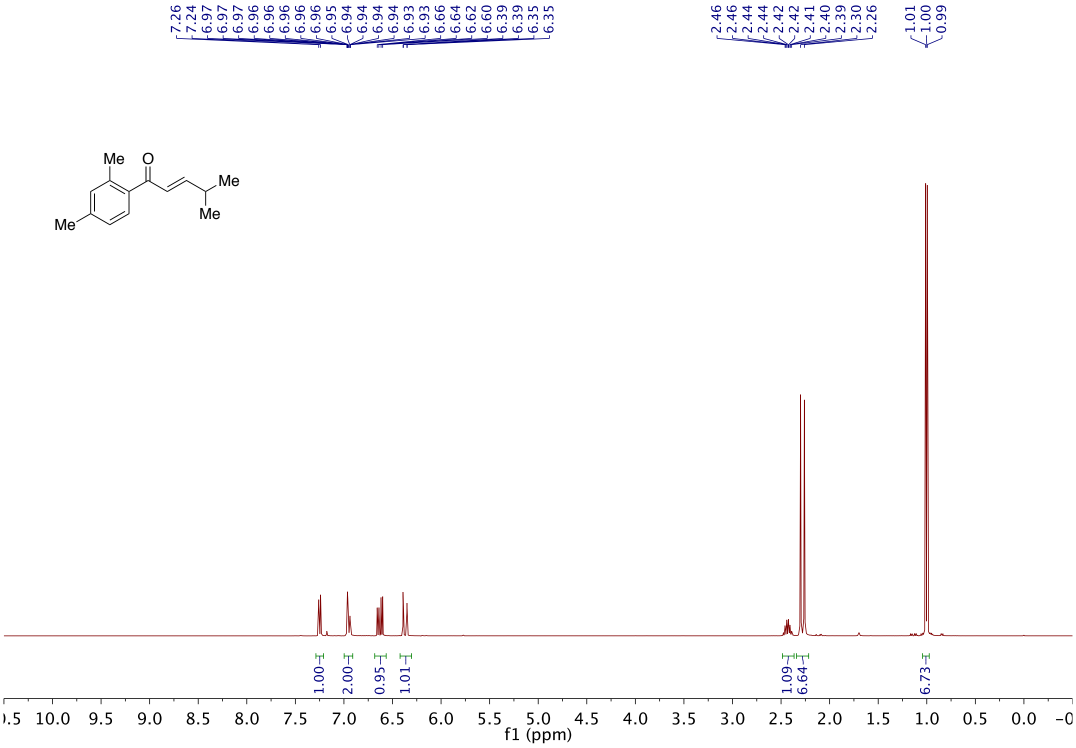

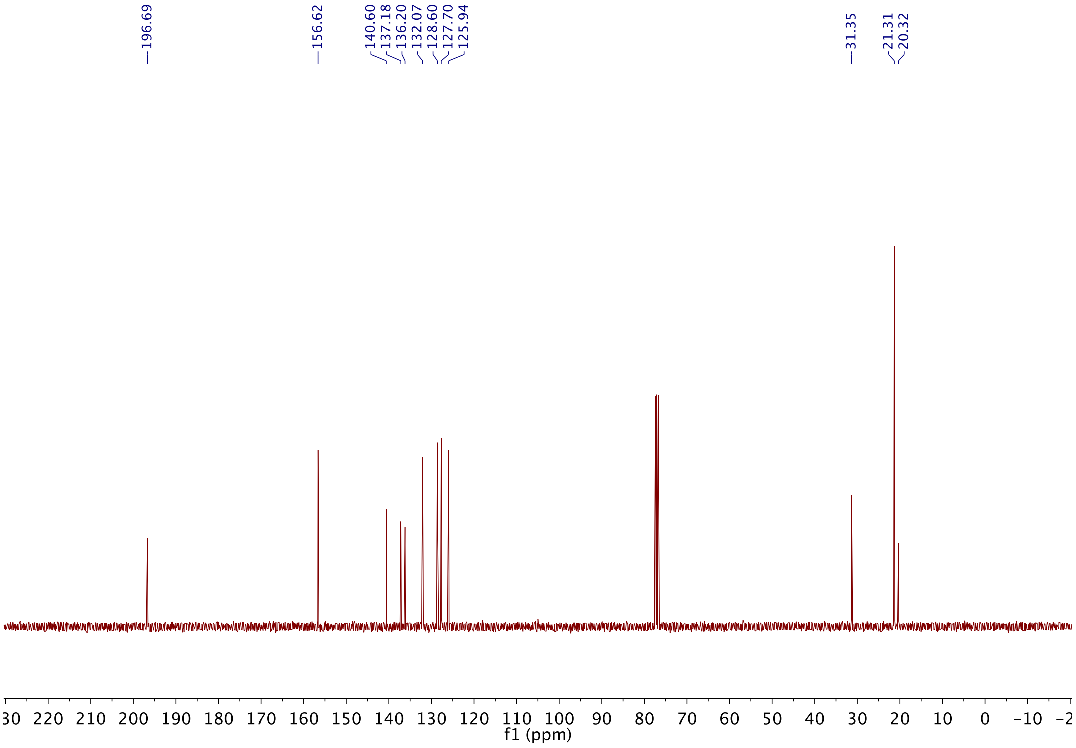

^{1}H and ^{13}C NMR spectra of (*E*)-4-methyl-1-(naphthalen-2-yl)pent-2-en-1-one (**3g**)

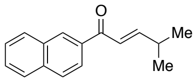

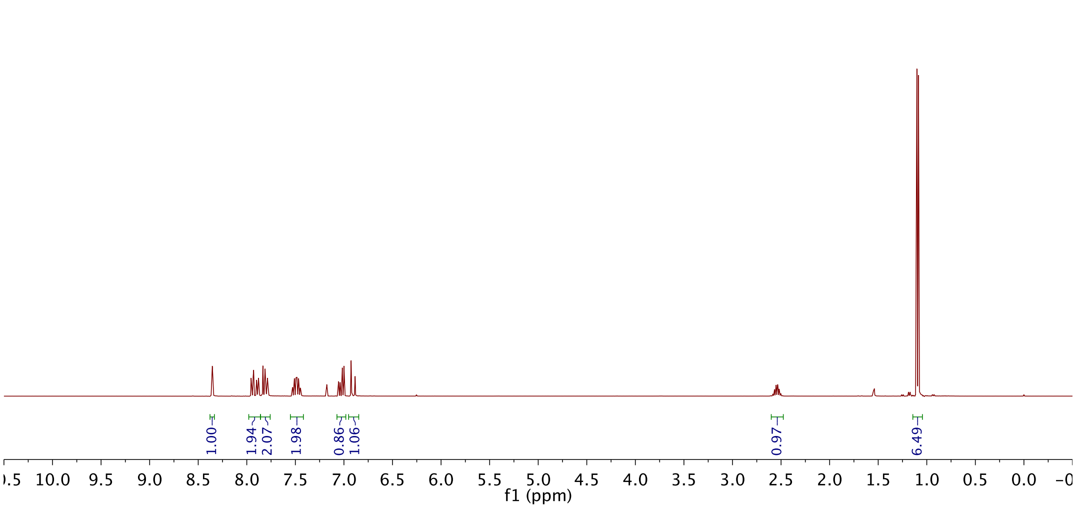

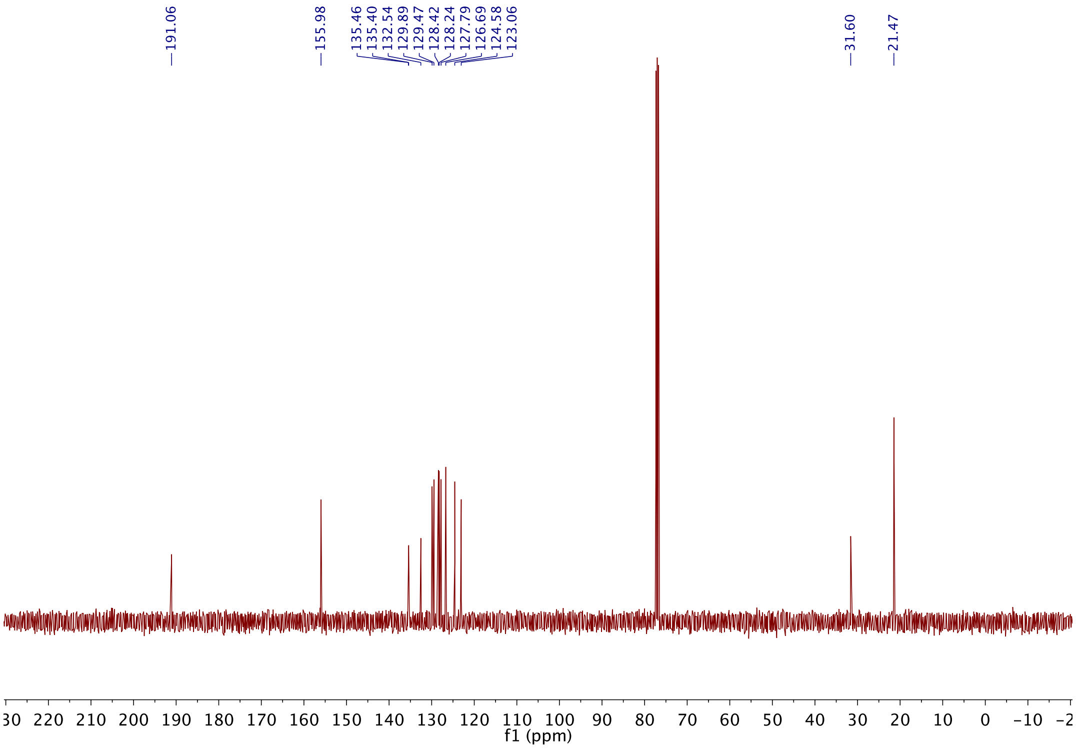

^{1}H and ^{13}C NMR spectra of (*E*)-4-methyl-1-(thiophen-2-yl)pent-2-en-1-one (**3h**)

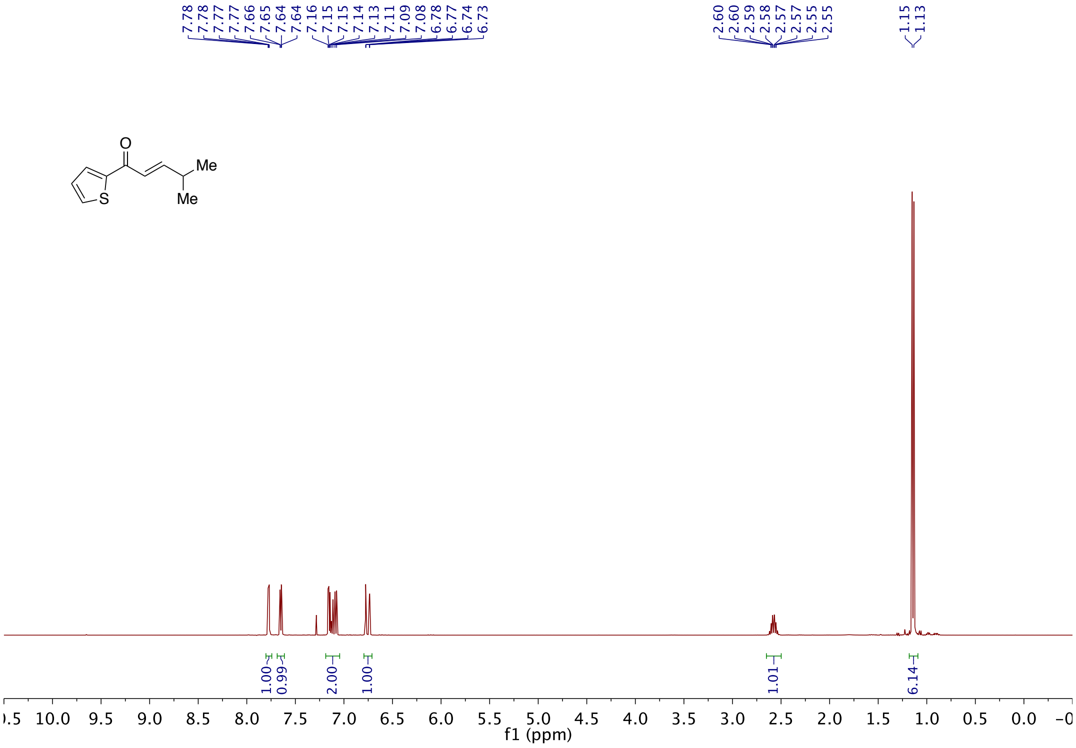

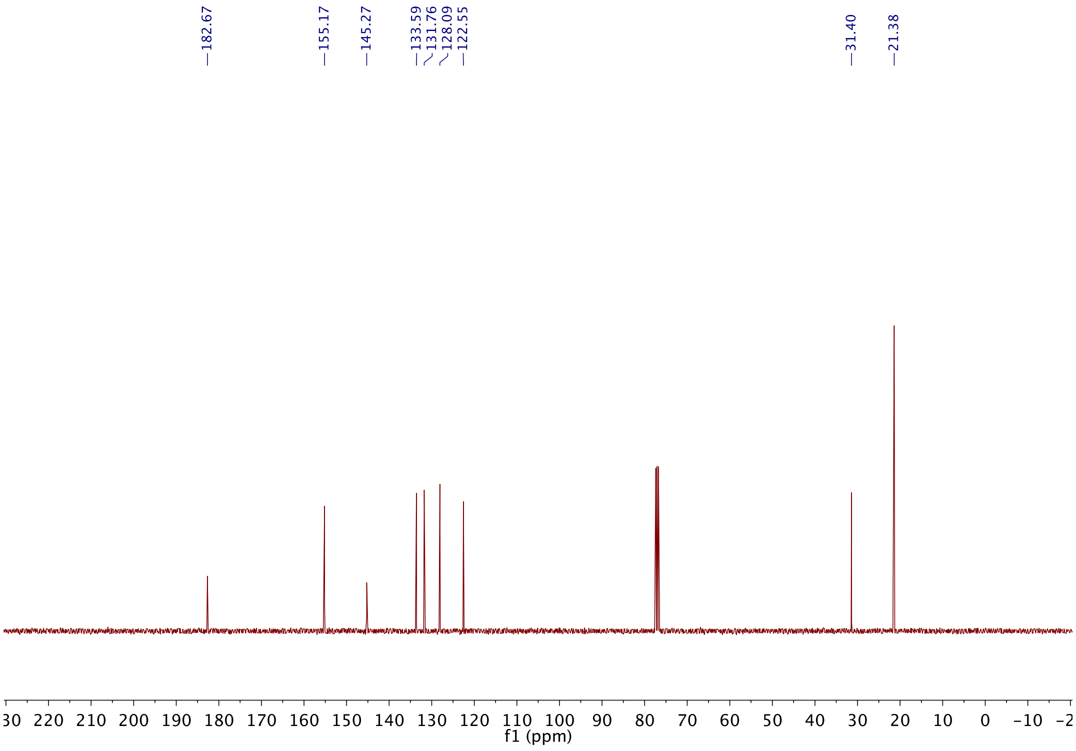

^{1}H and ^{13}C NMR spectra of (*E*)-4-methyl-1-phenylhex-2-en-1-one (**3i**)

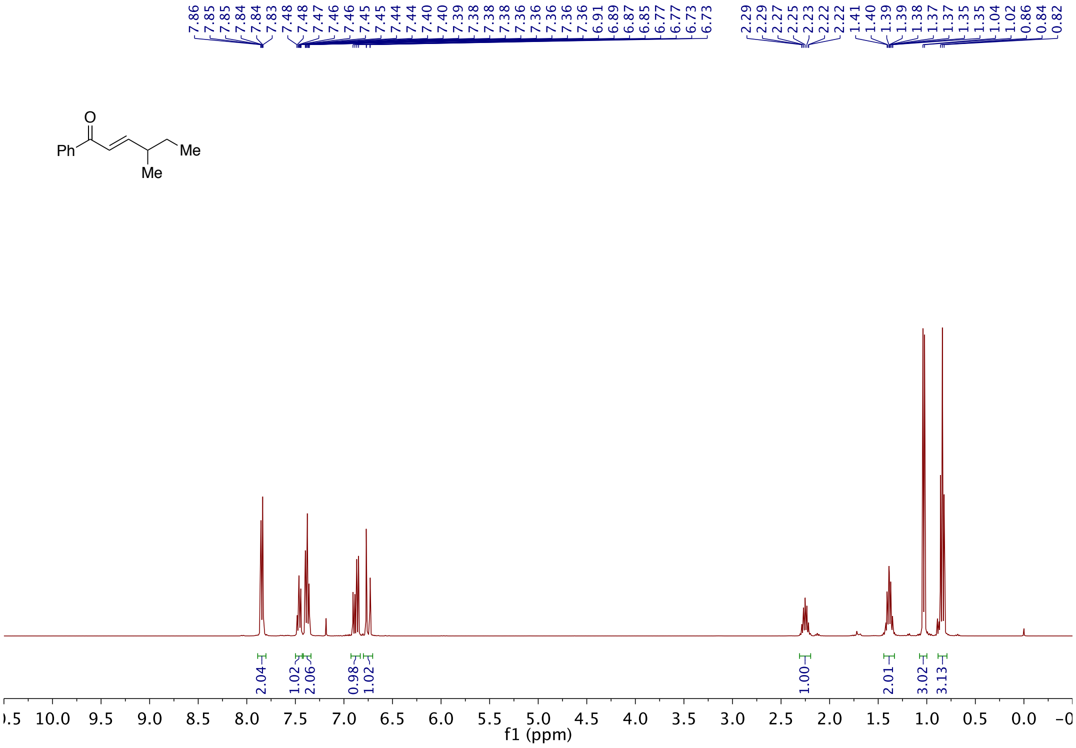

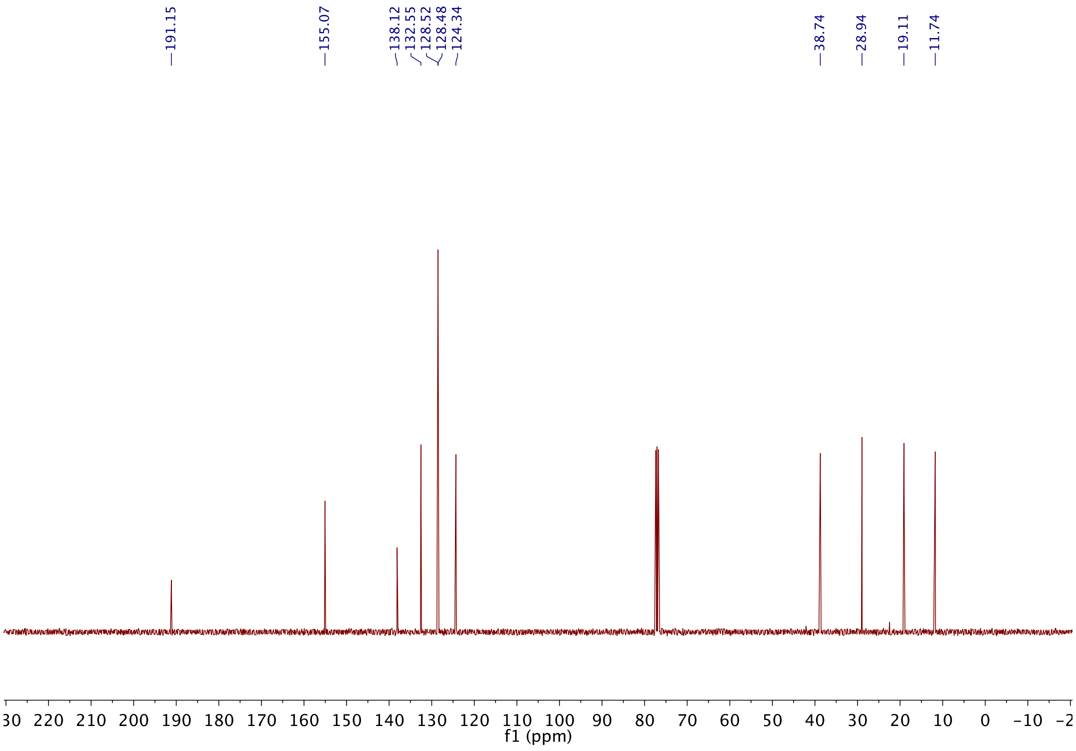

^{1}H and ^{13}C NMR spectra of (*E*)-4-ethyl-1-phenylhex-2-en-1-one (**3j**)

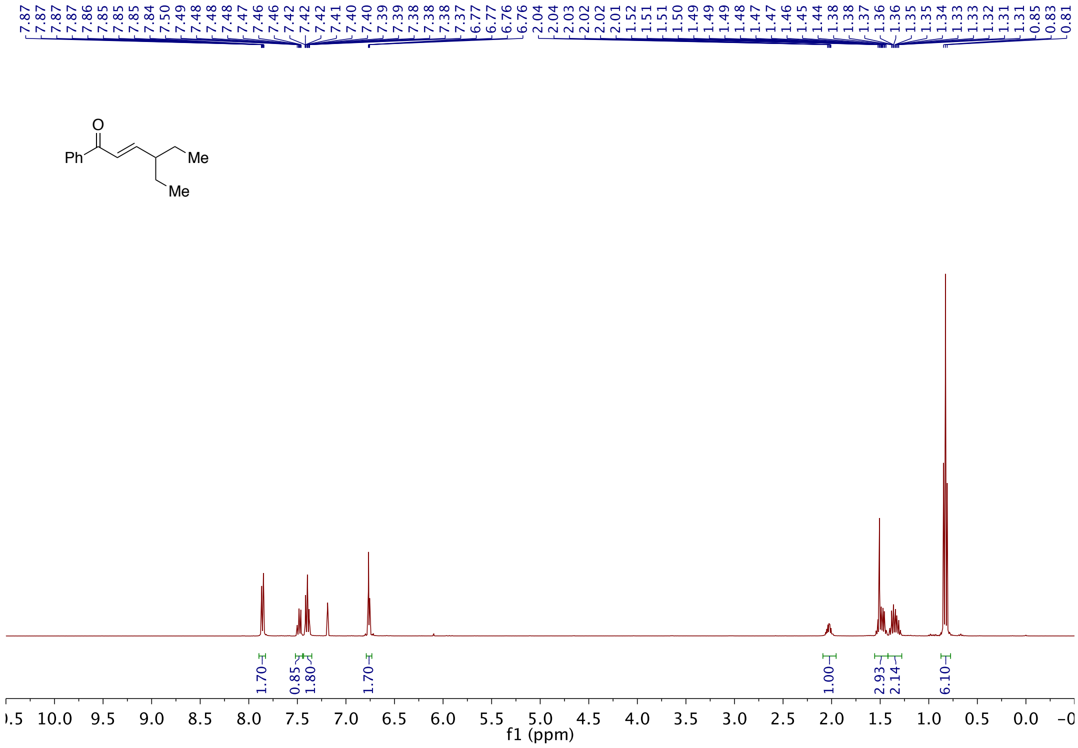

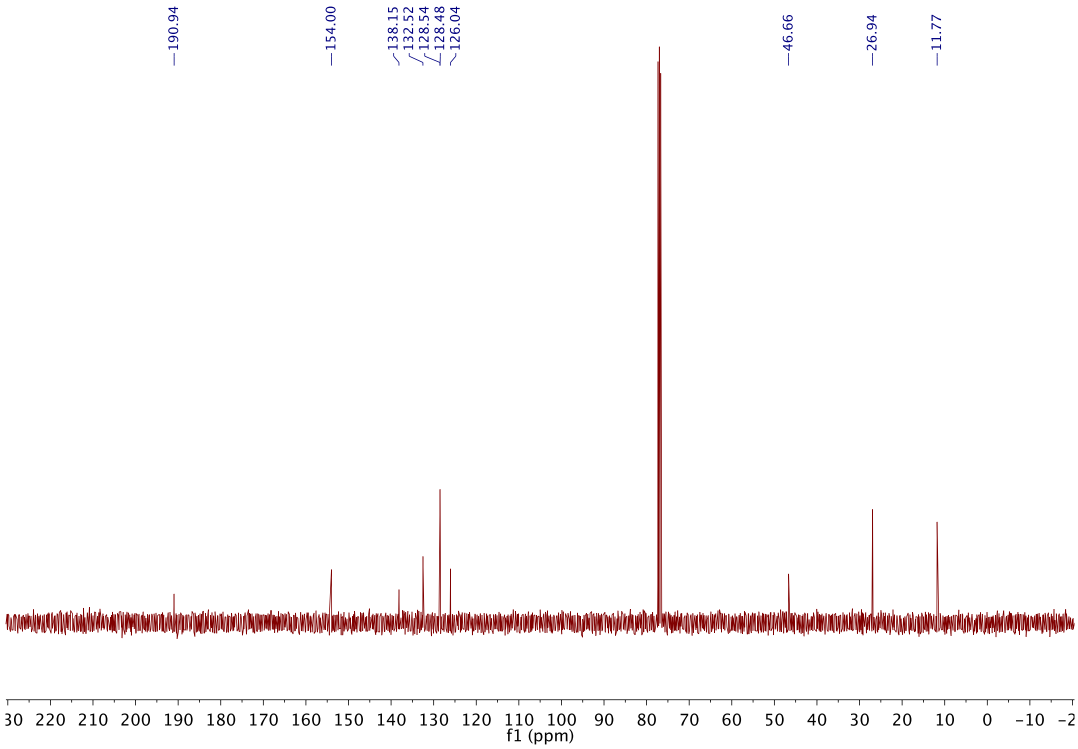

^{1}H and ^{13}C NMR spectra of (*E*)-3-cyclohexyl-1-phenylprop-2-en-1-one (**3k**)

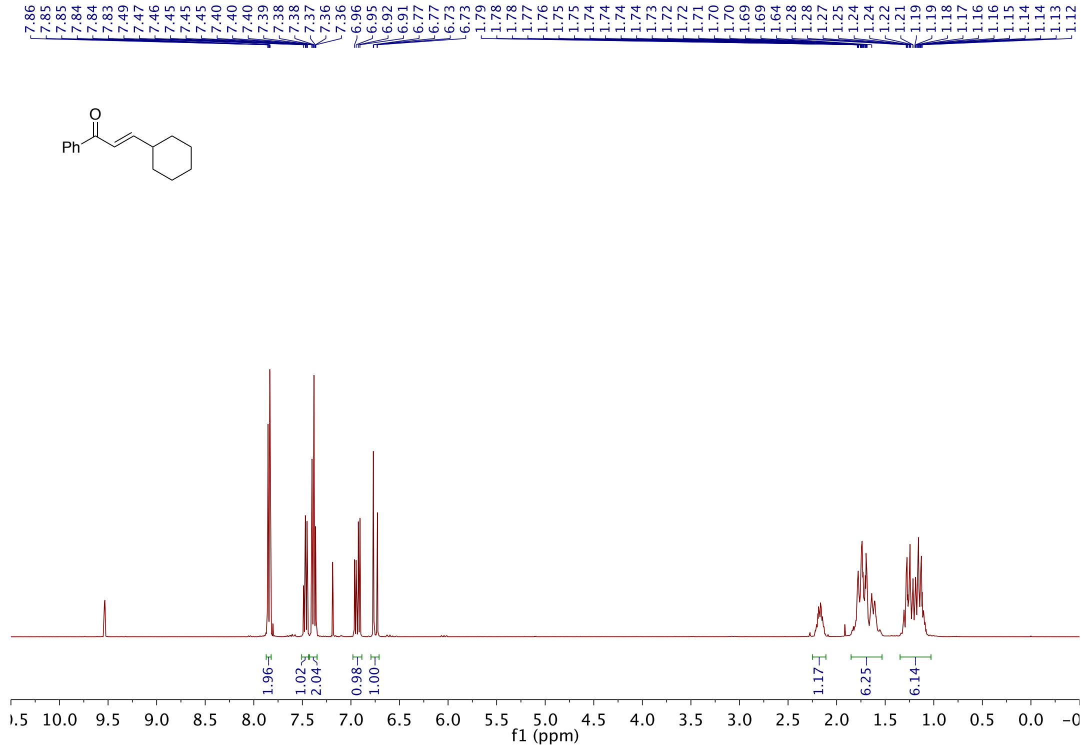

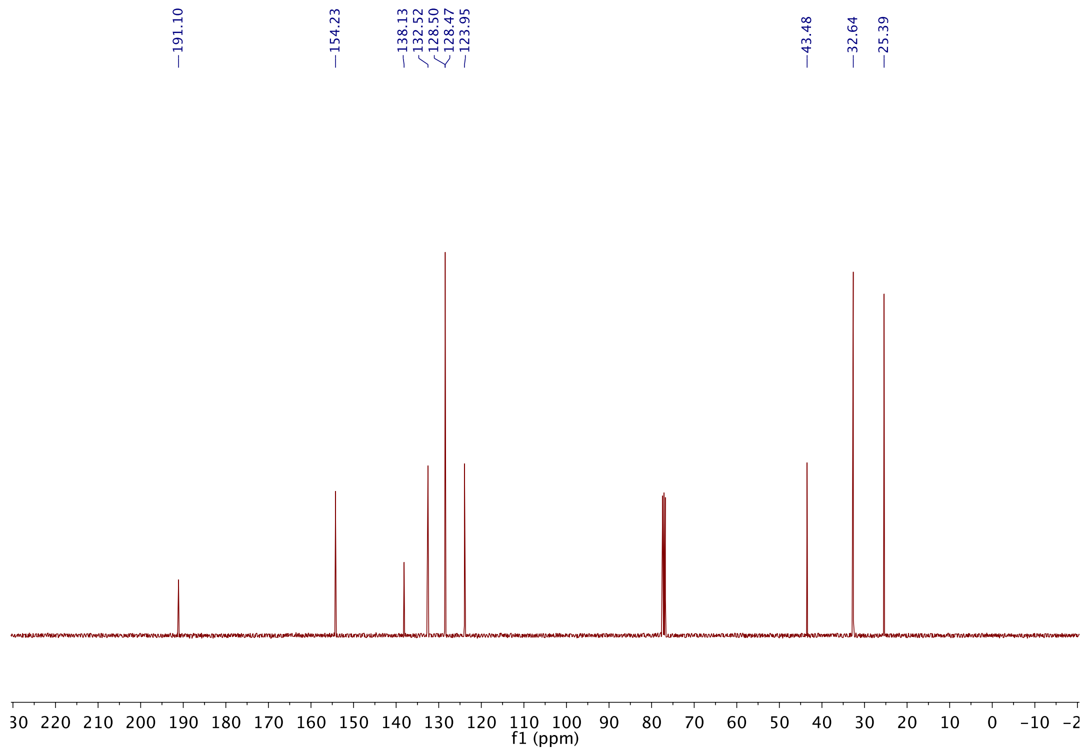

^{1}H and ^{13}C NMR spectra of 4-ethyl-*N*-methoxy-*N*-methylbenzamide (**5f**)

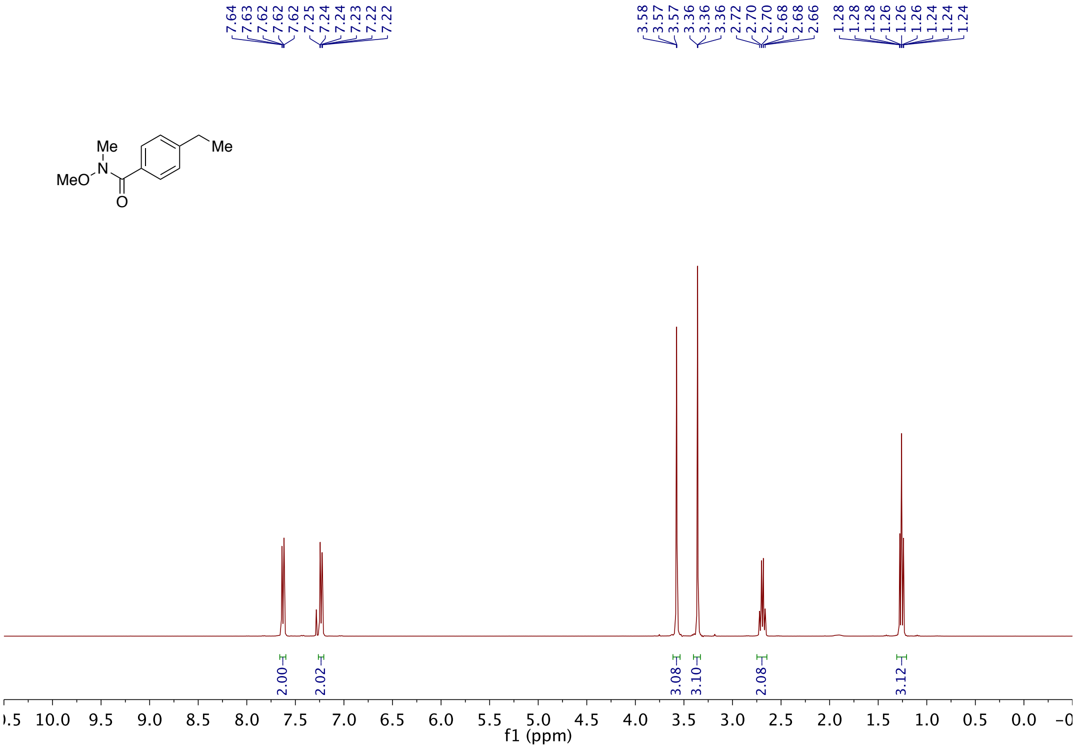

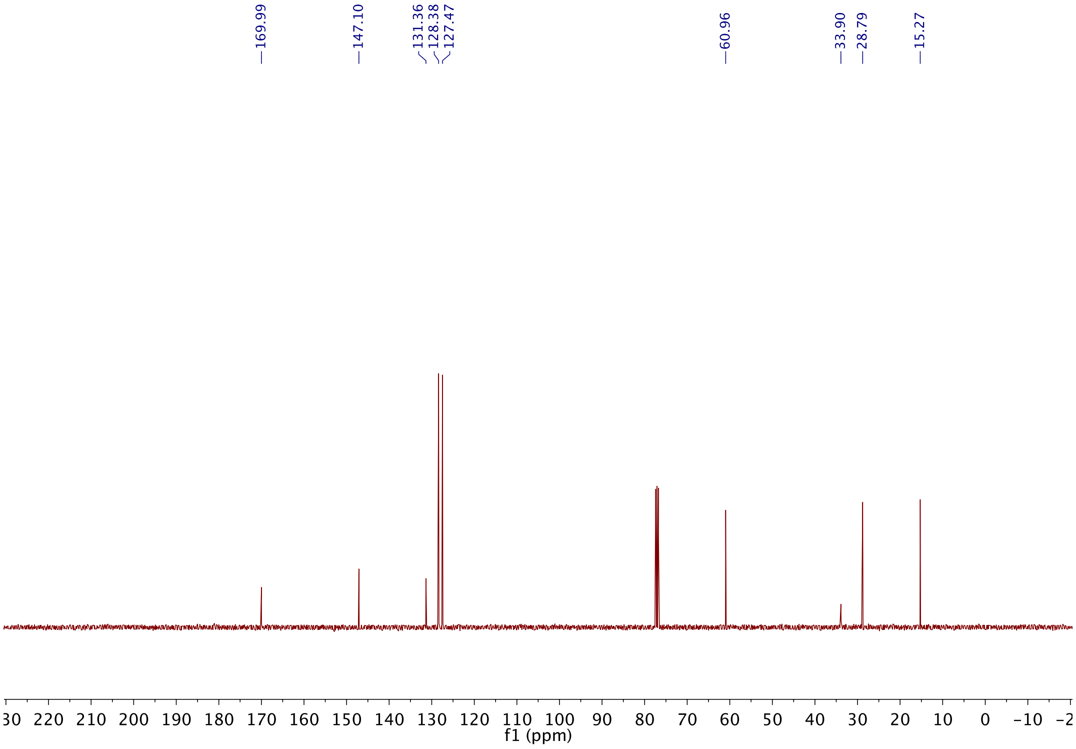

^{1}H and ^{13}C NMR spectra of 1-(4-butylphenyl)pentan-1-one (**5h**)

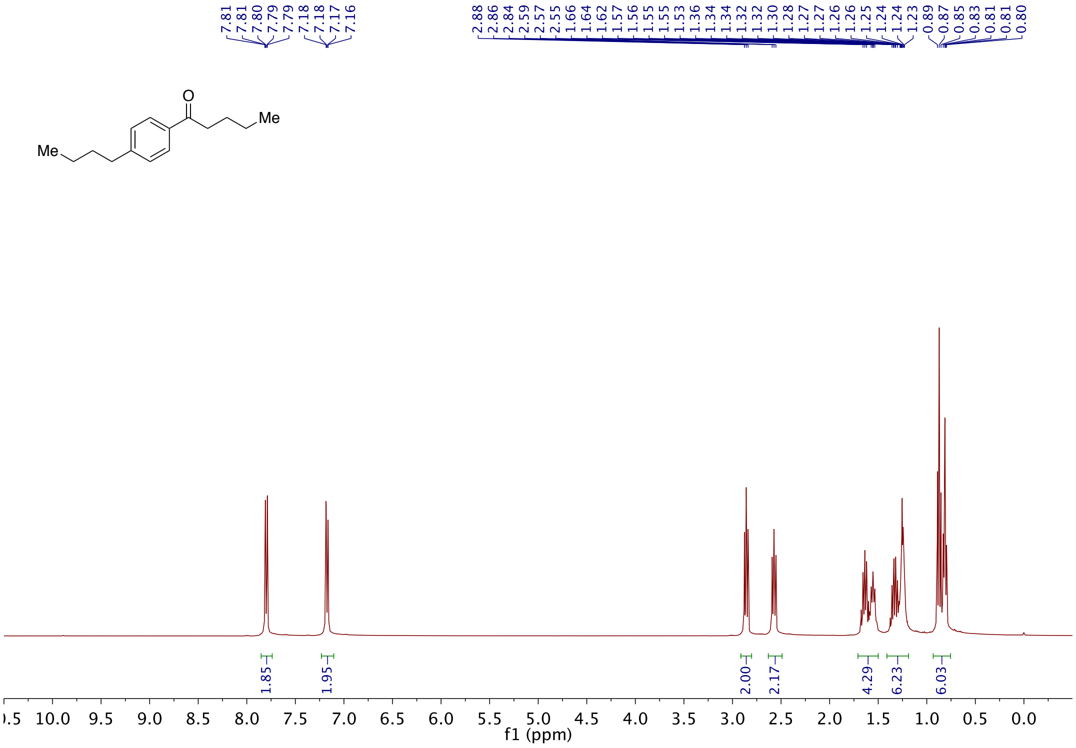

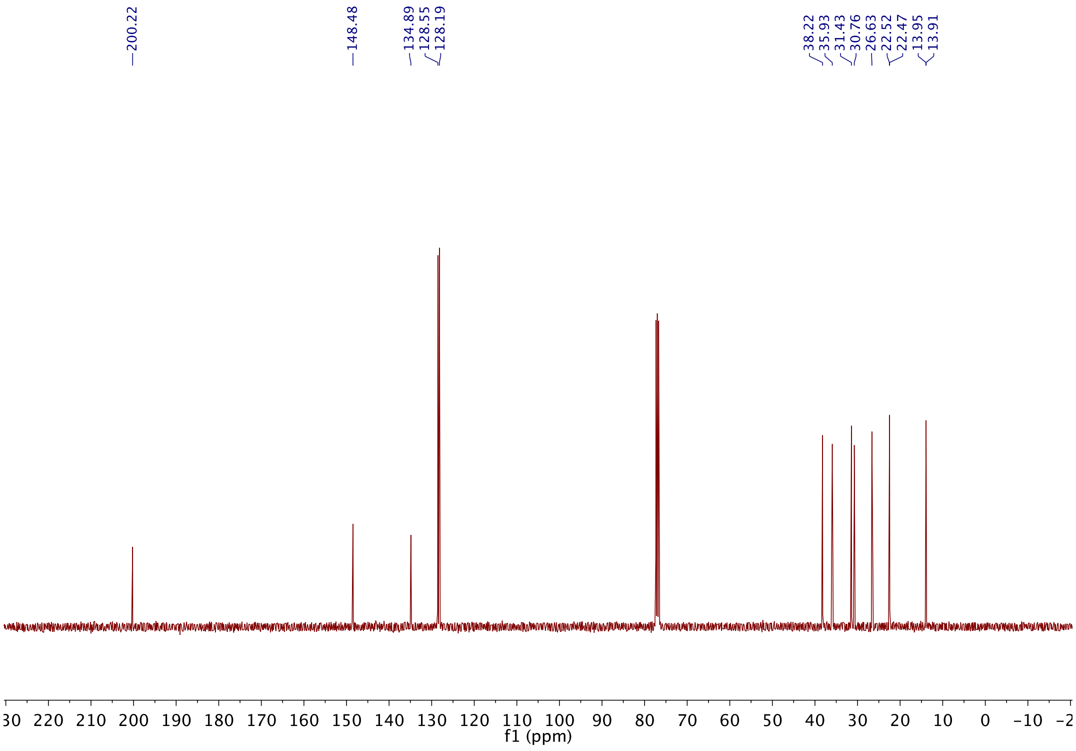

^{1}H and ^{13}C NMR spectra of methyl 4-butylbenzoate (**5i**)

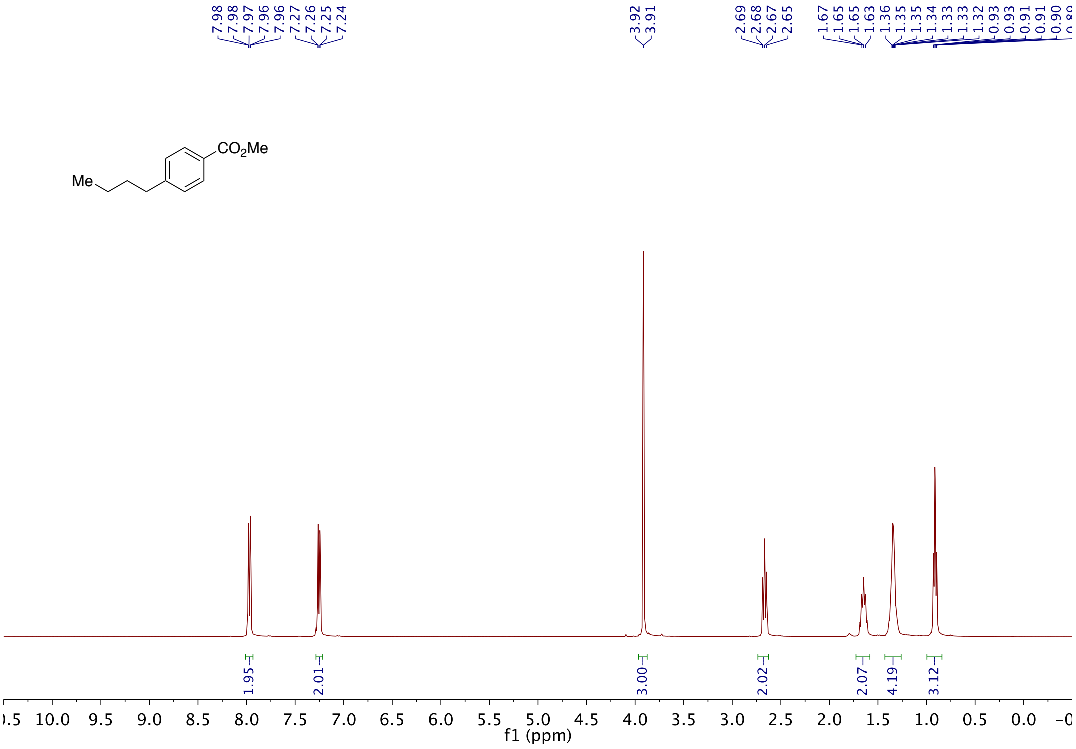

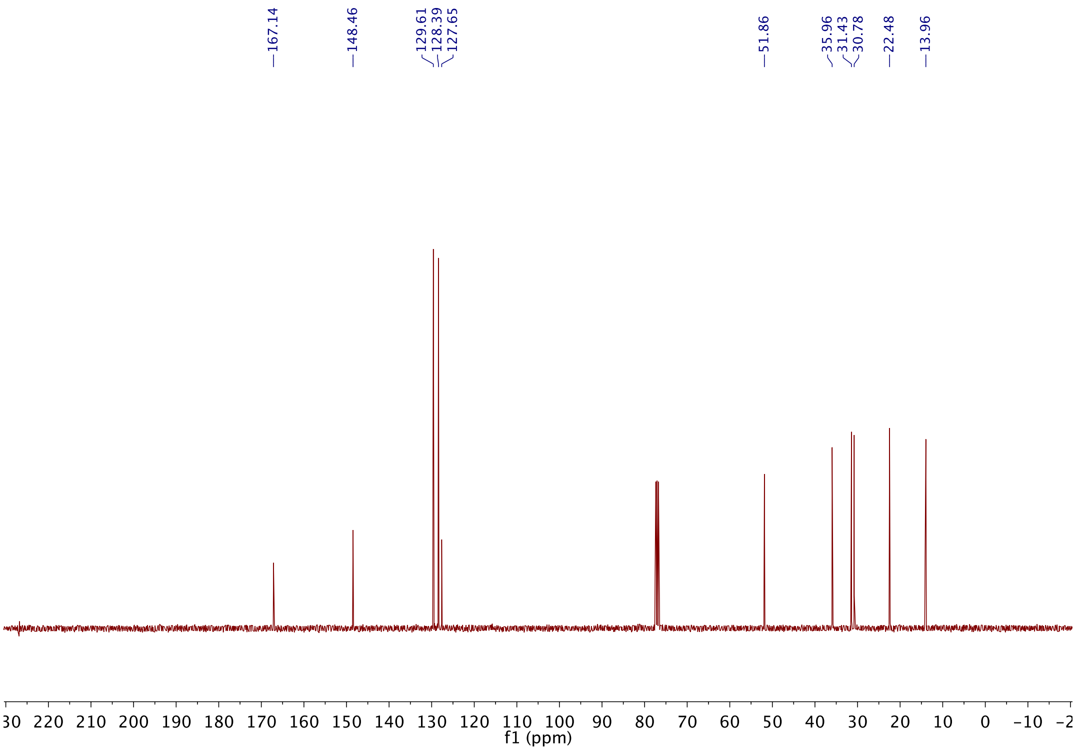

^{1}H and ^{13}C NMR spectra of 4-butyl-*N*-methoxy-*N*-methylbenzamide (**5j**)

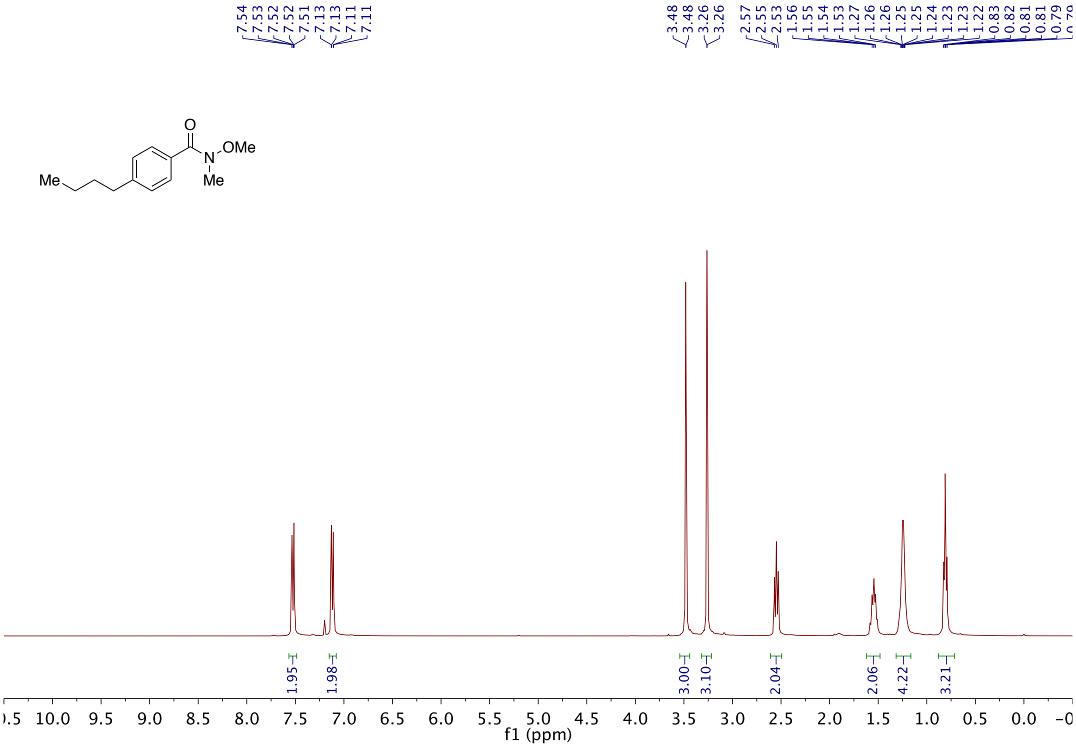

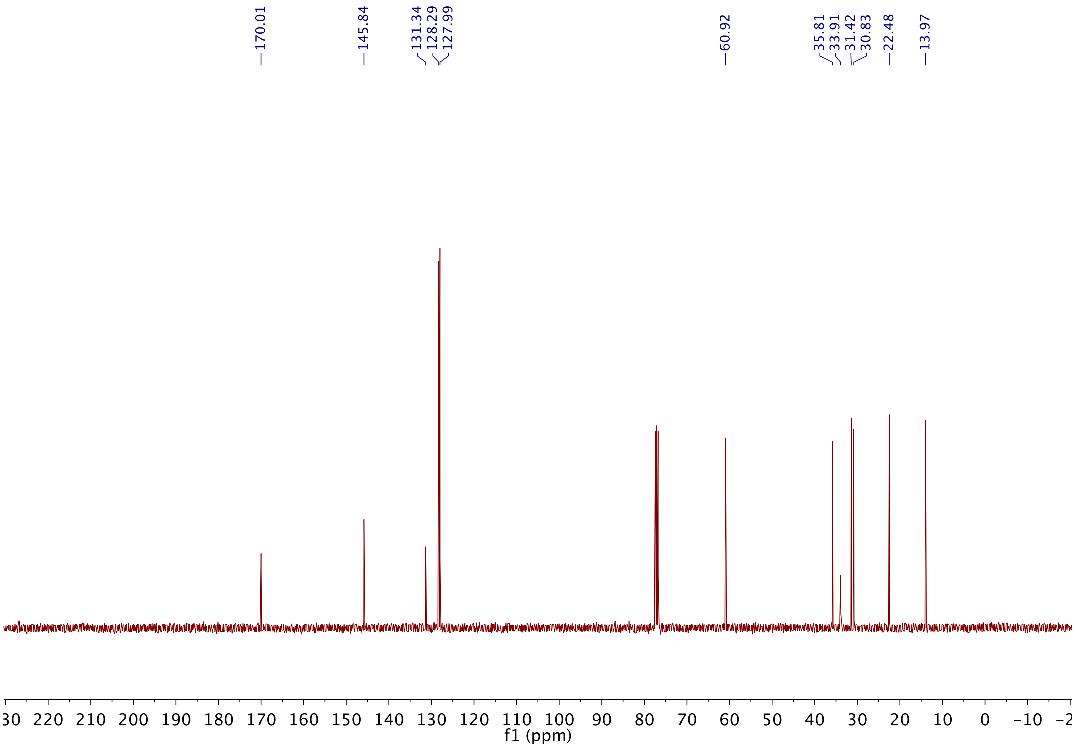

^{1}H and ^{13}C NMR spectra of 1-(2-chloropyridin-4-yl)-4-methylpentan-1-one (**S8**)

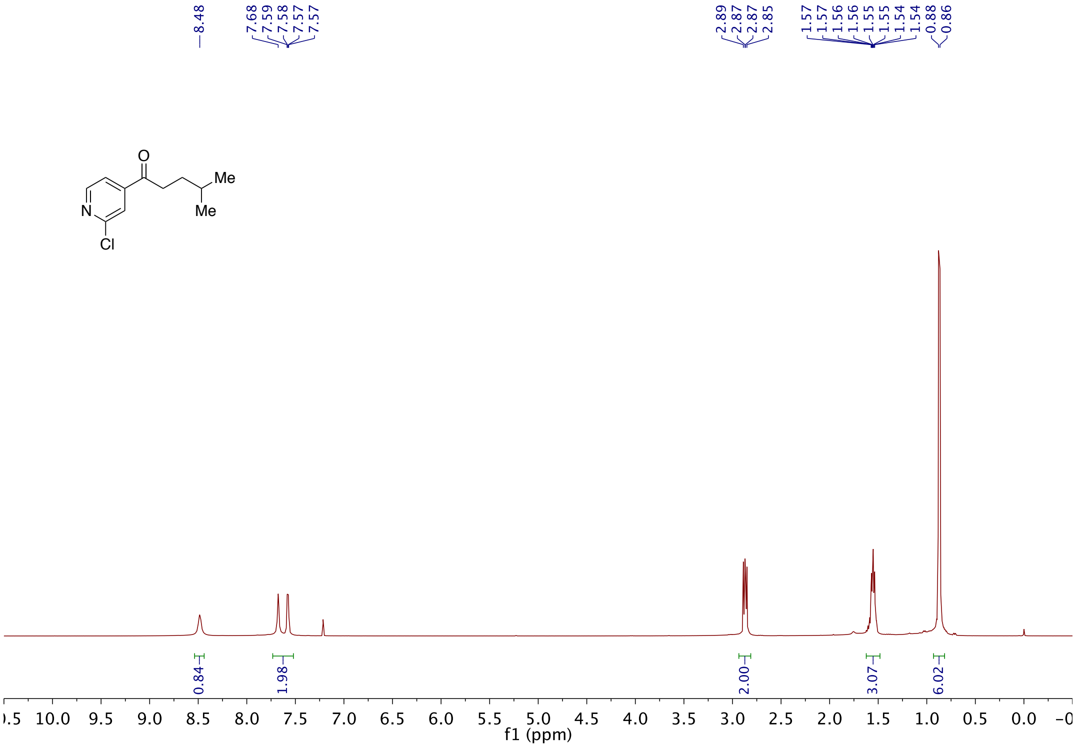

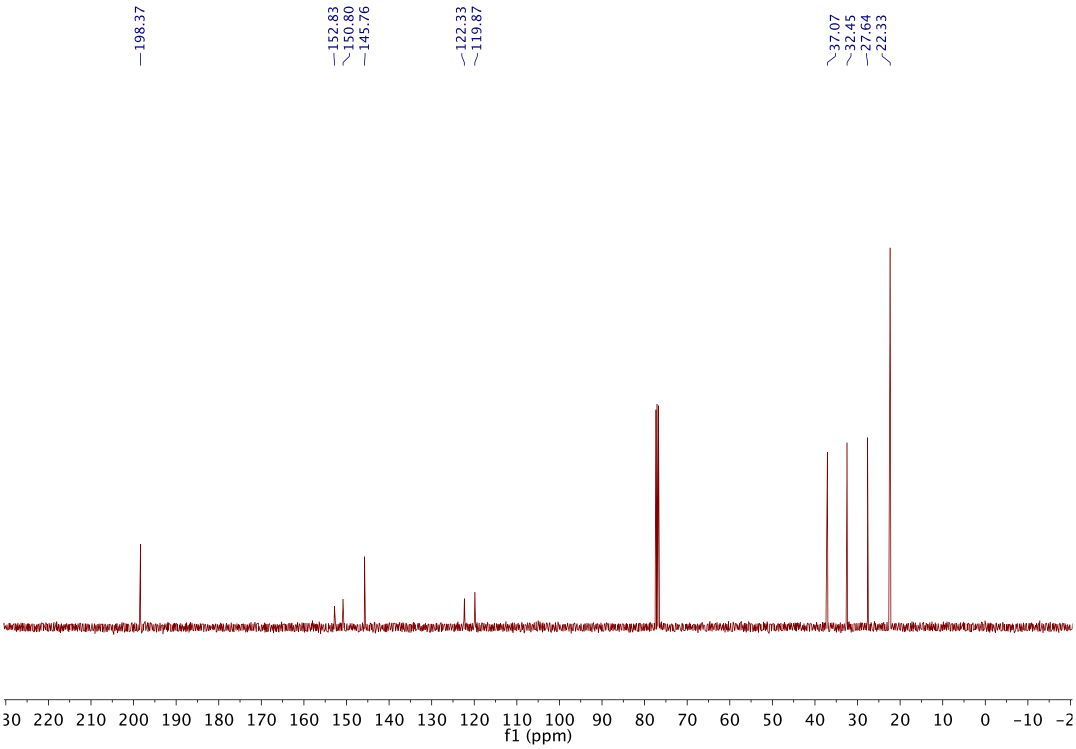

^{1}H and ^{13}C NMR spectra of methyl 2-(1,3-dioxoisoindolin-2-yl)-4-methylpentanoate (**S9**)

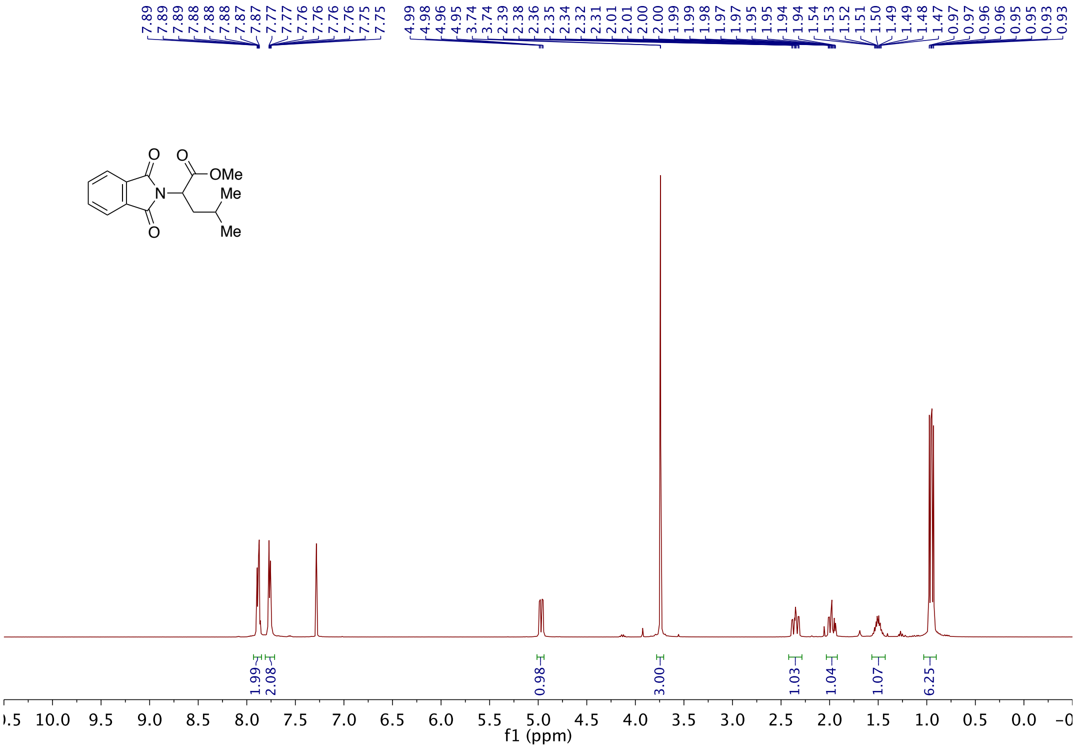

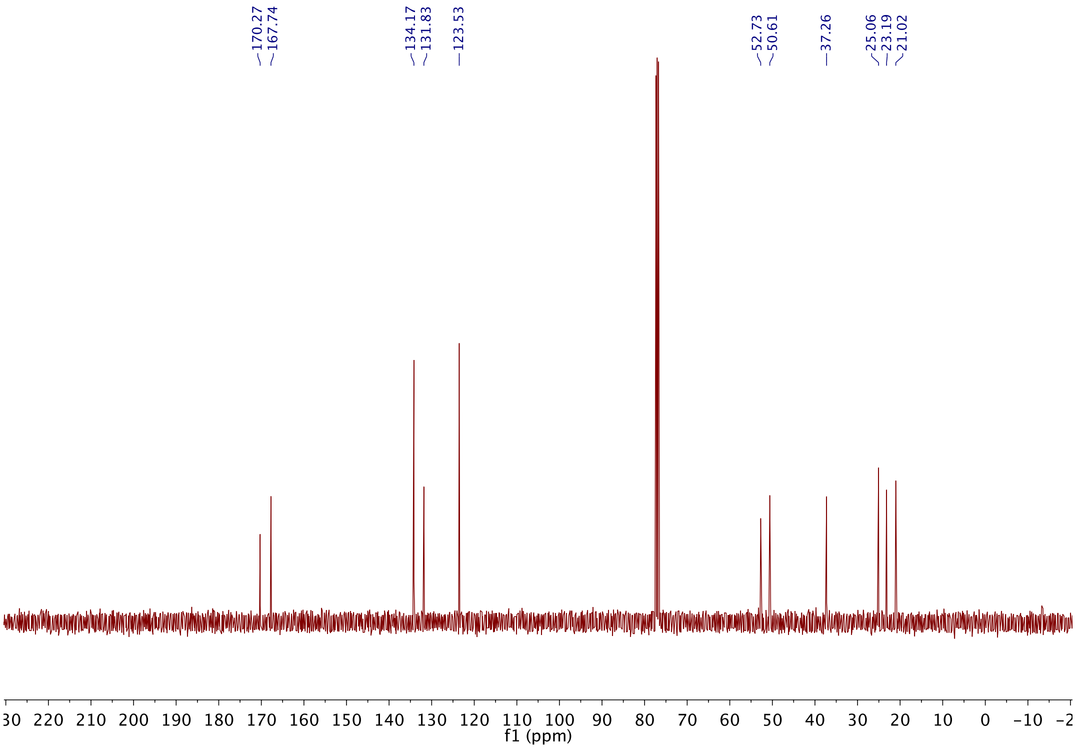

^{1}H and ^{13}C NMR spectra of methyl 2-(4-isobutylphenyl)propanoate (**S10**)

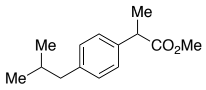

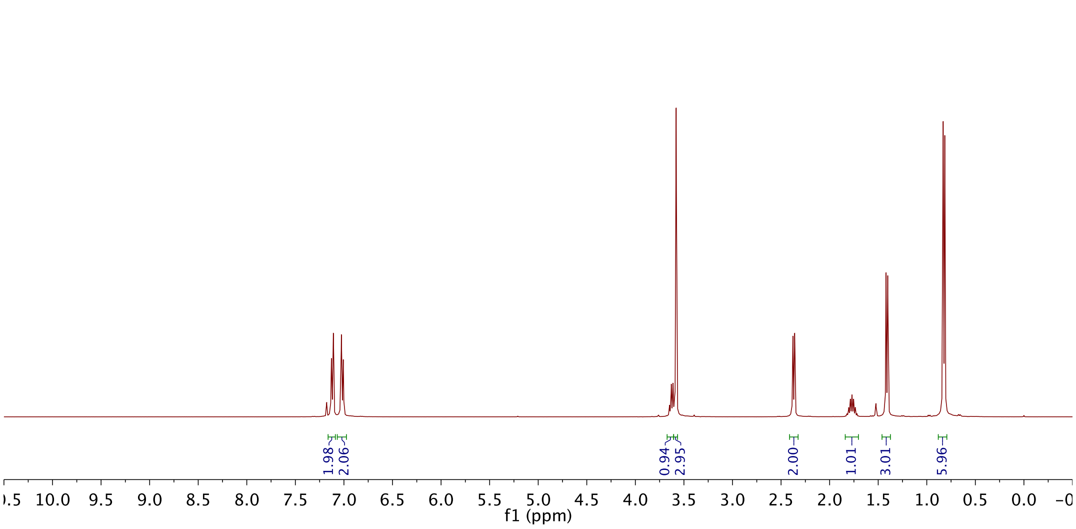

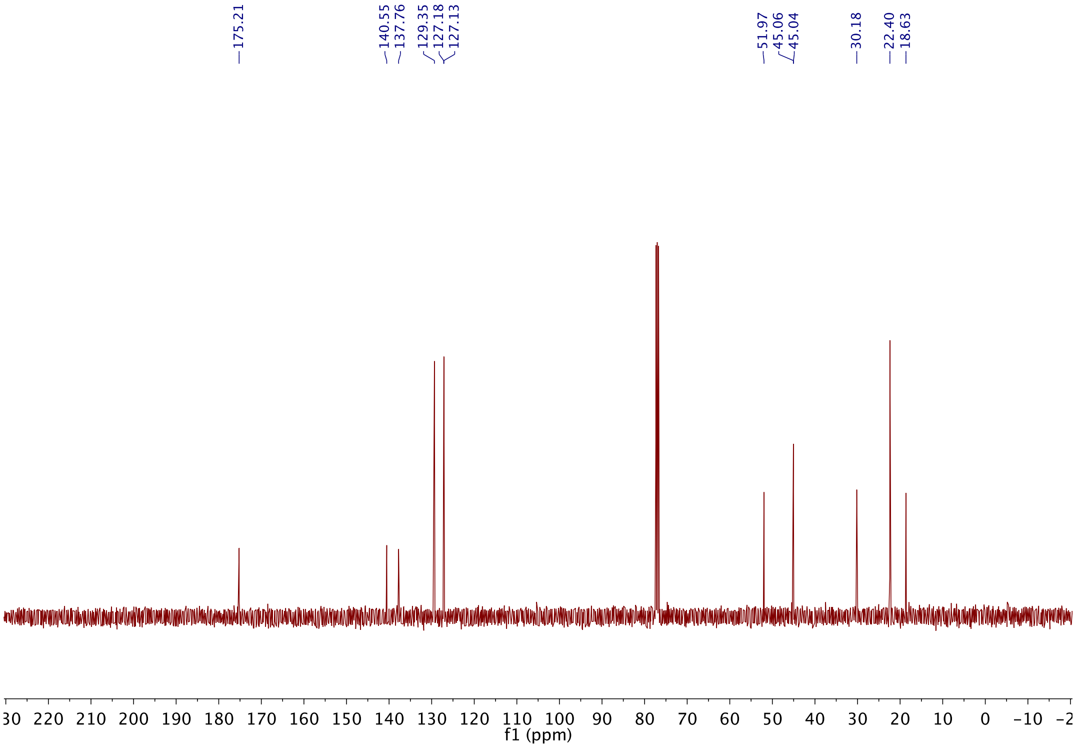

^{1}H and ^{13}C NMR spectra of methyl 4'-hexyl-[1,1'-biphenyl]-4-carboxylate (**S11**)

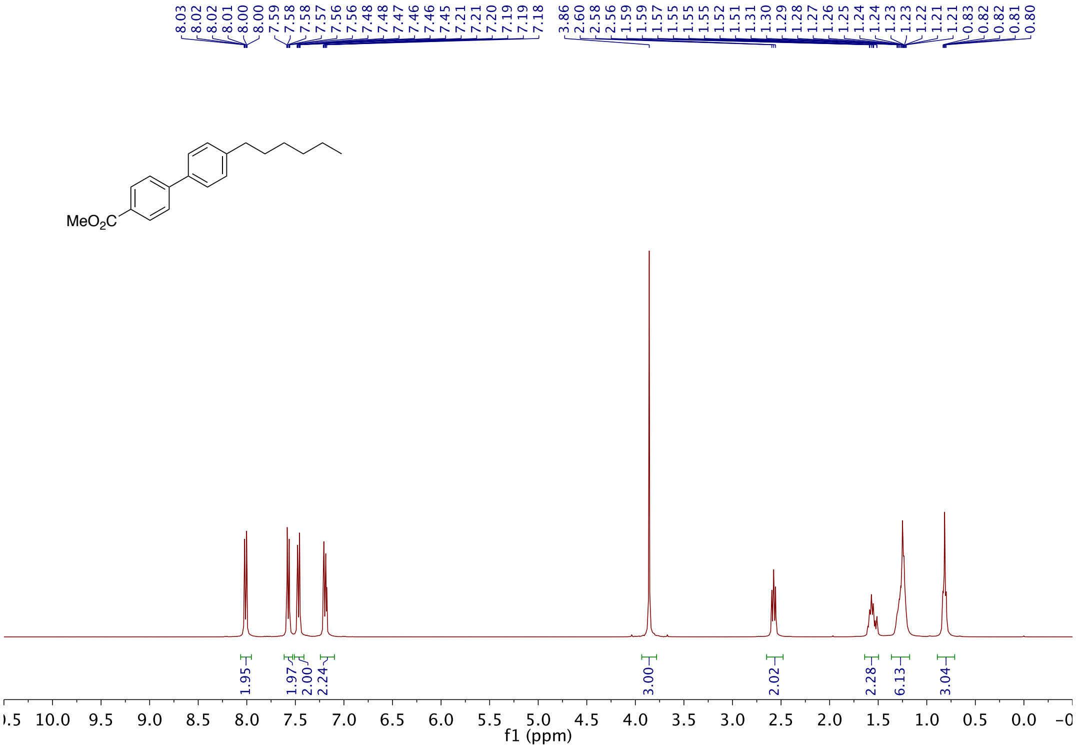

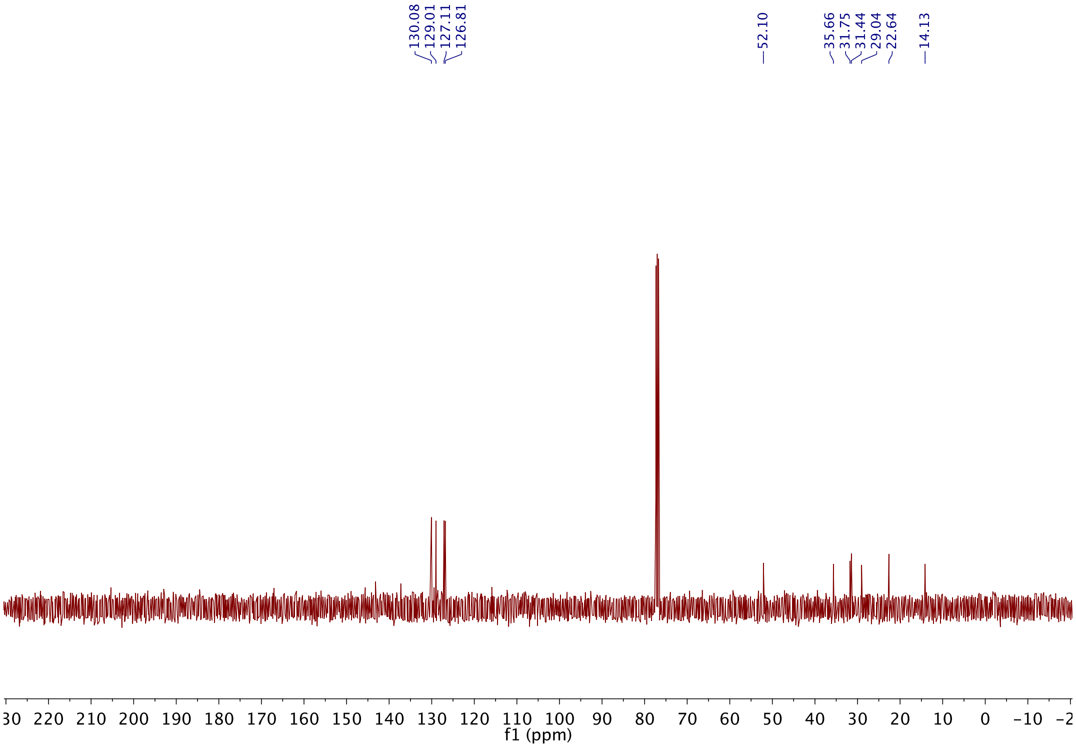

^{1}H and ^{13}C NMR spectra of 4-ethylbenzyl 7,7-dimethyl-3-oxo-2-oxabicyclo[2.2.1]heptane-6-carboxylate (**S12**)

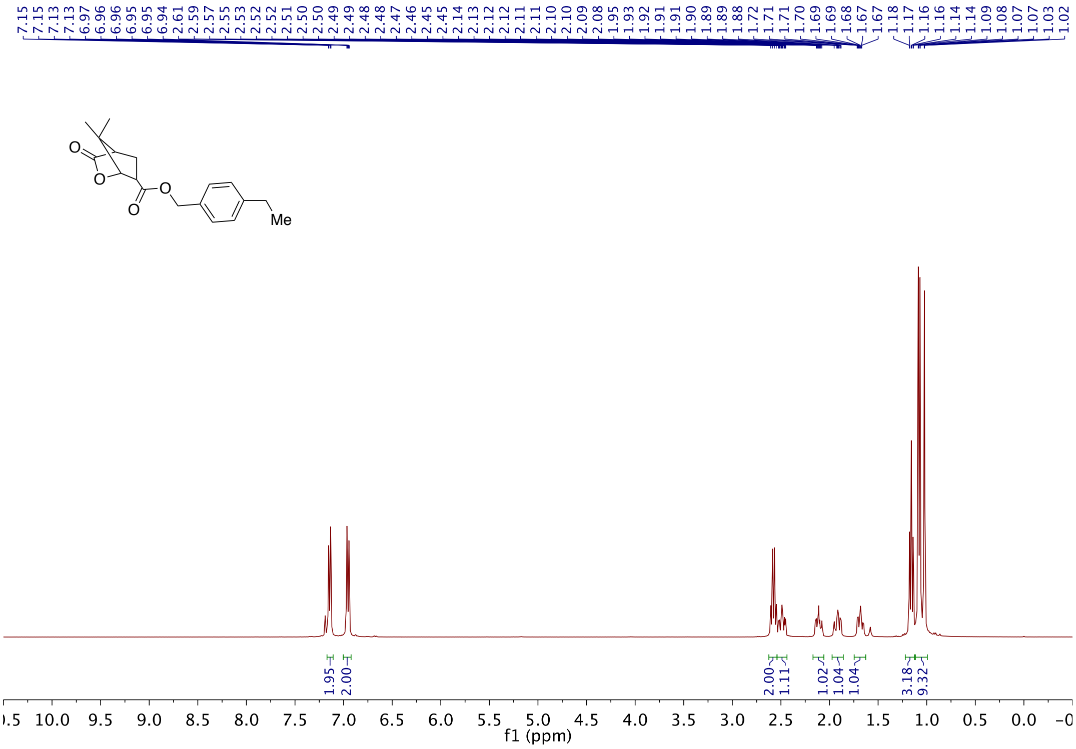

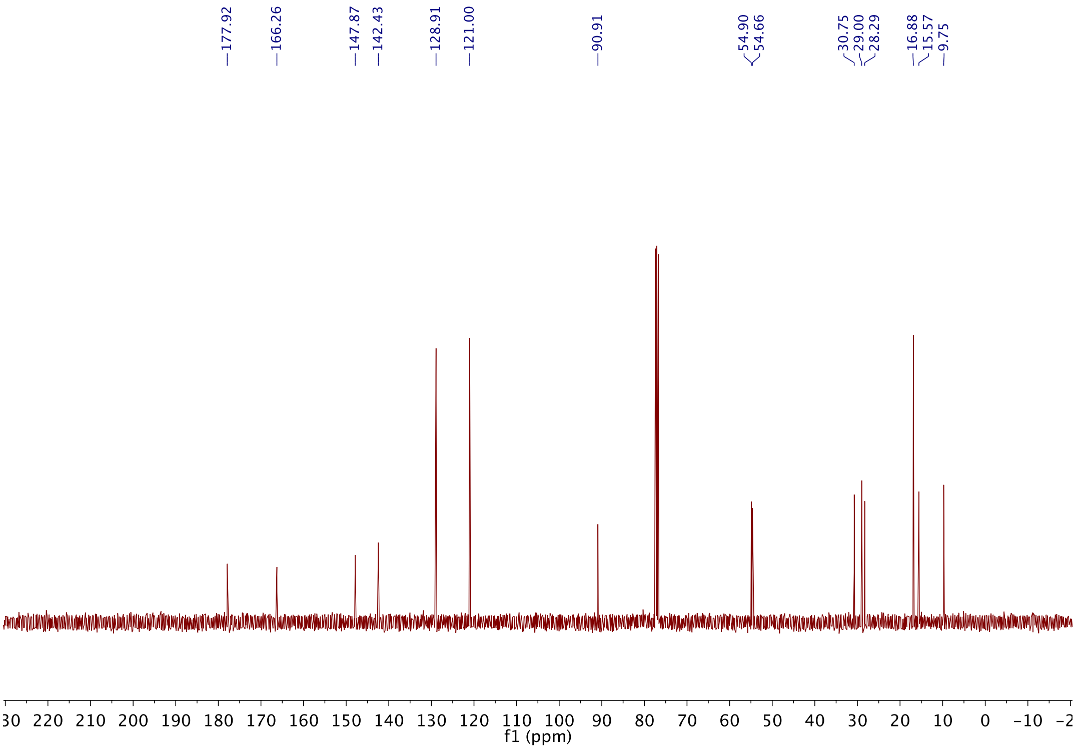

^{1}H and ^{13}C NMR spectra of 4-ethylphenyl 2-(4-(4-chlorobenzoyl)phenoxy)-2-methylpropanoate (**S13**)

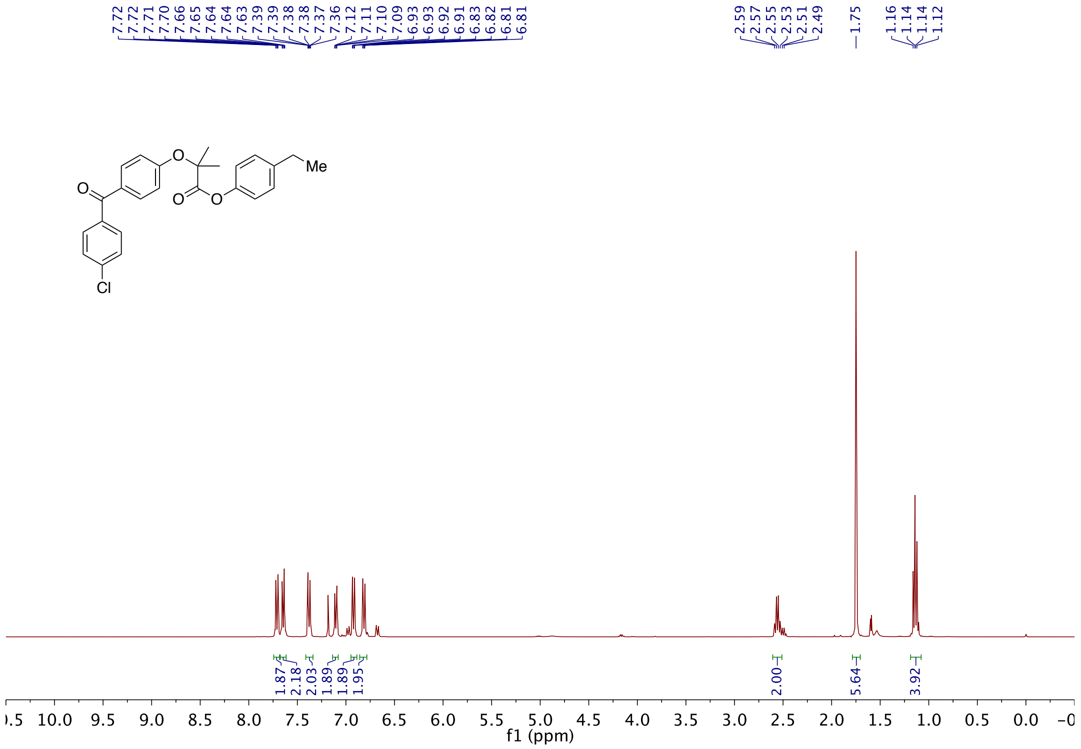

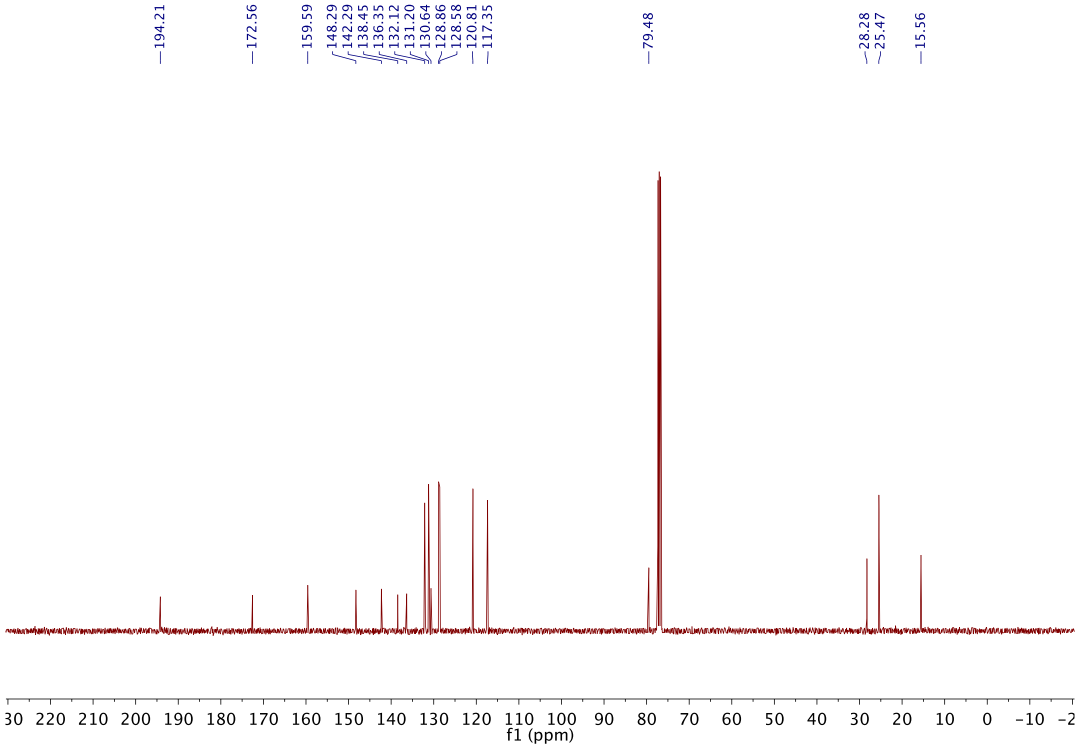

^{1}H and ^{13}C NMR spectra of (*E*)-3-cyclopropyl-1-phenylprop-2-en-1-one (**3l**)

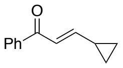

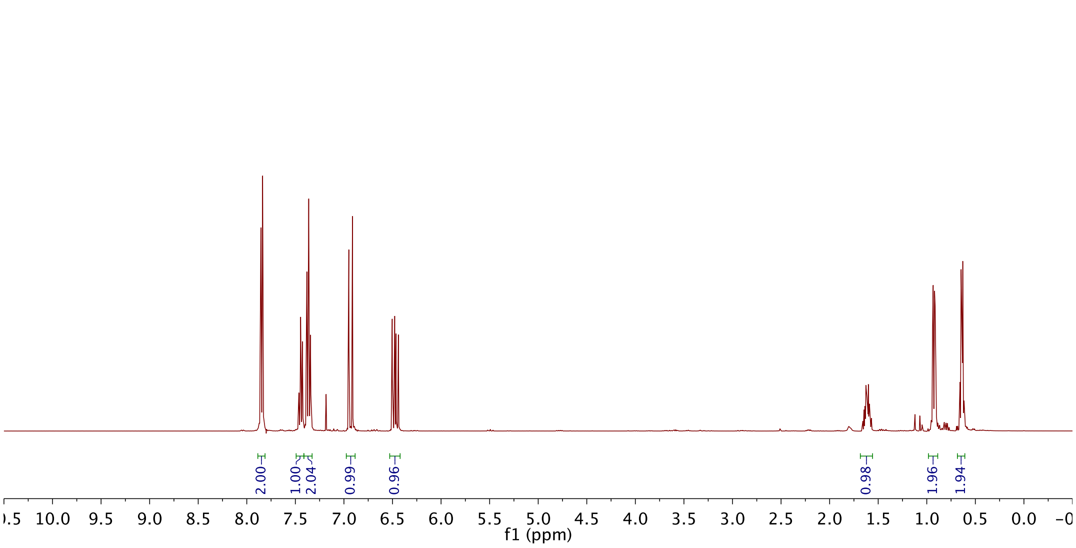

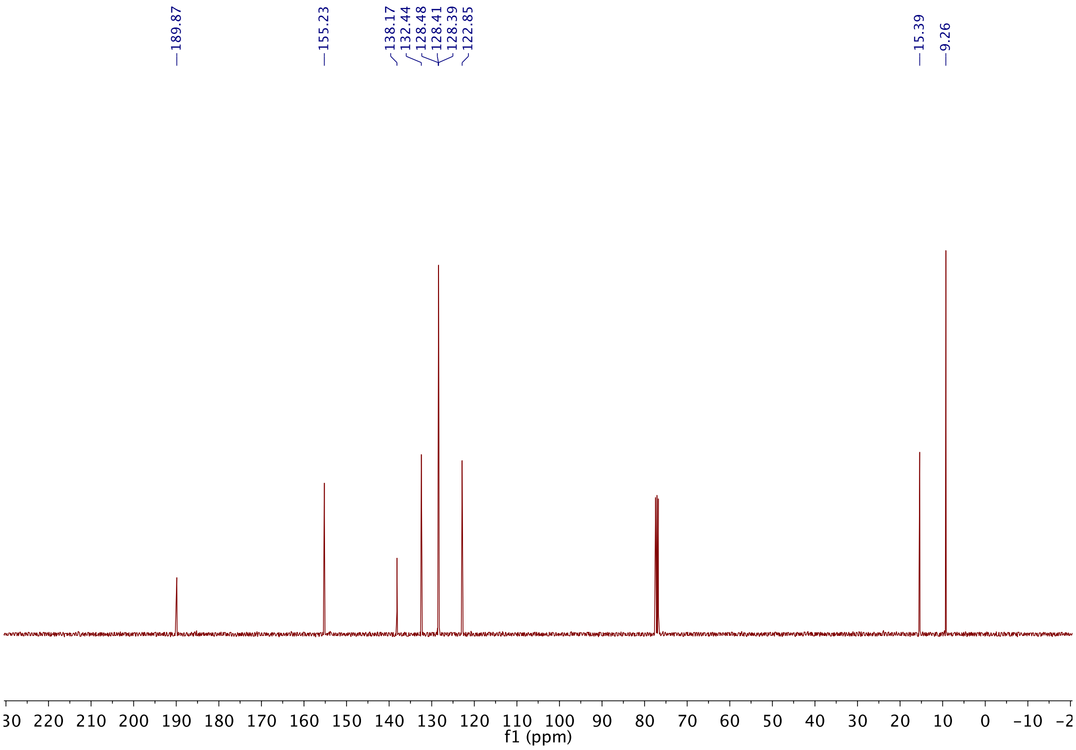

^{1}H and ^{13}C NMR spectra of 1-phenylpentan-1-one-2,2-*d*2 (d_2-**1s**)

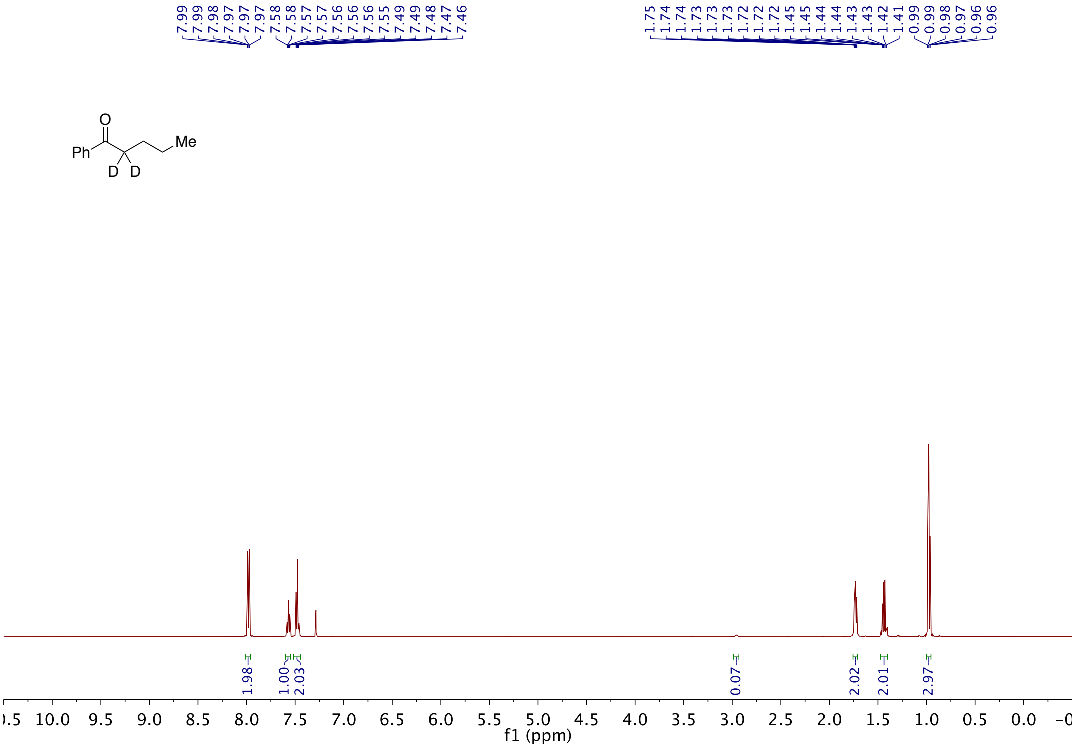

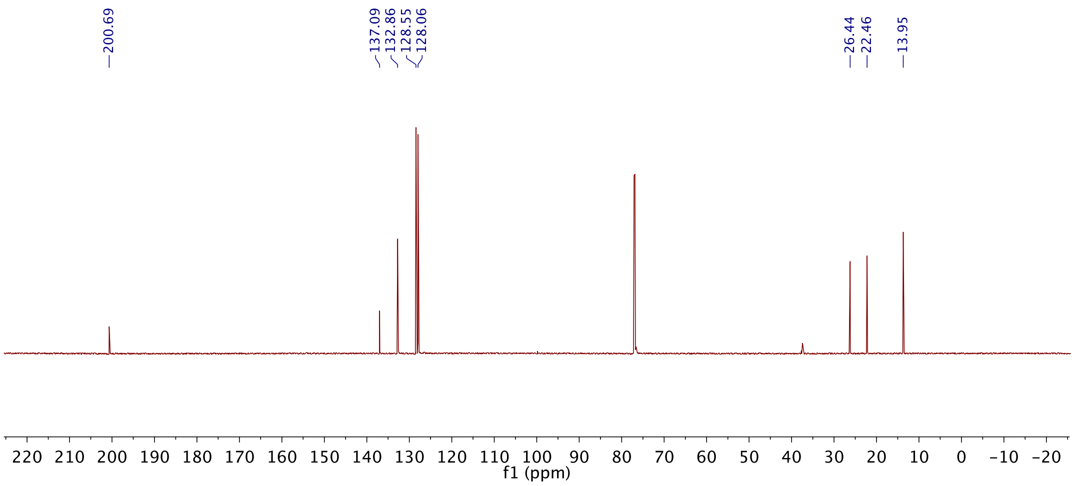

^{1}H and ^{13}C NMR spectra of 1-(4-(methyl-*d*3)phenyl)ethan-1-one (*d*$_3$-**5c**)

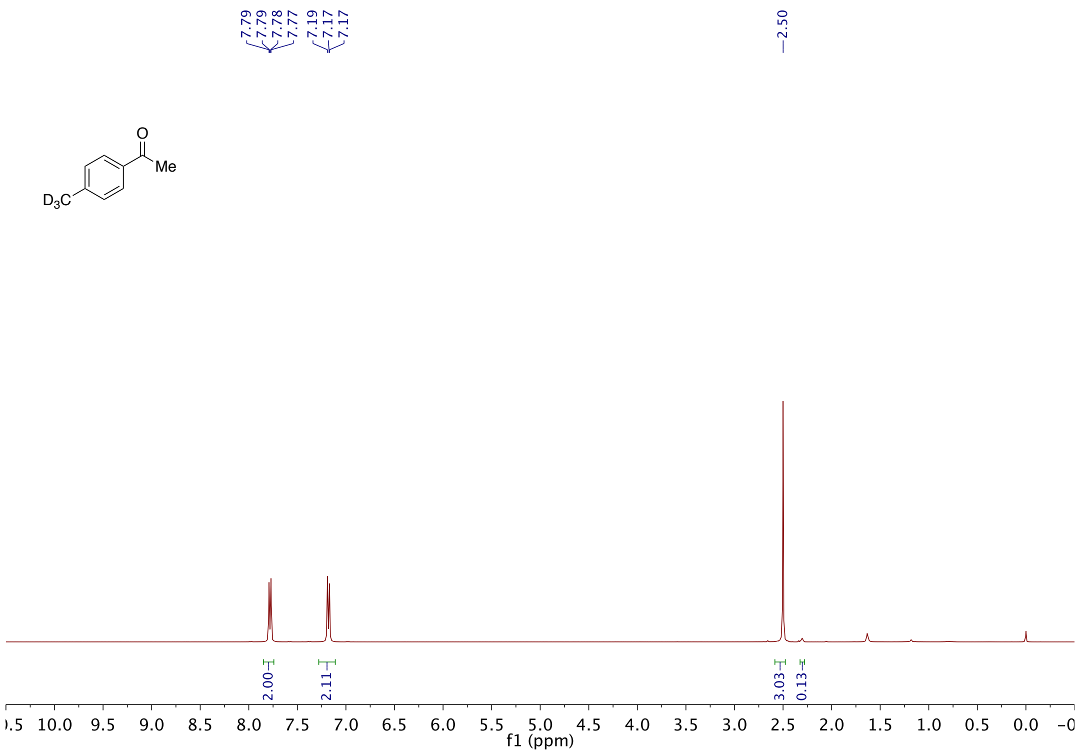

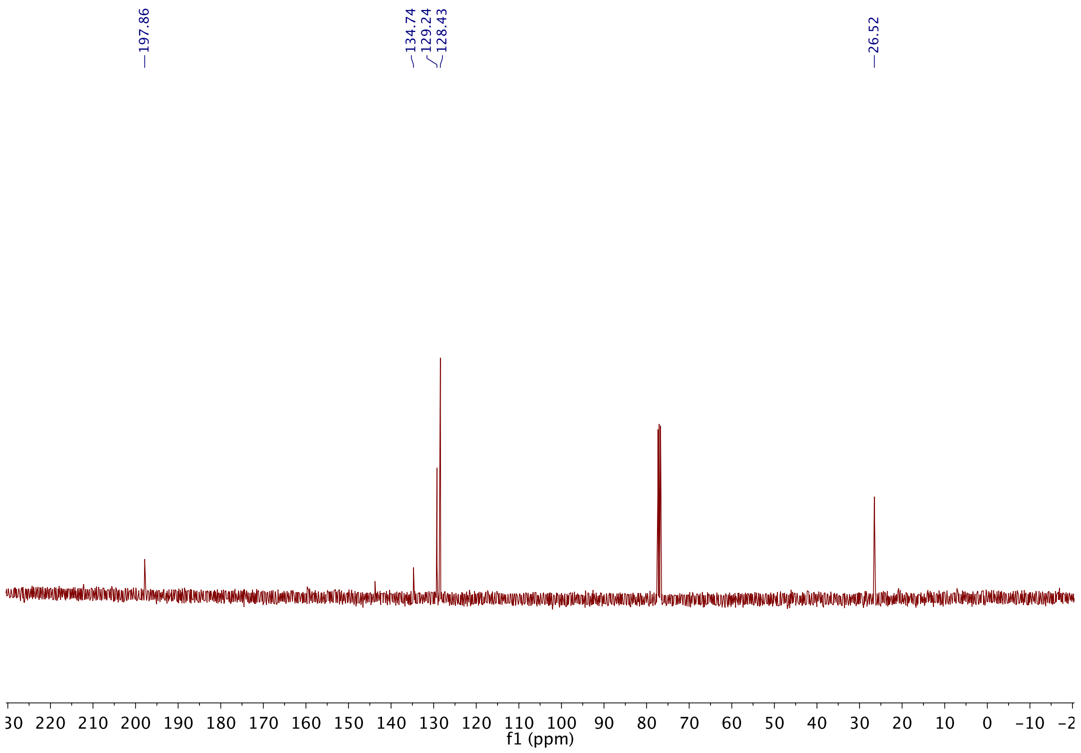

^{1}H and ^{13}C NMR spectra of 4-chloro-4-methyl-1-phenylpentan-1-one (**2a**)

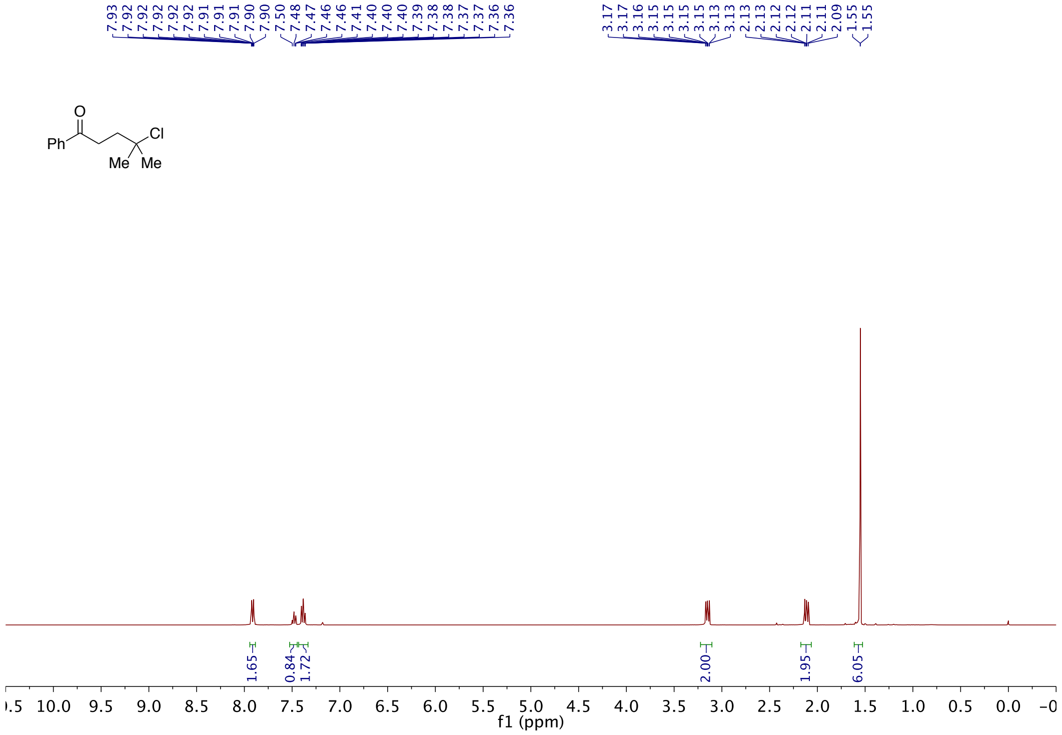

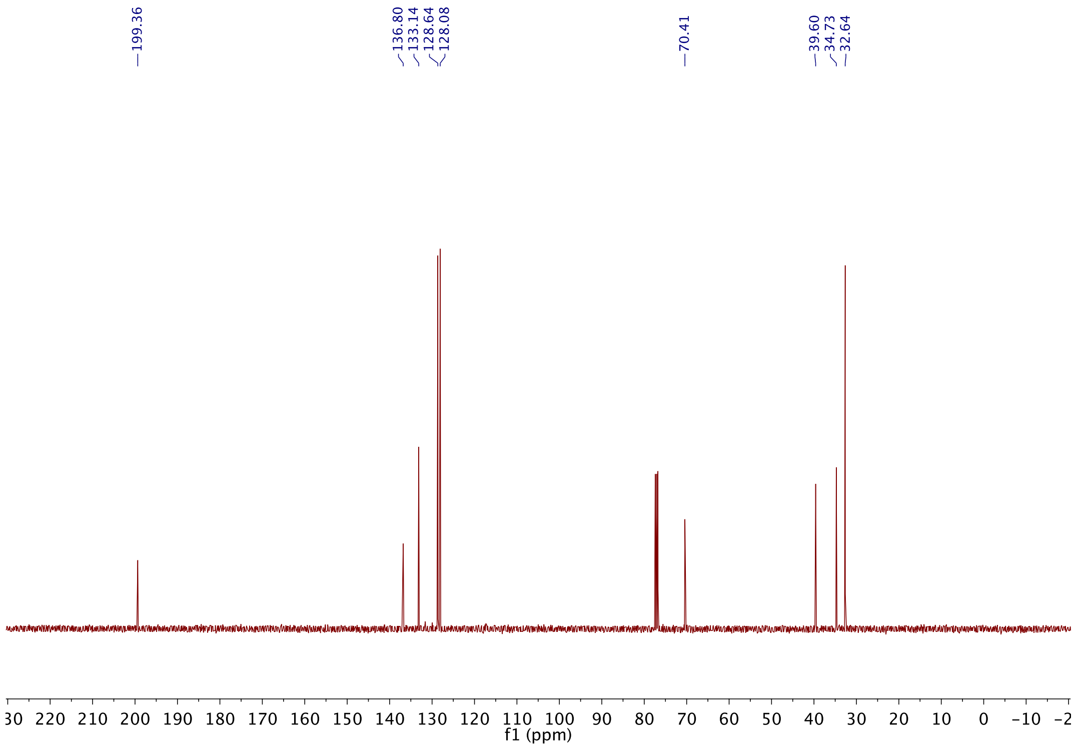

^{1}H and ^{13}C NMR spectra of 1-(4-(*tert*-butyl)phenyl)-4-chloro-4-methylpentan-1-one (**2b**)

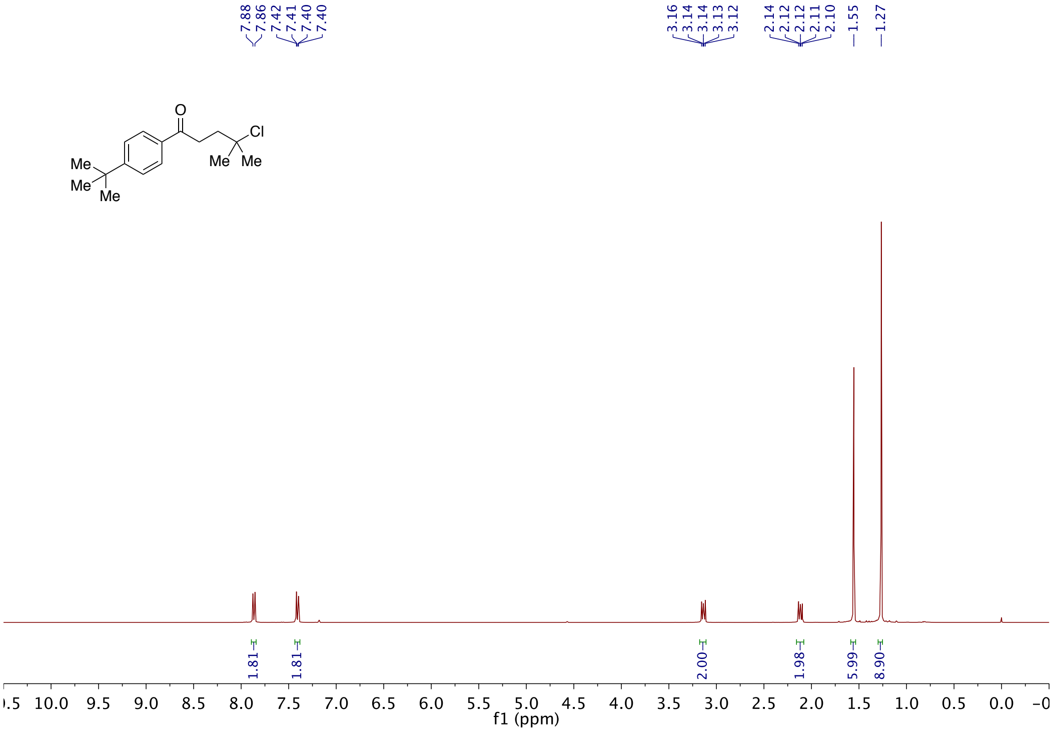

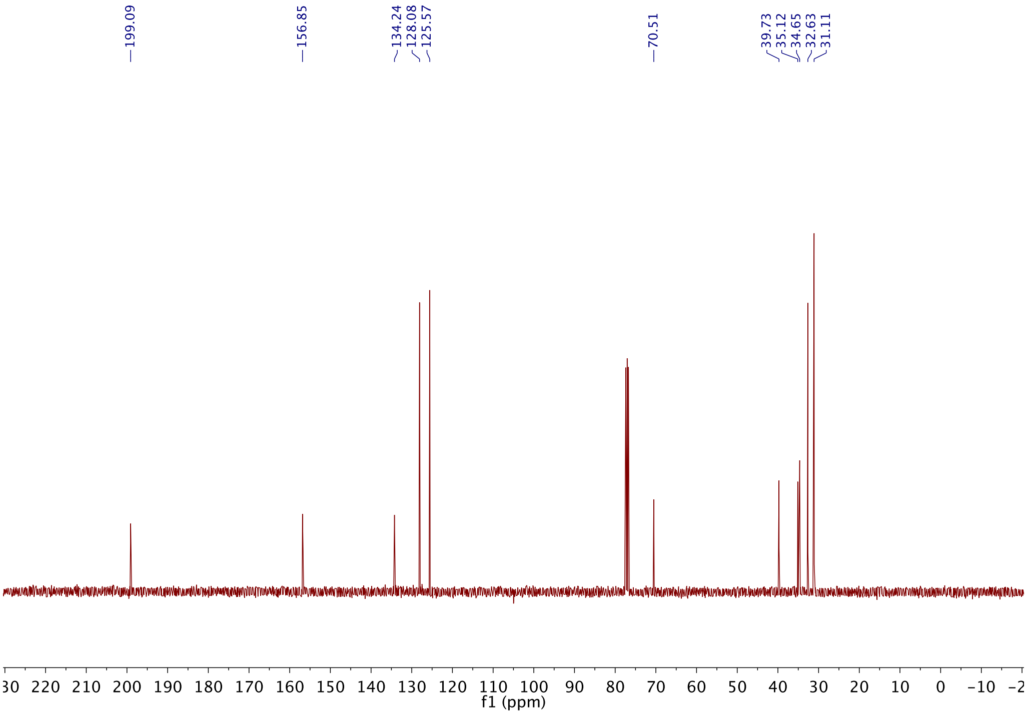

^{1}H and ^{13}C NMR spectra of 4-chloro-1-(4-methoxyphenyl)-4-methylpentan-1-one (**2c**)

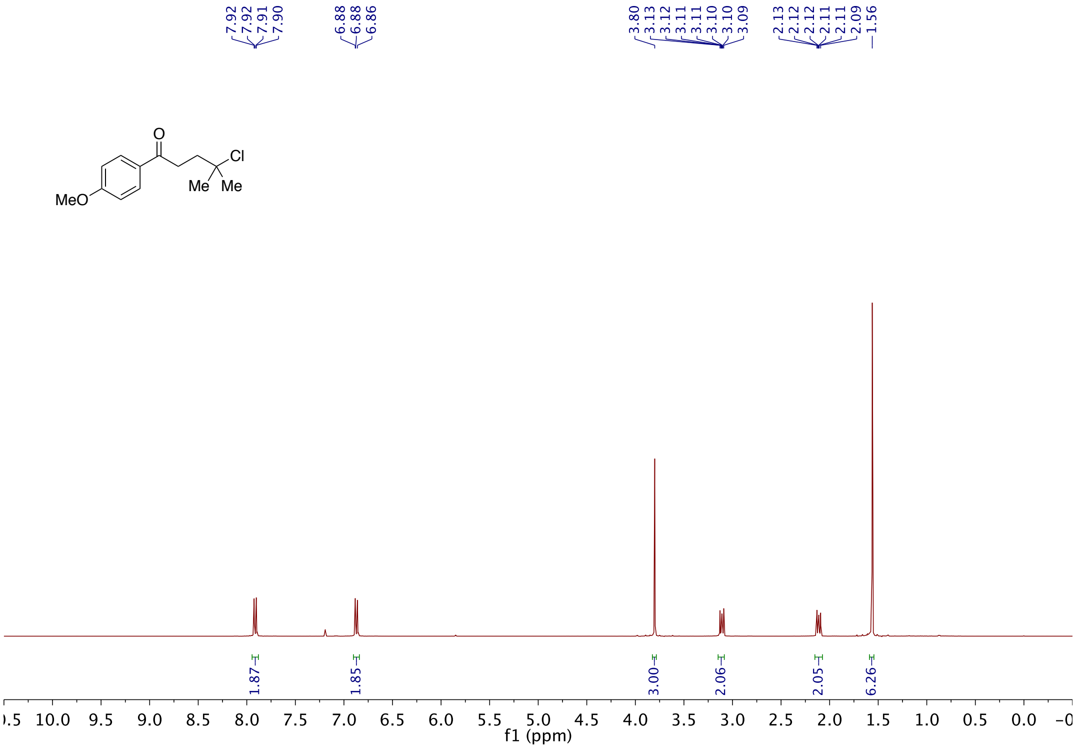

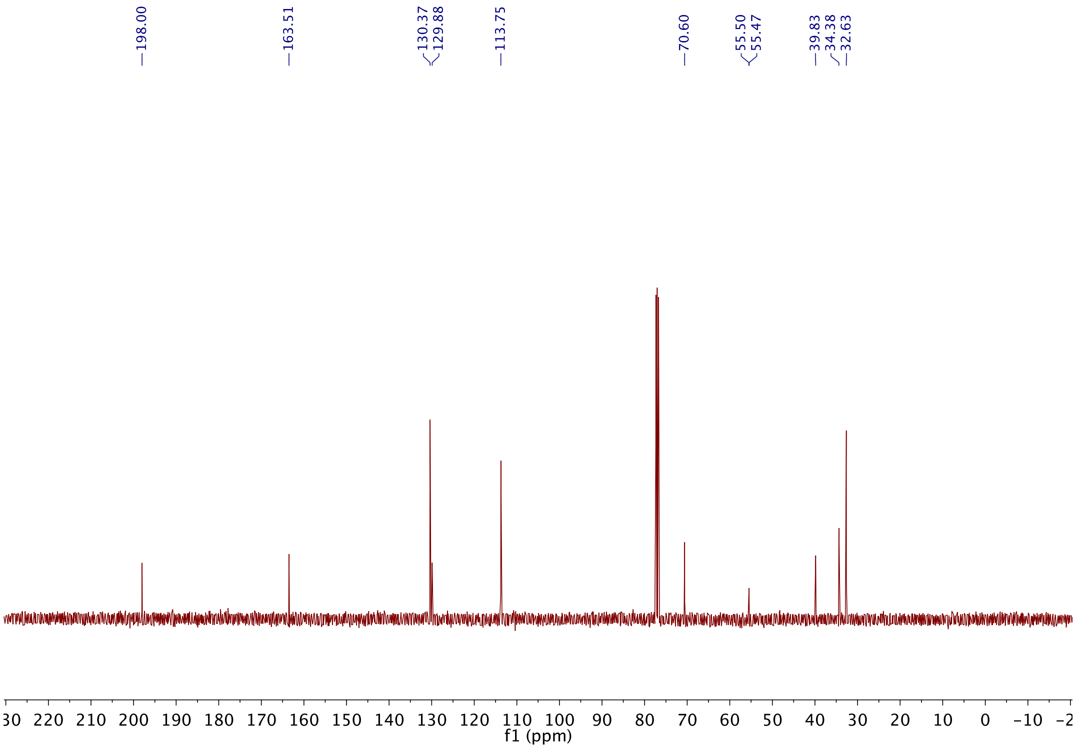

^{1}H and ^{13}C NMR spectra of 4-chloro-4-methyl-1-(4-(trifluoromethyl)phenyl)pentan-1-one (**2d**)

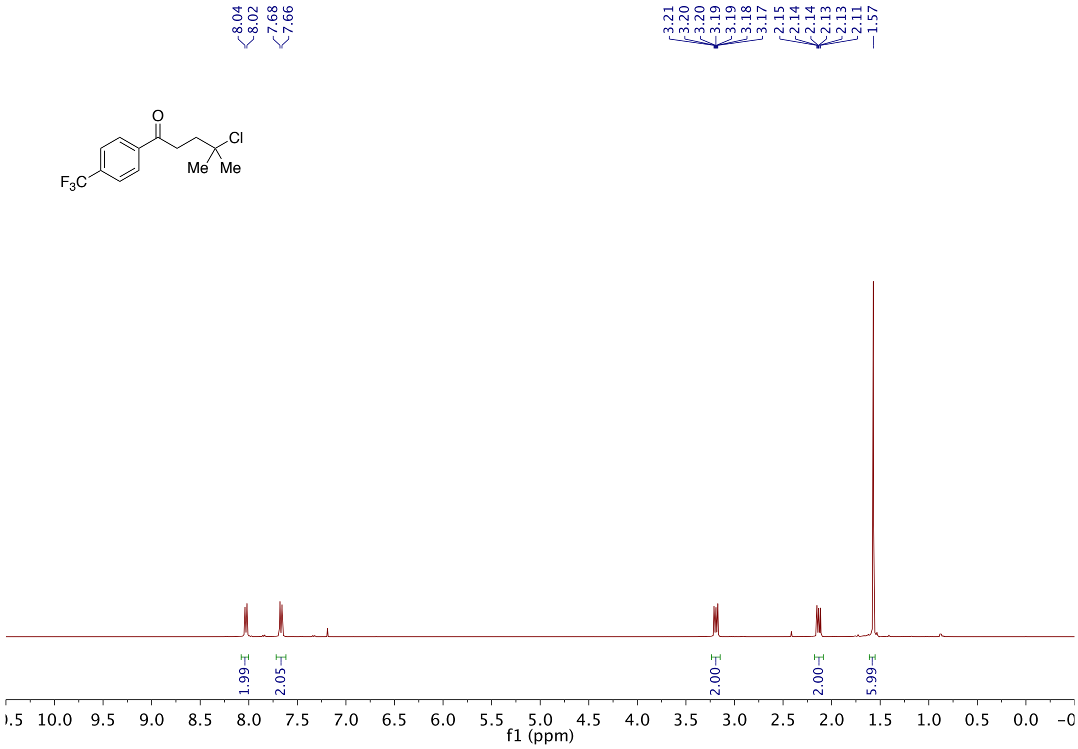

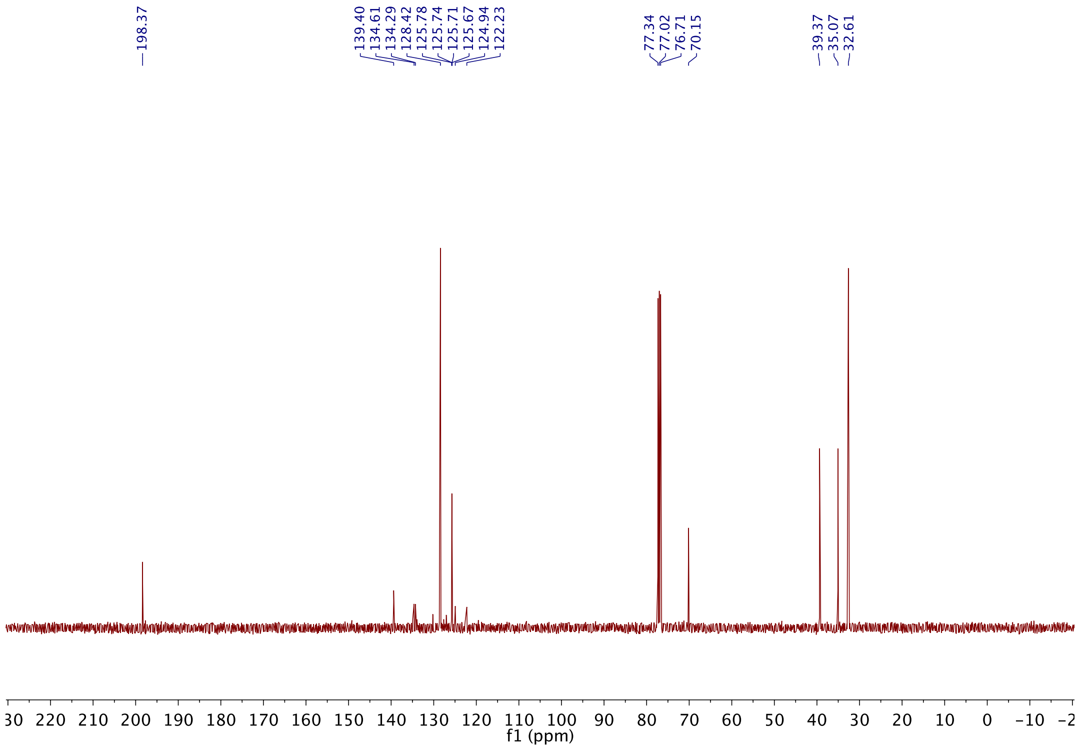

^{1}H and ^{13}C NMR spectra of 1-(4-bromophenyl)-4-chloro-4-methylpentan-1-one (**2e**)

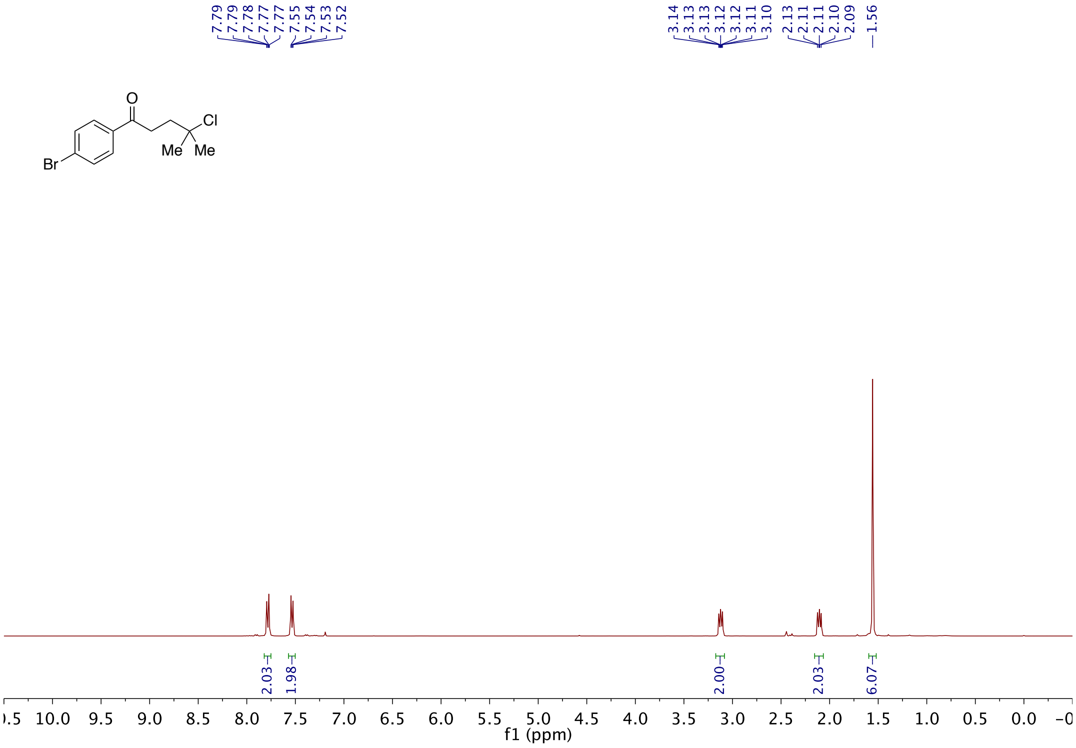

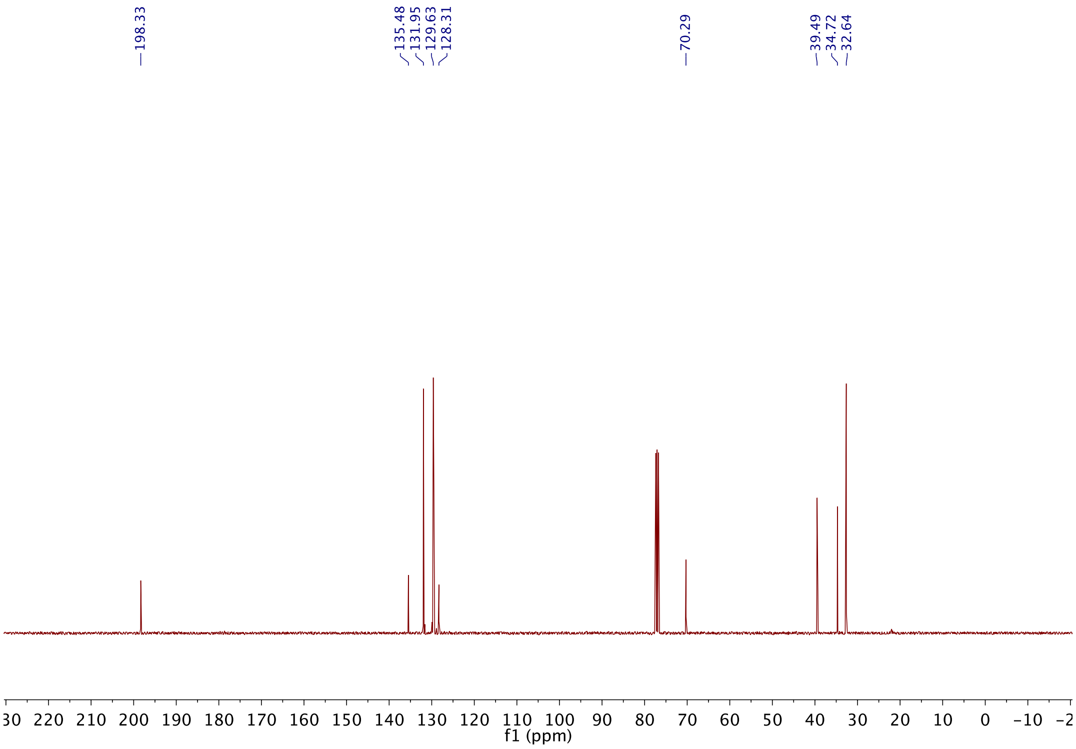

^{1}H and ^{13}C NMR spectra of 4-chloro-1-(3-methoxyphenyl)-4-methylpentan-1-one (**2f**)

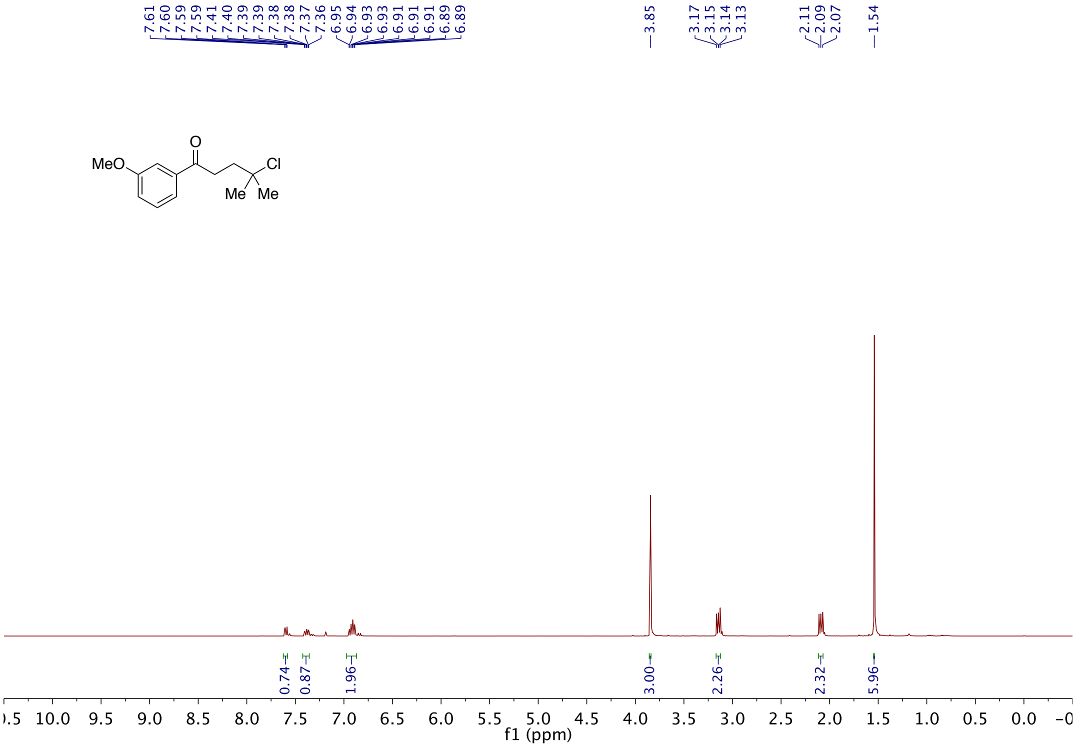

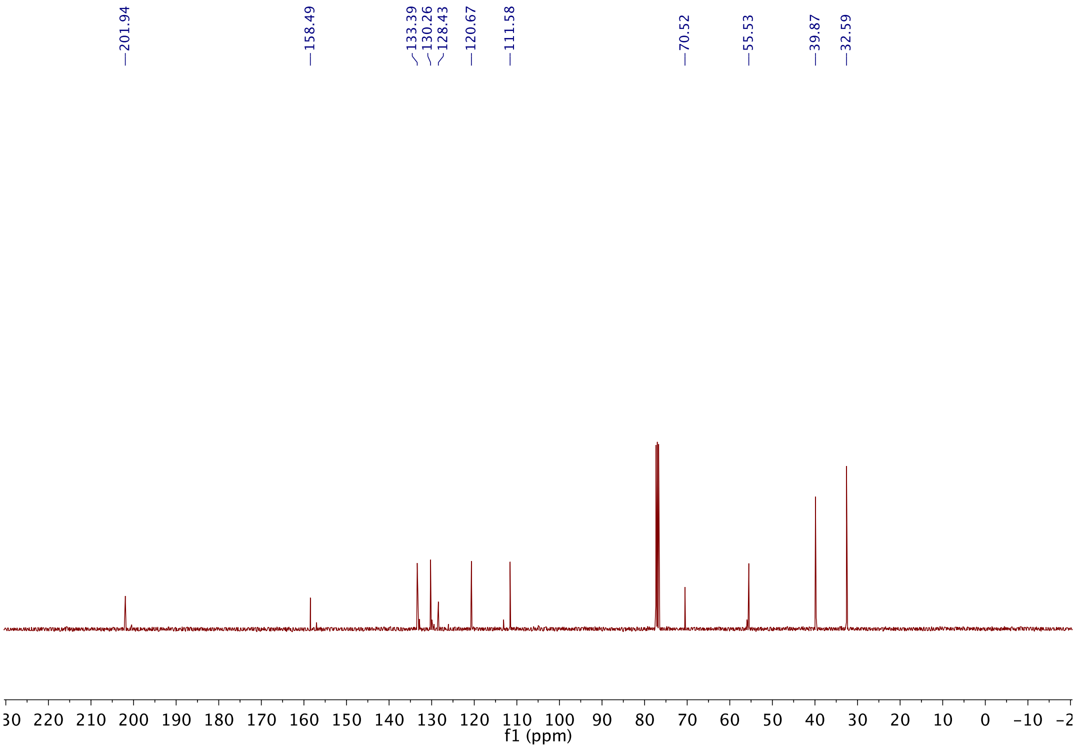

^{1}H and ^{13}C NMR spectra of 4-chloro-4-methyl-1-(*o*-tolyl)pentan-1-one (**2g**)

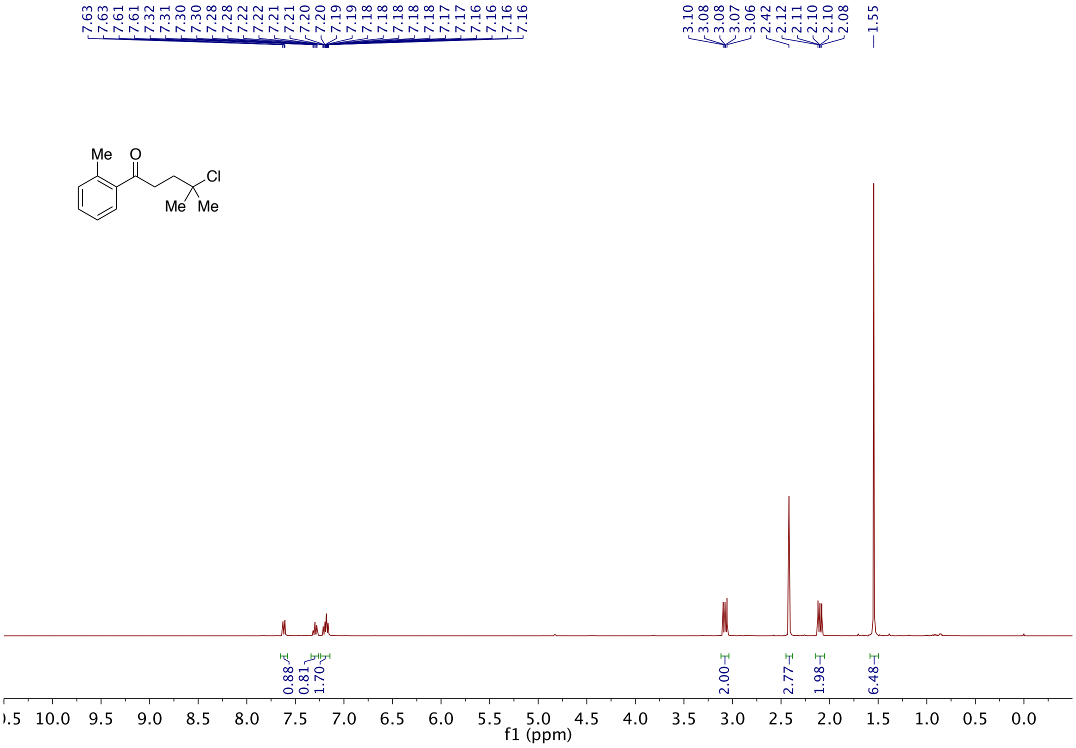

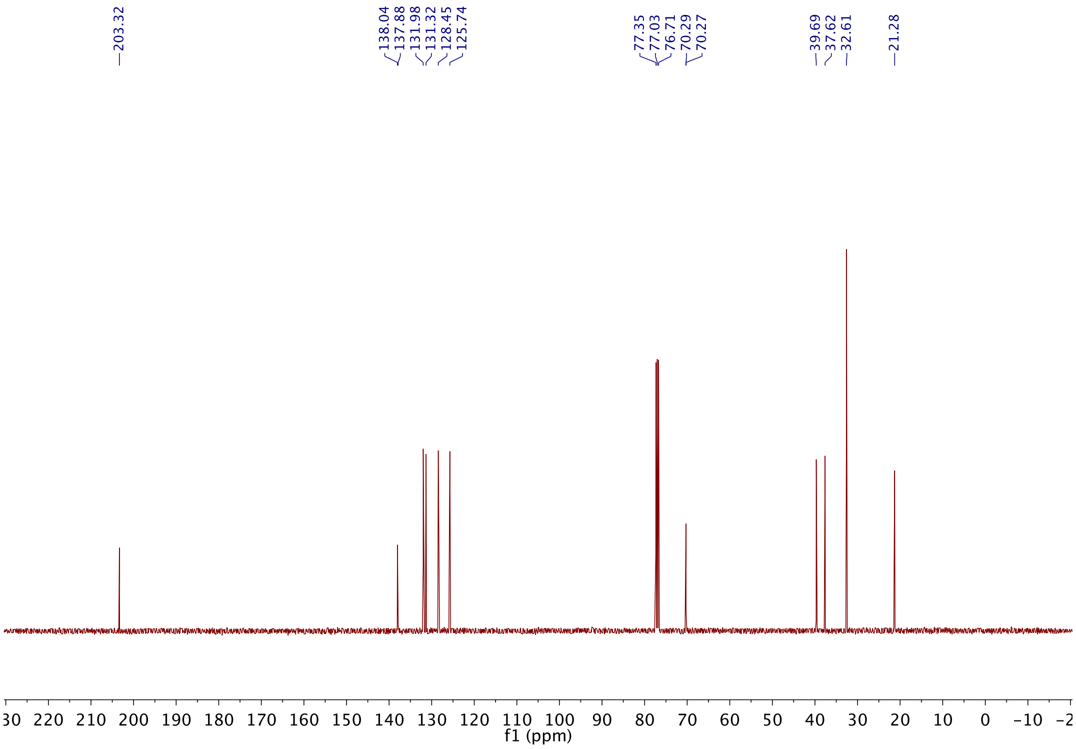

^{1}H and ^{13}C NMR spectra of 4-chloro-1-(3-fluorophenyl)-4-methylpentan-1-one (**2h**)

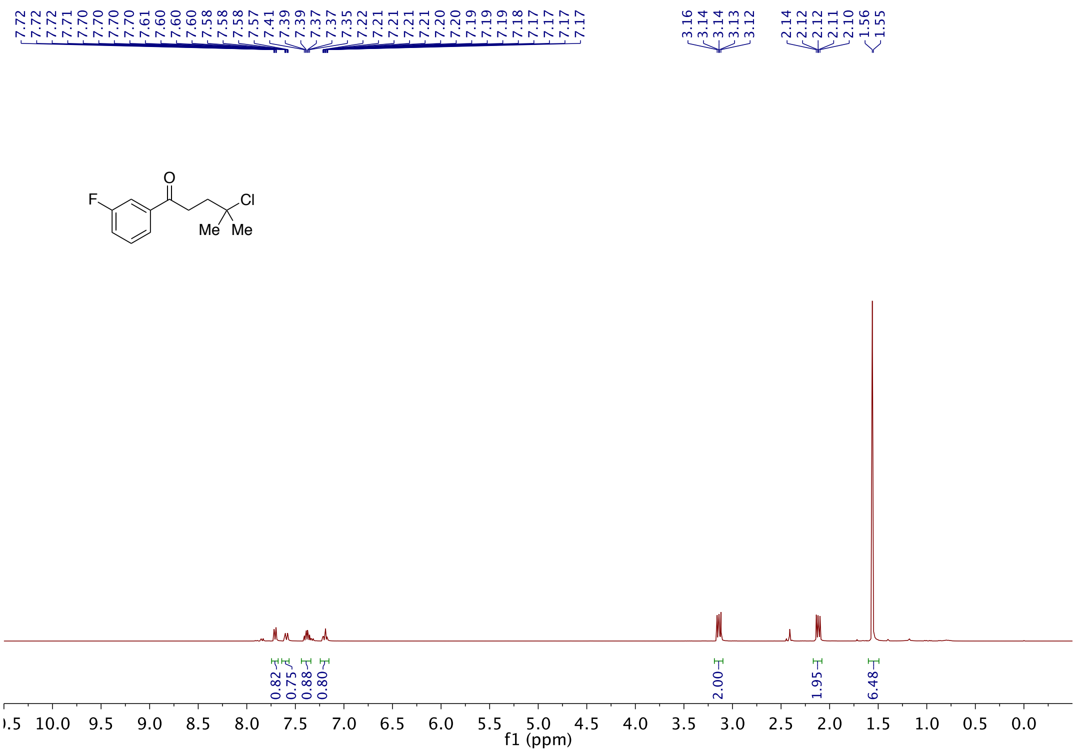

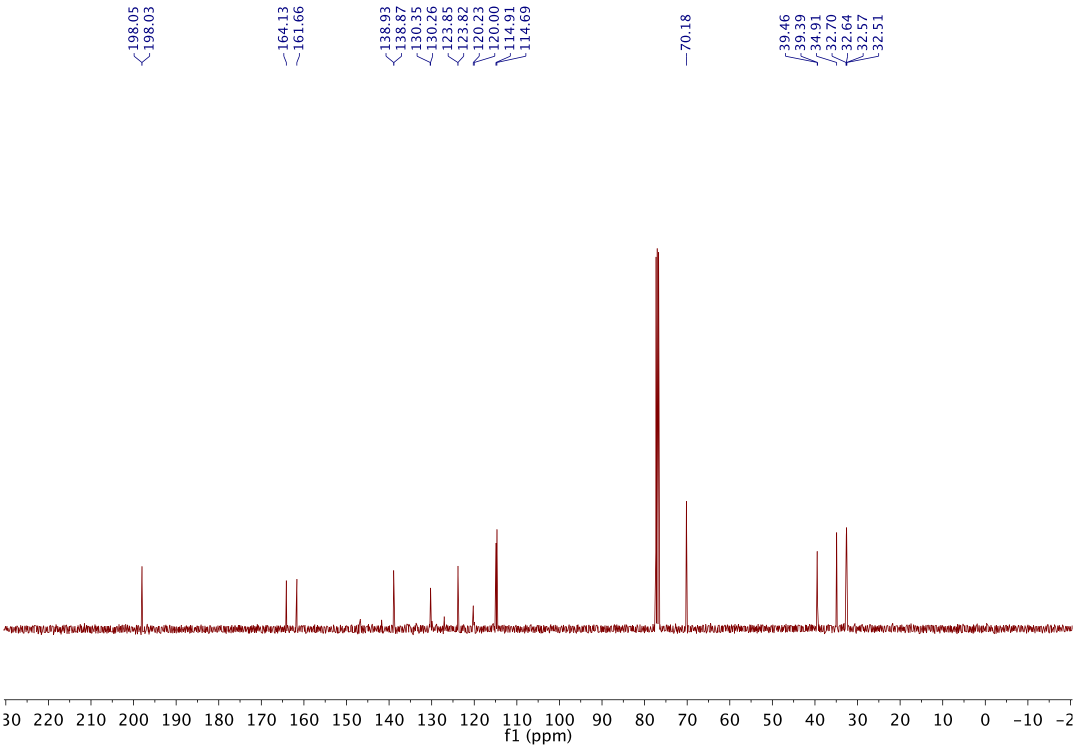

^1H and ^{13}C NMR spectra of 1-(3,5-bis(trifluoromethyl)phenyl)-4-chloro-4-methylpentan-1-one (**2i**)

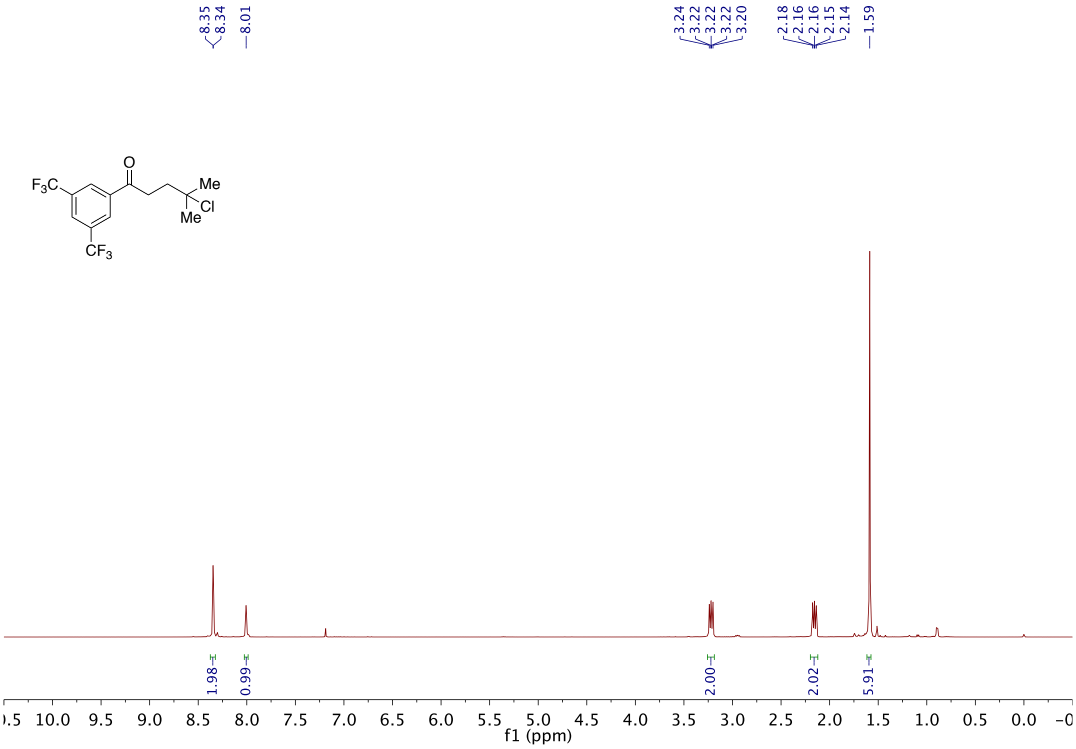

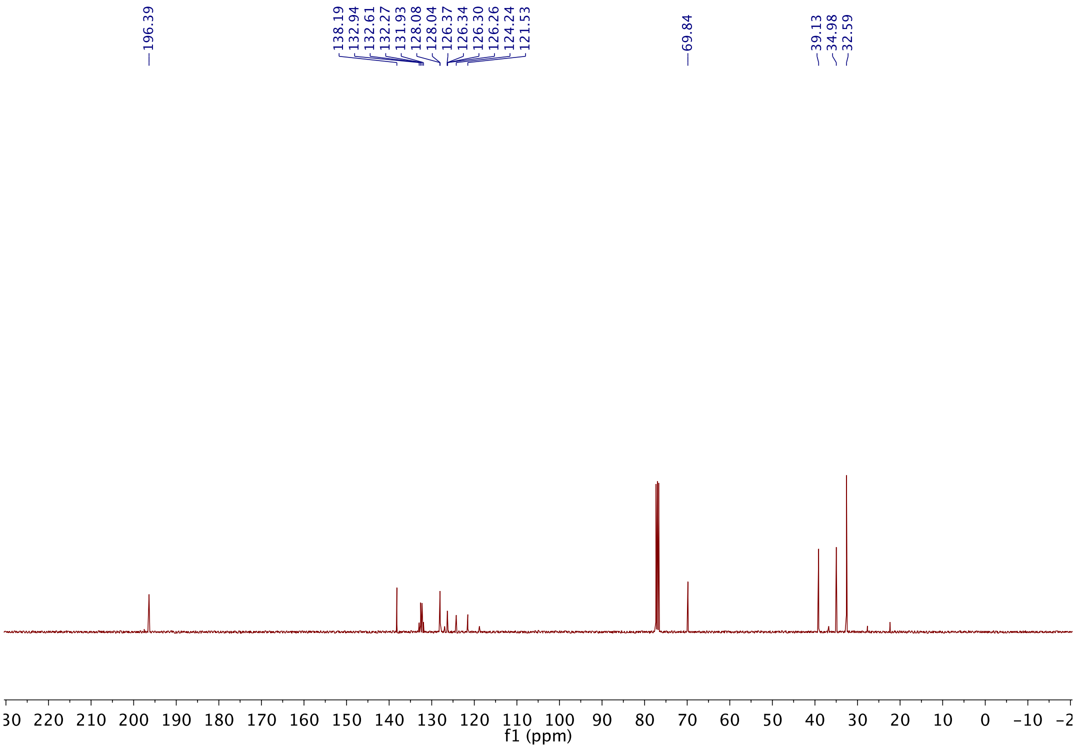

^{1}H and ^{13}C NMR spectra of 4-chloro-4-methyl-1-(phenanthren-9-yl)pentan-1-one (**2j**)

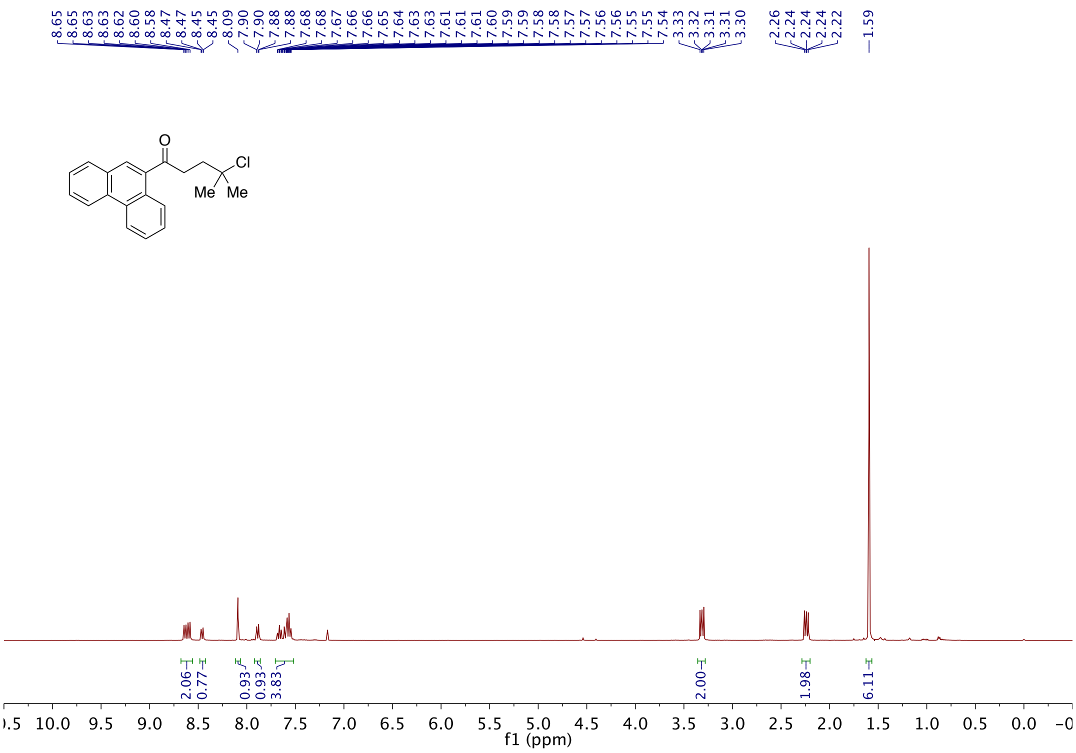

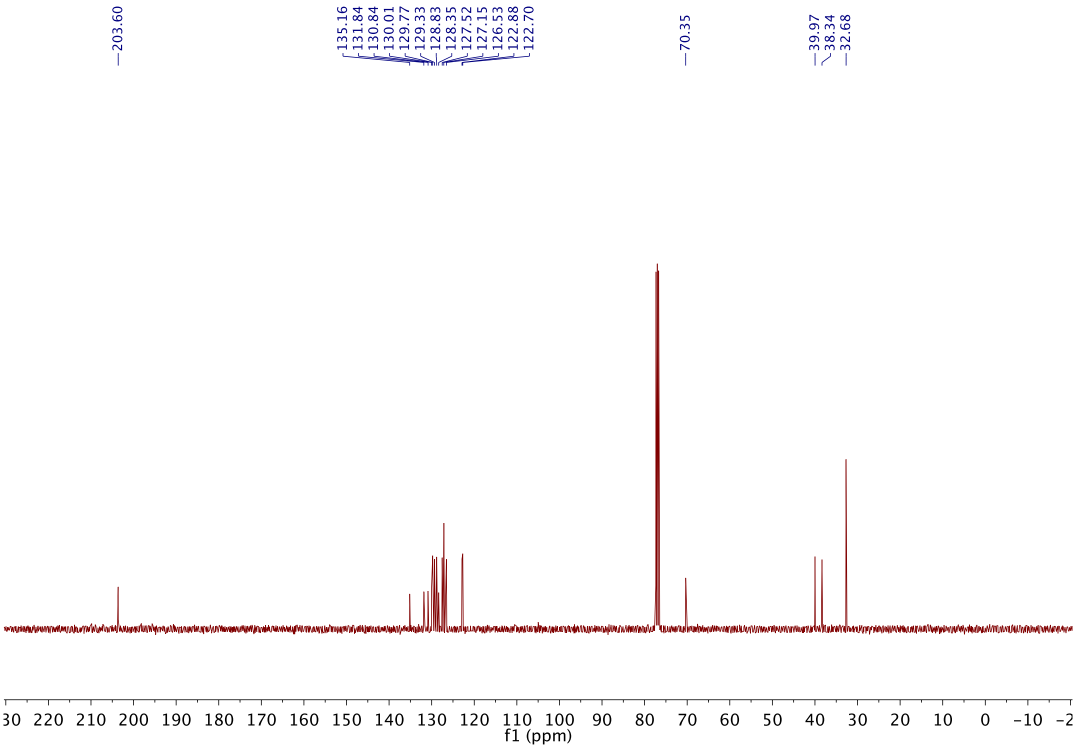

^{1}H and ^{13}C NMR spectra of 4-chloro-4-methyl-1-phenyloctan-1-one (**2k**)

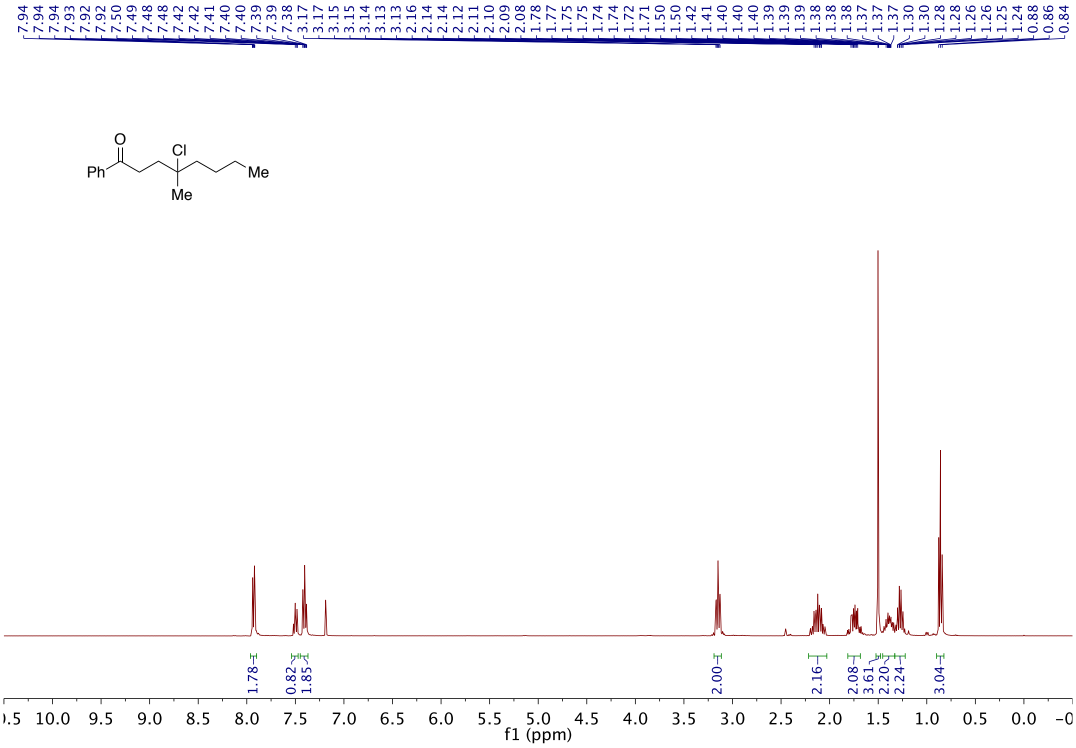

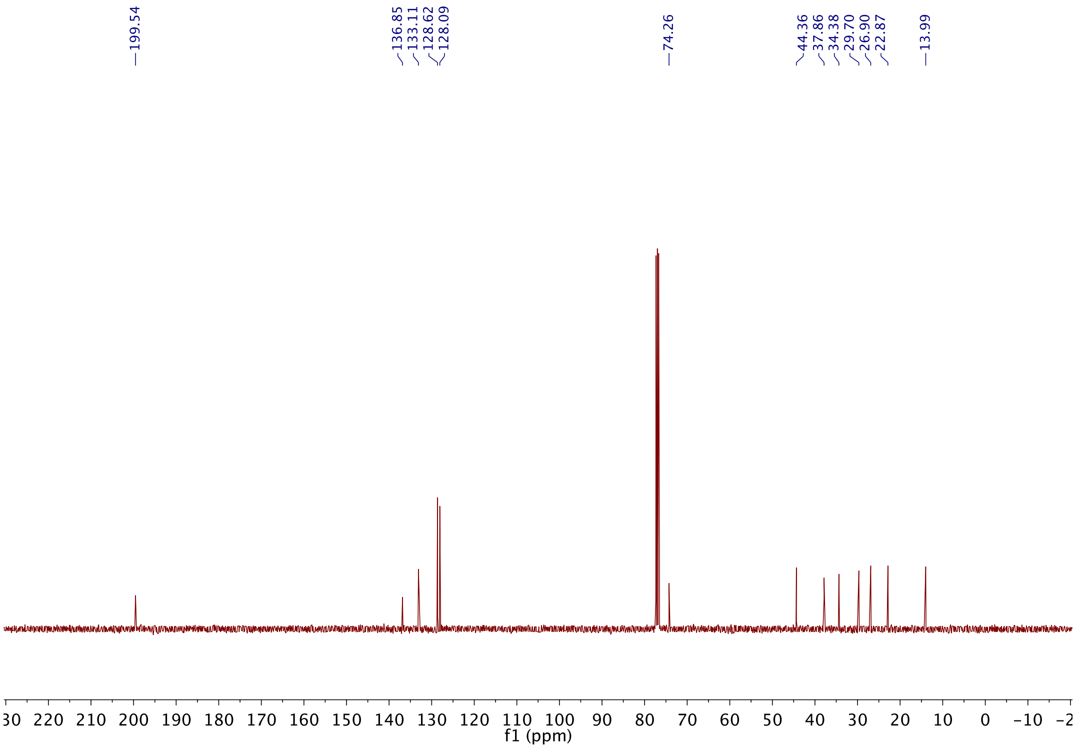

^{1}H and ^{13}C NMR spectra of 4-chloro-4-ethyl-1-phenyloctan-1-one (**2l**)

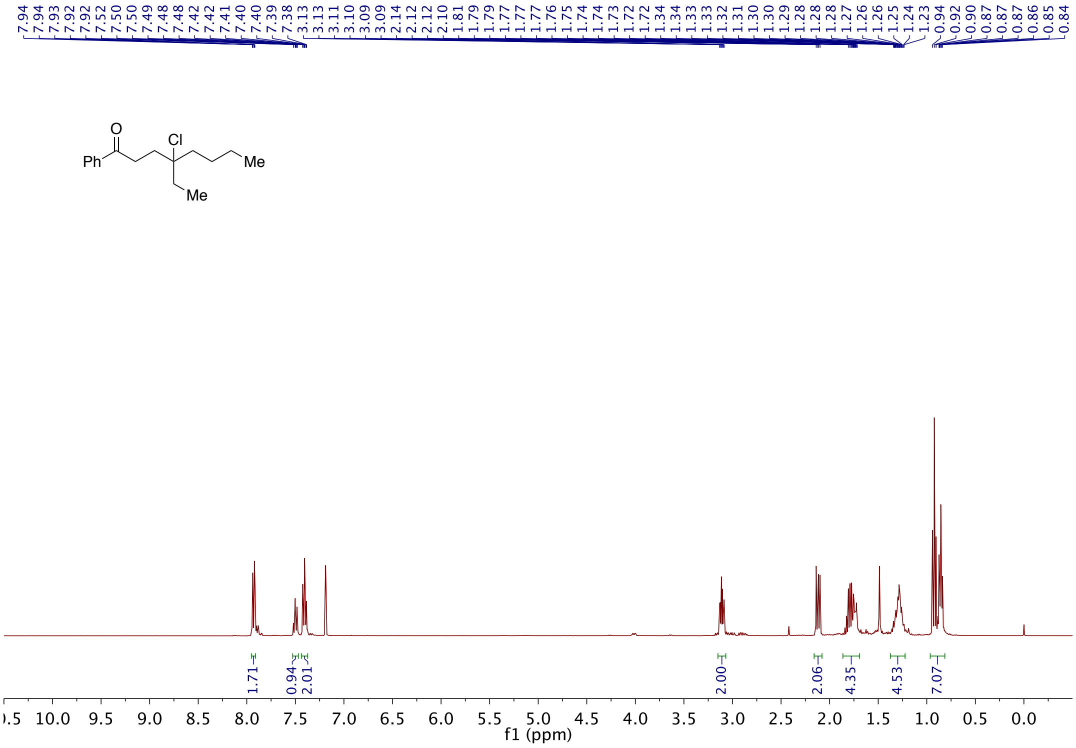

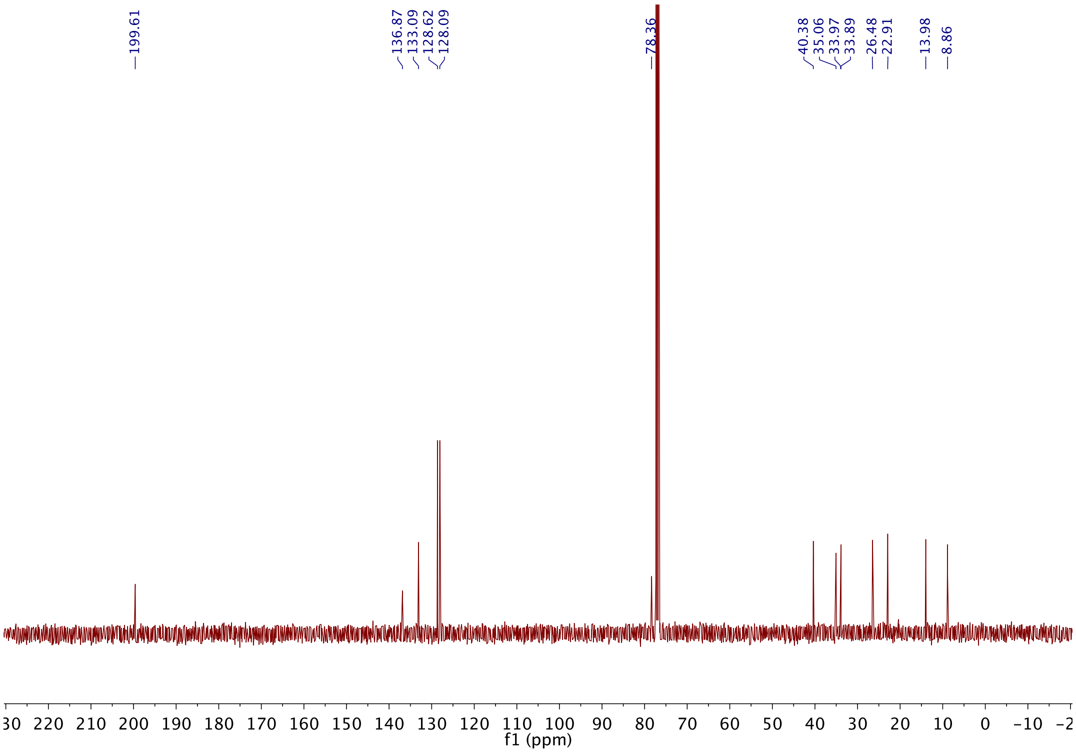

^{1}H and ^{13}C NMR spectra of 4-chloro-2,4-dimethyl-1-phenylpentan-1-one (**2m**)

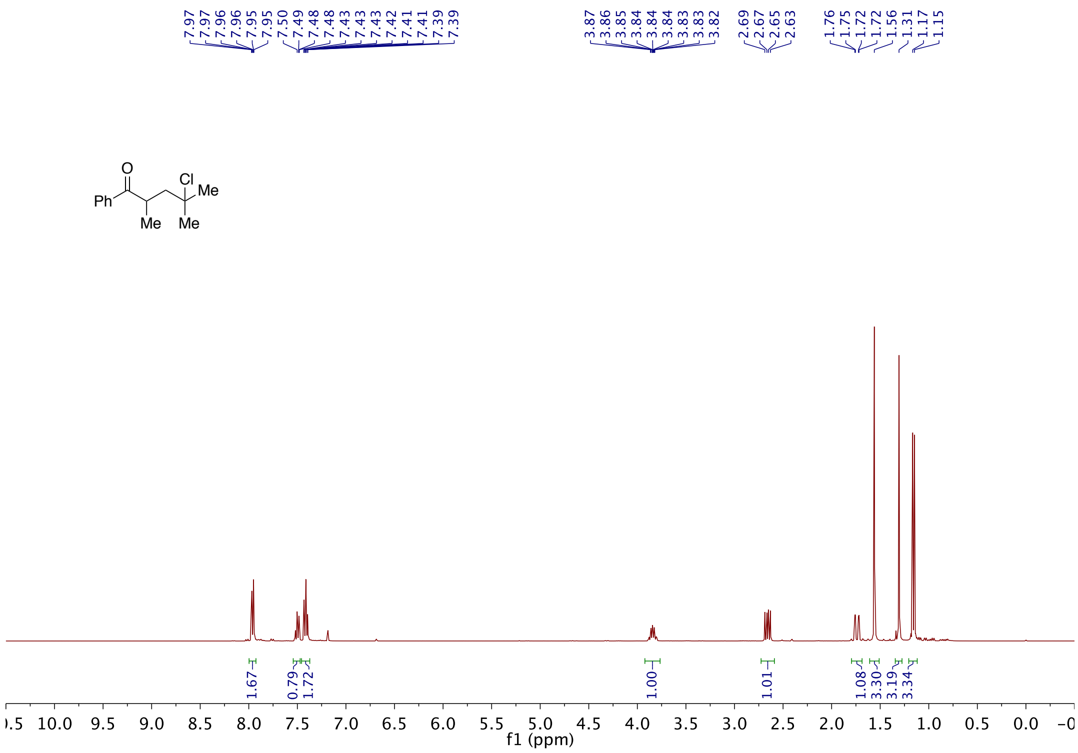

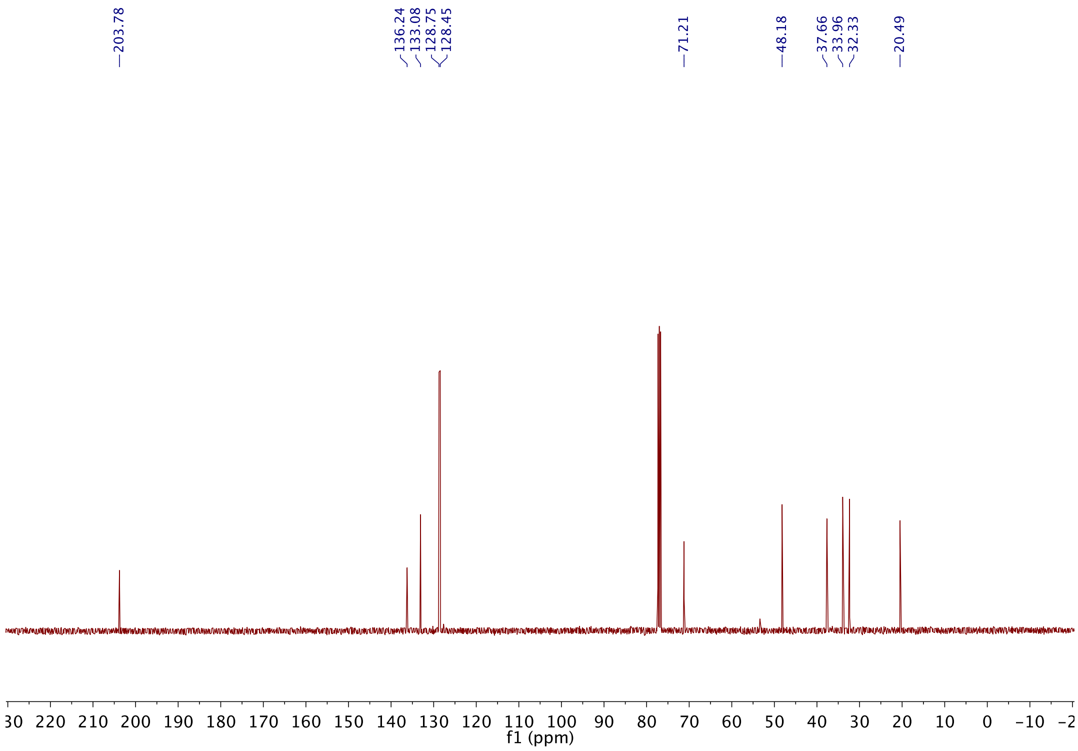

^{1}H and ^{13}C NMR spectra of 3-(1-chlorocyclopentyl)-1-phenylpropan-1-one (**2n**)

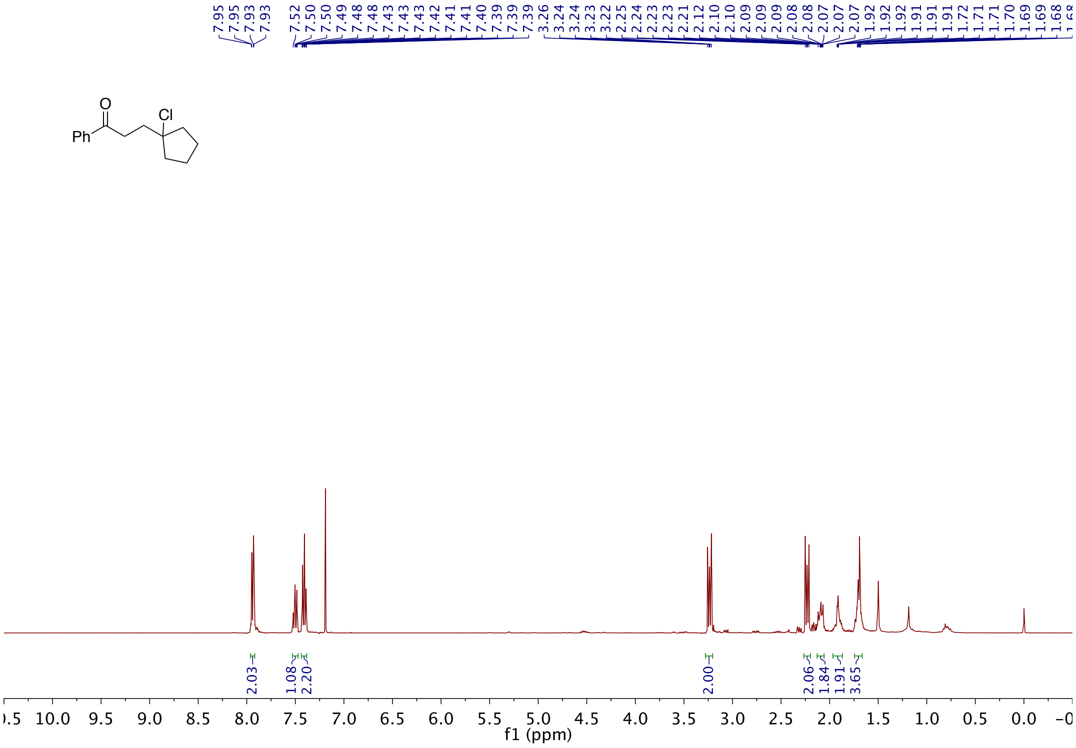

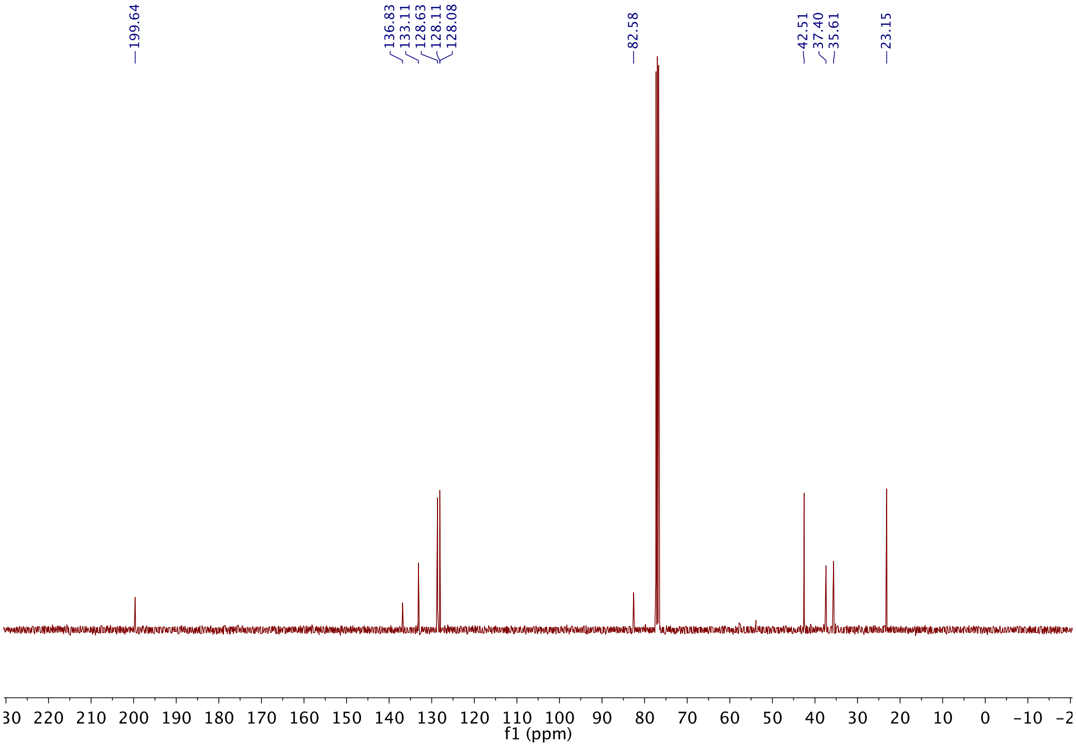

^{1}H and ^{13}C NMR spectra of 3-(1-chlorocyclohexyl)-1-phenylpropan-1-one (**2o**)

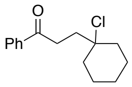

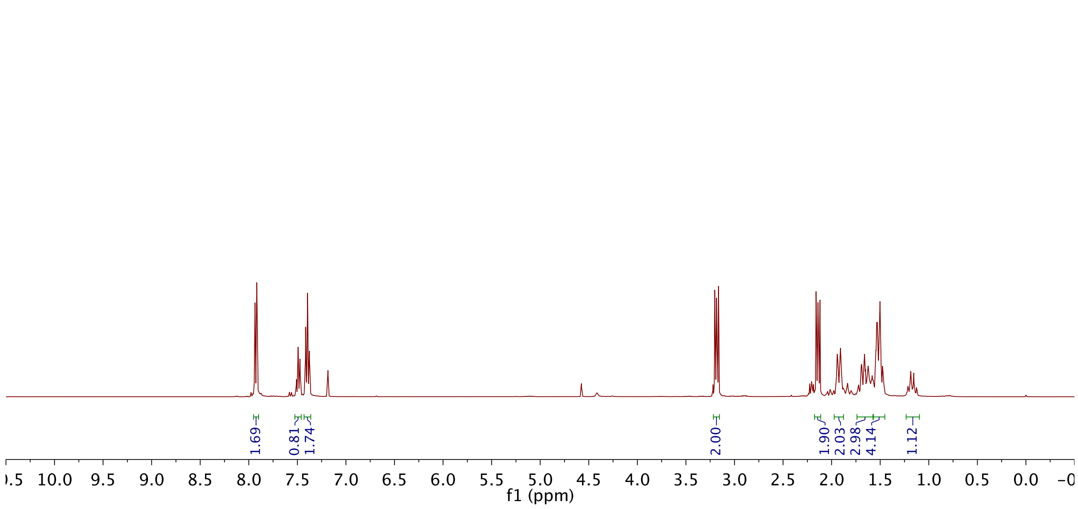

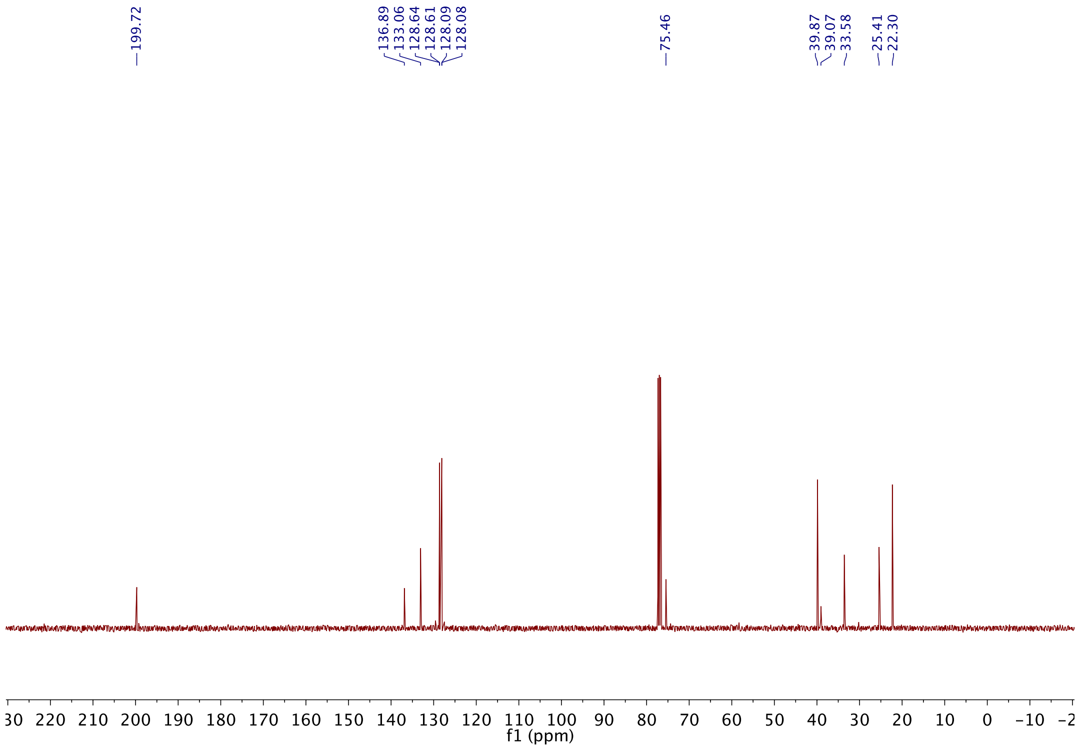

^{1}H and ^{13}C NMR spectra of 6-chloro-6-methyl-2-phenylheptan-3-one (**2p**)

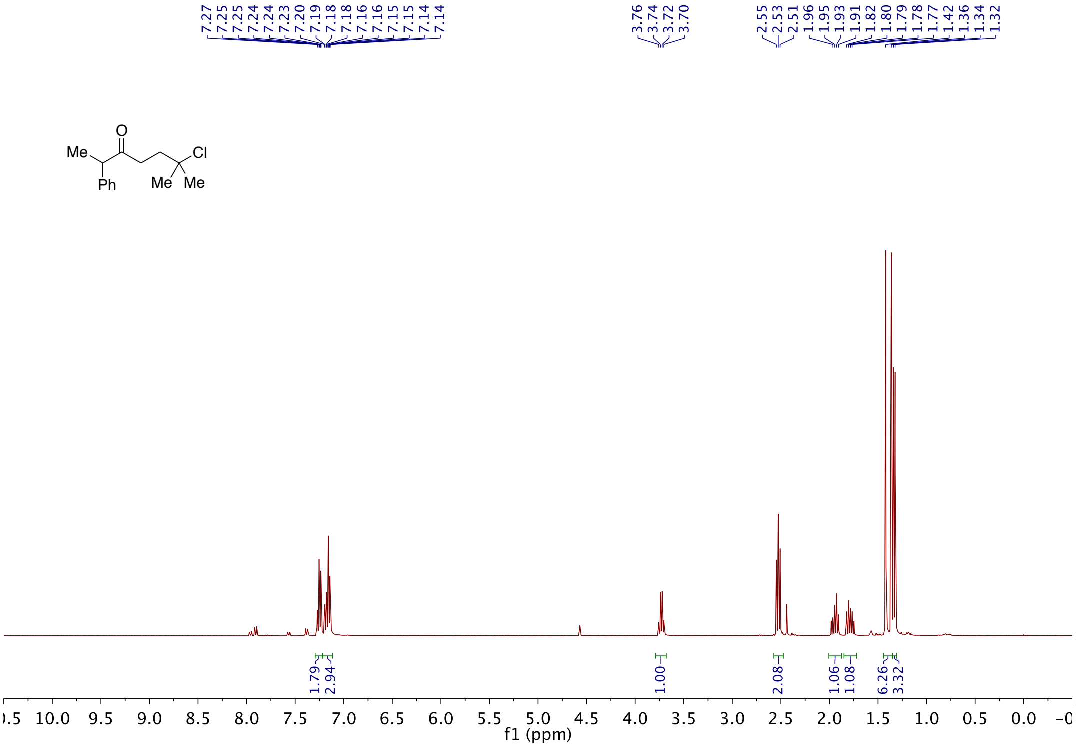

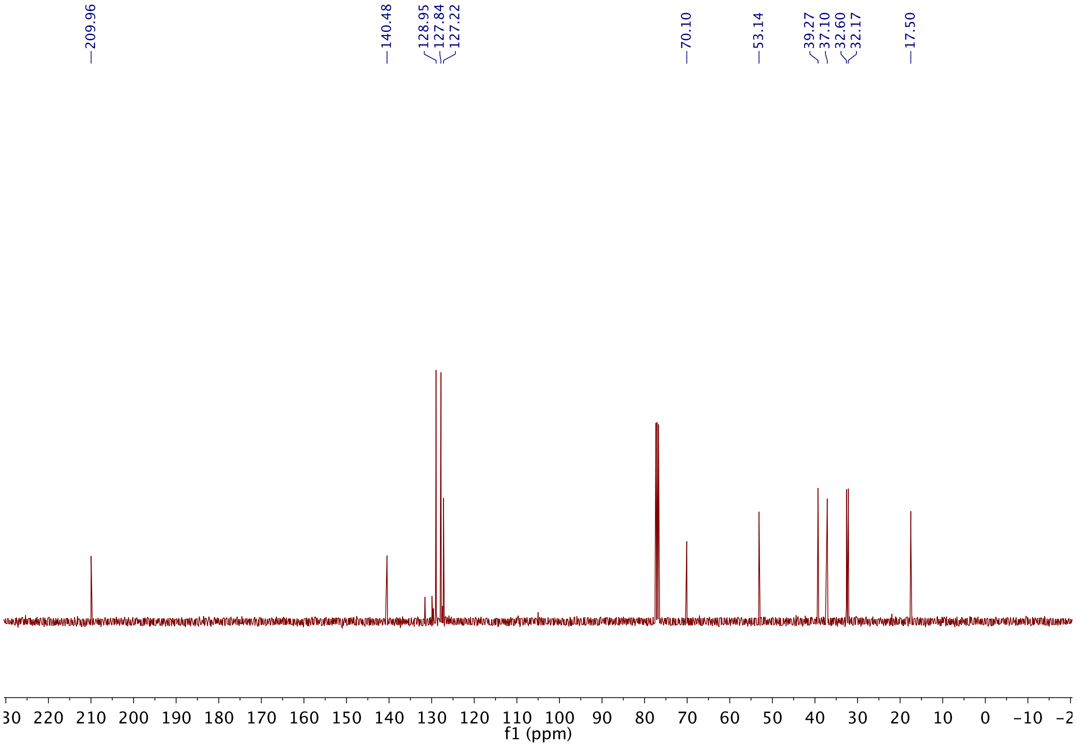

^{1}H and ^{13}C NMR spectra of 7-chloro-7-methyl-3-phenyloctan-4-one (**2q**)

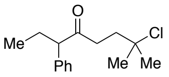

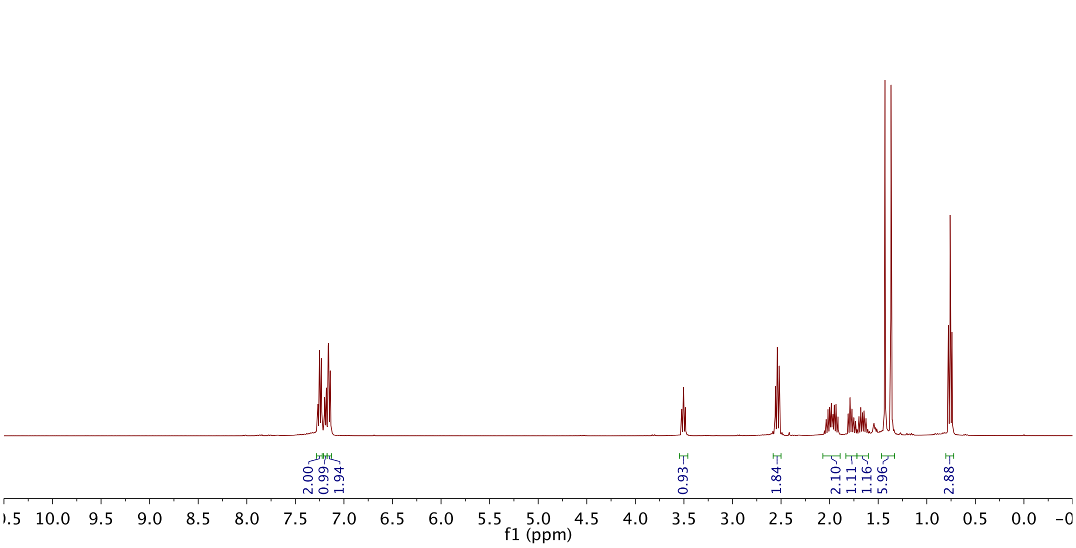

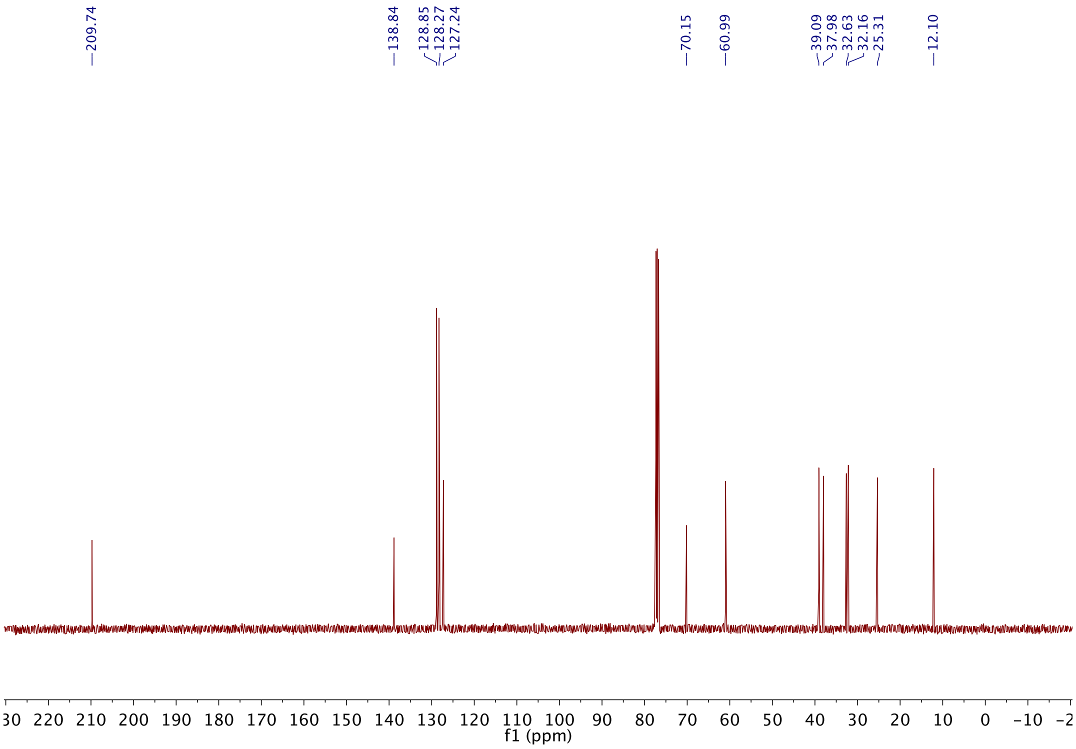

^{1}H and ^{13}C NMR spectra of phenyl 4-chloro-4-methylpentanoate (**2r**)

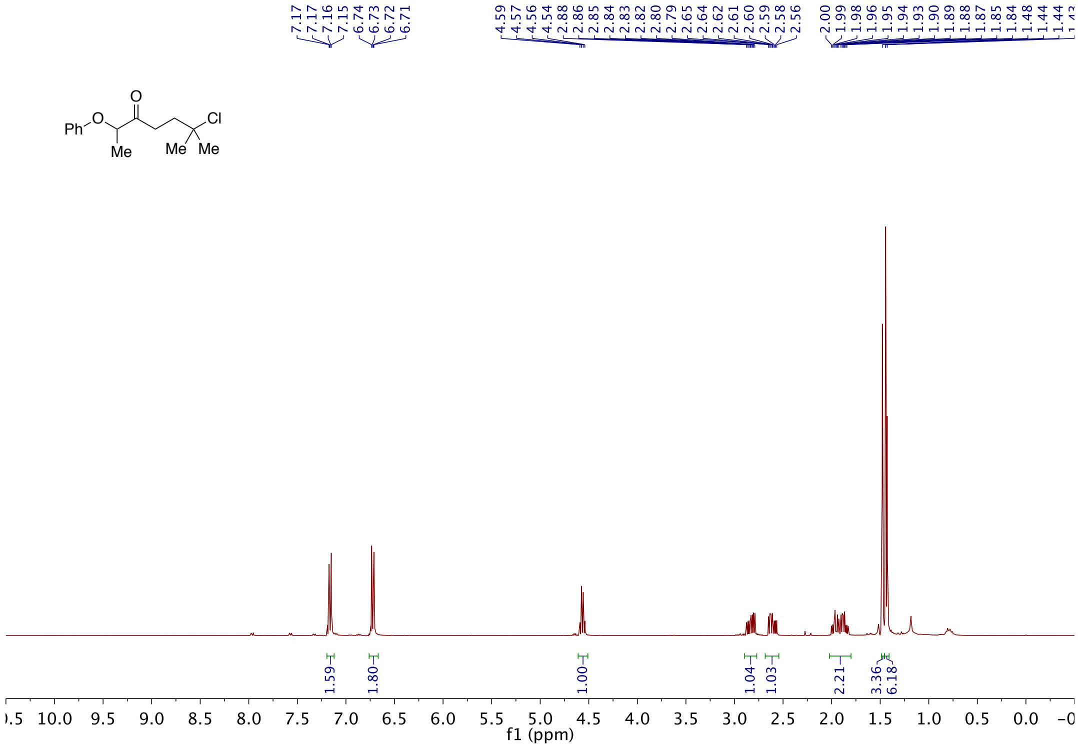

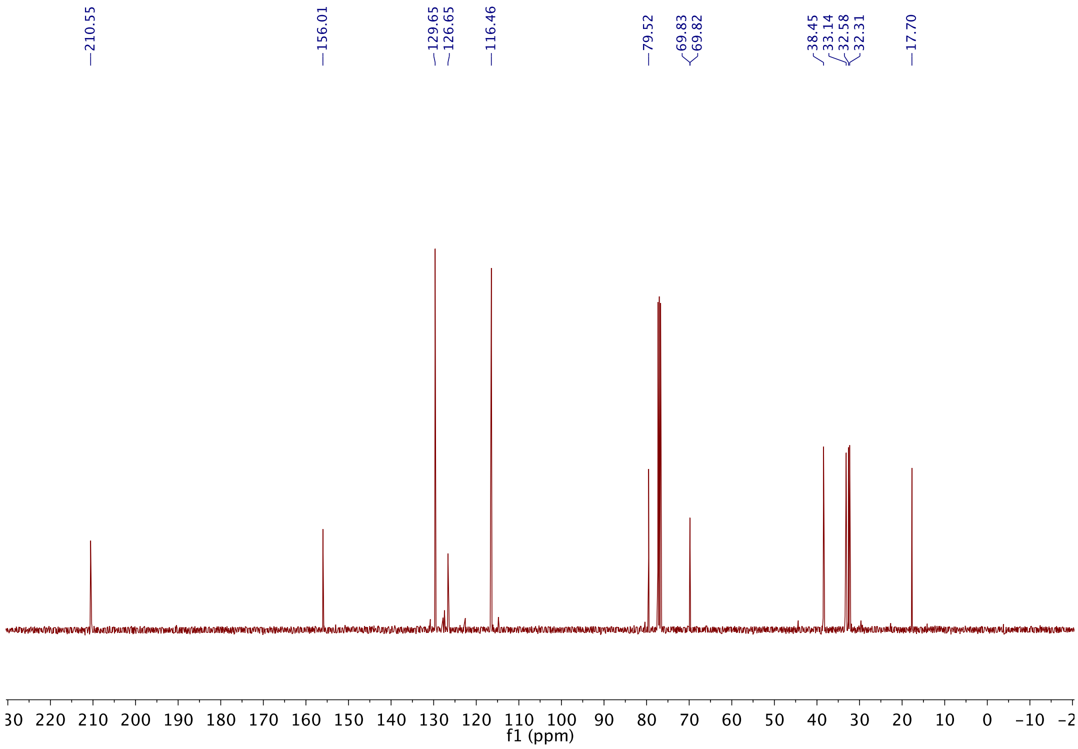

^{1}H and ^{13}C NMR spectra of 4-chloro-1-phenylpentan-1-one (**2s**)

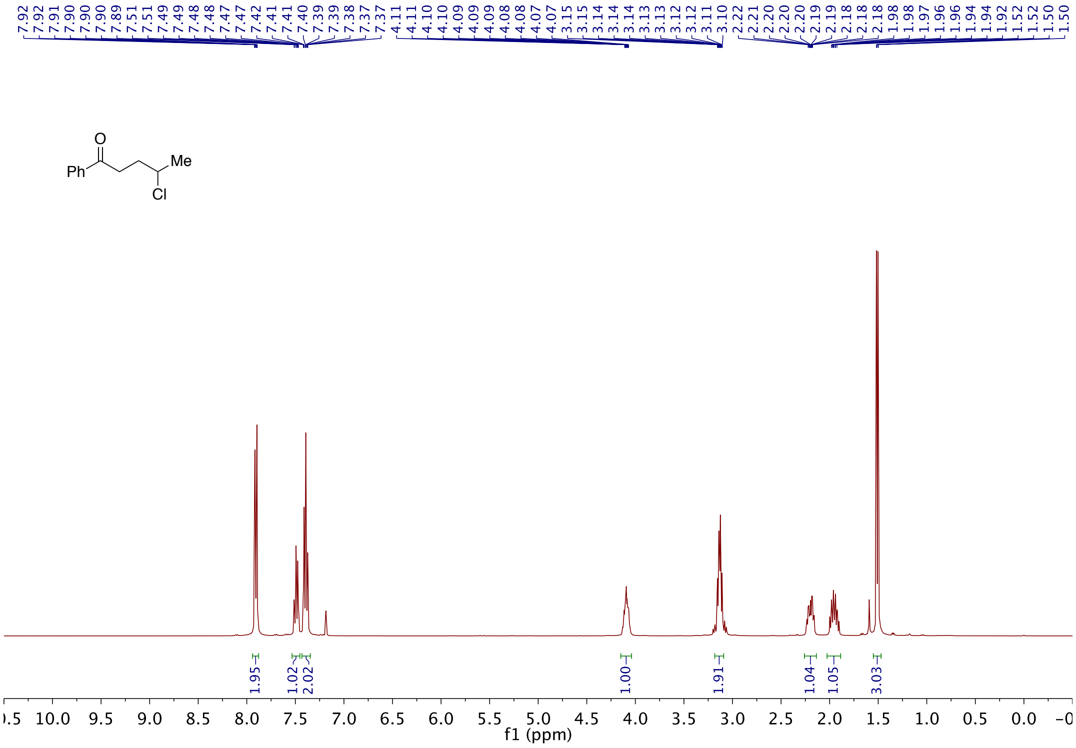

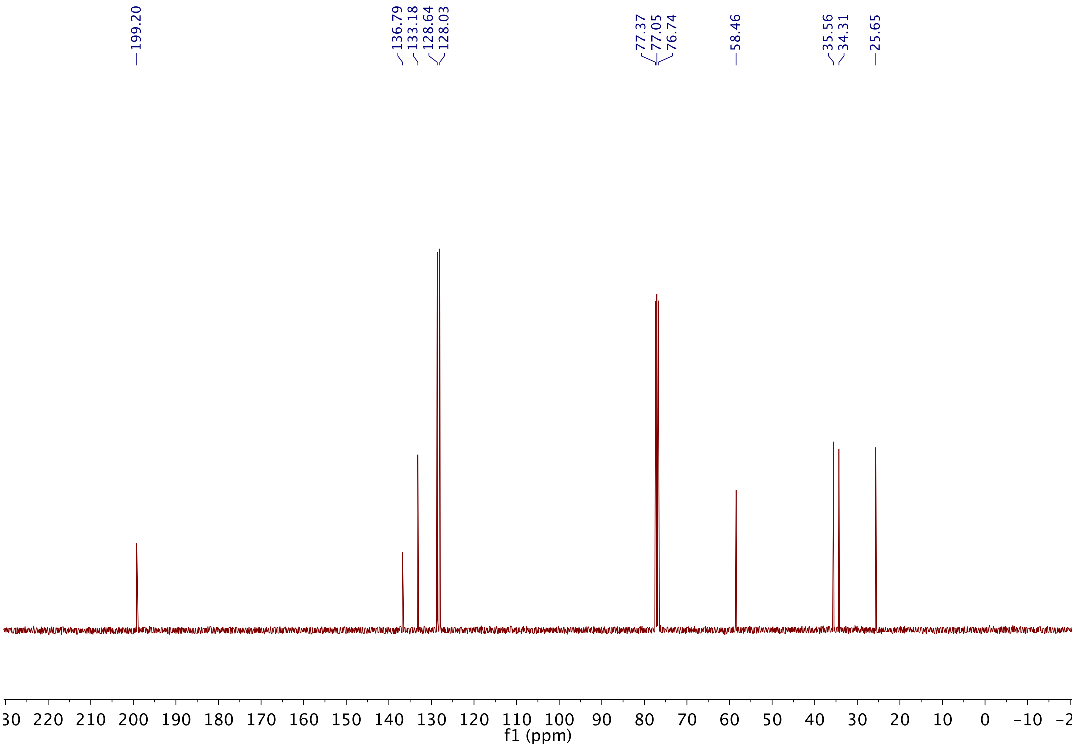

^{1}H and ^{13}C NMR spectra of 4-chloro-1-(4-(trifluoromethyl)phenyl)pentan-1-one (**2t**)

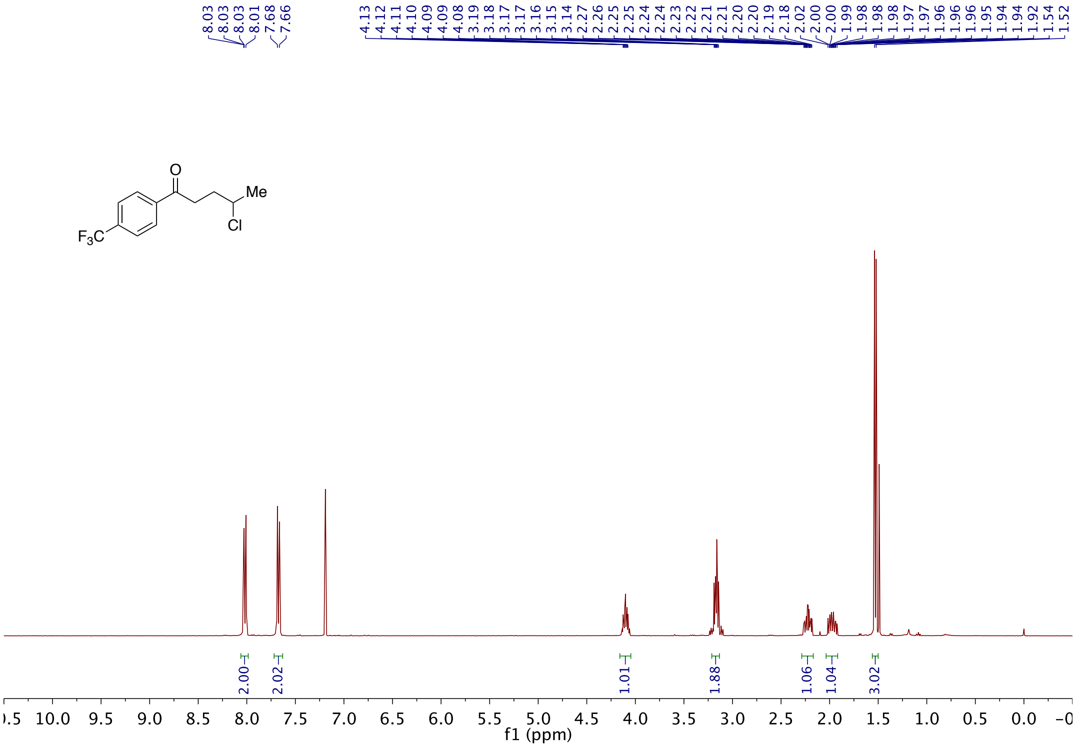

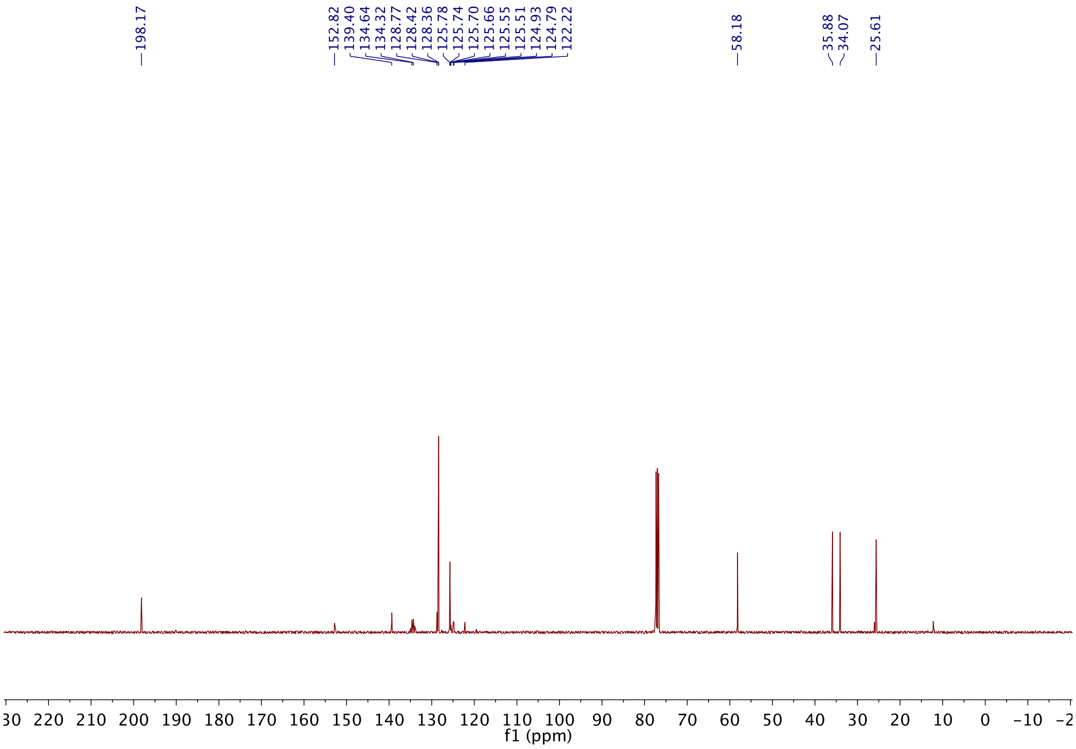

^{1}H and ^{13}C NMR spectra of 4-chloro-1-(4-chlorophenyl)pentan-1-one (**2u**)

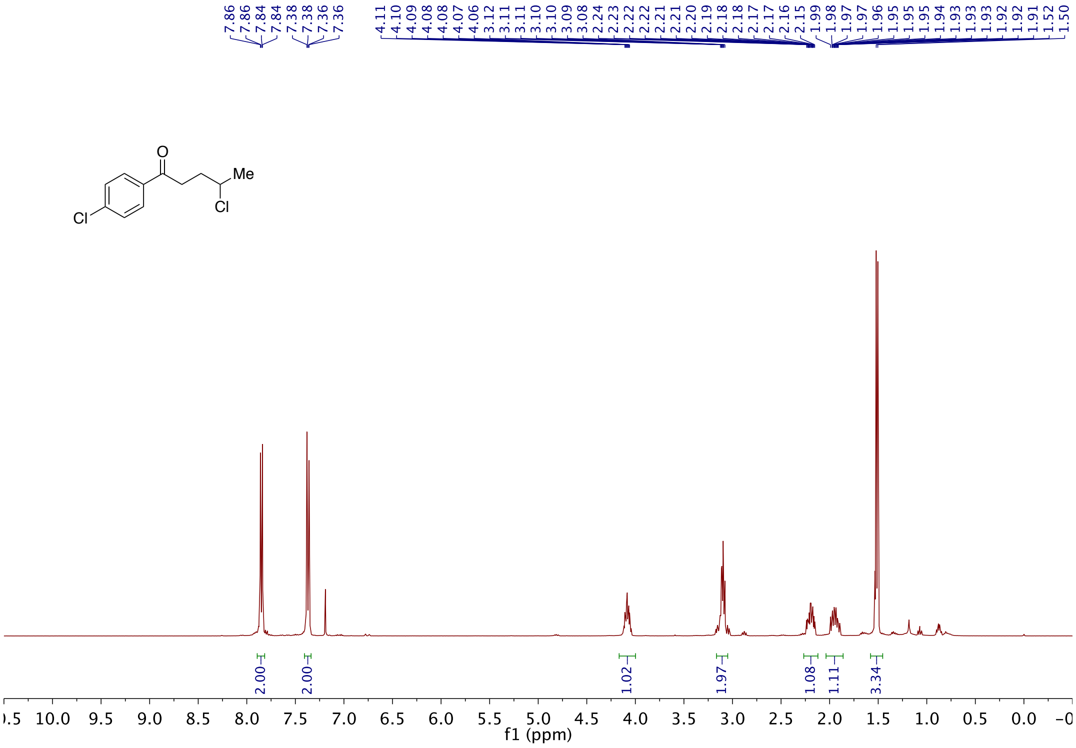

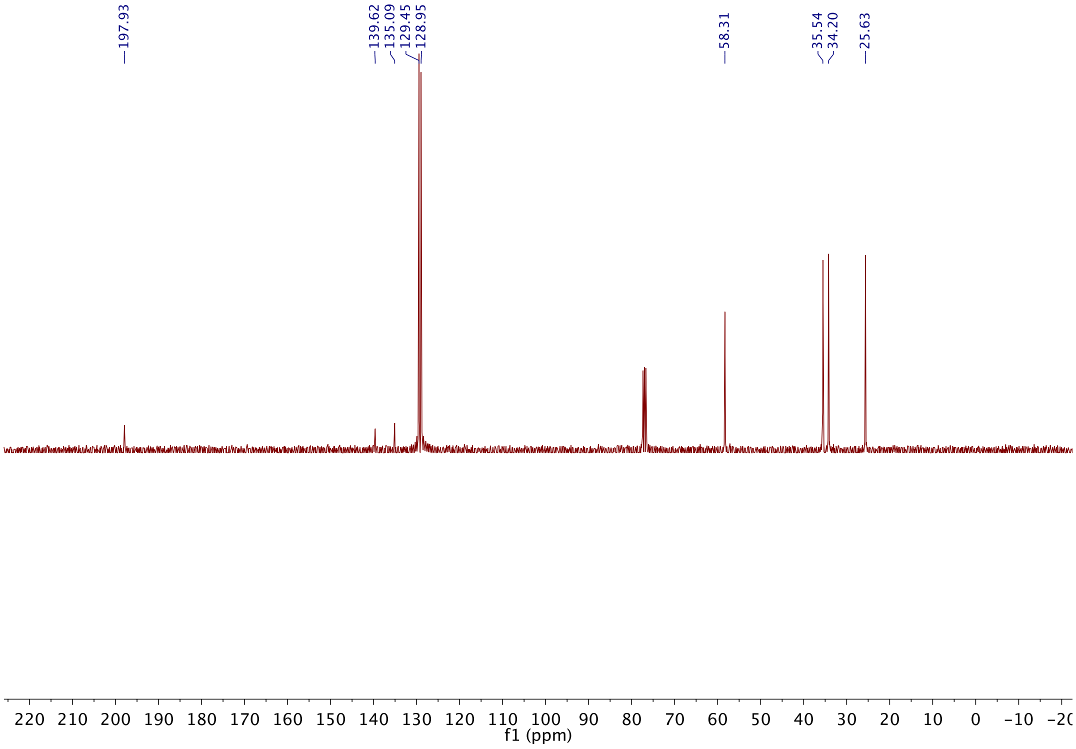

^{1}H and ^{13}C NMR spectra of 4-chloro-1-(3-fluorophenyl)pentan-1-one (**2v**)

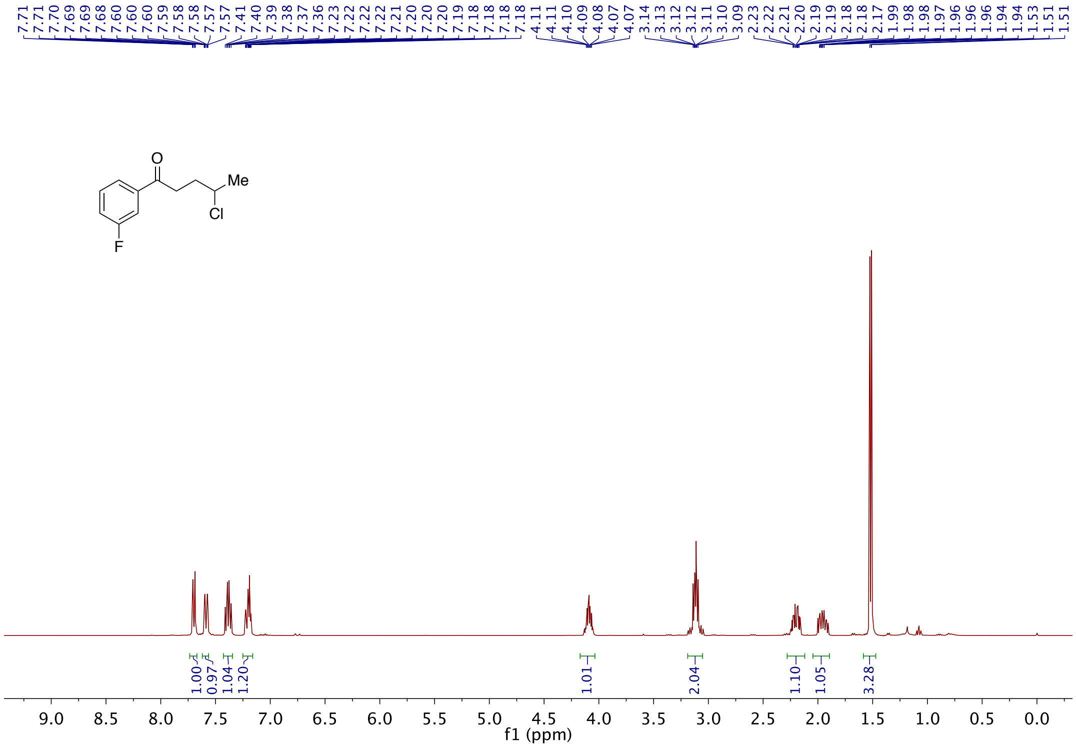

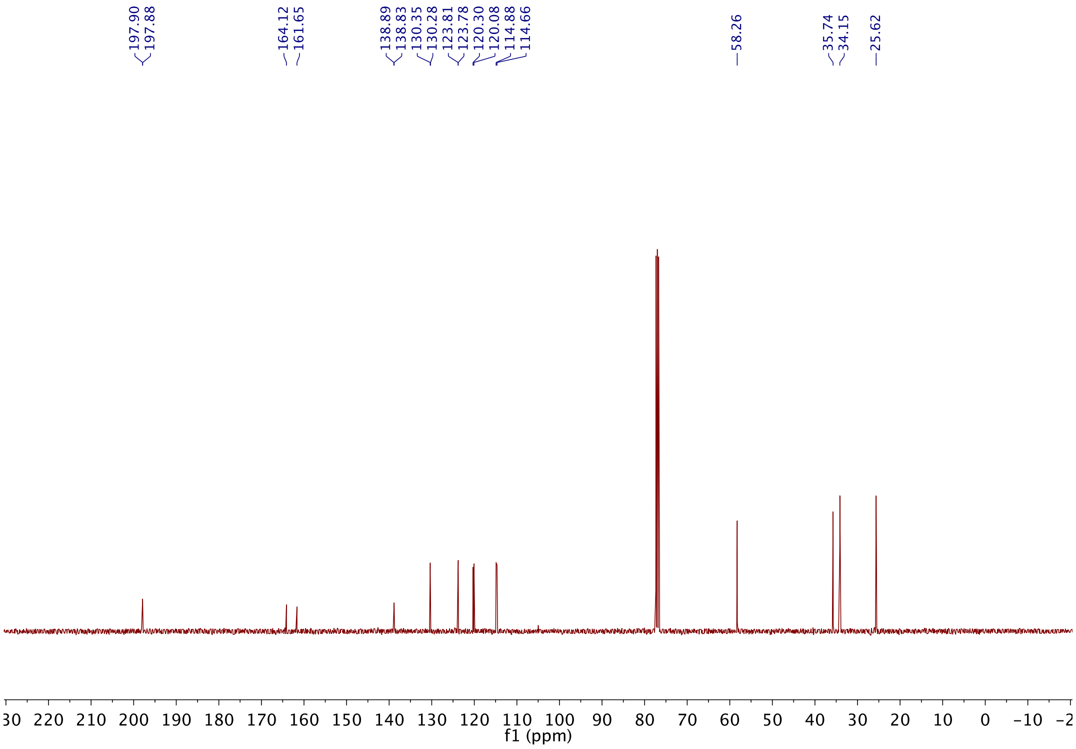

^{1}H and ^{13}C NMR spectra of 4-chloro-1-(3-chloro-4-methoxyphenyl)pentan-1-one (**2w**)

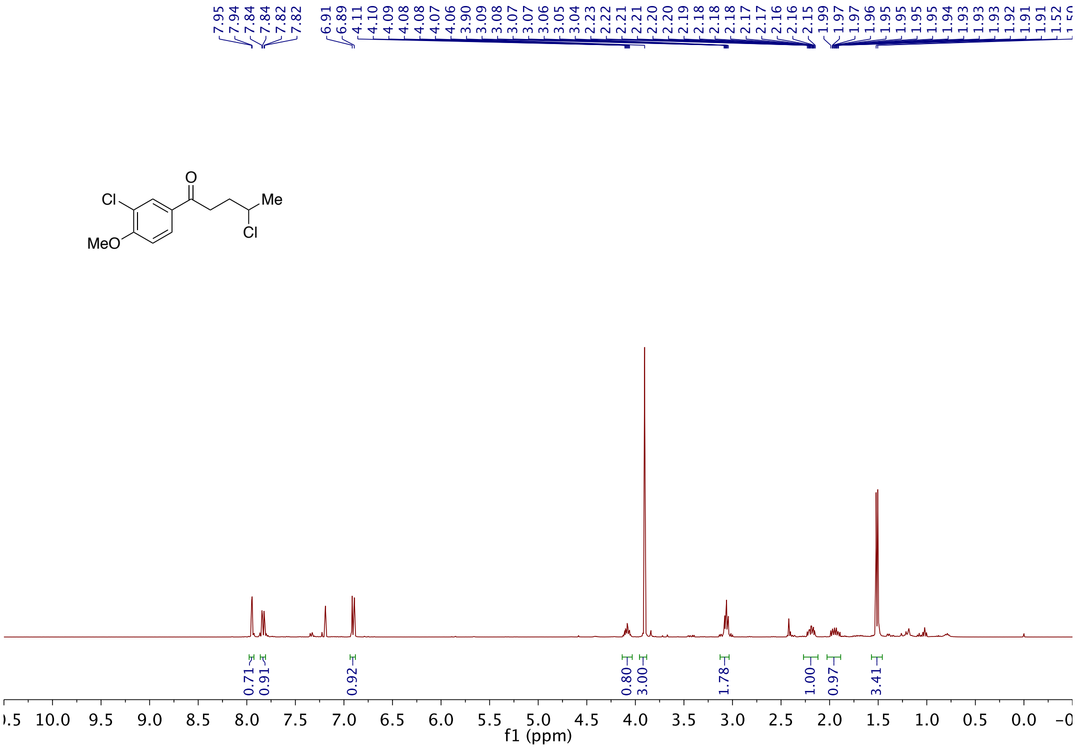

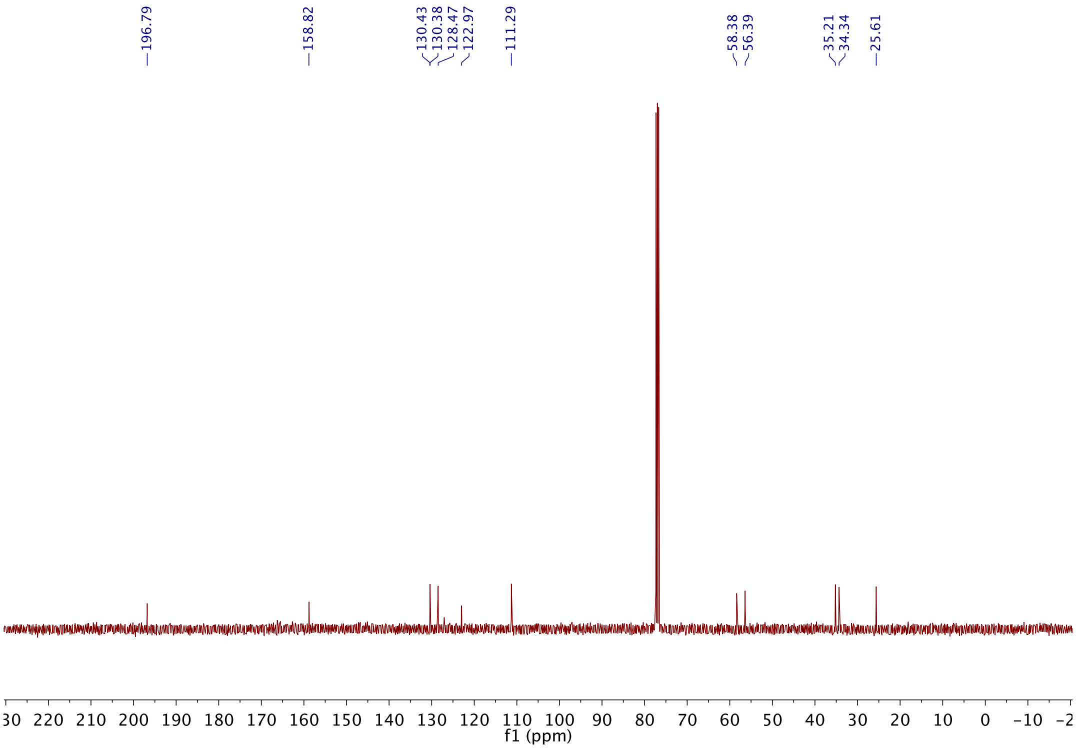

^{1}H and ^{13}C NMR spectra of 6-chloro-2-phenylheptan-3-one (**2x**)

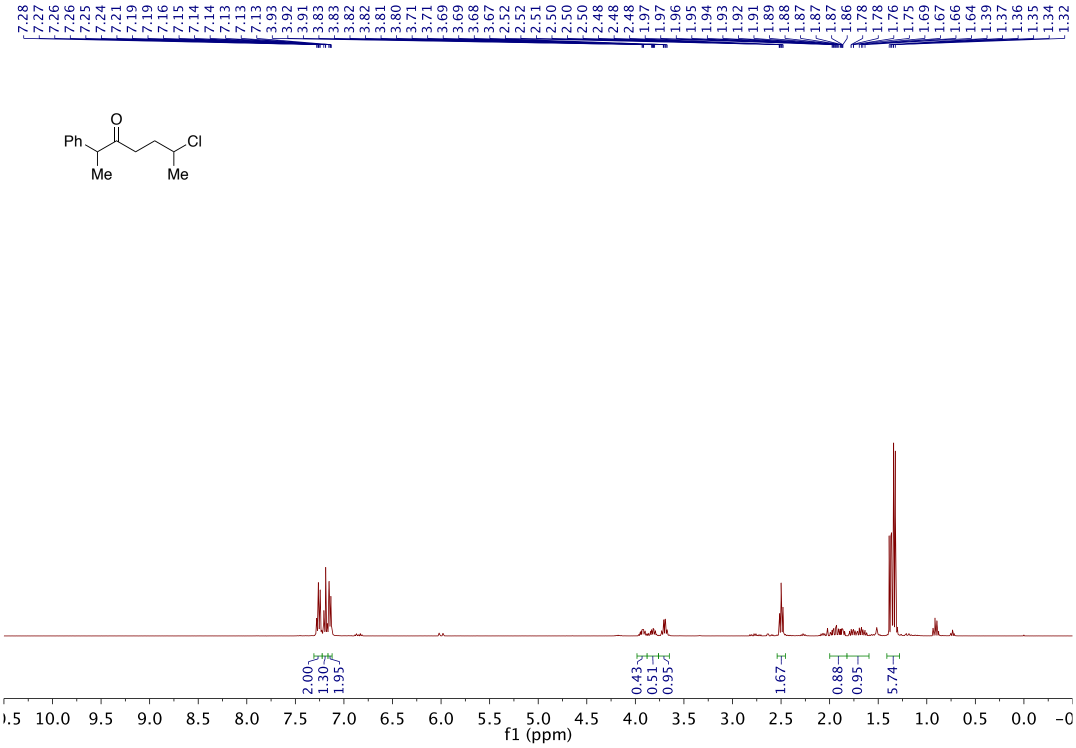

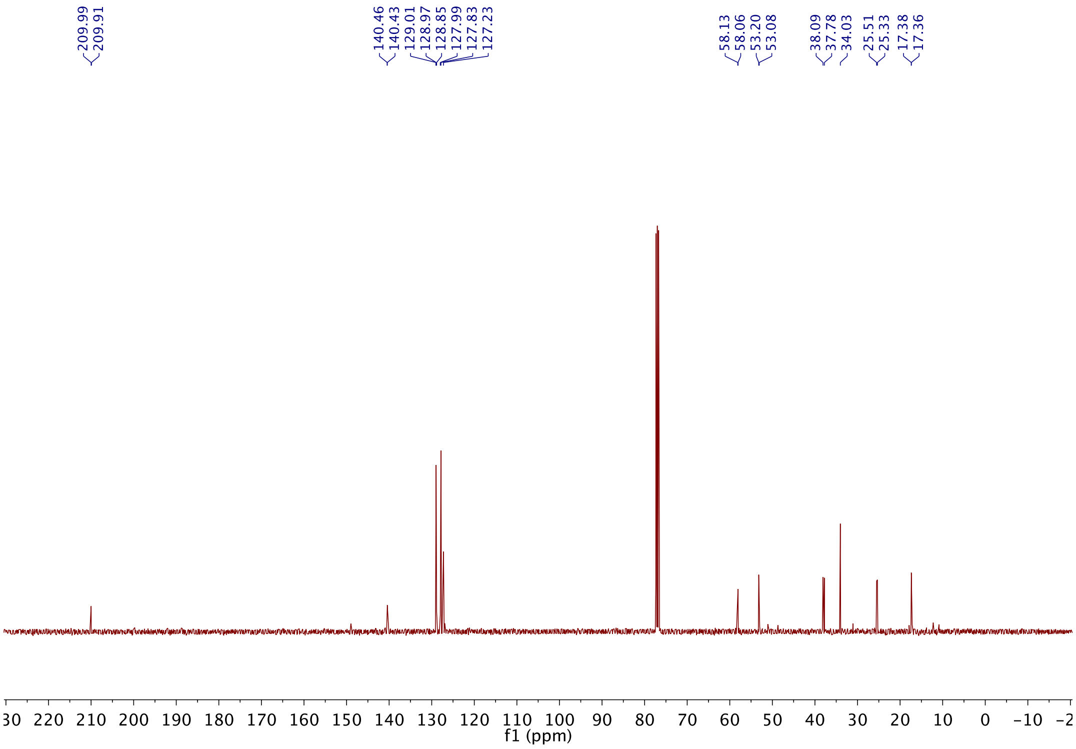

^{1}H and ^{13}C NMR spectra of phenyl 4-chloro-4-methylpentanoate (**2y**)

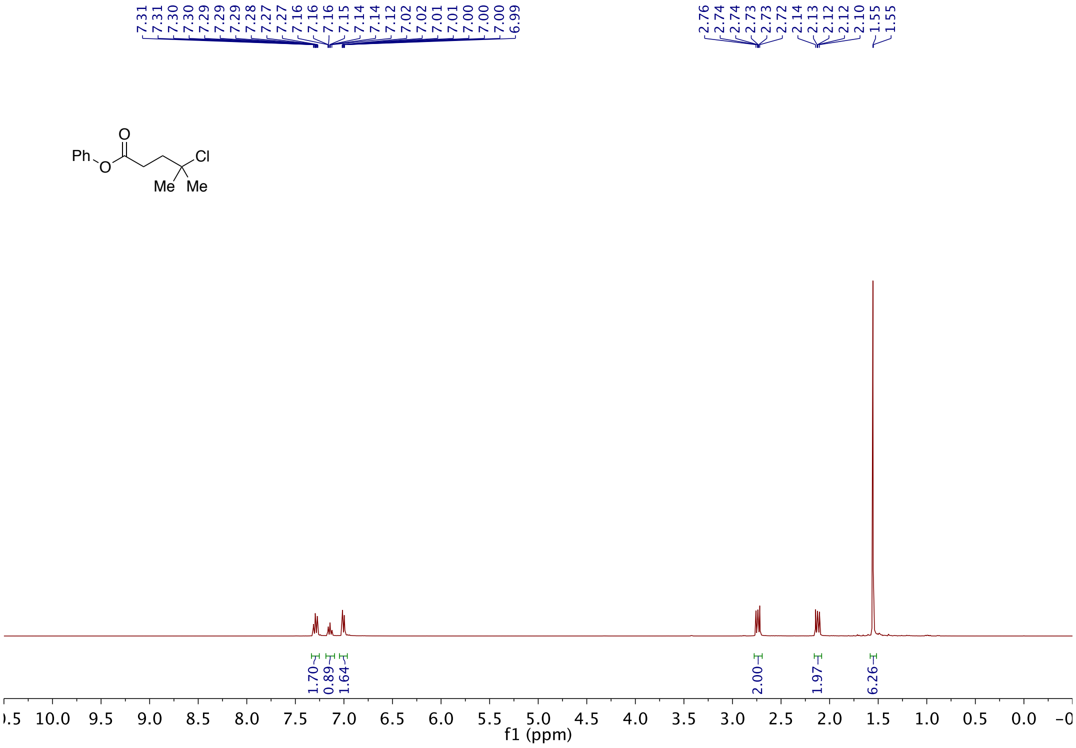

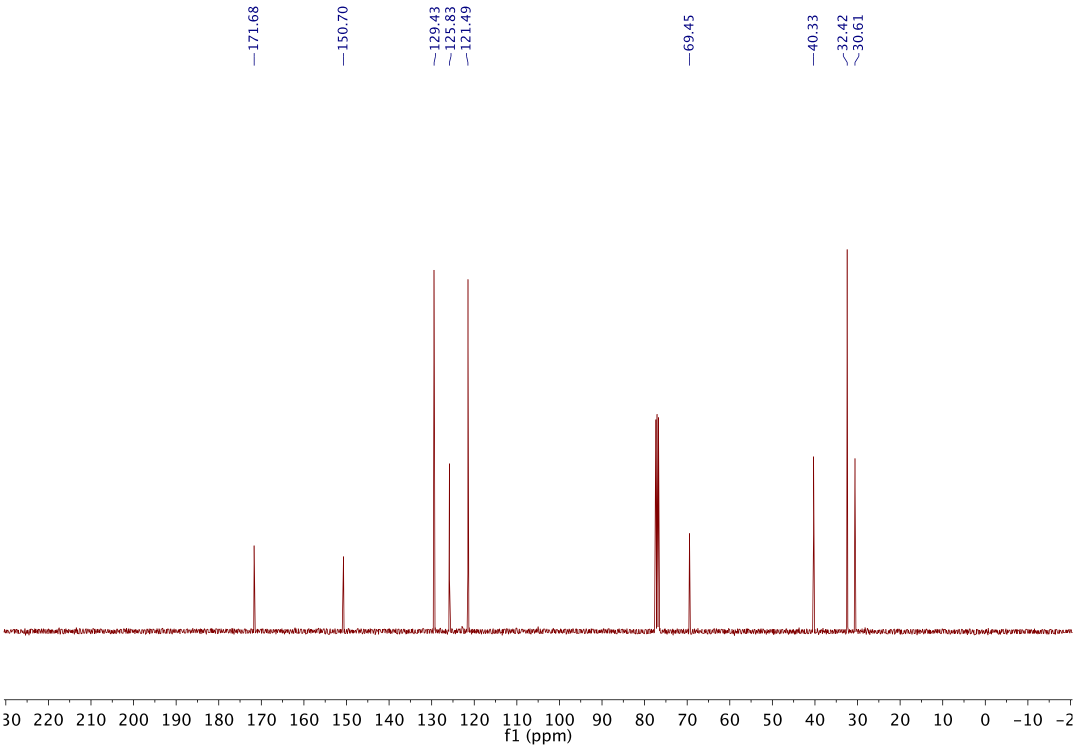

^{1}H and ^{13}C NMR spectra of 2-(1,3-dioxoisoindolin-2-yl)ethyl 4-chloro-4-methylpentanoate (**2z**)

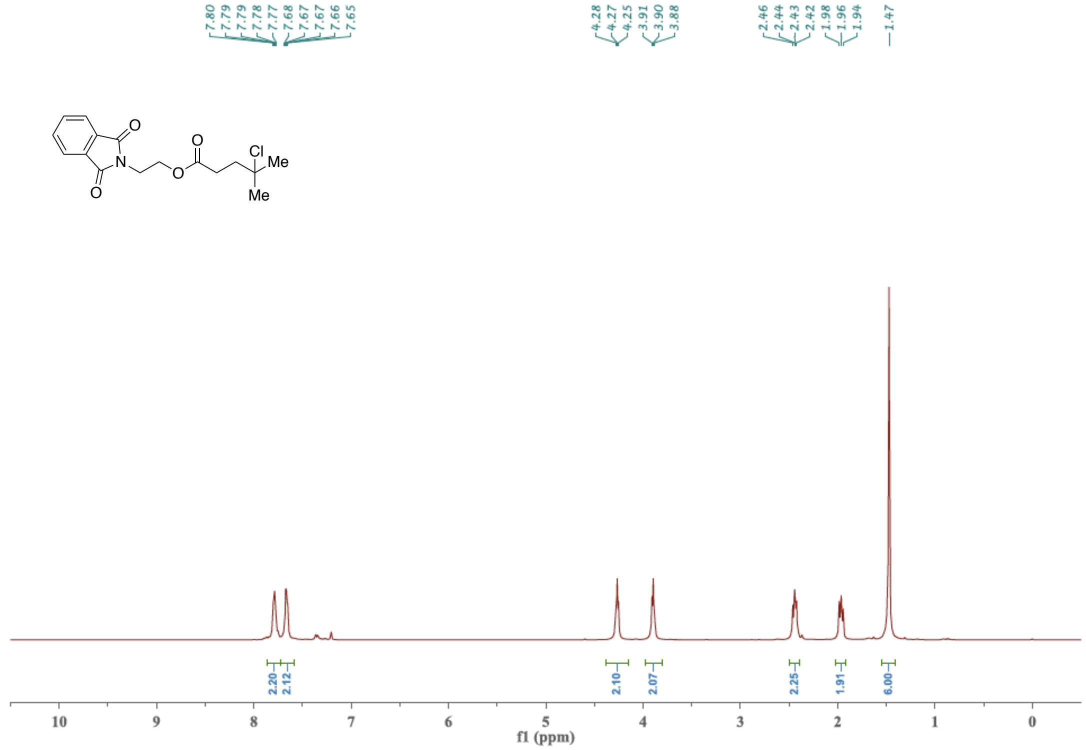

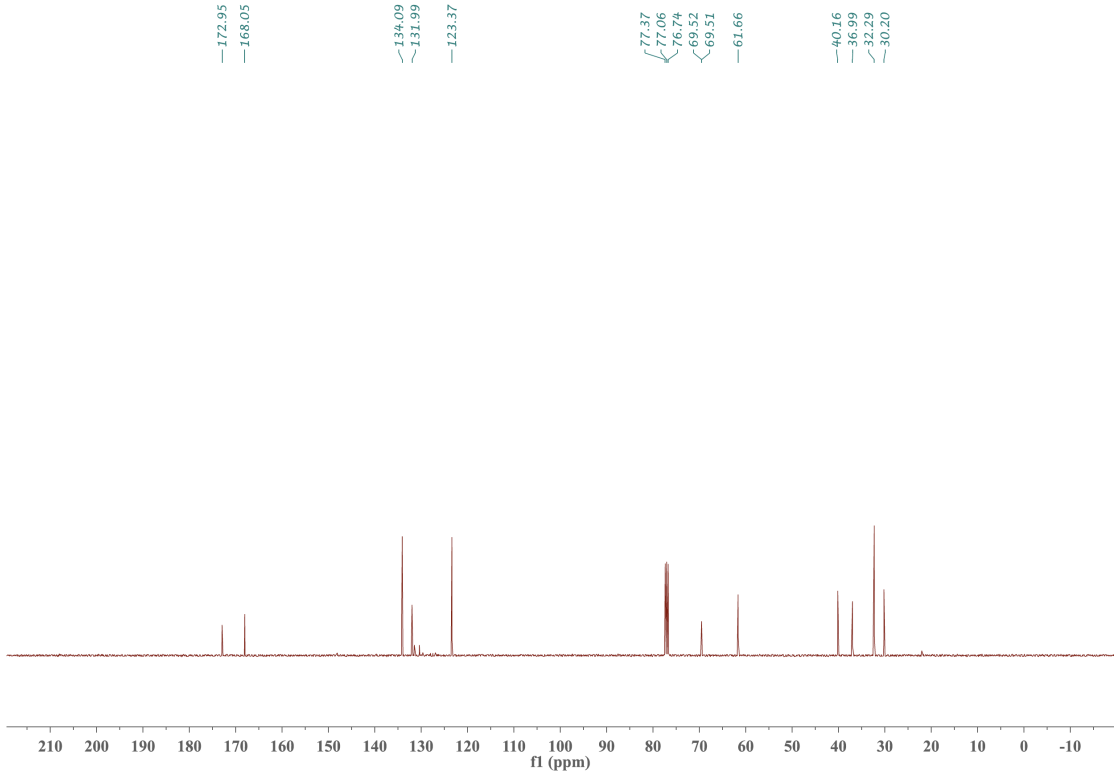

^{1}H and ^{13}C NMR spectra of 4-chloro-*N*,4-dimethyl-*N*-phenylpentanamide (**2α**)

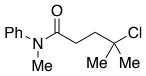

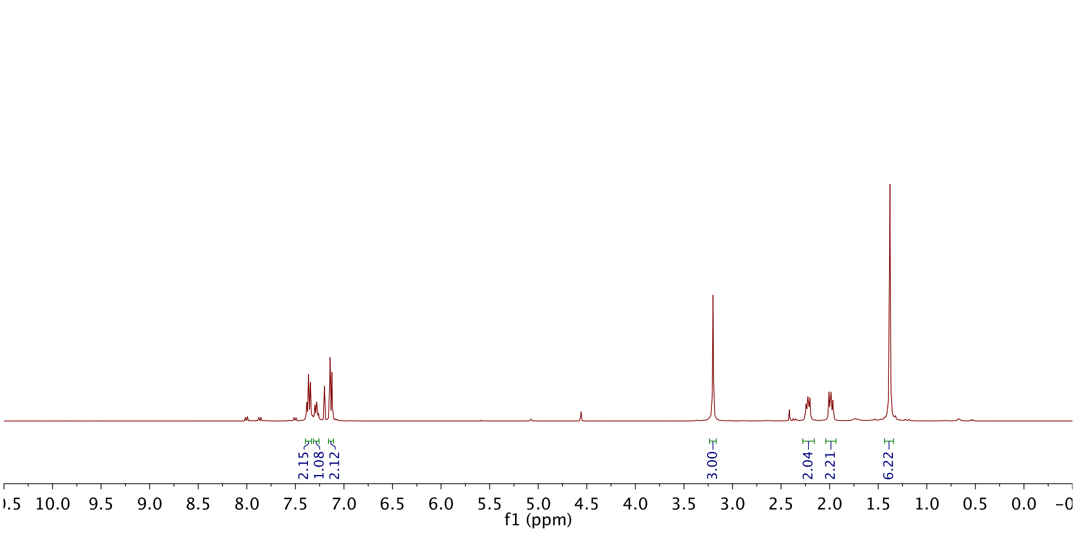

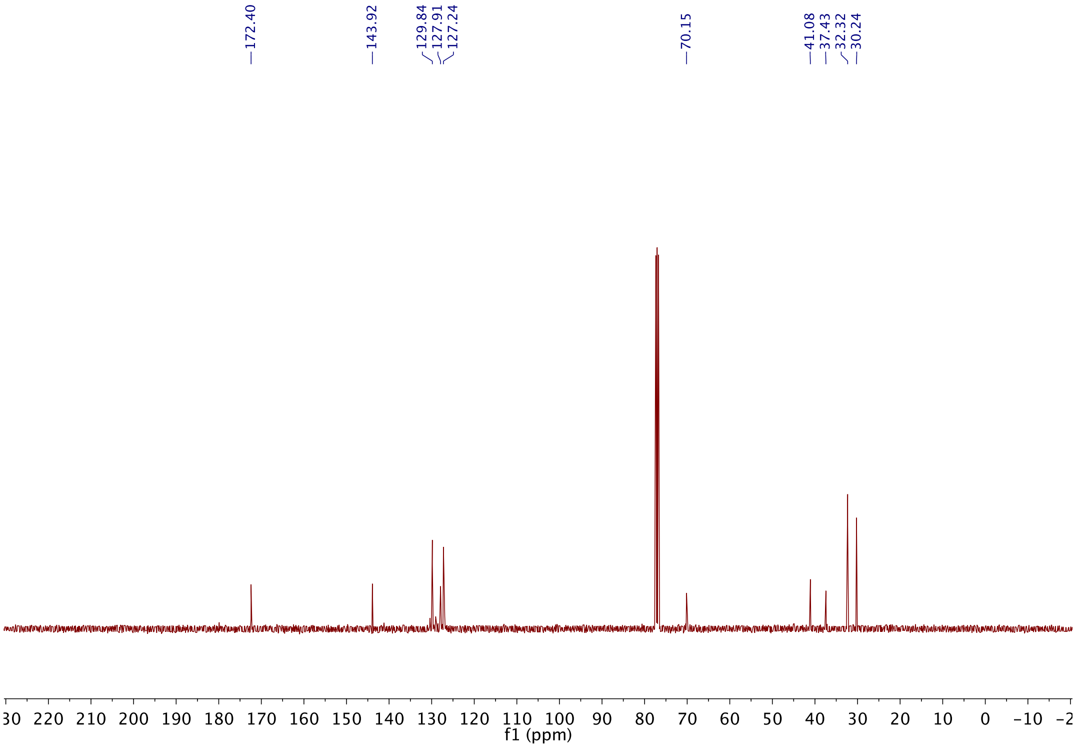

^{1}H and ^{13}C NMR spectra of (*E*)-4-chloro-4-methyl-1-phenylpent-2-en-1-one (**4a**)

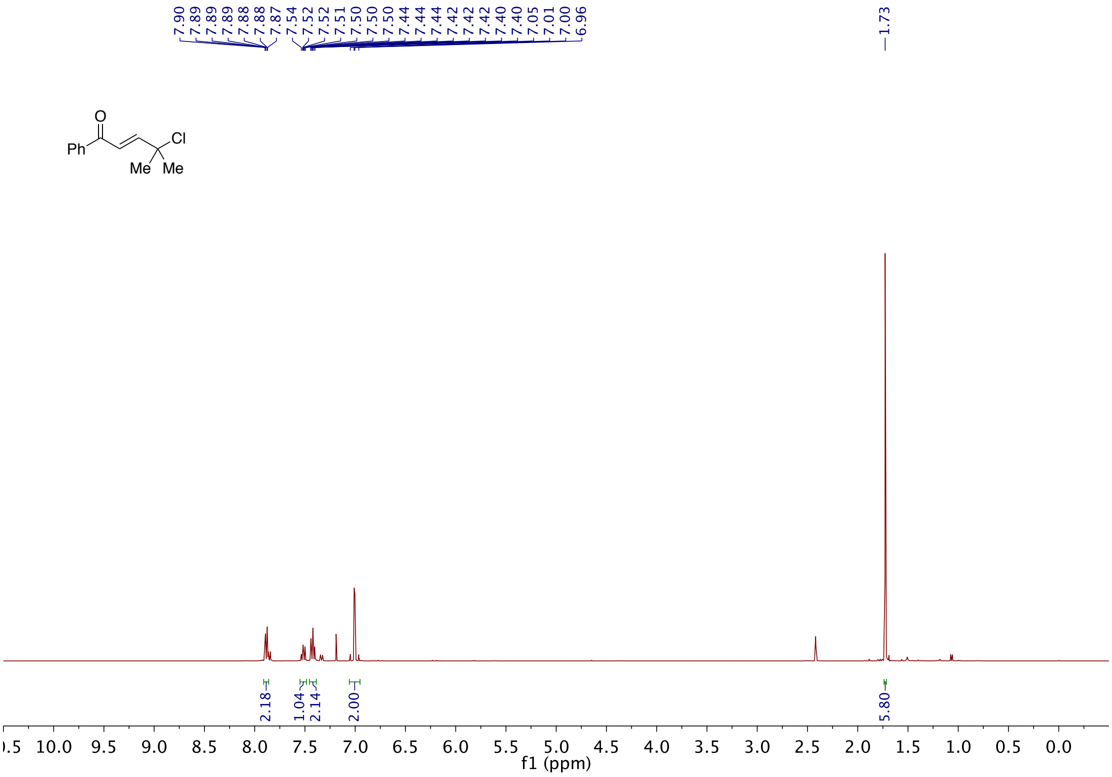

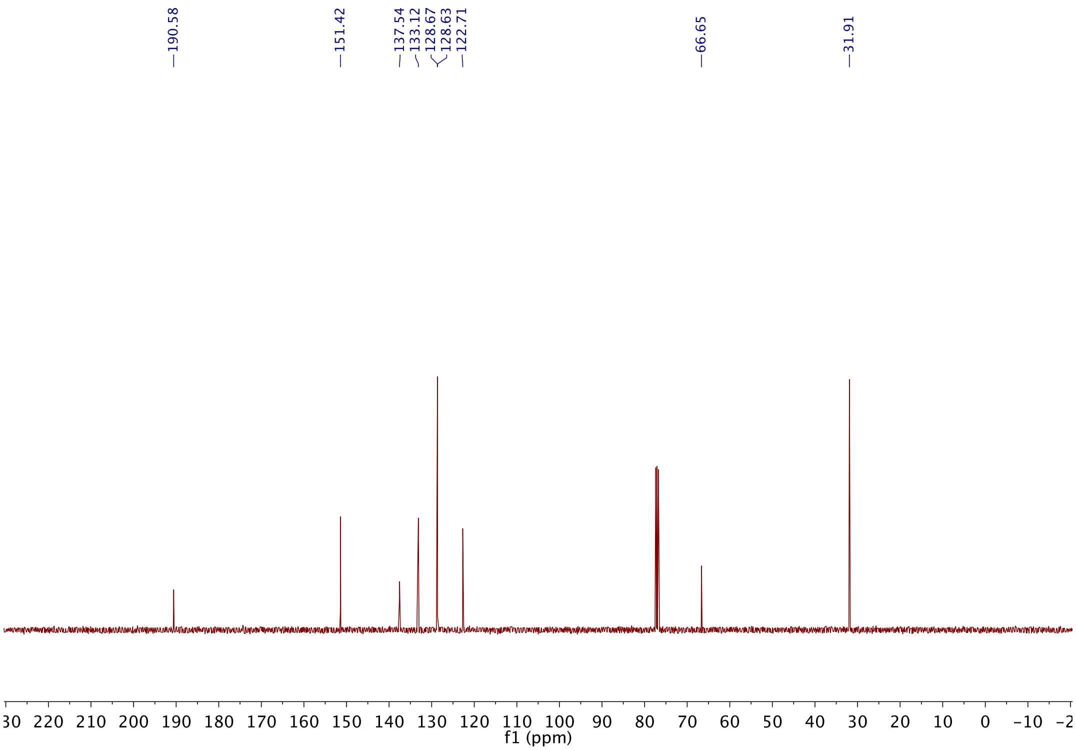

^{1}H and ^{13}C NMR spectra of 4-chloro-*N*,4-dimethyl-*N*-phenylpentanamide (**4b**)

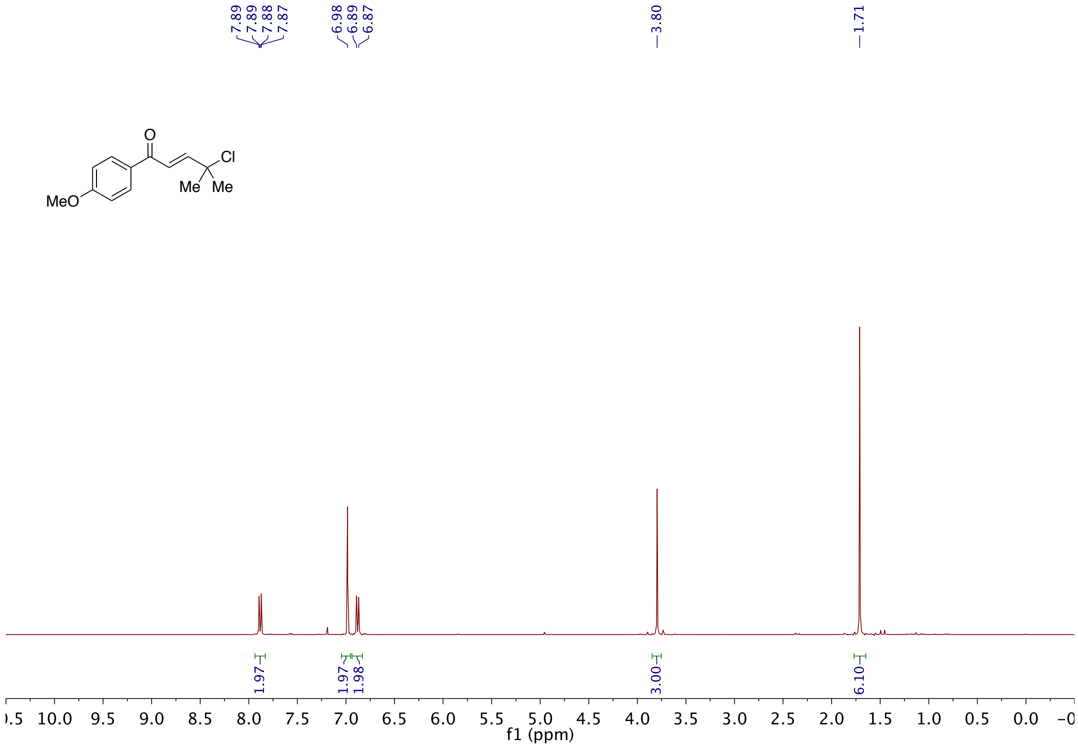

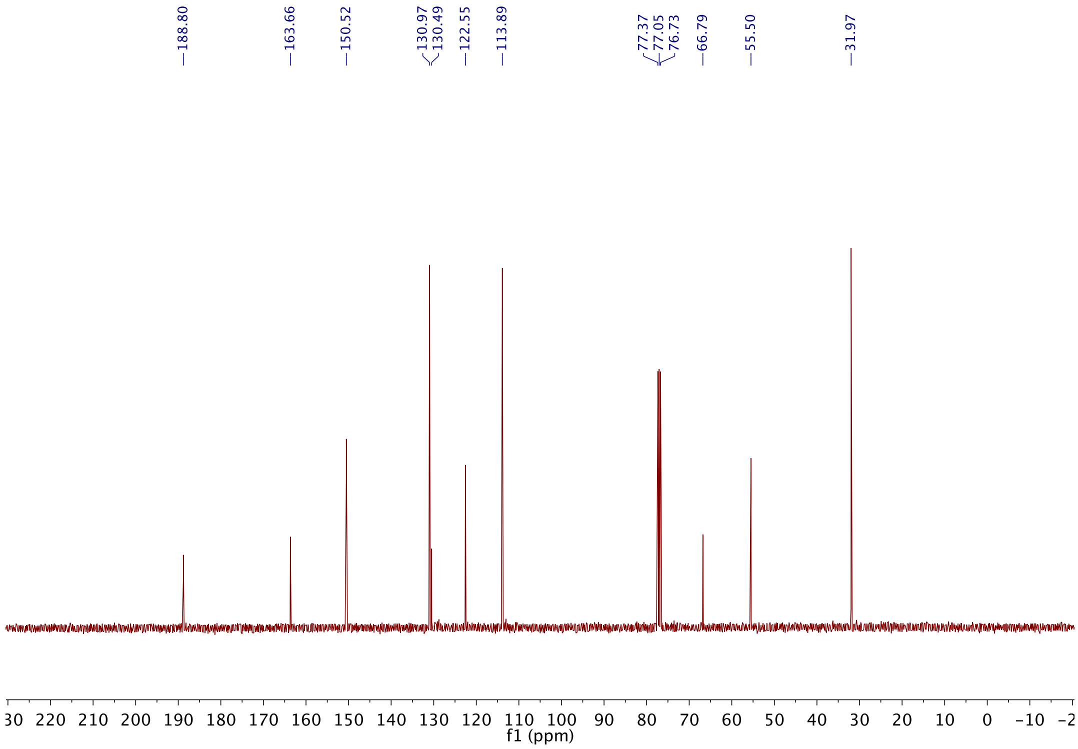

^{1}H and ^{13}C NMR spectra of (*E*)-4-chloro-4-methyl-1-(*p*-tolyl)pent-2-en-1-one (**4c**)

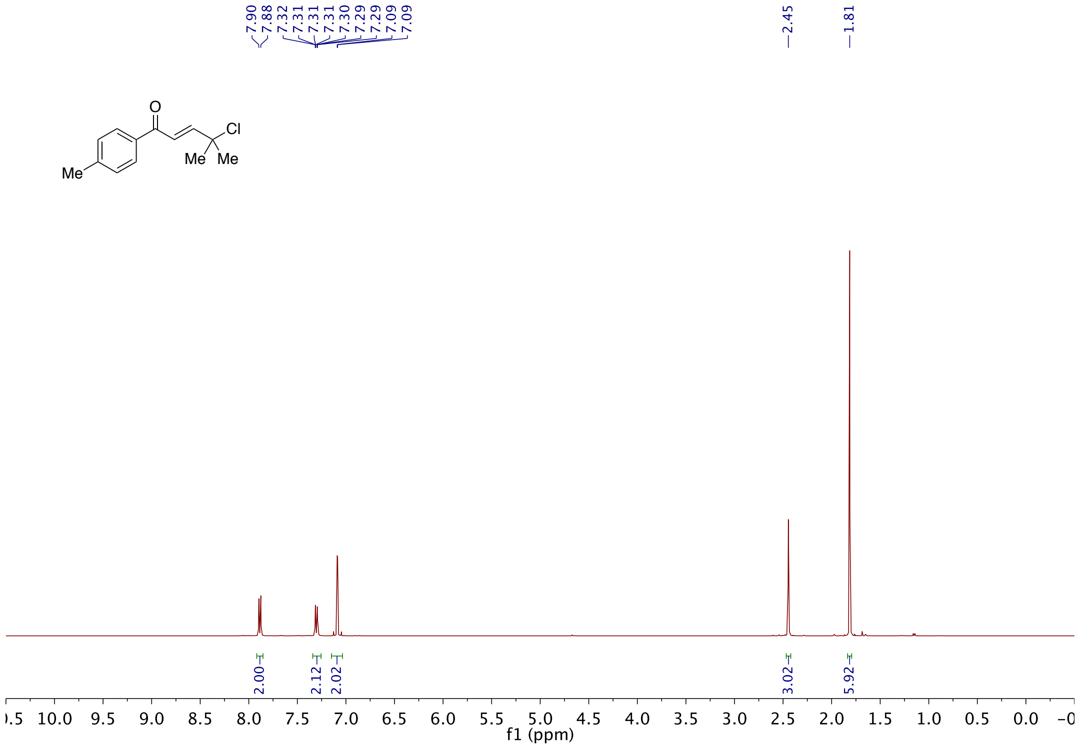

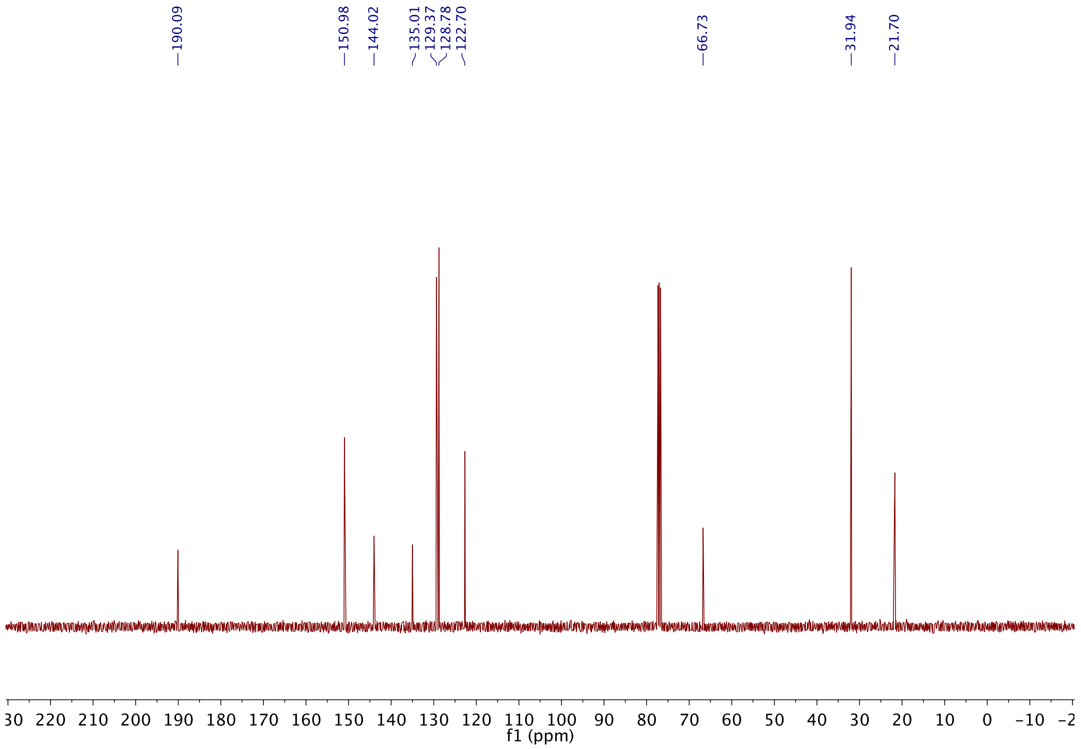

^{1}H and ^{13}C NMR spectra of (*E*)-1-([1,1'-biphenyl]-4-yl)-4-chloro-4-methylpent-2-en-1-one (**4d**)

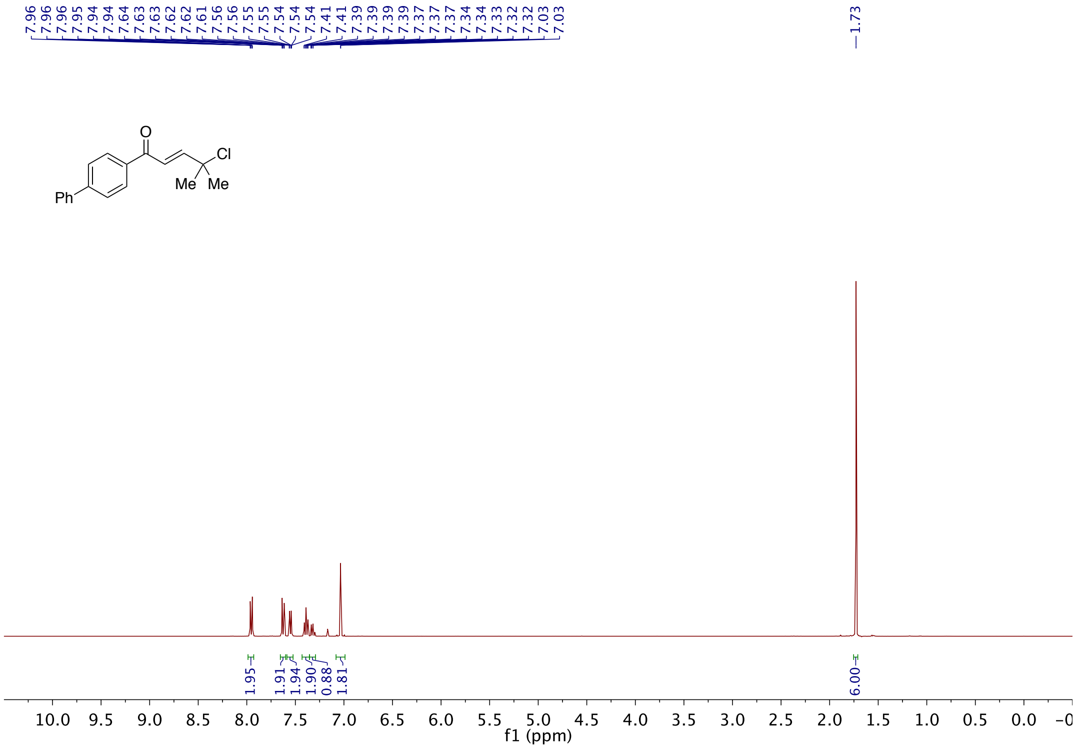

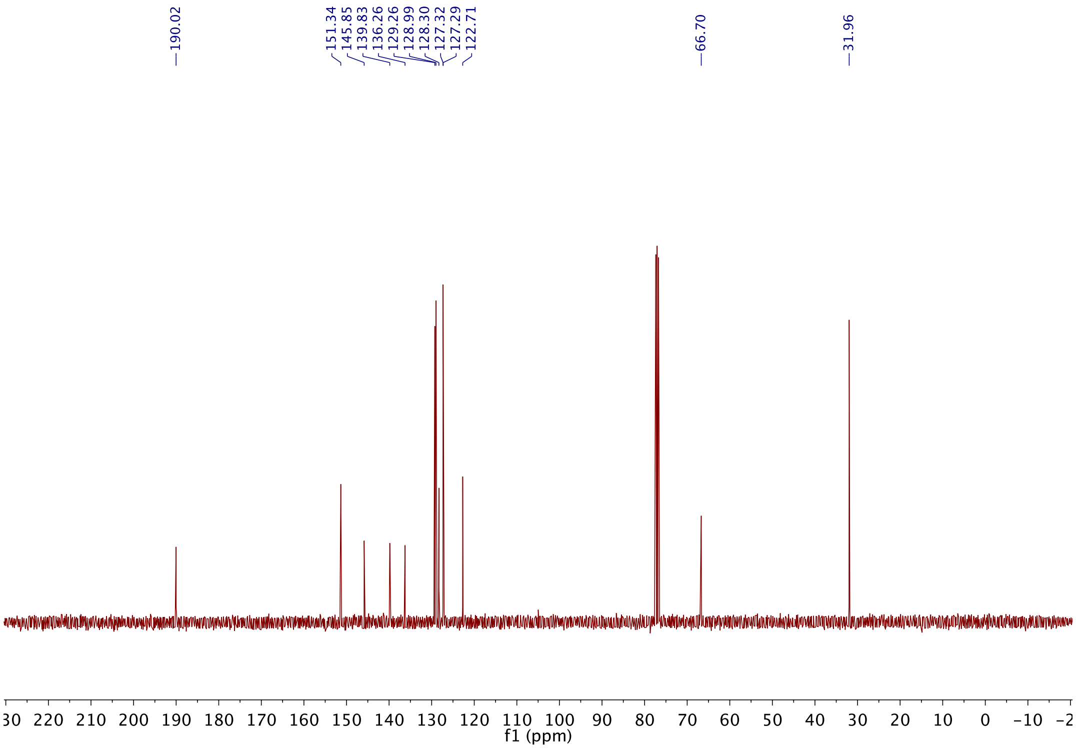

^{1}H and ^{13}C NMR spectra of (*E*)-4-chloro-4-methyl-1-(4-(trifluoromethyl)phenyl)pent-2-en-1-one (**4e**)

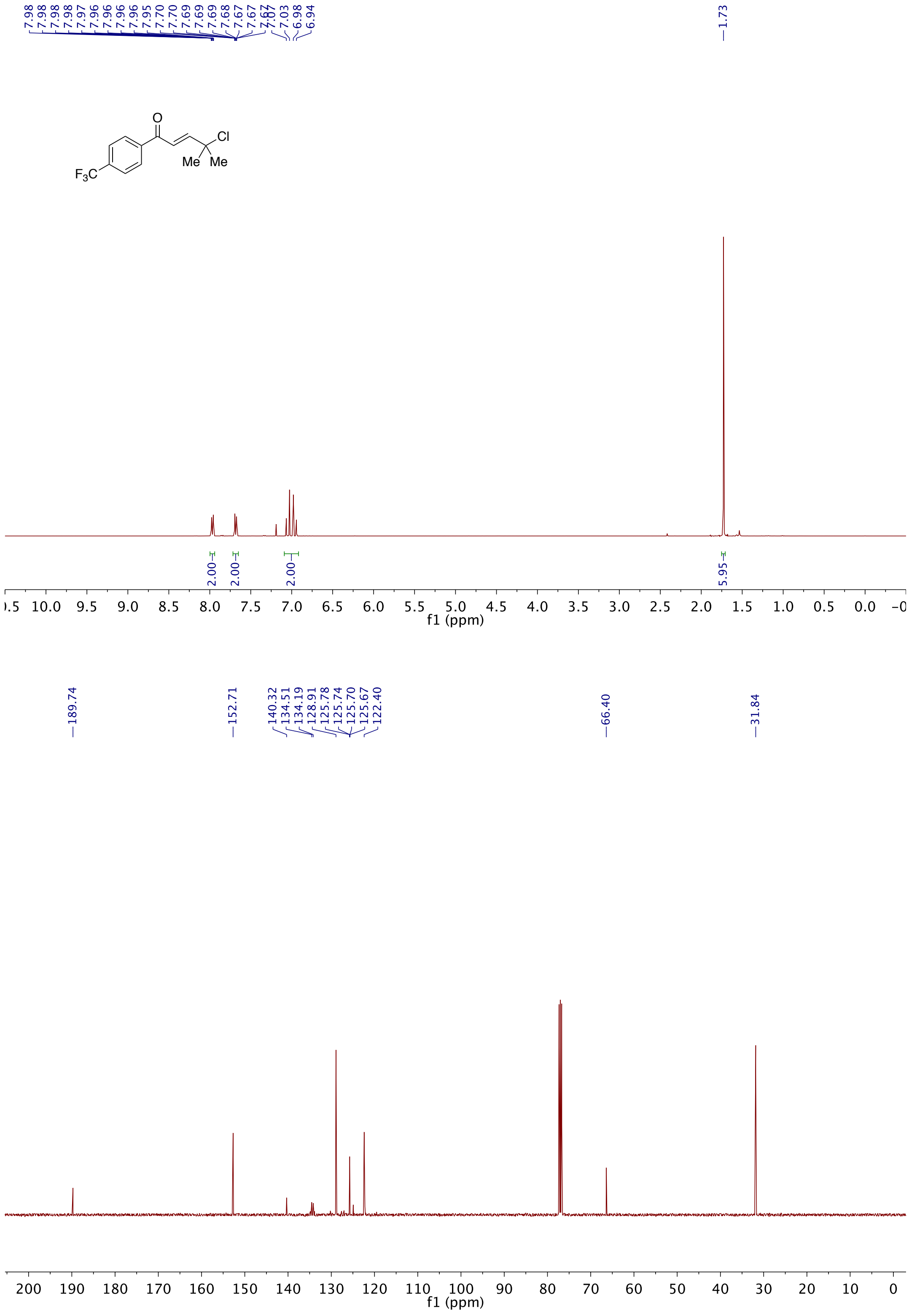

^{1}H and ^{13}C NMR spectra of (*E*)-4-chloro-1-(2,4-dimethylphenyl)-4-methylpent-2-en-1-one (**4f**)

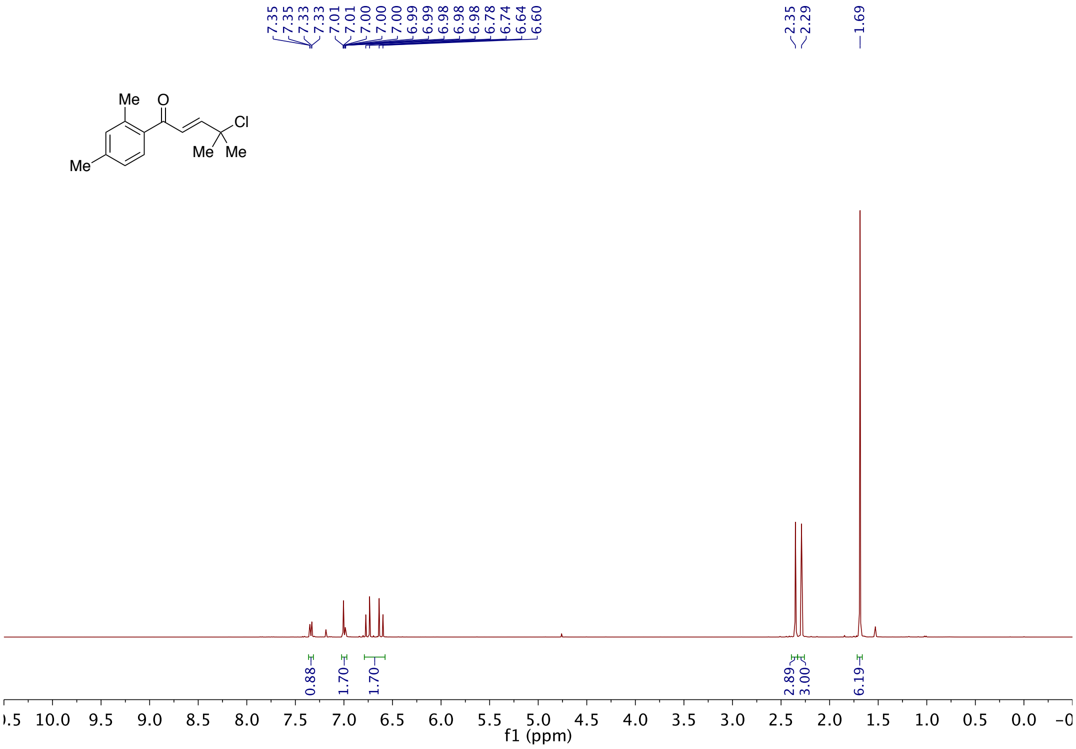

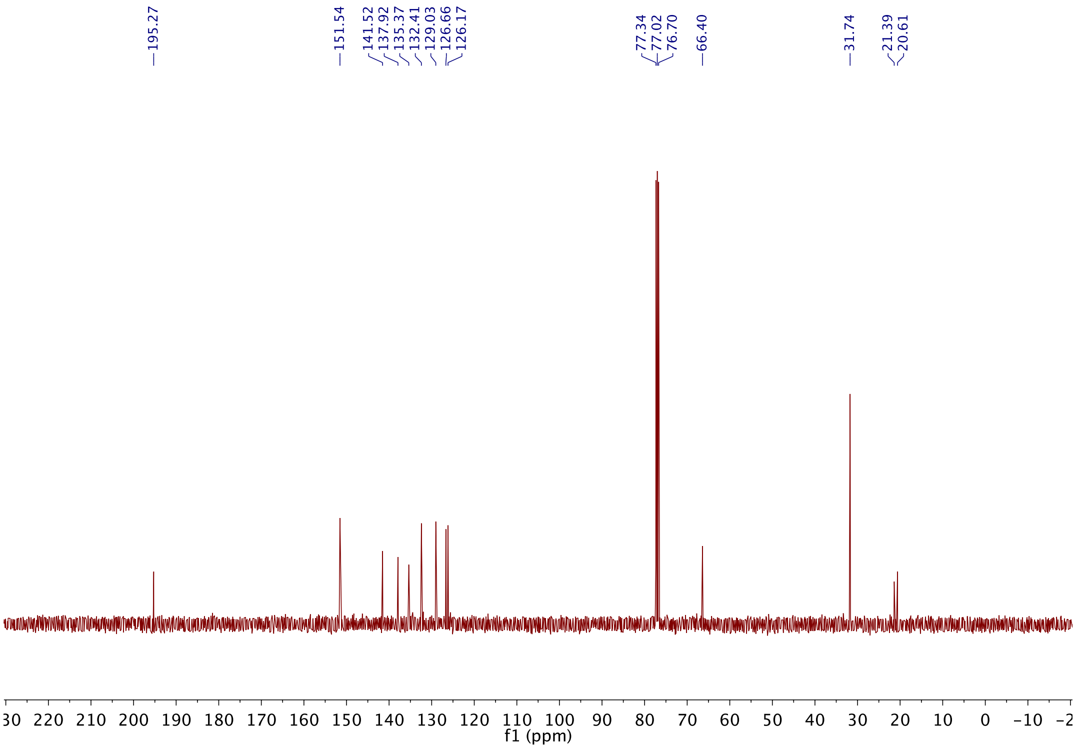

^{1}H and ^{13}C NMR spectra of (*E*)-4-chloro-4-methyl-1-(naphthalen-2-yl)pent-2-en-1-one (**4g**)

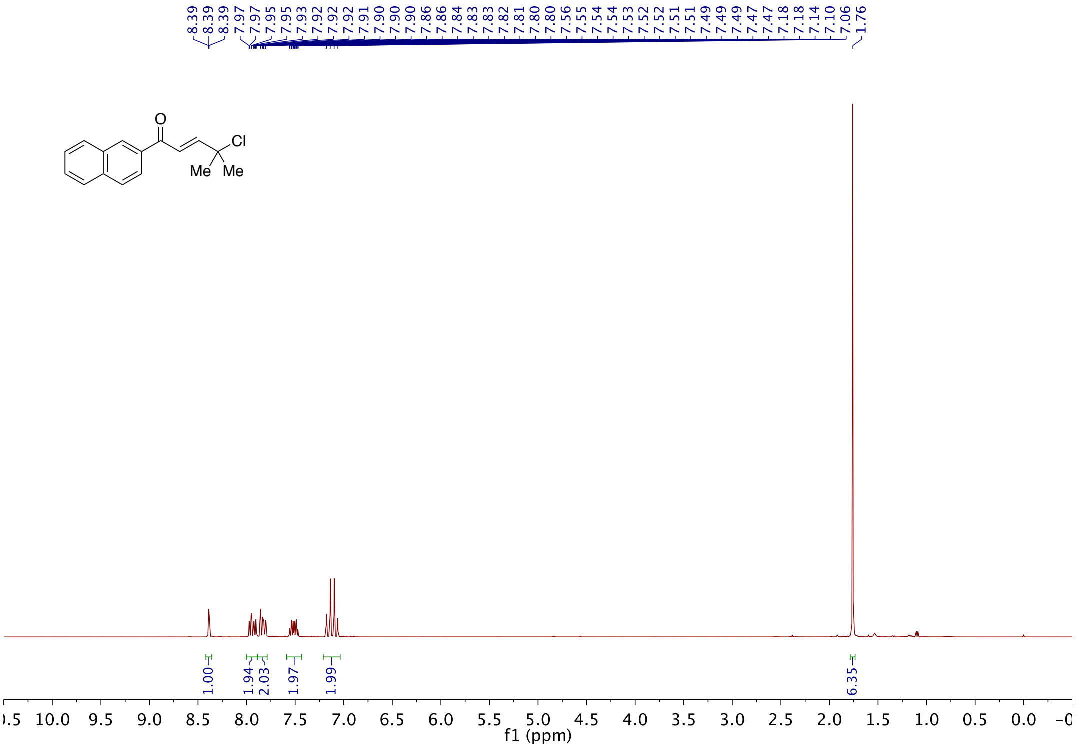

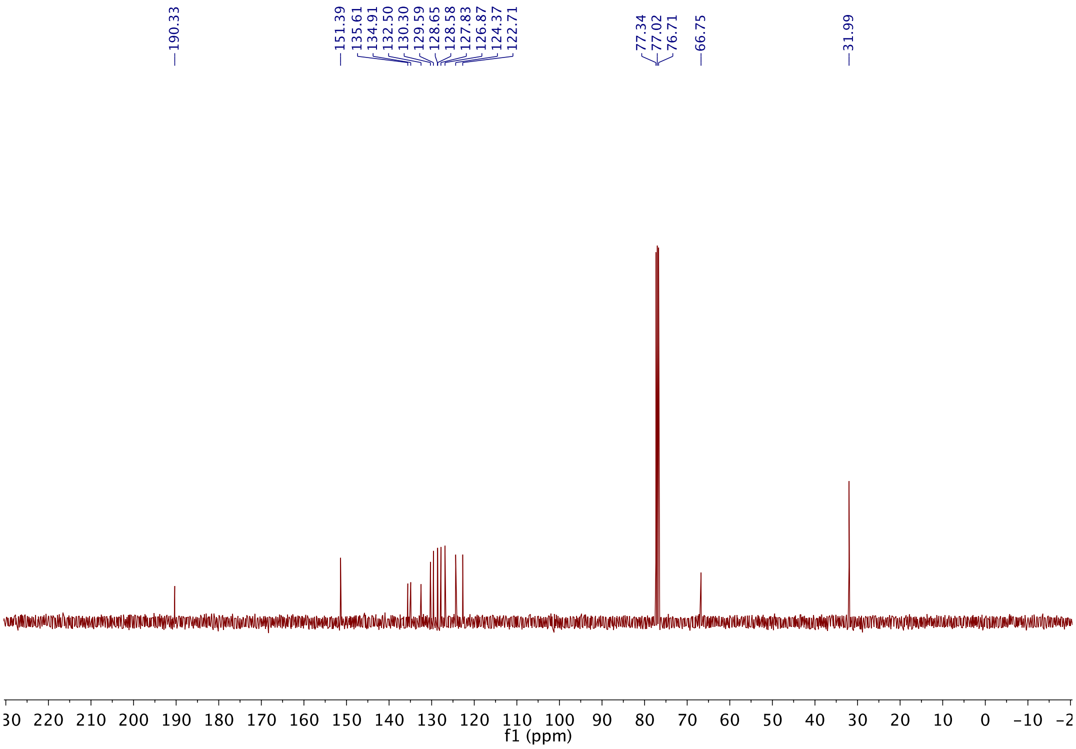

^{1}H and ^{13}C NMR spectra of (*E*)-4-chloro-4-methyl-1-(thiophen-2-yl)pent-2-en-1-one (**4h**)

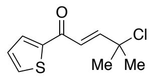

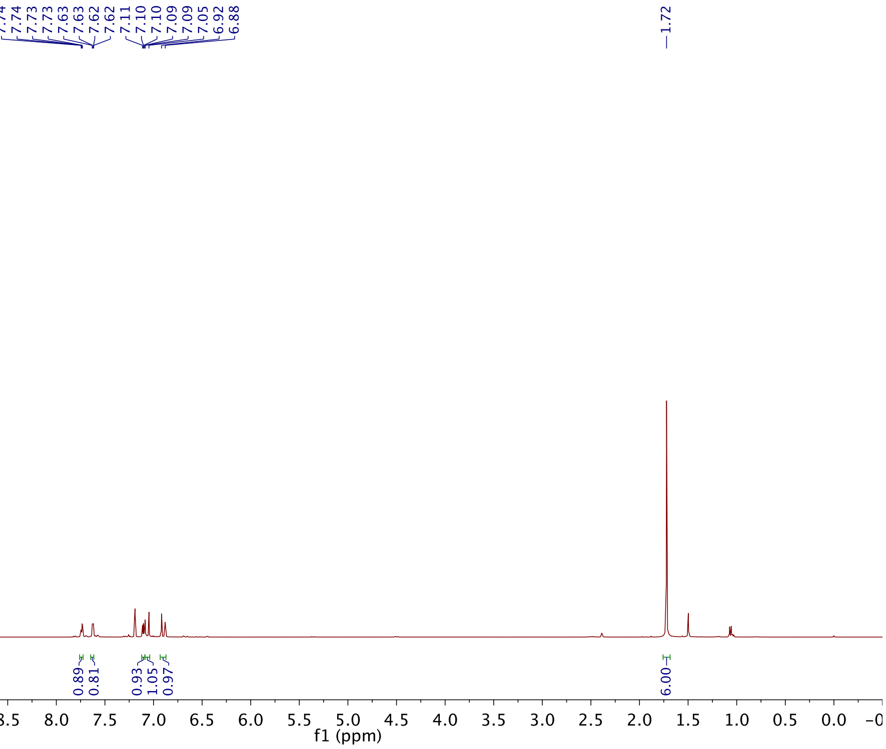

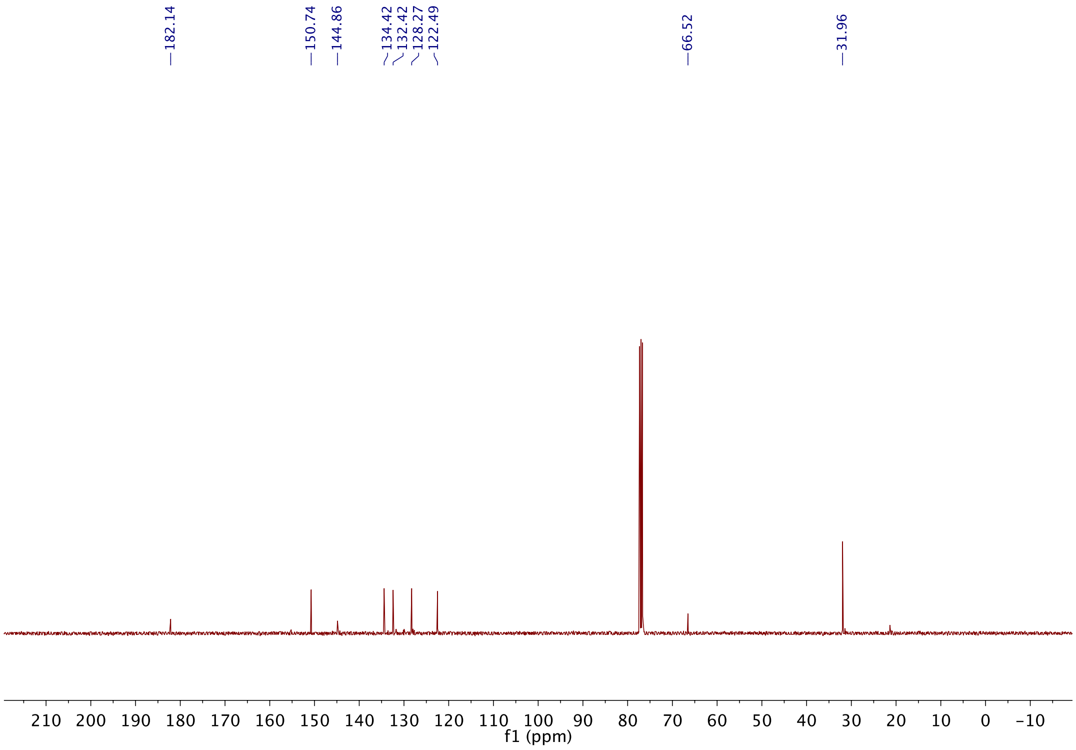

^{1}H and ^{13}C NMR spectra of (*E*)-4-chloro-4-methyl-1-phenylhex-2-en-1-one (**4i**)

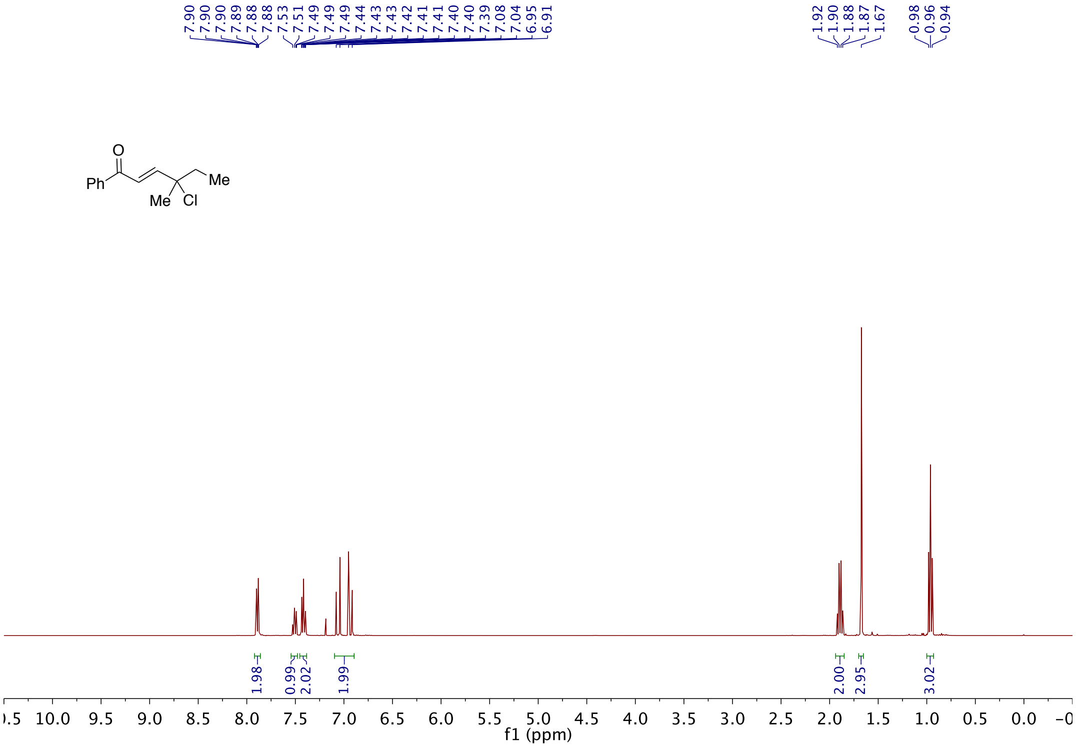

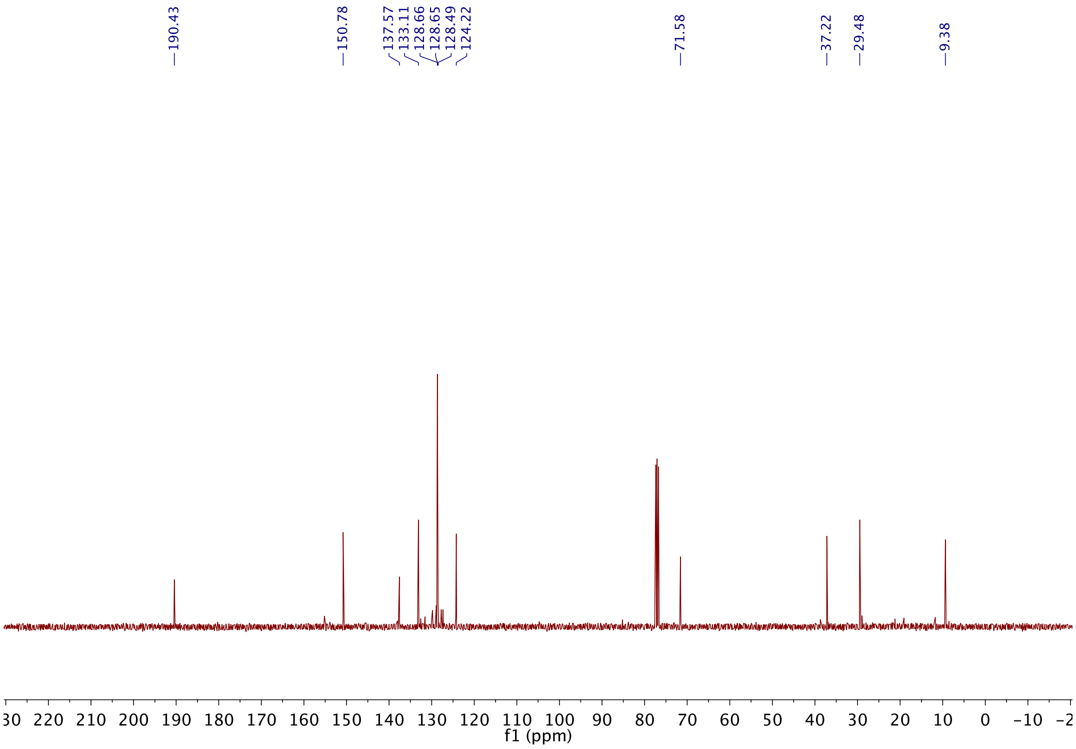

^{1}H and ^{13}C NMR spectra of (*E*)-4-chloro-4-ethyl-1-phenylhex-2-en-1-one (**4j**)

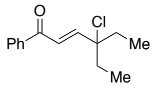

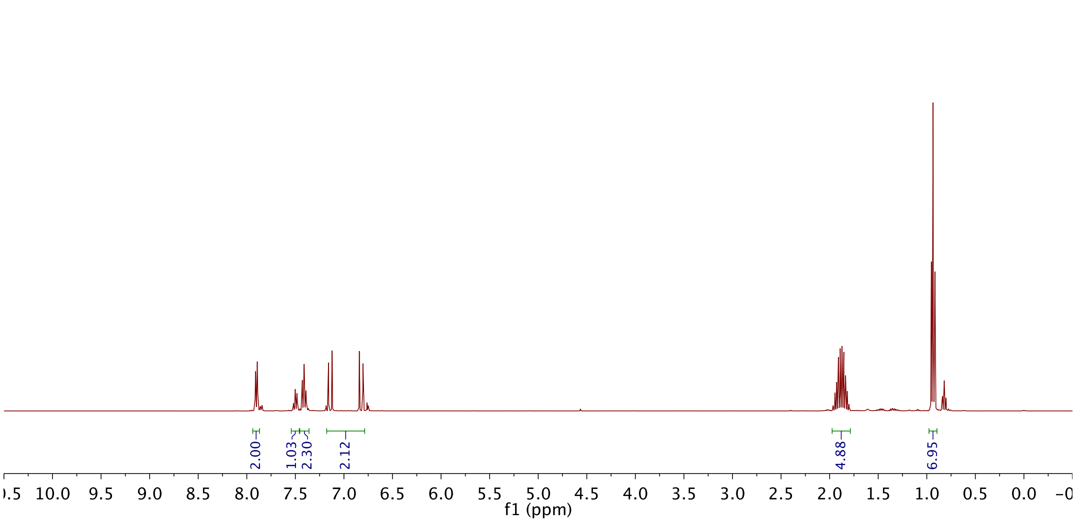

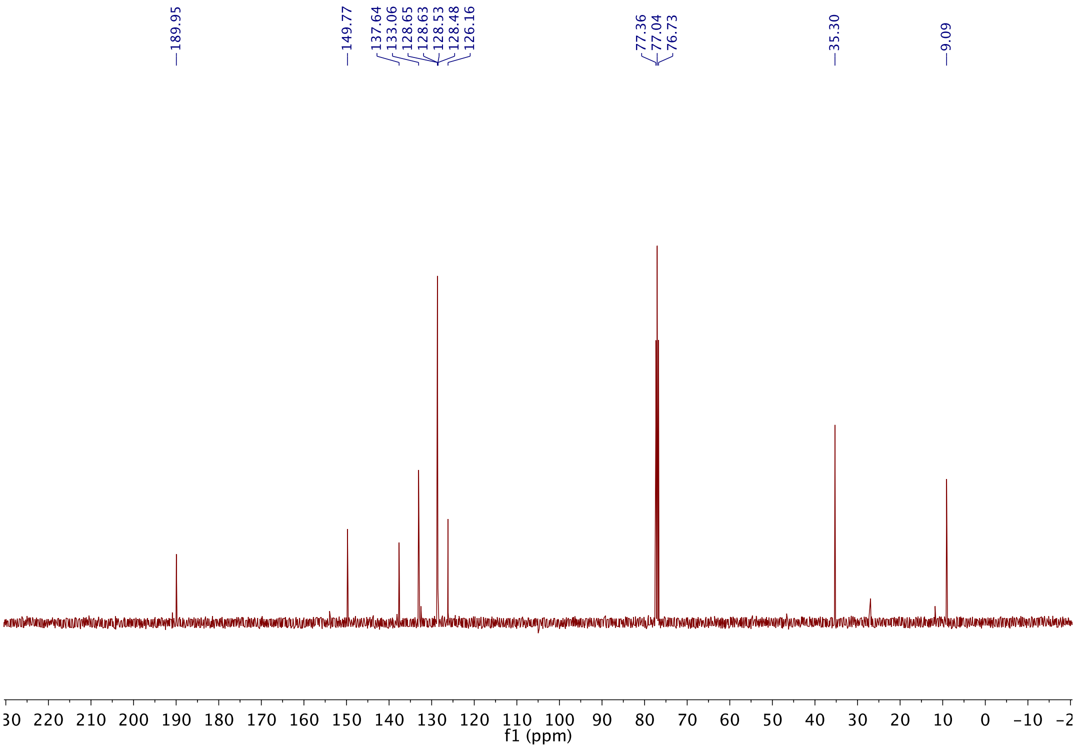

^{1}H and ^{13}C NMR spectra of (*E*)-3-(1-chlorocyclohexyl)-1-phenylprop-2-en-1-one (**4k**)

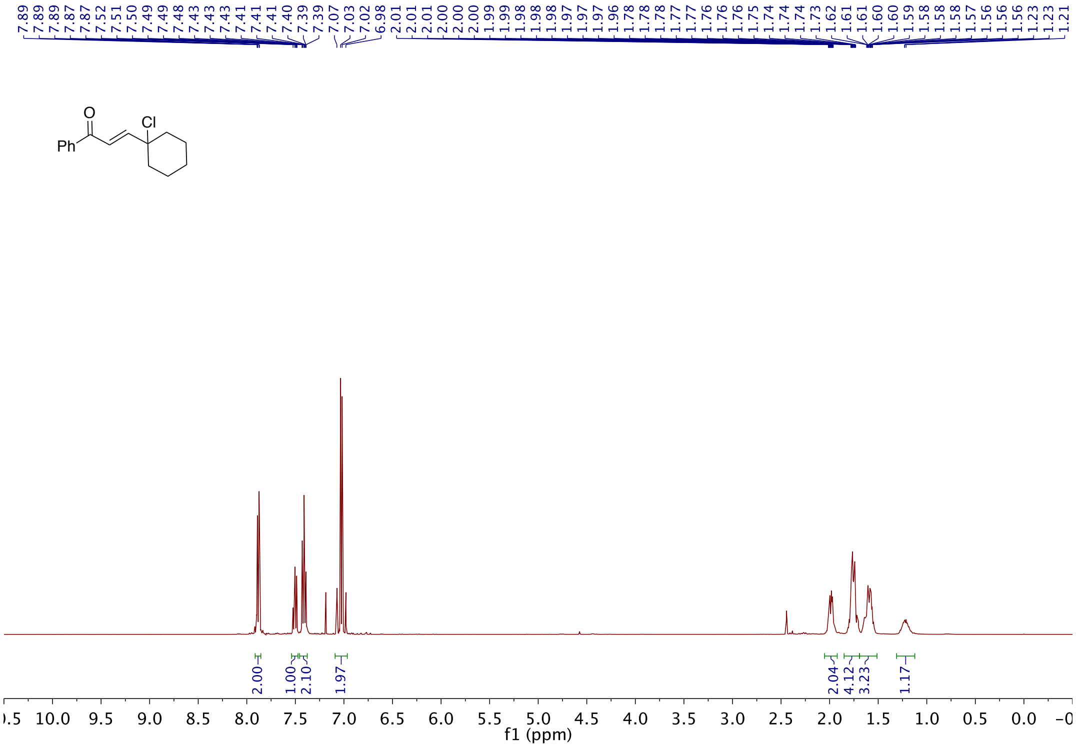

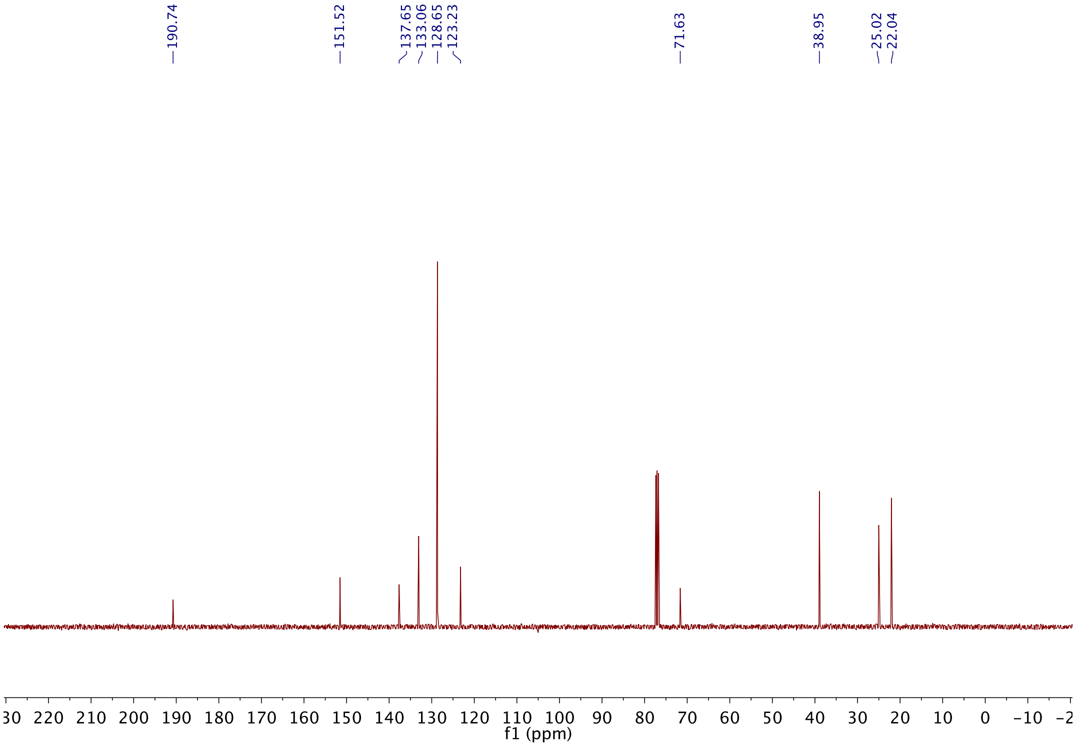

^{1}H NMR spectra of crude (chloromethyl)benzene (**6a**)

^{1}H NMR spectrum of reaction mixture (toluene + $TsNCl_2$)

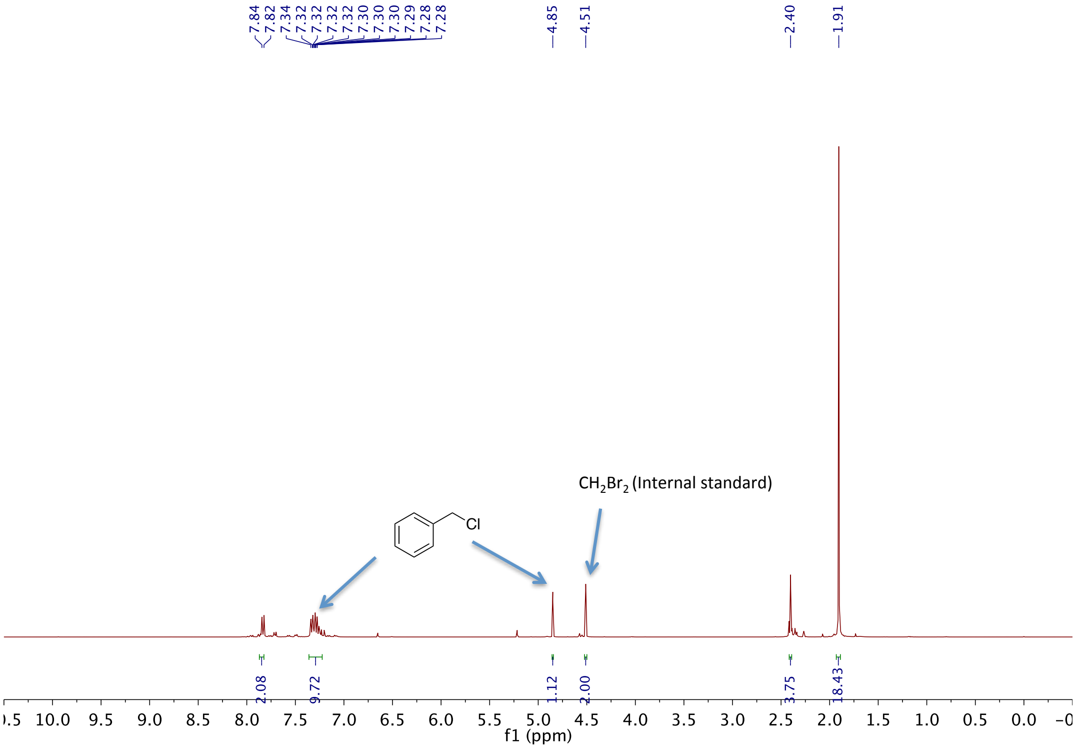

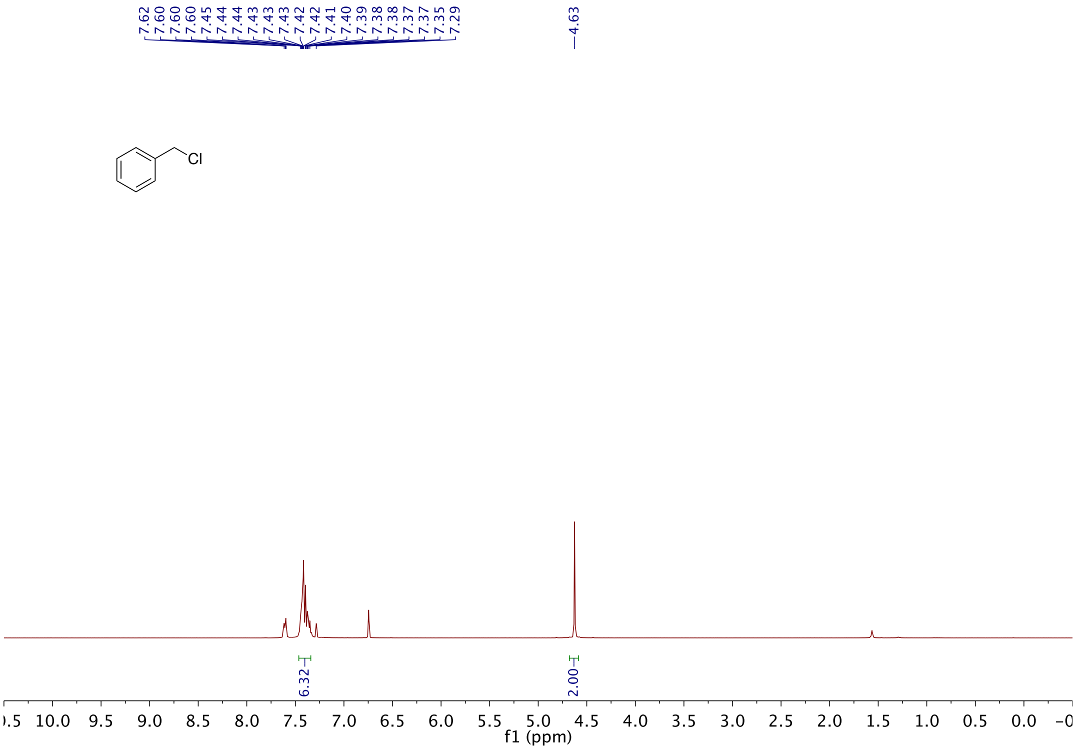

^{1}H and ^{13}C NMR spectra of 1-(3-(chloromethyl)phenyl)ethan-1-one (**6b**)

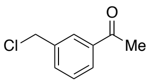

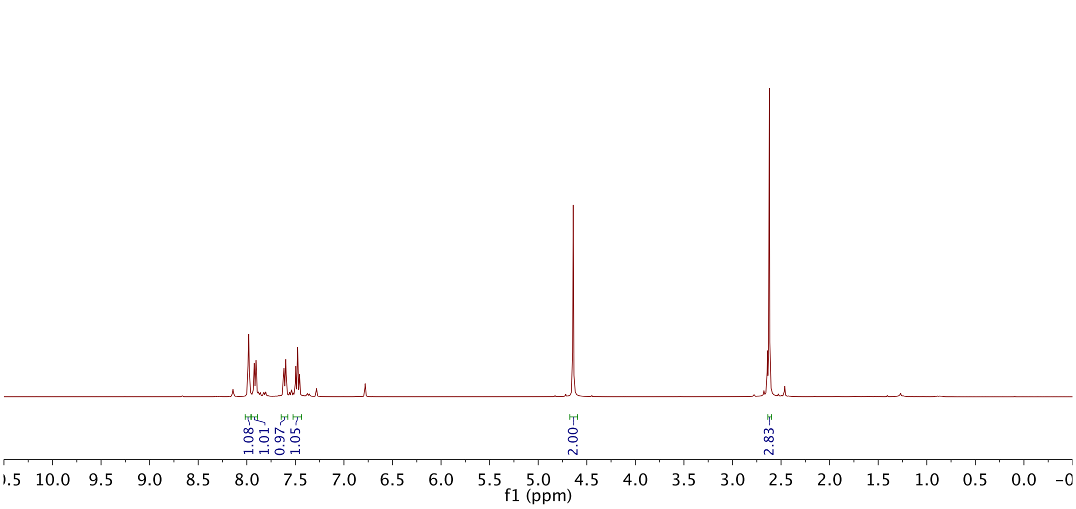

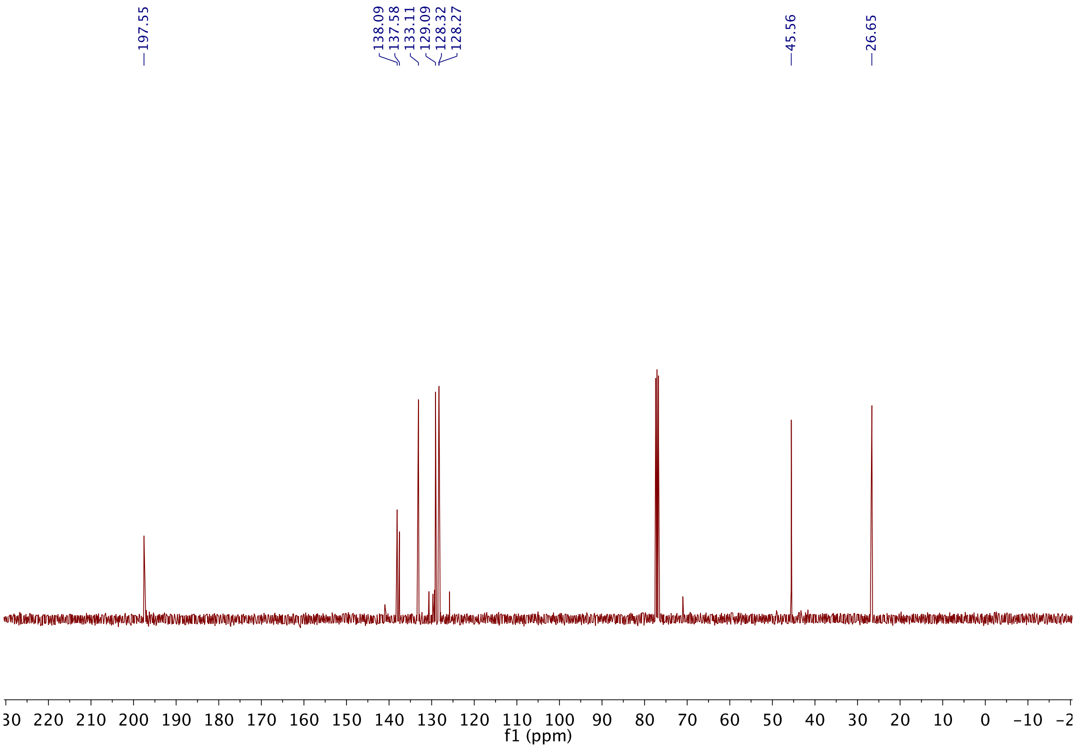

^{1}H and ^{13}C NMR spectra of 1-(4-(chloromethyl)phenyl)ethan-1-one (**6c**)

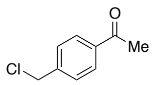

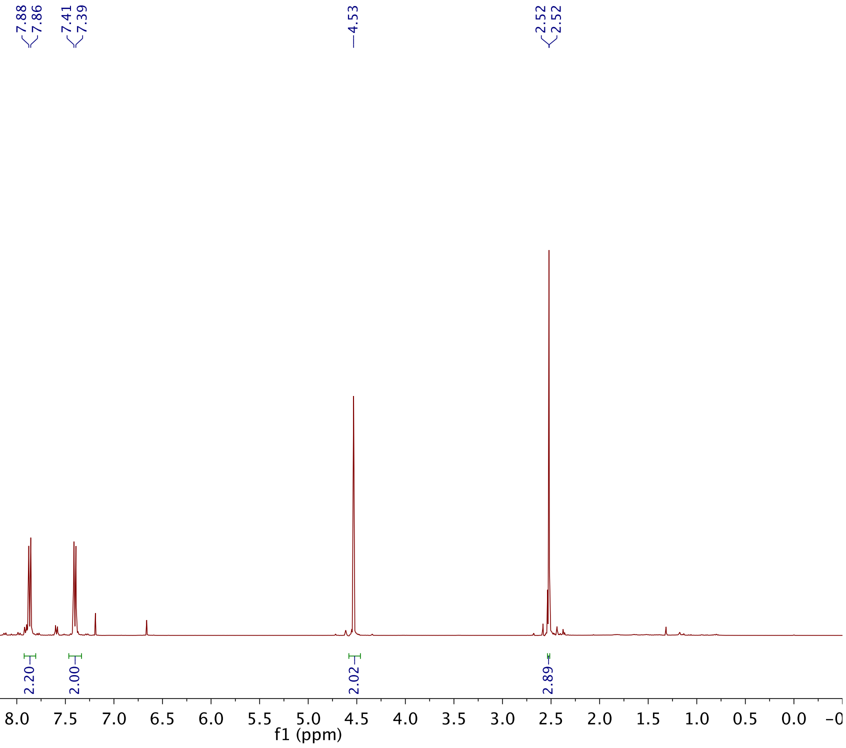

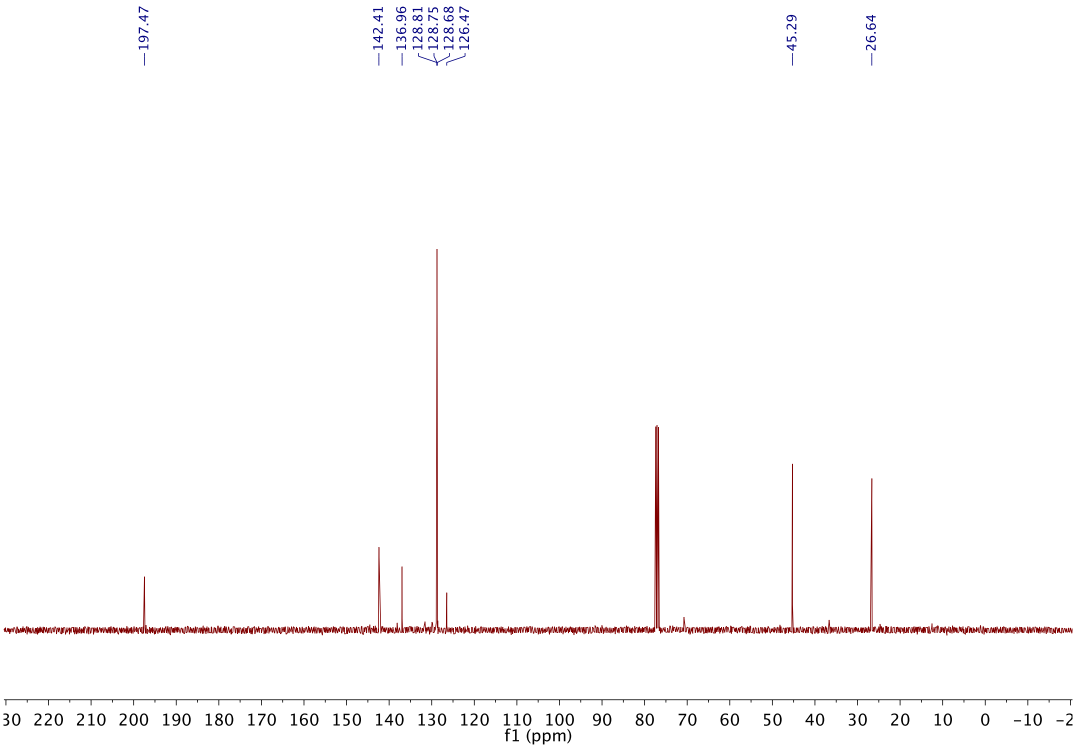

^{1}H and ^{13}C NMR spectra of (4-(chloromethyl)phenyl)(phenyl)methanone (**6d**)

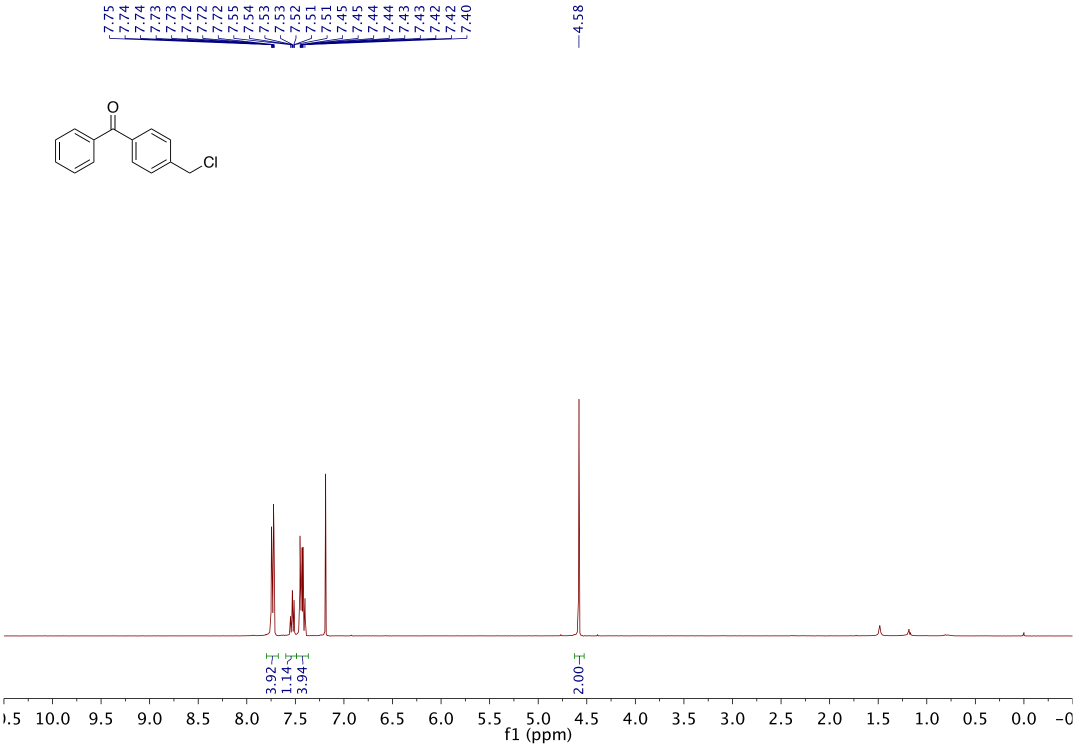

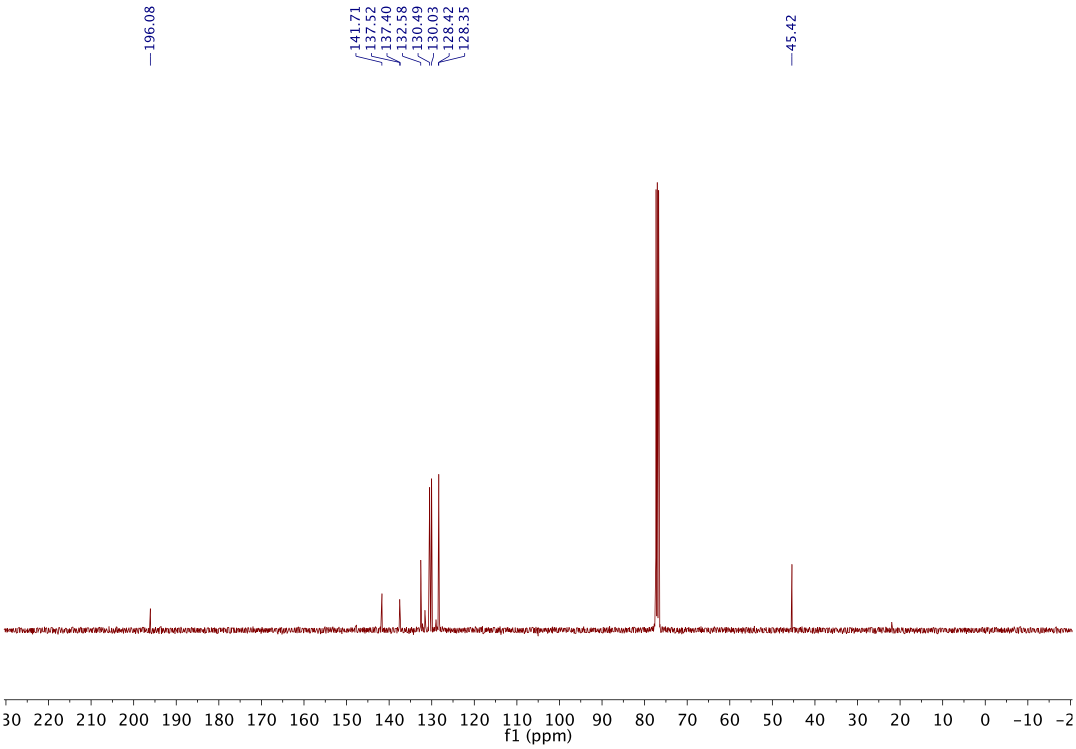

^{1}H and ^{13}C NMR spectra of 1-(4-(1-chloroethyl)phenyl)ethan-1-one (**6e**)

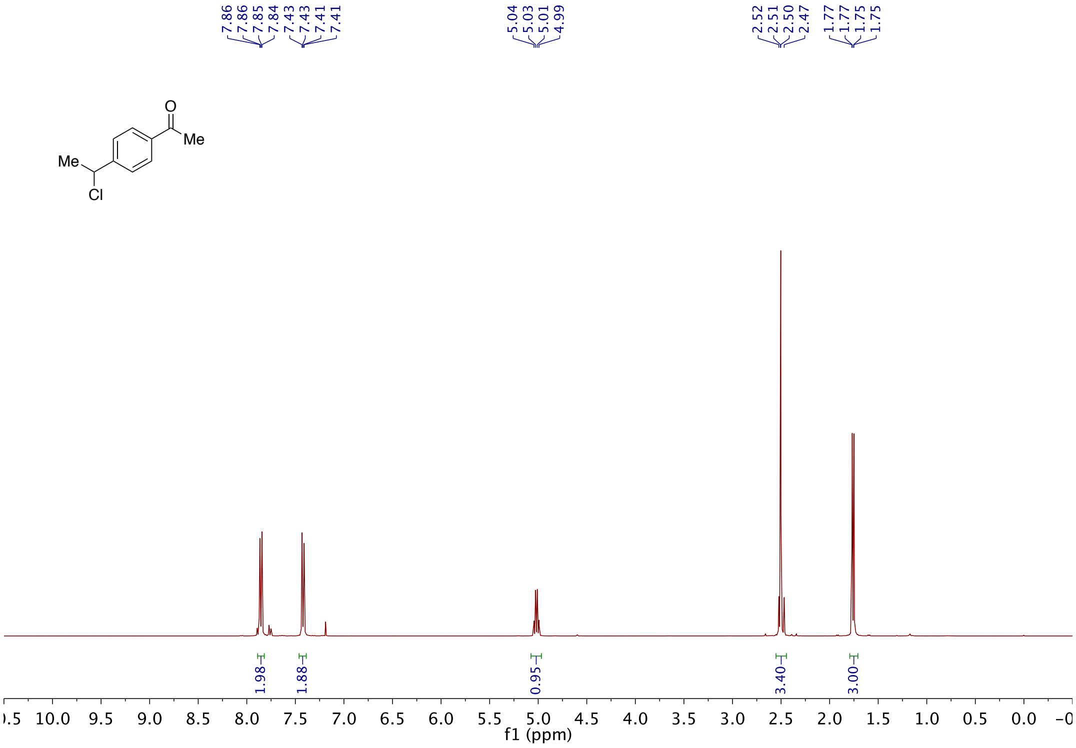

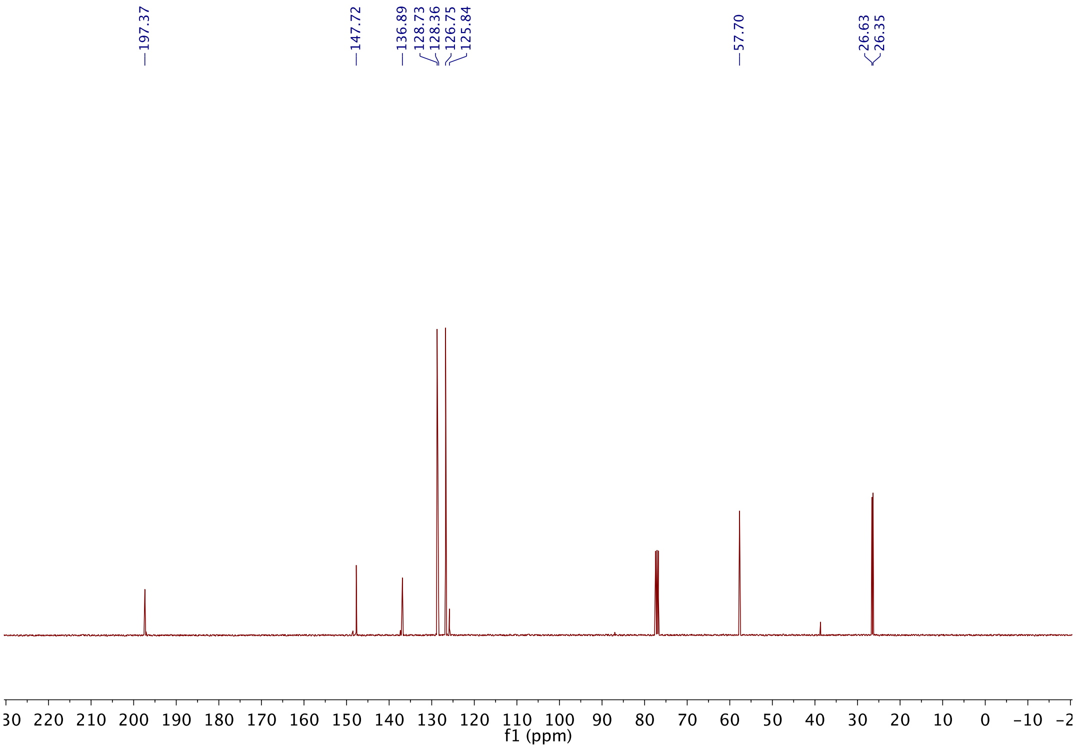

^{1}H and ^{13}C NMR spectra of 4-(1-chloroethyl)-*N*-methoxy-*N*-methylbenzamide (**6f**)

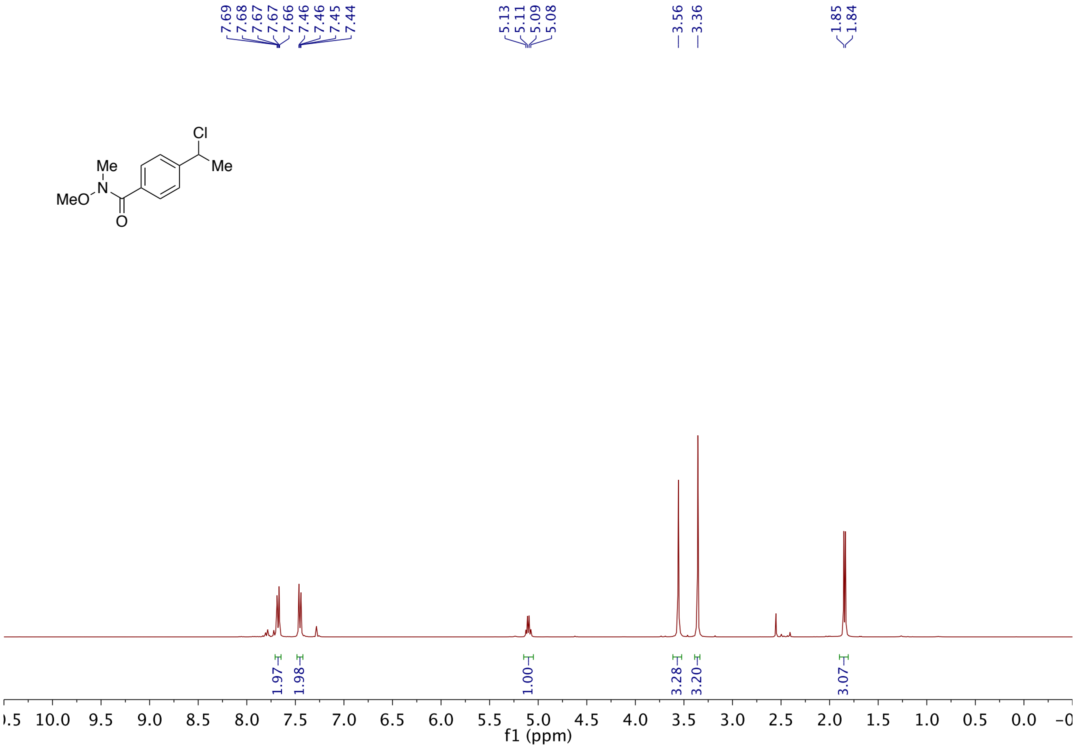

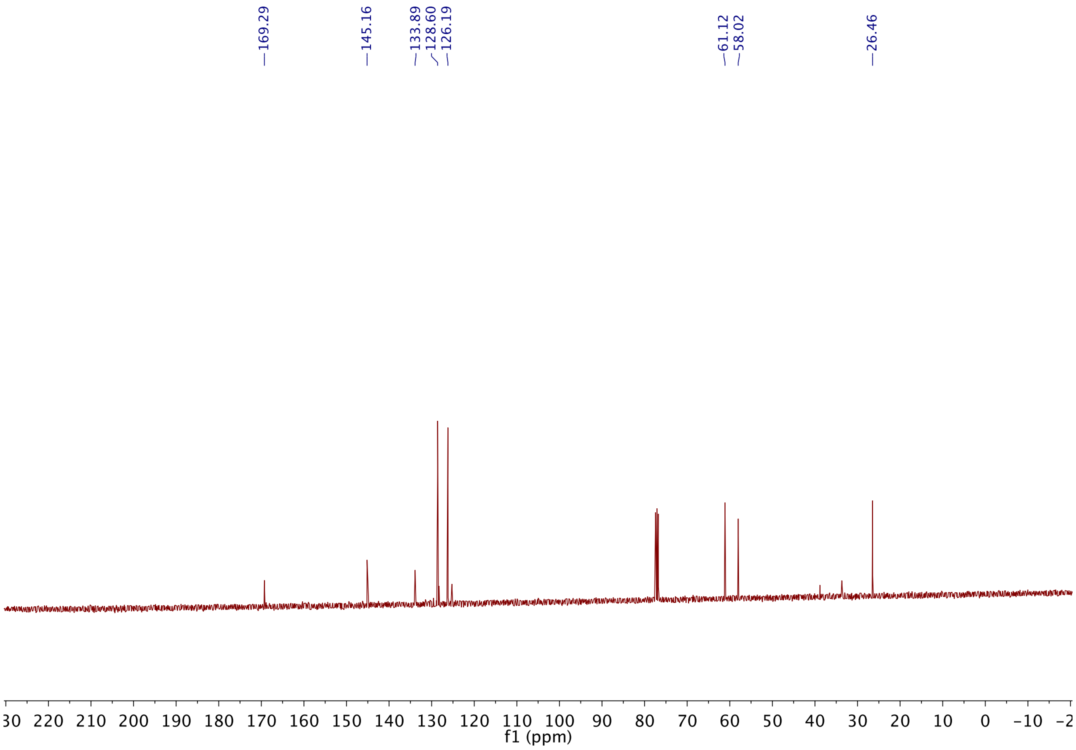

^{1}H and ^{13}C NMR spectra of 3-chloro-3-phenylpropyl acetate (**6g**)

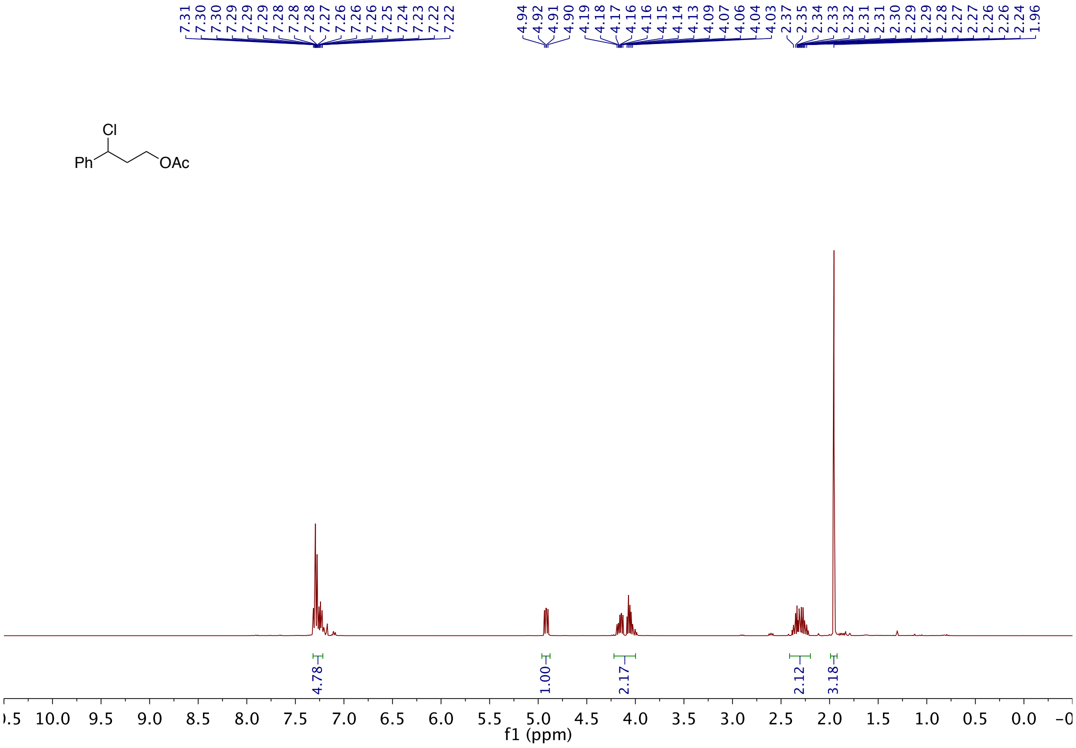

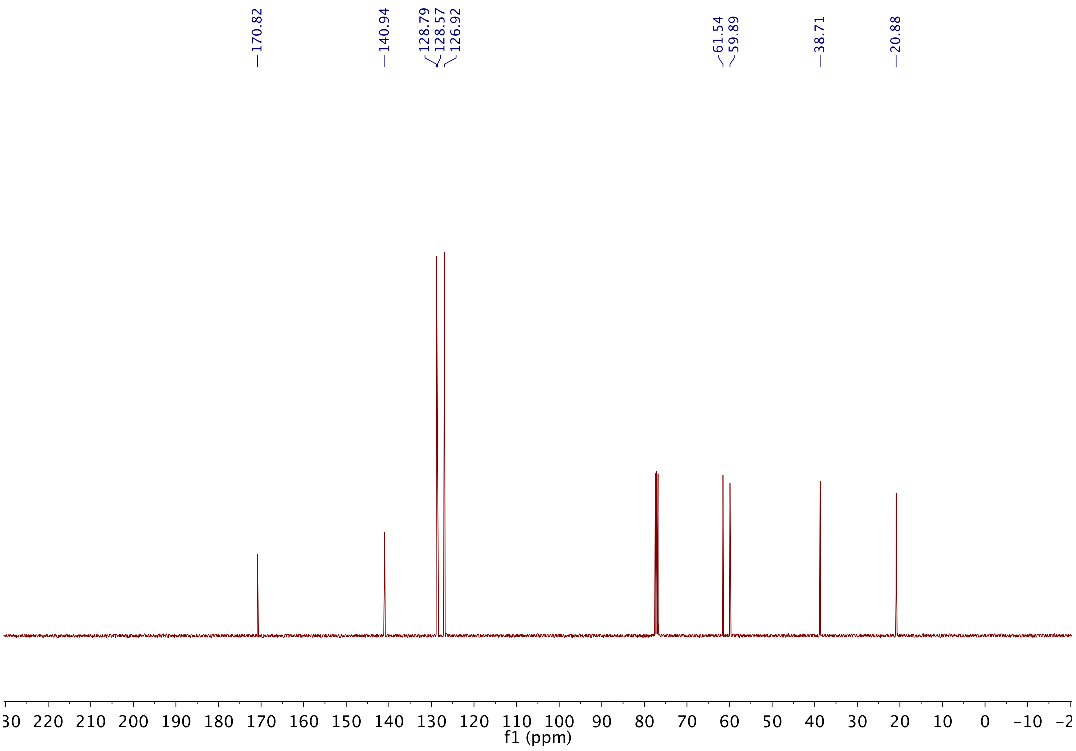

^{1}H and ^{13}C NMR spectra of 1-(4-(1-chloropentyl)phenyl)pentan-1-one (**6h**)

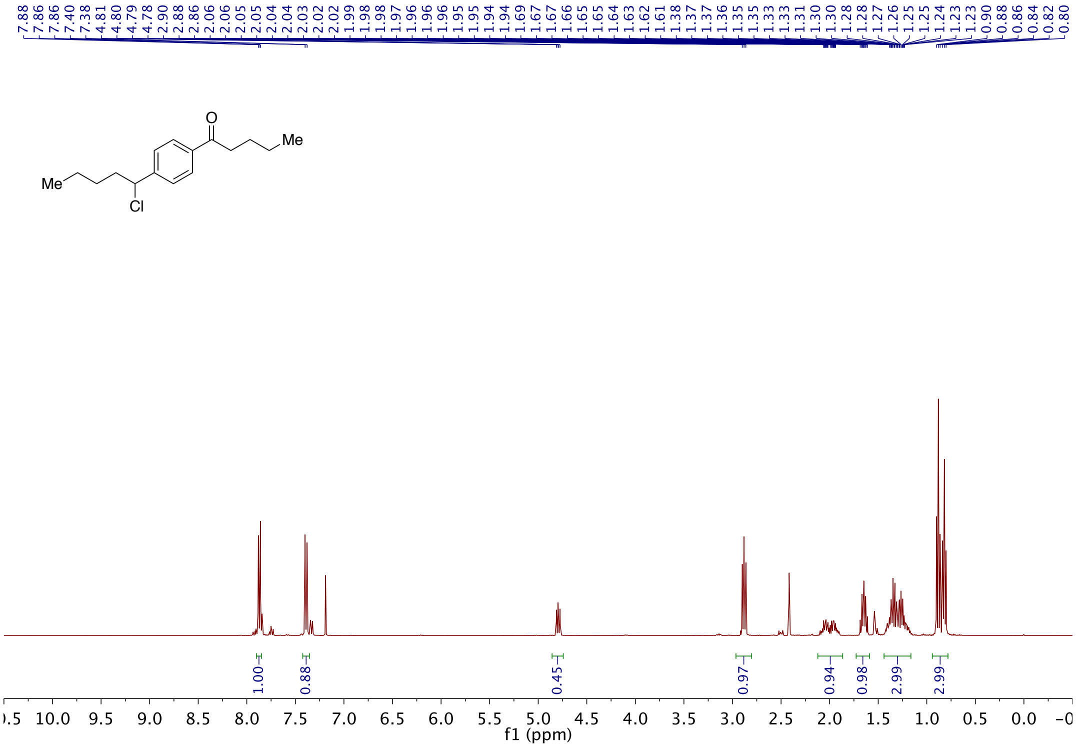

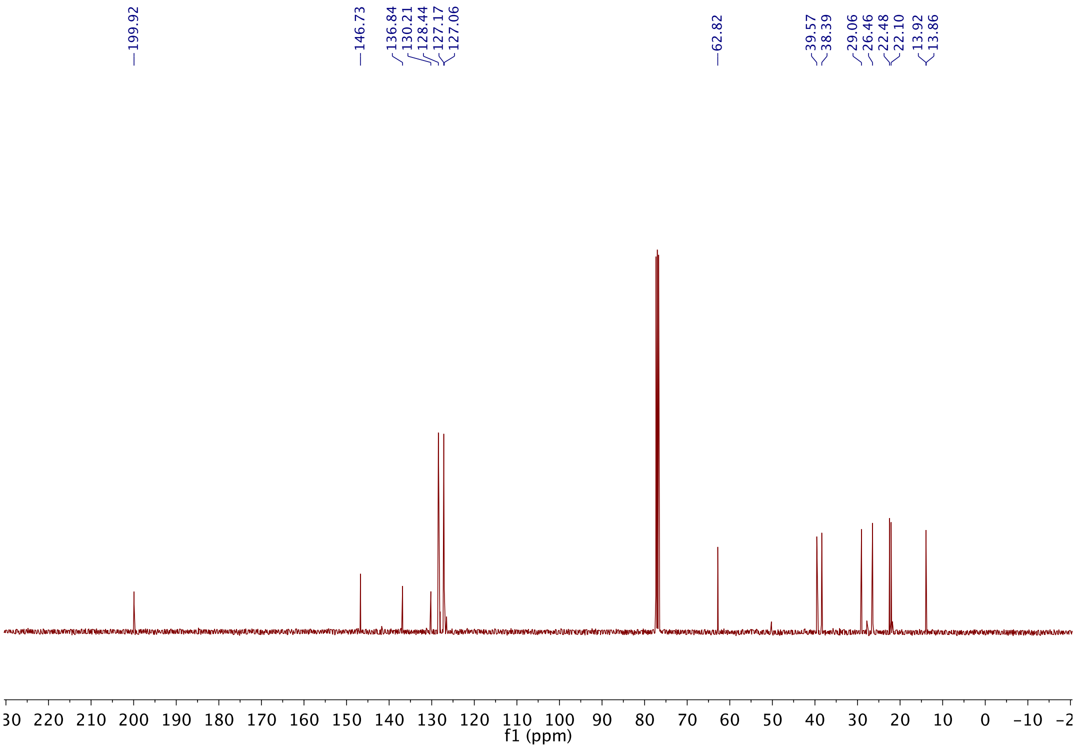

^{1}H and ^{13}C NMR spectra of methyl 4-(1-chloropentyl)benzoate (**6i**)

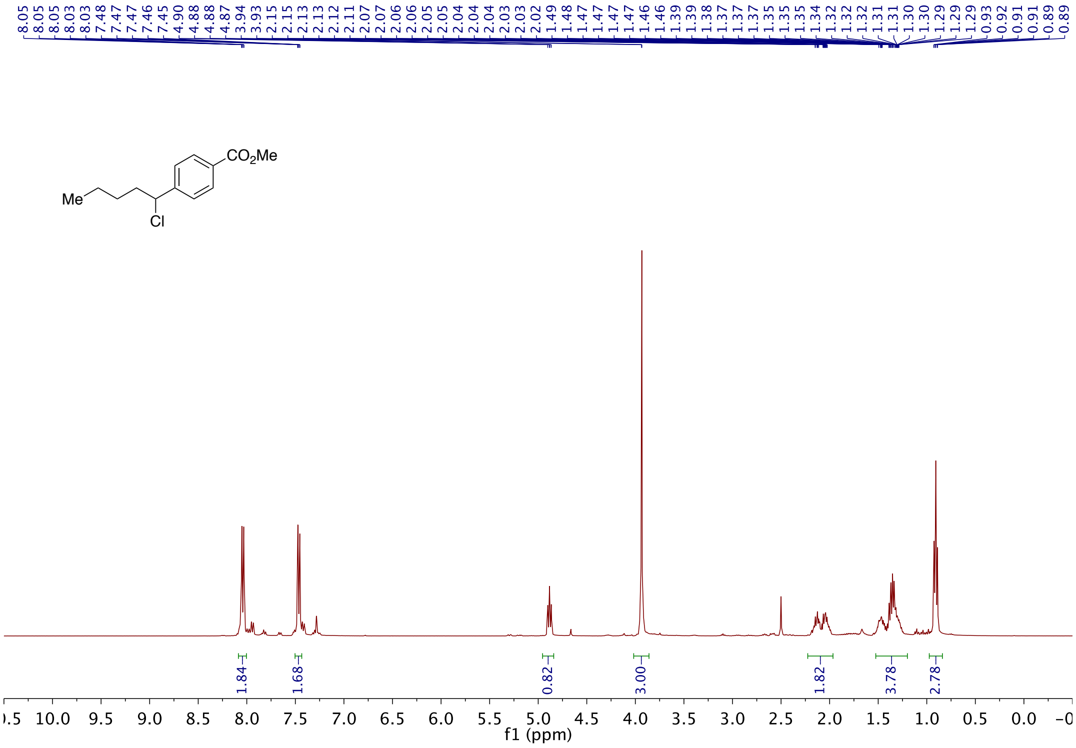

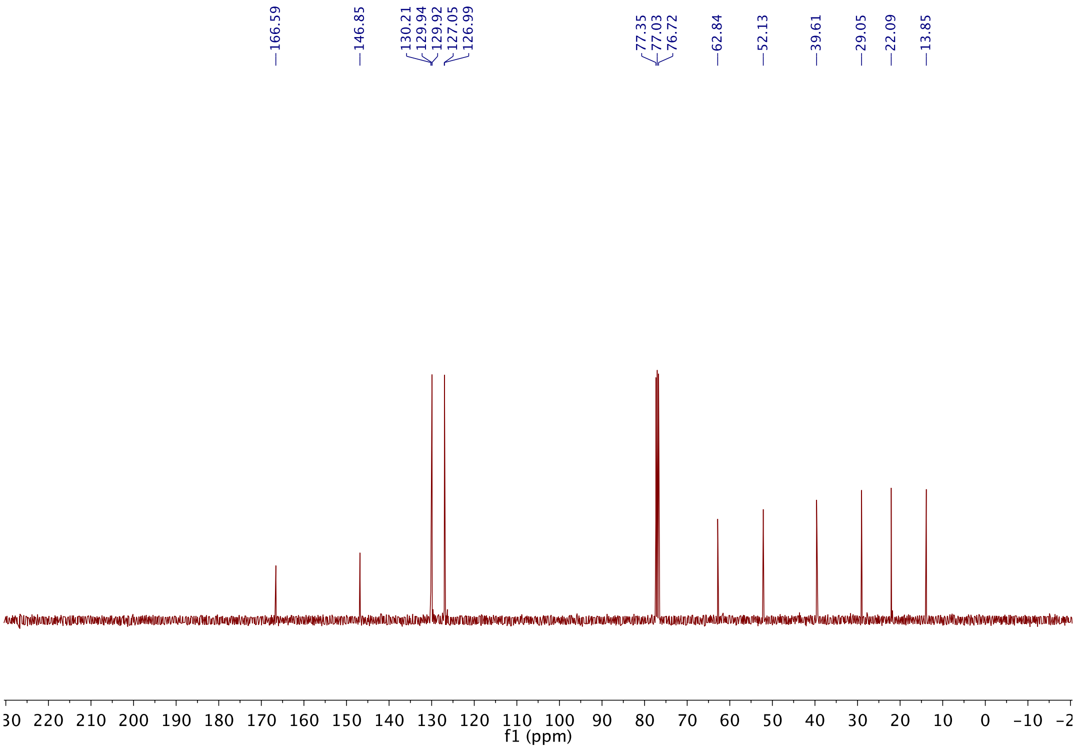

^{1}H and ^{13}C NMR spectra of 4-(1-chloropentyl)-*N*-methoxy-*N*-methylbenzamide (**6j**)

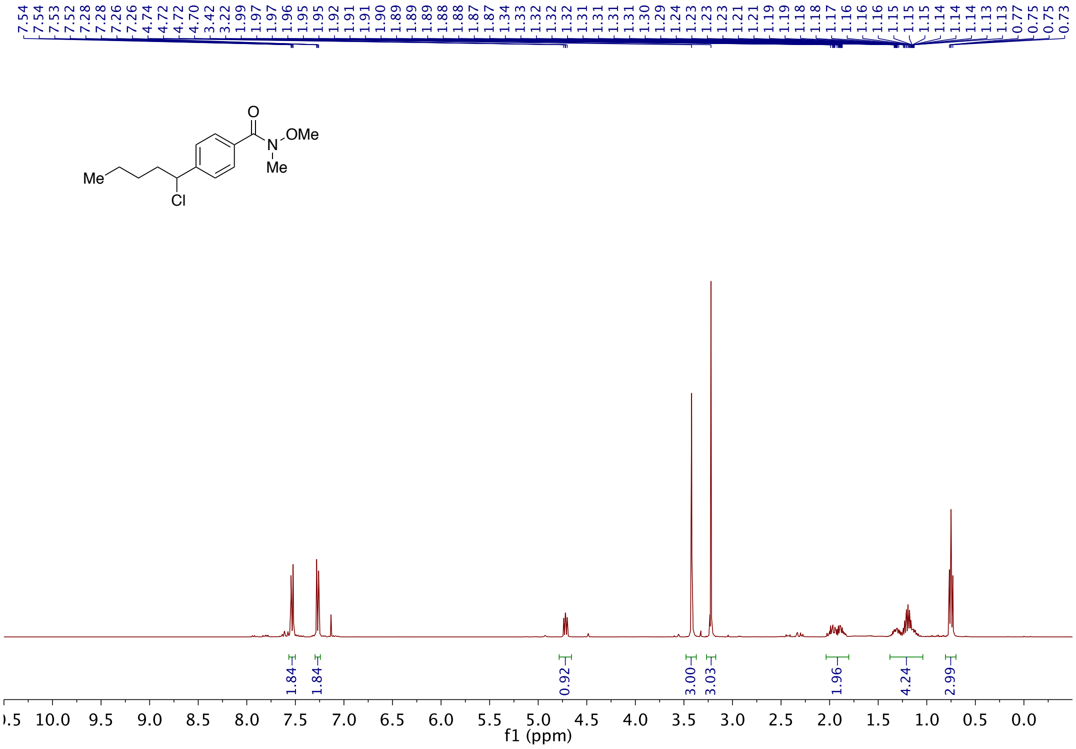

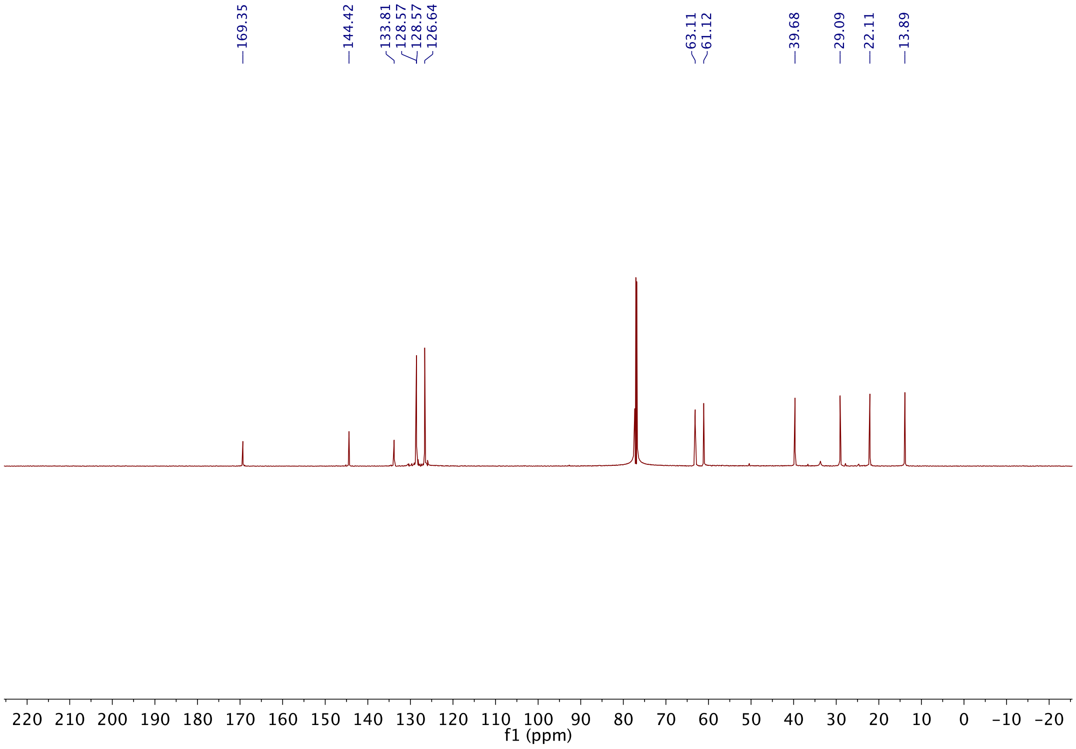

^{1}H and ^{13}C NMR spectra of 3-chloro-2,3-dihydro-1*H*-inden-1-one (**6k**)

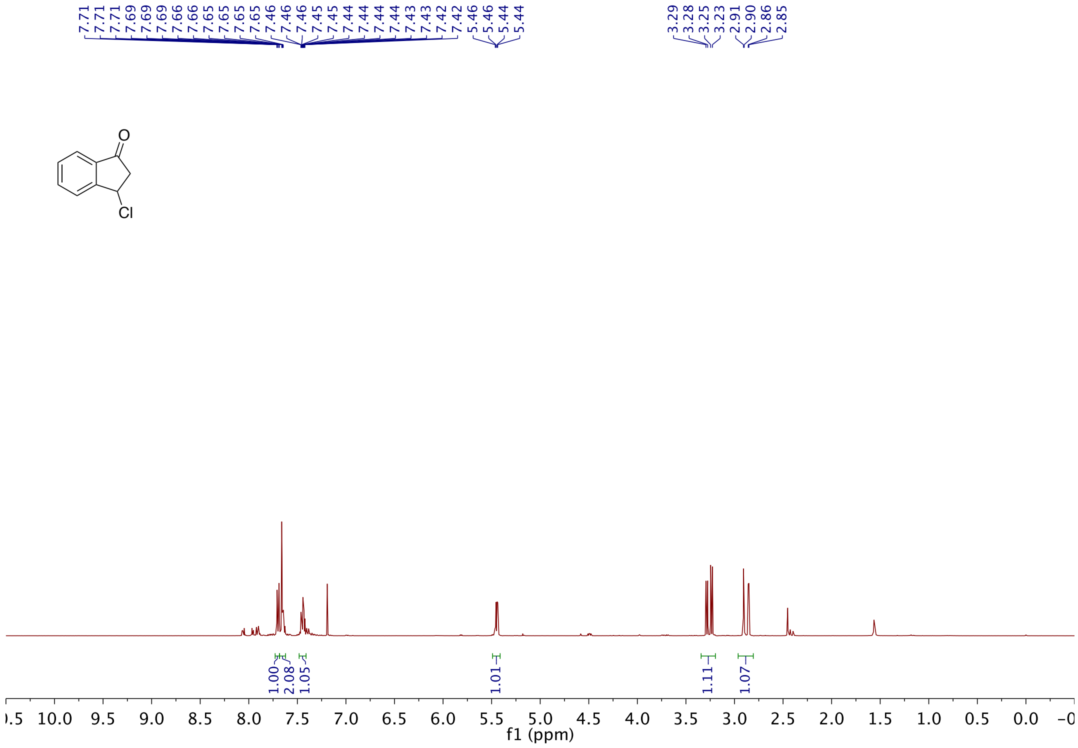

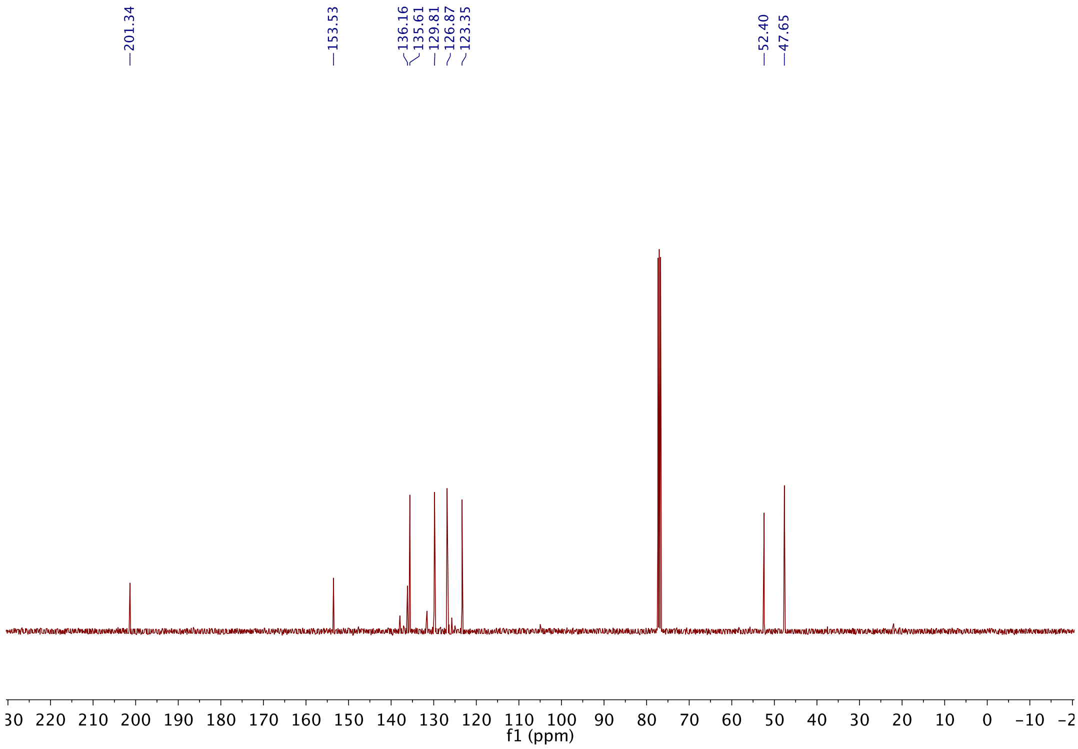

^{1}H and ^{13}C NMR spectra of 4-chloro-3,4-dihydronaphthalen-1(2*H*)-one (**6l**)

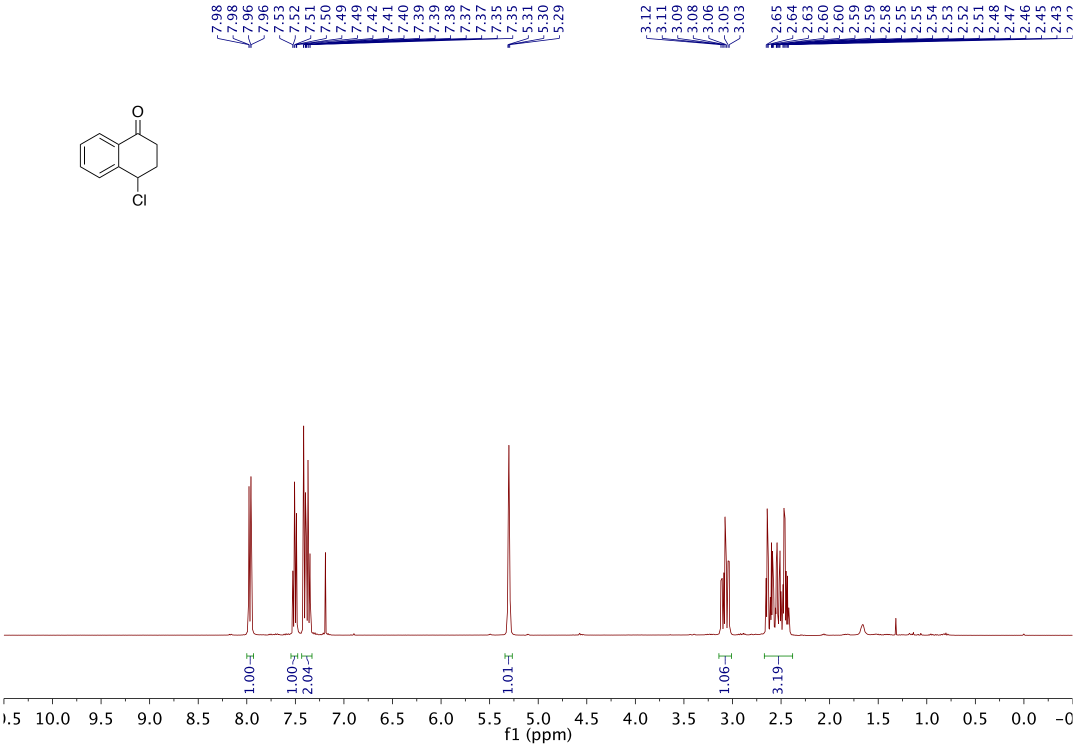

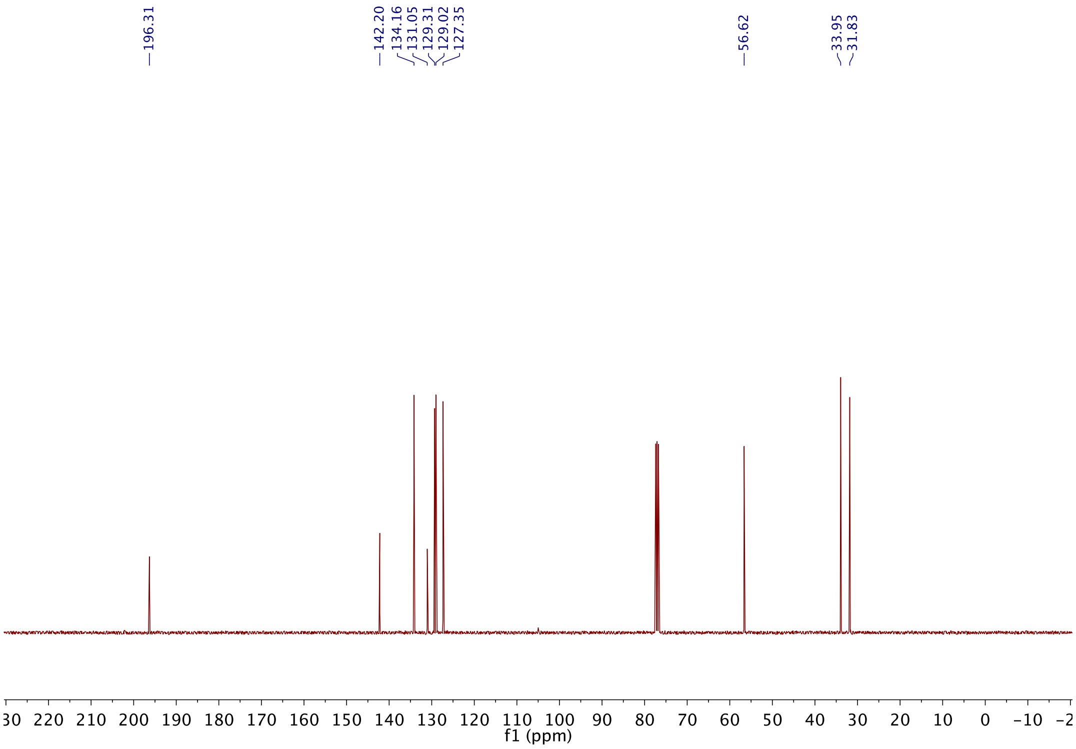

^{1}H and ^{13}C NMR spectra of (1-chloro-4-methylpentyl)benzene (**6m**)

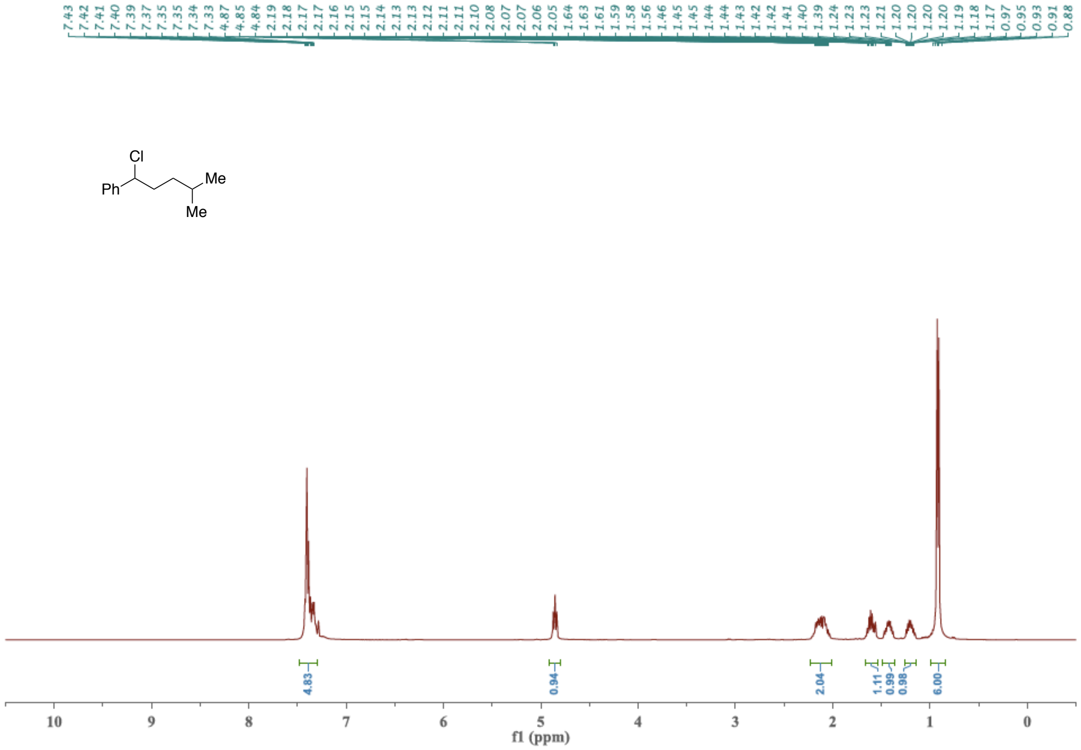

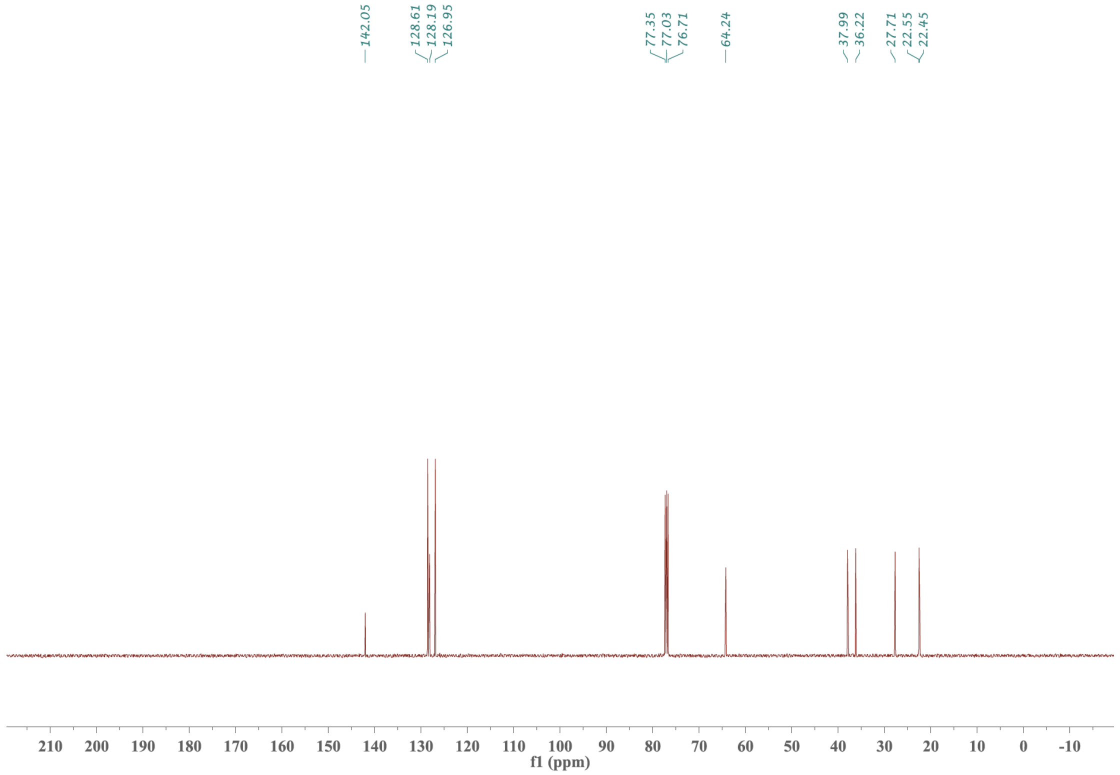

^{1}H and ^{13}C NMR spectra of *tert*-butyl 4-(1-(4-acetylphenyl)ethyl)piperazine-1-carboxylate (**7**)

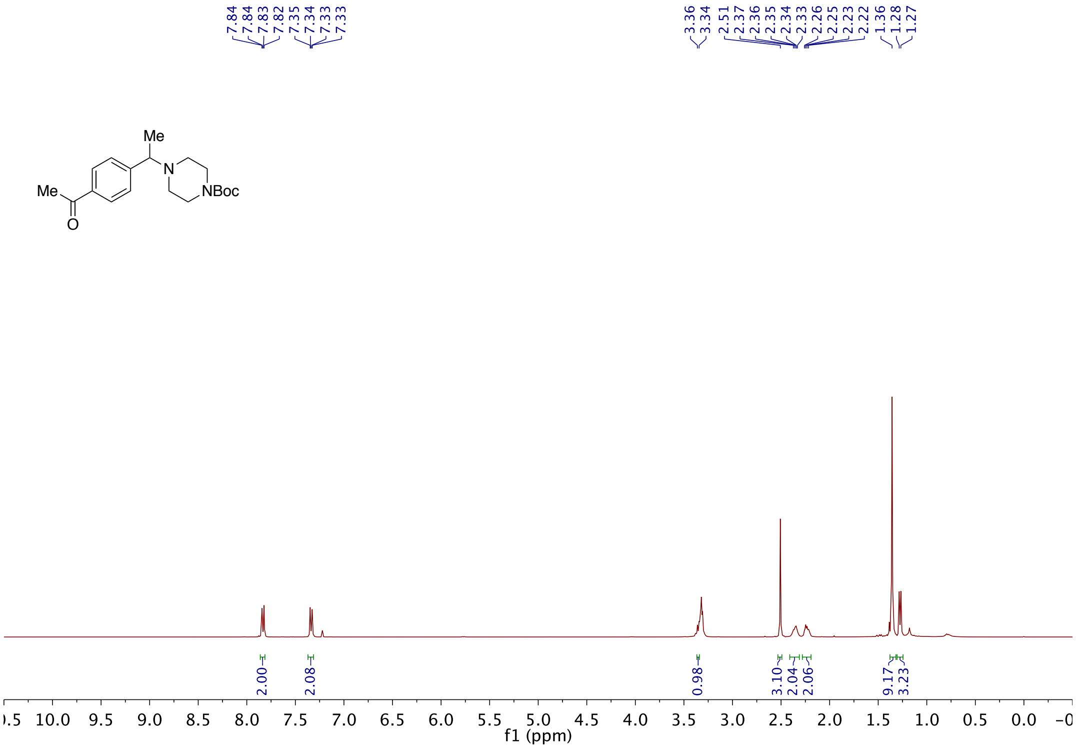

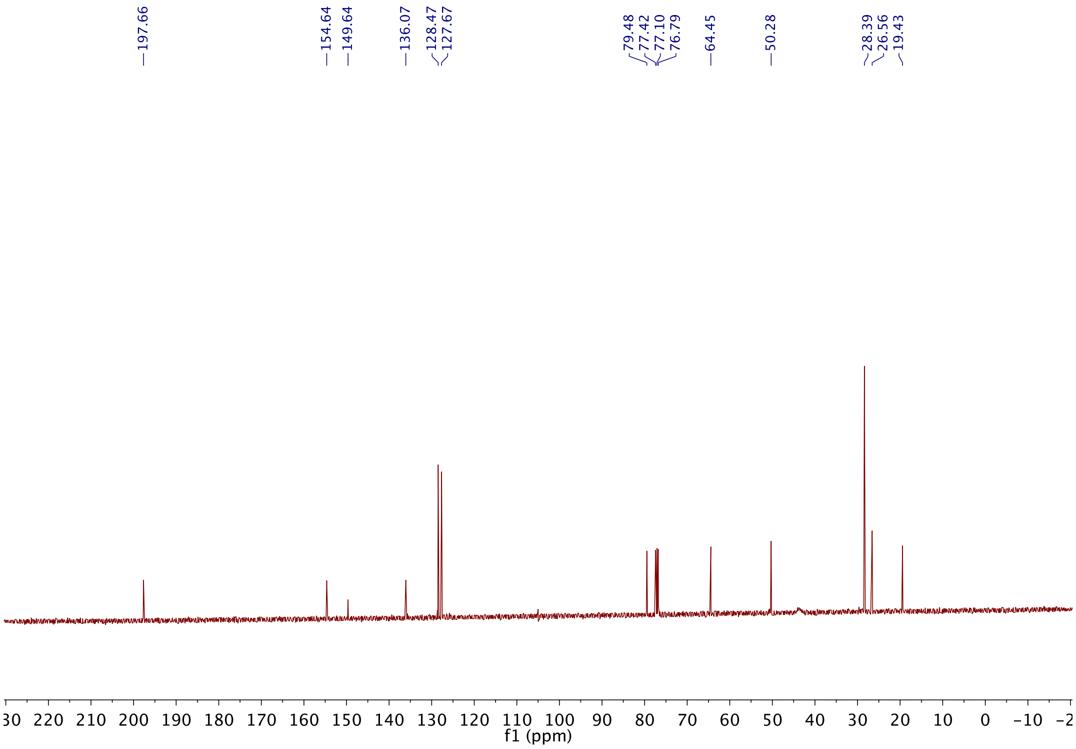

^{1}H and ^{13}C NMR spectra of 4-chloro-1-(2-chloropyridin-4-yl)-4-methylpentan-1-one (**8**)

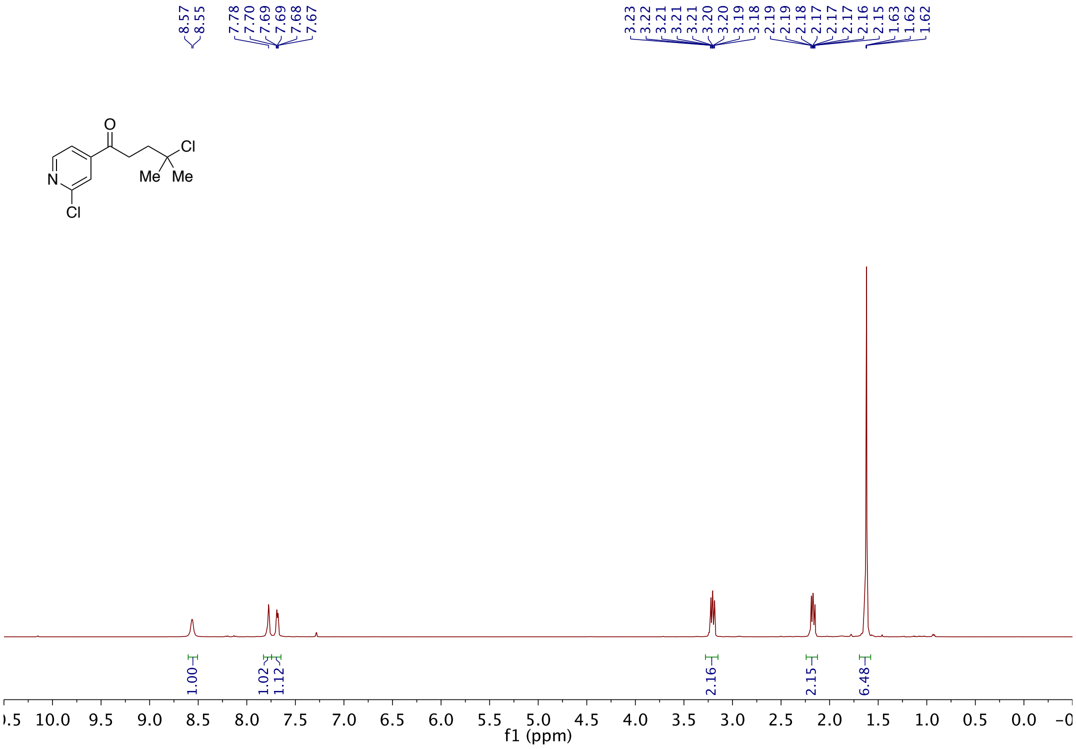

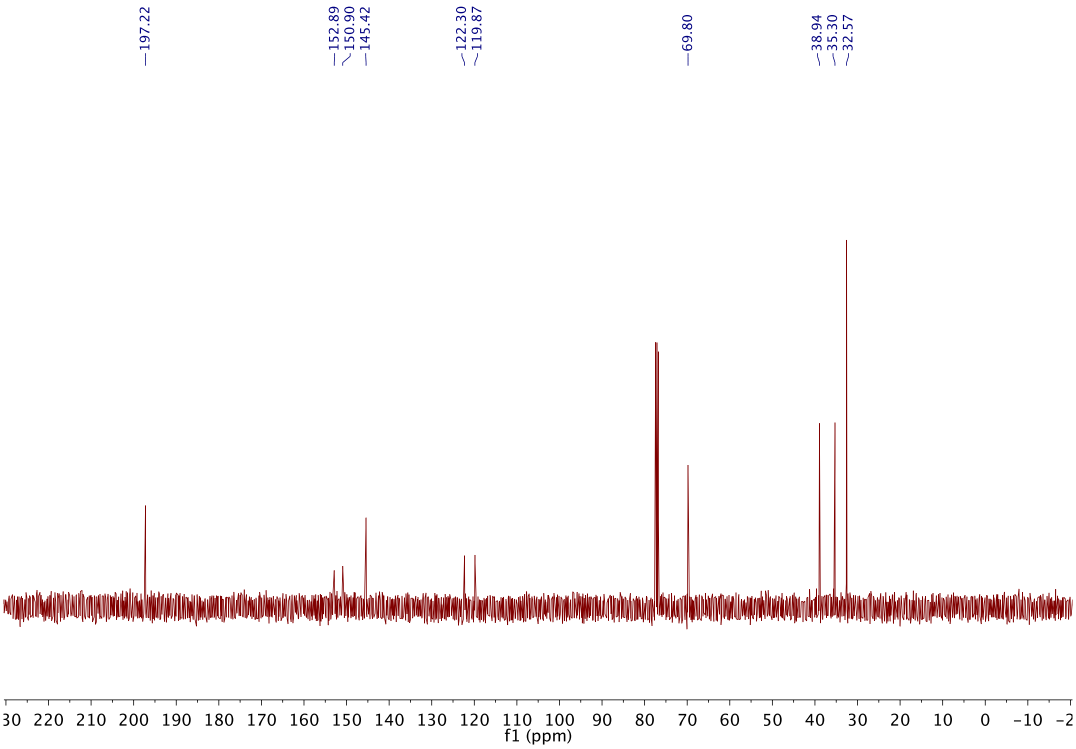

^{1}H and ^{13}C NMR spectra of methyl 4-chloro-2-(1,3-dioxoisoindolin-2-yl)-4-methylpentanoate (**9**)

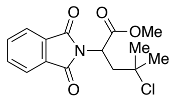

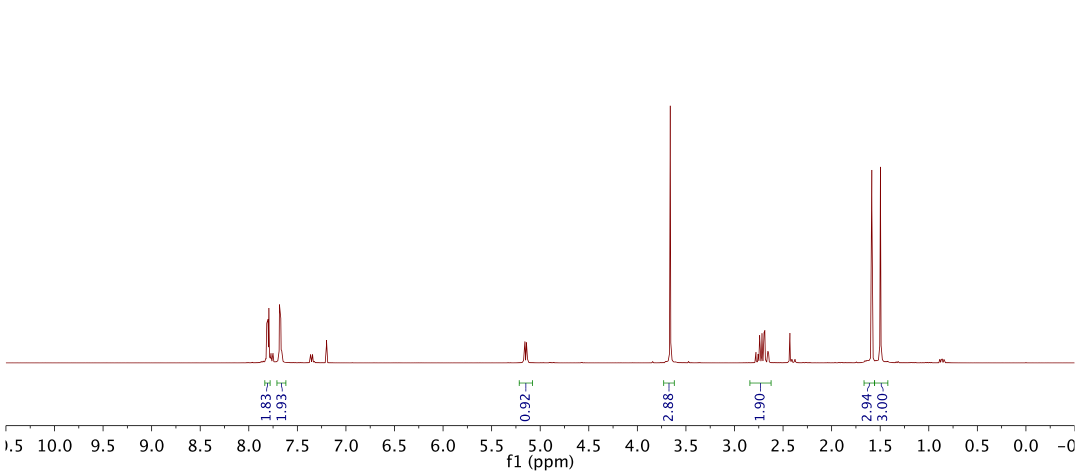

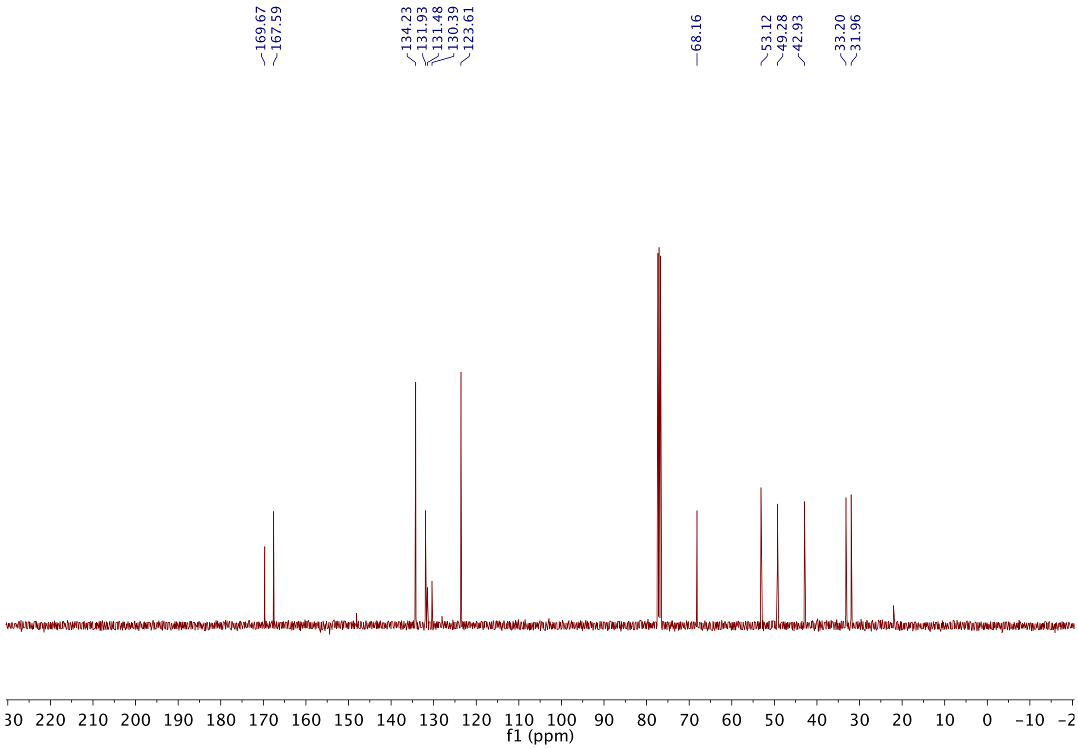

^{1}H and ^{13}C NMR spectra of methyl 2-(4-(1-chloro-2-methylpropyl)phenyl)propanoate (**10**)

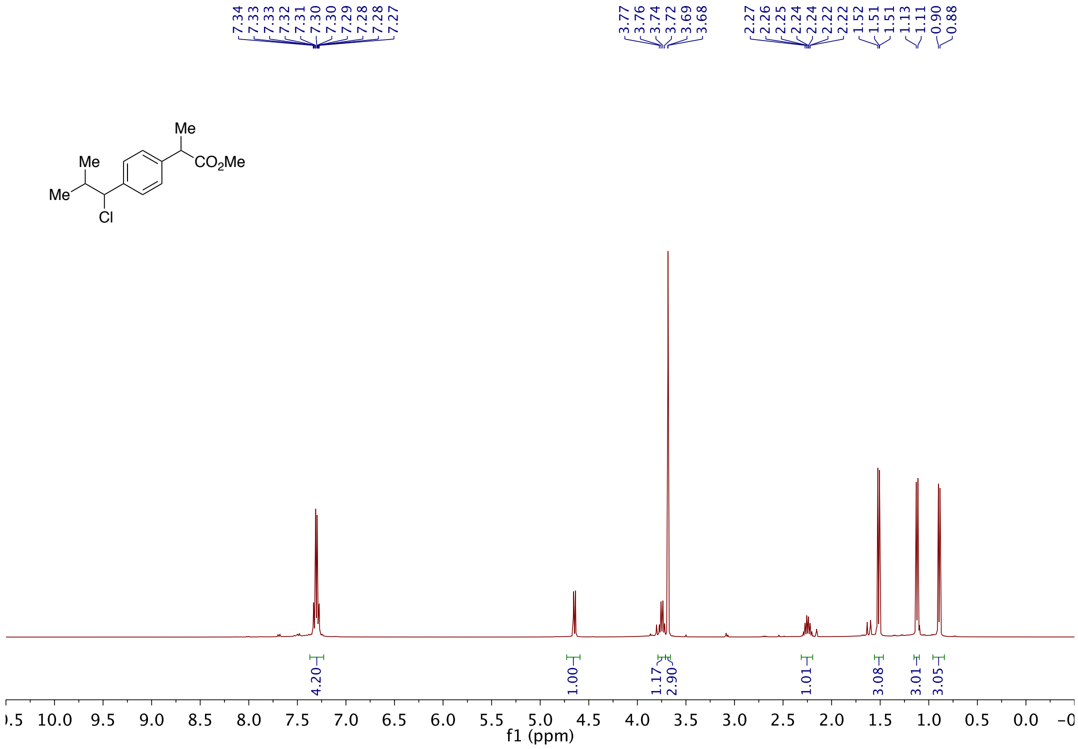

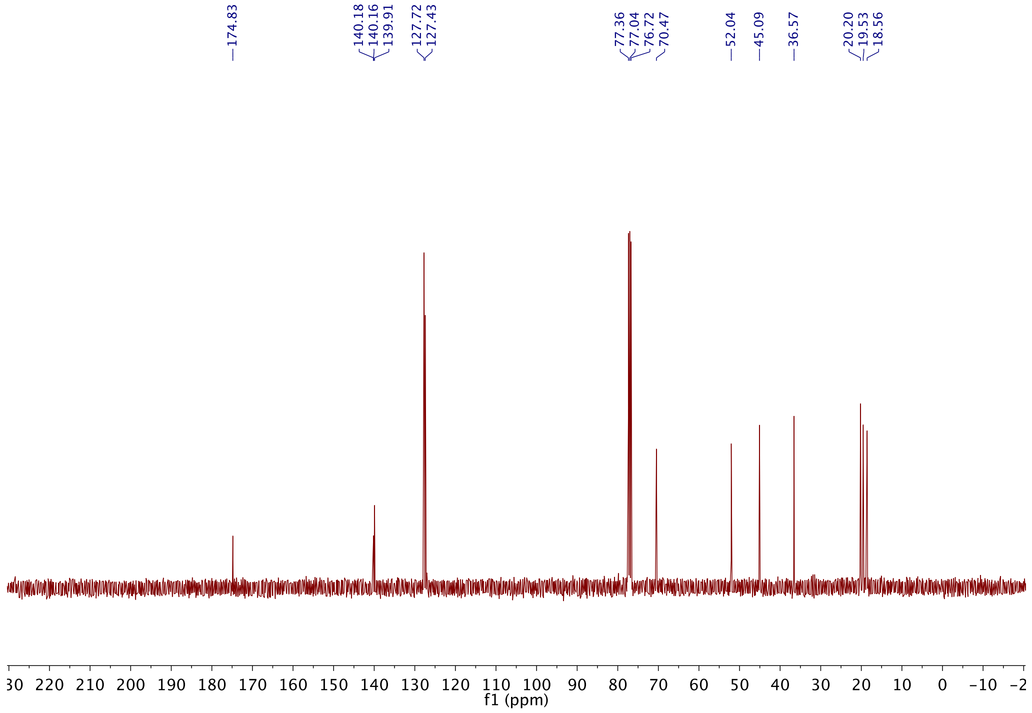

^{1}H and ^{13}C NMR spectra of methyl 4'-(1-chlorohexyl)-[1,1'-biphenyl]-4-carboxylate (**11**)

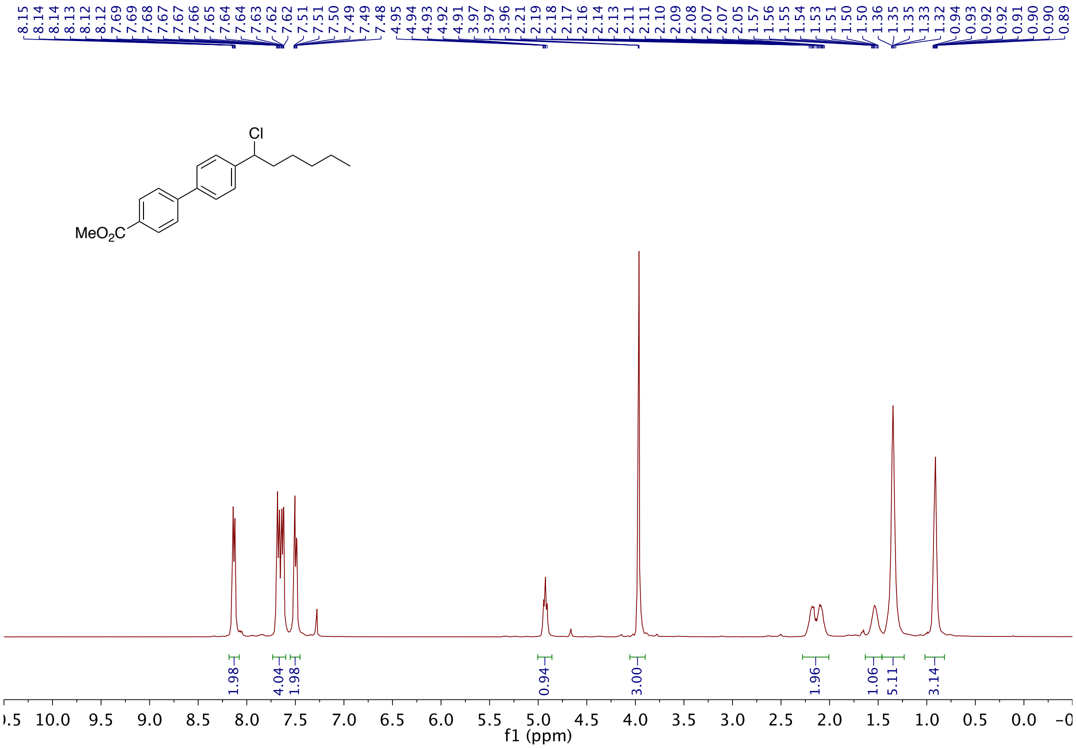

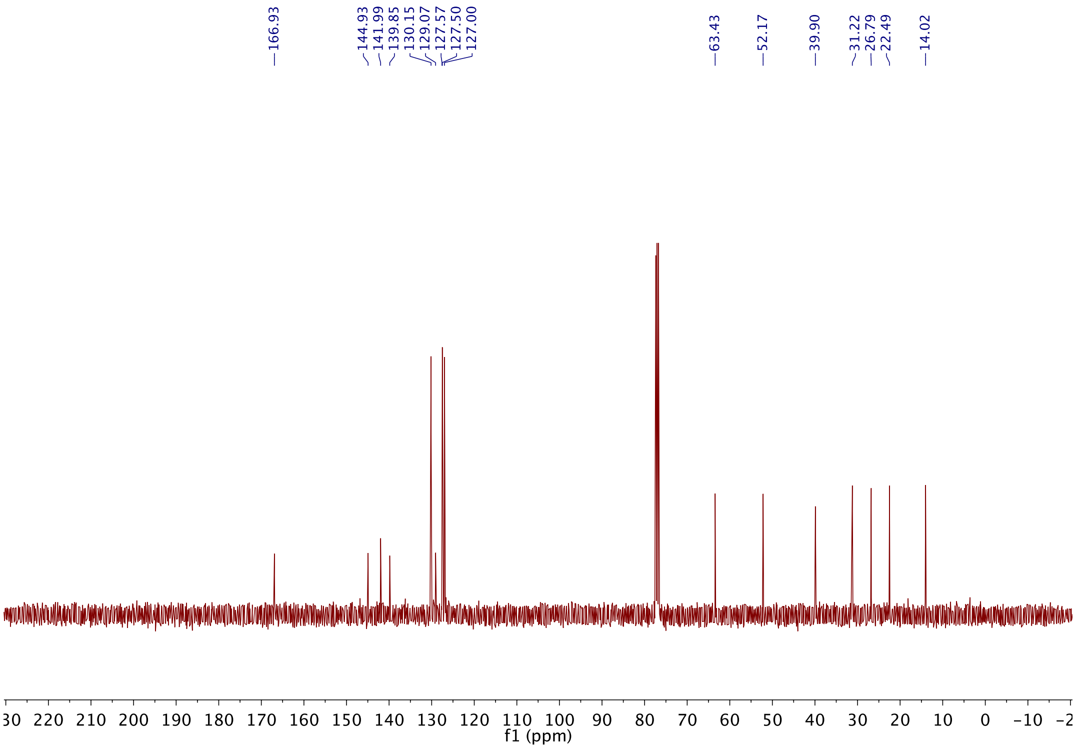

^{1}H and ^{13}C NMR spectra of 4-(1-chloroethyl)phenyl (4*R*)-4,7,7-trimethyl-3-oxo-2-oxabicyclo[2.2.1]heptane-1-carboxylate (**12**)

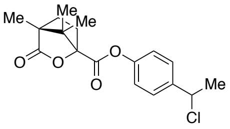

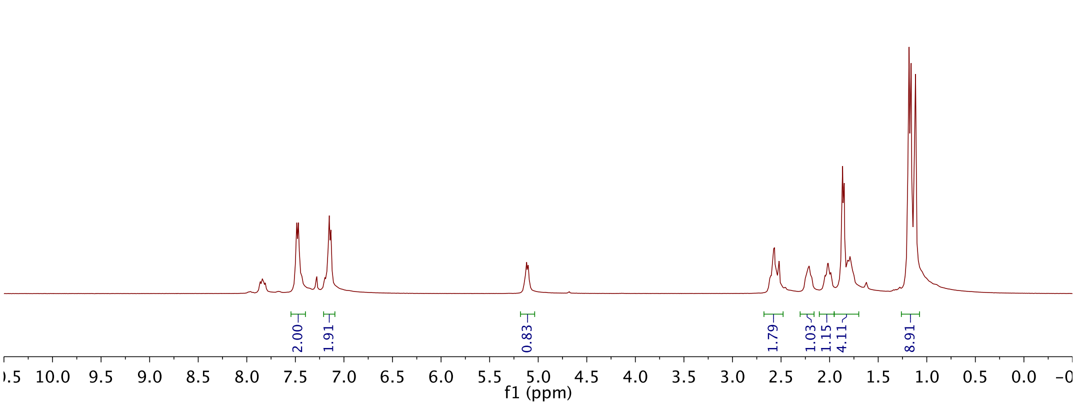

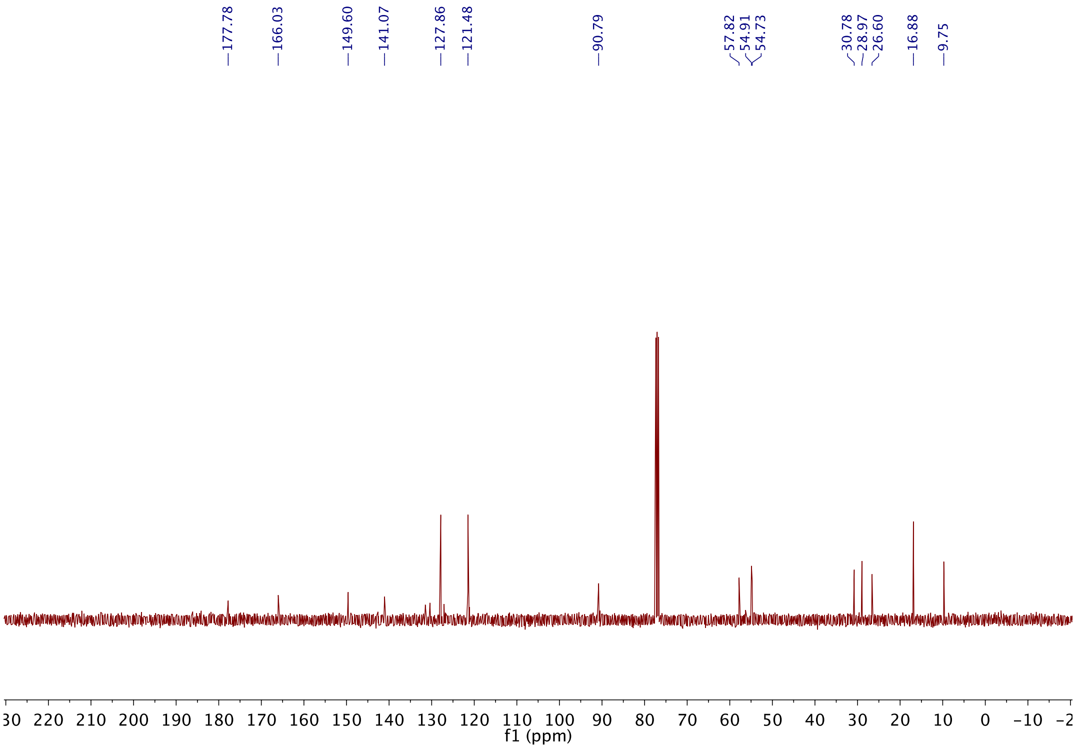

^{1}H and ^{13}C NMR spectra of 4-(1-chloroethyl)phenyl 2-(4-(4-chlorobenzoyl)phenoxy)-2-methylpropanoate (**13**)

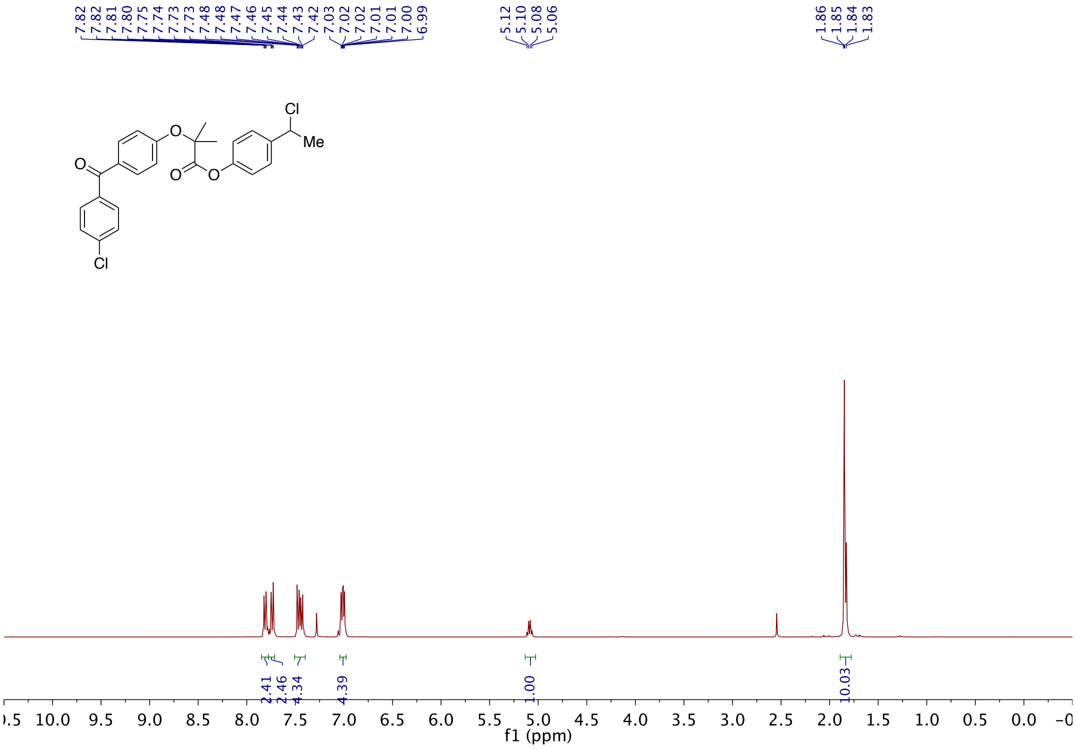

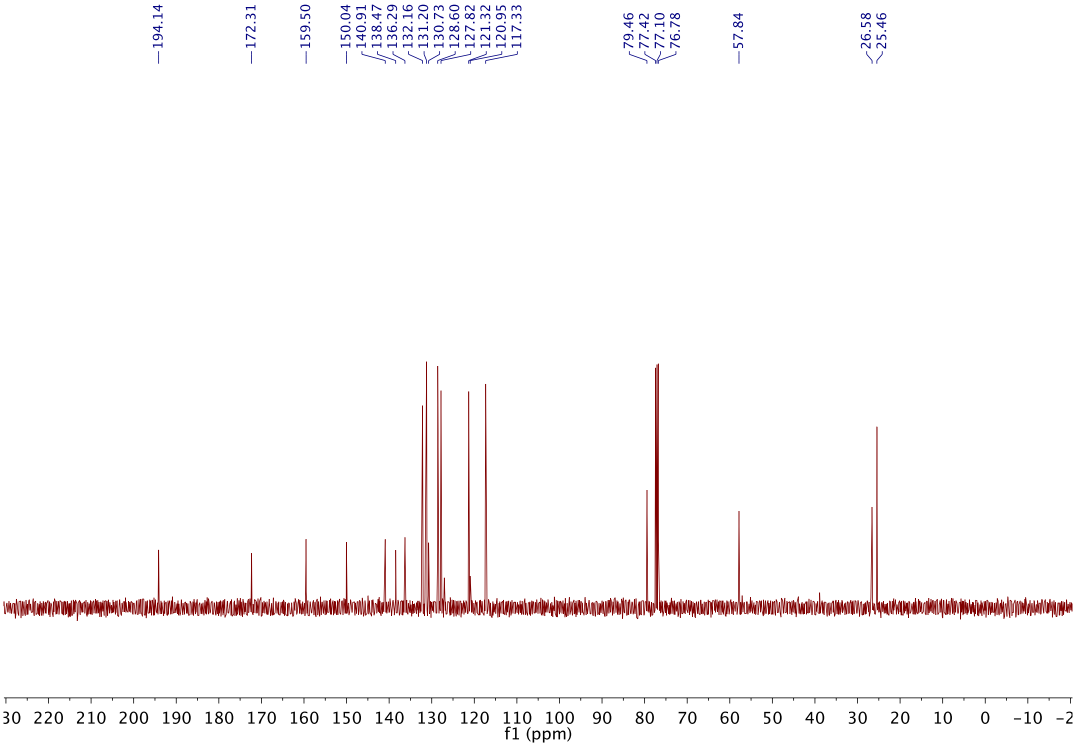

^{1}H and ^{13}C NMR spectra of 1-(6-(*tert*-butyl)-3-chloro-1,1-dimethyl-2,3-dihydro-1*H*-inden-4-yl)ethan-1-one (**14**)

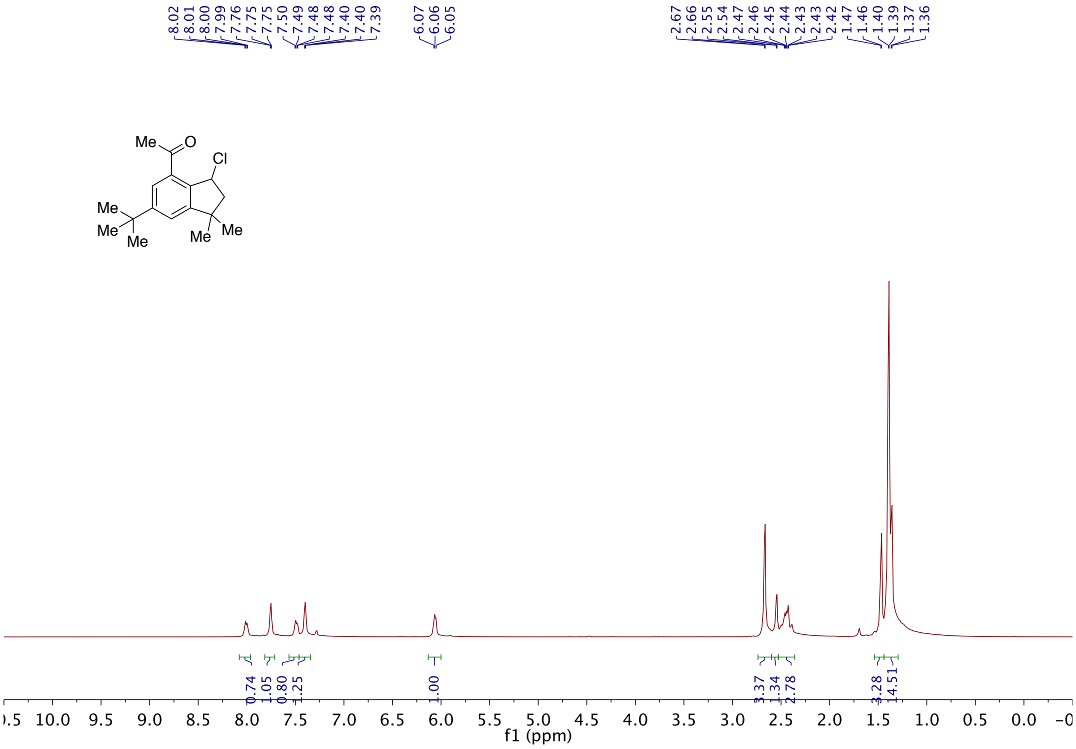

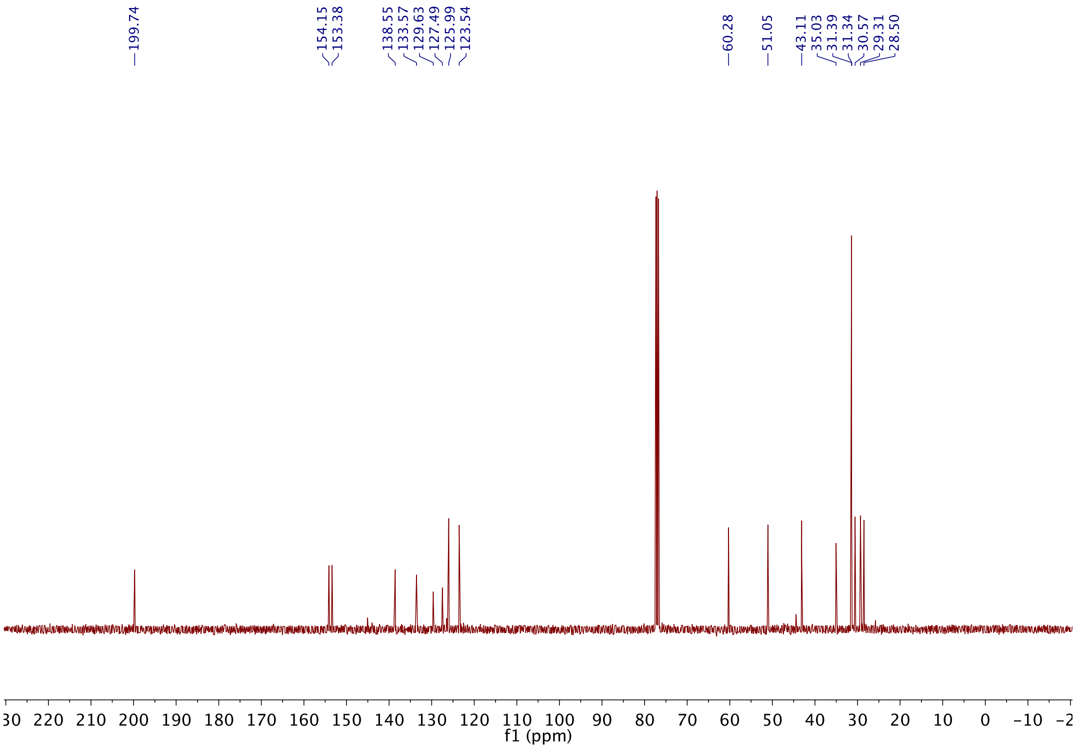

^{1}H and ^{13}C NMR spectra of 4-chloro-1-phenylpentan-1-one-2,2-d_2 (d_2-**2s**)

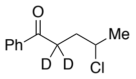

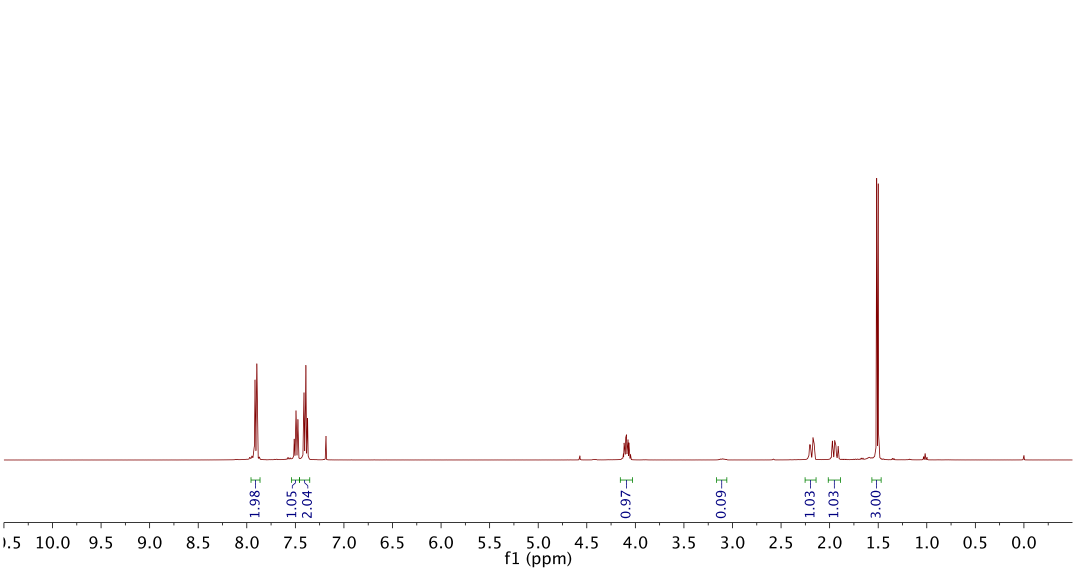

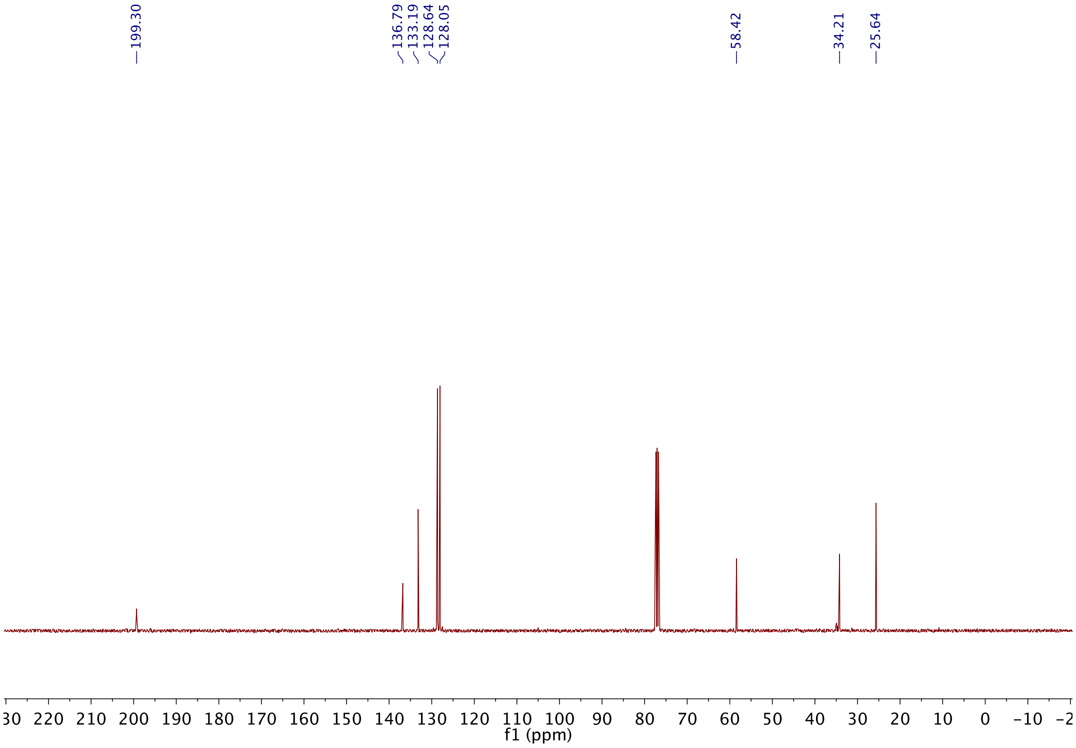

^{1}H and ^{13}C NMR spectra of 1-(4-(chloromethyl-d_2)phenyl)ethan-1-one (d_2-**6c**)

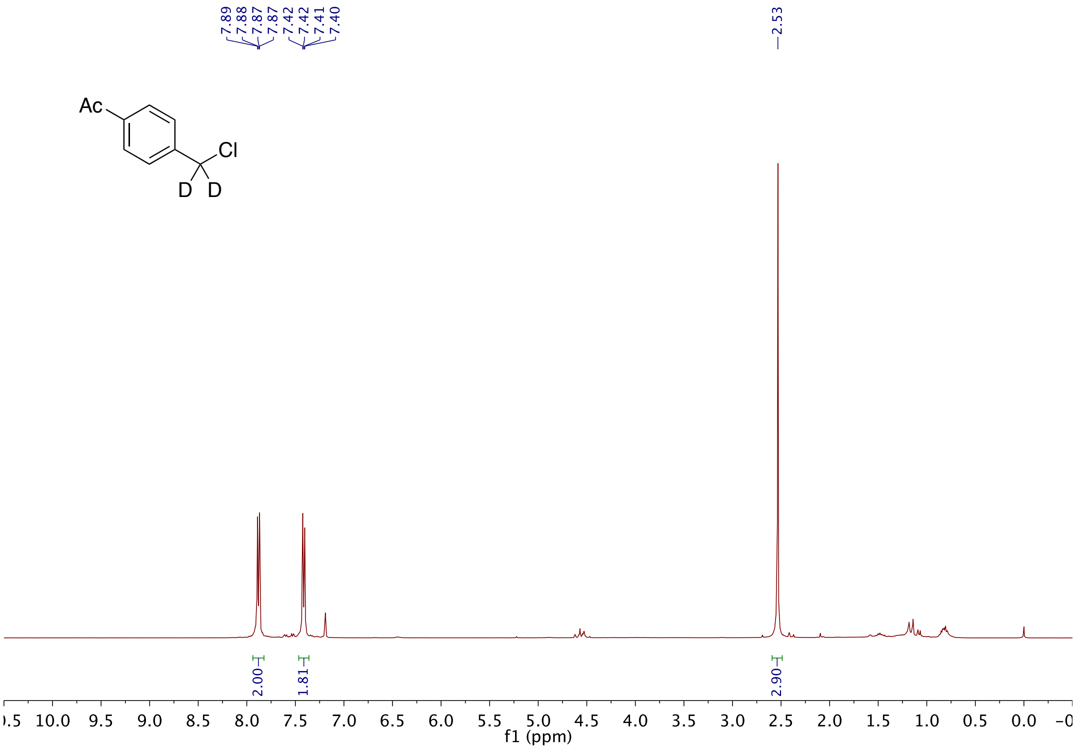

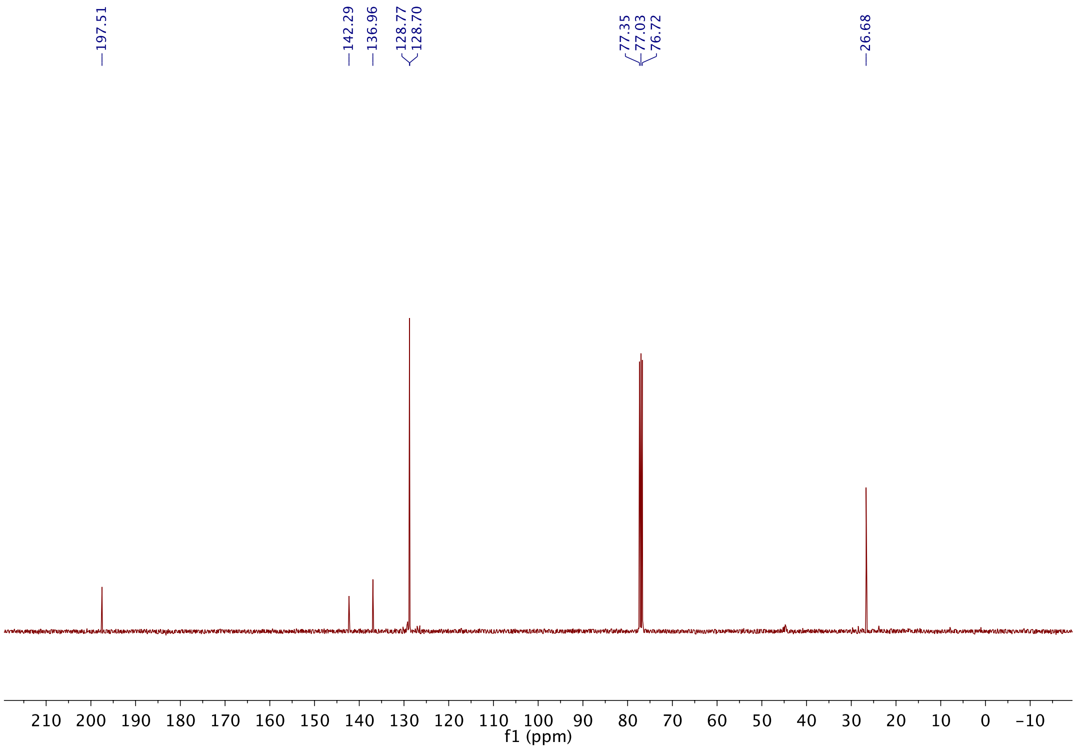

7. Supplementary References.

1. Zhan, G., He, Q., Yuan, X. & Chen, Y.-C. Asymmetric direct vinylogous Michael additions of allyl alkyl ketones to maleimides through dienamine catalysis. *Org. Lett.* **16**, 6000–6003 (2014).

2. Faulkner, A., Scott, J. S. & Bower, J. F. An umpolung approach to alkene carboamination: palladium catalyzed 1,2-amino-acylation, -carboxylation, -arylation, -vinylation, and -alkynylation. *J. Am. Chem. Soc.* **137**, 7224–7230 (2015).

3. Kurita, T. *et al.* Efficient and convenient heterogeneous palladium-catalyzed regioselective deuteration at the benzylic position. *Chem. Eur. J.* **14**, 664–673 (2008).

4. Roberts, J. T., Rittberg, B. R. & Kovacic, P. Chemistry of *N*-halo compounds. 32. Pyrolysis of *N*,*N*-dihalo derivatives of amides and sulfonamides. *J. Org. Chem.* **46**, 3988–3991 (1981).

5. Wu, X. *et al.* Tertiary-alcohol-directed functionalization of remote C(sp^3)–H Bonds by Sequential Hydrogen Atom and Heteroaryl Migrations. *Angew. Chem. Int. Ed.* **57**, 1640–1644 (2018).

6. Jiang, H. & Studer, A. α-Aminoxy-acid-auxiliary-enabled intermolecular radical γ-C(sp3)-H functionalization of ketones. *Angew. Chem. Int. Ed.* **57**, 1692–1696 (2018).

7. Zhang, X., Guo, S. & Tang, P. Transition-metal free oxidative aliphatic C–H fluorination. *Org. Chem. Front.* **2**, 806-810 (2015).

8. Ho, C. Y. *et. al.* Preparation of biphenyl derivatives as γ-secretase modulators. Worldwide Patent 2009052341 (2009).

9. Torres-Ochoa, R. O., Leclair, A., Wang, Q. & Zhu, J. Iron-catalysed remote C(sp(3))–H azidation of *O*-acyl oximes and *N*-acyloxy imidates enabled by 1,5-hydrogen atom transfer of iminyl and imidate radicals: Synthesis of gamma-azido ketones and beta-azido alcohols. *Chem. Eur. J.* **25**, 9477–9484 (2019).

10. Liu, P., Liang, R., Lu, L., Yu, Z. & Li, F. Use of a cyclometalated iridium(III) complex containing a N-C-N-coordinating terdentate ligand as a catalyst for the alpha-alkylation of ketones and *N*-alkylation of amines with alcohols. *J. Org. Chem.* **82**, 1943–1950 (2017).

11. Chan, L. K., Poole, D. L., Shen, D., Healy, M. P. & Donohoe, T. J. Rhodium-catalyzed ketone methylation using methanol under mild conditions: formation of alpha-branched products. *Angew. Chem. Int. Ed.* **53**, 761–765 (2014).

12. Xia, Y., Wang, J. & Dong, G. Suzuki-Miyaura coupling of simple ketones via activation of unstrained carbon-carbon bonds. *J Am. Chem. So.c* **140**, 5347–5351 (2018).

13. Ackerman, L. K. G., Martinez Alvarado, J. I. & Doyle, A. G. Direct C–C bond formation from alkanes using Ni-photoredox catalysis. *J. Am. Chem. Soc.* **140**, 14059–14063 (2018).

14. Ikeno, T., Kimura, T., Ohtsuka, Y. & Yamada. T. Selective 1,4-reduction of *α,β*-unsaturated carbonyl compounds by combined use of bis(1,3-diketonato)cobalt(II) complex and diisobutylaluminum hydride. *Synlett* 96–98 (1999).

15. Kan, J. *et al.* Oxidation of enones for regioselective [3+2] cycloaddition through gamma-enone radical intermediates. *Chem. Eur. J.* **25**, 15233–15238 (2019).

16. Rhee. I., Ryu, I., Omura. H., Murai. S. & Sonoda. N. The reaction of *α*-iodomercuric ketones with nickel carbonyl in the presence of carbonyl compounds. *Chem. Lett.* 1435–1436 (1979).

17. Concellón, J., Rodríguez-Solla, H., Concellón, C. & Díaz, P. Synthesis of *E-α,β*-unsaturated ketones with complete stereoselectivity *via* sequential aldol-type/elimination reactions promoted by samarium diiodide or chromium dichloride. *Synlett* 837–840 (2006).

18. Yang, F. & Ackermann, L. Ruthenium-catalyzed C–H oxygenation on aryl Weinreb amides. *Org. Lett.* **15**, 718–720 (2013).

19. Jiang, Q. *et al.* PIFA-mediated esterification reaction of alkynes with alcohols via oxidative cleavage of carbon triple bonds. *J. Org. Chem.* **79**, 2709–2715 (2014).

20. Martin. B. R. *et al.* Pyrazole cannabinoid agonist and antagonists. Patent No.: US 6,509,367 BI (2003).

21. Xiao, H. *et al.* Copper-catalyzed late-stage benzylic C(sp^3)–H trifluoromethylation. *Chem* **5**, 940–949 (2019).

22. Clark, J. R., Feng, K., Sookezian, A. & White, M. C. Manganese-catalysed benzylic C(sp^3)–H amination for late-stage functionalization. *Nat. Chem.* **10**, 583–591 (2018).

23. Thomson, C. J., Barber, D. M. & Dixon, D. J. One-pot catalytic enantioselective synthesis of 2-pyrazolines. *Angew. Chem. Int. Ed.* **58**, 2469–2473 (2019).

24. Workentin, M. S., Leigh, William J. & Jeffrey, K. R. Organic reactions in liquid crystalline solvents. 10. Studies of the ordering and mobilities of simple alkanophenones in CCH-n liquid crystals by deuterium NMR spectroscopy and Norrish II photoreactivity. *J. Am. Chem. Soc.* **112**, 20, 7329–7336 (1990).

25. Pinna, G. *et al.* An umpolung sulfoxide reagent for use as a functionalized benzyl carbanion equivalent. *Tetrahedron* **67**, 5268–5281 (2011).

26. CCDC 1562076 (**2d**) contains the supplementary crystallographic data for this paper. These data can be obtained free of charge from the Cambridge Crystallographic Data Centre via www.ccdc.cam.ac.uk/data_request/cif.

27. CCDC 1562075 (**4k**) contains the supplementary crystallographic data for this paper. These data can be obtained free of charge from the Cambridge Crystallographic Data Centre via www.ccdc.cam.ac.uk/data_request/cif.

28. Frisch, M. J. *et al.* Gaussian 16, Revision C.01, Gaussian Inc.: Wallingford, CT (2016).

29. Lee, C., Yang, W. & Parr, R. G. Development of the Colle-Salvetti correlation-energy formula into a functional of the electron density. *Phys. Rev. B* **37**, 785–789 (1988).

30. Basumatary, B. *et al.* Copper 1,19-diaza-21,24-dicarbacorrole: A corrole analogue with an n–n linkage stabilizes a ground-state singlet organocopper species. *Angew. Chem. Int. Ed.* **132**, 16031–16035 (2020).

31. Hu, H. *et al.* Copper-catalysed benzylic C–H coupling with alcohols *via* radical relay enabled by redox buffering. *Nat. Catal.* **3**, 358–367 (2020).

32. Paradisi, A. *et al.* Formation of a copper(II)–tyrosyl complex at the active site of lytic polysaccharide monooxygenases following oxidation by H_2O_2. *J. Am. Chem. Soc.* **141**, 18585–18599 (2019).

33. Mandal, M. *et al.* Mechanisms for hydrogen-atom abstraction by mononuclear copper(III) cores: hydrogen-atom transfer or concerted proton-coupled electron transfer? *J. Am. Chem. Soc.* **141**, 17236–17244 (2019).

34. Jiao, Y., Chiou, M. F., Li, Y. & Bao, H. Copper-catalyzed radical acyl-cyanation of alkenes with mechanistic studies on the tert-butoxy radical. *ACS Catal.* **9**, 5191–5197 (2019).

35. Isegawa, M. *et al.* Copper-catalyzed enantioselective boron conjugate addition: DFT and AFIR study on different selectivities of Cu(I) and Cu(II) catalysts. *ACS Catal.* **7**, 5370–5380 (2017).

36. Collins, L. R., Rajabi, N. A., Macgregor, S. A., Mahon, M. F. & Whittlesey, M. K. Experimental and computational studies of the copper borate complexes

[(NHC)Cu(HBEt$_3$)] and [(NHC)Cu(HB(C$_6$F$_5$)$_3$)]. *Angew. Chem.* **128**, 15768–15772 (2016).

37. Lemon, C. M. *et al.* Electronic structure of copper corroles. *Angew. Chem. Int. Ed.* **28**, 2216–2220 (2016).

38. Stopka, T. *et al.* Oxidative C–H bond functionalization and ring expansion with TMSCHN$_2$: A copper(I)-catalyzed approach to dibenzoxepines and dibenzoazepines. *Angew. Chem. Int. Ed.* **54**, 5049–5053 (2015).

39. Ling, L., Liu, K., Li, X. & Li, Y., General reaction mode of hypervalent iodine trifluoromethylation reagent: A density functional theory study. *ACS Catal.* **5**, 2458–2468 (2015).

40. Zhao, G. M., Liu, H. L., Zhang, D. D., Huang, X. R. & Yang, X. DFT study on mechanism of *N*-alkylation of amino derivatives with primary alcohols catalyzed by copper(II) acetate. *ACS Catal.* **4**, 2231–2240 (2014).

41. Marenich, A. V., Cramer, C. J. & Truhlar, D. G. Universal solvation model based on solute electron density and on a continuum model of the solvent defined by the bulk dielectric constant and atomic surface tensions. *J. Phys. Chem. B* **113**, 6378–6396 (2009).

42. Dolg, M., Wedig, U., Stoll, H. & Preuss, H. Energy-adjusted *ab initio* pseudopotentials for the first row transition elements. *J. Chem. Phys.* **86**, 866–872 (1987).

43. Bergner, A., Dolg, M., Küchle, W., Stoll, H. & Preuß, H. *Ab initio* energy-adjusted pseudopotentials for elements of groups 13–17. *Mol. Phys.* **80**, 1431–1441 (1993).

44. Hariharan, P. C. & Pople, J. A. The influence of polarization functions on molecular orbital hydrogenation energies. *Theor. Chem. Acta* **28**, 213–222 (1973).

45. Hay, P. J. & Wadt, W. R. *Ab initio* effective core potentials for molecular calculations. Potentials for the transition metal atoms Sc to Hg. *J. Chem. Phys.* **82**, 270–283 (1985).

46. Wadt, W. R. & Hay, P. J. *Ab initio* effective core potentials for molecular calculations. Potentials for main group elements Na to Bi. *J. Chem. Phys.* **82**, 284–298 (1985).

47. Höllwarth, A. *et al.* A set of *d*-polarization functions for pseudo-potential basis sets of the main group elements Al-Bi and f-type polarization functions for Zn, Cd, Hg. *Chem. Phys. Lett.* **208**, 237–240 (1993).

48. Grimme, S., Antony, J., Ehrlich, S. & Krieg, H. A consistent and accurate *ab initio* parametrization of density functional dispersion correction (DFT-D) for the 94 elements H-Pu. *J. Chem. Phys.* **132**, 154104 (2010).

49. Fukui, K. The path of chemical reactions - the IRC approach. *Acc. Chem. Res.* **14**, 363–368 (1981).

50. Fukui, K. Formulation of the reaction coordinate. *J. Phys. Chem.* **74**, 4161–4163 (1970).

51. Weigend, F., Furche, F. & Ahlrichs, R. Gaussian basis sets of quadruple zeta valence quality for atoms H–Kr. *J. Phys. Chem.* **119**, 12753–12762 (2003).

52. Weigend, F. & Ahlrichs, R. Balanced basis sets of split valence, triple zeta valence and quadruple zeta valence quality for H to Rn: Design and assessment of accuracy. *Phys. Chem. Chem. Phys.* **7**, 3297–3305 (2005).

53. Harvey, J. N., Aschi, M., Schwarz, H. & Koch, W. The singlet and triplet states of phenyl cation. A hybrid approach for locating minimum energy crossing points between non-interacting potential energy surfaces. *Theor. Chem. Acc.* **99**, 95–99 (1998).

54. Bryantsev, V. S., Diallo, M. S. & Goddard III, W.A. Calculation of solvation free energies of charged solutes using mixed cluster/continuum models. *J. Phys. Chem. B* **112**, 9709–9719 (2008).
