# Supplementary material for: Copper(I)-catalysed site-selective C(sp3)–H bond chlorination of ketones, (E)-enones and alkylbenzenes by dichloramine-T
Source: Nat Commun. 2021 Jul 1;12:4065. doi: 10.1038/s41467-021-23988-y (PMC8249392; doi:10.1038/s41467-021-23988-y)
Supplement: Supplementary file 3 — Description of Additional Supplementary Files [file 41467_2021_23988_MOESM3_ESM.docx]

Description of Additional Supplementary Files

Title: Supplementary Data 1

Description: Raw DFT data and XYZ coordinates
