# Supplementary material for: Copper(I)-catalysed site-selective C(sp3)–H bond chlorination of ketones, (E)-enones and alkylbenzenes by dichloramine-T
Source: Nat Commun. 2021 Jul 1;12:4065. doi: 10.1038/s41467-021-23988-y (PMC8249392; doi:10.1038/s41467-021-23988-y)
Supplement: Supplementary file 4 — Supplementary Data 1 [file 41467_2021_23988_MOESM4_ESM.pdf]

Supplementary Data 1

for

Copper(I)-Catalysed Site-Selective C(sp^3)–H Bond Chlorination of Ketones, (*E*)-Enones and Alkylbenzenes by Dichloramine-T

Jianwen Jin,[1,5] Yichao Zhao,[1,5] Sara Helen Kyne,[1] Kaveh Farshadfar,[2] Alireza Ariafard,[2,3]* and Philip Wai Hong Chan[1,4]*

[1]School of Chemistry, Monash University, Clayton, Victoria 3800, Australia

[2]Department of Chemistry, Islamic Azad University, Poonak, Tehran, Iran

[3]School of Natural Sciences–Chemistry, University of Tasmania, Hobart, Tasmania 7001, Australia

[4]Department of Chemistry, University of Warwick, Coventry CV4 7AL, United Kingdom

[5]These authors contributed equally to this work

Email:phil.chan@monash.edu (P. W H. C.); alireza.ariafard@utas.edu.au (A. A.)

Supplementary Table 1. Total potential (E), enthalpy (H) and Gibbs free energies (G) of all structures optimised at the BS1 level of theory, the total potential (E) energies calculated at the BS3//BS1 level of theory and Cartesian coordinates for all of the calculated structures.

II

E (BS1) = -462.827240710 au

H (BS1) = -462.723221 au

G (BS1) = -462.769806 au

E (BS3//BS1) = -1906.15223356 au

Cu	0.00000100	-0.00230200	-0.00040100
C	4.46812100	0.00421600	0.00058600
H	4.83298900	-0.52461600	-0.88495000
H	4.83224800	-0.49719400	0.90229300
H	4.82901700	1.03690100	-0.01525900
C	3.01916600	0.00094600	0.00024900
N	1.86234200	-0.00163100	-0.00003100
C	-4.46813600	0.00454600	0.00077400
H	-4.83099300	0.18265700	-1.01597100
H	-4.82874600	0.79780500	0.66233400
H	-4.83445000	-0.96327300	0.35580200
C	-3.01916700	0.00018300	0.00031000
N	-1.86234000	-0.00192000	-0.00056100

XI

E (BS1) = -2257.45831000 au

H (BS1) = -2257.202847 au

G (BS1) = -2257.290686 au

E (BS3//BS1) = -3701.11552016 au

Cu	3.04156200	0.34949100	-0.00378400
C	3.83662200	-4.05752300	0.00287300
H	2.98611200	-4.54749600	0.48613600
H	4.75376700	-4.28958100	0.55234100
H	3.92712400	-4.41442400	-1.02731600
C	3.62375800	-2.62418200	-0.00125700
N	3.44414100	-1.48165100	-0.00400900
C	1.65906000	4.58147500	0.00624200
H	0.56994400	4.50747300	0.07935700
H	1.92992700	5.09710900	-0.91978800
H	2.04676100	5.13987400	0.86329200
C	2.22021800	3.24635700	0.00141200
N	2.64532000	2.17097700	-0.00166500
S	-0.68130900	0.99275200	0.00972000
O	-0.33122100	1.60008900	1.29209100
O	-0.33077600	1.62029100	-1.26270000
N	0.39195400	-0.51436200	-0.00176100
Cl	0.09190700	-1.49756300	1.44417000
Cl	0.08827300	-1.47767400	-1.45991500
C	-2.35641300	0.44037300	0.00432500
C	-2.99346200	0.22178400	-1.22277100
C	-2.99536900	0.19821300	1.22738900
C	-4.30375000	-0.24624000	-1.21281100
H	-2.48447300	0.42929200	-2.15698100

C	-4.30435500	-0.26895300	1.20667800
H	-2.48722800	0.38816500	2.16577100
C	-4.97480300	-0.50346300	-0.00639700
H	-4.81529600	-0.41103900	-2.15636200
H	-4.81791000	-0.45163900	2.14606000
C	-6.38150700	-1.03897900	-0.00510200
H	-6.97399700	-0.59311500	0.79969100
H	-6.88347400	-0.85221800	-0.95813000
H	-6.37260000	-2.12358700	0.16124000

MECP1

E (BS1) = -2257.4292288100 au

29	-2.5973288	0.0025980	-0.3572775
6	-3.6079274	4.3957765	0.1136057
1	-2.7532036	4.8515341	0.6228943
1	-4.5019262	4.4950232	0.7363624
1	-3.7709872	4.8922839	-0.8474801
6	-3.3297709	2.9930262	-0.1098815
7	-3.0923626	1.8759366	-0.2859544
6	-2.5407532	-4.4854022	0.1429920
1	-1.4858424	-4.7672135	0.2147797
1	-3.0003196	-4.9902012	-0.7117313
1	-3.0617645	-4.7686567	1.0623681
6	-2.6259853	-3.0529685	-0.0358693
7	-2.6670039	-1.9070200	-0.1727314
16	0.5926164	-1.0789308	-0.4492918

8	0.1937651	-2.1135060	0.5031579
8	0.4043432	-1.2624586	-1.8930498
7	-0.5055749	0.3469275	-0.0473700
17	-0.3000643	0.7210479	2.1746979
17	0.0719461	1.7674834	-0.8756294
6	2.2383112	-0.5335092	-0.1034100
6	2.9925030	0.0568226	-1.1220500
6	2.7477987	-0.7187837	1.1875162
6	4.2871801	0.4761006	-0.8287294
1	2.5813229	0.1765594	-2.1175635
6	4.0447799	-0.2959216	1.4515134
1	2.1445347	-1.1864754	1.9558585
6	4.8292512	0.3122324	0.4556551
1	4.8860662	0.9360824	-1.6086907
1	4.4596165	-0.4395542	2.4447510
6	6.2196032	0.7907057	0.7769672
1	6.7622690	0.0515402	1.3749086
1	6.7938431	1.0003974	-0.1291670
1	6.1746226	1.7135672	1.3680222

MECP2

E (BS1) = -2257.41987923 au

29	-3.2228408	0.3421863	-0.5543233
6	-4.1763404	-3.9185707	-1.7366859
1	-3.5257277	-4.2131302	-2.5656230
1	-5.2231650	-3.9881588	-2.0477612

1	-4.0025453	-4.5769695	-0.8803646
6	-3.8796074	-2.5529642	-1.3599498
7	-3.6449526	-1.4613441	-1.0636211
6	-2.4962114	4.6622650	0.5417655
1	-1.4356789	4.7563235	0.7947576
1	-3.1065658	4.9282905	1.4101688
1	-2.7375680	5.3253647	-0.2943444
6	-2.7710671	3.2926030	0.1622668
7	-2.9840491	2.1977216	-0.1390793
16	1.7116163	0.3647969	2.0082026
8	0.9509244	1.6141160	2.0546276
8	2.5431598	-0.0484709	3.1482284
7	0.4411285	-0.8947446	1.9807232
17	-1.0896024	-0.2857634	0.2765894
17	1.0770913	-2.4384497	1.5693532
6	2.6610379	0.2708883	0.5163118
6	3.7813876	-0.5660504	0.4823583
6	2.2786524	1.0357176	-0.5925701
6	4.5296721	-0.6321660	-0.6891992
1	4.0693992	-1.1372329	1.3573048
6	3.0472277	0.9565391	-1.7496605
1	1.4134951	1.6871427	-0.5413819
6	4.1765152	0.1219966	-1.8186128
1	5.4037274	-1.2758372	-0.7267096
1	2.7724946	1.5542027	-2.6143625

6	4.9789757	0.0248958	-3.0890589
1	5.0754538	1.0012552	-3.5738700
1	5.9794577	-0.3742949	-2.9001058
1	4.4820275	-0.6446348	-3.8024785

XII

E (BS1) = -2257.43873490 au

H (BS1) = -2257.183412 au

G (BS1) = -2257.269378 au

E (BS3//BS1) = -3701.09151855 au

Cu	2.57402600	0.11571900	-0.07329900
C	3.40070500	-4.35490800	-0.16229200
H	2.55668100	-4.82115100	0.35532000
H	4.32695300	-4.56312600	0.38165500
H	3.47336200	-4.74840200	-1.18032800
C	3.18578600	-2.92556200	-0.21290500
N	2.99722100	-1.78718300	-0.24671300
C	2.23675900	4.61674300	0.28692200
H	1.16261900	4.81671300	0.35360300
H	2.64601900	5.08900700	-0.61100000
H	2.74235400	5.00786100	1.17463200
C	2.43365100	3.18751800	0.20916500
N	2.56348700	2.04217500	0.14771000
S	-0.53860900	1.19891800	-0.03709800
O	-0.19206900	1.72894400	1.28288700
O	-0.36415100	2.02466700	-1.23971300

N	0.54488900	-0.20405200	-0.21721600
Cl	0.22804200	-1.65899500	1.83893900
Cl	0.14072200	-1.13723100	-1.62992700
C	-2.17689600	0.52073900	-0.02221100
C	-2.89748700	0.45650100	-1.22058500
C	-2.71887800	0.08471300	1.18975200
C	-4.18772400	-0.06051900	-1.19227000
H	-2.46023900	0.81072500	-2.14704800
C	-4.01466400	-0.42537700	1.19197800
H	-2.13963800	0.14273700	2.10286000
C	-4.76508700	-0.50844700	0.00872000
H	-4.75940400	-0.11581200	-2.11430000
H	-4.45020500	-0.76411600	2.12736300
C	-6.17242400	-1.04425200	0.01670800
H	-6.89254300	-0.22842500	-0.12226300
H	-6.32754800	-1.75261700	-0.80384000
H	-6.40826600	-1.54448300	0.95960800

TS$_{XII-XIII}$

E (BS1) = -2257.43342217 au

H (BS1) = -2257.179224 au

G (BS1) = -2257.262437 au

E (BS3//BS1) = -3701.08665568 au

Cu	2.41665600	-0.07923700	0.11956800
C	3.26368300	-4.53022900	-0.31791000
H	2.43512700	-5.08167400	0.13678600

H	4.19304600	-4.75834400	0.21270400
H	3.36117700	-4.81044200	-1.37090700
C	2.99629700	-3.11207800	-0.22240800
N	2.77072600	-1.98244100	-0.14485600
C	2.80372700	4.43559800	0.24521500
H	1.78380300	4.83255700	0.25439300
H	3.33052000	4.78784100	-0.64665800
H	3.33607200	4.76194000	1.14360900
C	2.73729500	2.99223600	0.22448800
N	2.66324100	1.84048800	0.20849700
S	-0.63582000	1.32057200	-0.51450400
O	-0.19448300	2.13052300	0.61737200
O	-0.57566500	1.83912700	-1.88454200
N	0.48938900	-0.08234300	-0.51739300
Cl	0.56253500	-0.94843800	2.19483900
Cl	-0.01308300	-1.33769000	-1.55222900
C	-2.21993100	0.60631200	-0.19426200
C	-3.06386900	0.30930600	-1.27104800
C	-2.58650900	0.35082700	1.13123000
C	-4.30588000	-0.25337500	-1.00070000
H	-2.75676000	0.52087900	-2.28902400
C	-3.83571900	-0.21254300	1.37290800
H	-1.90808500	0.57838900	1.94453700
C	-4.71002300	-0.52322200	0.31864300
H	-4.97428400	-0.48814800	-1.82398600

H	-4.13688900	-0.41625800	2.39613700
C	-6.06817700	-1.11381400	0.58782200
H	-6.84491200	-0.34735200	0.47337100
H	-6.29779800	-1.91367000	-0.12345600
H	-6.13928900	-1.51407500	1.60246200

XIII

E (BS1) = -2257.45190503 au

H (BS1) = -2257.196634 au

G (BS1) = -2257.284353 au

E (BS3//BS1) = -3701.10586602 au

Cu	-2.00497200	1.06174200	-0.27159500
S	0.76810100	-0.56556700	1.85692500
O	0.54263800	0.78586300	2.37628000
O	0.89012400	-1.71757600	2.75336500
N	-0.67520500	-0.77849500	0.82427100
Cl	-1.07518300	0.60015600	-2.29196100
Cl	-0.90372800	-2.36897100	0.31207200
C	2.05935200	-0.57366000	0.65390400
C	2.70165100	-1.78122200	0.35239900
C	2.38499100	0.62001900	-0.00308800
C	3.69311800	-1.77848900	-0.62245700
H	2.44258300	-2.69289800	0.87923600
C	3.37993900	0.59637300	-0.97228900
H	1.88055000	1.54367900	0.24864000
C	4.04419800	-0.59790600	-1.30096400

H	4.20848300	-2.70451200	-0.85927900
H	3.64950700	1.51736700	-1.48068000
C	5.09503300	-0.61289600	-2.37725600
H	5.65257200	0.32809600	-2.40117700
H	5.79868900	-1.43842600	-2.24028400
H	4.62440600	-0.73843800	-3.36083300
C	-4.24603600	-1.05846800	-0.58443800
N	-3.44731400	-0.24196300	-0.41464800
C	-5.23460700	-2.09131500	-0.80339000
H	-4.72617400	-3.05502100	-0.90505000
H	-5.92222200	-2.12432900	0.04678900
H	-5.79000500	-1.86930100	-1.71968300
C	-0.09994900	3.35323000	0.53233500
N	-0.81557900	2.51948700	0.17884100
C	0.82475200	4.36858300	0.98528500
H	1.40912500	3.96531900	1.81798000
H	1.49201600	4.64329700	0.16312100
H	0.26646500	5.24856900	1.31811900

XV

E (BS1) = -1797.27242971 au

H (BS1) = -1797.020422 au

G (BS1) = -1797.102845 au

E (BS3//BS1) = -3240.89309860 au

Cu	2.47244700	0.21021800	-0.28567000
C	2.92403200	4.75449400	-0.06855600

H	1.91676400	5.18120200	-0.04900100
H	3.45985900	5.11636800	-0.95117300
H	3.46537500	5.04644300	0.83622000
C	2.82826000	3.31112100	-0.12594100
N	2.74808300	2.15935400	-0.17272000
C	2.50932300	-4.27047400	0.13756200
H	1.46353700	-4.46577400	0.39474100
H	3.15959900	-4.59775300	0.95401900
H	2.77101700	-4.80312300	-0.78140800
C	2.66264500	-2.84766600	-0.06518700
N	2.75509100	-1.70722700	-0.22580700
S	-0.41796100	-1.06779600	-0.28530700
O	-0.03209200	-1.28283700	-1.69099600
O	-0.28641200	-2.17153900	0.68136100
N	0.51893400	0.32122400	0.12950300
Cl	0.25558700	0.78670700	1.81965900
C	-2.08251100	-0.44059700	-0.21796100
C	-2.90367900	-0.77167500	0.86137100
C	-2.52851700	0.39054000	-1.25144500
C	-4.20055600	-0.26247300	0.89639000
H	-2.53619000	-1.41751400	1.65097500
C	-3.82636300	0.88768400	-1.19608900
H	-1.87518200	0.63544000	-2.08211200
C	-4.68105000	0.56978000	-0.12634900
H	-4.84930000	-0.51545700	1.73013200

H	-4.18532900	1.53157200	-1.99412700
C	-6.09430000	1.09058100	-0.09892300
H	-6.74779600	0.45118400	-0.70600200
H	-6.49731800	1.10397400	0.91761300
H	-6.15401900	2.10148700	-0.51372200

[TsNCl]$^{\bullet}$

E (BS1) = -1334.41735877 au

H (BS1) = -1334.272556 au

G (BS1) = -1334.327305 au

E (BS3//BS1) = -1334.71879681 au

S	1.41567700	-0.79439200	0.31019000
O	1.68415500	-2.15243600	-0.17376700
O	1.95580400	-0.32438200	1.59013400
N	2.01843500	0.18335600	-1.05301100
Cl	2.43162700	1.76749800	-0.60146000
C	-0.30921200	-0.42282000	0.19636300
C	-0.85554100	0.55160600	1.04029100
C	-1.08169100	-1.08123200	-0.76964600
C	-2.20666400	0.85509000	0.91615600
H	-0.23860800	1.04513500	1.78315200
C	-2.43013500	-0.75866300	-0.87453100
H	-0.63827000	-1.83498000	-1.41133500
C	-3.01146500	0.21203800	-0.04094800
H	-2.64795100	1.59927300	1.57265000
H	-3.04409800	-1.26761200	-1.61174200

C	-4.46385600	0.57778400	-0.18833300
H	-5.05174500	-0.26364100	-0.56582800
H	-4.89074100	0.90979000	0.76236800
H	-4.57441600	1.40308600	-0.90345000

XIV

E (BS1) = -923.019070624 au

H (BS1) = -922.912014 au

G (BS1) = -922.965343 au

E (BS3//BS1) = -2366.37664913 au

Cu	0.00520400	-0.24202600	-0.00934400
Cl	-0.02137000	2.01836900	0.00278200
C	-3.07713600	-0.60493000	-0.00372700
N	-1.92684400	-0.50229700	-0.02198700
C	-4.51827700	-0.72772100	0.01955400
H	-4.82000800	-1.25614400	0.92894800
H	-4.96379700	0.27157100	0.00835200
H	-4.84715400	-1.28897000	-0.86016700
C	3.08677100	-0.59199100	0.00991500
N	1.93645700	-0.48823000	0.01862800
C	4.52787800	-0.71644700	0.00254100
H	4.93581900	-0.23485500	0.89621900
H	4.79958900	-1.77616500	-0.00309100
H	4.92522500	-0.22872100	-0.89277500

I

E (BS1) = -1884.63061103 au

H (BS1) = -1884.485156 au

G (BS1) = -1884.561659 au

E (BS3//BS1) = -3328.33837177 au

Cu	-1.03468800	0.86485500	-0.24980900
Cl	-2.46280200	2.55852600	0.34729000
O	0.17966200	-0.51935800	-1.14305700
S	0.85891600	-1.71907400	-0.49489800
O	-0.00244000	-2.47532800	0.42515900
O	1.65975900	-2.46838600	-1.47085300
C	2.10258600	-0.89106700	0.61555900
F	2.84926300	-1.81277500	1.23449600
F	2.90909900	-0.08660200	-0.09250300
F	1.47438200	-0.15251200	1.54742600
N	-2.40798600	-0.52406100	-0.01463200
C	-3.14696300	-1.39661900	0.14015900
C	-4.06383900	-2.50055200	0.33603900
H	-4.59889100	-2.36648300	1.28064700
H	-4.78059600	-2.52767500	-0.49016000
H	-3.49668200	-3.43562800	0.36186700
N	0.52387000	2.10078400	-0.32989200
C	1.51863900	2.68155000	-0.25154400
C	2.77743600	3.39033200	-0.14435400
H	3.57752400	2.66207500	0.01826700
H	2.96639200	3.94385700	-1.06872900
H	2.73430100	4.08594200	0.69873900

CH_3CN

E (BS1) = -132.766171364 au

H (BS1) = -132.716374 au

G (BS1) = -132.744885 au

E (BS3//BS1) = -132.821594293 au

C	-1.17955700	-0.00009400	0.00008200
H	-1.55190800	0.89532800	-0.50615300
H	-1.55093400	-0.00935700	1.02899800
H	-1.55149200	-0.88657600	-0.52201200
C	0.27721800	0.00041700	-0.00043000
N	1.43833800	-0.00019100	0.00017900

OTf^-

E (BS1) = -961.572927809 au

H (BS1) = -961.537683 au

G (BS1) = -961.578329 au

E (BS3//BS1) = -961.942056843 au

O	-1.24008700	-0.90155500	-1.12938900
S	-0.91224200	0.00016700	-0.00004800
O	-1.23984300	1.42920700	-0.21589700
O	-1.23973100	-0.52729700	1.34550700
C	0.94542900	-0.00001200	-0.00006200
F	1.43304500	0.78337900	0.98187800
F	1.43187800	-1.24246500	0.18761400
F	1.43291900	0.45848100	-1.16956200

$Cl^{\bullet}$

E (BS1) = -460.135154372 au

H (BS1) = -460.132794 au

G (BS1) = -460.150832 au

E (BS3//BS1) = -460.169293711 au

Cl	0.00000000	0.00000000	0.00000000

Cl^{-}

E (BS1) = -460.357358878 au

H (BS1) = -460.354998 au

G (BS1) = -460.372382 au

E (BS3//BS1) = -460.396980269 au

Cl	0.00000000	0.00000000	0.00000000

$TsNCl_2$

E (BS1) = -1794.61577601 au

H (BS1) = -1794.468469 au

G (BS1) = -1794.522555 au

E (BS3//BS1) = -1794.95165132 au

S	-0.93375500	-0.86354800	0.50379000
O	-1.32454100	-0.56962000	1.88276000
O	-1.30350300	-2.12416100	-0.13898100
N	-1.67015900	0.37264900	-0.65722700
Cl	-3.42977500	0.08899100	-0.72189100
Cl	-1.35380300	2.01962600	-0.05900100
C	0.76807800	-0.47796500	0.22760100
C	1.35791000	-0.87040500	-0.98053700

C	1.47859800	0.21015200	1.21440600
C	2.69594100	-0.56060800	-1.19088400
H	0.78536900	-1.40241000	-1.73246600
C	2.81935600	0.50255300	0.97973700
H	0.99531400	0.50626400	2.13854600
C	3.44563900	0.12582900	-0.21869500
H	3.17052100	-0.85498300	-2.12245100
H	3.38747900	1.03301700	1.73795300
C	4.90030600	0.42811400	-0.46066600
H	5.49991800	-0.48496200	-0.35912500
H	5.05972000	0.80625500	-1.47564800
H	5.28309400	1.16278900	0.25230700

XVI

E (BS1) = -2257.45918051 au

H (BS1) = -2257.203611 au

G (BS1) = -2257.288312 au

E (BS3//BS1) = -3701.11756410 au

Cu	-1.97705100	1.77192500	0.24742200
C	1.61610700	3.97783900	-1.16790800
H	1.99494100	4.67583400	-0.41566000
H	1.46491800	4.50377100	-2.11495000
H	2.33362100	3.16245100	-1.30796500
C	0.36256600	3.40953100	-0.71669900
N	-0.61367500	2.90719100	-0.35063500
C	-5.00542300	-1.30150400	1.42305700

H	-4.47136500	-2.19929400	1.74758100
H	-5.61215700	-1.53522500	0.54352100
H	-5.65174500	-0.94732200	2.23154100
C	-4.04609100	-0.27014500	1.08292900
N	-3.27546900	0.54620400	0.80387000
S	2.61313100	-0.65771800	-0.63575000
O	2.85485900	0.62850500	-1.28917700
O	3.57201300	-1.75662000	-0.71689100
N	2.70921000	-0.18959800	1.14364200
Cl	1.49680000	1.05335400	1.50534400
Cl	2.44976200	-1.60301800	2.18566600
C	0.96872700	-1.21758700	-0.94911100
C	0.61197500	-2.53009600	-0.60980800
C	0.04366800	-0.31500900	-1.48112700
C	-0.70778100	-2.92500900	-0.79805900
H	1.34697000	-3.22327900	-0.21769700
C	-1.26784000	-0.74133800	-1.67164100
H	0.34077600	0.69299200	-1.73991100
C	-1.66707900	-2.03975800	-1.32100700
H	-1.00106400	-3.93737900	-0.53583800
H	-1.99497300	-0.04540400	-2.07869600
C	-3.09165900	-2.48757700	-1.50873100
H	-3.75139900	-1.64386600	-1.72575200
H	-3.16339500	-3.19730700	-2.34197600
H	-3.46076600	-3.00174100	-0.61553800

1s

E (BS1) = -502.882517281 au

H (BS1) = -502.646287 au

G (BS1) = -502.698721 au

E (BS3//BS1) = -503.063538103 au

C	-0.11069800	0.67259600	-0.00043700
O	-0.35328500	1.87537100	-0.00081900
C	-1.23158500	-0.35208000	-0.00011100
H	-1.09733100	-1.00463100	0.87365400
C	-2.62950900	0.26495100	-0.00018400
H	-2.73799300	0.91616900	0.87601000
C	-3.73910400	-0.79198600	-0.00028900
H	-3.62168300	-1.44217000	0.87767600
C	-5.14111400	-0.17674300	0.00094600
H	-5.29606800	0.45377200	-0.88294800
H	-5.91878100	-0.94874300	0.00047200
H	-5.29528200	0.45191800	0.88629000
C	1.30962700	0.18711600	-0.00018000
C	2.34104900	1.14175100	0.00035200
C	1.64310700	-1.17756800	-0.00043500
C	3.67547900	0.74413700	0.00066800
H	2.07402900	2.19360400	0.00052300
C	2.98094500	-1.57559400	-0.00015700
H	0.86651400	-1.93484400	-0.00088500
C	3.99826800	-0.61756300	0.00041600

H	4.46459100	1.49078600	0.00111700
H	3.22869800	-2.63313300	-0.00037900
H	5.03881900	-0.92990700	0.00066100
H	-1.09749900	-1.00515900	-0.87349800
H	-2.73786400	0.91619500	-0.87637400
H	-3.62266200	-1.44093100	-0.87930200

$TS_{1s\text{-}XVIIs}{}^{\alpha}$

E (BS1) = -1837.29350297 au

H (BS1) = -1836.916485 au

G (BS1) = -1836.999643 au

E (BS3//BS1) = -1837.77105909 au

S	0.56258300	-2.06701400	0.16362300
O	1.07993500	-3.26072900	0.84127700
O	-0.47604200	-2.17582300	-0.86758000
N	-0.25960000	-1.11806200	1.39165900
Cl	0.75382700	-0.80380600	2.78437300
C	1.91817300	-1.10949400	-0.46219700
C	1.71462300	-0.30092800	-1.58381100
C	3.14517200	-1.13804900	0.20966700
C	2.76567800	0.49282100	-2.03482300
H	0.75748700	-0.29671800	-2.09319400
C	4.17565400	-0.32374800	-0.25025600
H	3.28867500	-1.78330900	1.06868800
C	4.00270000	0.50391100	-1.37238400
H	2.61943900	1.12333200	-2.90656600

H	5.13034000	-0.32940800	0.26802700
C	5.13330500	1.37248700	-1.85851100
H	5.87480800	0.77137400	-2.39945200
H	4.77714400	2.15286400	-2.53633100
H	5.65276500	1.84736100	-1.02005600
C	-2.35192700	1.29421700	0.78073200
O	-2.78608900	1.83309200	1.79819900
C	-0.87312100	1.31097700	0.51815700
H	-0.57892900	0.08035900	0.88212300
H	-0.58590800	1.28868100	-0.53402300
C	-0.05391800	2.23544200	1.38717000
H	-0.13871200	1.91432600	2.43211700
H	-0.53192900	3.22684300	1.36183100
C	1.41733900	2.36740600	0.96671600
H	1.85208600	1.37309700	0.81911100
C	-3.27117900	0.62600500	-0.18887800
C	-4.65490200	0.71038800	0.04993100
C	-2.81217200	-0.08028500	-1.31289100
C	-5.55984600	0.10680300	-0.81768800
H	-5.00067800	1.25441200	0.92268700
C	-3.72125400	-0.69129000	-2.17728800
H	-1.75277200	-0.18103600	-1.51038700
C	-5.09370500	-0.59621200	-1.93522700
H	-6.62645400	0.17959200	-0.62580000
H	-3.35591900	-1.24173900	-3.03927400

H	-5.79917700	-1.06910700	-2.61267000
C	1.60775200	3.21219400	-0.29687800
H	1.23421300	4.23168800	-0.14243000
H	2.66557600	3.27740100	-0.57211300
H	1.07334400	2.78791300	-1.15346100
H	1.97275600	2.82723400	1.79283500

TS'$_{1s\text{-}XVIIs}$ $^{\alpha}$

E (BS1) = -1837.29457396 au

H (BS1) = -1836.917218 au

G (BS1) = -1837.002064 au

E (BS3//BS1) = -1837.77099649 au

S	-0.67307100	-1.97113900	0.16391300
O	0.42504700	-1.91783200	-0.80866900
O	-1.16526100	-3.25188400	0.68380800
N	0.04770900	-1.14537400	1.53223000
Cl	-1.10616400	-0.76929700	2.79093100
C	-2.04768700	-1.01884200	-0.43246100
C	-3.31741000	-1.23340000	0.11357600
C	-1.82649200	-0.05236300	-1.41878300
C	-4.37950300	-0.45589500	-0.33959100
H	-3.47081100	-1.99663800	0.86794000
C	-2.90534100	0.71032700	-1.85805800
H	-0.83557300	0.10646700	-1.82727800
C	-4.19212900	0.52459600	-1.32738100
H	-5.36972000	-0.61255400	0.07857400

H	-2.74516100	1.46378500	-2.62399700
C	-5.35501700	1.33829000	-1.83178900
H	-5.83119400	0.83488100	-2.68280800
H	-6.11826000	1.46634500	-1.05863900
H	-5.03245500	2.32572300	-2.17462800
C	1.64102500	1.02135200	-0.51842300
O	1.12140600	1.50951100	-1.52379500
C	0.98593600	1.20993800	0.81710600
H	1.68985800	1.28064700	1.64927000
C	-0.17326200	2.17065500	0.89456400
H	-0.69155600	2.02201400	1.84958800
H	-0.88127900	1.95322400	0.09082900
C	0.25956900	3.65428000	0.79071600
H	0.52872300	0.01978500	1.08549100
C	2.92354000	0.26285800	-0.60553900
C	3.63418700	0.30251700	-1.81735200
C	3.42834500	-0.49864500	0.46204300
C	4.83029000	-0.39563300	-1.95742700
H	3.23292300	0.88741900	-2.63845000
C	4.62138100	-1.20631300	0.31568900
H	2.88790700	-0.56485300	1.39986300
C	5.32640100	-1.15242700	-0.88981300
H	5.37581600	-0.35442000	-2.89570700
H	4.99994600	-1.80001900	1.14246300
H	6.25839800	-1.69984400	-0.99870000

C	1.18515600	4.11718400	1.91788700
H	2.14771100	3.59472400	1.89518000
H	1.39124200	5.19005100	1.83536500
H	0.73151400	3.93858300	2.90030900
H	0.74173200	3.80678800	-0.18135500
H	-0.65326700	4.26256200	0.79210900

$TS_{1s\text{-}XVIIs}{}^{\beta}$

E (BS1) = -1837.28985585 au

H (BS1) = -1836.913011 au

G (BS1) = -1836.997385 au

E (BS3//BS1) = -1837.76728914 au

S	-0.86022300	-0.28360500	1.59415300
O	0.35689800	-1.00846400	1.20264200
O	-1.26664800	-0.20987500	3.00255700
N	-0.47938200	1.34153900	1.09613400
Cl	-1.86195400	2.42252200	1.25993600
C	-2.23730800	-0.86828800	0.63016300
C	-3.53755200	-0.64691600	1.09309100
C	-1.99720500	-1.50417400	-0.59244200
C	-4.60965700	-1.06754900	0.30906300
H	-3.70596900	-0.16458400	2.04917100
C	-3.08321300	-1.91742100	-1.35857300
H	-0.98309200	-1.68137200	-0.93160600
C	-4.40199700	-1.70450700	-0.92399000
H	-5.62358200	-0.89933600	0.66069400

H	-2.90601500	-2.41554200	-2.30765300
C	-5.56645100	-2.18204500	-1.75214200
H	-5.76871000	-3.24171800	-1.55101800
H	-6.47770900	-1.62285000	-1.52212000
H	-5.35800300	-2.08889400	-2.82236200
C	2.52658900	0.70777900	-0.50149400
O	2.50287600	1.78002400	0.09007100
C	1.40018500	0.29180300	-1.44113800
H	1.10362000	-0.72968700	-1.19220900
H	1.82388400	0.24812600	-2.45792800
C	0.19374200	1.20249200	-1.45778300
H	-0.15871600	1.29372800	-0.18798100
C	3.67707400	-0.23517100	-0.32146300
C	4.66759700	0.10063200	0.61760200
C	3.80449700	-1.42848400	-1.05198800
C	5.75878200	-0.73816500	0.82679300
H	4.56185700	1.02455700	1.17666800
C	4.90180800	-2.26576200	-0.84535700
H	3.05776600	-1.71069500	-1.78606200
C	5.87820600	-1.92450000	0.09415200
H	6.51625500	-0.47098300	1.55802600
H	4.99312900	-3.18494000	-1.41663100
H	6.72922600	-2.58015800	0.25582000
C	0.37018500	2.62659700	-1.94502600
H	0.94354800	3.19537100	-1.20567200

H	1.01005900	2.58513600	-2.84157200
H	-0.69106500	0.70379500	-1.86557700
C	-0.94422000	3.33174800	-2.28647800
H	-1.49470900	2.78341700	-3.05967100
H	-1.59377200	3.41493300	-1.41013900
H	-0.75692800	4.34383000	-2.66057200

TS'$_{1s\text{-}XVIIs}$$^{\beta}$

E (BS1) = -1837.29007403 au

H (BS1) = -1836.912930 au

G (BS1) = -1836.998163 au

E (BS3//BS1) = -1837.76763090 au

S	2.27031800	-1.71998700	-0.65341400
O	2.35899600	-2.55839200	0.55055700
O	2.76947100	-2.20305100	-1.94668700
N	0.54601500	-1.54067400	-0.81572900
Cl	0.09492400	-0.38903800	-2.06833900
C	2.99790300	-0.13112900	-0.31832100
C	3.32470400	0.71314600	-1.38509700
C	3.20888100	0.25560700	1.00828400
C	3.85915000	1.96781300	-1.10667700
H	3.17276700	0.39099800	-2.40889200
C	3.74884900	1.51438500	1.26327400
H	2.96527800	-0.41894100	1.82147100
C	4.07715300	2.38866900	0.21570800
H	4.11420600	2.63160000	-1.92804200

H	3.91957400	1.82112300	2.29121000
C	4.68140400	3.74026100	0.49523000
H	5.76852200	3.71050900	0.34936600
H	4.28493200	4.49982800	-0.18592700
H	4.49300700	4.05961400	1.52386900
C	-2.60105700	-0.40163300	0.33602900
O	-2.47207200	-1.58574400	0.04638700
C	-1.48939000	0.36086700	1.04729000
H	-1.91268000	0.82123800	1.95372300
C	-0.27943500	-0.45920100	1.43186600
H	0.58524700	0.17546900	1.64332000
C	-0.42941300	-1.60193500	2.41442600
H	0.10822700	-1.03766200	0.31555500
C	-3.85219400	0.33956800	-0.02095900
C	-4.87223100	-0.36361800	-0.68526300
C	-4.04126200	1.69971200	0.27708100
C	-6.05596900	0.27586100	-1.04251300
H	-4.71675700	-1.41313400	-0.91268200
C	-5.22825600	2.33954600	-0.08218800
H	-3.26985300	2.26715500	0.78603300
C	-6.23626100	1.63051100	-0.74091800
H	-6.83820200	-0.27683600	-1.55482600
H	-5.36567700	3.39116700	0.15184600
H	-7.15936400	2.13136700	-1.01903000
C	-0.65526000	-1.08829600	3.84979000

H	-1.58173200	-0.50934100	3.92514700
H	-0.72615100	-1.93117400	4.54586800
H	0.17221600	-0.44790100	4.17526100
H	-1.25977200	-2.24443300	2.11071500
H	0.48306100	-2.20822500	2.38847400
H	-1.19280600	1.20242200	0.40973700

$TS_{1s\text{-}XVIIs}{}^{\gamma}$

E (BS1) = -1837.29186185 au

H (BS1) = -1836.914326 au

G (BS1) = -1836.997702 au

E (BS3//BS1) = -1837.76870159 au

S	2.62446600	-0.77762200	1.21405000
O	1.53172900	-1.05656800	2.15629700
O	4.02541500	-0.89590500	1.63757600
N	2.36446400	-2.00850400	0.00883200
Cl	3.48192900	-1.87845200	-1.34753400
C	2.37949700	0.82341900	0.47992700
C	3.42214400	1.41545300	-0.24051300
C	1.13888800	1.45238500	0.62144000
C	3.20081000	2.65092400	-0.84459200
H	4.38623100	0.92666800	-0.32061300
C	0.94315400	2.68874400	0.01144600
H	0.34136000	0.99785100	1.19798600
C	1.96268300	3.30353900	-0.73294400
H	4.00271300	3.11729800	-1.40981200

H	-0.01892400	3.18255800	0.11646700
C	1.73977000	4.65146500	-1.36823200
H	1.89200800	5.45099600	-0.63216500
H	2.43511600	4.82582900	-2.19409000
H	0.71671000	4.74844800	-1.74462400
C	-2.42476800	0.01232700	0.88527900
O	-1.97676400	0.94018500	1.55698800
C	-1.53103100	-1.15895000	0.52158900
H	-0.72189700	-1.19250900	1.25478900
H	-2.08112000	-2.10193400	0.57817900
C	-0.94011000	-0.97187800	-0.89295200
H	-0.49578200	0.02506200	-0.97922500
H	-1.75163800	-1.00947800	-1.63665600
C	0.08156700	-2.01185700	-1.29054400
H	1.15266400	-1.88423000	-0.53771700
C	-3.83391900	0.05776100	0.38351900
C	-4.60775200	1.19636700	0.67103500
C	-4.40523300	-0.98393700	-0.36726600
C	-5.92148900	1.29182600	0.22181500
H	-4.15811800	1.99695200	1.24915700
C	-5.72198900	-0.88607400	-0.81928800
H	-3.83092600	-1.87315000	-0.60320600
C	-6.48160200	0.24916900	-0.52565800
H	-6.51059100	2.17530000	0.45052400
H	-6.15462800	-1.69639900	-1.39866300

H	-7.50689700	0.32243000	-0.87728800
C	-0.25906900	-3.47168300	-1.11479600
H	-1.14229600	-3.72041600	-1.72220400
H	0.56493600	-4.11215200	-1.44266000
H	-0.49524300	-3.71670900	-0.07478300
H	0.54849500	-1.78294400	-2.25442200

TS'$_{1s\text{-}XVIIs}$$^{\gamma}$

E (BS1) = -1837.29439017 au

H (BS1) = -1836.916735 au

G (BS1) = -1837.001811 au

E (BS3//BS1) = -1837.77261421 au

S	-2.95326100	-0.83917300	-1.10508400
O	-1.99781300	-1.24546900	-2.14528900
O	-4.39955700	-0.93201700	-1.33874100
N	-2.57028400	-1.96435100	0.16966000
Cl	-3.41519600	-1.58911900	1.66979500
C	-2.56350800	0.80316900	-0.54586800
C	-3.56298900	1.58879000	0.03509400
C	-1.24649600	1.25808000	-0.65736700
C	-3.22707700	2.85550700	0.50803400
H	-4.57984400	1.21979800	0.10739800
C	-0.93295800	2.52448300	-0.17244600
H	-0.47364200	0.64022200	-1.09773500
C	-1.91314400	3.34167200	0.41376500
H	-3.99622700	3.47560700	0.95979800

H	0.09098700	2.87882300	-0.24898700
C	-1.56618600	4.72315400	0.90481400
H	-1.65703700	5.45206600	0.08940000
H	-2.23707000	5.04341900	1.70721500
H	-0.53564100	4.76938200	1.26942400
C	2.64218100	-0.45178400	-0.43056400
O	1.80626900	0.40376100	-0.71039100
C	2.25909100	-1.91899700	-0.32403200
H	2.78572700	-2.45327300	-1.12645700
H	2.65530800	-2.32641400	0.61281700
C	0.74474900	-2.17595100	-0.44051500
H	0.58398200	-3.25137900	-0.57454300
H	0.36806000	-1.67218300	-1.33481500
C	-0.01965300	-1.70358500	0.77928500
H	-1.27847300	-1.82047700	0.43619100
C	4.06999100	-0.06172700	-0.20434300
C	4.40029900	1.30440700	-0.23735700
C	5.08467700	-1.00526100	0.02973000
C	5.71354800	1.72024600	-0.03646100
H	3.61113400	2.02620500	-0.42078300
C	6.40148000	-0.58715200	0.22621000
H	4.85799600	-2.06554800	0.05500300
C	6.71789600	0.77363700	0.19537200
H	5.95739600	2.77845800	-0.06044700
H	7.17998400	-1.32344300	0.40322200

H	7.74353200	1.09628900	0.35110700
C	0.08550200	-2.52880100	2.03768900
H	1.11042900	-2.47464800	2.43404200
H	-0.58326000	-2.16085600	2.82162000
H	-0.13844800	-3.58379800	1.84585700
H	0.04200300	-0.62551000	0.94700600

$TS_{1s\text{-}XVIIs}{}^{\delta}$

E (BS1) = -1837.28388343 au

H (BS1) = -1836.906607 au

G (BS1) = -1836.991106 au

E (BS3//BS1) = -1837.76278829 au

S	-2.80263300	-1.43338300	-0.93712500
O	-1.70506000	-1.66739500	-1.88517000
O	-4.15543100	-1.92873900	-1.21776300
N	-2.24463200	-2.30700400	0.46445500
Cl	-3.30707200	-2.11480300	1.85578100
C	-2.89468800	0.30062700	-0.55493900
C	-4.01793900	0.80071900	0.11128600
C	-1.83094200	1.13277000	-0.91737000
C	-4.05632200	2.15535900	0.43425500
H	-4.84670200	0.14945500	0.36372100
C	-1.89295900	2.48372900	-0.58877500
H	-0.98018600	0.73099700	-1.45365700
C	-2.99914400	3.01424400	0.09513100
H	-4.92262600	2.55269800	0.95530300

H	-1.07043100	3.13674900	-0.86694400
C	-3.05885500	4.48304900	0.42460600
H	-3.36673200	5.06013000	-0.45643500
H	-3.77802300	4.68646700	1.22273800
H	-2.07818800	4.86001600	0.73149900
C	3.11110600	1.13112400	-0.27238500
O	2.89112000	2.17279700	0.33994400
C	1.36607000	-0.71243100	-0.46653800
H	0.58510800	-1.08760300	-1.13583000
H	2.10029100	-1.51790100	-0.35186100
C	0.75312400	-0.39111300	0.90223900
H	0.09077600	0.47991600	0.83372800
H	1.55073600	-0.09512000	1.60494400
C	0.02806600	-1.54728200	1.52839600
H	-1.08425500	-1.86042900	0.85605200
C	4.43136900	0.43517100	-0.13115100
C	5.34464100	0.92853500	0.81719500
C	4.78847200	-0.68235500	-0.90451900
C	6.58397500	0.31938700	0.99204600
H	5.06057500	1.79231700	1.40945900
C	6.03259200	-1.29094500	-0.73072700
H	4.10549300	-1.07964400	-1.64732300
C	6.93066200	-0.79334300	0.21687100
H	7.28068900	0.70747300	1.72961500
H	6.30028900	-2.15251600	-1.33544700

H	7.89767600	-1.26987100	0.35162400
H	-0.41922500	-1.35250300	2.50584300
H	0.53313800	-2.51566100	1.47656600
C	2.02543900	0.51306500	-1.13639600
H	2.42838500	0.22268300	-2.11213200
H	1.27496300	1.29200400	-1.29835800

TS'$_{1s\text{-}XVIIs}$ $^{\delta}$

E (BS1) = -1837.28824550 au

H (BS1) = -1836.910650 au

G (BS1) = -1836.993548 au

E (BS3//BS1) = -1837.76452658 au

S	-2.94291200	-1.08933300	-0.30716100
O	-2.41564800	-1.89780600	-1.41460800
O	-4.37889800	-1.06690700	-0.00613500
N	-2.15999200	-1.83770600	1.06333400
Cl	-2.38705700	-0.93312900	2.55643600
C	-2.35623100	0.58186900	-0.45459900
C	-3.03652700	1.60802800	0.20700600
C	-1.19554600	0.83177100	-1.19416900
C	-2.53541800	2.90607000	0.12399200
H	-3.94052100	1.39712300	0.76700600
C	-0.71413600	2.13502200	-1.26334400
H	-0.67425800	0.03186000	-1.70612400
C	-1.37043100	3.18869000	-0.60519400
H	-3.05622900	3.71043200	0.63545300

H	0.19239600	2.32530000	-1.82852800
C	-0.83318600	4.59289400	-0.70240100
H	-1.01521900	5.00583600	-1.70224300
H	-1.30555500	5.25688000	0.02645400
H	0.24975900	4.61069900	-0.54007800
C	2.53295000	-0.43832100	-1.42479600
O	1.74685700	0.25090000	-2.07205200
C	2.22650800	-2.91847200	-0.76474900
H	2.40626200	-3.91527700	-1.18256200
H	2.80609000	-2.85636100	0.16204700
C	0.71310900	-2.79822300	-0.44032400
H	0.36676000	-3.76713100	-0.06310800
H	0.15590300	-2.59332100	-1.36116500
C	0.41450300	-1.74366100	0.59404600
H	-0.89316300	-1.81318000	0.82040900
C	3.25668300	0.14390200	-0.25087500
C	2.80509800	1.36978500	0.26975700
C	4.35859100	-0.48926000	0.34806600
C	3.43474500	1.94591900	1.36927800
H	1.94913500	1.84970400	-0.19229900
C	4.99631900	0.09580100	1.44303600
H	4.73109700	-1.43021300	-0.04167900
C	4.53430200	1.31002000	1.95738200
H	3.07174000	2.88798800	1.77007000
H	5.85215800	-0.39693000	1.89496800

H	5.02818000	1.76000800	2.81400400
H	0.52456300	-0.69956400	0.30316900
H	0.79782600	-1.94170000	1.59824200
C	2.74688100	-1.89461400	-1.79700300
H	3.81697100	-2.07191100	-1.95906200
H	2.23485400	-2.04658800	-2.75144800

TsNHCl

E (BS1) = -1335.07560233 au

H (BS1) = -1334.917648 au

G (BS1) = -1334.970407 au

E (BS3//BS1) = -1335.37837954 au

S	1.48215800	-0.80174100	0.27940500
O	1.76704700	-2.10737800	-0.32573100
O	1.97342700	-0.45057400	1.61119600
N	2.28312200	0.26855700	-0.83245500
Cl	1.99144400	1.98094700	-0.52618500
C	-0.25916200	-0.45968400	0.18256800
C	-0.84632900	0.38002200	1.13142800
C	-1.00048100	-1.01109100	-0.86896500
C	-2.20651000	0.66459800	1.02222300
H	-0.25568400	0.78976100	1.94300000
C	-2.35574400	-0.71227100	-0.95804400
H	-0.52979100	-1.66909300	-1.59200500
C	-2.97813600	0.12986100	-0.01987000
H	-2.67514300	1.30994800	1.75950700

H	-2.94381400	-1.14149500	-1.76434200
C	-4.44186500	0.46112200	-0.15044500
H	-5.02468400	-0.42411800	-0.42431300
H	-4.84557600	0.87212000	0.77866400
H	-4.59555800	1.20632500	-0.94089900
H	1.92487500	0.07668500	-1.77485600

XVIIs$^{\alpha}$

E (BS1) = -502.228889298 au

H (BS1) = -502.005622 au

G (BS1) = -502.058884 au

E (BS3//BS1) = -502.410941468 au

C	-0.18102900	0.65980900	0.10016100
O	-0.44094500	1.84677400	-0.19707200
C	-1.22697400	-0.23152700	0.53019400
H	-0.98205200	-1.24300300	0.84056100
C	-2.65523000	0.17218600	0.56563500
H	-2.74750800	1.23094500	0.30599600
H	-3.03693600	0.04395900	1.59049400
C	-3.53191700	-0.68482200	-0.37914100
H	-3.16749900	-0.56648700	-1.40697200
H	-3.41122400	-1.74500500	-0.12303700
C	-5.00905200	-0.29308500	-0.30033400
H	-5.15344900	0.75874200	-0.57363200
H	-5.61586200	-0.90240300	-0.97916500
H	-5.39985200	-0.43017000	0.71485600

C	1.23985000	0.17410600	0.03626500
C	2.26205900	1.13556500	-0.03668800
C	1.58851200	-1.18700100	0.02233800
C	3.59894100	0.74980900	-0.10503000
H	1.98755700	2.18538100	-0.03650600
C	2.92774000	-1.57332500	-0.05487700
H	0.82096800	-1.95336500	0.04976100
C	3.93615400	-0.60795700	-0.11381000
H	4.37869800	1.50488400	-0.15256300
H	3.18244300	-2.62919800	-0.07212800
H	4.97795300	-0.91101700	-0.16937500

XVIIs$^{\gamma}$

E (BS1) = -502.213946013 au

H (BS1) = -501.992457 au

G (BS1) = -502.046916 au

E (BS3//BS1) = -502.396721612 au

C	0.15050300	0.67586100	-0.02739100
O	0.38799100	1.87967200	-0.03197300
C	1.27561000	-0.34333200	-0.04652500
H	1.13915600	-0.98668800	-0.92653800
C	2.67088600	0.28544100	-0.05547700
H	2.73990200	0.97612300	-0.91551100
C	3.76978800	-0.72511300	-0.10399100
H	3.57899400	-1.67432900	-0.60260000
C	5.18154700	-0.32734100	0.16771200

H	5.25759100	0.30333900	1.06449400
H	5.83558200	-1.19575300	0.30336700
H	5.60894300	0.26848000	-0.65959700
C	-1.26670900	0.18321100	-0.00414400
C	-2.30270600	1.13282200	0.00906300
C	-1.59302800	-1.18318600	0.00411500
C	-3.63489600	0.72845700	0.03011000
H	-2.04103500	2.18598900	0.00232600
C	-2.92867300	-1.58785300	0.02524600
H	-0.81267500	-1.93654600	-0.00564000
C	-3.95065800	-0.63487500	0.03833800
H	-4.42773300	1.47107000	0.04011700
H	-3.17111900	-2.64659800	0.03162200
H	-4.98949900	-0.95244600	0.05486200
H	1.15842200	-1.00627800	0.82076300
H	2.78956300	0.93170800	0.82578400

XVIIs$^{\gamma cy}$

E (BS1) = -502.235694215 au

H (BS1) = -502.011541 au

G (BS1) = -502.060137 au

E (BS3//BS1) = -502.415343546 au

C	-0.48013600	0.31201400	-0.10984200
O	-1.32578300	-0.75444600	-0.19576100
C	-1.23861400	1.60841600	-0.10818800
H	-1.19611300	2.08966100	-1.09673200

C	-2.66807400	1.14042500	0.21990700
H	-2.81488000	1.09972000	1.30507100
C	-2.69740600	-0.27888100	-0.35527000
C	-3.62941600	-1.25750200	0.32870100
H	-3.53280600	-2.25800200	-0.10422900
H	-3.40517500	-1.31400400	1.39896300
C	0.90987400	0.09009500	-0.04552700
C	1.45763200	-1.23037100	-0.06184800
C	1.82619600	1.18338100	0.03883900
C	2.82873900	-1.43121300	0.00645500
H	0.78318500	-2.07785000	-0.12797100
C	3.19430100	0.96037500	0.10587400
H	1.44599800	2.20059700	0.04727100
C	3.71465500	-0.34432800	0.09146400
H	3.21960500	-2.44587700	-0.00691400
H	3.86953600	1.81027200	0.16877500
H	4.78658900	-0.51026400	0.14356100
H	-0.84356100	2.32737200	0.61608600
H	-3.44519400	1.77467400	-0.21235700
H	-4.66649400	-0.92880500	0.20587000
H	-2.90094400	-0.24638800	-1.43469300

XVIIIs$^{\alpha}$

E (BS1) = -2386.88252664 au

H (BS1) = -2386.514371 au

G (BS1) = -2386.616517 au

E (BS3//BS1) = -3830.76597093 au

Cu	0.08426300	-0.09598900	1.55532100
Cl	0.69152900	1.22279800	3.32644300
C	2.77548500	-1.71001300	1.76982200
N	1.78778300	-1.12098000	1.67041500
C	4.01635600	-2.44975600	1.87800000
H	4.85785200	-1.75845900	1.77804700
H	4.05971100	-2.94287400	2.85334100
H	4.06065400	-3.20192900	1.08549800
C	-2.72067100	1.26071000	1.32271100
N	-1.68205200	0.76945400	1.42501400
C	-4.03250200	1.85970400	1.19075800
H	-4.67588800	1.50294100	2.00031000
H	-3.94105600	2.94757400	1.24470500
H	-4.46051300	1.57477400	0.22629200
C	1.86395600	1.72043400	-0.84112700
O	2.72356100	2.49336300	-0.36294900
C	2.21842300	0.36855900	-1.19015300
H	1.45726600	-0.31080600	-1.55398800
C	3.61818200	-0.11714700	-1.08424500
H	3.62135000	-1.18903400	-0.85489100
H	4.13302100	0.41510700	-0.27731100
C	4.41059200	0.10343300	-2.39974600
H	3.88583100	-0.39092600	-3.22632600
C	5.84114300	-0.42853600	-2.29395700

H	5.84698000	-1.50497600	-2.08658900
H	6.39348400	-0.26300200	-3.22528600
H	6.38790400	0.07005200	-1.48526200
C	0.45488100	2.20133400	-1.04043600
C	0.08300800	3.42594100	-0.45968400
C	-0.49825900	1.49006000	-1.78812100
C	-1.20736700	3.92571500	-0.61931100
H	0.82325800	3.97293300	0.11472600
C	-1.78832400	1.99362800	-1.95495500
H	-0.25008400	0.53764800	-2.23690500
C	-2.14696600	3.21180900	-1.37260000
H	-1.48304300	4.87095800	-0.16043300
H	-2.51431900	1.42483000	-2.52671700
H	-3.15253100	3.60187800	-1.50037800
O	-0.36379300	-1.23057400	-0.09232900
S	-1.38497900	-2.35692600	-0.15112000
O	-2.15038300	-2.52624700	1.09415800
O	-0.84965700	-3.55972900	-0.80284100
C	-2.61052100	-1.67760000	-1.37918500
F	-3.25442200	-0.61434500	-0.87668500
F	-3.51538500	-2.61833800	-1.67948100
F	-1.98507400	-1.30612300	-2.50624200
H	4.42165100	1.17601500	-2.62598300

XVIIIs$^{\gamma}$

E (BS1) = -2386.86300759 au

H (BS1) = -2386.492536 au

G (BS1) = -2386.600688 au

E (BS3//BS1) = -3830.74654936 au

Cu	-1.43623900	1.43575200	0.32082200
Cl	-1.16900600	3.65023800	-0.22448200
C	0.77258500	1.56085800	2.53989200
N	-0.09987300	1.56757000	1.78379000
C	1.86800500	1.55065200	3.48805200
H	1.51806000	1.15824200	4.44711500
H	2.66228400	0.91459900	3.08889700
H	2.24273600	2.56935400	3.62348600
C	-3.74114000	0.97765000	-1.73044700
N	-2.87572000	1.22253700	-1.00830900
C	-4.82662800	0.62858200	-2.62278300
H	-5.39218000	1.52605500	-2.88818000
H	-4.41630500	0.17112100	-3.52775700
H	-5.48191800	-0.08608600	-2.11628100
C	3.56606700	0.22669300	-0.22369300
O	2.95338300	0.22691500	0.84152000
C	2.97653800	0.87817500	-1.46321900
H	3.65352300	1.68974500	-1.76311100
H	2.99501100	0.15483800	-2.28759600
C	1.55166500	1.43162000	-1.24738300
H	1.29768500	2.07295700	-2.09851500
H	1.56194200	2.06234800	-0.35335400

C	0.52518500	0.34900400	-1.10270400
H	0.61328700	-0.29149600	-0.22927600
C	-0.14626300	-0.21584300	-2.31168200
H	0.57493300	-0.77428900	-2.93457800
H	-0.95073000	-0.90687500	-2.04910900
H	-0.55819500	0.57584700	-2.95010500
C	4.91949100	-0.40694100	-0.31542000
C	5.43643100	-1.04819600	0.82380000
C	5.68757900	-0.37841000	-1.49178500
C	6.69169600	-1.64924800	0.78942800
H	4.83685700	-1.06690700	1.72801700
C	6.94695900	-0.97856900	-1.52391700
H	5.31261500	0.11161000	-2.38373800
C	7.45044500	-1.61463000	-0.38598900
H	7.08096900	-2.14424200	1.67448400
H	7.53473400	-0.94981300	-2.43674200
H	8.43060100	-2.08233900	-0.41418500
O	-1.67442900	-0.51173100	0.92944700
S	-2.92135400	-1.33192500	1.21790700
O	-4.17893500	-0.57430500	1.12658900
O	-2.73741600	-2.22227000	2.37269800
C	-2.94447100	-2.48418300	-0.24588000
F	-3.18829000	-1.81567900	-1.38708200
F	-3.90549000	-3.40314400	-0.08451500
F	-1.76474800	-3.10646900	-0.36747400

XVIIIs$^{\gamma cy}$

E (BS1) = -2386.88460604 au

H (BS1) = -2386.513364 au

G (BS1) = -2386.616390 au

E (BS3//BS1) = -3830.76844555 au

Cu	0.85613200	0.86040700	1.41408500
Cl	0.13478000	2.71762100	2.53832100
C	3.13408100	2.47195600	-0.02221600
N	2.33813200	1.86982700	0.55802300
C	4.11816900	3.23442400	-0.76311300
H	4.43400600	2.66205200	-1.64016000
H	3.67233000	4.18124900	-1.08226200
H	4.98303600	3.43369900	-0.12370600
C	-1.67489100	-0.78835600	2.17978300
N	-0.72643200	-0.16919700	1.95857800
C	-2.85938100	-1.57316300	2.45045200
H	-3.05334600	-1.58146700	3.52699100
H	-3.71015100	-1.13303600	1.92229800
H	-2.69918800	-2.59389400	2.09315000
C	-1.70871900	0.85244400	-1.35397400
O	-1.74891500	2.20740800	-1.20976100
C	-0.37594300	0.39783400	-1.87476300
H	0.22107500	-0.05801200	-1.07854200
H	-0.46603200	-0.35415200	-2.66486400
C	0.25387700	1.71603400	-2.35587300

H	-0.00753800	1.90998200	-3.40202000
H	1.34191100	1.73319300	-2.25952700
C	-0.41671400	2.75660300	-1.45481000
H	0.08527700	2.79653300	-0.48069500
C	-0.56113300	4.14892900	-2.03055300
H	-1.09817700	4.11712500	-2.98429100
H	-1.10387500	4.80354600	-1.34150200
H	0.43091400	4.57969600	-2.20234100
C	-2.84384800	0.08466100	-1.02678800
C	-4.04908200	0.69141600	-0.55548000
C	-2.82619800	-1.33860100	-1.15729600
C	-5.16469900	-0.08105000	-0.26185600
H	-4.08354000	1.76953700	-0.43752600
C	-3.95374000	-2.09112900	-0.86155300
H	-1.91153100	-1.83419000	-1.46410300
C	-5.13671600	-1.47746800	-0.41585200
H	-6.07148700	0.40494500	0.09022400
H	-3.91398600	-3.17252400	-0.96765200
H	-6.01413000	-2.07434000	-0.18542000
O	1.79259600	-0.77849100	0.65666100
S	1.42316600	-2.24546200	0.47191200
O	0.02307800	-2.47634400	0.08575500
O	2.00125300	-3.11701500	1.50429000
C	2.39954800	-2.57982800	-1.07542600
F	1.98308700	-1.77893000	-2.06753200

F	2.22883500	-3.85402300	-1.45162300
F	3.70375000	-2.36160500	-0.86578100

MECPs$^{\alpha}$

E (BS1) = -2386.86431404 au

29	0.2832786	-0.7064160	1.0617454
17	1.9946755	-0.5861606	2.4971220
6	-1.1818701	-3.5565641	1.5896440
7	-0.4412457	-2.6671064	1.5762339
6	-2.1346943	-4.6507841	1.5845176
1	-2.7124041	-4.6128848	0.6560193
1	-1.6086107	-5.6073101	1.6489933
1	-2.8121474	-4.5480829	2.4371161
6	-1.2641777	1.9905968	1.9293505
7	-0.6975783	1.0026275	1.7350212
6	-1.9892092	3.2301773	2.1281696
1	-1.9046996	3.5514679	3.1698735
1	-1.5664879	3.9965843	1.4722437
1	-3.0420768	3.0742963	1.8768344
6	2.7300710	0.6562295	-0.4706103
8	3.9340805	0.6310507	-0.1947032
6	2.0384562	-0.6198155	-0.7565502
1	1.1674668	-0.6029707	-1.4050810
6	2.7598295	-1.9043093	-0.6348781
1	2.0414988	-2.7089364	-0.4389463
1	3.4836407	-1.8589613	0.1819486

6	3.5041571	-2.2236169	-1.9677274
1	2.7838150	-2.2335488	-2.7939649
6	4.2317884	-3.5665012	-1.8846658
1	3.5292511	-4.3865637	-1.6979159
1	4.7572113	-3.7786500	-2.8226446
1	4.9711211	-3.5646397	-1.0762044
6	1.9753854	1.9425277	-0.5299110
6	2.5757296	3.0825722	0.0340134
6	0.6975382	2.0549694	-1.1046943
6	1.9098160	4.3043798	0.0330892
1	3.5619736	2.9889581	0.4766763
6	0.0365118	3.2829911	-1.1151251
1	0.2042321	1.1944781	-1.5372981
6	0.6378002	4.4080463	-0.5445117
1	2.3769538	5.1762441	0.4818159
1	-0.9521397	3.3523127	-1.5558148
1	0.1188731	5.3622148	-0.5476819
8	-0.8870274	-0.8031169	-0.5845235
16	-2.3988509	-0.9613391	-0.6858127
8	-3.0924466	-0.9991846	0.6089935
8	-2.7733862	-1.9557499	-1.7004853
6	-2.8746712	0.6768059	-1.4412188
9	-2.7211763	1.6769539	-0.5606053
9	-4.1569954	0.6388769	-1.8231758
9	-2.1116947	0.9366231	-2.5142865

1	4.2152540	-1.4142306	-2.1650939

MECPs$^{\gamma}$

E (BS1) = -2386.85410275 au

29	1.4119430	-1.4130418	0.1922965
17	1.2954307	-3.6723010	0.3230767
6	0.9542746	-0.5049455	3.1937020
7	0.9813575	-1.0615537	2.1801003
6	0.9283711	0.2200382	4.4482044
1	1.2880024	1.2376731	4.2677993
1	-0.0946861	0.2531957	4.8329776
1	1.5772934	-0.2763024	5.1752765
6	3.6082549	-0.9608739	-2.0171662
7	2.6579398	-1.3734543	-1.5006492
6	4.7966142	-0.4175289	-2.6446733
1	5.4906630	-1.2261648	-2.8891760
1	4.5185495	0.1169224	-3.5572093
1	5.2703442	0.2770400	-1.9447137
6	-3.6382153	-0.0169236	0.2324010
8	-2.8061606	0.7700642	0.6737771
6	-3.2843961	-1.4755218	-0.0115603
1	-3.9297205	-2.0942309	0.6257854
1	-3.5435319	-1.7362619	-1.0442050
6	-1.8005102	-1.8062931	0.2812334
1	-1.6767921	-2.8933892	0.2536567
1	-1.5646236	-1.4573758	1.2907613

6	-0.8880212	-1.1636363	-0.7131462
1	-0.7394191	-0.0917099	-0.6065803
6	-0.7260763	-1.7451659	-2.0758828
1	-1.6478072	-1.5720443	-2.6593071
1	0.0946521	-1.2769882	-2.6264853
1	-0.5649217	-2.8271031	-2.0362804
6	-5.0304818	0.4431166	-0.0748727
6	-5.3383758	1.8033715	0.1016881
6	-6.0308038	-0.4306403	-0.5329389
6	-6.6162754	2.2817145	-0.1765792
1	-4.5602456	2.4713522	0.4561103
6	-7.3119179	0.0494064	-0.8084974
1	-5.8195184	-1.4848396	-0.6745681
6	-7.6063671	1.4040195	-0.6326540
1	-6.8435273	3.3350004	-0.0399916
1	-8.0788751	-0.6340411	-1.1607574
1	-8.6038071	1.7754032	-0.8507717
8	1.4604106	0.6323866	0.0367354
16	2.6100032	1.4602582	0.5894712
8	3.9016002	0.7496619	0.6094328
8	2.2450568	2.2347047	1.7866662
6	2.7800078	2.7010283	-0.7848743
9	3.0771729	2.0730922	-1.9346266
9	3.7648360	3.5630456	-0.5023794
9	1.6392343	3.3806098	-0.9504034

MECPs^{cy}

E (BS1) = -2386.88188529 au

29	-1.6796779	-1.0984680	0.2736775
17	-2.1068584	-3.3316609	0.0768618
6	-4.3327966	-0.3398001	-1.1955997
7	-3.3388087	-0.6372806	-0.6906380
6	-5.5840881	0.0306282	-1.8231041
1	-5.5692154	1.0990231	-2.0570478
1	-5.7127915	-0.5493212	-2.7415988
1	-6.4060814	-0.1862962	-1.1339705
6	1.0275950	-1.5136013	1.7485376
7	-0.0042419	-1.4242513	1.2405397
6	2.3210098	-1.6154245	2.3866281
1	2.2106484	-2.1397517	3.3403046
1	3.0020287	-2.1671371	1.7327698
1	2.7129995	-0.6080236	2.5527972
6	2.6092208	-0.4972315	-1.0724716
8	2.3843960	-1.7595846	-1.5500690
6	1.4378967	0.4064186	-1.3238641
1	0.9017131	0.6151652	-0.3942149
1	1.7369511	1.3736858	-1.7410549
6	0.5782256	-0.4214083	-2.2972786
1	0.8127090	-0.1633251	-3.3352977
1	-0.4954322	-0.2798033	-2.1410907
6	1.0053679	-1.8673498	-2.0123692

1	0.4242568	-2.2813941	-1.1801855
6	0.9614002	-2.8164370	-3.1915079
1	1.5616826	-2.4259557	-4.0199420
1	1.3461811	-3.8029040	-2.9129334
1	-0.0727198	-2.9342298	-3.5326471
6	3.8122258	-0.2116732	-0.4019026
6	4.8418372	-1.1930138	-0.2502972
6	4.0363570	1.0752155	0.1824276
6	6.0060825	-0.9008018	0.4470104
1	4.6990165	-2.1742545	-0.6911896
6	5.2077043	1.3438282	0.8778657
1	3.2713939	1.8411257	0.0958373
6	6.2056056	0.3655998	1.0224570
1	6.7726243	-1.6653545	0.5466777
1	5.3496204	2.3280795	1.3189839
1	7.1167282	0.5834752	1.5727117
8	-1.3842495	0.8925681	0.3797754
16	-0.9467160	1.7788064	1.5424255
8	0.4078165	1.4958968	2.0411319
8	-2.0081700	1.9979186	2.5343068
6	-0.7788299	3.3727573	0.6015159
9	0.1972894	3.2677122	-0.3112568
9	-0.4707995	4.3591478	1.4528185
9	-1.9240324	3.6782970	-0.0203125

XIXs$^{\alpha}$

E (BS1) = -2386.87793431 au

H (BS1) = -2386.507066 au

G (BS1) = -2386.611750 au

E (BS3//BS1) = -3830.75882835 au

Cu	-0.19086900	0.56618000	0.79791500
Cl	-1.61434100	0.92748200	2.43362200
C	2.26772400	3.16720600	0.53079900
N	1.24629800	2.94786700	1.03757100
C	3.54641100	3.42527400	-0.11253700
H	4.04660700	2.47020900	-0.29565200
H	3.38464200	3.94038000	-1.06371600
H	4.17050500	4.04981500	0.53328900
C	2.30783600	-0.50813600	2.43512900
N	1.30510100	-0.11133300	2.02086200
C	3.58182400	-1.01400400	2.90397700
H	3.50000700	-1.33518800	3.94579600
H	3.87260500	-1.86221700	2.27730200
H	4.33465600	-0.22482200	2.81993100
C	-2.96061700	0.22240100	-0.19539700
O	-4.02850600	0.79327700	-0.00454600
C	-1.74228200	1.06570800	-0.51240900
H	-1.15997000	0.69301000	-1.35573600
C	-1.89546900	2.54796400	-0.49074900
H	-0.91123900	3.01744100	-0.39618000

H	-2.51532500	2.85253700	0.35445100
C	-2.53856200	3.02895000	-1.82265900
H	-1.91225700	2.70910100	-2.66403900
C	-2.69960700	4.55042700	-1.83285800
H	-1.73027100	5.05170700	-1.73295600
H	-3.15771700	4.88277900	-2.77099600
H	-3.33917200	4.88433600	-1.00809500
C	-2.84536100	-1.26174100	-0.17254500
C	-3.89743400	-2.00085600	0.39530200
C	-1.72340000	-1.93914400	-0.67900100
C	-3.81915300	-3.38820100	0.47122100
H	-4.76115500	-1.47029700	0.78254300
C	-1.64830500	-3.32977100	-0.60498100
H	-0.91001000	-1.39576500	-1.14331900
C	-2.69247200	-4.05530700	-0.02665700
H	-4.63036900	-3.95278900	0.92111200
H	-0.77479300	-3.83893200	-0.99879000
H	-2.63170700	-5.13806200	0.03575300
O	0.87421300	0.30899600	-0.87411100
S	2.27181700	-0.20065300	-1.21890500
O	3.31313800	0.13677600	-0.23806700
O	2.56556700	0.02850500	-2.63770700
C	2.05068500	-2.04685400	-1.06895400
F	1.58962400	-2.37927300	0.14571900
F	3.22220700	-2.65792200	-1.26716800

F	1.17278600	-2.47745300	-1.98876500
H	-3.51269000	2.54294200	-1.93707700

XIXs$^{\gamma}$

E (BS1) = -2386.88111830 au

H (BS1) = -2386.509818 au

G (BS1) = -2386.616703 au

E (BS3//BS1) = -3830.76467166 au

Cu	-1.23681300	1.57367000	-0.02000200
Cl	-1.09513400	3.76939600	-0.27816600
C	-0.01718000	-0.09432800	2.44189400
N	-0.43239000	0.90649000	2.02639800
C	0.48653900	-1.36867300	2.91922600
H	0.33642500	-2.11641000	2.13493700
H	1.55379300	-1.28426000	3.13470500
H	-0.05779400	-1.67206900	3.81761000
C	-4.16776500	0.93218800	1.07029100
N	-3.21512600	1.47759400	0.70433200
C	-5.33780800	0.20271400	1.51997600
H	-5.71308000	0.63454000	2.45176900
H	-6.11881500	0.24785900	0.75591300
H	-5.04624500	-0.83825700	1.68571100
C	3.41741100	0.30712800	0.23904100
O	2.54969400	-0.26346400	0.89574800
C	3.15343500	1.66398700	-0.39276200
H	3.88003600	2.37868900	0.01313100

H	3.35762700	1.60473500	-1.46787300
C	1.73212600	2.22596100	-0.12604000
H	1.69487300	3.26099400	-0.46648100
H	1.54767700	2.19537400	0.94958100
C	0.71861200	1.39279500	-0.84141600
H	0.67380200	0.36525100	-0.48750600
C	0.50114200	1.56905700	-2.30173300
H	1.38144200	1.12409100	-2.79831000
H	-0.37042600	1.01563200	-2.65923500
H	0.43930400	2.61926500	-2.59112400
C	4.75902100	-0.32375200	0.04689000
C	4.97703700	-1.60019300	0.59484000
C	5.79826800	0.30580300	-0.65928000
C	6.20546800	-2.23576800	0.43904800
H	4.16856800	-2.07914900	1.13732900
C	7.02966600	-0.33214200	-0.81229600
H	5.65703200	1.29089500	-1.09042100
C	7.23508000	-1.60175000	-0.26561000
H	6.36325900	-3.22304300	0.86345600
H	7.82805600	0.16141600	-1.35850200
H	8.19420500	-2.09688600	-0.38863400
O	-1.45033900	-0.34153000	-0.74065300
S	-1.88853600	-1.65471700	-0.12124800
O	-2.45724200	-1.54981200	1.23247900
O	-0.91618800	-2.73462100	-0.35452800

C	-3.34512100	-2.05987200	-1.20563400
F	-4.27731100	-1.09618700	-1.11185400
F	-3.89256100	-3.22018200	-0.82101900
F	-2.96553700	-2.16156800	-2.48549200

XIXs$^{\gamma cy}$

E (BS1) = -2386.93597582 au

H (BS1) = -2386.562237 au

G (BS1) = -2386.666279 au

E (BS3//BS1) = -3830.81802450 au

Cu	-1.55279000	1.03226400	-1.33459000
Cl	-3.67526800	1.43142500	-2.03114200
C	-0.11496900	-1.73178200	-1.70857800
N	-0.75216200	-0.79439600	-1.46514800
C	0.67250200	-2.91110200	-2.01263000
H	1.73630700	-2.65499000	-1.99143900
H	0.48042900	-3.67805000	-1.25710000
H	0.39148100	-3.29310800	-2.99852600
C	0.78747000	3.02866000	-0.77014200
N	-0.15325200	2.37748200	-0.94947300
C	1.97953600	3.82458000	-0.54030900
H	1.87777500	4.79575600	-1.03268600
H	2.11421500	3.97393900	0.53469200
H	2.84923200	3.29837700	-0.94274500
O	2.02223300	-1.81017300	0.70633400
S	3.36668500	-1.44485500	0.18787900

O	3.76744200	-2.16721700	-1.04304200
O	4.41672500	-1.29205800	1.21520100
C	3.10272000	0.29237800	-0.41916600
F	2.18787000	0.32510800	-1.40634300
F	4.24054700	0.82682100	-0.89521000
F	2.66330600	1.08274800	0.58116100
C	-1.75975300	-1.01575000	1.56521200
O	-2.98107600	-1.16471800	1.18878100
C	-1.02349000	-2.30670000	1.65855600
H	-0.91546800	-2.52821900	2.72975600
C	-1.95259500	-3.29955100	0.93904600
H	-1.62147900	-3.45457900	-0.09022100
C	-3.32130900	-2.61410800	0.95087900
C	-4.12892100	-2.68404100	-0.32213500
H	-5.04950600	-2.10018800	-0.23966800
H	-3.54050300	-2.31762800	-1.16850700
C	-1.28576600	0.29750100	1.89679200
C	-2.18743200	1.38902500	1.93393100
C	0.07999400	0.49460500	2.19642900
C	-1.72641200	2.64892800	2.28361600
H	-3.23324600	1.23081800	1.69747000
C	0.52625200	1.76348900	2.54654700
H	0.78294400	-0.32763000	2.12353200
C	-0.37183100	2.83465900	2.59390500
H	-2.41369800	3.48789200	2.31742400

H	1.57569000	1.92176500	2.76817700
H	-0.01493600	3.82313500	2.86798900
H	-0.01638800	-2.23525000	1.24205000
H	-1.99374200	-4.26676900	1.44079700
H	-4.39587800	-3.72938300	-0.50653600
H	-3.91590000	-2.88321400	1.82796600

XXs$^{\gamma cy}$

E (BS1) = -1425.33252415 au

H (BS1) = -1424.997114 au

G (BS1) = -1425.079472 au

E (BS3//BS1) = -2868.86189071 au

Cu	-0.10018000	1.38491900	0.63226100
Cl	-0.77891900	0.42870500	2.60750200
C	-2.40010700	2.50038900	-1.15621900
N	-1.51240200	2.09826400	-0.52926800
C	-3.51899700	2.98163100	-1.94543700
H	-3.16278400	3.68749800	-2.70138500
H	-4.00288800	2.13339100	-2.43911800
H	-4.24140700	3.48065400	-1.29282000
C	2.99510600	1.72217300	0.41421600
N	1.84085100	1.63036300	0.44236600
C	4.44249300	1.82689200	0.37428800
H	4.75190400	2.79295000	0.78404700
H	4.88354400	1.02202700	0.96907300
H	4.78728700	1.74363500	-0.66024500

C	-0.52486700	-1.65006300	-0.69654200
O	-1.08071500	-2.33423000	0.23893400
C	-1.51634200	-1.08980900	-1.66088000
H	-1.50674600	-1.74271700	-2.54552900
C	-2.83326900	-1.19229900	-0.87665600
H	-3.00503300	-0.27351200	-0.30956900
C	-2.58599000	-2.35795600	0.07773900
C	-3.19869400	-2.27122000	1.45224100
H	-2.91101600	-3.12905000	2.06631000
H	-2.88823400	-1.34747700	1.94775900
C	0.90473800	-1.53905900	-0.74251600
C	1.69598900	-2.06445900	0.30535800
C	1.51829800	-0.91132700	-1.84914200
C	3.07717700	-1.96247900	0.23860800
H	1.21625700	-2.53304200	1.15714000
C	2.90348800	-0.83079600	-1.91207500
H	0.91380600	-0.50217500	-2.65085700
C	3.68002100	-1.35339100	-0.87136300
H	3.68993400	-2.35592300	1.04289800
H	3.38097300	-0.35698000	-2.76319000
H	4.76224200	-1.28108800	-0.92097300
H	-1.25304600	-0.08315000	-1.98451300
H	-3.69296600	-1.38152900	-1.52043100
H	-4.28801100	-2.27510100	1.34327900
H	-2.77464700	-3.32493500	-0.39786700

$TS_{XXs\gamma cy\text{-}II}$

E (BS1) = -1425.29680265 au

H (BS1) = -1424.962953 au

G (BS1) = -1425.047991 au

E (BS3//BS1) = -2868.82962933 au

Cu	2.83198300	-0.53269400	-0.25275100
Cl	2.56848400	1.87079500	-0.55129400
C	5.62166400	-0.88309800	1.03383400
N	4.57290400	-0.80071400	0.54966100
C	6.93652400	-0.98575600	1.63908900
H	6.95577600	-1.83035600	2.33423400
H	7.68566700	-1.14127000	0.85709700
H	7.16340300	-0.06244400	2.18048500
C	0.21585300	-1.95177600	-1.05326300
N	1.24052700	-1.47316700	-0.80138600
C	-1.07882100	-2.52760400	-1.36379000
H	-0.95527400	-3.56820900	-1.67730800
H	-1.71580900	-2.48767800	-0.47547100
H	-1.54717400	-1.95198600	-2.16639800
C	-2.40473700	0.63587300	0.11795800
O	-1.86398200	0.56643600	-0.99864100
C	-1.62575200	1.34021500	1.19942100
H	-1.76428300	0.88854000	2.18340600
C	-0.13600900	1.32520200	0.80103900
H	0.38624000	2.09343000	1.37515100

C	0.03263100	1.61039800	-0.66022700
C	-0.31092300	2.94508400	-1.22523500
H	0.10859500	3.73905800	-0.60149400
H	0.04136800	3.05180200	-2.25163300
C	-3.75125000	0.08331600	0.34448400
C	-4.36837100	-0.65231100	-0.68596700
C	-4.43110000	0.28240700	1.56009300
C	-5.63865900	-1.18614300	-0.49859000
H	-3.83925600	-0.79830000	-1.62139700
C	-5.70851200	-0.24497100	1.73724500
H	-3.97441100	0.85329500	2.36119800
C	-6.31030700	-0.98150000	0.71265400
H	-6.10886400	-1.75881200	-1.29207700
H	-6.23333200	-0.08402700	2.67382500
H	-7.30370300	-1.39643800	0.85696300
H	-2.01283200	2.36634700	1.25847100
H	0.30549800	0.35480500	1.03632400
H	-1.39943600	3.07171300	-1.21069600
H	0.38054400	0.83481300	-1.32015900

2s

E (BS1) = -962.485442198 au

H (BS1) = -962.256760 au

G (BS1) = -962.310933 au

E (BS3//BS1) = -962.700051553 au

C	-0.32968500	-0.67076200	0.34177100

O	0.04829400	-1.77008100	-0.05350300
C	0.62285100	0.27028600	1.06337000
H	0.22192500	0.43689900	2.07149400
C	2.05514000	-0.25498400	1.15631300
H	2.03697300	-1.27554800	1.55648700
H	2.62697600	0.35964600	1.85946500
C	-1.74805200	-0.23609500	0.13144200
C	-2.61618400	-1.10335300	-0.55422100
C	-2.23826200	0.99679400	0.59389400
C	-3.94383300	-0.74750500	-0.77544200
H	-2.22915400	-2.05377000	-0.90702200
C	-3.56969200	1.35189400	0.37276900
H	-1.58965000	1.68319100	1.12723500
C	-4.42349200	0.48266100	-0.31156600
H	-4.60592700	-1.42460100	-1.30755800
H	-3.94009400	2.30673800	0.73428800
H	-5.45928000	0.76198100	-0.48320500
H	0.59697200	1.24915400	0.57114000
C	4.19004700	-0.95871100	-0.03091700
H	4.71594500	-0.97777200	-0.98887400
H	4.80024800	-0.41511500	0.69719900
C	2.81330600	-0.33230500	-0.16572400
H	4.07320800	-1.99135900	0.31792200
H	2.21991700	-0.84849300	-0.92010200
Cl	3.01604200	1.38601000	-0.87238700

3a

E (BS1) = -540.969765043 au

H (BS1) = -540.727553 au

G (BS1) = -540.781995 au

E (BS3//BS1) = -541.165698121 au

C	0.20809000	0.74961400	-0.13420500
O	0.04453300	1.94740800	-0.38125600
C	-0.94639100	-0.15548500	0.08781800
C	-2.20826800	0.27694800	-0.06700000
C	-3.44704000	-0.54190600	0.14661900
H	-3.15428300	-1.55478600	0.45147500
C	-4.30314000	0.08149600	1.26587300
H	-4.60713600	1.10128100	1.00136500
H	-5.21088500	-0.51075000	1.42598500
H	-3.75193000	0.12609800	2.21104500
C	1.59969100	0.19384200	-0.03805400
C	2.66734700	1.10655100	0.03192300
C	1.88304600	-1.18267900	-0.03721300
C	3.98257500	0.65819000	0.11406400
H	2.44231700	2.16797600	0.02358200
C	3.20314600	-1.63159100	0.03405900
H	1.08538800	-1.91355800	-0.11138800
C	4.25396000	-0.71482200	0.11496700
H	4.79707600	1.37432200	0.17643200
H	3.40985000	-2.69789100	0.02588900

H	5.27981400	-1.06711300	0.17665800
H	-0.76338700	-1.17942500	0.39754100
H	-2.36171800	1.31291900	-0.37364200
C	-4.25089700	-0.63475600	-1.16448400
H	-4.55375500	0.36198400	-1.50676300
H	-3.66215600	-1.10255300	-1.96054500
H	-5.15816800	-1.23018400	-1.01378700

$TS_{3a-XXIa}$

E (BS1) = -1875.38248300 au

H (BS1) = -1874.999647 au

G (BS1) = -1875.087334 au

E (BS3//BS1) = -1875.87463629 au

S	-1.01131700	-1.62507100	-0.64864300
O	-0.19453400	-1.14742500	-1.77476100
O	-1.34571100	-3.04988300	-0.52118600
N	-0.00938000	-1.19689800	0.71048800
Cl	-0.76319900	-1.60040500	2.24179200
C	-2.50771300	-0.66764700	-0.58012700
C	-3.56638900	-1.09733700	0.22661000
C	-2.58666200	0.51633900	-1.31933400
C	-4.71391300	-0.31206600	0.29879300
H	-3.49819300	-2.02797200	0.77810700
C	-3.74819700	1.28038800	-1.24017600
H	-1.75609500	0.82898900	-1.94140800
C	-4.82380400	0.88375000	-0.42929800

H	-5.54005100	-0.63387800	0.92648700
H	-3.82011200	2.19990400	-1.81406400
C	-6.08460100	1.70592300	-0.36890600
H	-6.78944700	1.38006600	-1.14441700
H	-6.58694700	1.59585000	0.59658500
H	-5.87837500	2.76665200	-0.53902700
C	3.16911500	1.99927000	-0.45963700
O	3.87436800	2.95586500	-0.14922900
C	1.77152500	2.28058400	-0.93604500
C	0.58848600	2.07853000	-0.31503900
C	0.29740300	1.47044900	0.99882400
H	0.11653300	0.28249700	0.71561000
C	3.68462600	0.60312600	-0.45281000
C	4.99064700	0.36276500	0.00944200
C	2.89240500	-0.47040900	-0.88744000
C	5.49207800	-0.93468900	0.04111100
H	5.59138700	1.20262900	0.34358800
C	3.39747600	-1.77035000	-0.85207800
H	1.88452400	-0.29854200	-1.24418800
C	4.69458100	-2.00397100	-0.38854400
H	6.50005000	-1.11907300	0.40135900
H	2.77628500	-2.59706000	-1.18383000
H	5.08676000	-3.01677400	-0.35971400
C	1.41366700	1.38321000	2.03495100
H	1.77579500	2.38956100	2.27825100

H	1.03661300	0.92854500	2.95516400
H	2.26007300	0.78741400	1.69116500
C	-1.02773600	1.93298500	1.59733300
H	-1.82911100	1.95041500	0.85530700
H	-1.33153200	1.28734900	2.42615600
H	-0.91024100	2.94947500	1.99625100
H	1.75757200	2.80776500	-1.88935200
H	-0.29532200	2.40892300	-0.85800100

XXIa

E (BS1) = -540.339747659 au

H (BS1) = -540.110899 au

G (BS1) = -540.166810 au

E (BS3//BS1) = -540.536719087 au

C	0.15582100	-0.83032000	-0.00005900
O	0.03283400	-2.06978600	-0.00015100
C	-1.00899300	0.04102000	0.00000600
C	-2.30331400	-0.48964700	-0.00003500
C	-3.49269000	0.22170000	-0.00001300
C	-4.80691700	-0.50142000	0.00011200
H	-4.68610900	-1.58807300	0.00056400
H	-5.40355000	-0.21629800	0.87781300
H	-5.40330400	-0.21701600	-0.87799400
C	1.53524800	-0.22736400	-0.00001400
C	2.63130400	-1.10842000	0.00013600
C	1.78599300	1.15618200	-0.00014100

C	3.93770400	-0.62609300	0.00017000
H	2.43430400	-2.17526200	0.00022100
C	3.09553200	1.63985400	-0.00011500
H	0.97005100	1.87043000	-0.00027900
C	4.17470100	0.75265300	0.00004400
H	4.77199500	-1.32210400	0.00029100
H	3.27182300	2.71177200	-0.00022100
H	5.19271200	1.13202100	0.00006600
C	-3.58230900	1.71889100	-0.00000300
H	-4.14529400	2.06105900	0.87877900
H	-2.61333000	2.22085600	-0.00124900
H	-4.14750000	2.06095600	-0.87739300
H	-2.36975400	-1.57617300	-0.00005600
H	-0.86719100	1.11390500	0.00014200

XXIIa

E (BS1) = -2424.99805451 au

H (BS1) = -2424.622023 au

G (BS1) = -2424.725687 au

E (BS3//BS1) = -3868.89593991 au

Cu	-0.62655100	-0.93913200	-1.19420500
Cl	-0.59300100	-3.04135100	-2.09846900
C	2.31894900	-0.37066600	-2.10518700
N	1.21806200	-0.56271200	-1.81520700
C	3.70571700	-0.13276300	-2.44769500
H	4.07589100	0.71550300	-1.86481000

H	4.29127000	-1.02455900	-2.20750300
H	3.78627000	0.08458600	-3.51659700
C	-3.67290300	-1.09255300	-0.47610600
N	-2.55097600	-1.07725800	-0.74571800
C	-5.07438500	-1.09665200	-0.11017800
H	-5.68858500	-1.14530800	-1.01394200
H	-5.28145300	-1.96559500	0.52099700
H	-5.30166400	-0.17877700	0.43973600
C	2.31157700	-1.40906200	1.26028700
O	2.69400300	-2.56983300	1.01991600
C	0.96837100	-1.12428500	1.73435100
H	0.71854200	-0.10554800	1.99502900
C	-0.00168800	-2.12760300	1.83609900
H	0.31312000	-3.13028600	1.55376500
C	-1.31641900	-1.95777000	2.24278600
C	-2.24814000	-3.13035300	2.29662200
H	-2.67357600	-3.23977000	3.30384000
H	-3.10043000	-2.97259900	1.62173000
H	-1.75711000	-4.06745900	2.02074100
C	3.25866700	-0.25945400	1.06365500
C	4.63681900	-0.52783300	1.02578400
C	2.81446500	1.05728700	0.85334700
C	5.55409900	0.49807800	0.80681800
H	4.97096100	-1.55043700	1.16946100
C	3.73314000	2.08114300	0.61708900

H	1.75589300	1.28537700	0.82276900
C	5.10413300	1.80741400	0.60083500
H	6.61822400	0.27962900	0.79100200
H	3.37297300	3.09005600	0.43754000
H	5.81790300	2.60694700	0.42296800
O	-0.53992600	0.76435500	-0.07458800
S	-0.78800200	2.18085500	-0.56787100
O	-1.20848900	2.25209800	-1.97653100
O	0.24625800	3.11887600	-0.10739900
C	-2.30328300	2.62815500	0.41808300
F	-3.32342400	1.81282700	0.11184100
F	-2.66530300	3.88803900	0.14530200
F	-2.05222000	2.52506800	1.73077200
C	-1.90001500	-0.64194700	2.66395700
H	-2.08539700	-0.64668100	3.74750900
H	-1.27008500	0.21270800	2.42293600
H	-2.87420100	-0.48807900	2.18546400

MECP3a

E (BS1) = -2424.9825799900 au

29	-0.6595778	1.2661554	1.0706445
17	-0.5531589	3.6268089	1.1483786
6	2.1931988	0.6134489	2.1481067
7	1.0925063	0.8179658	1.8548580
6	3.5796462	0.3566479	2.4904462
1	3.9108365	-0.5580671	1.9907716

1	4.1959630	1.1948203	2.1532562
1	3.6810617	0.2427258	3.5734658
6	-3.7116521	1.0006909	0.4180260
7	-2.6295298	1.0670344	0.8232199
6	-5.0578803	0.9047960	-0.1160373
1	-5.6910393	1.6743246	0.3347087
1	-5.0297167	1.0511971	-1.1999585
1	-5.4704875	-0.0836140	0.1045572
6	2.4680146	1.3507695	-1.3563262
8	2.8763779	2.5120805	-1.2574283
6	1.0815977	1.0878730	-1.8222792
1	0.7987237	0.0750487	-2.0744185
6	0.1712706	2.1125750	-1.8979760
1	0.4941111	3.1015949	-1.5942086
6	-1.1837058	1.9423901	-2.2567750
6	-2.1062712	3.0985755	-2.1892100
1	-2.4037376	3.3649875	-3.2162508
1	-3.0367114	2.8056906	-1.6852405
1	-1.6701217	3.9664096	-1.6955961
6	3.3425955	0.1905711	-1.0342160
6	4.7276748	0.4080358	-0.9274347
6	2.8233199	-1.0912640	-0.7832062
6	5.5808127	-0.6425258	-0.6037825
1	5.1147402	1.4058315	-1.1054020
6	3.6794285	-2.1370993	-0.4397997

1	1.7569205	-1.2745042	-0.8048283
6	5.0574493	-1.9192783	-0.3597071
1	6.6508472	-0.4701346	-0.5383972
1	3.2605940	-3.1172198	-0.2319615
1	5.7240899	-2.7384259	-0.1042869
8	-0.4936317	-0.7670771	-0.0863577
16	-0.7313680	-2.1558335	0.4284391
8	-1.1031860	-2.2251079	1.8549688
8	0.2662379	-3.1386055	-0.0413500
6	-2.2905019	-2.6395959	-0.4746337
9	-3.3232011	-1.8590855	-0.1082606
9	-2.6156998	-3.9139571	-0.2108422
9	-2.1233895	-2.5093636	-1.8031238
6	-1.7559973	0.6362254	-2.6685394
1	-2.7089523	0.7668030	-3.1855151
1	-1.0742402	0.0450501	-3.2843001
1	-1.9372918	0.0534069	-1.7555464

XXIIIa

E (BS1) = -2424.99496109 au

H (BS1) = -2424.618110 au

G (BS1) = -2424.725180 au

E (BS3//BS1) = -3868.89415280 au

Cu	2.94538800	0.26168400	-0.93489300
Cl	4.95211900	0.67710600	-0.01289200
C	0.34059900	1.55023200	-2.02032300

N	1.37413200	1.20859700	-1.62333100
C	-0.96022500	1.96687100	-2.50709800
H	-1.71683100	1.26058800	-2.15069800
H	-1.19059700	2.96675400	-2.12872200
H	-0.95307400	1.98278700	-3.60114500
C	1.67098700	-2.63759400	-1.30079600
N	2.36226900	-1.71221200	-1.19787800
C	0.79244300	-3.78763500	-1.40986300
H	1.22346400	-4.52153800	-2.09698800
H	0.67256000	-4.24442500	-0.42269900
H	-0.18791000	-3.46104500	-1.77218900
C	0.32221000	2.17271000	1.42192300
O	1.34415400	2.82295600	1.63523300
C	0.34968300	0.69021400	1.67950400
H	-0.57216800	0.12650700	1.65367400
C	1.54044100	0.07181600	1.90045000
H	2.45272900	0.65990200	1.87460300
C	1.66390600	-1.34045200	2.04642500
C	3.01462500	-1.91715200	2.09463300
H	3.24043300	-2.02556700	3.17295200
H	3.03957400	-2.92797600	1.67918500
H	3.77361900	-1.26823600	1.65176100
C	-0.91876800	2.79754000	0.90658000
C	-0.87013000	4.16669900	0.57295600
C	-2.11303100	2.08289900	0.70302300

C	-1.98959200	4.80352200	0.05287100
H	0.05716100	4.70874800	0.72424600
C	-3.23340500	2.72618400	0.17932100
H	-2.18794300	1.02866700	0.92567400
C	-3.17635400	4.08225200	-0.14455000
H	-1.94396600	5.85750000	-0.20407500
H	-4.13969200	2.15547400	0.00760700
H	-4.04950400	4.57946300	-0.55719900
O	-0.87017700	-0.99514100	-0.53595100
S	-2.24571300	-1.42292300	-0.90673700
O	-2.31009100	-2.56770600	-1.84368300
O	-3.18423200	-0.31151800	-1.18423200
C	-2.88374100	-2.12826900	0.69220700
F	-2.19953900	-3.23727300	1.03035500
F	-4.18228400	-2.44532200	0.60495200
F	-2.73674700	-1.23323800	1.69529200
C	0.50214600	-2.24077400	2.12905900
H	0.78406800	-3.24120400	2.45893900
H	-0.30695600	-1.83801800	2.74254200
H	0.09846700	-2.30655800	1.10561300

XXIVa

E (BS1) = -1463.38902408 au

H (BS1) = -1463.049670 au

G (BS1) = -1463.138980 au

E (BS3//BS1) = -2906.93634020 au

Cu	1.37826800	-1.38291300	0.25954000
Cl	2.32149900	-2.05549700	-1.73144100
C	-1.58693900	-2.04746800	0.92094800
N	-0.48028600	-1.79080400	0.69328500
C	-2.97504700	-2.35900200	1.20511500
H	-3.46022200	-1.48175300	1.64185100
H	-3.48764000	-2.63073900	0.27844700
H	-3.02499200	-3.19482700	1.90928500
C	3.47491400	0.20923400	1.96423700
N	2.65453900	-0.38043700	1.39693800
C	4.49960700	0.96822900	2.65764400
H	5.43407900	0.39970600	2.66817600
H	4.65947900	1.91753600	2.13715200
H	4.18251700	1.16652500	3.68558400
C	-1.53766900	0.81152800	-1.26148300
O	-1.44689700	0.19067000	-2.31789500
C	-0.34351700	1.59785300	-0.78818800
H	-0.48965900	2.38010200	-0.05232100
C	0.88583300	1.33125800	-1.30268300
H	0.98930000	0.52416300	-2.02209700
C	2.08041800	1.98606600	-0.87736700
C	3.35050300	1.48813700	-1.42644100
H	3.37894500	1.80813900	-2.48316700
H	4.22605900	1.88797100	-0.91497000
H	3.35186600	0.38970300	-1.46340000

C	-2.78073300	0.83330900	-0.45548500
C	-3.94335600	0.27517600	-1.02064600
C	-2.82698000	1.34944500	0.85259800
C	-5.13216700	0.25321700	-0.30109600
H	-3.89412000	-0.13026800	-2.02558200
C	-4.01891300	1.31315800	1.57577700
H	-1.94023000	1.76135100	1.32132900
C	-5.17203200	0.77359500	0.99894400
H	-6.02769800	-0.16968100	-0.74587800
H	-4.04781500	1.70750800	2.58668400
H	-6.10084700	0.75540800	1.56149500
C	2.12372400	3.11843100	0.06001300
H	2.75001200	3.90580700	-0.38166200
H	1.15811900	3.52343300	0.35495800
H	2.67712100	2.78819000	0.95223700

4a

E (BS1) = -1000.56955663 au

H (BS1) = -1000.335562 au

G (BS1) = -1000.392546 au

E (BS3//BS1) = -1000.79922524 au

C	0.81445400	-0.87765400	0.02591300
O	0.69518000	-2.09840300	-0.09180100
C	-0.37966100	-0.00728500	0.21413500
C	-1.61295000	-0.52754300	0.14430500
C	2.17394100	-0.24833600	-0.00547000

C	3.29167300	-1.09980900	0.06295900
C	2.38099600	1.13738200	-0.12021500
C	4.58263500	-0.58141800	0.02998500
H	3.12485100	-2.16870700	0.14532300
C	3.67657000	1.65512900	-0.16316800
H	1.54262400	1.82096500	-0.19556300
C	4.77814700	0.79991400	-0.08377200
H	5.43708400	-1.24928300	0.09131600
H	3.82461100	2.72676100	-0.25935000
H	5.78523700	1.20634100	-0.11184500
C	-3.74101800	-0.47205700	1.43529900
H	-4.72138100	0.00458600	1.51810200
H	-3.87644000	-1.53369100	1.21411700
C	-2.89629100	0.21797400	0.36545500
H	-3.22552300	-0.37945700	2.39797300
Cl	-3.83653200	0.02400800	-1.27332500
H	-0.23129000	1.04432900	0.42440400
H	-1.71883500	-1.59103200	-0.06027900
C	-2.75502300	1.71230200	0.61717900
H	-2.22029100	1.86452800	1.56097600
H	-2.20113000	2.20712100	-0.18383400
H	-3.74074300	2.17502700	0.70394200

5a

E (BS1) = -271.589097583 au

H (BS1) = -271.454036 au

G (BS1) = -271.492225 au

E (BS3//BS1) = -271.684930686 au

C	2.42524200	0.00005400	0.00900500
H	2.83343400	-0.88503300	-0.48927400
H	2.83346400	0.88963800	-0.48111600
C	0.91535300	0.00012100	-0.01269900
C	0.19536100	1.20436800	-0.00925900
C	0.19552500	-1.20426800	-0.00927200
C	-1.20178100	1.20691400	0.00230800
H	0.73628900	2.14791900	-0.01742800
C	-1.20157700	-1.20702700	0.00229800
H	0.73660900	-2.14773500	-0.01745900
C	-1.90650700	-0.00009500	0.00859800
H	-1.73954800	2.15144400	0.00244100
H	-1.73921100	-2.15163300	0.00242400
H	-2.99304100	-0.00018600	0.01458700
H	2.80231200	-0.00481900	1.03995400

$TS_{5a\text{-}XXVa}$

E (BS1) = -1606.00550011 au

H (BS1) = -1605.729377 au

G (BS1) = -1605.797943 au

E (BS3//BS1) = -1606.39857305 au

S	-1.87562900	-1.62829900	0.06800300
O	-2.56453100	-2.08839400	-1.14530800
O	-2.36456800	-2.00833400	1.40075300

N	-0.28871800	-2.32837800	0.07560800
Cl	0.60891700	-1.99963000	-1.39825100
C	-1.72164500	0.14566200	-0.00472200
C	-1.84202000	0.88646800	1.17355800
C	-1.46617800	0.76799900	-1.23222600
C	-1.69896200	2.27218800	1.11615000
H	-2.04849300	0.38782400	2.11381600
C	-1.31055300	2.15046100	-1.26406100
H	-1.39759100	0.18490300	-2.14311200
C	-1.42382300	2.92318100	-0.09593300
H	-1.79685700	2.85503300	2.02742600
H	-1.10290400	2.64096000	-2.21100300
C	-1.26975600	4.42069000	-0.15830000
H	-2.15107300	4.87944200	-0.62328100
H	-1.15185500	4.85448200	0.83824600
H	-0.40281600	4.70121400	-0.76559300
C	1.17605800	-1.01214300	1.86933600
H	0.43659300	-1.67748300	1.04341200
H	1.57668600	-1.82770200	2.47405800
H	0.42754000	-0.40867300	2.38413900
C	2.13181200	-0.28942900	1.04784200
C	1.81144700	0.98564500	0.51976000
C	3.37321300	-0.87335100	0.69443400
C	2.70330100	1.65326800	-0.31036700
H	0.85850700	1.43666500	0.77359700

C	4.26352400	-0.19897300	-0.13262300
H	3.62253400	-1.85708300	1.08280500
C	3.93255800	1.06558900	-0.63970800
H	2.44404600	2.63135400	-0.70540100
H	5.21591200	-0.65390700	-0.38886800
H	4.62822000	1.58841100	-1.28969000

XXVa

E (BS1) = -270.935332504 au

H (BS1) = -270.813943 au

G (BS1) = -270.850199 au

E (BS3//BS1) = -271.032689581 au

C	-2.40308100	0.00000200	0.00027500
C	-0.99554700	-0.00000300	0.00021000
C	-0.25292100	-1.22062900	0.00004000
C	-0.25292100	1.22062700	0.00001500
C	1.13450500	-1.21357600	-0.00012400
H	-0.79511500	-2.16286600	0.00014000
C	1.13450700	1.21357700	-0.00016300
H	-0.79511400	2.16286500	0.00006600
C	1.84090100	0.00000100	-0.00020700
H	1.67796000	-2.15473300	-0.00018700
H	1.67795000	2.15474200	-0.00020000
H	2.92695900	-0.00000700	-0.00033000
H	-2.96265100	0.93024700	0.00003800
H	-2.96264600	-0.93024800	0.00019500

XXVIa

E (BS1) = -2155.58322320 au

H (BS1) = -2155.314269 au

G (BS1) = -2155.408535 au

E (BS3//BS1) = -3599.38442975 au

Cu	0.11320000	0.77677900	0.68789400
Cl	0.99968600	2.41308800	2.02612200
C	2.18669700	-1.39967100	1.56090100
N	1.44343000	-0.56759700	1.26691800
C	3.11062300	-2.45372000	1.92913100
H	3.20900700	-3.15248600	1.09397700
H	4.08678500	-2.01853900	2.15927500
H	2.72562100	-2.98089600	2.80714500
C	-2.22295200	2.56574600	-0.41994100
N	-1.32173300	1.99167100	0.01663400
C	-3.37028400	3.25527500	-0.97402300
H	-3.76347300	3.96479300	-0.24052200
H	-3.07161500	3.79072900	-1.88002600
H	-4.13677100	2.51449200	-1.22009100
C	1.46687100	1.77476400	-2.03627300
O	-0.67081100	-0.63247500	-0.55858400
S	-1.59383300	-1.76802700	-0.14263200
O	-1.39280600	-2.22441500	1.24112000
O	-1.71201400	-2.78005100	-1.20007200
C	-3.24985800	-0.91402400	-0.11238900

F	-3.30893900	-0.04758100	0.91207600
F	-4.22825500	-1.81570100	0.03544400
F	-3.45535300	-0.23787700	-1.25320000
H	0.62224300	1.46476100	-2.64232200
H	1.51263800	2.81013800	-1.71457000
C	2.47409400	0.85908200	-1.67355800
C	3.58334400	1.26021000	-0.86729600
C	2.41723800	-0.50790600	-2.08358300
C	4.56374700	0.35138400	-0.49785300
H	3.64260300	2.29399300	-0.53765900
C	3.40606500	-1.40595100	-1.71047600
H	1.57534600	-0.83746700	-2.68539900
C	4.48649100	-0.98730300	-0.91593700
H	5.39608300	0.67726500	0.12026400
H	3.34244300	-2.44208400	-2.03187700
H	5.25660600	-1.69523300	-0.62474400

MECP4a

E (BS1) = -2155.5786404700 au

29	-0.0689417	1.4440847	0.5136530
17	-1.5339524	3.0439077	1.2314320
6	2.1668842	3.6044758	-0.2986695
7	1.4277299	2.8070983	0.0958731
6	3.0894224	4.6049520	-0.8025054
1	3.8593763	4.1183243	-1.4080837
1	2.5412753	5.3238082	-1.4186552

1	3.5599595	5.1285766	0.0345958
6	-1.0806479	-1.0416435	2.2639004
7	-0.7763611	-0.0600321	1.7360454
6	-1.4358122	-2.2919265	2.9066706
1	-1.9119445	-2.0920956	3.8703716
1	-2.1265536	-2.8416634	2.2616942
1	-0.5291294	-2.8839672	3.0604475
6	-1.5024622	1.1598312	-1.6097608
1	-0.5741562	0.9979110	-2.1470904
8	1.0274968	0.0368804	-0.5406405
16	2.2002973	-0.7588445	0.0031500
8	2.3995824	-0.6201740	1.4550144
8	3.3872800	-0.6788929	-0.8613383
6	1.5872964	-2.5044679	-0.2270784
9	0.4968774	-2.7427471	0.5194963
9	2.5439886	-3.3708588	0.1318009
9	1.2707728	-2.7239525	-1.5137294
1	-1.9070686	2.1648410	-1.5826569
6	-2.2681425	0.0675779	-1.1591982
6	-3.5395493	0.2722922	-0.5424647
6	-1.7700814	-1.2666299	-1.2549609
6	-4.2616269	-0.7998395	-0.0455154
1	-3.9230186	1.2844161	-0.4604707
6	-2.4987937	-2.3284820	-0.7465186
1	-0.7969606	-1.4280251	-1.7021918

6	-3.7452360	-2.1037704	-0.1392471
1	-5.2275030	-0.6340366	0.4222087
1	-2.0980106	-3.3360157	-0.8045558
1	-4.3109879	-2.9388528	0.2629490

XXVIIa

E (BS1) = -2155.59974094 au

H (BS1) = -2155.328636 au

G (BS1) = -2155.419182 au

E (BS3//BS1) = -3599.39747279 au

Cu	-0.59122900	1.29529000	-0.71951300
Cl	-2.10065300	2.74542200	-1.43351400
C	1.41061700	2.95618100	1.20385000
N	0.55778700	2.64056100	0.48699900
C	2.49669200	3.30289500	2.10023000
H	3.20224200	2.46673100	2.11576700
H	3.00108600	4.20588100	1.74506000
H	2.10506200	3.47461300	3.10693000
C	-0.78729200	-0.00044000	2.57689500
N	-1.68647900	0.70885000	2.38349200
C	0.34066200	-0.88853700	2.81679300
H	1.27456700	-0.36222300	2.60310400
H	0.33438200	-1.22436800	3.85765400
H	0.26850900	-1.75220300	2.15288400
C	-1.56001900	-0.24912700	-1.83403100
H	-0.62632400	-0.66589600	-2.20484400

O	0.66325600	-0.13881400	-0.16545800
S	2.17024100	-0.13992800	-0.39115700
O	2.93498000	0.21275700	0.81716000
O	2.57897100	0.46783000	-1.66569900
C	2.40653200	-1.97298900	-0.60715600
F	2.04744200	-2.62373500	0.50822300
F	3.68763700	-2.24415200	-0.87569200
F	1.64560000	-2.41184300	-1.62181500
H	-2.13240500	0.33897400	-2.54217900
C	-2.26762700	-0.96384900	-0.81784500
C	-3.59366600	-0.58894700	-0.47260600
C	-1.65866000	-2.05554500	-0.14308100
C	-4.28282800	-1.28554500	0.50701500
H	-4.04715300	0.25801200	-0.97777600
C	-2.36093300	-2.75141700	0.82981200
H	-0.64553200	-2.33565600	-0.40307700
C	-3.66717200	-2.36436400	1.16037100
H	-5.29517300	-0.99724300	0.77158400
H	-1.89816400	-3.59101400	1.33854500
H	-4.20897200	-2.90474900	1.93107400

$TS_{XXVIIa\text{-}I}$

E (BS1) = -2155.58699706 au

H (BS1) = -2155.317150 au

G (BS1) = -2155.408865 au

E (BS3//BS1) = -3599.38782064 au

Cu	-0.16835800	1.93434500	0.19837000
Cl	-0.86418300	3.42566100	-1.34075100
C	2.88253400	2.18253000	1.05329500
N	1.78347000	2.53430000	0.92809700
C	4.24559600	1.69637500	1.17506700
H	4.95206200	2.52386500	1.06498900
H	4.38252800	1.22618900	2.15276300
H	4.41312200	0.95303000	0.38967800
C	-0.74451600	-0.24285700	2.24850200
N	-0.79573900	0.75352100	1.65870900
C	-0.65514600	-1.51426600	2.93937300
H	-0.14574000	-1.38227300	3.89818900
H	-1.65723400	-1.91853000	3.10853100
H	-0.07974100	-2.20066200	2.31262500
C	-2.36691800	0.96586200	-1.85421300
H	-1.51045100	0.76093900	-2.48631900
O	0.73467700	0.03269100	-0.88560900
S	1.89116800	-0.77136700	-0.38549000
O	2.02531700	-0.84192600	1.08542700
O	3.14932500	-0.55840400	-1.13369200
C	1.38571400	-2.50795400	-0.83760500
F	0.38307500	-2.93151800	-0.03850800
F	2.41332200	-3.35690800	-0.69681200
F	0.95125400	-2.57061800	-2.10868700
H	-2.95062900	1.85265900	-2.06419800

C	-2.79418700	0.03823700	-0.91299300
C	-3.93803100	0.31065000	-0.09954900
C	-2.12080800	-1.21608600	-0.78736100
C	-4.38755900	-0.64002000	0.79516100
H	-4.43626100	1.27030900	-0.19772000
C	-2.59831400	-2.16669900	0.09431500
H	-1.24577100	-1.39937700	-1.39696500
C	-3.72261900	-1.87678400	0.88484700
H	-5.25175400	-0.44369600	1.42059000
H	-2.10049400	-3.12450600	0.18866000
H	-4.08683300	-2.62449300	1.58342900

6a

E (BS1) = -731.184990077 au

H (BS1) = -731.057246 au

G (BS1) = -731.097540 au

E (BS3//BS1) = -731.315190291 au

C	-0.02092500	0.00000000	0.46061900
C	0.65607100	1.21010100	0.25048800
C	0.65607100	-1.21010100	0.25048900
C	1.98991000	1.21055800	-0.16112300
H	0.13427100	2.15038300	0.41203800
C	1.98990900	-1.21055900	-0.16112300
H	0.13427000	-2.15038200	0.41203800
C	2.65871900	0.00000000	-0.36863600
H	2.50737700	2.15325800	-0.31776600

H	2.50737700	-2.15325800	-0.31776500
H	3.69754300	-0.00000100	-0.68783300
C	-1.45099800	0.00000100	0.89960400
H	-1.71624900	-0.89340700	1.46550800
H	-1.71624800	0.89341000	1.46550700
Cl	-2.61299300	0.00000000	-0.55609600

III

E (BS1) = -1424.42155996 au

H (BS1) = -1424.279497 au

G (BS1) = -1424.351815 au

E (BS3//BS1) = -2868.10166891 au

O	0.14248100	-0.56473000	-1.21359200
S	-0.43507200	-1.75133200	-0.50403600
O	-1.19037200	-2.66399500	-1.38383300
O	0.47142200	-2.37967700	0.47896800
C	-1.75490300	-0.98668500	0.56429400
F	-2.45719100	-1.93900700	1.19903200
F	-1.21505300	-0.17904200	1.49831700
F	-2.61182100	-0.25525900	-0.17253000
Cu	1.04550600	1.27899200	-0.15503600
C	5.16929300	-0.39280000	0.41744400
H	5.89412100	0.10429500	-0.23396400
H	5.11325900	-1.45427300	0.15799700
H	5.48416200	-0.28843500	1.45995000
C	3.86386900	0.21404200	0.24292100

N	2.81944100	0.69248900	0.10288500
C	-3.01311600	3.18180600	-0.08671200
H	-3.24666100	3.70150700	-1.02057800
H	-3.12418200	3.87341700	0.75355700
H	-3.69455600	2.33553100	0.04159700
C	-1.65261000	2.68339100	-0.13213400
N	-0.57537500	2.26279000	-0.16124900

IV

E (BS1) = -1291.63969826 au

H (BS1) = -1291.549410 au

G (BS1) = -1291.608447 au

E (BS3//BS1) = -2735.26612239 au

O	-0.52804700	-1.04954200	-0.71483000
S	-1.70174200	-0.78992400	0.22929500
O	-2.92455100	-1.44753700	-0.24485500
O	-1.35449900	-0.90723100	1.65381700
C	-1.99285000	1.02927300	-0.03873000
F	-3.02848800	1.44086900	0.70195700
F	-0.90425700	1.73331300	0.31828500
F	-2.25431400	1.27573300	-1.32815900
Cu	1.26111100	-0.47463700	-0.35953600
C	5.52166900	0.62431000	0.37910400
H	6.11358100	0.37024400	-0.50538700
H	5.89998200	0.07065400	1.24360800
H	5.59566500	1.69871500	0.57198600

C	4.13533800	0.27005900	0.14779400
N	3.02882700	-0.01328000	-0.03759900

V

E (BS1) = -728.381232791 au

H (BS1) = -728.174971 au

G (BS1) = -728.255753 au

E (BS3//BS1) = -2171.80754329 au

Cu	-0.00046400	0.00483300	-0.00909200
C	3.60020900	1.25325200	-2.65669700
H	4.34492000	1.76167400	-2.03725700
H	3.26420600	1.92981200	-3.44792500
H	4.04988700	0.36351200	-3.10712200
C	2.46760300	0.86384500	-1.83532500
N	1.56293800	0.55430000	-1.18183000
C	-2.30959300	3.77927700	1.39535500
H	-2.90642000	3.53753900	2.27973400
H	-2.97127500	4.12065100	0.59389600
H	-1.60006100	4.57421700	1.64291000
C	-1.58507300	2.59853400	0.95898800
N	-1.00908000	1.65626100	0.61021800
C	1.59431500	-2.31059500	3.68708000
H	2.59912200	-1.95975400	3.93996400
H	1.63066200	-3.37978100	3.45870200
H	0.92724200	-2.14398900	4.53798700
C	1.10042900	-1.58228400	2.53153400

N	0.70637800	-1.00135200	1.61037400
C	-2.87980100	-2.74035900	-2.40039700
H	-3.31271900	-3.49385900	-1.73598800
H	-2.32463500	-3.23875500	-3.20043800
H	-3.68208100	-2.13793300	-2.83656700
C	-1.98234300	-1.88356600	-1.64544600
N	-1.26593100	-1.20094000	-1.04372900

VI

E (BS1) = -1557.19481798 au

H (BS1) = -1557.003141 au

G (BS1) = -1557.093024 au

E (BS3//BS1) = -3000.92844313 au

O	-0.52465300	-0.03795400	0.77733500
S	-1.71385200	0.60477800	0.12485700
O	-2.02279200	1.95869300	0.62747000
O	-1.79314000	0.41600900	-1.33854100
C	-3.09256000	-0.45664300	0.77876500
F	-4.27769800	-0.03775200	0.30348000
F	-2.91941400	-1.73647100	0.40632000
F	-3.13726200	-0.41157000	2.12057300
Cu	1.45028400	-0.06128800	-0.23603300
C	1.35481300	4.21333300	-1.90000500
H	0.51969800	4.71469700	-1.40178600
H	1.17121200	4.18941300	-2.97815300
H	2.27891600	4.76370100	-1.70083200

C	1.47112200	2.85724900	-1.39427500
N	1.55207500	1.77481500	-0.99031300
C	4.05502300	-0.64583800	3.54391300
H	3.68938500	0.01336700	4.33669200
H	5.09422900	-0.39344700	3.31358800
H	4.00076800	-1.68450200	3.88269100
C	3.23824300	-0.47732500	2.35467900
N	2.58628100	-0.34166400	1.40712700
C	0.99874600	-3.73945500	-2.95148300
H	1.83662700	-4.42943900	-2.81566900
H	0.95114500	-3.42847600	-3.99910100
H	0.06656800	-4.24421500	-2.68084300
C	1.18189000	-2.57421200	-2.10427800
N	1.32525000	-1.64525000	-1.42777200

VII

E (BS1) = -2124.66320783 au

H (BS1) = -2124.460912 au

G (BS1) = -2124.535342 au

E (BS3//BS1) = -3568.26622873 au

Cu	2.50226800	0.25145500	0.08099200
N	4.35173300	-0.05062400	0.00921600
C	5.49114000	-0.24856300	-0.02056600
C	6.91598700	-0.50808800	-0.05506100
H	7.38152600	0.10900400	-0.82871300
H	7.35223400	-0.26715400	0.91881200

H	7.08746000	-1.56592200	-0.27728800
S	-0.43990600	-0.95710900	-0.64656200
O	-0.04321200	-0.73994400	-2.03386500
O	0.02265100	-2.09703500	0.13765200
N	0.51582400	0.50017100	0.23423100
Cl	0.00215600	0.52419900	1.95372000
C	-2.13590300	-0.60970900	-0.37424700
C	-2.76040400	-1.14932800	0.76107400
C	-2.81441400	0.21292200	-1.28384500
C	-4.09837700	-0.85095000	0.97496600
H	-2.21693000	-1.79263800	1.44317700
C	-4.15281500	0.49729300	-1.03716500
H	-2.31497200	0.60592600	-2.16125800
C	-4.81130500	-0.02128600	0.09017800
H	-4.60251400	-1.26714700	1.84193800
H	-4.69640600	1.12869700	-1.73297600
C	-6.25087400	0.31215300	0.36919800
H	-6.72699800	0.79647000	-0.48691300
H	-6.81785100	-0.58792900	0.62855700
H	-6.31925100	0.99227900	1.22714800
Cl	0.00606900	2.04123200	-0.52182900

VIII

E (BS1) = -2120.44901647 au

H (BS1) = -2120.373546 au

G (BS1) = -2120.444174 au

E (BS3//BS1) = -3564.37636541 au

O	1.56472400	-1.14897000	0.33393600
S	2.97646300	-0.85179800	-0.16740200
O	3.98136400	-1.59920600	0.59826700
O	3.09637600	-0.82778000	-1.63332500
C	3.20071800	0.92309900	0.35305900
F	4.44266300	1.32961000	0.06245500
F	2.32968900	1.71773000	-0.29296700
F	2.99780000	1.05342700	1.67021300
Cu	-0.00432600	-0.33614600	-0.39481000
O	-3.84889800	1.45106300	-1.07612000
S	-2.75902900	0.91332700	-0.25296600
O	-1.59816600	0.42787900	-1.11908700
O	-2.34059100	1.67784900	0.93202100
C	-3.42101800	-0.68927200	0.42872000
F	-2.50408900	-1.28350800	1.21205700
F	-4.51736800	-0.45500200	1.16022900
F	-3.73426900	-1.52735500	-0.56763400

IX

E (BS1) = -2124.64258097 au

H (BS1) = -2124.439336 au

G (BS1) = -2124.513109 au

E (BS3//BS1) = -3568.25417042 au

Cu	2.93566500	-0.34911200	-0.21683400
N	4.79135300	-0.60582300	0.04034500

C	5.93004900	-0.77656900	0.15302200
C	7.35603300	-0.99245900	0.29116300
H	7.60590900	-1.10906100	1.34986100
H	7.63922200	-1.89777000	-0.25428400
H	7.89464100	-0.13366800	-0.12041600
S	-1.46879300	1.73560800	-0.46199100
O	-1.73616500	2.84305600	0.45004400
O	-1.13432300	1.93802900	-1.86822000
N	0.16244800	1.12621700	0.21496900
Cl	0.69952500	-0.32024800	-0.77000700
C	-2.62516700	0.42759400	-0.25180800
C	-2.76849800	-0.51806900	-1.27570600
C	-3.33900200	0.33970300	0.95079400
C	-3.65384500	-1.57180700	-1.07884500
H	-2.21879600	-0.41989200	-2.20496900
C	-4.21692200	-0.72486500	1.11803500
H	-3.22229500	1.09135800	1.72293500
C	-4.38297300	-1.69631100	0.11589900
H	-3.78576600	-2.30740700	-1.86645500
H	-4.78588900	-0.80313800	2.03956900
C	-5.30986700	-2.86212700	0.32954100
H	-6.15999400	-2.58536800	0.95963400
H	-5.68456800	-3.25428800	-0.61988500
H	-4.77741900	-3.67671200	0.83690600
Cl	0.01870000	0.66192500	1.90428900

X

E (BS1) = -2953.47731401 au

H (BS1) = -2953.289764 au

G (BS1) = -2953.369151 au

E (BS3//BS1) = -4397.38016507 au

Cu	-1.23663800	-0.48258400	0.38949100
S	1.85627800	-1.33581800	-0.07778200
O	1.69666800	-2.16744200	1.11033900
O	1.36087100	-1.73323700	-1.39166000
N	0.64013300	0.13222300	0.33935400
Cl	0.83635800	1.38340500	-0.92973400
C	3.44334100	-0.59607400	-0.16837200
C	3.89013800	-0.11202300	-1.40650900
C	4.21080400	-0.48804200	1.00054500
C	5.14001800	0.49131500	-1.46162500
H	3.28446400	-0.21993200	-2.29859600
C	5.45576600	0.12192800	0.91163000
H	3.85111100	-0.88364900	1.94313700
C	5.93523100	0.62575900	-0.31030400
H	5.50721400	0.86282600	-2.41333600
H	6.06886500	0.20655500	1.80361200
C	7.26769300	1.31952100	-0.38035600
H	7.96289200	0.92641400	0.36667000
H	7.71729400	1.22139600	-1.37227000
H	7.14109800	2.39127800	-0.18018800

Cl	1.15585400	0.84299700	1.90845600
O	-3.06310700	-1.06826300	0.57673900
S	-4.13230700	-0.64860700	-0.43387600
O	-3.64767200	-0.59674300	-1.82195200
O	-5.41143500	-1.31595500	-0.16710200
C	-4.40595600	1.13169400	0.04068300
F	-5.37135400	1.66320200	-0.71771600
F	-4.76209600	1.22220200	1.32758800
F	-3.27847500	1.84294100	-0.14060000

$TS^{s}_{XII\text{-}XIII}$

E (BS1) = -2257.41932317 au

H (BS1) = -2257.165514 au

G (BS1) = -2257.251009 au

E (BS3//BS1) = -3701.07412181 au

Cu	1.99760200	-0.21051500	-0.31584800
S	-0.92750100	-0.01005400	1.41896400
O	-0.38114200	-1.32338400	1.74116400
O	-0.84915500	1.11356800	2.35276000
N	0.26305000	0.60622800	0.13614100
Cl	0.02683000	-0.96514800	-1.43346800
Cl	-0.20847200	2.08200700	-0.57695500
C	-2.51884800	-0.11864600	0.68139700
C	-3.31903800	1.03010100	0.62700600
C	-2.93357000	-1.34354400	0.14102200
C	-4.56182600	0.93752600	0.00977400

H	-2.98599300	1.96417200	1.06446400
C	-4.18173100	-1.40556600	-0.46526500
H	-2.29876700	-2.21940300	0.20172800
C	-5.00993800	-0.27216800	-0.54598200
H	-5.19505800	1.81804500	-0.04051100
H	-4.52059500	-2.34748900	-0.88606800
C	-6.36613100	-0.36726300	-1.19051200
H	-7.09701700	-0.76241300	-0.47362900
H	-6.72225300	0.61162200	-1.52229000
H	-6.35049900	-1.04807700	-2.04690600
C	3.89576200	-2.66944400	0.03929500
N	3.20247800	-1.75316700	-0.09031300
C	4.76420200	-3.81785800	0.20579200
H	4.66057000	-4.20463800	1.22391400
H	4.48169200	-4.59417700	-0.51116500
H	5.80164500	-3.51754500	0.03187900
C	3.81506800	2.47163000	-0.31662800
N	3.18606200	1.50429800	-0.40409700
C	4.60114300	3.68588600	-0.20586200
H	4.74888500	3.92702800	0.85102100
H	5.57281000	3.53703800	-0.68575600
H	4.07272000	4.50810500	-0.69703700

XIII^{s}

E (BS1) = -2257.43492569 au

H (BS1) = -2257.179734 au

G (BS1) = -2257.263878 au

E (BS3//BS1) = -3701.08841785 au

Cu	-1.77620900	-0.50479100	0.17908400
S	0.86488400	1.03873000	-0.63912700
O	0.26373000	0.65282800	-1.91866100
O	0.83145500	2.42460600	-0.16799300
N	0.12458400	-0.11802100	0.53157400
Cl	-1.28707300	-2.65996200	0.47537800
Cl	0.28751200	0.44254900	2.16098600
C	2.48673900	0.35759300	-0.47760100
C	3.42382200	0.99886800	0.34387700
C	2.77514800	-0.84476500	-1.14409100
C	4.68404600	0.43148100	0.46876400
H	3.17194100	1.92115300	0.85536900
C	4.04296900	-1.39057700	-1.00139600
H	2.02512800	-1.32896900	-1.75978700
C	5.01417500	-0.76709800	-0.19672600
H	5.42998100	0.91951600	1.08881100
H	4.28699200	-2.31514900	-1.51549100
C	6.39120000	-1.35105200	-0.06417300
H	6.39982400	-2.41894800	-0.29718000
H	7.07595500	-0.85227000	-0.76288200
H	6.79149200	-1.20031600	0.94287400
C	-2.35625200	2.51800300	-0.10181900
N	-2.13025500	1.38853000	-0.02925600

C	-2.62141500	3.93538400	-0.19449100
H	-1.66798000	4.47081900	-0.23159900
H	-3.19705100	4.13809200	-1.10251600
H	-3.19068800	4.25285400	0.68441300
C	-4.74956600	-1.23959700	-0.41478600
N	-3.64938700	-0.97027500	-0.19139200
C	-6.12820300	-1.58061500	-0.69591100
H	-6.24733300	-1.73511700	-1.77260900
H	-6.38828300	-2.49911300	-0.16131800
H	-6.77757300	-0.76411800	-0.36680600

I[i]

E (BS1) = -1055.80617644 au

H (BS1) = -1055.645774 au

G (BS1) = -1055.710922 au

E (BS3//BS1) = -2499.21347477 au

Cu	-0.01803600	-0.37439700	-0.12136700
Cl	-0.09614900	-2.65658700	-0.21616200
N	1.96968700	-0.37175700	0.06411200
C	3.11723400	-0.38364800	0.19316400
C	4.55655900	-0.40119100	0.35688200
H	4.80889800	-0.94579800	1.27167300
H	5.01175700	-0.90072400	-0.50337700
H	4.92779600	0.62534800	0.42538400
N	-2.00377300	-0.25296900	0.06013600
C	-3.15032100	-0.20808400	0.19083500

C	-4.58868900	-0.15897800	0.35700400
H	-4.91230900	0.88321300	0.43025900
H	-5.06772800	-0.63355800	-0.50443300
H	-4.86435400	-0.69520400	1.27002000
N	0.05330600	1.70678600	-0.12342900
C	0.11461000	2.86077900	-0.12094900
C	0.19557300	4.30804500	-0.11804500
H	1.09157500	4.61962100	0.42684700
H	0.25028300	4.66903700	-1.14924900
H	-0.69267500	4.72161100	0.36818300

I[ii]

E (BS1) = -2713.44703957 au

H (BS1) = -2713.316349 au

G (BS1) = -2713.400643 au

E (BS3//BS1) = -4157.45482514 au

Cu	-0.10511800	-0.40866700	-0.18245600
Cl	-0.27233600	-2.63728400	-0.40810200
C	0.65071700	2.62603200	-0.06212400
N	0.20773400	1.56408000	-0.14704300
C	1.21977300	3.95226300	0.05139800
H	1.40845000	4.17113300	1.10615900
H	2.16401200	3.98230700	-0.49961600
H	0.52710800	4.69103400	-0.36122600
O	3.84499400	-0.87345000	1.80419300
S	2.92142900	-0.05709700	1.00494600

O	1.51071000	-0.62520300	1.04003100
O	2.99829100	1.40436100	1.16177100
C	3.42632100	-0.36053000	-0.76077100
F	2.58751500	0.26750400	-1.60557100
F	4.66353500	0.10688000	-0.96694300
F	3.40513400	-1.66944400	-1.03956400
O	-4.19607700	0.42983700	-1.62005000
S	-3.06745500	0.61088900	-0.69734700
O	-1.82798300	-0.11895300	-1.20391400
O	-2.81244500	1.96064900	-0.17539800
C	-3.52367300	-0.37968100	0.81304600
F	-2.53081000	-0.33964300	1.72329800
F	-4.62673700	0.13478500	1.37153300
F	-3.75618300	-1.65730100	0.49380500

I[iii]

E (BS1) = -1751.83990765 au

H (BS1) = -1751.747730 au

G (BS1) = -1751.811974 au

E (BS3//BS1) = -3195.49062309 au

Cu	-0.94878900	0.53200700	-0.23184100
Cl	-1.99775600	2.32211200	0.59497000
O	0.95943800	1.35281100	-0.38825600
S	1.57543800	0.02763600	-0.80555900
O	0.38625400	-0.89161100	-1.01468800
O	2.60470900	0.04965300	-1.83507500

C	2.36896900	-0.59994400	0.76395400
F	2.86564900	-1.81762800	0.55801600
F	3.34304900	0.23324700	1.12461500
F	1.44262800	-0.65066900	1.72676100
C	-3.35203200	-1.45351700	-0.02879100
N	-2.45065900	-0.74269100	-0.15637300
C	-4.47764400	-2.34816500	0.14138500
H	-5.13488300	-2.27070000	-0.72978400
H	-4.11358800	-3.37477300	0.23923900
H	-5.02811500	-2.06357400	1.04335300

I^{iv}

E (BS1) = -2580.65887395 au

H (BS1) = -2580.580385 au

G (BS1) = -2580.655672 au

E (BS3//BS1) = -4024.61066552 au

Cu	-0.20568100	1.06562300	0.06484800
Cl	-0.07421200	3.27601100	-0.15106100
O	-2.13906800	1.01688400	0.89327000
S	-2.21846100	-0.49381500	0.79951900
O	-0.84135900	-0.92470600	0.34115900
O	-2.81799000	-1.21201700	1.91731800
C	-3.30339000	-0.79512500	-0.68632900
F	-3.33008100	-2.09917700	-0.95919400
F	-4.53716500	-0.36149100	-0.43115300
F	-2.80369900	-0.13033700	-1.73322900

O	3.94030800	0.74515600	-0.87823800
S	2.79529800	0.32564100	-0.06319000
O	2.75933700	0.71570300	1.35437900
O	1.46917200	0.55639300	-0.79736700
C	2.85943200	-1.53407600	-0.05039300
F	1.87072700	-2.02583500	0.70508800
F	2.73448000	-2.01196000	-1.29425500
F	4.03213000	-1.93881600	0.45522500

Supplementary Table 2. Total potential (E), enthalpy (H) and Gibbs free energies (G) of all structures optimised at the BS2 level of theory, the total potential (E) energies calculated at the BS3//BS2 level of theory and Cartesian coordinates for all of the calculated structures.

II

E (BS2) = -461.619187469 au

H (BS2) = -461.515831 au

G (BS2) = -461.560943 au

E (BS3//BS2) = -1906.15196080 au

Cu	0.00000000	-0.00167100	-0.00089100
C	-4.50328100	0.00258300	0.00202400
H	-4.86955600	-1.00842600	0.20839400
H	-4.87005200	0.33054300	-0.97609700
H	-4.86688000	0.68699800	0.77547400
C	-3.05193900	0.00088400	-0.00020500
N	-1.89371900	-0.00044500	-0.00138900
C	4.50331100	0.00309500	0.00123900
H	4.86987400	-0.62526200	0.81951700

H	4.86674300	1.02698000	0.13683400
H	4.86971800	-0.39030600	-0.95266300
C	3.05193900	0.00036700	0.00082600
N	1.89371700	-0.00150600	0.00011400

III

E (BS1) = -1423.21266473 au

H (BS2) = -1423.070210 au

G (BS2) = -1423.143004 au

E (BS3//BS2) = -2868.10111409 au

O	0.14662900	-0.55753300	-1.20133400
S	-0.46536200	-1.74581200	-0.52690000
O	-1.27762100	-2.59089100	-1.42388100
O	0.43280500	-2.45085100	0.41013800
C	-1.72835900	-0.96953600	0.59672600
F	-2.41165200	-1.91346700	1.26414700
F	-1.13941200	-0.16476900	1.50287600
F	-2.61143800	-0.23192700	-0.10306500
Cu	1.05671900	1.28349300	-0.20039500
C	5.19870100	-0.40940000	0.47172300
H	5.94781800	0.09318900	-0.14870100
H	5.14569600	-1.46694300	0.19274100
H	5.48441600	-0.32568100	1.52534100
C	3.90112500	0.21010000	0.26812000
N	2.86377400	0.69925500	0.10356600
C	-3.07341800	3.08798300	-0.02661300

H	-3.39484800	3.53130000	-0.97451600
H	-3.17545200	3.82742600	0.77428300
H	-3.69678300	2.21647900	0.19864900
C	-1.68992100	2.66007500	-0.12770500
N	-0.59387000	2.29306500	-0.19991600

IV

E (BS2) = -1290.43444909 au

H (BS2) = -1290.343941 au

G (BS2) = -1290.403159 au

E (BS3//BS2) = -2735.26619189 au

O	-0.54805400	-1.04874800	-0.71556600
S	-1.70951200	-0.79160900	0.23512600
O	-2.94146200	-1.45060200	-0.21675300
O	-1.34821100	-0.89888900	1.65762500
C	-2.00444900	1.02399800	-0.03714400
F	-3.01342900	1.44937900	0.73381400
F	-0.90073000	1.72512900	0.27836200
F	-2.30840000	1.26473300	-1.31883000
Cu	1.25607300	-0.46632800	-0.37756900
C	5.56378800	0.60892100	0.40229200
H	6.16823800	0.35092500	-0.47355400
H	5.92700700	0.05212000	1.27233200
H	5.64567200	1.68323100	0.59739700
C	4.17585200	0.26580600	0.15096400
N	3.06840900	-0.00808700	-0.04970100

V

E (BS2) = -727.164879956 au

H (BS2) = -726.960633 au

G (BS2) = -727.030651 au

E (BS3//BS2) = -2171.80662910 au

Cu	0.00812500	-0.00423700	0.00210000
C	-2.76925100	0.49120400	3.74845000
H	-3.60297500	1.15668900	3.50252300
H	-2.19965000	0.91658500	4.58098000
H	-3.16212700	-0.48780100	4.04141500
C	-1.90345200	0.34332500	2.58900600
N	-1.21325600	0.22549900	1.66542500
C	2.43358300	3.94164000	-0.72319000
H	2.73585500	3.99737900	-1.77389400
H	3.32538600	3.95775800	-0.08837200
H	1.80228400	4.80296900	-0.48193800
C	1.69182700	2.71177800	-0.49288900
N	1.09827500	1.73331300	-0.30969200
C	-2.73644400	-0.90288300	-3.69561600
H	-3.69435100	-0.38629100	-3.57701400
H	-2.91222100	-1.98162200	-3.75922100
H	-2.25186500	-0.56036300	-4.61562200
C	-1.88099800	-0.61381900	-2.55528500
N	-1.20110600	-0.38367200	-1.64532600
C	3.04765300	-3.51072100	0.66686400

H	3.26390200	-4.00486600	-0.28586200
H	2.63582800	-4.24265400	1.36914400
H	3.97366500	-3.09604600	1.07806400
C	2.08572500	-2.44032000	0.45572500
N	1.32162700	-1.58515300	0.28823800

VI

E (BS2) = -1555.98220554 au

H (BS2) = -1555.790868 au

G (BS2) = -1555.875739 au

E (BS3//BS2) = -3000.92767211 au

O	-0.47510200	-0.02160300	0.70258300
S	-1.72412900	0.51883500	0.07148200
O	-2.10642000	1.86764800	0.53664900
O	-1.83657600	0.27102600	-1.38023100
C	-3.01110700	-0.59898400	0.80987400
F	-4.23573300	-0.26943900	0.36399400
F	-2.77506500	-1.87943900	0.47599600
F	-3.01368700	-0.50819300	2.15042600
Cu	1.49831200	0.02536600	-0.28921200
C	0.91357000	4.37799900	-1.79029800
H	0.03570000	4.74312300	-1.24691200
H	0.70203400	4.38513900	-2.86447700
H	1.76579000	5.03353700	-1.58454800
C	1.21867600	3.02329700	-1.36007100
N	1.45182900	1.94301700	-1.01084300

C	3.85238200	-0.53194700	3.70036100
H	3.38209800	0.11732300	4.44607000
H	4.90953600	-0.26457000	3.60323100
H	3.77127500	-1.57409800	4.02592100
C	3.18429800	-0.36639700	2.41936100
N	2.64722600	-0.23543000	1.40054100
C	1.23180900	-3.80012500	-2.92016000
H	2.09781000	-4.44726900	-2.74752900
H	1.17194400	-3.55081100	-3.98454800
H	0.32049700	-4.32856000	-2.62124000
C	1.36699500	-2.58171900	-2.13800800
N	1.47290800	-1.60851200	-1.51750600

VII

E (BS2) = -2123.45052308 au

H (BS2) = -2123.246836 au

G (BS2) = -2123.321317 au

E (BS3//BS2) = -3568.26585136 au

Cu	2.50572200	0.16428300	0.02712800
N	4.40059200	-0.10899500	-0.04237000
C	5.55927600	-0.14950700	-0.05947000
C	7.00921400	-0.21444500	-0.09868200
H	7.33410200	-0.48997200	-1.10761000
H	7.42929300	0.76155300	0.16547600
H	7.35904500	-0.96768800	0.61523000
S	-0.47418800	-1.14168100	-0.24801800

O	-0.04652400	-1.39810800	-1.61979600
O	-0.05606100	-1.97356500	0.87641200
N	0.49982700	0.49907900	0.13944600
Cl	0.03845800	1.04935800	1.77938900
C	-2.16699700	-0.69194800	-0.15935100
C	-2.83003300	-0.78797200	1.07198700
C	-2.80163300	-0.21617300	-1.31726400
C	-4.16212000	-0.39417300	1.13188000
H	-2.32412500	-1.17373500	1.94965400
C	-4.13082300	0.17229300	-1.22336000
H	-2.27353800	-0.16649900	-2.26270900
C	-4.82794600	0.09765300	-0.00334200
H	-4.69451000	-0.47147200	2.07503400
H	-4.64075900	0.53560000	-2.11079900
C	-6.25987600	0.55238400	0.07603000
H	-6.83591200	0.19208500	-0.78371900
H	-6.74293400	0.20752900	0.99490300
H	-6.30773000	1.64948800	0.05987600
Cl	0.00869100	1.74240300	-1.05331700

VIII

E (BS2) = -2119.24728033 au

H (BS2) = -2119.171693 au

G (BS2) = -2119.241737 au

E (BS3//BS2) = -3564.37704623 au

O	1.59229400	1.15654100	-0.33548500

S	2.99820100	0.85273700	0.16133400
O	4.01579100	1.58287100	-0.60648200
O	3.12268200	0.83202100	1.62748000
C	3.20503900	-0.92530400	-0.34777500
F	4.43297400	-1.35483000	-0.02768500
F	2.30554900	-1.70235500	0.28150800
F	3.03109900	-1.06061700	-1.66895600
Cu	-0.00230200	0.33508300	0.39565100
O	-3.88185300	-1.45252200	1.05914400
S	-2.78057900	-0.91472800	0.24917900
O	-1.62103200	-0.44458400	1.11542400
O	-2.36721600	-1.67096300	-0.94360500
C	-3.42790500	0.69586500	-0.42166500
F	-2.48944200	1.30471900	-1.16835200
F	-4.50312200	0.47536000	-1.18964000
F	-3.77298300	1.51818600	0.57787500

IX

E (BS2) = -2123.43066592 au

H (BS2) = -2123.226943 au

G (BS2) = -2123.302492 au

E (BS3//BS2) = -3568.25385753 au

Cu	-2.95803200	0.33956200	-0.29040900
N	-4.84520600	0.60731400	0.01527000
C	-5.97960900	0.76888500	0.18374200
C	-7.40127100	0.97131000	0.39412600

H	-7.58522200	1.20307600	1.44828900
H	-7.74755700	1.80321400	-0.22797300
H	-7.94433700	0.06068700	0.12059700
S	1.46880600	-1.74325100	-0.39305100
O	1.69681900	-2.81175800	0.57471300
O	1.17195700	-2.00749900	-1.79757900
N	-0.16725300	-1.08114100	0.20909100
Cl	-0.66804500	0.31369900	-0.84985400
C	2.64231000	-0.44527200	-0.21218900
C	2.83202300	0.44892400	-1.27382300
C	3.32473200	-0.31274700	1.00493000
C	3.73190600	1.49542200	-1.10115200
H	2.30688200	0.31814200	-2.21342400
C	4.21794200	0.74269500	1.14741600
H	3.17340600	-1.02517200	1.80791900
C	4.43020300	1.66312100	0.10629300
H	3.89880200	2.19118900	-1.91818900
H	4.76288000	0.85419800	2.08044000
C	5.37538300	2.81974300	0.29407200
H	6.23497100	2.53503000	0.90989200
H	5.73829200	3.20121900	-0.66522500
H	4.86455200	3.64483700	0.80865000
Cl	-0.05697500	-0.53624900	1.87995100

X

E (BS2) = -2952.26757818 au

H (BS2) = -2952.078675 au

G (BS2) = -2952.161818 au

E (BS3//BS2) = -4397.38081591 au

Cu	-1.25429700	0.46676900	-0.40180000
S	1.85811500	1.30603800	0.10705800
O	1.68956800	2.16532100	-1.05980200
O	1.34583900	1.66042800	1.42672300
N	0.68153700	-0.16393400	-0.35011300
Cl	0.87827200	-1.44185800	0.88876000
C	3.46180800	0.60101300	0.18946700
C	3.90971500	0.08538300	1.41423400
C	4.23750700	0.54018600	-0.97732300
C	5.16901300	-0.50031100	1.45824500
H	3.29756900	0.15508900	2.30599900
C	5.49195300	-0.05268000	-0.90008800
H	3.87679000	0.95876100	-1.90985900
C	5.97253800	-0.58721900	0.30821500
H	5.53641800	-0.89598800	2.40048600
H	6.11120500	-0.09989100	-1.79102600
C	7.31560700	-1.26362900	0.36405100
H	8.01176900	-0.83167300	-0.36192400
H	7.75643700	-1.19560500	1.36354000
H	7.20853100	-2.33000700	0.12257600
Cl	1.19840200	-0.82873400	-1.93346900
O	-3.10075400	1.06321500	-0.59634800

S	-4.15939500	0.66671600	0.42562600
O	-3.65855200	0.61805800	1.80908200
O	-5.44103500	1.33867700	0.17654200
C	-4.45027600	-1.11346800	-0.02943100
F	-5.38197900	-1.64816300	0.76964400
F	-4.86076500	-1.21305900	-1.30001000
F	-3.31343200	-1.82041200	0.10775900

CH_3CN

E (BS2) = -132.761905045 au

H (BS2) = -132.711898 au

G (BS2) = -132.740400 au

E (BS3//BS2) = -132.821565623 au

C	-1.18012300	-0.00011600	0.00002300
H	-1.55275300	-0.92800700	0.44615100
H	-1.55330600	0.07672600	-1.02644800
H	-1.55414000	0.84989200	0.57982100
C	0.27794300	0.00068400	0.00012000
N	1.43904000	-0.00028800	-0.00005400

OTf^-

E (BS2) = -961.572928628 au

H (BS2) = -961.537683 au

G (BS2) = -961.578349 au

E (BS3//BS2) = -961.942057871 au

O	-1.23979800	0.83538300	1.17931800
S	-0.91225500	-0.00009700	0.00004000

O	-1.23993700	-1.43910500	0.13393300
O	-1.23995400	0.60363100	-1.31299800
C	0.94542900	-0.00001200	-0.00002800
F	1.43259500	-0.72791700	-1.02395200
F	1.43222100	1.25080100	-0.11855100
F	1.43307600	-0.52262300	1.14222600

$TsNCl_2$

E (BS2) = -1794.60537702 au

H (BS2) = -1794.457745 au

G (BS2) = -1794.511813 au

E (BS3//BS2) = -1794.95161851 au

S	-0.93427800	-0.86390000	0.50334500
O	-1.32571000	-0.57095600	1.88230600
O	-1.30411300	-2.12396200	-0.14042700
N	-1.67007300	0.37318900	-0.65705800
Cl	-3.42964900	0.08986200	-0.72211500
Cl	-1.35356200	2.01980700	-0.05774600
C	0.76778100	-0.47863900	0.22787000
C	1.35756100	-0.86949000	-0.98106300
C	1.47891300	0.20860200	1.21512200
C	2.69583800	-0.55950900	-1.19143000
H	0.78502600	-1.40057300	-1.73401600
C	2.81990900	0.50110400	0.98032400
H	0.99660200	0.50431800	2.14017200
C	3.44603400	0.12558000	-0.21860100

H	3.17013200	-0.85286400	-2.12387100
H	3.38830900	1.03089900	1.73928100
C	4.90133700	0.42838000	-0.46067100
H	5.50253500	-0.48511600	-0.36127500
H	5.06079400	0.80980800	-1.47555200
H	5.28449600	1.16241100	0.25444400

XI

E (BS2) = -2256.2398748 au

H (BS2) = -2255.983788 au

G (BS2) = -2256.071204 au

E (BS3//BS2) = -3701.1145506 au

Cu	-3.05959400	0.26258700	-0.06407200
C	-1.54013800	4.46798300	-0.17677000
H	-0.66685400	4.46576200	0.48388500
H	-2.24021300	5.24553400	0.14459700
H	-1.21759200	4.66759200	-1.20372600
C	-2.18198100	3.16890700	-0.11940800
N	-2.67157000	2.12080300	-0.07478100
C	-3.70482900	-4.19657700	-0.09462400
H	-2.82485900	-4.65415500	0.36954600
H	-3.79760800	-4.55049000	-1.12653600
H	-4.60116900	-4.47630000	0.46843900
C	-3.55528900	-2.75323000	-0.08834800
N	-3.42539700	-1.60260400	-0.08101100
S	0.59550600	0.37272500	-0.95234100

O	0.05349600	-0.56815500	-1.93057400
O	0.37069700	1.81280900	-1.05922300
N	-0.38128000	-0.07747900	0.56048200
Cl	-0.08718700	-1.77757200	0.98061500
Cl	0.07890900	0.96381700	1.92318900
C	2.28769400	0.02890000	-0.59636600
C	3.06502000	1.02614700	0.00647700
C	2.80380200	-1.23920300	-0.89011400
C	4.39000600	0.73538100	0.31361700
H	2.64917100	2.00607100	0.21146000
C	4.13287700	-1.50093000	-0.57371100
H	2.18767100	-1.99193400	-1.36870200
C	4.94137200	-0.52681400	0.03584700
H	5.00864900	1.50149100	0.77224100
H	4.55160200	-2.47590800	-0.80620900
C	6.36789000	-0.83702500	0.40519100
H	6.80004200	-1.58784500	-0.26404100
H	6.99254900	0.06163300	0.37863000
H	6.41511700	-1.23976100	1.42610600

XVI

E (BS2) = -2256.24178791 au

H (BS2) = -2255.986438 au

G (BS2) = -2256.069143 au

E (BS3//BS2) = -3701.11710347 au

Cu	-1.91390900	1.88493100	0.15044000

C	1.81245300	4.03849900	-1.15243500
H	2.14971100	4.74033500	-0.38289600
H	1.69124000	4.56839000	-2.10263600
H	2.55034800	3.23805900	-1.26795200
C	0.54781400	3.45054600	-0.75415300
N	-0.44863800	2.95660900	-0.43041800
C	-5.13884500	-1.02892800	1.34195300
H	-4.67191700	-1.85399000	1.88916000
H	-5.59358300	-1.41327400	0.42349100
H	-5.91090700	-0.56796800	1.96659700
C	-4.13080400	-0.04080200	1.00457000
N	-3.32371300	0.74246000	0.72816300
S	2.52484900	-0.59521600	-0.65753900
O	2.62828400	0.73210700	-1.26336400
O	3.56360900	-1.60654000	-0.83557500
N	2.67532200	-0.19679800	1.13463200
Cl	1.39933300	0.93796300	1.61347000
Cl	2.57065600	-1.67132700	2.11798200
C	0.91682200	-1.27486000	-0.91349800
C	0.69197100	-2.63117300	-0.64011700
C	-0.11198200	-0.42605800	-1.33304100
C	-0.59981000	-3.12777700	-0.77910400
H	1.50607900	-3.28055800	-0.33904700
C	-1.39314300	-0.95210700	-1.47278400
H	0.08191100	0.61666700	-1.55003700

C	-1.66133200	-2.29825500	-1.18237900
H	-0.78941700	-4.17734500	-0.57231500
H	-2.19762100	-0.29812700	-1.79523700
C	-3.06235100	-2.84254800	-1.27346800
H	-3.71462000	-2.18041000	-1.85082000
H	-3.07297700	-3.83639400	-1.73455700
H	-3.49299400	-2.94740000	-0.26895400

MECP1

E (BS2) = -2256.20850213 au

Cu	-2.62007350	0.00093000	-0.35697300
C	-3.60911800	4.44065840	0.13149580
H	-2.76492920	4.91168030	0.64602830
H	-4.50888570	4.52678300	0.74918050
H	-3.77514710	4.93572440	-0.83084120
C	-3.31012410	3.03976100	-0.08986040
N	-3.06056210	1.92422510	-0.26401950
C	-2.54152270	-4.52161720	0.14384520
H	-1.48813530	-4.81098900	0.21409240
H	-3.00813160	-5.03109170	-0.70577080
H	-3.06126120	-4.79663120	1.06753890
C	-2.62159390	-3.08827810	-0.04510460
N	-2.66298520	-1.94210250	-0.18921400
S	0.59726120	-1.08824780	-0.47224810
O	0.20178720	-2.12670590	0.47684610
O	0.42627710	-1.27346430	-1.91742480

N	-0.50411430	0.32398110	-0.07205240
Cl	-0.32790990	0.69967850	2.19739900
Cl	0.05587370	1.74968400	-0.89021840
C	2.23779130	-0.53055140	-0.11258410
C	2.99237640	0.07373020	-1.12328560
C	2.74686070	-0.72413070	1.17763200
C	4.28547960	0.49365430	-0.82436340
H	2.58362600	0.20040700	-2.11949540
C	4.04338920	-0.30154940	1.44732870
H	2.14593630	-1.20155380	1.94214560
C	4.82842510	0.31660240	0.45831790
H	4.88432400	0.96260160	-1.59949940
H	4.45676510	-0.45586530	2.44010470
C	6.22123050	0.78990940	0.78212810
H	6.76133520	0.04689850	1.37929630
H	6.79717810	0.99765510	-0.12505380
H	6.18213540	1.71325760	1.37480250

MECP2

E (BS2) = -2256.19721917 au

Cu	-3.27245860	0.32783810	-0.45645110
C	-4.19067020	-3.94315330	-1.74166210
H	-3.53987530	-4.25319330	-2.56564250
H	-5.23832330	-4.02564780	-2.04915180
H	-4.01193680	-4.58454550	-0.87215860
C	-3.90026100	-2.56808920	-1.38718610

N	-3.66989500	-1.47193670	-1.09885350
C	-2.50224240	4.69309560	0.54740920
H	-1.43551720	4.78827080	0.77699060
H	-3.09321730	5.01713830	1.41007960
H	-2.74804190	5.31443790	-0.31986110
C	-2.80269540	3.30725680	0.24768250
N	-3.03488590	2.19974150	0.00784090
S	1.70743480	0.36250710	2.00054030
O	0.95437440	1.61607550	2.05078560
O	2.51896430	-0.06995630	3.14777830
N	0.41786910	-0.87811640	1.92724680
Cl	-0.95008710	-0.25352690	0.05634600
Cl	1.04570140	-2.43783080	1.57332300
C	2.67284410	0.27277590	0.52016980
C	3.78733550	-0.57235230	0.48635370
C	2.29728130	1.04149740	-0.58827870
C	4.53676100	-0.64070500	-0.68543000
H	4.07124370	-1.14705470	1.36021620
C	3.06442070	0.95767780	-1.74554080
H	1.43549720	1.69701440	-0.53715260
C	4.18814440	0.11577760	-1.81526210
H	5.41017740	-1.28587990	-0.72032100
H	2.79449240	1.56097210	-2.60759790
C	4.99063760	0.01984540	-3.08665430
H	5.08852150	0.99820690	-3.56986350

H	5.99147950	-0.38159770	-2.90023980
H	4.49444950	-0.64902000	-3.80255940

XII

E (BS2) = -2256.21567298 au

H (BS2) = -2255.960546 au

G (BS2) = -2256.044525 au

E (BS3//BS2) = -3701.09103755 au

Cu	2.60468300	0.13119700	-0.06328800
C	3.37384900	-4.37570500	-0.18188900
H	2.52134100	-4.82838100	0.33560600
H	4.29813400	-4.61636800	0.35348400
H	3.43029600	-4.76141300	-1.20484400
C	3.19505100	-2.93846300	-0.21531100
N	3.03606100	-1.79389100	-0.23648500
C	2.13937800	4.65442500	0.27844600
H	1.10963600	4.82159000	0.61204900
H	2.28283100	5.09913500	-0.71178500
H	2.83651800	5.10751300	0.99046700
C	2.37667300	3.22875400	0.20275400
N	2.53910300	2.08625200	0.14176100
S	-0.54562100	1.19773200	-0.04357800
O	-0.20327400	1.72935400	1.27615700
O	-0.37451200	2.01891700	-1.24870100
N	0.54576500	-0.20503800	-0.21476000
Cl	0.25174100	-1.65436000	1.85011600

Cl	0.16086300	-1.14505300	-1.62249500
C	-2.17865000	0.50800900	-0.02839500
C	-2.89937600	0.44366300	-1.22548300
C	-2.72151200	0.07392800	1.18569800
C	-4.19308500	-0.06857000	-1.19549900
H	-2.46512100	0.80078000	-2.15251000
C	-4.01804900	-0.43093300	1.18925700
H	-2.14405400	0.13688300	2.09986600
C	-4.76963200	-0.51541300	0.00495400
H	-4.76759700	-0.11556200	-2.11635600
H	-4.45706700	-0.76141000	2.12653100
C	-6.16022200	-1.09429800	0.01995600
H	-6.67042600	-0.88288200	0.96555000
H	-6.76648400	-0.70185800	-0.80277800
H	-6.11890600	-2.18625700	-0.09240300

$TS_{XII\text{-}XIII}$

E (BS2) = -2256.21045358 au

H (BS2) = -2255.955583 au

G (BS2) = -2256.041121 au

E (BS3//BS2) = -3701.08719094 au

Cu	-2.47111100	0.01307000	-0.03350200
C	-3.53124300	-4.43389300	-0.15225300
H	-2.88597200	-5.00382200	0.52450900
H	-3.38350100	-4.77993100	-1.18053400
H	-4.57874600	-4.57090900	0.13526900

C	-3.18835300	-3.02929400	-0.06699000
N	-2.90262500	-1.91149000	0.00193500
C	-2.32004100	4.54432900	-0.41819500
H	-1.27109600	4.79819600	-0.23064400
H	-2.95888300	5.05388700	0.31020600
H	-2.59703200	4.85190300	-1.43174900
C	-2.47615500	3.11171500	-0.28693200
N	-2.57120400	1.96502600	-0.18227700
S	0.71674500	1.12505800	0.90969600
O	0.30396300	2.24500800	0.06885000
O	0.70668100	1.23769700	2.37138700
N	-0.46435000	-0.17939600	0.54179800
Cl	-0.73252200	-0.28890900	-2.07666700
Cl	0.00303900	-1.67738700	1.22290400
C	2.26005800	0.46765300	0.34936100
C	3.11064700	-0.15458300	1.26788000
C	2.58325400	0.57336200	-1.00976500
C	4.31625600	-0.67679100	0.80663100
H	2.84191500	-0.21861300	2.31639700
C	3.79217900	0.04189900	-1.44358700
H	1.90508000	1.05823500	-1.70191100
C	4.67180300	-0.59381700	-0.54915700
H	4.99182200	-1.15499400	1.51005600
H	4.06111100	0.12029500	-2.49336900
C	5.96136200	-1.18963700	-1.04926000

H	6.46618800	-0.51075500	-1.74572600
H	6.64452100	-1.42035100	-0.22630500
H	5.76358500	-2.12231100	-1.59431100

XIII

E (BS2) = -2256.22529402 au

H (BS2) = -2255.969228 au

G (BS2) = -2256.057273 au

E (BS3//BS2) = -3701.10508137 au

Cu	-2.02456100	1.07614000	-0.24821900
S	0.77415300	-0.52569400	1.86478300
O	0.56023800	0.83770600	2.35591000
O	0.88864500	-1.65960900	2.78480200
N	-0.67001200	-0.74799000	0.83521900
Cl	-1.07616300	0.51956200	-2.31159000
Cl	-0.92397400	-2.34896700	0.37188300
C	2.06236100	-0.56939400	0.65916800
C	2.69224700	-1.78870500	0.37853700
C	2.39767200	0.60818000	-0.02219900
C	3.68156400	-1.81408200	-0.59858200
H	2.42593200	-2.68860000	0.92216100
C	3.39068900	0.55666300	-0.99248200
H	1.90192600	1.54148400	0.21164300
C	4.04301200	-0.64999800	-1.29974200
H	4.18720600	-2.74979400	-0.81923100
H	3.66784300	1.46553100	-1.51905300

C	5.09297700	-0.69455600	-2.37709000
H	5.67463800	0.23300500	-2.40345800
H	5.77707200	-1.53723500	-2.23763600
H	4.61970100	-0.81045400	-3.36166600
C	-4.26954600	-1.06080000	-0.58148600
N	-3.47515300	-0.24046400	-0.40130100
C	-5.25297200	-2.09835300	-0.81422000
H	-4.74004300	-3.05981400	-0.92338300
H	-5.94516300	-2.14431900	0.03270900
H	-5.80729200	-1.87180200	-1.73109000
C	-0.04968600	3.38253500	0.45624100
N	-0.77669100	2.54922600	0.12094100
C	0.88807400	4.39893200	0.88657800
H	1.45342900	4.01825200	1.74378700
H	1.57443900	4.63185200	0.06615500
H	0.34203800	5.30209800	1.17784200

$Cl^{\bullet}$

E (BS2) = -460.135154372 au

H (BS2) = -460.132794 au

G (BS2) = -460.150832 au

E (BS3//BS2) = -460.169293711 au

Cl	0.00000000	0.00000000	0.00000000

Cl^{-}

E (BS2) = -460.357358878 au

H (BS2) = -460.354998 au

G (BS2) = -460.372382 au

E (BS3//BS2) = -460.396980269 au

Cl	0.00000000	0.00000000	0.00000000

XV

E (BS2) = -1796.05045812 au

H (BS2) = -1795.797789 au

G (BS2) = -1795.880331 au

E (BS3//BS2) = -3240.89237495 au

Cu	-2.51563400	-0.21314100	-0.37579000
C	-2.82354900	-4.79112800	0.06139900
H	-1.79872200	-5.16722200	0.14619100
H	-3.31133000	-5.24993200	-0.80460100
H	-3.38098000	-5.04053900	0.97020500
C	-2.79277600	-3.35075300	-0.10584300
N	-2.76209900	-2.20155700	-0.23506800
C	-2.49273700	4.28086700	0.25864900
H	-1.44593900	4.45970900	0.52608300
H	-3.14290000	4.59380200	1.08183400
H	-2.74440000	4.84559500	-0.64489200
C	-2.66366400	2.86444400	0.01070900
N	-2.77233400	1.73048000	-0.18893500
S	0.42308400	1.10509400	-0.25024000
O	0.04436500	1.39521600	-1.64242700
O	0.27688000	2.14584700	0.78012400
N	-0.51825800	-0.31779200	0.08334000

Cl	-0.24697800	-0.88375000	1.72951900
C	2.08428700	0.47154400	-0.20304200
C	2.90099700	0.75644000	0.89350300
C	2.53531500	-0.31363500	-1.27029300
C	4.19841200	0.24858900	0.91082300
H	2.53255500	1.37198300	1.70680200
C	3.83366600	-0.81195100	-1.23064400
H	1.88787000	-0.51937600	-2.11638700
C	4.68236300	-0.54279400	-0.14291600
H	4.84653600	0.47322700	1.75351400
H	4.19872200	-1.41569000	-2.05714800
C	6.07624500	-1.11387900	-0.10150800
H	6.51471400	-1.16756500	-1.10371600
H	6.73494300	-0.51801200	0.53837700
H	6.05910600	-2.13553900	0.30170300

[TsNCl]$^{\bullet}$

E (BS2) = -1334.40696625 au

H (BS2) = -1334.261839 au

G (BS2) = -1334.316592 au

E (BS3//BS2) = -1334.71876378 au

S	1.41659400	-0.79446100	0.30992900
O	1.68546100	-2.15216600	-0.17466400
O	1.95706400	-0.32498300	1.58986100
N	2.01926900	0.18398100	-1.05295900
Cl	2.42935500	1.76893300	-0.60124500

C	-0.30848000	-0.42333700	0.19666900
C	-0.85540700	0.55081700	1.04084200
C	-1.08111300	-1.08125400	-0.76985400
C	-2.20684800	0.85411000	0.91667700
H	-0.23923600	1.04482200	1.78439200
C	-2.42986000	-0.75878400	-0.87468400
H	-0.63803600	-1.83472100	-1.41253200
C	-3.01154800	0.21155300	-0.04087800
H	-2.64834800	1.59810900	1.57381700
H	-3.04377200	-1.26773900	-1.61244800
C	-4.46474500	0.57716300	-0.18815800
H	-5.05283100	-0.26485200	-0.56714300
H	-4.89259700	0.90720400	0.76403500
H	-4.57680200	1.40441600	-0.90237700

XIV

E (BS2) = -921.80344229 au

H (BS2) = -921.695927 au

G (BS2) = -921.74956 au

E (BS3//BS2) = -2366.37588163 au

Cu	0.00736200	-0.27601500	-0.07104600
Cl	-0.03007300	2.03988700	0.01802200
C	-3.10080600	-0.59867000	0.02130800
N	-1.94909200	-0.51541500	-0.03530700
C	-4.54421000	-0.69777300	0.08994900
H	-4.83082100	-1.19064200	1.02542400

H	-4.97672800	0.30716300	0.05454600
H	-4.90712600	-1.28318100	-0.76080600
C	3.11424600	-0.57562600	0.04363300
N	1.96159700	-0.48741000	0.02297400
C	4.55843500	-0.68584400	0.06413000
H	4.94271600	-0.25575600	0.99496900
H	4.84279200	-1.74137700	0.00308700
H	4.97339200	-0.14260600	-0.79105600

I

E (BS2) = -1883.41291543 au

H (BS2) = -1883.267031 au

G (BS2) = -1883.343736 au

E (BS3//BS2) = -3328.33779935 au

Cu	-1.10517000	0.78619500	-0.19506800
Cl	-2.58722400	2.51015300	0.36422700
O	0.14689800	-0.60276700	-1.00838700
S	0.87433500	-1.69672600	-0.23936200
O	0.17981700	-2.12058000	0.98532700
O	1.39849000	-2.73140800	-1.13968500
C	2.37952000	-0.78675400	0.37403100
F	3.25701900	-1.65360200	0.89434000
F	2.97334100	-0.12002900	-0.62693500
F	2.03304800	0.09449700	1.32600100
N	-2.53531300	-0.59371100	-0.07630300
C	-3.32211600	-1.43390200	0.02444800

C	-4.30719000	-2.49066800	0.15371600
H	-4.85041700	-2.36812100	1.09636400
H	-5.01155000	-2.43709600	-0.68276800
H	-3.80163100	-3.46165000	0.14524900
N	0.43431500	2.06193700	-0.26682200
C	1.42794500	2.65108800	-0.24139000
C	2.69131900	3.36153600	-0.19977700
H	3.50527400	2.62895200	-0.20291000
H	2.77754100	4.01236800	-1.07566900
H	2.74196900	3.96576800	0.71162900

I^{i}

E (BS2) = -1054.58538619 au

H (BS2) = -1054.424328 au

G (BS2) = -1054.489579 au

E (BS3//BS2) = -2499.21289743 au

Cu	-0.02265200	-0.36532100	-0.11748400
Cl	-0.07932600	-2.69034800	-0.24309900
N	1.97560300	-0.37397600	0.07237300
C	3.12371200	-0.39016900	0.20550500
C	4.56408500	-0.41483600	0.37428700
H	4.81223700	-0.96260300	1.28939900
H	5.02162200	-0.91493400	-0.48550400
H	4.94160300	0.61009300	0.44639900
N	-2.01781400	-0.27610000	0.06733500
C	-3.16475300	-0.22581100	0.20208100

C	-4.60397300	-0.16786100	0.37279800
H	-4.92142900	0.87682200	0.45124600
H	-5.09064900	-0.63546400	-0.48925600
H	-4.88243600	-0.70555300	1.28513000
N	0.04349400	1.74358600	-0.12171700
C	0.11325600	2.89807900	-0.12817700
C	0.20556200	4.34662100	-0.13709700
H	1.10278700	4.65749400	0.40793900
H	0.26613900	4.70052700	-1.17132700
H	-0.68072100	4.77314400	0.34338600

I[ii]

E (BS2) = -2712.23081441 au

H (BS2) = -2712.099924 au

G (BS2) = -2712.185576 au

E (BS3//BS2) = -4157.45441038 au

Cu	-0.09852200	-0.28626300	-0.21788600
Cl	-0.43843800	-2.53949600	-0.47375900
C	0.91705400	2.71950900	-0.16561300
N	0.41510900	1.68928800	-0.31224000
C	1.55815800	4.00435300	0.03409500
H	1.59663400	4.22139600	1.10652400
H	2.57697900	3.96440700	-0.36321100
H	0.99243900	4.78892700	-0.47774200
O	3.76152100	-1.01623700	1.86968800
S	2.92766000	-0.15285200	1.02256000

O	1.48315800	-0.62822800	1.00409500
O	3.09457200	1.30198100	1.16705800
C	3.48064000	-0.51881400	-0.71645100
F	2.67455000	0.08516400	-1.60865400
F	4.72829000	-0.06883900	-0.89974600
F	3.45664100	-1.83637700	-0.94930300
O	-4.03567600	1.25304400	-1.36078200
S	-3.00439200	0.84115800	-0.39969300
O	-1.84296400	0.14837800	-1.10337100
O	-2.59594500	1.79946200	0.63779200
C	-3.75400100	-0.56747300	0.55209900
F	-2.88022900	-1.01424600	1.46765700
F	-4.85949500	-0.14962900	1.18565800
F	-4.08219700	-1.57520900	-0.26381100

I^{iii}

E (BS2) = -1750.62494996 au

H (BS2) = -1750.531511 au

G (BS2) = -1750.595776 au

E (BS3//BS2) = -3195.49029558 au

Cu	-0.94506900	0.52980000	-0.23374800
Cl	-2.05146700	2.33113800	0.62833500
O	0.96062700	1.36834200	-0.28772300
S	1.60667600	0.08747100	-0.78771200
O	0.43839200	-0.83064600	-1.07645800
O	2.66211000	0.20207800	-1.78327900

C	2.37189000	-0.64641700	0.74559600
F	2.83281500	-1.86564800	0.47053800
F	3.37083400	0.12974800	1.16366100
F	1.43674100	-0.72589600	1.69771100
C	-3.37197400	-1.48043200	-0.05528900
N	-2.47022800	-0.76510300	-0.16861300
C	-4.50379300	-2.37430900	0.09105500
H	-5.11968600	-2.33215700	-0.81314400
H	-4.14315600	-3.39683900	0.24268900
H	-5.09971400	-2.06342600	0.95545500

TS'$_{1s\text{-}XVIIs}$$^{\gamma}$

E (BS2) = -1837.26180194 au

H (BS2) = -1836.883507 au

G (BS2) = -1836.967815 au

E (BS3//BS2) = -1837.77253759 au

S	-2.95969200	-0.83534200	-1.10043200
O	-2.02388900	-1.21672000	-2.15202600
O	-4.39791600	-0.93908100	-1.31140700
N	-2.55500900	-1.96489500	0.14545400
Cl	-3.37481200	-1.61032300	1.63143400
C	-2.57942100	0.79130100	-0.52813500
C	-3.58333900	1.57508500	0.03729500
C	-1.26448900	1.24739600	-0.62244000
C	-3.25733200	2.84047800	0.50826700
H	-4.60409200	1.20323100	0.09907900

C	-0.96067200	2.51136000	-0.13952400
H	-0.47942100	0.63047800	-1.05753000
C	-1.94839900	3.32611500	0.42717600
H	-4.03302800	3.46556600	0.94975700
H	0.06493400	2.87513400	-0.20477700
C	-1.60816100	4.69891900	0.91477300
H	-1.56541300	5.40883100	0.07621900
H	-2.35610400	5.07262900	1.62397800
H	-0.62450000	4.71846300	1.40058500
C	2.65289900	-0.41797300	-0.40194600
O	1.82290500	0.44204000	-0.66423200
C	2.26874500	-1.87427200	-0.29857700
H	2.81360000	-2.41895900	-1.08636300
H	2.64962500	-2.28161800	0.65051000
C	0.76820500	-2.12506400	-0.43995400
H	0.60315000	-3.19987000	-0.60547600
H	0.40238200	-1.59863800	-1.33249200
C	-0.00721300	-1.69312100	0.77809100
H	-1.24421400	-1.78698400	0.41237700
C	4.08105300	-0.04531000	-0.19482000
C	4.42448700	1.31084300	-0.24965200
C	5.08259100	-0.99412100	0.04193900
C	5.73981100	1.71359500	-0.06799200
H	3.63846100	2.04015300	-0.43649300
C	6.40151600	-0.59049600	0.22011600

H	4.84131700	-2.05476600	0.08483200
C	6.73115000	0.76150200	0.16688900
H	5.99652500	2.77081400	-0.10972800
H	7.17509200	-1.33466300	0.40116500
H	7.76433500	1.07500400	0.30866600
C	0.06055400	-2.58512900	1.98141000
H	1.09142700	-2.62599100	2.37181800
H	-0.57656100	-2.22641800	2.79950200
H	-0.23291100	-3.61448700	1.72986500
H	0.06664500	-0.61925100	1.00270000

I^iv^

E (BS2) = -2579.44749985 au

H (BS2) = -2579.368925 au

G (BS2) = -2579.443020 au

E (BS3//BS2) = -4024.61052259 au

Cu	-0.19773700	1.03999100	0.07503300
Cl	-0.06912400	3.29918300	-0.14757200
O	-2.12442700	0.99751800	0.90241600
S	-2.24426700	-0.51125600	0.80282200
O	-0.87808500	-0.97557900	0.35804900
O	-2.88219000	-1.21184900	1.91025500
C	-3.31651600	-0.77157300	-0.69650800
F	-3.38383800	-2.07315700	-0.97656500
F	-4.54031200	-0.29971700	-0.45952700
F	-2.77862700	-0.11967200	-1.73216200

O	3.95551000	0.75367300	-0.87303300
S	2.81154300	0.32687300	-0.06012800
O	2.77417700	0.71044200	1.35879900
O	1.48491700	0.55791400	-0.79154700
C	2.87568800	-1.53121100	-0.05682800
F	1.86753100	-2.02470200	0.67097700
F	2.78208900	-2.00251000	-1.30591500
F	4.03524800	-1.93979400	0.47543100

$TS^{S}_{XII\text{-}XIII}$

E (BS2) = -2256.19933108 au

H (BS2) = -2255.944680 au

G (BS2) = -2256.030831 au

E (BS3//BS2) = -3701.07386454 au

Cu	1.96339500	-0.25904600	-0.23166400
S	-0.97009000	0.12255400	1.43305800
O	-0.41218600	-1.15167800	1.87120700
O	-0.93427400	1.31632200	2.28029200
N	0.25519600	0.66831000	0.16276900
Cl	0.03168500	-1.11284000	-1.35925100
Cl	-0.21006600	2.03788300	-0.71658300
C	-2.54271900	-0.06611400	0.67296100
C	-3.35162800	1.06466800	0.50470200
C	-2.94119100	-1.33997000	0.24264000
C	-4.58749900	0.90415300	-0.11518600
H	-3.03344700	2.03856700	0.85908500

C	-4.18178600	-1.46901800	-0.36695900
H	-2.30062600	-2.20100100	0.39169300
C	-5.01909600	-0.35499400	-0.56073300
H	-5.22786500	1.77056200	-0.25129200
H	-4.51010100	-2.44945600	-0.70031000
C	-6.35948200	-0.52859800	-1.22336700
H	-7.01665600	-1.15018900	-0.60186000
H	-6.85517200	0.43177200	-1.39133500
H	-6.25247200	-1.04015400	-2.18735900
C	4.03881200	-2.62367200	0.04132800
N	3.28237100	-1.75223800	-0.04549100
C	4.98587100	-3.71745300	0.15085700
H	5.22560300	-3.88739200	1.20555100
H	4.54502800	-4.62473500	-0.27462100
H	5.90049900	-3.46467700	-0.39533600
C	3.80339200	2.46924600	-0.35031900
N	3.18254100	1.49188300	-0.35453000
C	4.57976800	3.69686800	-0.34526500
H	4.76538000	4.00739500	0.68806900
H	5.53535200	3.52851500	-0.85221900
H	4.02421900	4.48299100	-0.86673400

XIII^{S}

E (BS2) = -2256.20672355 au

H (BS2) = -2255.951050 au

G (BS2) = -2256.036523 au

E (BS3//BS2) = -3701.08673742 au

Cu	1.75677600	0.51983000	0.18767900
S	-0.86024300	-1.10450100	-0.65472600
O	-0.22474400	-0.73084200	-1.91985700
O	-0.85306600	-2.48500300	-0.17082500
N	-0.16151400	0.06338900	0.53444700
Cl	1.19671500	2.70256600	0.57031900
Cl	-0.31910100	-0.49481100	2.15345400
C	-2.46532500	-0.38578600	-0.51139800
C	-3.42465700	-1.00273500	0.30711500
C	-2.71451900	0.83301500	-1.17032900
C	-4.66449800	-0.39744600	0.43257700
H	-3.20323400	-1.93617000	0.81284700
C	-3.96382900	1.41746400	-1.02642200
H	-1.94725000	1.29858800	-1.77933300
C	-4.95464100	0.81973000	-0.22522400
H	-5.42892000	-0.86533600	1.04596800
H	-4.17824200	2.35326400	-1.53349900
C	-6.30914100	1.44709000	-0.07615500
H	-6.31167700	2.48965900	-0.40605200
H	-7.04341200	0.89792800	-0.68244000
H	-6.65364300	1.39749600	0.96297600
C	2.36781700	-2.55363700	-0.05885600
N	2.15018300	-1.42063700	0.01256300
C	2.62521700	-3.97528200	-0.15219000

H	1.66896900	-4.50670900	-0.19779300
H	3.20580600	-4.18176700	-1.05719400
H	3.18683600	-4.30148900	0.72931800
C	4.75464700	1.31609300	-0.45872700
N	3.65942400	1.03788600	-0.21480400
C	6.12796300	1.66715900	-0.76611400
H	6.17175400	2.11439700	-1.76455500
H	6.49304500	2.38708000	-0.02647700
H	6.74954600	0.76645400	-0.74064000

5a

E (BS2) = -271.577303489 au

H (BS2) = -271.441922 au

G (BS2) = -271.480150 au

E (BS3//BS2) = -271.684885569 au

C	-2.42608200	-0.00005000	0.00923100
H	-2.83473100	0.88573000	-0.48980200
H	-2.83471500	-0.89039800	-0.48156000
C	-0.91544700	-0.00010800	-0.01247400
C	-0.19529600	-1.20442300	-0.00938200
C	-0.19544500	1.20433400	-0.00938600
C	1.20213600	-1.20714500	0.00227900
H	-0.73642400	-2.14831000	-0.01779900
C	1.20195200	1.20724700	0.00228100
H	-0.73671000	2.14814800	-0.01780900
C	1.90706500	0.00008600	0.00862800

H	1.73995600	-2.15212300	0.00223900
H	1.73965000	2.15229400	0.00224600
H	2.99403400	0.00016700	0.01456800
H	-2.80435900	0.00484800	1.04085900

TS$_{5a\text{-}XXVa}$

E (BS2) = -1605.98125938 au

H (BS2) = -1605.704499 au

G (BS2) = -1605.773008 au

E (BS3//BS2) = -1606.39856930 au

S	-1.88556500	1.61954800	-0.07051400
O	-2.57839900	2.07749800	1.14114100
O	-2.37295100	1.99720400	-1.40441000
N	-0.30109700	2.32529000	-0.07501200
Cl	0.59473300	2.00316000	1.40238700
C	-1.72374100	-0.15379200	0.00336600
C	-1.83694500	-0.89631400	-1.17466500
C	-1.46774200	-0.77436800	1.23198800
C	-1.68610900	-2.28147300	-1.11596800
H	-2.04365600	-0.40038000	-2.11658700
C	-1.30425300	-2.15607700	1.26499600
H	-1.40481800	-0.19077600	2.14330400
C	-1.40989200	-2.93024400	0.09695900
H	-1.77850900	-2.86543800	-2.02752500
H	-1.09616800	-2.64490100	2.21313300
C	-1.24379100	-4.42713600	0.16069400

H	-2.10896000	-4.89208500	0.65130300
H	-1.14832100	-4.86406000	-0.83803400
H	-0.35752000	-4.69932700	0.74558700
C	1.17746300	1.02155700	-1.86822300
H	0.42313800	1.68584500	-1.03320800
H	1.57231800	1.84509800	-2.46734600
H	0.42955400	0.41815700	-2.38578400
C	2.13440500	0.30145100	-1.04787900
C	1.82048400	-0.97834600	-0.52607700
C	3.37200100	0.89108600	-0.68860300
C	2.71397700	-1.64416300	0.30387700
H	0.87084500	-1.43436100	-0.78485800
C	4.26404100	0.21855300	0.13825500
H	3.61659200	1.87818600	-1.07229700
C	3.93905200	-1.05033800	0.63939300
H	2.45937600	-2.62586000	0.69401400
H	5.21334700	0.67823100	0.39899800
H	4.63612800	-1.57183000	1.28949900

XXVa

E (BS2) = -270.924713136 au

H (BS2) = -270.803108 au

G (BS2) = -270.839357 au

E (BS3//BS2) = -271.032655168 au

C	-2.40377600	0.00000800	-0.00029700
C	-0.99561200	-0.00001300	-0.00011900

C	-0.25301300	-1.22045300	0.00007800
C	-0.25301500	1.22044700	0.00001900
C	1.13486500	-1.21363500	-0.00002600
H	-0.79537200	-2.16303700	0.00017900
C	1.13486500	1.21363900	0.00010100
H	-0.79536000	2.16302400	-0.00011200
C	1.84150700	0.00000600	-0.00003100
H	1.67819900	-2.15531500	-0.00014800
H	1.67815200	2.15534800	0.00013600
H	2.92799200	-0.00001300	-0.00003600
H	-2.96429000	0.93048900	0.00056000
H	-2.96424300	-0.93050000	0.00107200

TsNHCl

E (BS2) = -1335.06205300 au

H (BS2) = -1334.903731 au

G (BS2) = -1334.956434 au

E (BS3//BS2) = -1335.37830709 au

S	1.48301200	-0.80195800	0.27895300
O	1.76861800	-2.10703700	-0.32698000
O	1.97564900	-0.45063000	1.61010200
N	2.28326900	0.27045800	-0.83345000
Cl	1.98896500	1.98241600	-0.52457800
C	-0.25856900	-0.46082100	0.18307700
C	-0.84691500	0.37645800	1.13369800
C	-0.99933300	-1.00915100	-0.87075900

C	-2.20736600	0.66092200	1.02427500
H	-0.25763500	0.78483900	1.94729000
C	-2.35486000	-0.71032500	-0.95994200
H	-0.52845300	-1.66498500	-1.59605800
C	-2.97826400	0.12909000	-0.01991800
H	-2.67671400	1.30427600	1.76337000
H	-2.94228200	-1.13758100	-1.76824300
C	-4.44273500	0.46042800	-0.15042200
H	-5.02610600	-0.42508900	-0.42655200
H	-4.84720900	0.86902300	0.78073800
H	-4.59754600	1.20853900	-0.93938000
H	1.92657700	0.07975200	-1.77746700

XXVIa

E (BS2) = -2154.35528497 au

H (BS2) = -2154.085601 au

G (BS2) = -2154.177933 au

E (BS3//BS2) = -3599.38401392 au

Cu	0.11189700	0.78076800	0.68087700
Cl	1.03734500	2.46177300	2.01976200
C	2.22462900	-1.36671900	1.58632600
N	1.46925500	-0.54950700	1.27733100
C	3.16476500	-2.40084400	1.97600400
H	3.26321600	-3.12482100	1.16146900
H	4.13972200	-1.94929500	2.18302200
H	2.79587500	-2.90673600	2.87430400

C	-2.24445600	2.56927100	-0.42305800
N	-1.34002600	2.01180900	0.03103100
C	-3.39486500	3.23847900	-0.99956300
H	-3.81080100	3.94830200	-0.27772500
H	-3.09153000	3.77264400	-1.90585000
H	-4.14796800	2.48502000	-1.25241900
C	1.44980400	1.76456400	-2.05031900
O	-0.67995600	-0.62865400	-0.55999000
S	-1.58763700	-1.76802300	-0.12404800
O	-1.37648500	-2.19713700	1.26691500
O	-1.69686400	-2.79985500	-1.16334800
C	-3.25060400	-0.93005800	-0.10088400
F	-3.30913000	-0.04554200	0.90788700
F	-4.22039900	-1.83719700	0.07031000
F	-3.47001800	-0.27688900	-1.25215600
H	0.58208600	1.45567800	-2.62464700
H	1.51974500	2.80670800	-1.75367200
C	2.45181400	0.83998900	-1.69460300
C	3.58908600	1.23942500	-0.92750300
C	2.36133600	-0.53448300	-2.07271900
C	4.56562800	0.32259500	-0.56679400
H	3.67365100	2.27906500	-0.62103700
C	3.34674000	-1.44060400	-1.70950600
H	1.49560600	-0.86366600	-2.64035500
C	4.45608200	-1.02310900	-0.95497700

H	5.42022800	0.64771500	0.02126400
H	3.25690300	-2.48262900	-2.00603800
H	5.22358600	-1.73736400	-0.67087800

MECP3a

E (BS2) = -2423.75174132 au

29	0.0106719	1.4533540	0.6486312
17	-1.4683449	3.0626448	1.4945182
6	2.1018570	3.6594354	-0.4327064
7	1.3125910	2.8782360	-0.1082202
6	3.0962971	4.6384223	-0.8357107
1	3.8846352	4.1434293	-1.4114205
1	2.6228875	5.4101306	-1.4510297
1	3.5318789	5.1009465	0.0559624
6	-1.0438410	-1.0600923	2.3213069
7	-0.7170580	-0.0671379	1.8273698
6	-1.4340262	-2.3218656	2.9240400
1	-1.9234472	-2.1421991	3.8860703
1	-2.1260035	-2.8405209	2.2539295
1	-0.5434834	-2.9393709	3.0763935
6	-1.5612913	1.1352862	-1.7412704
1	-0.5954198	0.9704937	-2.2079176
8	1.0757330	0.0395281	-0.4541683
16	2.2343316	-0.7909732	0.0580235
8	2.4327321	-0.7067110	1.5140186
8	3.4271995	-0.6989577	-0.7987537

6	1.5961638	-2.5155636	-0.2311864
9	0.4918584	-2.7583813	0.4937401
9	2.5323709	-3.4105456	0.1129672
9	1.2916807	-2.6901471	-1.5281539
1	-1.9556074	2.1464579	-1.7281949
6	-2.3003521	0.0637845	-1.2228681
6	-3.5742353	0.2785257	-0.6094509
6	-1.7826727	-1.2678302	-1.2579634
6	-4.2793540	-0.7791911	-0.0622633
1	-3.9723958	1.2884105	-0.5723572
6	-2.4990537	-2.3163520	-0.7062647
1	-0.8093608	-1.4370289	-1.7028426
6	-3.7476377	-2.0806511	-0.1058430
1	-5.2446926	-0.6056943	0.4044755
1	-2.0883559	-3.3216157	-0.7264332
1	-4.3049562	-2.9061973	0.3275438

XXVIIa

E (BS2) = -2154.37415812 au

H (BS2) = -2154.102411 au

G (BS2) = -2154.192671 au

E (BS3//BS2) = -3599.39152009 au

Cu	0.73485900	-1.54244600	-0.42260000
Cl	2.22539300	-3.22474800	-0.79335000
C	-1.90002800	-2.89492900	0.98644200
N	-0.84050900	-2.74588600	0.54178700

C	-3.24160100	-3.03089200	1.52703800
H	-3.94408100	-2.53661800	0.84846600
H	-3.50596300	-4.08828300	1.62429900
H	-3.28990400	-2.54951600	2.50856300
C	0.66637000	0.31103000	2.28387200
N	1.11475500	-0.60878700	1.73657900
C	0.10573700	1.47716500	2.94738700
H	-0.55375400	1.16026800	3.76109900
H	0.91069400	2.09925600	3.35059500
H	-0.47463500	2.05078100	2.22077000
C	2.06447900	-0.26702800	-1.77939600
H	1.22978400	-0.14696500	-2.46661700
O	-0.65082600	-0.07477000	-0.85586600
S	-2.03663500	0.20075700	-0.31480600
O	-2.14497500	0.16591500	1.15406000
O	-3.11136000	-0.45753800	-1.07649700
C	-2.18417400	2.00953700	-0.73272300
F	-1.34652700	2.73881600	0.02810900
F	-3.43427500	2.43209100	-0.50857900
F	-1.87766500	2.22844500	-2.02012700
H	2.80959800	-1.00947000	-2.03707300
C	2.39927800	0.77272700	-0.88661900
C	3.57314600	0.67599000	-0.08371600
C	1.56445400	1.92055900	-0.76477400
C	3.89164500	1.68775000	0.80442600

H	4.19700200	-0.20850700	-0.17026700
C	1.89465800	2.92713500	0.12535700
H	0.67703900	1.98781500	-1.37967900
C	3.05114500	2.80866300	0.91325800
H	4.78210100	1.61647000	1.42083300
H	1.25809900	3.80071500	0.22044100
H	3.30069500	3.59701400	1.61775000

$TS_{XXVIIa\text{-}I}$

E (BS2) = -2154.37004621 au

H (BS2) = -2154.099491au

G (BS2) = -2154.191014 au

E (BS3//BS2) = -3599.38683072 au

Cu	-0.21942600	1.89025100	0.15559200
Cl	-0.91347000	3.43834900	-1.39562100
C	2.81453800	2.19556700	1.13388000
N	1.72128800	2.54077000	0.95214900
C	4.17505100	1.72136400	1.32720700
H	4.87366600	2.56383000	1.32240000
H	4.24793600	1.19161200	2.28190700
H	4.41691500	1.03035700	0.51330400
C	-0.75497700	-0.24563800	2.28276300
N	-0.88423700	0.76444600	1.72699900
C	-0.58259900	-1.53036400	2.93586100
H	-0.01106100	-1.40532800	3.86092600
H	-1.55976100	-1.96694300	3.16552900

H	-0.03327300	-2.18898900	2.25728200
C	-2.40144700	0.92764600	-1.82891700
H	-1.55958400	0.74462500	-2.48742100
O	0.70479300	0.05644900	-0.88205700
S	1.88892800	-0.71370700	-0.38826100
O	2.00748800	-0.80836300	1.08215200
O	3.14336300	-0.43333700	-1.11819900
C	1.45249400	-2.45601700	-0.88674200
F	0.46829100	-2.94104500	-0.10073900
F	2.51414200	-3.26522200	-0.76737600
F	1.02203700	-2.50091400	-2.15954600
H	-3.00205000	1.80936500	-2.01265300
C	-2.78951700	-0.01683000	-0.88725100
C	-3.91648900	0.22926700	-0.04149800
C	-2.09397200	-1.26240400	-0.79191900
C	-4.32911000	-0.73767900	0.85362900
H	-4.43102000	1.18264900	-0.11565900
C	-2.53309900	-2.22837200	0.09289500
H	-1.23399800	-1.42877500	-1.42740100
C	-3.64206100	-1.96424100	0.91438400
H	-5.18041200	-0.56136600	1.50274600
H	-2.01884000	-3.17978000	0.16445400
H	-3.97662800	-2.72484200	1.61416400

6a

E (BS2) = -731.174762382 au

H (BS2) = -731.046680 au

G (BS2) = -731.087032 au

E (BS3//BS2) = -731.315189865 au

C	0.02092500	0.00000000	0.46061900
C	-0.65607100	-1.21010100	0.25048800
C	-0.65607100	1.21010100	0.25048900
C	-1.98991000	-1.21055800	-0.16112300
H	-0.13427100	-2.15038300	0.41203800
C	-1.98990900	1.21055900	-0.16112300
H	-0.13427000	2.15038200	0.41203800
C	-2.65871900	0.00000000	-0.36863600
H	-2.50737700	-2.15325800	-0.31776600
H	-2.50737700	2.15325800	-0.31776500
H	-3.69754300	0.00000100	-0.68783300
C	1.45099800	-0.00000100	0.89960400
H	1.71624900	0.89340700	1.46550800
H	1.71624800	-0.89341000	1.46550700
Cl	2.61299300	0.00000000	-0.55609600

3a

E (BS2) = -540.949435172 au

H (BS2) = -540.706592 au

G (BS2) = -540.761054 au

E (BS3//BS2) = -541.165641244 au

C	0.20829600	-0.74959000	0.13733700
O	0.04526700	-1.94645800	0.38930700

C	-0.94676300	0.15374800	-0.08879900
C	-2.20934600	-0.27736500	0.06828500
C	-3.44762100	0.54147200	-0.14951500
H	-3.15494400	1.55322900	-0.45984700
C	-4.30446800	-0.08742500	-1.26549200
H	-4.60868200	-1.10684400	-0.99587300
H	-5.21282000	0.50524000	-1.42784600
H	-3.75278600	-0.13611800	-2.21142600
C	1.59983900	-0.19371400	0.03913500
C	2.66758300	-1.10645400	-0.03336600
C	1.88396500	1.18286200	0.03894100
C	3.98303700	-0.65837700	-0.11751300
H	2.44374000	-2.16837600	-0.02578100
C	3.20425500	1.63172800	-0.03397900
H	1.08676900	1.91447000	0.11531400
C	4.25499400	0.71475800	-0.11757800
H	4.79729100	-1.37519000	-0.18198400
H	3.41088900	2.69842900	-0.02504100
H	5.28119300	1.06690300	-0.18071200
H	-0.76332800	1.17636400	-0.40375700
H	-2.36538700	-1.31149800	0.38015600
C	-4.25169400	0.64138700	1.16114500
H	-4.55522500	-0.35425700	1.50908800
H	-3.66200300	1.11391600	1.95520200
H	-5.15931700	1.23721400	1.00645300

$TS_{3a\text{-}XXIa}$

E (BS2) = -1875.34979867 au

H (BS2) = -1874.966190 au

G (BS2) = -1875.052335 au

E (BS3//BS2) = -1875.87463612 au

S	-1.00944100	-1.59853300	-0.65038300
O	-0.19974600	-1.10216800	-1.77359400
O	-1.32359200	-3.02872800	-0.53227300
N	-0.01644400	-1.16510900	0.70967600
Cl	-0.77238600	-1.57153600	2.24172400
C	-2.52138100	-0.66501700	-0.57813000
C	-3.57540500	-1.11843100	0.22225100
C	-2.61763100	0.52489600	-1.30563700
C	-4.73518100	-0.35168200	0.29981900
H	-3.49421300	-2.05334400	0.76523800
C	-3.79132300	1.27051800	-1.22114500
H	-1.79145200	0.85755100	-1.92361500
C	-4.86226400	0.84965400	-0.41662700
H	-5.55753200	-0.69258200	0.92306700
H	-3.87613900	2.19474400	-1.78630400
C	-6.13668900	1.65137600	-0.34877500
H	-6.85393100	1.29449700	-1.10030700
H	-6.61644100	1.55548200	0.63098300
H	-5.95344500	2.71232600	-0.54747600
C	3.18788000	1.97988600	-0.47362700

O	3.88954100	2.94000400	-0.16612200
C	1.79301400	2.25738200	-0.96144800
C	0.60565400	2.07475000	-0.33916700
C	0.30848000	1.49557400	0.98278400
H	0.11245900	0.28879600	0.71278200
C	3.70395200	0.58443100	-0.45185300
C	5.00835800	0.34859900	0.01792900
C	2.91397100	-0.49383500	-0.87966300
C	5.51024700	-0.94849300	0.06392500
H	5.60870200	1.19112700	0.34688300
C	3.41914800	-1.79346500	-0.82955100
H	1.90720900	-0.32548500	-1.24222800
C	4.71470300	-2.02236200	-0.35856400
H	6.51726500	-1.12881500	0.42996900
H	2.79917900	-2.62379200	-1.15594800
H	5.10715100	-3.03504300	-0.31836700
C	1.42127100	1.40291400	2.02141000
H	1.79834100	2.40732200	2.25472400
H	1.03571400	0.96328200	2.94654200
H	2.26008400	0.79089200	1.68447300
C	-1.01541500	1.97082800	1.57319100
H	-1.81413900	1.99143700	0.82692100
H	-1.32798400	1.33024900	2.40402900
H	-0.89134400	2.98898200	1.96899800
H	1.78403000	2.76415000	-1.92615800

H	-0.27475200	2.39860900	-0.89192700

XXIa

E (BS2) = -540.320647287 au

H (BS2) = -540.091167 au

G (BS2) = -540.146929 au

E (BS3//BS2) = -540.536645292 au

C	-0.15620300	-0.82985100	0.00012300
O	-0.03302300	-2.06928700	0.00032400
C	1.00912300	0.04112300	-0.00003100
C	2.30412400	-0.48917800	0.00004400
C	3.49386900	0.22171400	-0.00001000
C	4.80821100	-0.50276000	-0.00013600
H	4.68613200	-1.59043300	-0.00068100
H	5.40577700	-0.21861700	-0.87885000
H	5.40545100	-0.21947200	0.87907800
C	-1.53581400	-0.22705000	0.00002400
C	-2.63198600	-1.10839200	-0.00019100
C	-1.78755200	1.15649900	0.00019600
C	-3.93882300	-0.62661900	-0.00025800
H	-2.43605000	-2.17565400	-0.00029800
C	-3.09746200	1.63988200	0.00014200
H	-0.97204600	1.87166400	0.00040400
C	-4.17657500	0.75225000	-0.00009200
H	-4.77292500	-1.32348700	-0.00043300
H	-3.27381100	2.71220300	0.00029000

H	-5.19511000	1.13132200	-0.00013300
C	3.58587700	1.71942500	-0.00006000
H	4.14934500	2.06160200	-0.87987700
H	2.61663400	2.22307200	0.00126400
H	4.15167300	2.06153300	0.87827600
H	2.37143800	-1.57585300	0.00015000
H	0.86693800	1.11416100	-0.00028500

XXIIa

E (BS2) = -2423.76082957 au

H (BS2) = -2423.384781 au

G (BS2) = -2423.487207 au

E (BS3//BS2) = -3868.89438183 au

Cu	-0.67925300	-0.97846900	-1.12170500
Cl	-0.82551100	-3.00741300	-2.28856000
C	2.30527200	-0.60896300	-2.03875500
N	1.19593500	-0.71473800	-1.73272000
C	3.70329100	-0.48534800	-2.40189400
H	4.11731200	0.41619000	-1.93974500
H	4.24481300	-1.36325000	-2.03582200
H	3.79595300	-0.42270100	-3.49079100
C	-3.71531700	-1.11069400	-0.30253300
N	-2.60830500	-1.10889000	-0.63223500
C	-5.09683000	-1.09710600	0.13749400
H	-5.64311900	-1.92278600	-0.32874100
H	-5.12574200	-1.20682900	1.22651700

H	-5.55561900	-0.14398900	-0.14473600
C	2.42716300	-1.32633500	1.30194100
O	2.83014100	-2.48848400	1.10798700
C	1.08516500	-1.04690800	1.78449600
H	0.82735100	-0.02500700	2.02697100
C	0.12459100	-2.05622800	1.91468600
H	0.43519500	-3.05959600	1.62825500
C	-1.18003900	-1.88659800	2.35418700
C	-2.12047000	-3.05302000	2.40247800
H	-2.43555300	-3.24740200	3.43850400
H	-3.04007600	-2.83383000	1.84167900
H	-1.67531600	-3.96669700	1.99632100
C	3.34545000	-0.16801600	1.03504600
C	4.73017500	-0.40111200	1.00534000
C	2.86372400	1.11786500	0.73656100
C	5.61689900	0.63030100	0.70110200
H	5.09532000	-1.40098200	1.21899900
C	3.75104200	2.14536300	0.41205400
H	1.79892300	1.31469000	0.70442300
C	5.12889700	1.90732900	0.39891300
H	6.68688700	0.44000500	0.69073000
H	3.36070300	3.12823900	0.16208600
H	5.81884700	2.70960000	0.15074900
O	-0.55943900	0.74816900	-0.02152400
S	-0.78169500	2.15634700	-0.55037200

O	-1.08087700	2.20911400	-1.99070200
O	0.20199000	3.10911900	-0.01509700
C	-2.37996000	2.61268600	0.28751000
F	-3.37388800	1.80939700	-0.12090200
F	-2.70144300	3.87722700	-0.01425500
F	-2.26024100	2.50207200	1.61778700
C	-1.74869200	-0.56179700	2.77636600
H	-2.50325100	-0.69852900	3.56014000
H	-0.98957400	0.13388000	3.14387000
H	-2.24386000	-0.07565200	1.92724500

MECP4a

E (BS2) = -2155.5786404700 au

29	-0.6334861	1.1725834	1.0939141
17	-0.6685655	3.4202863	2.0071611
6	2.3061290	0.5788048	2.0881171
7	1.2086109	0.7383267	1.7565437
6	3.6930117	0.3866119	2.4741163
1	4.0794934	-0.5212014	2.0006112
1	4.2820703	1.2462705	2.1394243
1	3.7660970	0.2957639	3.5621692
6	-3.7142632	1.0878408	0.2923700
7	-2.6060000	1.1532932	0.6185336
6	-5.0957829	0.9901142	-0.1452889
1	-5.6990254	1.7581138	0.3489811
1	-5.1435744	1.1333229	-1.2298727

1	-5.4878446	-0.0005317	0.1055183
6	2.4351640	1.3461221	-1.2815933
8	2.8294629	2.5093477	-1.1506349
6	1.0532246	1.0797384	-1.7541563
1	0.7777030	0.0646043	-2.0060797
6	0.1417534	2.1023985	-1.8839551
1	0.4542688	3.1030615	-1.5987944
6	-1.1886329	1.9215762	-2.3084638
6	-2.1112585	3.0824231	-2.3310294
1	-2.3299042	3.3308969	-3.3827245
1	-3.0783352	2.8104639	-1.8874113
1	-1.7043551	3.9652232	-1.8324018
6	3.3290984	0.1872375	-1.0078849
6	4.7148328	0.4172291	-0.9373821
6	2.8305124	-1.1046565	-0.7600543
6	5.5876243	-0.6264344	-0.6453446
1	5.0887018	1.4204228	-1.1166615
6	3.7065544	-2.1430794	-0.4457442
1	1.7651753	-1.3004343	-0.7560926
6	5.0844627	-1.9100475	-0.3957605
1	6.6579209	-0.4431383	-0.6049303
1	3.3067118	-3.1311185	-0.2354312
1	5.7654823	-2.7233358	-0.1601127
8	-0.5350828	-0.7888250	-0.1069950
16	-0.7735031	-2.1628247	0.4495616

8	-1.1167890	-2.1829869	1.8844745
8	0.2062816	-3.1644012	-0.0155405
6	-2.3526420	-2.6466868	-0.4087548
9	-3.3610716	-1.8318719	-0.0510130
9	-2.6952570	-3.9048991	-0.0948115
9	-2.2058419	-2.5653723	-1.7430199
6	-1.7413588	0.6014248	-2.7099201
1	-2.6737742	0.7145333	-3.2691212
1	-1.0325708	0.0022062	-3.2890573
1	-1.9544596	0.0313805	-1.7948064

XXIIIa

E (BS2) = -2423.76092295 au

H (BS2) = -2423.383253 au

G (BS2) = -2423.493466 au

E (BS3//BS2) = -3868.88930448 au

Cu	0.59354600	1.63102500	-1.20365300
Cl	0.15128700	4.00962900	-0.85302600
C	-2.13161100	0.47467200	-2.27251200
N	-1.09655900	0.87861000	-1.94298000
C	-3.43242600	-0.03252100	-2.67554700
H	-3.35956800	-1.10143800	-2.89681500
H	-4.14851400	0.11742400	-1.86173100
H	-3.77612000	0.50165000	-3.56728500
C	3.64241500	0.98686500	-0.84654800
N	2.53531900	1.27815700	-1.02433700

C	5.02512500	0.60729900	-0.60786900
H	5.69631400	1.32274300	-1.09318900
H	5.22093300	0.60207400	0.46932900
H	5.20450700	-0.39404600	-1.01065900
C	-2.46726400	0.99037300	1.58487800
O	-2.91769800	2.11471800	1.79422500
C	-1.02243600	0.73128600	1.92052500
H	-0.72531900	-0.27486400	2.18517700
C	-0.12696400	1.75663300	1.90332900
H	-0.46222900	2.74356300	1.60061000
C	1.26305600	1.58823200	2.15605200
C	2.12654100	2.77321400	1.99004000
H	1.89946400	3.45735600	2.82641300
H	3.19051700	2.53218200	2.01109900
H	1.84877100	3.32047100	1.07823100
C	-3.29010900	-0.11778900	1.04810600
C	-4.68407000	0.06264300	0.96339500
C	-2.71560300	-1.31563400	0.58744900
C	-5.48925200	-0.94814500	0.44830400
H	-5.11697600	0.99461700	1.31256100
C	-3.52612500	-2.31865800	0.05701000
H	-1.64306200	-1.46192200	0.59743500
C	-4.91060200	-2.14202800	-0.00539000
H	-6.56542800	-0.81033600	0.39581500
H	-3.06625800	-3.23119900	-0.31037300

H	-5.54127900	-2.92797300	-0.41199000
O	0.53778300	-0.82962400	0.11273100
S	0.88345800	-2.10643000	-0.56757600
O	1.40342100	-1.97281200	-1.94407200
O	-0.09720500	-3.19529200	-0.35236600
C	2.36913400	-2.66799600	0.40259600
F	3.36939200	-1.76221800	0.32119400
F	2.83732900	-3.83970900	-0.05595900
F	2.06295200	-2.81856000	1.70481700
C	1.89890000	0.32712400	2.56989900
H	2.54118600	0.53262200	3.43828800
H	1.22112000	-0.49642700	2.77891400
H	2.58086500	0.03063400	1.76107000

XXIVa

E (BS2) = -1462.16134152 au

H (BS2) = -1461.820959 au

G (BS2) = -1461.910327 au

E (BS3//BS2) = -2906.93552874 au

Cu	1.48557600	-1.34017100	0.35235400
Cl	2.35335700	-2.25437900	-1.61948500
C	-1.52432800	-1.93519700	1.14090600
N	-0.42423900	-1.69477300	0.86451200
C	-2.90435600	-2.23277200	1.48336700
H	-3.41737300	-1.30850800	1.76624100
H	-3.41005800	-2.67640300	0.62047800

H	-2.93008700	-2.93620900	2.32210000
C	3.53790900	0.46679200	1.95274100
N	2.77337000	-0.22930500	1.42789400
C	4.49033200	1.36012200	2.59039000
H	5.48699000	0.90744100	2.58016300
H	4.51801400	2.30860100	2.04303000
H	4.18748200	1.54826500	3.62545600
C	-1.60410800	0.66913300	-1.33176800
O	-1.50201300	-0.06320100	-2.31273100
C	-0.42418800	1.52634900	-0.95778000
H	-0.59488900	2.42168700	-0.37105900
C	0.82049800	1.18423800	-1.38465000
H	0.94330900	0.27730200	-1.97076000
C	2.00385500	1.90188800	-1.03898300
C	3.28566400	1.36879100	-1.52909000
H	3.33768100	1.63253300	-2.60201100
H	4.14944000	1.80688800	-1.02606800
H	3.29700800	0.27116700	-1.49654200
C	-2.84292500	0.75573600	-0.52401300
C	-3.99298400	0.10444800	-1.00982400
C	-2.89650100	1.42213000	0.71457500
C	-5.17804600	0.13789500	-0.28405300
H	-3.93840200	-0.41689300	-1.95980200
C	-4.08418100	1.44119200	1.44571400
H	-2.01914700	1.90985500	1.12617800

C	-5.22581800	0.80807100	0.94532000
H	-6.06430100	-0.35760600	-0.66942700
H	-4.11904800	1.95281600	2.40284500
H	-6.15172200	0.83326600	1.51302600
C	2.02809500	3.13205000	-0.23142400
H	2.67736300	3.86733400	-0.72670200
H	1.05528600	3.56833800	-0.01023500
H	2.54236600	2.88849900	0.71250200

4a

E (BS2) = -1000.55052941 au

H (BS2) = -1000.315923 au

G (BS2) = -1000.372862 au

E (BS3//BS2) = -1000.79917271 au

C	-0.81485200	0.87737600	0.02227900
O	-0.69576600	2.09792500	-0.09786600
C	0.37984700	0.00799900	0.21178000
C	1.61374900	0.52798300	0.14196100
C	-2.17451100	0.24805700	-0.00696700
C	-3.29224600	1.09956100	0.06575400
C	-2.38276900	-1.13750200	-0.12395300
C	-4.58371400	0.58165700	0.03495700
H	-3.12631400	2.16868300	0.15008100
C	-3.67877800	-1.65496500	-0.16504400
H	-1.54490500	-1.82173500	-0.20281700
C	-4.78018300	-0.79957200	-0.08123900

H	-5.43785300	1.25020600	0.09980300
H	-3.82699300	-2.72678700	-0.26319100
H	-5.78781200	-1.20571000	-0.10775800
C	3.73944800	0.47297500	1.43712600
H	4.72081700	-0.00364700	1.52132100
H	3.87513000	1.53559400	1.21584900
C	2.89673500	-0.21753200	0.36534700
H	3.22230800	0.38024600	2.40002100
Cl	3.84039400	-0.02493500	-1.27032000
H	0.23143400	-1.04365400	0.42317600
H	1.72146600	1.59122200	-0.06370100
C	2.75539600	-1.71212200	0.61820500
H	2.21830100	-1.86462100	1.56170600
H	2.20297600	-2.20817400	-0.18437700
H	3.74214600	-2.17461700	0.70704000

1s

E (BS2) = -502.19484277 au

H (BS2) = -501.972715 au

G (BS2) = -502.027092 au

E (BS3//BS2) = -502.396642729 au

C	0.14986800	0.67759400	-0.02944900
O	0.38678500	1.88142500	-0.03318100
C	1.27647000	-0.34163800	-0.05090100
H	1.14111300	-0.98505200	-0.93192300
C	2.67171500	0.28921300	-0.06102100

H	2.73924500	0.97972200	-0.92220700
C	3.77023900	-0.72271800	-0.11055800
H	3.58362600	-1.66276500	-0.62860900
C	5.18119200	-0.33220400	0.17907000
H	5.25038400	0.29062400	1.08298400
H	5.83037500	-1.20567500	0.31438900
H	5.62039300	0.26894000	-0.63956300
C	-1.26692000	0.18399200	-0.00463300
C	-2.30435500	1.13232700	0.01050600
C	-1.59206000	-1.18286700	0.00335000
C	-3.63629700	0.72641600	0.03314300
H	-2.04533200	2.18640300	0.00401400
C	-2.92736000	-1.58936200	0.02609700
H	-0.81062700	-1.93552800	-0.00785600
C	-3.95068900	-0.63748900	0.04109100
H	-4.43001000	1.46862800	0.04461400
H	-3.16827400	-2.64884700	0.03226200
H	-4.98949900	-0.95629200	0.05888600
H	1.16260100	-1.00573800	0.81684900
H	2.79090300	0.93460100	0.82143700

$TS_{1s\text{-}XVIIs}{}^{\alpha}$

E (BS2) = -1837.26075895 au

H (BS2) = -1836.882749 au

G (BS2) = -1836.966164 au

E (BS3//BS2) = -1837.77100970 au

S	0.56391100	-2.06810900	0.16875500
O	1.08525100	-3.26085500	0.84481200
O	-0.48125900	-2.17761900	-0.85545300
N	-0.25053900	-1.11760900	1.40109100
Cl	0.77050700	-0.80294300	2.78862800
C	1.91574200	-1.11062300	-0.46588900
C	1.70545400	-0.30175500	-1.58636600
C	3.14679100	-1.13754400	0.19893700
C	2.75316400	0.49356600	-2.04300700
H	0.74548100	-0.29743700	-2.09098900
C	4.17408600	-0.32199200	-0.26689600
H	3.29677600	-1.78226700	1.05761100
C	3.99418500	0.50571600	-1.38788200
H	2.60091100	1.12457200	-2.91383200
H	5.13185900	-0.32680700	0.24639400
C	5.12139900	1.37591600	-1.88111200
H	5.85269300	0.77884500	-2.44216200
H	4.75732800	2.16665100	-2.54439000
H	5.65632500	1.83961900	-1.04486500
C	-2.35489200	1.28999500	0.78457500
O	-2.79301300	1.82236000	1.80398500
C	-0.87565300	1.31214700	0.52689600
H	-0.57058200	0.07450800	0.89422900
H	-0.58281000	1.29009700	-0.52403000
C	-0.05992300	2.23640800	1.39980900

H	-0.14606100	1.91324300	2.44476600
H	-0.53748100	3.22887700	1.37404800
C	1.41258100	2.36919000	0.98220400
H	1.84794700	1.37462000	0.83256300
C	-3.27035500	0.62433100	-0.19058800
C	-4.65460900	0.69584200	0.05086200
C	-2.80857400	-0.06753500	-1.32273300
C	-5.55679500	0.09378600	-0.82104000
H	-5.00429500	1.22857500	0.92929800
C	-3.71460000	-0.67674000	-2.19194200
H	-1.74865400	-0.15755700	-1.52409800
C	-5.08753500	-0.59473300	-1.94655500
H	-6.62386000	0.15664500	-0.62606300
H	-3.34617500	-1.21558600	-3.06039800
H	-5.79088600	-1.06648400	-2.62755200
C	1.60498400	3.21721000	-0.27925800
H	1.23067600	4.23711900	-0.12233700
H	2.66457400	3.28317200	-0.55182500
H	1.07158100	2.79440200	-1.13843500
H	1.96670300	2.82802200	1.81081400

TS'$_{1s\text{-}XVIIs}$$^{\alpha}$

E (BS2) = -1837.26197425 au

H (BS2) = -1836.883598 au

G (BS2) = -1836.968358 au

E (BS3//BS2) = -1837.77093863 au

S	-0.68522200	-1.96041600	0.17599800
O	0.40694700	-1.91335700	-0.80354200
O	-1.17039200	-3.23780500	0.71016500
N	0.04041100	-1.11997400	1.53297500
Cl	-1.11052400	-0.72569400	2.78937600
C	-2.06626500	-1.01682600	-0.42000200
C	-3.33652400	-1.24974200	0.11571800
C	-1.84960300	-0.03734400	-1.39569800
C	-4.40525000	-0.47980400	-0.33799000
H	-3.48718300	-2.02145000	0.86236900
C	-2.93362000	0.71738700	-1.83444000
H	-0.85793300	0.13684500	-1.79712200
C	-4.22273500	0.51152700	-1.31469100
H	-5.39648800	-0.65229600	0.07209600
H	-2.77711600	1.48045200	-2.59221100
C	-5.38734300	1.32275200	-1.82128000
H	-5.74520300	0.92207900	-2.77923400
H	-6.22665800	1.30304400	-1.11898900
H	-5.09919400	2.36547200	-1.99429700
C	1.65930100	1.01249400	-0.53530900
O	1.14551400	1.49415900	-1.54692000
C	1.00456700	1.22141600	0.79715700
H	1.70720900	1.28900600	1.63128700
C	-0.14352800	2.19591600	0.86691400
H	-0.66438100	2.05963000	1.82324900

H	-0.85470500	1.98243200	0.06407600
C	0.30715300	3.67403300	0.75505100
H	0.52374700	0.03319300	1.07629900
C	2.93543000	0.24228500	-0.61131300
C	3.65480400	0.26998600	-1.81852200
C	3.42630600	-0.51934400	0.46291100
C	4.84584000	-0.43942000	-1.94741900
H	3.26551700	0.85479600	-2.64577400
C	4.61389100	-1.23876400	0.32780900
H	2.87871900	-0.57666900	1.39749300
C	5.32791200	-1.19631400	-0.87314600
H	5.39820600	-0.40678000	-2.88246600
H	4.98117000	-1.83255700	1.16010100
H	6.25599700	-1.75266900	-0.97331200
C	1.23429100	4.13284400	1.88299900
H	2.19175600	3.59891000	1.86614700
H	1.45216600	5.20400200	1.79457300
H	0.77439200	3.96443300	2.86542800
H	0.79471100	3.81598200	-0.21671100
H	-0.59946700	4.29295400	0.75070600

$TS_{1s\text{-}XVIIs}{}^{\beta}$

E (BS2) = -1837.25698980 au

H (BS2) = -1836.879134 au

G (BS2) = -1836.963328 au

E (BS3//BS2) = -1837.25698980 au

S	0.85593600	-0.29349400	-1.59077700
O	-0.36258100	-1.01193800	-1.19225700
O	1.25960900	-0.22942900	-3.00026800
N	0.48128600	1.33591700	-1.10142500
Cl	1.86731200	2.41183300	-1.27197600
C	2.23334000	-0.87538800	-0.62529400
C	3.53351200	-0.66580000	-1.09440700
C	1.99426600	-1.49535500	0.60589500
C	4.60615900	-1.08299300	-0.30878200
H	3.70233500	-0.19490600	-2.05639500
C	3.08082100	-1.90535300	1.37343900
H	0.98047100	-1.66315700	0.95160700
C	4.39939400	-1.70484400	0.93213800
H	5.62003100	-0.92374700	-0.66575000
H	2.90389500	-2.39129100	2.32926300
C	5.56464700	-2.17882100	1.76246900
H	5.76205600	-3.24273000	1.57374700
H	6.47870500	-1.62615400	1.52288100
H	5.36036600	-2.07142600	2.83324000
C	-2.52690000	0.71475300	0.49643100
O	-2.50378400	1.78021400	-0.10700700
C	-1.40309800	0.31361600	1.44736100
H	-1.10348100	-0.71221400	1.21781700
H	-1.83020800	0.28629900	2.46428500
C	-0.19705800	1.22508500	1.45467400

H	0.16217700	1.29972900	0.17326100
C	-3.67343900	-0.23396100	0.32214800
C	-4.65892700	0.08489900	-0.62838500
C	-3.80265800	-1.41698200	1.06919300
C	-5.74638100	-0.76031300	-0.83270000
H	-4.55328000	1.00076100	-1.20105700
C	-4.89632200	-2.26067700	0.86782000
H	-3.06000100	-1.68602100	1.81273300
C	-5.86749900	-1.93631400	-0.08338000
H	-6.49965600	-0.50596000	-1.57334800
H	-4.98861000	-3.17178300	1.45244900
H	-6.71578700	-2.59703100	-0.24117200
C	-0.37263500	2.65657400	1.91992200
H	-0.93363400	3.21943800	1.16574600
H	-1.02296700	2.63225000	2.81053300
H	0.68812500	0.73122700	1.86886200
C	0.94219300	3.35948300	2.26640000
H	1.47980400	2.81965400	3.05586800
H	1.60331300	3.42448700	1.39589600
H	0.75560200	4.37946000	2.62210600

TS'$_{1s\text{-}XVIIs}$$^{\beta}$

E (BS2) = -1837.25727236 au

H (BS2) = -1836.879091 au

G (BS2) = -1836.964180 au

E (BS3//BS2) = -1837.76758857 au

S	2.27791000	-1.71310000	-0.66814000
O	2.37231500	-2.56357800	0.52676100
O	2.77514200	-2.18107900	-1.96749200
N	0.55237100	-1.53714100	-0.82430000
Cl	0.09468500	-0.37684400	-2.06714500
C	3.00024200	-0.12475200	-0.31869000
C	3.32378200	0.73055500	-1.37796600
C	3.20908300	0.25198500	1.01133800
C	3.85332200	1.98508900	-1.08853500
H	3.17349200	0.41741700	-2.40507900
C	3.74414900	1.51091200	1.27726100
H	2.96818200	-0.42966300	1.81973300
C	4.06970600	2.39547100	0.23747000
H	4.10583600	2.65701000	-1.90451100
H	3.91320000	1.80946400	2.30823400
C	4.66861900	3.74772000	0.52880900
H	5.75597400	3.72690000	0.37583400
H	4.26284600	4.51382900	-0.14118300
H	4.48447700	4.05455000	1.56315000
C	-2.60076000	-0.40689900	0.32896900
O	-2.47223800	-1.58701500	0.02363700
C	-1.48969900	0.34500000	1.05460600
H	-1.91519400	0.79594300	1.96574200
C	-0.28310600	-0.48218000	1.43477800
H	0.58292400	0.14796900	1.65666100

C	-0.43736800	-1.63833000	2.40016500
H	0.11416700	-1.05078000	0.30283300
C	-3.84998200	0.34061200	-0.02139100
C	-4.87146200	-0.35305400	-0.69394000
C	-4.03647200	1.69818600	0.29046400
C	-6.05374000	0.29286200	-1.04529100
H	-4.71949200	-1.40077100	-0.93288900
C	-5.22180500	2.34481000	-0.06299400
H	-3.26382600	2.25879100	0.80570700
C	-6.23129800	1.64495100	-0.72972600
H	-6.83702800	-0.25313900	-1.56393900
H	-5.35656200	3.39468300	0.18196400
H	-7.15341200	2.15089200	-1.00327900
C	-0.67191700	-1.14384800	3.84133100
H	-1.60050500	-0.56681200	3.91882300
H	-0.74527700	-1.99732300	4.52581500
H	0.15386900	-0.50628600	4.17965200
H	-1.26590100	-2.27824900	2.08420700
H	0.47661200	-2.24356800	2.37254200
H	-1.18668200	1.19391000	0.42856700

$TS_{1s\text{-}XVIIs}{}^{\gamma}$

E (BS2) = -1837.25914820 au

H (BS2) = -1836.880587 au

G (BS2) = -1836.963894 au

E (BS3//BS2) = -1837.76869836 au

S	2.62448300	-0.78239900	1.21512900
O	1.53189100	-1.05718900	2.15857000
O	4.02533100	-0.90794900	1.63635300
N	2.35687200	-2.00981900	0.00817100
Cl	3.47441600	-1.88439800	-1.34923900
C	2.38640800	0.82039100	0.48215300
C	3.43347700	1.41066700	-0.23364400
C	1.14625000	1.45206900	0.61731200
C	3.21758200	2.64711000	-0.83836700
H	4.39728800	0.92013300	-0.31011100
C	0.95600600	2.68927800	0.00675500
H	0.34444200	0.99893300	1.18940800
C	1.98045800	3.30257700	-0.73224000
H	4.02344700	3.11186900	-1.39992800
H	-0.00597300	3.18502400	0.10713700
C	1.76330100	4.65173300	-1.36857200
H	1.91335100	5.45236800	-0.63175000
H	2.46372200	4.82478700	-2.19183800
H	0.74159300	4.75087000	-1.75108700
C	-2.42961800	0.01519500	0.88392400
O	-1.97921800	0.94297300	1.55402200
C	-1.53727500	-1.15790100	0.52011000
H	-0.72701400	-1.19339200	1.25281600
H	-2.08766000	-2.10150700	0.57571600
C	-0.94675700	-0.97019200	-0.89488400

H	-0.50025200	0.02668000	-0.98031000
H	-1.75969800	-1.00583700	-1.63836000
C	0.07276100	-2.01160200	-1.29362200
H	1.15494600	-1.88292200	-0.53314200
C	-3.83917400	0.06267000	0.38328400
C	-4.61103200	1.20291000	0.67081800
C	-4.41378800	-0.97829600	-0.36638000
C	-5.92527400	1.30078200	0.22288100
H	-4.16044700	2.00392400	1.24804400
C	-5.73104700	-0.87829600	-0.81721000
H	-3.84179500	-1.86915400	-0.60293800
C	-6.48845100	0.25869500	-0.52352100
H	-6.51233500	2.18599800	0.45191700
H	-6.16561200	-1.68865200	-1.39582400
H	-7.51431000	0.33378000	-0.87421100
C	-0.26589100	-3.47137300	-1.11469500
H	-1.15260000	-3.72204500	-1.71824900
H	0.55817800	-4.11178400	-1.44590900
H	-0.49708200	-3.71518100	-0.07228000
H	0.54126500	-1.78321400	-2.25748000

$TS_{1s\text{-}XVIIs}{}^{\delta}$

E (BS2) = -1837.25109746 au

H (BS2) = -1836.872798 au

G (BS2) = -1836.957195 au

E (BS3//BS2) = -1837.76274666 au

S	-2.80584300	-1.43408400	-0.93794600
O	-1.70980900	-1.66819600	-1.88754500
O	-4.15829600	-1.93189200	-1.21510700
N	-2.24415700	-2.30429000	0.46483400
Cl	-3.30696600	-2.11357900	1.85667300
C	-2.89943600	0.30019900	-0.55702400
C	-4.02497700	0.80058800	0.10545500
C	-1.83379800	1.13250200	-0.91429000
C	-4.06437400	2.15545800	0.42864500
H	-4.85524100	0.14974300	0.35522600
C	-1.89682800	2.48355300	-0.58535500
H	-0.97994200	0.73153500	-1.44682800
C	-3.00586800	3.01441900	0.09382400
H	-4.93293100	2.55270900	0.94670200
H	-1.07236300	3.13636400	-0.85967800
C	-3.06641400	4.48378800	0.42383900
H	-3.36562000	5.06347500	-0.45972600
H	-3.79300500	4.68773700	1.21655300
H	-2.08728700	4.85951100	0.74080800
C	3.11411600	1.12786100	-0.27339000
O	2.89187600	2.16947600	0.33822600
C	1.36956600	-0.71736700	-0.46083700
H	0.58790200	-1.09431800	-1.12935200
H	2.10452700	-1.52282400	-0.34384900
C	0.75686900	-0.39151100	0.90741000

H	0.09446200	0.48010400	0.83598400
H	1.55570100	-0.09448400	1.60950100
C	0.03023300	-1.54577800	1.53556500
H	-1.09155600	-1.85803700	0.85449400
C	4.43613500	0.43524800	-0.13267700
C	5.35102600	0.93408800	0.81159600
C	4.79418400	-0.68481100	-0.90230700
C	6.59222300	0.32814500	0.98613000
H	5.06782300	1.79974600	1.40195700
C	6.04007200	-1.29044400	-0.72896700
H	4.11046900	-1.08700900	-1.64218500
C	6.93958900	-0.78723200	0.21463800
H	7.28987600	0.72101700	1.72083700
H	6.30779200	-2.15426500	-1.33115500
H	7.90820300	-1.26138400	0.34916200
H	-0.42024400	-1.34838200	2.51193000
H	0.53323400	-2.51607000	1.48603700
C	2.02824200	0.50635000	-1.13552200
H	2.42986700	0.21171400	-2.11136000
H	1.27583000	1.28389500	-1.30017800

TS'$_{\mathbf{1s\text{-}XVIIs}}$ $^{\delta}$

E (BS2) = -1837.25543158 au

H (BS2) = -1836.876791 au

G (BS2) = -1836.959486 au

E (BS3//BS2) = -1837.76450902 au

S	-2.95258700	-1.08435500	-0.30801600
O	-2.43146100	-1.89341100	-1.41781300
O	-4.38712100	-1.05895900	-0.00119400
N	-2.16543700	-1.83514800	1.05938000
Cl	-2.38927000	-0.93323200	2.55509600
C	-2.36164700	0.58542700	-0.45593100
C	-3.03751800	1.61352200	0.20742500
C	-1.20054500	0.83249700	-1.19644300
C	-2.53213200	2.91032800	0.12481200
H	-3.94186100	1.40608900	0.76865100
C	-0.71507100	2.13441100	-1.26517900
H	-0.68212400	0.03123500	-1.70971900
C	-1.36717300	3.18988900	-0.60543100
H	-3.05011800	3.71608800	0.63763200
H	0.19122200	2.32330600	-1.83182600
C	-0.82275400	4.59225000	-0.70012800
H	-0.96913800	4.99612500	-1.71046100
H	-1.31680600	5.26574900	0.00684600
H	0.25529800	4.60882000	-0.50109100
C	2.53814700	-0.44918100	-1.42278900
O	1.74964100	0.23507600	-2.07227600
C	2.22855700	-2.92631600	-0.75291200
H	2.40808400	-3.92569000	-1.16675800
H	2.80563200	-2.86076700	0.17600200
C	0.71373300	-2.80237700	-0.43404900

H	0.36420500	-3.77043800	-0.05543000
H	0.16068400	-2.59827500	-1.35839000
C	0.41415700	-1.74493700	0.59666800
H	-0.90547500	-1.81316400	0.81859500
C	3.26219800	0.13989700	-0.25249100
C	2.81261300	1.37023500	0.25996200
C	4.36276500	-0.49083600	0.35195900
C	3.44257700	1.95295100	1.35609600
H	1.95752900	1.84971800	-0.20482100
C	5.00093100	0.10045400	1.44362900
H	4.73437200	-1.43537100	-0.03066500
C	4.54080700	1.31919100	1.94954000
H	3.08058400	2.89873400	1.75004500
H	5.85572800	-0.39130500	1.89949000
H	5.03502800	1.77433200	2.80368700
H	0.52438100	-0.70087100	0.30347000
H	0.79294700	-1.94094300	1.60367300
C	2.75366900	-1.90757000	-1.78844700
H	3.82450700	-2.08838800	-1.94634100
H	2.24400500	-2.06498300	-2.74410700

XVIIs$^{\gamma cy}$

E (BS2) = -502.217011107 au

H (BS2) = -501.992244 au

G (BS2) = -502.040810 au

E (BS3//BS2) = -502.415278329 au

C	-0.48028300	0.31218500	-0.10988400
O	-1.32612200	-0.75436900	-0.19575700
C	-1.23921700	1.60911200	-0.10822300
H	-1.19813500	2.09169600	-1.09699700
C	-2.66864000	1.14059000	0.22036300
H	-2.81538500	1.10042800	1.30628500
C	-2.69743200	-0.27900800	-0.35508400
C	-3.62985700	-1.25836100	0.32861700
H	-3.53174500	-2.25985500	-0.10436500
H	-3.40662400	-1.31460500	1.40010900
C	0.90987100	0.09008100	-0.04560100
C	1.45798100	-1.23041000	-0.06171600
C	1.82642100	1.18332600	0.03859200
C	2.82937400	-1.43139900	0.00651100
H	0.78370700	-2.07848900	-0.12756000
C	3.19488600	0.96055900	0.10557600
H	1.44591700	2.20084000	0.04710900
C	3.71545800	-0.34432100	0.09127800
H	3.22025500	-2.44650500	-0.00673500
H	3.87018700	1.81095000	0.16837500
H	4.78784300	-0.51029100	0.14335800
H	-0.84501800	2.32880400	0.61696800
H	-3.44624800	1.77507100	-0.21270400
H	-4.66800000	-0.93004000	0.20442100
H	-2.89915800	-0.24718100	-1.43477900

XVIIs$^{\alpha}$

E (BS2) = -502.210086350 au

H (BS2) = -501.986213 au

G (BS2) = -502.039623 au

E (BS3//BS2) = -502.410890301 au

C	-0.18759100	0.65006700	0.11469100
O	-0.45607300	1.83443600	-0.18533600
C	-1.22620300	-0.24457600	0.55595500
H	-0.97533000	-1.25566000	0.86299300
C	-2.65746200	0.15029600	0.59562700
H	-2.75484100	1.21938800	0.38193500
H	-3.05283500	-0.02992200	1.60754000
C	-3.51514400	-0.66548500	-0.40340600
H	-3.13754500	-0.49320500	-1.41952000
H	-3.39082700	-1.73685400	-0.19767300
C	-4.99606800	-0.28679300	-0.32520400
H	-5.14332400	0.77753600	-0.54783700
H	-5.58937300	-0.86587800	-1.04309400
H	-5.39953800	-0.47752400	0.67734300
C	1.23551800	0.17171000	0.04136400
C	2.25201800	1.13868900	-0.04258300
C	1.59258100	-1.18745000	0.02947400
C	3.59084500	0.76058100	-0.11971500
H	1.97280900	2.18750700	-0.04364200
C	2.93362500	-1.56643100	-0.05678800

H	0.83003100	-1.95883600	0.06542000
C	3.93619100	-0.59539100	-0.12666800
H	4.36578200	1.52058800	-0.17562600
H	3.19404000	-2.62133500	-0.07242400
H	4.97967400	-0.89259200	-0.18922100

XVIIs$^{\gamma}$

E (BS2) = -502.194842770 au

H (BS2) = -501.972715 au

G (BS2) = -502.027092 au

E (BS3//BS2) = -502.396642729 au

C	0.14986800	0.67759400	-0.02944900
O	0.38678500	1.88142500	-0.03318100
C	1.27647000	-0.34163800	-0.05090100
H	1.14111300	-0.98505200	-0.93192300
C	2.67171500	0.28921300	-0.06102100
H	2.73924500	0.97972200	-0.92220700
C	3.77023900	-0.72271800	-0.11055800
H	3.58362600	-1.66276500	-0.62860900
C	5.18119200	-0.33220400	0.17907000
H	5.25038400	0.29062400	1.08298400
H	5.83037500	-1.20567500	0.31438900
H	5.62039300	0.26894000	-0.63956300
C	-1.26692000	0.18399200	-0.00463300
C	-2.30435500	1.13232700	0.01050600
C	-1.59206000	-1.18286700	0.00335000

C	-3.63629700	0.72641600	0.03314300
H	-2.04533200	2.18640300	0.00401400
C	-2.92736000	-1.58936200	0.02609700
H	-0.81062700	-1.93552800	-0.00785600
C	-3.95068900	-0.63748900	0.04109100
H	-4.43001000	1.46862800	0.04461400
H	-3.16827400	-2.64884700	0.03226200
H	-4.98949900	-0.95629200	0.05888600
H	1.16260100	-1.00573800	0.81684900
H	2.79090300	0.93460100	0.82143700

XVIIIs$^{\gamma}$

E (BS2) = -2385.62131803 au

H (BS2) = -2385.251224 au

G (BS2) = -2385.362302 au

E (BS3//BS2) = -3830.74393818 au

Cu	1.51427400	-1.31627600	0.23272700
Cl	1.52869900	-3.63495200	0.03732600
C	0.34379600	-1.44845100	3.17649700
N	0.78411800	-1.41091900	2.10869700
C	-0.21920300	-1.51579000	4.51223100
H	-0.10221500	-0.54641100	5.00745200
H	-1.28322700	-1.76449500	4.44284000
H	0.29977200	-2.28745200	5.08955800
C	3.19750200	-0.86931100	-2.39403600
N	2.53917900	-1.10342000	-1.47424100

C	4.03171500	-0.56624100	-3.54086300
H	4.00151400	-1.39897100	-4.25001500
H	3.66352500	0.34395300	-4.02516700
H	5.06191600	-0.40842300	-3.20500400
C	-3.60785000	-0.06804900	0.03221500
O	-2.80380200	0.55133000	0.72207900
C	-3.23883400	-1.40319300	-0.59676400
H	-3.89909700	-2.16813200	-0.16257500
H	-3.47661200	-1.37630200	-1.66754000
C	-1.75988600	-1.79271600	-0.38063000
H	-1.63764500	-2.84603300	-0.66054200
H	-1.53265900	-1.70649200	0.68735700
C	-0.81821900	-0.94399900	-1.17919900
H	-0.67367800	0.08032400	-0.84501800
C	-0.55237300	-1.26107400	-2.61422600
H	-1.46968200	-1.15998800	-3.22200900
H	0.20078400	-0.59510100	-3.04748200
H	-0.21188100	-2.29826000	-2.74000600
C	-4.98780300	0.47440600	-0.19080800
C	-5.31272600	1.72323000	0.36699800
C	-5.96218100	-0.21774100	-0.92954100
C	-6.58054500	2.27098600	0.18897300
H	-4.55500000	2.25135500	0.93714900
C	-7.23367800	0.33081700	-1.10565600
H	-5.73815400	-1.18502200	-1.36716800

C	-7.54481800	1.57456300	-0.54891600
H	-6.81993400	3.23798200	0.62313000
H	-7.98068600	-0.21245500	-1.67784300
H	-8.53480400	2.00051900	-0.68907000
O	1.43959500	0.71253000	0.38155000
S	2.51833500	1.55707800	1.04669700
O	3.67304100	0.77341300	1.51567500
O	1.95686700	2.56839800	1.95254100
C	3.15648900	2.53027200	-0.40677300
F	3.80650800	1.73202900	-1.26791800
F	4.00783600	3.47240900	0.02041100
F	2.14516600	3.12235000	-1.05380000

XVIIIs$^{\alpha}$

E (BS2) = -2385.64658750 au

H (BS2) = -2385.274393 au

G (BS2) = -2385.384005 au

E (BS3//BS2) = -3830.76521007 au

Cu	0.12470500	-0.21791800	1.48625100
Cl	0.81474700	1.02306400	3.34163700
C	2.75457200	-1.97076600	1.62260900
N	1.79590400	-1.32836900	1.56055500
C	3.96708200	-2.76469200	1.68475600
H	4.83576000	-2.09958300	1.64716700
H	3.98322400	-3.33493500	2.61911000
H	3.99573300	-3.45360400	0.83463700

C	-2.63932400	1.26879200	1.40469700
N	-1.61481300	0.73808900	1.44945600
C	-3.93488500	1.91731700	1.34059600
H	-4.57038500	1.53848000	2.14776100
H	-3.80824600	2.99848300	1.44958700
H	-4.39981900	1.69866600	0.37467900
C	1.92699300	1.74530800	-0.74171700
O	2.80504600	2.50566700	-0.27834900
C	2.25881800	0.39134200	-1.10665000
H	1.48205200	-0.28194500	-1.45118000
C	3.66073400	-0.10071400	-1.06219300
H	3.66927900	-1.18361000	-0.89111500
H	4.19956900	0.38642800	-0.24110800
C	4.41868200	0.19172600	-2.38446800
H	3.87210900	-0.25857200	-3.22304600
C	5.85105100	-0.34544300	-2.34451200
H	5.86052500	-1.43245700	-2.19398800
H	6.37867500	-0.13079800	-3.28167400
H	6.42004100	0.11094000	-1.52471600
C	0.51918100	2.24351100	-0.90679300
C	0.16433700	3.44545800	-0.27000800
C	-0.44215900	1.58140900	-1.68929000
C	-1.11714400	3.97288000	-0.40998400
H	0.91042500	3.95482600	0.33141200
C	-1.72170800	2.11691700	-1.84071700

H	-0.20450300	0.64917800	-2.18647100
C	-2.06329300	3.31193400	-1.20285400
H	-1.37996800	4.89942500	0.09351200
H	-2.45303900	1.59133200	-2.44629500
H	-3.06145200	3.72539600	-1.31859100
O	-0.41655800	-1.29081000	-0.17620200
S	-1.54249900	-2.31227600	-0.22683400
O	-2.29931400	-2.41031700	1.03083000
O	-1.13305400	-3.55782600	-0.88969500
C	-2.71222300	-1.51088600	-1.43552900
F	-3.29507100	-0.43021500	-0.89764900
F	-3.67021600	-2.38239600	-1.77709300
F	-2.05726400	-1.13580200	-2.54502900
H	4.42493300	1.27572100	-2.55188300

XVIIIs$^{\gamma cy}$

E (BS2) = -2385.64881973 au

H (BS2) = -2385.276504 au

G (BS2) = -2385.379074 au

E (BS3//BS2) = -3830.76772489 au

Cu	-1.26369200	-0.84142700	1.20142600
Cl	-1.15266800	-2.98760700	2.14987700
C	-3.90103600	-1.65149100	-0.32391200
N	-2.93561000	-1.34015400	0.22951600
C	-5.11228400	-2.04865200	-1.01638900
H	-5.28890000	-1.37477900	-1.86066000

H	-5.00160500	-3.07485800	-1.38139000
H	-5.95892000	-1.99657900	-0.32393200
C	1.49680100	0.15235200	2.23159000
N	0.48022100	-0.32447400	1.95991700
C	2.76202800	0.77838300	2.55149400
H	2.90388300	0.79447100	3.63674100
H	3.57423100	0.21784800	2.07843900
H	2.74874500	1.80009900	2.16076500
C	1.72202600	-1.06024500	-1.22583600
O	1.53773500	-2.39787300	-1.04132700
C	0.50790700	-0.42406600	-1.83623800
H	-0.08679900	0.08725000	-1.07058300
H	0.75444900	0.32444600	-2.59563800
C	-0.24597100	-1.64104200	-2.39811700
H	0.08538200	-1.86094100	-3.41984100
H	-1.33156300	-1.51030500	-2.40318300
C	0.18546400	-2.76919400	-1.45617600
H	-0.42834200	-2.76395000	-0.54804400
C	0.22540700	-4.16159700	-2.05042200
H	0.87101900	-4.18402500	-2.93576900
H	0.60155400	-4.88709600	-1.32073000
H	-0.78530000	-4.46539600	-2.34644800
C	2.94241700	-0.46928800	-0.84476900
C	4.01787200	-1.24643400	-0.31213800
C	3.14668600	0.93890300	-0.97794900

C	5.21898800	-0.64696500	0.04300400
H	3.88541000	-2.31735100	-0.19496700
C	4.35518500	1.51767600	-0.61739100
H	2.33450200	1.56285500	-1.33554500
C	5.40662900	0.73779400	-0.10632400
H	6.02377000	-1.26102800	0.44121900
H	4.48326800	2.59240200	-0.72560900
H	6.34933000	1.19926500	0.17395200
O	-1.65477100	1.06662800	0.56303700
S	-0.94609900	2.41274600	0.59440700
O	0.51604200	2.34112200	0.44453000
O	-1.48229900	3.31703900	1.62178200
C	-1.54421500	3.09309300	-1.02980400
F	-1.12369400	2.31976900	-2.04174000
F	-1.06136100	4.33013100	-1.20748000
F	-2.88199800	3.14411700	-1.05550200

MECP$^{\alpha}$

E (BS2) = -2385.62312718 au

Cu	0.26691660	-0.71776450	1.07822870
Cl	1.95753480	-0.67310610	2.61069110
C	-1.21956050	-3.58880340	1.57358870
N	-0.48143290	-2.69699250	1.56109790
C	-2.17240000	-4.68454190	1.57418150
H	-2.74934410	-4.65428750	0.64406500
H	-1.64649130	-5.64141260	1.64843440

H	-2.85231100	-4.57375990	2.42495270
C	-1.26106830	2.01799690	1.93687040
N	-0.66009410	1.05045670	1.73812900
C	-2.02380990	3.23503230	2.14597900
H	-1.94535430	3.55228080	3.19048270
H	-1.62590810	4.02014620	1.49524360
H	-3.07392090	3.05166480	1.89670280
C	2.74949380	0.67876530	-0.47235590
O	3.94082690	0.65731680	-0.13419910
C	2.08808460	-0.58734140	-0.82080940
H	1.20401030	-0.56808470	-1.45036830
C	2.78964990	-1.87938310	-0.67792590
H	2.05568850	-2.66920800	-0.47188730
H	3.51047540	-1.83344710	0.14226910
C	3.53563970	-2.23645670	-2.00053640
H	2.81658470	-2.25433220	-2.82884810
C	4.24679460	-3.58626610	-1.88750590
H	3.53286470	-4.39345460	-1.68339600
H	4.77076760	-3.82442380	-2.82125130
H	4.98653060	-3.57632960	-1.07783110
C	1.98785600	1.96236130	-0.53266490
C	2.58392250	3.10587830	0.02979680
C	0.70602670	2.07100370	-1.10012170
C	1.91285590	4.32482900	0.03369260
H	3.57378850	3.01899190	0.46634050

C	0.03758870	3.29535140	-1.10327790
H	0.21198320	1.20934760	-1.52914320
C	0.63586610	4.42307810	-0.53534530
H	2.38018720	5.19783310	0.47985400
H	-0.95578430	3.35748120	-1.53648110
H	0.11142110	5.37477930	-0.53137450
O	-0.87374210	-0.78969680	-0.58410410
S	-2.38453570	-0.96550280	-0.68494600
O	-3.07060650	-1.01640600	0.61310860
O	-2.75100700	-1.96162920	-1.70067350
C	-2.88122880	0.67018810	-1.42898630
F	-2.74710470	1.66219260	-0.53602790
F	-4.16120440	0.61874760	-1.81747140
F	-2.11806440	0.95230070	-2.49620390
H	4.25843390	-1.44085290	-2.21468220

MECP$^{\gamma}$

E (BS2) = -2385.60333438 au

Cu	1.49382160	-1.40712970	0.22052600
Cl	1.40677610	-3.70818900	0.40798710
C	0.97428410	-0.52618190	3.18878110
N	1.02103660	-1.07781690	2.17314890
C	0.93717710	0.20044130	4.44269930
H	1.29451130	1.21927900	4.25785920
H	-0.08780760	0.23252770	4.82538690
H	1.58761200	-0.29147150	5.17282690

C	3.61366290	-0.99833280	-1.99881350
N	2.67285300	-1.41156090	-1.46561340
C	4.79066540	-0.44009020	-2.63518340
H	5.49693650	-1.23644100	-2.88893770
H	4.49957190	0.09774360	-3.54258550
H	5.25739550	0.25691300	-1.93136220
C	-3.66696600	-0.01627600	0.24538620
O	-2.84453570	0.76681400	0.71032580
C	-3.30890100	-1.47332570	-0.00666630
H	-3.94819570	-2.09309310	0.63874070
H	-3.57732250	-1.73694160	-1.03730230
C	-1.82086760	-1.79043450	0.26067360
H	-1.68984730	-2.87828330	0.23095620
H	-1.57041720	-1.45197670	1.27160130
C	-0.91496880	-1.14036100	-0.74011520
H	-0.75734400	-0.06987560	-0.63420500
C	-0.73292960	-1.73990020	-2.09773690
H	-1.64359600	-1.61161360	-2.71036900
H	0.09289140	-1.26795140	-2.64120160
H	-0.53957540	-2.81853250	-2.03615820
C	-5.05553650	0.44765030	-0.07591040
C	-5.36109990	1.80909770	0.09617730
C	-6.05680680	-0.42389330	-0.53570230
C	-6.63709150	2.28991380	-0.18614100
H	-4.58242350	2.47669600	0.45067510

C	-7.33657740	0.05812470	-0.81418240
H	-5.84739540	-1.47928290	-0.67497350
C	-7.62916780	1.41347960	-0.64135540
H	-6.86113140	3.34460650	-0.05324530
H	-8.10464640	-0.62490730	-1.16676740
H	-8.62590650	1.78698690	-0.86165200
O	1.51459550	0.62416570	0.05115340
S	2.65218630	1.47786500	0.59257900
O	3.95285060	0.78478850	0.60133710
O	2.28034860	2.24611170	1.79039470
C	2.78773380	2.72142840	-0.78161990
F	3.08099480	2.09839310	-1.93393090
F	3.76332390	3.59585290	-0.50616080
F	1.63676380	3.38575720	-0.93112170

MECP^{cy}

E (BS2) = -2385.64291731 au

Cu	-1.70593640	-1.04257280	0.30822900
Cl	-2.16434420	-3.27576960	0.14497600
C	-4.36099800	-0.32534660	-1.16187960
N	-3.37692150	-0.61680530	-0.63422970
C	-5.60292180	0.03468740	-1.81486530
H	-5.59270070	1.10273850	-2.05360880
H	-5.71208810	-0.54892680	-2.73476530
H	-6.43646600	-0.18487460	-1.13919850
C	1.01559110	-1.51833910	1.71375650

N	-0.03727630	-1.41298300	1.25392000
C	2.33694770	-1.63638300	2.29020880
H	2.28094690	-2.21195820	3.21981370
H	2.99868150	-2.13741700	1.57757100
H	2.72355140	-0.63257190	2.49187730
C	2.64483010	-0.49155830	-1.06253280
O	2.41742360	-1.76969550	-1.56621760
C	1.46930440	0.41301540	-1.31631190
H	0.92683910	0.63612440	-0.39442460
H	1.75948870	1.37697440	-1.75146240
C	0.60712760	-0.42332010	-2.28095200
H	0.84298490	-0.17726800	-3.32321620
H	-0.46832520	-0.27762420	-2.13231030
C	1.03527850	-1.86742490	-1.98348420
H	0.45852380	-2.26100490	-1.13715250
C	0.94139170	-2.83026020	-3.15139690
H	1.53225450	-2.46184100	-3.99805400
H	1.31257490	-3.82273220	-2.87131710
H	-0.10266270	-2.93261390	-3.47006040
C	3.84247080	-0.21328060	-0.38568930
C	4.86919730	-1.19811270	-0.21570600
C	4.08398460	1.07580380	0.19205560
C	6.04045770	-0.90400950	0.47196990
H	4.71520100	-2.18814200	-0.63450530
C	5.26401300	1.34746230	0.87327990

H	3.32310750	1.84795990	0.10462450
C	6.26281540	0.36886840	1.02450270
H	6.79852740	-1.67761250	0.58140510
H	5.41361820	2.33847870	1.29849210
H	7.18301940	0.59084220	1.55790770
O	-1.36858600	0.88401760	0.34027750
S	-1.00147530	1.78189260	1.53079200
O	0.33392330	1.51898620	2.08095010
O	-2.11572460	1.96208370	2.46853760
C	-0.84251340	3.37186740	0.57821890
F	0.15137370	3.27711480	-0.31198930
F	-0.57133420	4.36514930	1.43198280
F	-1.98210070	3.64599290	-0.06380140

XIXs$^{\alpha}$

E (BS2) = -2385.64587679 au

H (BS2) = -2385.273049 au

G (BS2) = -2385.378065 au

E (BS3//BS2) = -3830.75744679 au

Cu	0.21760300	-0.64001400	0.80111700
Cl	1.77392400	-0.87220200	2.40681200
C	-2.13291400	-3.16495300	0.74243800
N	-1.05472900	-2.81923200	0.99635500
C	-3.49118900	-3.57107700	0.41694700
H	-4.03973400	-2.69390700	0.06007000
H	-3.47505000	-4.33552100	-0.36672400

H	-3.98448800	-3.97690900	1.30585600
C	-2.34484200	0.49814200	2.35594200
N	-1.30976600	0.08015900	2.05258800
C	-3.65400300	1.03373100	2.67933200
H	-3.64944900	1.46678900	3.68410300
H	-3.90469700	1.80740300	1.94651600
H	-4.39769200	0.23236000	2.62847300
C	3.00438700	-0.02918600	-0.22976900
O	4.12668900	-0.50809700	-0.10658700
C	1.87574200	-0.98115500	-0.55660300
H	1.21540500	-0.64922400	-1.35912700
C	2.13024500	-2.44189100	-0.52714100
H	1.17950300	-2.97735900	-0.42811000
H	2.78206800	-2.70482300	0.30791400
C	2.78579300	-2.87788800	-1.87460600
H	2.14079700	-2.57697100	-2.70896000
C	3.00678100	-4.39207300	-1.89557800
H	2.05667900	-4.93182500	-1.80328900
H	3.48018500	-4.69449400	-2.83747600
H	3.65828200	-4.70714600	-1.07171500
C	2.75240500	1.43314200	-0.13745400
C	3.75960700	2.23993800	0.42078700
C	1.55805000	2.02888100	-0.57868900
C	3.56745700	3.61197200	0.55085700
H	4.67984000	1.77405800	0.75839400

C 1.37020200 3.40488400 -0.45259200

H 0.77716800 1.43115400 -1.03191000

C 2.37120000 4.19700700 0.11530400

H 4.34487300 4.22819300 0.99342800

H 0.44388900 3.85182200 -0.79901600

H 2.22248700 5.26838800 0.21835700

O -0.85010400 -0.37518900 -0.91965000

S -2.27434500 0.02499400 -1.27026300

O -3.28950800 -0.36679600 -0.28019200

O -2.56762000 -0.23780600 -2.68466800

C -2.18595400 1.88238300 -1.14222000

F -1.74529600 2.26224100 0.06704100

F -3.39899300 2.40983600 -1.34083800

F -1.34322600 2.36908000 -2.06747200

H 3.73788100 -2.34899800 -1.98489200

XIXs$^{\gamma}$

E (BS2) = -2385.65049002 au

H (BS2) = -2385.278337 au

G (BS2) = -2385.385248 au

E (BS3//BS2) = -3830.76397729 au

Cu -1.30243100 1.61675800 -0.00880500

Cl -1.07802700 3.86065400 -0.25711200

C -0.02792800 -0.03854500 2.44066600

N -0.46013700 0.94807000 2.00937700

C 0.49772600 -1.29634000 2.94132600

H	0.37747700	-2.05701900	2.16341800
H	1.55986500	-1.18640400	3.17438700
H	-0.05386100	-1.60204100	3.83556000
C	-4.24193800	0.87427900	1.03386400
N	-3.33573700	1.49385200	0.66465000
C	-5.35328800	0.05951400	1.49155400
H	-5.80589300	0.50652300	2.38189800
H	-6.10596300	-0.02011000	0.70123000
H	-4.97010500	-0.93691100	1.73225000
C	3.41474100	0.31410700	0.21987000
O	2.53305700	-0.23576000	0.87543800
C	3.17131000	1.66691800	-0.43129800
H	3.90327700	2.38292400	-0.03644700
H	3.36605700	1.59381900	-1.50776500
C	1.75357600	2.25162100	-0.16272500
H	1.72853700	3.28513500	-0.50947400
H	1.56897900	2.22027900	0.91345900
C	0.75068700	1.42269800	-0.87782300
H	0.64401600	0.40934000	-0.49841200
C	0.48478700	1.60477600	-2.32040400
H	1.33428400	1.11656600	-2.83654800
H	-0.41538800	1.07632800	-2.64582400
H	0.45985500	2.65456500	-2.61829900
C	4.75017800	-0.33267500	0.04884000
C	4.96322000	-1.58654100	0.64922100

C	5.78915200	0.25932700	-0.68987400
C	6.18710800	-2.23575400	0.51414500
H	4.15574000	-2.03809500	1.21656900
C	7.01521200	-0.39323100	-0.82413300
H	5.65046900	1.22535000	-1.16361500
C	7.21620600	-1.63948900	-0.22401800
H	6.34169100	-3.20466000	0.98086700
H	7.81266800	0.07077800	-1.39762400
H	8.17159900	-2.14603000	-0.33156900
O	-1.48120400	-0.31721800	-0.77223400
S	-1.84718100	-1.63711400	-0.12814800
O	-2.41953000	-1.53269000	1.22523500
O	-0.82225700	-2.67352500	-0.33657300
C	-3.28445000	-2.14714800	-1.19104400
F	-4.26967100	-1.23670500	-1.10903600
F	-3.76332700	-3.32869600	-0.77826600
F	-2.91129500	-2.25622600	-2.47265000

XIXs$^{\gamma cy}$

E (BS2) = -2385.70846475 au

H (BS2) = -2385.333483 au

G (BS2) = -2385.436634 au

E (BS3//BS2) = -3830.81744250 au

Cu	1.67085800	1.02412900	1.28412700
Cl	3.86997500	1.29087300	1.91674400
C	0.08794600	-1.72359000	1.73316800

N	0.75962900	-0.81004800	1.48891500
C	-0.73832900	-2.87818100	2.03774300
H	-1.79552500	-2.59601300	1.99947100
H	-0.55571600	-3.65993800	1.29381100
H	-0.48367200	-3.25768800	3.03273600
C	-0.73865800	3.02137900	0.81269700
N	0.22169400	2.38680600	0.94687300
C	-1.95048700	3.80539100	0.64080600
H	-1.83262300	4.78067000	1.12332200
H	-2.14428600	3.94856100	-0.42669200
H	-2.79451300	3.27472100	1.09116600
O	-2.09730400	-1.79224500	-0.71319200
S	-3.42705600	-1.39718000	-0.17991400
O	-3.83524300	-2.11788700	1.04959700
O	-4.48268000	-1.20988100	-1.19600800
C	-3.11444500	0.32804900	0.43733300
F	-2.17253600	0.33519600	1.39869400
F	-4.22977000	0.87710500	0.94990500
F	-2.68917800	1.12207400	-0.56635900
C	1.69875500	-1.02348800	-1.56587800
O	2.92281800	-1.19869300	-1.21070900
C	0.93978700	-2.30196200	-1.66630100
H	0.82039400	-2.51492400	-2.73870500
C	1.85880900	-3.31576200	-0.96215900
H	1.53508700	-3.47068000	0.07031000

C	3.23943100	-2.65401400	-0.98524100
C	4.05984600	-2.74523400	0.27917600
H	4.98843000	-2.17317600	0.19065000
H	3.48624100	-2.37452100	1.13474800
C	1.24357500	0.30182600	-1.87171800
C	2.15760000	1.38401100	-1.86753600
C	-0.11467600	0.52154900	-2.18980900
C	1.71779200	2.65574200	-2.20211700
H	3.19598600	1.20981200	-1.61071300
C	-0.53953200	1.80167600	-2.52549200
H	-0.82774700	-0.29421100	-2.14711400
C	0.37225600	2.86277100	-2.53691600
H	2.41471800	3.48788600	-2.20325400
H	-1.58297200	1.97661300	-2.76412500
H	0.03202900	3.86057300	-2.79941300
H	-0.06285900	-2.21975600	-1.24034700
H	1.87725600	-4.28114200	-1.47037100
H	4.31361400	-3.79649800	0.45408100
H	3.81994700	-2.92799100	-1.86998600

XXs$^{\gamma cy}$

E (BS2) = -1424.10592379 au

H (BS2) = -1423.768452 au

G (BS2) = -1423.855326 au

E (BS3//BS2) = -2868.86108319 au

Cu	-0.09408000	1.29127000	0.57038700

Cl	-0.76323800	0.42315900	2.64866000
C	-2.45767300	2.54798900	-1.11504600
N	-1.55418400	2.08735600	-0.55280400
C	-3.59825600	3.10855100	-1.81981700
H	-3.25544700	3.66187600	-2.70008100
H	-4.26392200	2.29870100	-2.13635400
H	-4.14384900	3.78637400	-1.15518400
C	3.03807900	1.72054100	0.47491800
N	1.88349400	1.62305200	0.44742300
C	4.48705600	1.82953100	0.50532600
H	4.77638900	2.78148300	0.96231600
H	4.90384200	1.00479500	1.09206900
H	4.88060900	1.78252200	-0.51491400
C	-0.52895600	-1.64117400	-0.71185200
O	-1.06238900	-2.34641000	0.22139600
C	-1.54295400	-1.08318800	-1.65567800
H	-1.54720200	-1.73032900	-2.54531100
C	-2.84257100	-1.20185400	-0.84523900
H	-3.00336300	-0.29066300	-0.26146500
C	-2.56945600	-2.38004200	0.08686900
C	-3.15699700	-2.31807900	1.47440000
H	-2.85490300	-3.18593800	2.06900500
H	-2.83908200	-1.40132700	1.98027700
C	0.89811400	-1.50836500	-0.77532900
C	1.71208700	-2.03509800	0.25477300

C	1.48786600	-0.85876300	-1.88280500
C	3.09086600	-1.91532500	0.16831000
H	1.25149800	-2.51908700	1.10881500
C	2.87087100	-0.75989200	-1.96563200
H	0.86628800	-0.44820200	-2.67087700
C	3.66966700	-1.28544800	-0.94327700
H	3.72055700	-2.31031200	0.95912900
H	3.32931300	-0.26916900	-2.81809700
H	4.75037400	-1.19898600	-1.00803700
H	-1.29582000	-0.07094400	-1.97637800
H	-3.71616800	-1.38496100	-1.47308900
H	-4.24921600	-2.32305200	1.38638200
H	-2.75905800	-3.34119100	-0.39965100

TS$_{\mathbf{XXs\gamma cy\text{-}II}}$

E (BS2) = -1424.07099732 au

H (BS2) = -1423.736899 au

G (BS2) = -1423.818583 au

E (BS3//BS2) = -2868.82908696 au

Cu	2.73234700	-0.57010900	-0.24559200
Cl	2.61994800	1.90257400	-0.56361900
C	5.56390300	-1.00313900	1.01766400
N	4.50532500	-0.90045200	0.55706300
C	6.89206600	-1.12747000	1.59345800
H	6.86019900	-1.80973000	2.44918600
H	7.58172000	-1.52303600	0.84062900

H	7.24240300	-0.14458600	1.92503800
C	0.06672000	-1.88830000	-1.19606900
N	1.09460700	-1.45590100	-0.87882300
C	-1.22866600	-2.41332400	-1.59019700
H	-1.13076000	-3.46703000	-1.87049200
H	-1.92977300	-2.32237100	-0.75466700
H	-1.60940500	-1.84160500	-2.44182900
C	-2.34330700	0.68456900	0.16331400
O	-1.83180000	0.71123000	-0.96859200
C	-1.54175700	1.31389000	1.27607400
H	-1.65616700	0.79479600	2.23024600
C	-0.05859300	1.33196800	0.84820300
H	0.47131100	2.07032100	1.45488700
C	0.08178500	1.69675600	-0.60018900
C	-0.25334800	3.06954300	-1.07615700
H	0.23096700	3.81639200	-0.43968500
H	0.04258400	3.22112500	-2.11567800
C	-3.67617200	0.09400400	0.37636300
C	-4.32355200	-0.53229400	-0.70732900
C	-4.31282000	0.14398100	1.63004100
C	-5.57899800	-1.10530800	-0.53585100
H	-3.82891000	-0.56401500	-1.67211000
C	-5.57499500	-0.42398300	1.79352600
H	-3.83383000	0.62889400	2.47369300
C	-6.20612400	-1.05124900	0.71501600

H	-6.07153100	-1.59248500	-1.37223200
H	-6.06488400	-0.38013600	2.76166300
H	-7.18735500	-1.49849800	0.84810800
H	-1.92822100	2.33328900	1.41510800
H	0.39147800	0.35232800	1.02435400
H	-1.33479800	3.22927500	-0.98742500
H	0.39581800	0.95324400	-1.31125000

2s

E (BS2) = -962.466667756 au

H (BS2) = -962.237329 au

G (BS2) = -962.291484 au

E (BS3//BS2) = -962.699998820 au

C	0.33055100	0.67235300	0.34205600
O	-0.04692600	1.77200500	-0.05247800
C	-0.62281600	-0.26914800	1.06397800
H	-0.22398100	-0.43506400	2.07395800
C	-2.05589700	0.25585600	1.15579500
H	-2.03935900	1.27647900	1.55772500
H	-2.62748200	-0.36069700	1.85863700
C	1.74861600	0.23689900	0.13138100
C	2.61769200	1.10312700	-0.55485100
C	2.23828300	-0.99623000	0.59424900
C	3.94524800	0.74613800	-0.77618200
H	2.23248800	2.05421600	-0.90859400
C	3.56960400	-1.35271000	0.37319700

H	1.58903900	-1.68207600	1.12815300
C	4.42422100	-0.48438500	-0.31176500
H	4.60781700	1.42290600	-1.30890300
H	3.93913100	-2.30807400	0.73538700
H	5.46012200	-0.76463100	-0.48348200
H	-0.59754500	-1.24935900	0.57283600
C	-4.19147700	0.95784500	-0.03215500
H	-4.71762600	0.97594300	-0.99118000
H	-4.80181600	0.41330400	0.69656600
C	-2.81386600	0.33203200	-0.16695300
H	-4.07544900	1.99164700	0.31671700
H	-2.22149300	0.84923900	-0.92167500
Cl	-3.01702300	-1.38650200	-0.87169500
